# Global, regional, and national burden of respiratory tract cancers and associated risk factors from 1990 to 2019: a systematic analysis for the Global Burden of Disease Study 2019

**DOI:** 10.1016/S2213-2600(21)00164-8

**Published:** 2021-09

**Authors:** GBD 2019 Respiratory Tract Cancers Collaborators, Hedyeh Ebrahimi, Zahra Aryan, Sahar Saeedi Moghaddam, Catherine Bisignano, Shahabeddin Rezaei, Farhad Pishgar, Lisa M Force, Hassan Abolhassani, Eman Abu-Gharbieh, Shailesh M Advani, Sohail Ahmad, Fares Alahdab, Vahid Alipour, Syed Mohamed Aljunid, Saeed Amini, Robert Ancuceanu, Catalina Liliana Andrei, Tudorel Andrei, Jalal Arabloo, Morteza Arab-Zozani, Malke Asaad, Marcel Ausloos, Atalel Fentahun Awedew, Atif Amin Baig, Ali Bijani, Antonio Biondi, Tone Bjørge, Dejana Braithwaite, Michael Brauer, Hermann Brenner, Maria Teresa Bustamante-Teixeira, Zahid A Butt, Giulia Carreras, Carlos A Castañeda-Orjuela, Odgerel Chimed-Ochir, Dinh-Toi Chu, Michael T Chung, Aaron J Cohen, Kelly Compton, Baye Dagnew, Xiaochen Dai, Lalit Dandona, Rakhi Dandona, Frances E Dean, Meseret Derbew Molla, Abebaw Alemayehu Desta, Tim Robert Driscoll, Emerito Jose A Faraon, Pawan Sirwan Faris, Irina Filip, Florian Fischer, Weijia Fu, Silvano Gallus, Birhan Gebresillassie Gebregiorgis, Ahmad Ghashghaee, Mahaveer Golechha, Kebebe Bekele Gonfa, Giuseppe Gorini, Bárbara Niegia Garcia Goulart, Maximiliano Ribeiro Guerra, Nima Hafezi-Nejad, Samer Hamidi, Simon I Hay, Claudiu Herteliu, Chi Linh Hoang, Nobuyuki Horita, Mihaela Hostiuc, Mowafa Househ, Ivo Iavicoli, Irena M Ilic, Milena D Ilic, Seyed Sina Naghibi Irvani, Farhad Islami, Ashwin Kamath, Supreet Kaur, Rovshan Khalilov, Ejaz Ahmad Khan, Jonathan M Kocarnik, Burcu Kucuk Bicer, G Anil Kumar, Carlo La Vecchia, Qing Lan, Iván Landires, Savita Lasrado, Paolo Lauriola, Elvynna Leong, Bingyu Li, Stephen S Lim, Alan D Lopez, Azeem Majeed, Reza Malekzadeh, Navid Manafi, Ritesh G Menezes, Tomasz Miazgowski, Sanjeev Misra, Abdollah Mohammadian-Hafshejani, Shafiu Mohammed, Ali H Mokdad, Alex Molassiotis, Lorenzo Monasta, Rahmatollah Moradzadeh, Lidia Morawska, Joana Morgado-da-Costa, Shane Douglas Morrison, Mukhammad David Naimzada, Javad Nazari, Cuong Tat Nguyen, Huong Lan Thi Nguyen, Rajan Nikbakhsh, Virginia Nuñez-Samudio, Andrew T Olagunju, Nikita Otstavnov, Stanislav S Otstavnov, Mahesh P A, Adrian Pana, Eun-Kee Park, Faheem Hyder Pottoo, Akram Pourshams, Mohammad Rabiee, Navid Rabiee, Amir Radfar, Alireza Rafiei, Muhammad Aziz Rahman, Pradhum Ram, Priya Rathi, David Laith Rawaf, Salman Rawaf, Nima Rezaei, Nicholas L S Roberts, Thomas J Roberts, Luca Ronfani, Gholamreza Roshandel, Abdallah M Samy, Milena M Santric-Milicevic, Brijesh Sathian, Ione Jayce Ceola Schneider, Mario Sekerija, Sadaf G Sepanlou, Feng Sha, Masood Ali Shaikh, Rajesh Sharma, Aziz Sheikh, Sara Sheikhbahaei, Sudeep K Siddappa Malleshappa, Jasvinder A Singh, Freddy Sitas, Emma Elizabeth Spurlock, Paschalis Steiropoulos, Rafael Tabarés-Seisdedos, Eyayou Girma Tadesse, Ken Takahashi, Eugenio Traini, Bach Xuan Tran, Khanh Bao Tran, Ravensara S Travillian, Marco Vacante, Paul J Villeneuve, Francesco S Violante, Zabihollah Yousefi, Deniz Yuce, Vesna Zadnik, Maryam Zamanian, Kazem Zendehdel, Jianrong Zhang, Zhi-Jiang Zhang, Farshad Farzadfar, Christopher J L Murray, Mohsen Naghavi

## Abstract

**Background:**

Prevention, control, and treatment of respiratory tract cancers are important steps towards achieving target 3.4 of the UN Sustainable Development Goals (SDGs)—a one-third reduction in premature mortality due to non-communicable diseases by 2030. We aimed to provide global, regional, and national estimates of the burden of tracheal, bronchus, and lung cancer and larynx cancer and their attributable risks from 1990 to 2019.

**Methods:**

Based on the Global Burden of Diseases, Injuries, and Risk Factors Study (GBD) 2019 methodology, we evaluated the incidence, mortality, years lived with disability, years of life lost, and disability-adjusted life-years (DALYs) of respiratory tract cancers (ie, tracheal, bronchus, and lung cancer and larynx cancer). Deaths from tracheal, bronchus, and lung cancer and larynx cancer attributable to each risk factor were estimated on the basis of risk exposure, relative risks, and the theoretical minimum risk exposure level input from 204 countries and territories, stratified by sex and Socio-demographic Index (SDI). Trends were estimated from 1990 to 2019, with an emphasis on the 2010–19 period.

**Findings:**

Globally, there were 2·26 million (95% uncertainty interval 2·07 to 2·45) new cases of tracheal, bronchus, and lung cancer, and 2·04 million (1·88 to 2·19) deaths and 45·9 million (42·3 to 49·3) DALYs due to tracheal, bronchus, and lung cancer in 2019. There were 209 000 (194 000 to 225 000) new cases of larynx cancer, and 123 000 (115 000 to 133 000) deaths and 3·26 million (3·03 to 3·51) DALYs due to larynx cancer globally in 2019. From 2010 to 2019, the number of new tracheal, bronchus, and lung cancer cases increased by 23·3% (12·9 to 33·6) globally and the number of larynx cancer cases increased by 24·7% (16·0 to 34·1) globally. Global age-standardised incidence rates of tracheal, bronchus, and lung cancer decreased by 7·4% (−16·8 to 1·6) and age-standardised incidence rates of larynx cancer decreased by 3·0% (−10·5 to 5·0) in males over the past decade; however, during the same period, age-standardised incidence rates in females increased by 0·9% (−8·2 to 10·2) for tracheal, bronchus, and lung cancer and decreased by 0·5% (−8·4 to 8·1) for larynx cancer. Furthermore, although age-standardised incidence and death rates declined in both sexes combined from 2010 to 2019 at the global level for tracheal, bronchus, lung and larynx cancers, some locations had rising rates, particularly those on the lower end of the SDI range. Smoking contributed to an estimated 64·2% (61·9–66·4) of all deaths from tracheal, bronchus, and lung cancer and 63·4% (56·3–69·3) of all deaths from larynx cancer in 2019. For males and for both sexes combined, smoking was the leading specific risk factor for age-standardised deaths from tracheal, bronchus, and lung cancer per 100 000 in all SDI quintiles and GBD regions in 2019. However, among females, household air pollution from solid fuels was the leading specific risk factor in the low SDI quintile and in three GBD regions (central, eastern, and western sub-Saharan Africa) in 2019.

**Interpretation:**

The numbers of incident cases and deaths from tracheal, bronchus, and lung cancer and larynx cancer increased globally during the past decade. Even more concerning, age-standardised incidence and death rates due to tracheal, bronchus, lung cancer and larynx cancer increased in some populations—namely, in the lower SDI quintiles and among females. Preventive measures such as smoking control interventions, air quality management programmes focused on major air pollution sources, and widespread access to clean energy should be prioritised in these settings.

**Funding:**

Bill & Melinda Gates Foundation.

## Introduction

Tracheal, bronchus, and lung cancer is the leading cause of cancer deaths worldwide and the second leading cause of new cancer cases.^1^ Larynx cancer is a less common but lethal cancer of the respiratory tract that shares some similar risk factors with tracheal, bronchus, and lung cancer.^2^ Members of the UN committed to a one-third reduction in premature mortality due to non-communicable diseases by 2030 as target 3.4 of the UN Sustainable Development Goals (SDGs).^3^, ^4^ To realise this goal, the 17th World Health Assembly did a comprehensive review and assessment of the progress achieved in the prevention and control of cancers worldwide.^5^ Prevention, control, and treatment of respiratory tract cancers, which include both tracheal, bronchus, and lung cancer and larynx cancer, are important steps towards achieving this SDG target.

**Research in context**

**Evidence before this study**

Available datasets, registries, and the scientific literature were searched for information about tracheal, bronchus, lung, and larynx cancer, without any language restrictions. A 2019 report by the Global Burden of Disease Cancer Collaboration based on estimates from the Global Burden of Diseases, Injuries, and Risk Factors Study (GBD) 2017 showed that, despite decreasing age-standardised death rates, tracheal, bronchus, and lung cancer was still the leading cause of death due to cancer worldwide. The Global Cancer Incidence, Mortality and Prevalence (GLOBOCAN) project provides estimates about lung cancer mortality, but without identifying the attributable risk factors or showing the trend of estimates from past to present. The UN committed to a one-third reduction in premature mortality due to non-communicable diseases by 2030 as part of the UN Sustainable Development Goals (SDGs). Reducing mortality from tracheal, bronchus, and lung cancer would help countries to meet this SDG target by 2030. Considerable efforts have been made to decrease mortality rates of tracheal, bronchus, and lung cancer, including through expansion of smoking control programmes around the world, enactment and enforcement of air pollution regulations (although efforts have been limited in low-income and middle-income countries), implementation of low-dose CT screening strategies in certain countries for high-risk patients, and improvement of available therapies for patients with identifiable lesions.

**Added value of this study**

As part of GBD 2019, this study expands on the estimation of the incidence, mortality, and disability from respiratory tract cancers and their attributable risk factors in GBD 2019. This study provides estimates of the burden of respiratory tract cancers and attributable risk factors from 1990 to 2019, with an emphasis on trends from 2010 to 2019, in 204 countries and territories, and by socio-demographic status. It identifies the top risk factors associated with mortality from tracheal, bronchus, and lung cancer and larynx cancer in different populations worldwide and highlights disparate trends in the incidence of and mortality from tracheal, bronchus, and lung cancer and larynx cancer over the past decade. The findings from this study could encourage policy makers to identify populations with a disproportionately large burden of respiratory tract cancer and implement targeted strategies to reduce the risk and burden of this disease.

**Implications of all the available evidence**

Although the age-standardised death and incidence rates of tracheal, bronchus, and lung cancer and larynx cancer for both sexes combined decreased globally over the past decade, rates trended upwards for some populations, particularly females in certain countries lower on the Socio-demographic Index (SDI). This changing trend is important for researchers and policy makers to understand how comparative risk assessment, prevention, and cancer surveillance can be prioritised in low SDI countries. Smoking is still the single most important risk factor for mortality from larynx cancer and tracheal, bronchus, and lung cancer worldwide. However, the contribution of household air pollution from solid fuels to mortality from tracheal, bronchus, and lung cancer was even higher than that of smoking among females in many low SDI countries. Preventive measures, including smoking control programmes and clean energy for cooking and heating, should be prioritised in these settings to reduce the incidence of and mortality from larynx cancer and tracheal, bronchus, and lung cancer. The high SDI quintile had the highest age-standardised incidence rate of tracheal, bronchus, and lung cancer in 2019, but the fastest rate of decline over the past decade, so these countries should continue preventive measures and further optimise them to maintain their declines. In high SDI countries, screening in high-risk populations and early targeted treatment might help further decrease mortality from larynx cancer and tracheal, bronchus, and lung cancer. Our results on the relative contribution of various risk factors to tracheal, bronchus, and lung cancer mortality are not only helpful to guide risk reduction measures but also important to identify high-risk populations that might benefit from intensified strategies for prevention and treatment.

Smoking is a major risk factor for tracheal, bronchus, and lung cancer and larynx cancer, but smoking control programmes and other targeted health policies have curtailed smoking prevalence in recent years.^6^, ^7^, ^8^ Environmental exposures, including air pollution and occupational carcinogens, are other important risk factors for respiratory tract cancers that can also be reduced by appropriate regulations.^6^ Alcohol consumption is another risk factor for larynx cancer.^9^ So far, considerable efforts have been made to decrease the incidence of and mortality from larynx cancer and tracheal, bronchus, and lung cancer in high-income countries through smoking control programmes.^7^, ^10^ The relative contribution of each risk factor to mortality from respiratory tract cancers varies by sex and geographical area, with different behavioural, environmental, and occupational exposures, and different methods might be required to effectively address these risk factors.

To develop a clear understanding of the policy importance of the incidence and mortality of larynx cancer and tracheal, bronchus, and lung cancer, it is important to identify populations at high risk by geographical location. By analysing data from the Global Burden of Diseases, Injuries, and Risk Factors Study (GBD) 2019, we aimed to describe the burden of respiratory tract cancers (ie, tracheal, bronchus, and lung cancer and larynx cancer) and attributable risk factors, by sex and Socio-demographic Index (SDI), in 204 countries and territories from 1990 to 2019. We aimed to provide an overview of the current burden of tracheal, bronchus, and lung cancer and larynx cancer globally and regionally, as well as progress to reduce mortality and incidence over the past decade. We also aimed to shed light on risk factors for respiratory tract cancer so that policy makers can make informed decisions about the potential benefits of risk reduction policies, particularly in populations with high exposure to these risk factors.

## Methods

### Overview

Details of the methodology of GBD 2019, processes for estimating the burden of cancers, and risk factor quantification have been presented in previous publications,^6^, ^11^ as well as in appendix 1 (pp 10–24). Here, we briefly review the methods for estimating the burden of respiratory tract cancers—tracheal, bronchus, and lung cancer and larynx cancer—and their attributable risk factors.

### Estimation of mortality, incidence, and DALYs

In GBD 2019, the initial step in the process of estimating the burden of cancer was modelling cause-specific mortality. Mortality data from multiple sources, including vital registries and verbal autopsies, were extracted. Because of scarce mortality data for some locations and time points, mortality measures were also estimated from the cancer registry incidence data with separately modelled mortality-to-incidence ratios (MIRs). The codes corresponding to cancers in the GBD cause hierarchy were taken from the International Classification of Diseases (ICD)-9 and ICD-10 codebooks and mapped to the GBD cause list for each cancer (appendix 1 p 28). The mortality estimates were then used as inputs for a Cause of Death Ensemble model (CODEm), which predicts single-cause mortality based on the available data and covariates with a causal relationship.^11^, ^12^ Additionally, to ensure that all single-cause mortality estimates matched the separately modelled all-cause mortality estimates, CoDCorrect was used to scale single-cause mortality estimates to all-cause mortality estimates.^1^ The incidence of each cancer was calculated by dividing the cause-specific mortality estimates by the MIRs.

The survival of each cancer was modelled on the basis of MIR estimates for each location, year, sex, and age. The yearly prevalence of the population that did not survive beyond 10 years was divided into four sequelae corresponding to phases of the disease—diagnosis and primary therapy, the controlled phase, the metastatic phase, and the terminal phase—while the yearly prevalence of the population that survived beyond 10 years was only divided into the first and second phases. Disability weights associated with each of these four phases were multiplied by the sequelae prevalence to obtain the years lived with disability (YLDs). For larynx cancer, additional disability due to laryngectomy was also calculated using hospital data to estimate the proportion of the population with larynx cancer that underwent a laryngectomy. The hospital data sources and related ICD codes are described in appendix 1 (p 31). The years of life lost (YLLs) associated with each cancer were calculated by multiplying the number of deaths by age using a standard life expectancy at that age.^1^ Disability-adjusted life-years (DALYs) were calculated by summing the YLDs and YLLs.^1^

### Risk factor estimation

GBD 2019 followed comparative risk assessment (CRA) methodology to quantify attributable burden—the reduction in current disease burden that would have been possible if past population exposure had shifted to the theoretical minimum risk exposure level (TMREL).^6^, ^13^, ^14^ To model the attributable burden associated with a specific risk factor, four metrics were assessed: the metric of burden under study (deaths, YLDs, YLLs, or DALYs), the exposure level for the risk, the relative risk of outcomes caused by the exposure, and the counterfactual level of the risk factor exposure. For instance, in order to estimate the DALYs for tracheal, bronchus, and lung cancer attributable to a specific risk, these DALYs were multiplied by the population attributable fraction (PAF)—the proportion by which the DALYs would be decreased in a specific year if the exposure to a risk factor in the past was equal to the TMREL—for the tracheal, bronchus, and lung cancer risk–outcome pair for a given sex, age, year, and location.

GBD risk factors are classified into a risk hierarchy containing four levels, from broad risk categories (behavioural, environmental and occupational, and metabolic; Level 1) to the most specific (such as household air pollution from solid fuels; Level 4).^6^ Specific risk factors associated with each cause were selected in accordance with the World Cancer Research Fund grades of convincing or probable evidence, and include smoking; secondhand smoke; ambient particulate matter pollution; household air pollution from solid fuels; diets low in fruits; high fasting plasma glucose; residential radon exposure; and occupational exposure to asbestos, arsenic, beryllium, cadmium, chromium, diesel engine exhaust, nickel, polycyclic aromatic hydrocarbons, and silica for tracheal, bronchus, and lung cancer; and smoking, alcohol use, and occupational exposure to asbestos and sulphuric acid for larynx cancer. The definition and input data for each exposure are summarised in appendix 1 (pp 32–34). Details about the modelling strategy and TMREL for each exposure are summarised in appendix 1 of the report by the GBD 2019 Risk Factors Collaborators.^6^

To calculate age-standardised rates, we used the GBD global standard population.^15^ All rates are reported per 100 000 population in a given year, and point estimates are presented with 95% uncertainty intervals (UIs). 95% UIs were estimated by generating 1000 draws in each computational step, and taking the 25th and 975th ordered values of the draws. Moreover, the SDI, an index that incorporates total fertility rate in women younger than 25 years, lag-distributed income per capita, and average years of education, and ranges from 0 to 100,^15^ was used to categorise the 204 GBD countries and territories into quintiles. GBD locations are also aggregated into 21 regions and seven super-regions.

### Role of the funding source

The funder of the study had no role in study design, data collection, data analysis, data interpretation, or writing of the report.

## Results

### Burden of tracheal, bronchus, and lung cancer

In 2019, there were 2·26 million (95% UI 2·07–2·45) incident cases of tracheal, bronchus, and lung cancer globally; 1·52 million (1·37–1·68) cases were diagnosed in males and 737 000 (658 000–814 000) in females. From 2010 to 2019, the total number of new cases of tracheal, bronchus, and lung cancer increased by 23·3% (12·9–33·6) globally (table 1). In 2019, the age-standardised incidence rate of tracheal, bronchus, and lung cancer was 27·7 (25·3–30·0) cases per 100 000 population in both sexes combined, and 40·4 (36·5–44·4) cases per 100 000 in males and 16·8 (15·0–18·6) cases per 100 000 in females, globally.

**Table 1:** Trends in the number of incident cases and age-standardised incidence rates from 2010 to 2019 globally and by SDI quintiles, by sex and for both sexes combined, for tracheal, bronchus, and lung cancer and larynx cancer

		**Incidence in 2019**		**Percentage change in number of incident cases, 2010–19 (%)**	**Percentage change in age-standardised incidence rate, 2010–19 (%)**
		Number of cases (all ages)	Age-standardised rate (per 100 000)		
**Global**					
Larynx cancer					
	Both sexes	209 000 (194 000 to 225 000)	2·5 (2·3 to 2·7)	24·7% (16·0 to 34·1)	−2·5% (−9·3 to 4·8)
	Females	28 500 (26 100 to 31 300)	0·7 (0·6 to 0·7)	26·8% (16·8 to 37·9)	−0·5% (−8·4 to 8·1)
	Males	181 000 (166 000 to 196 000)	4·6 (4·2 to 5·0)	24·4% (14·6 to 34·6)	−3·0% (−10·5 to 5·0)
Tracheal, bronchus, and lung cancer					
	Both sexes	2 260 000 (2 070 000 to 2 450 000)	27·7 (25·3 to 30·0)	23·3% (12·9 to 33·6)	−4·6% (−12·5 to 3·3)
	Females	737 000 (658 000 to 814 000)	16·8 (15·0 to 18·6)	30·2% (18·5 to 42·2)	0·9% (−8·2 to 10·2)
	Males	1 520 000 (1 370 000 to 1 680 000)	40·4 (36·5 to 44·4)	20·3% (7·8 to 32·5)	−7·4% (−16·8 to 1·6)
**High SDI quintile**					
Larynx cancer					
	Both sexes	43 700 (39 300 to 48 500)	2·5 (2·2 to 2·8)	13·2% (2·2 to 25·5)	−5·5% (−15·0 to 5·0)
	Females	6230 (5390 to 7140)	0·7 (0·6 to 0·8)	12·1% (0·3 to 25·1)	−4·4% (−14·9 to 7·2)
	Males	37 500 (33 500 to 41 900)	4·5 (4·0 to 5·0)	13·3% (1·3 to 26·7)	−6·9% (−16·9 to 4·1)
Tracheal, bronchus, and lung cancer					
	Both sexes	709 000 (637 000 to 773 000)	37·4 (33·9 to 40·8)	11·9% (3·3 to 21·4)	−8·7% (−15·8 to −0·5)
	Females	278 000 (244 000 to 310 000)	27·3 (24·2 to 30·5)	16·0% (4·7 to 27·7)	−3·4% (−13·3 to 6·9)
	Males	431 000 (389 000 to 478 000)	49·7 (44·8 to 55·0)	9·4% (−0·5 to 20·9)	−12·8% (−20·7 to −3·6)
**High-middle SDI quintile**					
Larynx cancer					
	Both sexes	57 800 (52 500 to 63 500)	2·8 (2·5 to 3·1)	15·4% (5·0 to 26·8)	−7·8% (−16·1 to 1·2)
	Females	5990 (5350 to 6730)	0·5 (0·5 to 0·6)	19·6% (8·1 to 34·4)	−3·3% (−12·8 to 8·8)
	Males	51 900 (46 800 to 57 200)	5·4 (4·9 to 6·0)	14·9% (3·9 to 26·9)	−9·3% (−17·9 to 0·0)
Tracheal, bronchus, and lung cancer					
	Both sexes	671 000 (603 000 to 735 000)	32·6 (29·3 to 35·7)	19·9% (7·5 to 33·5)	−4·9% (−14·7 to 5·8)
	Females	193 000 (168 000 to 222 000)	17·1 (15·0 to 19·7)	30·7% (14·5 to 51·0)	4·8% (−8·2 to 21·2)
	Males	479 000 (422 000 to 539 000)	51·9 (45·7 to 58·4)	16·0% (1·7 to 31·8)	−9·3% (−20·4 to 2·7)
**Middle SDI quintile**					
Larynx cancer					
	Both sexes	51 400 (46 600 to 56 800)	2·0 (1·8 to 2·2)	39·2% (25·6 to 54·1)	3·7% (−6·0 to 14·6)
	Females	8260 (7280 to 9360)	0·6 (0·6 to 0·7)	35·5% (19·4 to 52·1)	0·5% (−11·3 to 12·4)
	Males	43 200 (38 700 to 48 300)	3·5 (3·2 to 3·9)	39·9% (24·8 to 58·1)	5·2% (−5·8 to 18·4)
Tracheal, bronchus, and lung cancer					
	Both sexes	580 000 (509 000 to 650 000)	23·7 (20·8 to 26·5)	36·4% (19·3 to 54·3)	1·2% (−11·1 to 14·1)
	Females	203 000 (172 000 to 236 000)	15·9 (13·5 to 18·5)	46·4% (25·5 to 69·7)	7·5% (−7·5 to 24·1)
	Males	377 000 (320 000 to 435 000)	32·5 (27·6 to 37·4)	31·6% (10·6 to 54·3)	−1·5% (−16·5 to 14·8)
**Low-middle SDI quintile**					
Larynx cancer					
	Both sexes	37 600 (33 500 to 42 100)	2·7 (2·4 to 3·0)	32·1% (17·7 to 49·1)	0·5% (−10·3 to 13·1)
	Females	5890 (5110 to 6810)	0·8 (0·7 to 0·9)	40·3% (23·0 to 63·4)	5·6% (−7·3 to 22·5)
	Males	31 700 (27 800 to 36 100)	4·7 (4·1 to 5·3)	30·7% (13·7 to 49·5)	0·8% (−11·8 to 15·2)
Tracheal, bronchus, and lung cancer					
	Both sexes	170 000 (153 000 to 186 000)	12·6 (11·3 to 13·8)	38·7% (24·0 to 52·0)	4·8% (−6·0 to 14·5)
	Females	51 800 (45 000 to 58 900)	7·4 (6·4 to 8·4)	55·8% (37·6 to 74·2)	15·8% (1·9 to 29·5)
	Males	118 000 (106 000 to 131 000)	18·3 (16·4 to 20·2)	32·4% (16·1 to 49·5)	1·6% (−10·9 to 14·5)
**Low SDI quintile**					
Larynx cancer					
	Both sexes	11 200 (9760 to 12 700)	2·1 (1·8 to 2·3)	27·2% (11·2 to 45·5)	−4·5% (−15·9 to 8·7)
	Females	2150 (1850 to 2450)	0·8 (0·7 to 0·9)	31·8% (15·4 to 52·9)	−1·1% (−12·9 to 13·7)
	Males	9020 (7670 to 10 500)	3·4 (2·9 to 4·0)	26·2% (7·4 to 49·0)	−4·8% (−18·1 to 11·4)
Tracheal, bronchus, and lung cancer					
	Both sexes	40 800 (35 100 to 48 600)	8·1 (7·0 to 9·5)	40·0% (25·4 to 55·8)	4·3% (−6·2 to 15·2)
	Females	11 000 (9620 to 12 400)	4·2 (3·7 to 4·8)	62·5% (42·6 to 83·0)	20·4% (6·1 to 35·9)
	Males	29 800 (24 800 to 37 200)	12·1 (10·1 to 14·9)	33·2% (17·8 to 52·0)	−0·2% (−11·4 to 13·0)

Data in parentheses are 95% uncertainty intervals. SDI=Socio-demographic Index.

**Figure 1:**
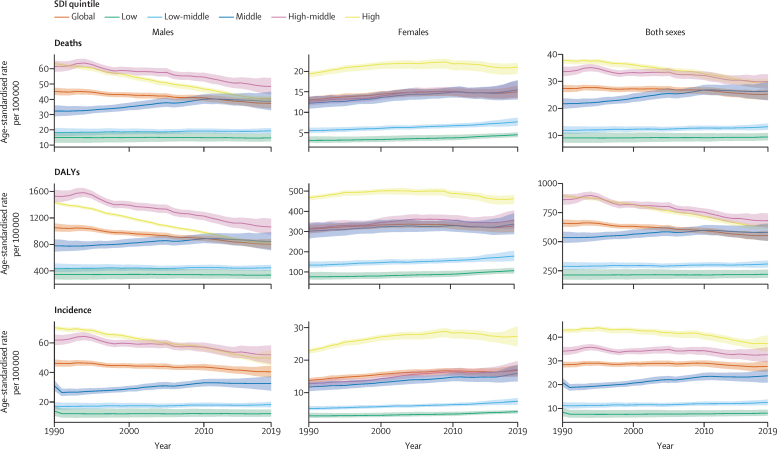
Trends in age-standardised rates of tracheal, bronchus, and lung cancer, 1990–2019 Deaths for males, females, and both sexes combined; disability-adjusted life-years (DALYs) for males, females, and both sexes combined; and incidence for males, females, and both sexes combined are shown. SDI=Socio-demographic Index.

Although the age-standardised incidence rate of tracheal, bronchus, and lung cancer was lower in females than in males in every year from 1990 to 2019, the female rate rose over the study period, while the male rate declined. The female age-standardised incidence rate went up by 22·3% (95% UI 10·2 to 40·0) from 1990 to 2019, although it only rose non-significantly by 0·9% (−8·2 to 10·2) from 2010 to 2019 (figure 1, table 1), while there was a decline in the male age-standardised incidence rate of 7·4% (−16·8 to 1·6) between 2010 and 2019, and by 12·5% (2·5 to 22·7) since 1990.

Throughout the study period, the age-standardised incidence of tracheal, bronchus, and lung cancer was highest in the high SDI quintile, at 37·4 (95% UI 33·9 to 40·8) new cases per 100 000 in 2019, higher than both the global age-standardised rate of 27·7 (25·3 to 30·0) cases per 100 000 and the age-standardised rate of 8·1 (7·0 to 9·5) per 100 000 in the low SDI quintile (table 1). There were 709 000 (637 000–773 000) incident cases of tracheal, bronchus, and lung cancer in the high SDI quintile in 2019. However, from 2010 to 2019, the high SDI quintile saw the largest decline in age-standardised incidence rate (−8·7% [–15·8 to −0·5]), while the low-middle and low SDI quintiles saw the largest increases (4·8% [–6·0 to 14·5] for the low-middle SDI quintile and 4·3% [–6·2 to 15·2] for the low SDI quintile).

Tracheal, bronchus, and lung cancer was the leading cause of cancer death globally in 2019, with nearly double the number of attributable deaths compared to the next-highest cancer.^1^ There were 2·04 million (95% UI 1·88 to 2·19) deaths attributable to tracheal, bronchus, and lung cancer in 2019: 1·39 million (1·26 to 1·51) deaths in males and 657 000 (590 000 to 719 000) deaths in females (table 2). The age-standardised death rate due to tracheal, bronchus, and lung cancer was 25·2 (23·2 to 27·0) per 100 000 in both sexes combined in 2019, 37·4 (34·1 to 40·7) per 100 000 in males and 15·0 (13·5 to 16·4) per 100 000 in females. The age-standardised death rate of tracheal, bronchus, and lung cancer declined by 5·7% (−12·6 to 1·5) globally from 2010 to 2019.

**Table 2:** DALYs and deaths in 2019 (counts and age-standardised rates) and trends from 2010 to 2019, globally and by SDI quintiles, by sex and for both sexes combined, for tracheal, bronchus, and lung cancer and larynx cancer

		**DALYs**			**Deaths**		
		Number of DALYs, 2019	Age-standardised rate per 100 000, 2019	Percentage change in age-standardised rate, 2010– 19 (%)	Number of deaths, 2019	Age-standardised rate per 100 000, 2019	Percentage change in age-standardised rate, 2010–19 (%)
**Global**							
Larynx cancer							
	Both sexes	3 260 000 (3 030 000 to 3 510 000)	38·8 (36·1 to 41·8)	−9·3% (−15·3 to −2·9)	123 000 (115 000 to 133 000)	1·5 (1·4 to 1·6)	−8·5% (−14·3 to −2·3)
	Females	464 000 (421 000 to 512 000)	10·7 (9·7 to 11·8)	−5·2% (−13·3 to 4·0)	17 800 (16 200 to 19 700)	0·4 (0·4 to 0·5)	−4·9% (−12·8 to 4·1)
	Males	2 800 000 (2 590 000 to 3 030 000)	69·3 (64·1 to 75·2)	−10·0% (−16·9 to −3·2)	106 000 (97 800 to 115 000)	2·7 (2·5 to 3·0)	−9·3% (−15·9 to −2·8)
Tracheal, bronchus, and lung cancer							
	Both sexes	45 900 000 (42 300 000 to 49 300 000)	551·6 (509·0 to 593·1)	−7·3% (−14·6 to 0·4)	2 040 000 (1 880 000 to 2 190 000)	25·2 (23·2 to 27·0)	−5·7% (−12·6 to 1·5)
	Females	14 300 000 (13 000 000 to 15 700 000)	327·6 (298·5 to 360·4)	−1·3% (−10·0 to 7·7)	657 000 (590 000 to 719 000)	15·0 (13·5 to 16·4)	0·1% (−8·2 to 8·6)
	Males	31 600 000 (28 600 000 to 34 700 000)	802·9 (727·6 to 879·8)	−10·0% (−18·6 to −0·4)	1 390 000 (12 600 00 to 1 510 000)	37·4 (34·1 to 40·7)	−8·7% (−16·8 to 0·2)
**High SDI quintile**							
Larynx cancer							
	Both sexes	342 000 (324 000 to 359 000)	20·1 (19·1 to 21·2)	−10·3% (−13·6 to −6·5)	14 600 (13 700 to 15 300)	0·8 (0·7 to 0·8)	−8·9% (−11·8 to −5·6)
	Females	52 100 (47 300 to 56 800)	6·0 (5·5 to 6·5)	−7·8% (−11·8 to −2·9)	2280 (2030 to 2490)	0·2 (0·2 to 0·2)	−7·5% (−11·1 to −2·9)
	Males	290 000 (274 000 to 305 000)	35·6 (33·7 to 37·5)	−11·8% (−15·4 to −7·7)	12 400 (11 700 to 12 900)	1·5 (1·4 to 1·5)	−10·7% (−14·1 to −7·2)
Tracheal, bronchus, and lung cancer							
	Both sexes	11 300 000 (10 800 000 to 11 700 000)	636·0 (606·9 to 656·8)	−11·0% (−13·3 to −8·8)	578 000 (534 000 to 603 000)	29·8 (27·8 to 31·0)	−8·9% (−11·0 to −6·9)
	Females	4 310 000 (4 010 000 to 4 510 000)	461·9 (436·4 to 481·2)	−5·5% (−8·7 to −2·3)	224 000 (201 000 to 237 000)	21·1 (19·4 to 22·1)	−3·7% (−6·7 to −0·7)
	Males	7 030 000 (6 730 000 to 7 260 000)	835·7 (801·8 to 863·2)	−14·8% (−16·8 to −12·6)	354 000 (333 000 to 367 000)	40·6 (38·3 to 42·1)	−13·1% (−15·0 to −11·1)
**High-middle SDI quintile**							
Larynx cancer							
	Both sexes	780 000 (723 000 to 836 000)	38·0 (35·2 to 40·7)	−18·3% (−24·2 to −12·5)	30 400 (28 100 to 32 500)	1·5 (1·4 to 1·6)	−16·9% (−22·7 to −11·4)
	Females	77 800 (70 800 to 85 900)	7·3 (6·6 to 8·0)	−12·9% (−20·6 to −4·0)	3250 (2940 to 3600)	0·3 (0·3 to 0·3)	−11·9% (−19·5 to −2·8)
	Males	703 000 (649 000 to 756 000)	72·9 (67·4 to 78·4)	−19·5% (−25·7 to −13·5)	27 200 (25 100 to 29 200)	2·9 (2·7 to 3·2)	−18·4% (−24·5 to −12·7)
Tracheal, bronchus, and lung cancer							
	Both sexes	14 000 000 (12 700 000 to 15 300 000)	680·7 (620·1 to 743·5)	−9·6% (−17·9 to −0·2)	614 000 (559 000 to 670 000)	29·9 (27·2 to 32·6)	−7·1% (−15·6 to 2·3)
	Females	3 870 000 (3 430 000 to 4 420 000)	355·1 (314·9 to 404·8)	−0·6% (−13·3 to 14·5)	175 000 (154 000 to 200 000)	15·4 (13·6 to 17·6)	2·1% (−10·5 to 17·6)
	Males	10 100 000 (8 960 000 to 11 300 000)	1064·2 (943·6 to 1187·2)	−13·4% (−23·4 to −1·9)	439 000 (390 000 to 491 000)	48·3 (43·1 to 53·9)	−11·4% (−21·2 to 0·1)
**Middle SDI quintile**							
Larynx cancer							
	Both sexes	912 000 (829 000 to 1 000 000)	34·7 (31·5 to 38·1)	−8·5% (−16·5 to −0·3)	34 900 (31 600 to 38 200)	1·4 (1·3 to 1·6)	−7·8% (−15·7 to 0·2)
	Females	135 000 (121 000 to 151 000)	10·1 (9·1 to 11·3)	−10·9% (−20·4 to −0·6)	5380 (4810 to 6070)	0·4 (0·4 to 0·5)	−10·2% (−19·8 to 0·1)
	Males	777 000 (699 000 to 860 000)	61·1 (55·2 to 67·5)	−7·2% (−16·7 to 2·3)	29 500 (26 500 to 32 600)	2·5 (2·3 to 2·8)	−6·5% (−15·6 to 2·8)
Tracheal, bronchus, and lung cancer							
	Both sexes	14 900 000 (13 000 000 to 16 900 000)	579·4 (506·1 to 655·0)	−3·1% (−15·5 to 10·3)	630 000 (551 000 to 712 000)	26·3 (23·0 to 29·7)	−1·7% (−13·8 to 11·6)
	Females	4 470 000 (3 810 000 to 5 200 000)	336·7 (287·3 to 390·8)	2·3% (−13·4 to 19·2)	194 000 (165 000 to 224 000)	15·5 (13·2 to 17·8)	3·7% (−11·8 to 19·5)
	Males	10 400 000 (8 760 000 to 12 200 000)	844·4 (711·1 to 986·0)	−4·7% (−19·6 to 12·2)	436 000 (368 000 to 509 000)	38·7 (32·8 to 45·0)	−3·4% (−18·0 to 13·2)
**Low-middle SDI quintile**							
Larynx cancer							
	Both sexes	900 000 (801 000 to 1 020 000)	61·3 (54·6 to 69·4)	−5·2% (−16·0 to 7·3)	32 100 (28 700 to 36 200)	2·3 (2·1 to 2·6)	−4·5% (−14·8 to 7·0)
	Females	141 000 (120 000 to 165 000)	18·6 (15·8 to 21·7)	0·4% (−13·0 to 16·8)	4970 (4260 to 5780)	0·7 (0·6 to 0·8)	0·2% (−12·5 to 15·6)
	Males	759 000 (662 000 to 868 000)	106·8 (93·4 to 122·0)	−5·1% (−17·5 to 8·8)	27 100 (23 800 to 31 000)	4·1 (3·7 to 4·7)	−4·0% (−15·8 to 9·0)
Tracheal, bronchus, and lung cancer							
	Both sexes	4 400 000 (3 980 000 to 4 830 000)	308·2 (278·6 to 337·5)	2·9% (−7·5 to 12·6)	174 000 (158 000 to 190 000)	13·2 (12·0 to 14·3)	3·7% (−6·4 to 12·7)
	Females	1 320 000 (1 140 000 to 1 510 000)	178·5 (154·4 to 204·3)	14·0% (−0·0 to 28·8)	52 500 (45 800 to 59 600)	7·7 (6·7 to 8·7)	14·3% (0·3 to 28·3)
	Males	3 080 000 (2 780 000 to 3 400 000)	447·9 (405·3 to 492·4)	−0·4% (−12·0 to 11·4)	122 000 (110 000 to 134 000)	19·3 (17·5 to 21·2)	0·6% (−10·7 to 12·0)
**Low SDI quintile**							
Larynx cancer							
	Both sexes	326 000 (285 000 to 376 000)	56·3 (49·2 to 64·6)	−8·7% (−19·7 to 4·3)	11 200 (9790 to 12 800)	2·2 (1·9 to 2·5)	−7·4% (−17·7 to 5·0)
	Females	57 900 (49 600 to 66 900)	19·1 (16·5 to 22·1)	−5·7% (−17·4 to 8·9)	1910 (1640 to 2210)	0·7 (0·6 to 0·8)	−3·6% (−14·7 to 10·3)
	Males	268 000 (228 000 to 319 000)	94·5 (80·7 to 111·4)	−8·8% (−21·7 to 6·6)	9320 (7970 to 11 000)	3·7 (3·2 to 4·3)	−7·6% (−20·0 to 6·8)
Tracheal, bronchus, and lung cancer							
	Both sexes	1 220 000 (1 040 000 to 1 450 000)	219·9 (188·7 to 259·9)	2·7% (−7·8 to 13·9)	46 000 (39 600 to 53 900)	9·4 (8·1 to 10·9)	3·0% (−6·9 to 13·4)
	Females	306 000 (264 000 to 348 000)	106·4 (92·5 to 120·6)	19·6% (4·7 to 35·9)	11 300 (9780 to 12 700)	4·5 (3·9 to 5·1)	20·2% (4·9 to 36·5)
	Males	910 000 (754 000 to 1 130 000)	337·3 (282·2 to 412·8)	−1·4% (−13·2 to 12·1)	34 700 (29 000 to 42 200)	14·7 (12·4 to 17·5)	−1·2% (−12·3 to 11·3)

Data in parentheses are 95% uncertainty intervals. DALY=disability-adjusted life-year. SDI=Socio-demographic Index.

Globally in 2019, there were 45·9 million (95% UI 42·3 to 49·3) DALYs due to tracheal, bronchus, and lung cancer, of which 98·8% came from YLLs and 1·2% from YLDs. In 2019, the age-standardised DALY rate for tracheal, bronchus, and lung cancer was almost 2·5 times higher in males than in females and 3·1 times higher in the high-middle SDI quintile (where rates were highest) than in the low SDI quintile (table 2). The global age-standardised DALY rate declined by 7·3% (−14·6 to 0·4) for both sexes combined from 2010 to 2019.

While age-standardised rates of incidence, death, and DALYs for tracheal, bronchus, and lung cancer declined globally over the past decade, non-significant increases in all three measures were observed in central, eastern, and western sub-Saharan Africa; south and southeast Asia; and Oceania, and in the low and low-middle SDI quintiles (appendix 2 pp 3–40). In these locations, age-standardised rates increased even more substantially among females than among males. More detailed estimates of incidence, mortality, DALYs, YLDs, and YLLs for tracheal, bronchus, and lung cancer at regional and national levels are summarised in appendix 2 (pp 3–40, 78–136).

### Burden of larynx cancer

In 2019, there were 209 000 (95% UI 194 000 to 225 000) incident cases of larynx cancer for both sexes combined worldwide, 181 000 (166 000 to 196 000) cases in males and 28 500 (26 100 to 31 300) in females (table 1). From 2010 to 2019, the number of incident cases of larynx cancer increased by 24·7% (16·0 to 34·1; table 1). The global age-standardised incidence rate declined by 2·5% (−9·3 to 4·8) over the same time period.

In 2019, the number of deaths attributable to larynx cancer was 123 000 (95% UI 115 000 to 133 000) for both sexes combined; 106 000 (97 800 to 115 000) deaths occurred in males and 17 800 (16 200 to 19 700) deaths occurred in females. From 2010 to 2019, the age-standardised death rate of larynx cancer decreased (−9·3% [–15·9 to −2·8] in males and −4·9% [–12·8 to 4·1] in females) at the global level. Globally, there were 3·26 million (3·03 to 3·51) DALYs due to larynx cancer in 2019, of which 97% came from YLLs and 3% from YLDs. Age-standardised DALY rates declined by 9·3% (2·9 to 15·3) from 2010 to 2019 globally for both sexes combined. The age-standardised DALY rate attributable to larynx cancer was approximately six times higher in males than in females (table 2). Age-standardised DALY rates for larynx cancer in 2019 were highest in the low-middle SDI quintile (61·3 (54·6 to 69·4] per 100 000) and low SDI quintile (56·3 [49·2 to 64·6] per 100 000) and lowest in the high SDI quintile (20·1 [19·1 to 21·2] per 100 000).

Similarly to tracheal, bronchus, and lung cancer, while age-standardised incidence, death, and DALY rates for larynx cancer declined globally over the past decade, there was a non-significant rise in rates for all three measures in the Caribbean, and non-significant increases in age-standardised incidence rates were observed in east Asia, southeast Asia, north Africa and the Middle East, and the middle and low-middle SDI quintiles (appendix 2 pp 41–77). More regional and national estimates for incidence, deaths, DALYs, YLLs, and YLDs attributable to larynx cancers are summarised in appendix 2 (pp 41–136).

### Mortality from tracheal, bronchus, and lung cancer and larynx cancer attributable to leading risk factors

**Figure 2:**
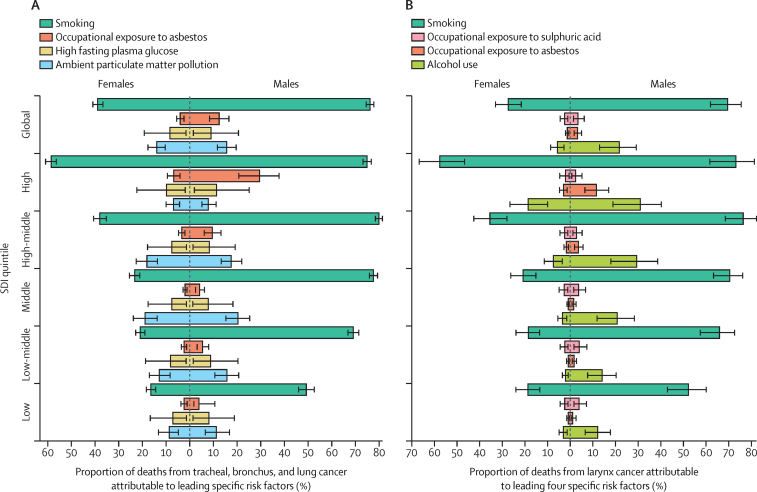
Proportion of deaths attributable to leading specific risk factors, by sex and SDI quintile, 2019, for tracheal, bronchus, and lung cancer (A) and larynx cancer (B) Leading four specific risks for attributable deaths are shown for females and males. SDI=Socio-demographic Index. The error bars indicate 95% uncertainty intervals.

In 2019, an estimated 80·3% (95% UI 77·5–83·2) of all deaths from tracheal, bronchus, and lung cancer were attributable to risk factor exposure. Smoking was the leading risk factor for deaths from tracheal, bronchus, and lung cancer that year among the most specific GBD risks, contributing to 64·2% (61·9–66·4) of total deaths from tracheal, bronchus, and lung cancer for both sexes combined, or 16·1 (14·7–17·5) age-standardised deaths per 100 000. The proportion of deaths attributable to smoking varied substantially by sex, with 76·2% (74·6–77·8) of deaths from tracheal, bronchus, and lung cancer attributable to smoking among males compared to 38·9% (36·7–40·9) among females (figure 2A).

**Figure 3:**
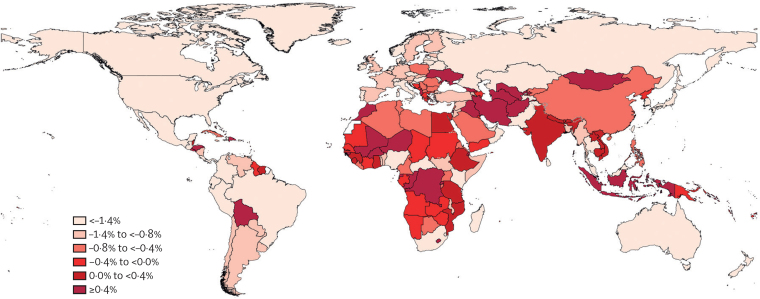
Annualised rate of change in the age-standardised death rate of tracheal, bronchus, and lung cancer attributable to smoking, 2010–19

In 2019, age-standardised death rates for tracheal, bronchus, and lung cancer attributable to smoking were generally highest in countries in central Europe, such as Montenegro and Hungary, and western Europe, such as Monaco and Greece (appendix 2 pp 137–203). Likewise, among SDI quintiles, rates were highest in the high SDI quintiles. Rates were highest in higher SDI countries in 2019 as a function of historically high rates. However, from 2010 to 2019, countries in north Africa and the Middle East (eg, Iraq and Palestine), western and eastern sub-Saharan Africa (eg, Cabo Verde and Rwanda), and the Caribbean (eg, Saint Kitts and Nevis) had some of the highest annualised rates of increase in age-standardised death rates of tracheal, bronchus, and lung cancer attributable to smoking, while many countries with the highest smoking-attributable rates (eg, Greenland, Hungary, Poland, and Serbia) saw steady declines over the same period (figure 3). Among SDI quintiles, the high SDI quintile had the largest annualised rate of decline in age-standardised death rates of tracheal, bronchus, and lung cancer attributable to smoking from 2010 to 2019 (1·5% [95% UI 0·6 to 2·5] decrease), while the middle SDI quintile was the only quintile that did not have a negative annualised rate of change (0·0% [–1·9 to 2·0]).

Overall, between 2010 and 2019, the greatest decline in age-standardised death rates attributable to smoking was seen in the high SDI quintile, with a decrease of 12·9% (95% UI 11·0–14·9) in both sexes combined. Smoking-attributable age-standardised deaths from tracheal, bronchus, and lung cancer decreased among males in all GBD regions, except for central sub-Saharan Africa, between 2010 and 2019. However, during the same period, an increase in smoking-attributable age-standardised deaths from tracheal, bronchus, and lung cancer was observed among females in 11 of 21 GBD regions, most prominently in eastern Europe, central sub-Saharan Africa, south Asia, north Africa and the Middle East, and eastern sub-Saharan Africa.

The second-highest specific risk factor for tracheal, bronchus, and lung cancer was ambient particulate matter pollution, contributing to 15·1% (95% UI 11·3 to 18·9) of deaths from tracheal, bronchus, and lung cancer for both sexes combined, or an age-standardised attributable death rate of 3·8 (2·8 to 4·9) per 100 000. The attributable proportion of deaths was similar for males and females, at 15·6% (11·7 to 19·6) for males and 13·9% (10·3 to 17·6) for females (figure 2A). Globally, from 2010 to 2019, the age-standardised death rate attributable to ambient particulate matter pollution remained almost unchanged (−0·6% [–10·4 to 11·4]). Trends in age-standardised death rates attributable to ambient particulate matter pollution varied starkly by SDI quintile, with the low SDI quintile showing a 43·3% (17·4 to 86·2) increase, the low-middle SDI quintile showing a 36·0% (15·0 to 65·8) increase, and the middle SDI quintile showing a non-significant 6·5% (−9·4 to 26·2) increase, from 2010 to 2019, compared to a 21·3% (15·8 to 27·6) decrease in the high SDI quintile and a non-significant 6·2% (−17·3 to 7·2) decrease in the high-middle SDI quintile. The rise in the age-standardised death rate attributable to ambient particulate matter pollution in the low SDI quintile was more prominent in females, with an 80·4% (44·3 to 137·5) increase. From 1990 to 2019, the low, low-middle, and middle SDI quintiles all had increases in age-standardised death rates attributable to ambient particulate matter pollution higher than 130%, compared to a decrease of nearly 50% in the high SDI quintile. Several high SDI countries saw age-standardised tracheal, bronchus, and lung cancer death rates attributable to ambient particulate matter pollution decline by more than 65% over the same 30-year period, such as Finland (−75·0% [–94·8 to 30·2]), Estonia (−70·5% [–89·3 to −29·5]), Switzerland (−70·1% [–83·4 to −35·2]), Sweden (−68·2% [–92·2 to 30·4]), and the USA (−68·0% [–85·3 to −22·8]).

Occupational exposure to asbestos was the third-highest specific risk factor for deaths from tracheal, bronchus, and lung cancer in 2019 for both sexes combined (9·7% [95% UI 6·9–12·5] of all deaths; 2·5 [1·8–3·3] age-standardised deaths per 100 000). For males, 12·4% (8·3–16·6) of deaths from tracheal, bronchus, and lung cancer were attributable to asbestos, compared to 4·0% (2·4–5·5) for females (figure 2A). The age-standardised death rate for tracheal, bronchus, and lung cancer attributable to asbestos was highest in the high SDI quintile, at 5·7 (4·1–7·3) deaths per 100 000, and lowest in the low SDI quintile, at 0·4 (0·2–1·0) deaths per 100 000 (appendix 2 pp 137–203).

**Figure 4:**
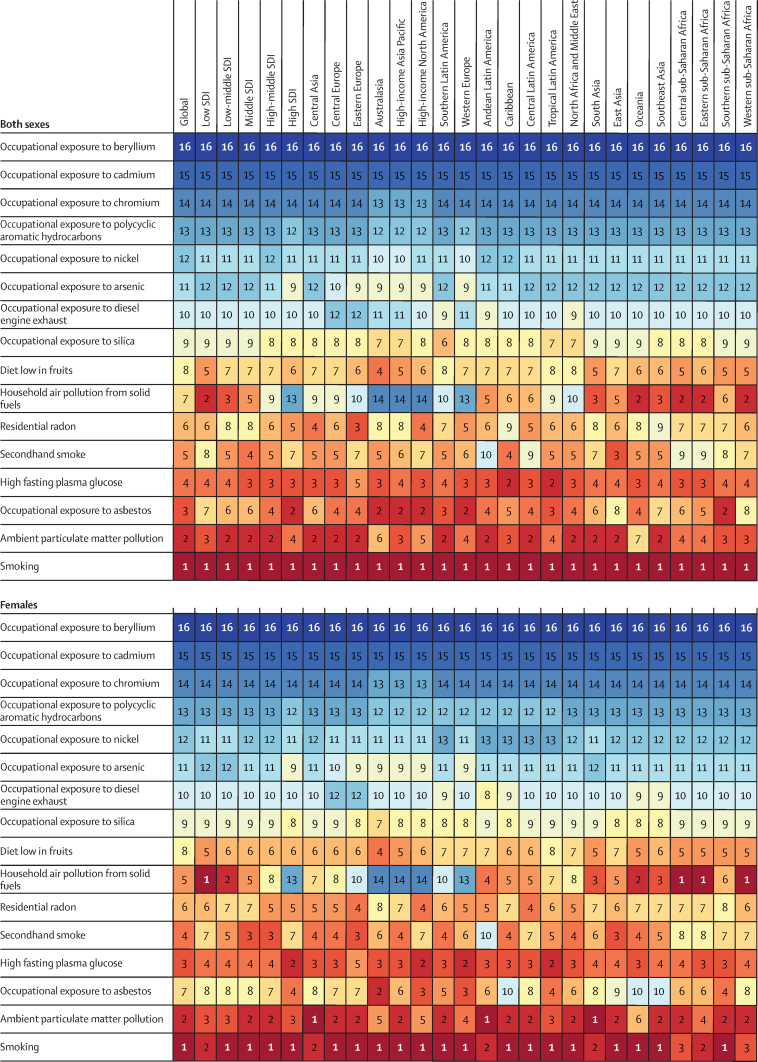

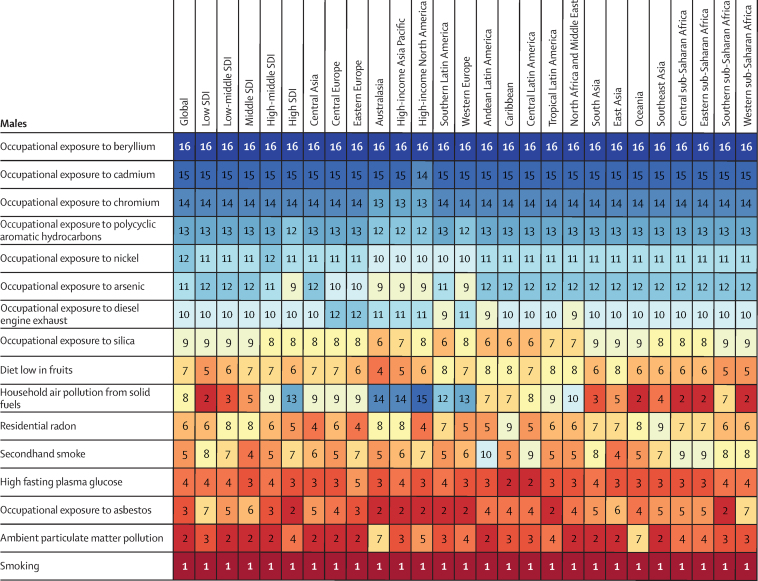
Ranked contribution of risk factors to the age-standardised death rate of tracheal, bronchus, and lung cancer by region, 2019, for both sexes combined, females, and males Risk factors are ranked from 1 (leading risk factor for age-standardised death; dark red) to 16 (lowest risk factor for age-standardised death; dark blue).

There were some regional disparities in the risk-attributable burden of tracheal, bronchus, and lung cancer between sexes. In every region, smoking was the leading specific risk factor for age-standardised deaths from tracheal, bronchus, and lung cancer per 100 000 for males and for both sexes combined. However, in females, household air pollution from solid fuels was the leading specific risk factor in the low SDI quintile and in central, eastern, and western sub-Saharan Africa in 2019, while ambient particulate matter pollution was the leading specific risk factor in central and south Asia and Andean Latin America in 2019 (figure 4). Between 2010 and 2019, female age-standardised death rates attributable to household air pollution from solid fuels remained almost constant in the low SDI quintile (2·1% [95% UI −13·7 to 19·0]), but decreased significantly in the high SDI quintile (−40·3% [–54·4 to −26·9]). Secondhand smoke was the fourth-leading specific risk factor for deaths from tracheal, bronchus, and lung cancer in females (and the fifth-leading risk factor in males). Age-standardised death rates for tracheal, bronchus, and lung cancer attributable to secondhand smoke decreased in high, high-middle, and middle SDI quantiles among males between 2010 and 2019. However, a decline was only seen in the high SDI quintile among females during the same period. Figure 4 provides more information about regional age-standardised death rates for tracheal, bronchus, and lung cancer by attributable risks in 2019. appendix 2 (pp 137–203) summarises the deaths from tracheal, bronchus, and lung cancer attributable to GBD risk factors at the regional and national levels.

Similarly to tracheal, bronchus, and lung cancer, the leading specific risk factor for larynx cancer in 2019 was smoking, contributing to 63·4% (95% UI 56·3 to 69·3) of all deaths from larynx cancer, or 0·9 (0·8 to 1·1) age-standardised deaths per 100 000 (appendix 2 pp 204–237). 69·5% (61·9 to 75·5) of deaths from larynx cancer in males were attributable to smoking, compared to 27·4% (21·6 to 33·0) in females (figure 2B; results for country-level annualised rates of change in smoking-attributable deaths from larynx cancer are summarised in appendix 2 p 238). Between 2010 and 2019, in both sexes combined, age-standardised death rates attributable to smoking declined in almost all regions globally and in almost all SDI quintiles (−12·1% [–15·2 to −9·0] in the high SDI quintile and −10·9% [–23·6 to 4·4] in the low SDI quintile). The second-highest risk factor was alcohol use, contributing to 19·4% (11·6 to 26·3) of all deaths from larynx cancer in both sexes combined, 21·7% (12·9 to 29·2) among males and 5·7% (2·8 to 8·6) among females. Occupational exposure to sulphuric acid was the third-highest attributable risk factor, contributing to 3·3% (1·4 to 6·0) of deaths in both sexes combined: 3·4% (1·4 to 6·2) in males and 2·5% (1·0 to 4·5) in females. Deaths from larynx cancer attributable to GBD risk factors at the regional and national levels are summarised in appendix 2 (pp 204–237).

## Discussion

### Overview

Globally, the total number of incident cases of, and deaths and DALYs due to, tracheal, bronchus, and lung cancer and larynx cancer for both sexes combined increased over the past decade, while age-standardised rates steadily decreased. Despite considerable progress on decreasing these rates at the global level, concerning trends were observed in certain populations and geographical regions. While age-standardised incidence and death rates for tracheal, bronchus, and lung cancer decreased globally in males from 2010 to 2019, they increased among females globally over the same period, albeit more slowly than over the previous two decades. Moreover, increases in tracheal, bronchus, and lung cancer incidence and death rates were observed in the low SDI and low-middle SDI quintiles for both sexes combined, with even larger increases among females in these SDI quintiles.

Although smoking was the leading specific risk factor for mortality from respiratory tract cancer worldwide in 2019, household air pollution due to solid fuels and ambient particulate matter pollution were also important risk factors for tracheal, bronchus, and lung cancer in females in the low SDI quintile and several GBD regions. Furthermore, the age-standardised death rates of tracheal, bronchus, and lung cancer attributable to ambient particulate matter pollution rose significantly in these populations over the past decade, while the age-standardised death rates attributable to household air pollution remained stagnant. These trends underline the need for expanded smoking control programmes and regulations worldwide, as well as stricter air quality regulations and an expansion of access to clean sources of cooking and heating energy in lower SDI countries, to reduce the risk of tracheal, bronchus, and lung cancer. More geographically specific air pollution exposure assessments could be considered to identify populations at high risk of developing tracheal, bronchus, and lung cancer. In the USA and some high SDI countries in Europe, screening protocols for lung cancer have recently been explored or implemented for certain high-risk subpopulations;^16^, ^17^, ^18^, ^19^, ^20^, ^21^ however, less information is available about their potential use or challenges associated with their implementation in lower SDI settings.

### Reducing burden of tracheal, bronchus, and lung cancer and larynx cancer through risk factor mitigation

Smoking was the leading specific risk factor for deaths from larynx cancer and tracheal, bronchus, and lung cancer worldwide in 2019. However, over the past decade, the age-standardised death rates of tracheal, bronchus, and lung cancer attributable to smoking in the high SDI quintile decreased by 12·9% (95% UI 11·1–14·9) and those for larynx cancer decreased by 12·1% (9·0–15·2), compared to overall decreases in age-standardised death rates of 8·9% (5·6–11·8) for tracheal, bronchus, and lung cancer and 8·9% (6·9–11·0) for larynx cancer. These findings suggest that smoking control programmes in high SDI regions have been successful in reducing mortality from respiratory tract cancer. Policies involving health-related warnings on cigarette packages; bans on advertising, promotion, and sponsorship; and taxation on tobacco products have been shown to reduce smoking prevalence worldwide.^22^, ^23^ The MPOWER policy package by the Tobacco Free Initiative has succeeded in expanding evidence-based tobacco control measures to almost two-thirds of the global population as of July, 2019.^24^ The MPOWER tobacco control package includes monitoring tobacco use and preventive measures, warning about tobacco use, enforcing bans on advertising tobacco, and raising taxes on tobacco.^24^

Despite substantial progress in reducing the smoking-attributable respiratory tract cancer burden at the global level, and strong evidence showing that smoking control measures are effective, the age-standardised death rate for tracheal, bronchus, and lung cancer attributable to smoking increased in 55 countries and territories from 2010 to 2019, particularly in north Africa and the Middle East, western and eastern sub-Saharan Africa, and the Caribbean. These contradictory findings suggest that there is a huge opportunity to implement culturally appropriate policies and regulations that target smoking control measures to help reduce the burden of respiratory tract cancer in these countries. A variety of tobacco use methods, including waterpipes (hookah) are commonly used in the Middle East and have a long history in the region, highlighting the importance of community partnership and cultural sensitivity in optimising smoking control measures in these settings.^25^ More research might be necessary to assess the barriers to effective smoking control programmes in locations where death rates for tracheal, bronchus, and lung cancer due to smoking are on the rise.

These trends are particularly concerning among females, with 11 of 21 GBD regions, particularly eastern Europe, central sub-Saharan Africa, and south Asia, showing increases in age-standardised mortality rates of tracheal, bronchus, and lung cancer attributable to smoking in females over the past decade compared to just one region for males—central sub-Saharan Africa. This finding might be partially explained by the four-stage model of the cigarette epidemic.^26^, ^27^ Based on this model, an increase in the prevalence of female smoking tends to occur decades later than male smoking, so there is a considerable lag time before the cumulative effects of smoking on health outcomes are observed in females.^27^ Secondhand smoke also contributed to an increasing attributable age-standardised death rate of tracheal, bronchus, and lung in females in countries lower on the SDI range. Genetic and hormonal factors are also thought to potentially play a role in residual deaths from lung cancer in females, but research on this subject is still very scarce.^28^

Ambient particulate matter pollution was the second-highest specific risk factor for deaths from tracheal, bronchus, and lung cancer in 2019, accounting for 15·1% (95% UI 11·3–18·9) of tracheal, bronchus, and lung cancer mortality worldwide. This finding is in line with previous research, which found that air pollution was a major risk factor for lung cancer incidence, particularly in non-smokers.^29^, ^30^, ^31^, ^32^ Researchers found that each 10 μg/m^3^ increase in criteria air pollutants (ie, carbon monoxide, ground-level ozone, lead, nitrogen dioxide, particulate matter, and sulphur dioxide) increased lung cancer mortality by up to 27% in the USA (for PM_2·5_ concentrations over a 26-year period)^30^ and by up to 30% in the Netherlands (for PM_10_ concentrations over a 7-year period).^33^ As of 2016, approximately 92% of the world's population was living in areas not meeting WHO air quality criteria,^34^ again demonstrating the considerable potential to reduce the global burden of tracheal, bronchus, and lung cancer through risk factor mitigation, in this case through improved air quality.

One successful example of a policy to control ambient particulate matter pollution is the US Clean Air Act, which, in 1970, gave the federal Environmental Protection Agency the power to regulate the emission of criteria air pollutants by setting air quality requirements and expanding federal enforcement. As of 2017, it had succeeded in reducing particulate matter concentration by more than 80% since its establishment.^35^ As a likely consequence of this risk reduction, the age-standardised death rate of tracheal, bronchus, and lung cancer attributable to ambient particulate matter pollution declined by 68·0% (95% UI 22·8–85·3) in the USA from 1990 to 2019, one of the largest reductions in the world.

Our findings demonstrate that, despite considerable progress in reducing the age-standardised death rate of tracheal, bronchus, and lung cancer attributable to ambient particulate matter pollution in high SDI countries over the past decade, age-standardised rates remained unchanged globally. This was due to a rise of more than 35% in the age-standardised death rate of tracheal, bronchus, and lung cancer attributable to ambient particulate matter pollution in low SDI and low-middle SDI quintiles between 2010 and 2019. In these settings, reducing exposure to ambient particulate matter pollutants will be crucial to reducing the risk of tracheal, bronchus, and lung cancer in the future.

Furthermore, in the low SDI quintile, household air pollution due to solid fuels was an even larger contributor to deaths from tracheal, bronchus, and lung cancer than ambient particulate matter pollution (ranked second to smoking). In 2019, household air pollution due to solid fuels was the first-ranked specific risk factor for tracheal, bronchus, and lung cancer in females in the low SDI quintile as a whole and in central, eastern, and western sub-Saharan Africa. Solid fuel exposure has previously been identified as an important risk factor for lung cancer, particularly in never-smokers.^36^, ^37^ In many low SDI countries, the adoption and sustainable use of clean fuels (ie, gas and electricity) have been limited due to high costs.^38^ Solid fuels with incomplete combustion produce more air pollutants and can cause both indoor and outdoor air pollution when used for cooking purposes.^39^ The Clean Cooking Alliance is one example of an initiative to expand access to clean, affordable, and modern energy for cooking.^38^ Expanding access to cleaner energy and other efforts to reduce air pollution require investments in the energy sector and are of particular importance in regions such as sub-Saharan Africa, where about half of the population does not have access to electricity.^38^

Occupational exposure to asbestos was the third-leading specific risk factor for deaths from tracheal, bronchus, and lung cancer worldwide in 2019, contributing to 9·7% (95% UI 6·9–12·5) of all deaths. Occupational exposures can be reduced substantially by enacting policies such as banning the use of asbestos, as has been done in many countries, such as Australia, Taiwan, the USA, and European countries such as Denmark, France, Germany, and more.^40^, ^41^, ^42^ A study of a cohort of textile workers showed that up to 24% of deaths were preventable by following US Occupational Safety and Health Administration standards.^43^ In the high SDI quintile, the age-standardised death rate of tracheal, bronchus, and lung cancer attributable to asbestos was nearly 15 times higher than in the low SDI quintile, reflective of industrialisation and cumulative occupational exposures that occurred decades ago. It is essential that countries in low SDI regions also ban the use of products that contain asbestos and other occupational carcinogens as these countries continue to industrialise, to minimise the burden of tracheal, bronchus, and lung cancer due to these risk factors in the future.^44^

### Reducing burden of tracheal, bronchus, and lung cancer through health-system strengthening and advances in treatment options

We found that more than 98% of the burden of tracheal, bronchus, and lung cancer and larynx cancer in 2019 came from YLLs, with less than 2% from YLDs, indicating that these cancers cause far more premature death than disability. Delayed diagnosis, limited curative therapeutic options, and inadequate health systems are important contributors to the high burden of and mortality from tracheal, bronchus, and lung cancer and larynx cancer. The 17th World Health Assembly emphasised enhancing access to health care for patients diagnosed with cancer by strengthening national health systems and international cooperation.^5^ Over the past decade, many higher SDI countries succeeded in reducing the age-standardised death rate of tracheal, bronchus, and lung cancer by more than 10%, primarily through effective preventive measures (risk exposure reduction) and, to a lesser extent, advances in treatment options. In high SDI European countries, China, and the USA, advances in treatment, including molecular agents and immunotherapies recently approved by the US Food and Drug Administration, as well as potential new cytotoxic agents, emerging molecularly targeted agents, and novel immunotherapeutic strategies for treating tracheal, bronchus, and lung cancer have opened up an avenue to improve survival outcomes of patients with larynx cancer and tracheal, bronchus, and lung cancer in the near future.^45^ Such advances in the treatment of tracheal, bronchus, and lung cancer and larynx cancer could reduce the disease burden of these cancers in upcoming years as these treatments become more widely available, at least in locations that have the financial capacity to consider their implementation. To reduce the burden of tracheal, bronchus, and lung cancer through better treatment options beyond high SDI countries, however, effective therapies will need to be made accessible at much lower costs. Many lower SDI countries will not have the resources to support new, elaborate, and intensive cancer treatments, as many do not have the health-system capacity to provide widespread access to current treatment options such as surgery, chemotherapy, genetic analysis, and targeted medications. Although risk reduction remains the best option for reducing the burden of tracheal, bronchus, and lung cancer and larynx cancer in most populations, lower SDI countries should also be considering opportunities for more broad health-system strengthening, which could further help to reduce the burden of these and other non-communicable diseases.

### Reducing tracheal, bronchus, and lung cancer burden through screening and subsequent early treatment

One of the reasons for the low survival of patients with tracheal, bronchus, and lung cancer and larynx cancer is that patients are often diagnosed in advanced stages. This is due to several reasons, including rapid progression of the disease, challenges with referral pathways and health-system capacity to diagnose cancer, and the scarcity of effective population screening modalities.^16^, ^46^, ^47^ There is currently no screening protocol for larynx cancer, but lung cancer screening (specifically, low-dose CT) was recently shown to be a cost-effective approach for high-risk populations in the USA^48^ and is under assessment in Europe.^17^, ^18^ Lung cancer screening could improve the survival of patients with lung cancer by identifying patients at early stages.^16^ The National Lung Screening Trial (NLST) in the USA found that the implementation of annual low-dose CT resulted in a 20% reduction in lung cancer mortality in people at high risk of developing lung cancer.^16^, ^48^, ^49^ Based on the available evidence, the US Preventive Services Task Force now recommends annual lung cancer screening with low-dose CT for people in the USA aged 55–80 years who have at least a 30-pack-year history of smoking, currently smoke, or have quit smoking within the past 15 years.^19^ In addition to low-dose CT, a signature of non-coding mRNAs, DNA methylation, and somatic mutations that can be detected from peripheral blood are under investigation to detect tracheal, bronchus, and lung cancers at early stages.^20^, ^50^

A screening strategy might serve as a cost-effective action to reduce the burden of tracheal, bronchus, and lung cancer in some high SDI regions, where the incidence of tracheal, bronchus, and lung cancer is highest and where the resources needed to implement such a programme are available.^20^ This is particularly true for populations at the highest risk of death from tracheal, bronchus, and lung cancer, since lung cancer screening is most cost-effective when the risk of premature death is highest.^20^ Despite progress on tracheal, bronchus, and lung cancer screening research, however, screening should only be seen as an effective method for reducing the burden of tracheal, bronchus, and lung cancer in certain populations and locations with the capacity to diagnose and manage imaging findings, and only in combination with programmes aimed at reducing exposure to risk factors. Many countries are not in a position to adopt lung cancer screening protocols due to a combination of factors, including expenses and inadequate health-system capacity required for screening and subsequent treatment, and the need to consider additional unique challenges associated with implementation in low-resource settings. Moreover, with 80·3% (95% UI 77·5–83·2) of all deaths from tracheal, bronchus, and lung cancer in 2019 attributable to risk factor exposure, preventive measures are still the most important steps to control incidence, and thereby mortality, worldwide. If the necessary resources become more widely available in the future, the cost-effectiveness of screening protocols for tracheal, bronchus, and lung cancer could be assessed and then considered for wider implementation in high-risk populations around the world. For policy makers evaluating whether and how to expand screening in the future, it is important first to consider local risk-factor contexts, challenges with misdiagnosis, and, as previously discussed, the ability of the health-care system to treat patients who are diagnosed. Screening programmes alone will not improve health outcomes without subsequent access to effective diagnosis and treatment options. Any strategy to reduce the burden of tracheal, bronchus, and lung cancer should take all of these local factors into consideration and prioritise risk reduction and health-system strengthening before implementing screening protocols, particularly in lower SDI settings.

Finally, the age-standardised death rate due to tracheal, bronchus, and lung cancer increased by 3·0% (95% UI −6·9 to 13·4) in the low SDI quintile between 2010 and 2019 for both sexes combined; this finding warrants the urgent attention of health-care policy makers in these countries. Even more concerning, the age-standardised death rate from tracheal, bronchus, and lung cancer in this quintile increased by 20·2% (4·9 to 36·5) among females over the same period. If current trends continue, many low SDI countries may struggle to meet the SDG target to reduce non-communicable disease mortality by at least a third by 2030.^4^, ^5^ In low SDI countries, prioritisation of resources to reduce the risk of larynx cancer and tracheal, bronchus, and lung cancer by implementing smoking control programmes, stronger air quality regulations and enforcement, better access to clean fuel sources, and occupational risk factor legislation, can be cost-effective approaches to reduce deaths from larynx cancer and tracheal, bronchus, and lung cancer.^51^ Furthermore, previous research suggests that joint exposure to ambient particulate matter pollution and smoking might have greater than additive effects on death rates for tracheal, bronchus, and lung cancer,^30^ so such interventions might produce larger benefits than suggested in our analysis.

### Limitations and strengths

Although GBD 2019 provides a comprehensive review and assessment of global incidence, death, and DALYs of major cancers, it faces several limitations. Some data sources were not as reliable as others, particularly in lower SDI countries. It is possible that the number of tracheal, bronchus, and lung cancer cases was underestimated in some low SDI countries because of limited access to diagnostic tools. Another limitation of this study is that we were not able to capture the impact of genetic factors in the incidence of and therapeutic response in patients with tracheal, bronchus, and lung cancer. Although estimation of the proportion of cases of tracheal, bronchus, and lung cancer attributable to these non-modifiable risk factors was beyond the scope of the present study, these risk factors could have an impact on the epidemiological picture of tracheal, bronchus, and lung cancer in different regions. For instance, a mutation in the epidermal growth factor receptor (EGFR) affects the therapeutic response to, and outcome of, tracheal, bronchus, and lung cancer, and presence of this mutation varies from 9·1% in the Dutch population to 51·4% in east Asia.^52^, ^53^ As another example, the *2 allele of mitochondrial aldehyde dehydrogenase (ALDH2*2), which is associated with decreased metabolism of alcohol, higher alcohol toxicity, and possible carcinogenicity, is more prevalent in east Asian populations than in other populations.^54^ Additionally, the magnitude of the contribution of each risk factor to lung cancer varies for different histological subtypes of lung cancer,^55^ and we could not differentiate these different histological subtypes. Similarly, we were unable to separate statistical records related to tracheal cancers from lung and bronchial cancers because of the registration overlap in data sources for this study.

GBD 2019 global estimates of tracheal, bronchus, and lung cancer incidence and mortality are slightly higher than the Global Cancer Incidence, Mortality and Prevalence (GLOBOCAN) 2018^56^ estimates (2·19 million *vs* 2·09 million incident cases in 2018, and 1·98 million *vs* 1·76 million deaths in 2018), which can be partially attributed to GBD 2019 covering 19 more countries than GLOBOCAN and to each set of estimates having different data sources and methods. GBD 2019 includes many aggregated cancer registry databases, including Cancer Incidence in Five Continents (CI5), EUREG, US SEER Database, and NORDCAN.^56^, ^57^, ^58^

### Conclusion

Although tracheal, bronchus, and lung cancer was still the leading cause of cancer death globally in 2019, age-standardised incidence and death rates for respiratory tract cancer (ie, tracheal, bronchus, and lung cancer and larynx cancer) appeared to decline between 2010 and 2019 at the global level. However, our study highlights the increasing rates of tracheal, bronchus, and lung cancer incidence in the low SDI quintile, particularly among females. Smoking remained the leading risk factor for deaths from tracheal, bronchus, and lung cancer at the global level for both males and females in 2019, but for females in six GBD regions and in the low SDI quintile the leading risk factor was either ambient particulate matter pollution or household air pollution from solid fuels. These findings should provide the impetus for policy makers to expand culturally responsive smoking control programmes, enact and enforce stricter air quality regulations, and expand access to clean energy in homes. Policies such as smoking cessation programmes and smoking regulations, air quality acts with enforceable regulations, low-emission zones in towns and cities, widespread access to clean energy in locations without it, and occupational risk standards that minimise asbestos exposure all have the potential to help reduce the incidence of and mortality attributable to tracheal, bronchus, and lung cancer, if done using locally appropriate, evidence-based strategies. Screening protocols such as low-dose CT for tracheal, bronchus, and lung cancer and the introduction of effective treatments such as mutation-targeted cancer treatment could be additional steps to reduce mortality from and the burden of larynx cancer and tracheal, bronchus, and lung cancer in high SDI countries with adequate health-care infrastructure. In lower SDI settings, improving access to health care, streamlining referral pathways for cancer, and strengthening health systems for cancer diagnosis and treatment could be important strategies to reduce tracheal, bronchus, and lung cancer mortality, in addition to risk reduction programmes.

## Declaration of interests

R Ancuceanu reports consultancy or speakers' fees from UCB, Sandoz, AbbVie, Zentiva, Teva, Laropharm, CEGEDIM, Angelini, Biessen Pharma, Hofigal, AstraZeneca, and Stada. J A Singh reports fees from Crealta and Horizon, Medisys, Fidia, Two labs Inc, Adept Field Solutions, Clinical Care options, ClearView Healthcare Partners, Putnam Associates, FocusForward, Navigant Consulting, Spherix, MedIQ, UBM LLC, Trio Health, Medscape, WebMD, and Practice Point communications; and the National Institutes of Health and the American College of Rheumatology; placement on the speaker's bureau of Simply Speaking; ownership of stock options in TPT Global Tech, Vaxart pharmaceuticals and Charlotte's Web Holdings. J A Singh previously owned stock options in Amarin, Viking, and Moderna pharmaceuticals; placement on the steering committee of OMERACT, an international organisation that develops measures for clinical trials and receives arm's length funding from 12 pharmaceutical companies; and serves on the FDA Arthritis Advisory Committee. J A Singh is also a member of the Veterans Affairs Rheumatology Field Advisory Committee; and is the editor and the Director of the UAB Cochrane Musculoskeletal Group Satellite Center on Network Meta-analysis. All other authors declare no competing interests.
