# Supplementary material for: Global, regional, and national burden of respiratory tract cancers and associated risk factors from 1990 to 2019: a systematic analysis for the Global Burden of Disease Study 2019
Source: Lancet Respir Med. 2021 Sep;9(9):1030–49. doi: 10.1016/S2213-2600(21)00164-8 (PMC8410610; doi:10.1016/S2213-2600(21)00164-8)
Supplement: Supplementary appendix 1 [file mmc1.pdf]

THE LANCET
Respiratory Medicine

Supplementary appendix 1

This appendix formed part of the original submission and has been peer reviewed. We post it as supplied by the authors.

Supplement to: GBD 2019 Respiratory Tract Cancers Collaborators. Global, regional, and national burden of respiratory tract cancers and associated risk factors from 1990 to 2019: a systematic analysis for the Global Burden of Disease Study 2019. *Lancet Respir Med* 2021; published online August 16. http://dx.doi.org/10.1016/S2213-2600(21)00164-8.

Supplementary appendix 1

Methods appendix to "Global, regional, and national burden of respiratory tract cancers and associated risk factors from 1990 to 2019: a systematic analysis for the Global Burden of Disease Study 2019"

This appendix provides further methodological detail for "Global, regional, and national burden of respiratory tract cancers and associated risk factors from 1990 to 2019: a systematic analysis for the Global Burden of Disease Study 2019".

Portions of this appendix have been reproduced or adapted from Vos et al.[1] and Fitzmaurice et al.[2] References are provided for reproduced sections.

Preamble

This appendix provides further methodological detail for " Global, regional, and national burden of respiratory tract cancers and associated risk factors from 1990 to 2019: a systematic analysis for the Global Burden of Disease Study 2019." This study complies with the Guidelines for Accurate and Transparent Health Estimates Reporting (GATHER) recommendations (table S1).

A detailed description of the data sources and processing steps for the GBD 2019 cancer estimation can be found in appendix 1 of Vos et al.”[1]

Authors' Contributions

Managing the estimation or publications process

Hedyeh Ebrahimi, Zahra Aryan, Lisa Force, Kelly Compton, Shahabeddin Rezaei, and Christopher J L Murray.

Writing the first draft of the manuscript

Hedyeh Ebrahimi, Zahra Aryan, Catherine Bisignano, Sahar Saeedi Moghaddam, Shahabeddin Rezaei, Farhad Pishgar, Lisa Force, Farshad Farzadfar, and Mohsen Naghavi.

Primary responsibility for applying analytical methods to produce estimates

Lisa Force, Weijia Fu, Jonathan Kocarnik, and Frances Dean.

Providing data or critical feedback on data sources

Hassan Abolhassani, Shailesh Advani, Sohail Ahmad, Fares Alahdab, Syed Aljunid, Saeed Amini, Catalina Liliana Andrei, Jalal Arabloo, Morteza Arab-Zozani, Marcel Ausloos, Dejana Braithwaite, Michael Brauer, Carlos Castañeda-Orjuela, Dinh-Toi Chu, Michael Chung, Aaron Cohen, Xiaochen Dai, Lalit Dandona, Rakhi Dandona, Meseret Derbew Molla, Tim Driscoll, Weijia Fu, Birhan Gebregiorgis, Ahmad Ghashghaee, Mahaveer Golechha, Bárbara Goulart, Nima Hafezi-Nejad, Claudiu Herteliu, Chi Hoang, Nobuyuki Horita, Mowafa Househ, Seyed Sina Irvani, Rovshan Khalilov, Jonathan Kocarnik, Burcu Kucuk Bicer, G Anil Kumar, Savita Lasrado, Stephen Lim, Reza Malekzadeh, Navid Manafi, Ritesh Menezes, Tomasz Miazgowski, Abdollah Mohammadian-Hafshejani, Shafiu Mohammed, Ali H Mokdad, Lorenzo Monasta, Joana Morgado-da-Costa, Javad Nazari, Cuong Nguyen, Huong Nguyen, Rajan Nikbakhsh,

Andrew Olagunju, Mahesh P A, Adrian Pana, Mohammad Rabiee, Navid Rabiee, Alireza Rafiei, Pradhum Ram, David Laith Rawaf, Salman Rawaf, Nima Rezaei, Nicholas Roberts, Luca Ronfani, Abdallah Samy, Milena Santric-Milicevic, Brijesh Sathian, Mario Sekerija, Sadaf Sepanlou, Feng Sha, Masood Shaikh, Sara Sheikhbahaei, Jasvinder Singh, Rafael Tabarés-Seisdedos, Bach Tran, Deniz Yuce, Vesna Zadnik, Kazem Zendehdel, Zhi-Jiang Zhang, Farshad Farzadfar, Christopher J L Murray, and Mohsen Naghavi.

Developing methods or computational machinery

Hedyeh Ebrahimi, Zahra Aryan, Farhad Pishgar, Michael Brauer, Aaron Cohen, Xiaochen Dai, Frances Dean, Tim Driscoll, Weijia Fu, Mowafa Househ, Rovshan Khalilov, Jonathan Kocarnik, Alan D Lopez, Ali H Mokdad, Shahabeddin Rezaei, Nicholas Roberts, Abdallah Samy, Farshad Farzadfar, Christopher J L Murray, and Mohsen Naghavi.

Providing critical feedback on methods or results

Hedyeh Ebrahimi, Zahra Aryan, Sahar Saeedi Moghaddam, Farhad Pishgar, Lisa Force, Hassan Abolhassani, Eman Abu-Gharbieh, Shailesh Advani, Sohail Ahmad, Fares Alahdab, Vahid Alipour, Syed Aljunid, Saeed Amini, Robert Ancuceanu, Catalina Liliana Andrei, Tudorel Andrei, Jalal Arabloo, Morteza Arab-Zozani, Marcel Ausloos, Atalel Awedew, Atif Baig, Ali Bijani, Antonio Biondi, Dejana Braithwaite, Maria Teresa Bustamante-Teixeira, Zahid Butt, Giulia Carreras, Carlos Castañeda-Orjuela, Odgerel Chimed-Ochir, Dinh-Toi Chu, Michael Chung, Aaron Cohen, Baye Dagnew, Xiaochen Dai, Lalit Dandona, Rakhi Dandona, Frances Dean, Meseret Derbew Molla, Abebaw Desta, Tim Driscoll, Emerito Jose Faraon, Pawan Faris,

Irina Filip, Florian Fischer, Weijia Fu, Birhan Gebregiorgis, Ahmad Ghashghaee, Mahaveer Golechha, Dr Kebebe Gonfa, Bárbara Goulart, Maximiliano Guerra, Nima Hafezi-Nejad, Samer Hamidi, Claudiu Herteliu, Chi Hoang, Nobuyuki Horita, Mihaela Hostiuc, Mowafa Househ, Irena Ilic, Milena Ilic, Seyed Sina Irvani, Farhad Islami, Ashwin Kamath, Supreet Kaur, Rovshan Khalilov, Ejaz Khan, Jonathan Kocarnik, G Anil Kumar, Carlo La Vecchia, Savita Lasrado, Paolo Lauriola, Elvynna Leong, Bingyu Li, Azeem Majeed, Reza Malekzadeh, Navid Manafi, Ritesh Menezes, Tomasz Miazgowski, Sanjeev Misra, Abdollah Mohammadian-Hafshejani, Shafiu Mohammed, Ali H Mokdad, Alex Molassiotis, Rahmatollah Moradzadeh, Lidia Morawska, Joana Morgado-da-Costa, Mukhammad David Naimzada, Javad Nazari, Cuong Nguyen, Huong Nguyen, Rajan Nikbakhsh, Andrew Olagunju, Nikita Otstavnov, Stanislav Otstavnov, Mahesh P A, Adrian Pana, Eun-Kee Park, Faheem Pottoo, Akram Pourshams, Mohammad Rabiee, Navid Rabiee, Amir Radfar, Alireza Rafiei, Muhammad Aziz Rahman, Pradhum Ram, Priya Rathi, David Laith Rawaf, Salman Rawaf, Nima Rezaei, Shahabeddin Rezaei, Nicholas Roberts, Thomas Roberts, Gholamreza Roshandel, Abdallah Samy, Milena Santric-Milicevic, Brijesh Sathian, Ione Schneider, Sadaf Sepanlou, Feng Sha, Masood Shaikh, Rajesh Sharma, Sara Sheikhbahaei, Sudeep Siddappa Malleshappa, Jasvinder Singh, Rafael Tabarés-Seisdedos, Eyayou Tadesse, Eugenio Traini, Bach Tran, Khanh Tran, Ravensara Travillian, Marco Vacante, Paul Villeneuve, Francesco Violante, Zabihollah Yousefi, Deniz Yuce, Vesna Zadnik, Maryam Zamanian, Kazem Zendehdel, Jianrong Zhang, Christopher J L Murray, and Mohsen Naghavi.

Drafting the work or revising it critically for important intellectual content

Hedyeh Ebrahimi, Zahra Aryan, Catherine Bisignano, Sahar Saeedi Moghaddam, Farhad Pishgar, Lisa Force, Hassan Abolhassani, Eman Abu-Gharbieh, Shailesh Advani, Sohail Ahmad, Fares Alahdab, Saeed Amini, Robert Ancuceanu, Catalina Liliana Andrei, Jalal Arabloo, Morteza Arab-Zozani, Malke Asaad, Marcel Ausloos, Atif Baig, Antonio Biondi, Tone Bjørge, Dejana Braithwaite, Hermann Brenner, MARIA Teresa Bustamante-Teixeira, Michael Chung, Aaron Cohen, Baye Dagnew, Meseret Derbew Molla, Abebaw Desta, Emerito Jose Faraon, Irina Filip, Florian Fischer, Silvano Gallus, Birhan Gebregiorgis, Ahmad Ghashghaee, Giuseppe Gorini, Bárbara Goulart, Maximiliano Guerra, Nima Hafezi-Nejad, Simon Hay, Claudiu Herteliu, Chi Hoang, Nobuyuki Horita, Mowafa Househ, Ivo Iavicoli, Irena Ilic, Milena Ilic, Seyed Sina Irvani, Farhad Islami, Rovshan Khalilov, Ejaz Khan, Jonathan Kocarnik, Carlo La Vecchia, Qing Lan, Iván Landires, Savita Lasrado, Paolo Lauriola, Elvynna Leong, Reza Malekzadeh, Navid Manafi, Ritesh Menezes, Tomasz Miazgowski, Abdollah Mohammadian-Hafshejani, Shafiu Mohammed, Ali H Mokdad, Alex Molassiotis, Lorenzo Monasta, Joana Morgado-da-Costa, Shane Morrison, Mukhammad David Naimzada, Javad Nazari, Cuong Nguyen, Huong Nguyen, Rajan Nikbakhsh, Virginia Nuñez-Samudio, Andrew Olagunju, Nikita Otstavnov, Stanislav Otstavnov, Mahesh P A, Adrian Pana, Amir Radfar, Pradhum Ram, Priya Rathi, David Laith Rawaf, Salman Rawaf, Nima Rezaei, Shahabeddin Rezaei, Thomas Roberts, Luca Ronfani, Gholamreza Roshandel, Abdallah Samy, Milena Santric-Milicevic, Ione Schneider, Mario Sekerija, Sadaf Sepanlou, Masood Shaikh, Aziz Sheikh, Sara Sheikhbahaei, Sudeep Siddappa Malleshappa, Jasvinder Singh, Freddy Sitas, Paschalis Steiropoulos, Eyayou Tadesse, Ken Takahashi, Bach Tran, Ravensara Travillian, Marco Vacante, Deniz Yuce, Vesna

Zadnik, Maryam Zamanian, Kazem Zendehdel, Jianrong Zhang, Zhi-Jiang Zhang, Farshad Farzadfar, Christopher J L Murray, and Mohsen Naghavi.

Extracting, cleaning, or cataloging data; designing or coding figures and tables

Hedyeh Ebrahimi, Zahra Aryan, Sahar Saeedi Moghaddam, Farhad Pishgar, Saeed Amini, Rovshan Khalilov, Jonathan Kocarnik, Ali H Mokdad, Shahabeddin Rezaei, Abdallah Samy, Emma Spurlock, and Mohsen Naghavi.

Managing the overall research enterprise

Michael Brauer, Lalit Dandona, Alan D Lopez, Ali H Mokdad, Christopher J L Murray, and Mohsen Naghavi.

Table of Contents

Section 1: Definition of indicator 10

Section 2: Data sources 11

Section 2.1: Cancer incidence data sources 11

Section 2.2: Cancer mortality data sources 11

Section 2.3: Mortality/incidence ratio data sources 11

Section 2.4: Bias of categories of input data 11

Section 3: Data analysis 13

Section 3.1: Cancer registry data formatting 13

Section 3.2: Mortality-to-incidence ratio estimation 15

Section 3.3: CODEm models 17

Section 3.4: CoDCorrect 18

Section 3.5: Incidence estimation 18

Section 3.6: Prevalence and YLD estimation 18

Section 4: GBD global population age standard 23

Section 5: Risk-specific estimation 24

Funding Sources 25

References 37

List of appendix tables and figures

Appendix tables

Table S1. GATHER guideline Checklist 26
Table S2. List of International Classification of Diseases codes mapped to Global Burden of Disease cause list for tracheal, bronchus, and lung (TBL), and larynx cancer incidence and mortality[1] 28
Table S3. Duration of four prevalence phases by cancer 29
Table S4. Disabilities weights 30
Table S5. Sources of hospital data[27] 31
Table S6. Definition and input sources for risk factors used in Global Burden of Disease estimations for tracheal, bronchus, and lung (TBL), and larynx cancers[23] 32

Appendix figures

Figure S1. Flowchart of GBD 2019 cancer mortality and YLL estimation 35
Figure S2. Flowchart of GBD 2019 cancer incidence, prevalence, and YLD estimation 36

Section 1: Definition of indicator

In this study, we provided estimates for tracheal, bronchus, and lung (TBL) and larynx cancers, in addition to their attributable risk factors, for both sexes, from 1990 to 2019 for 204 countries and territories. Additionally, all ICD-9 and ICD-10 codes corresponding to TBL and larynx cancer (see table S2) were included in these estimates. Countries and territories included in the analysis, as well as their respective SDI values, can be found in table S3.

Section 2: Data sources

Section 2.1: Cancer incidence data sources[2]

In GBD 2019, cancer incidence data were gathered from individual population-based cancer registries or aggregated databases of cancer registry data, including "Cancer Incidence in Five Continents (CI5)",[2-11] EUREG,[12] and NORDCAN.[13] Data were excluded if they were not representative of the coverage population (eg, hospital-based registries), if they did not cover all malignant neoplasms as defined in ICD-9 (140–208) or ICD-10 (C00–C96), if they did not include data for both sexes and all age groups (except for paediatric cancer registries), if the data were limited to years before 1980, or if the source did not provide details on the population covered. Preference was given to registries with national coverage over those with only local coverage, except those from countries where GBD provides subnational estimates. Data input sources are available in the online GBD citation tool, http://ghdx.healthdata.org/gbd-2019.

Section 2.2: Cancer mortality data sources

A detailed description of the data sources and processing steps for the cause of death (CoD) database can be found in appendix 1 of Vos et al.[1]

Section 2.3: Mortality and incidence ratio data sources[2]

While most cancer registries only report cancer incidence, mortality data were also extracted from the source if a cancer registry also reported cancer mortality. These data were used as inputs to the mortality-to-incidence model.

Section 2.4: Bias of categories of input data[1]

Cancer registry data can be biased in multiple ways. A high proportion of ill-defined cancer cases in the registry data requires redistribution of these cases to other cancers. Changes between coding systems can lead to artificial differences in disease estimates; however, in GBD 2019, this

bias was adjusted by mapping the different coding systems to the GBD causes. Underreporting of cancers that require advanced diagnostic techniques (eg, leukaemia, brain, pancreatic, and liver cancer) can be an issue in cancer registries from low-income countries. However, misclassification of metastatic sites as primary cancer can lead to the overestimation of cancer sites that are common sites for metastases. Since many cancer registries are located in urban areas, the representativeness of the registry for the general population can also be problematic. The accuracy of mortality data reported in cancer registries usually depends on the quality of the vital registration system. If the vital registration system is incomplete or of poor quality, the mortality-to-incidence ratio (MIR) can be biased to lower ratios.

Section 3: Data analysis

Flowcharts describing the conceptual overview of the data processing of the GBD 2019 cancer estimation are available in figure S1 and figure S2.

Section 3.1: Cancer registry data formatting[1]

Cancer registry data went through multiple processing steps before integration with the COD database. First, the original data were transformed into standardised files, which included standardisation of format, categorisation, and registry names (#1 in figure S1). Second, some cancer registries report individual codes as well as aggregated totals (eg, C18, C19, and C20 are reported individually, but the aggregated group of C18–C20 [colorectal cancer] is also reported in the registry data). The data processing step “subtotal recalculation” (#2 in figure S1) verifies these totals and subtracts the values of any individual codes from the aggregates.

In the third step (#3 in figure S1), cancer registry incidence data and cancer registry mortality data are mapped to GBD causes. A different map is used for incidence and for mortality data because of the assumption that there are no deaths for certain cancers. The assumption is that deaths assigned to benign neoplasms are miscoded and should correctly be assigned to the invasive cancer. Examples are benign or in situ neoplasms. Benign or in situ neoplasms found in the cancer registry incidence dataset were simply dropped from that dataset. The same neoplasms reported in a cancer registry mortality dataset were mapped to the respective invasive cancer (eg, melanoma in situ in the cancer registry incidence dataset was dropped from the dataset; melanoma in situ in the cancer registry mortality dataset was mapped to melanoma).

In the fourth data processing step (#4 in figure S1), cancer registry data were standardised to GBD age groups. Age-specific incidence rates were generated using all datasets that include microdata, and datasets that report age groups up to 95+ years of age. Age-specific mortality

rates were generated from the CoD data using methods described in Vos et al.[1] Age-specific proportions were then generated by applying the age-specific rates to a given registry population that required age-splitting to produce the expected number of cases or deaths for that registry by age. The expected number of cases or deaths for each sex, age, and cancer were then normalised to 1, creating final, age-specific proportions. These proportions were then applied to the total number of cases or deaths by sex and cancer to get the age-specific number of cases or deaths. In the rare case that the cancer registry only contained data for both sexes combined, the age-specific cases or deaths were split and re-assigned to separate sexes using the same weights that are used for the age-splitting process. Starting from the expected number of deaths, proportions were generated by sex for each age (eg, if for ages 15–19 years old there are 6 expected deaths for males and 4 expected deaths for females, then 60% of the combined-sex deaths for ages 15–19 years would be assigned to males and the remaining 40% would be assigned to females).

In the fifth step (#5 in figure S1), data for cause entries that are aggregates of GBD causes were redistributed. Examples of these aggregated causes include some registries reporting ICD-10 codes C00-C14 together as, "lip, oral cavity, and pharyngeal cancer." These groups were broken down into sub-causes that could be mapped to single GBD causes. In this example, those include lip and oral cavity cancer (C00–C08), nasopharyngeal cancer (C11), cancer of other parts of the pharynx (C09-C10, C12–C13), and "Malignant neoplasm of other and ill-defined sites in the lip, oral cavity, and pharynx" (C14). To redistribute the data, weights were created using the same method employed in age-sex splitting (see step four above). For the undefined code (C14 in the example) an "average all cancer" weight was used, which was generated by adding all cases from SEER/NORDCAN/CI5 and dividing those by the combined population. Then, proportions were generated by sub-cause for each aggregate cause as in the sex-splitting example above (see

step four). The total number of cases from the aggregated group (C00–C14) was recalculated for each subgroup and the undefined code (C14). C14 was then redistributed as a garbage code in step six.

In the sixth step (#6 in figure S1), unspecified codes ("garbage code") were redistributed. Redistribution of cancer registry incidence and mortality data mirrored the process of the redistribution used in the cause of death database and has not changed compared to GBD 2013.[14]

In the seventh step (#7 in figure S1), duplicate or redundant sources were removed from the processed cancer registry dataset. Duplicate sources were present if, for example, the cancer registry was part of the CI5 database, but we also had data from the registry directly. Redundancies occurred and were removed as described in Vos et al.[1] where more detailed data were available, or when national registry data could replace regionally representative data. From here, two parallel selection processes were run to generate input data for the MIR models and to generate incidence for final mortality estimation. Higher priority was given to registry data from the most standardised source when creating the final incidence input, whereas for the MIR model input, only sources that reported incidence and mortality were used.

Section 3.2: Mortality-to-incidence ratio estimation[1,2]

In the eighth step (#8 in figure S1), the processed incidence and mortality data from cancer registries were matched by cancer, age, sex, year, and location to generate MIRs. These MIRs were used as input for a three-step modelling approach using the general GBD 2019 spatiotemporal Gaussian process regression (ST-GPR) approach with the Healthcare Access and Quality Index (HAQ Index) as a covariate in the linear step mixed-effects model using a logit link function.[15]

$$\text{logit}\left(MI\ ratio_{c,a,s,t}\right) = \alpha + \beta_1 HAQI_{c,t} + \sum_{\alpha}^{A} \beta_2 I_{\alpha} + \beta_3 I_s + \epsilon_{c,a,s,t}$$

c: country, a: age group, t: time (years); s: sex

HAQI: Healthcare Access and Quality index

I: indicator variable

$\epsilon_{c,a,s,t}$: Gaussian error term

Predictions were made without the random effects. The ST-GPR model has three main hyper-parameters that control for smoothing across time, age, and geography, which were adjusted for GBD 2019. The time adjustment parameter (λ) aims to borrow strength from neighbouring time points (ie, the exposure in this year is highly correlated with exposure in the previous year but less so further back in time). Lambda was lowered from 2 to 0.05, reducing the weight of more distant years. The age adjustment parameter ω was set to 0.5, which borrows strength from data in neighbouring age groups. The space adjustment parameter ξ was set to 0.01. Zeta aims to borrow strength across the hierarchy of geographical locations.[1] For the remaining parameter in the Gaussian process regression, we used 1 and for the scale, we used a value of 10.

The data cleaning has remained the same as in GBD 2017. For each cancer, MIRs from locations in HAQ quintiles 1-4 were dropped if they were below the median of MI ratios from locations in HAQ quintile 5. If the MIRs were above the third quartile + 1.5 * IQR (inter-quartile range), MIRs from locations in HAQ quintiles 1–4 were dropped. We dropped all MIRs that were based on less than 15 cases to avoid noise due to small numbers except for mesothelioma and acute lymphoid leukaemia, where we dropped MIRs that were based on less than 10 cases because of lower data availability for these two cancers. We also aggregated incidence and mortality to the youngest five-year age bin where SEER reported at least 50 cases from 1990 to 2015, to avoid unstable MIR predictions in young age groups on too few data points. The MIRs in the age-bin that was used to aggregate MIRs, were used to backfill the MIRs for younger age groups. The

MIR in the minimum age-bin was used to backfill the MIR down to the lowest age group estimated for that cancer.

Since MI ratios can be above 1, especially in older age groups and cancers with low cure rates, we used the 95th percentile of the cleaned dataset that only included MIRs that were based on 50 or more cases to cap the MIR input data. This "upper cap" was used to allow MIRs over 1 but to constrain the MIRs to a maximum level. To run the logit model, the input data were divided by the upper caps to get data from 0 to 1. Model predictions from ST-GPR were then rescaled back by multiplying them by the upper caps.

To constrain the model at the lower end, we used the 5th percentile of the cancer-specific cleaned MIR input data to replace all model predictions with this lower cap. Final MI ratios were matched with the cancer registry incidence dataset in the ninth step (#9 in figure S1) to generate mortality estimates (Incidence * Mortality/Incidence = Mortality) (#10 in figure S1). These mortality estimates are then smoothed by a Bayesian noise-reduction algorithm (to deal with problems with zero counts, as also applied to the VR and VA data) and uploaded into the CoD database (#11 in figure S1). Cancer-specific mortality modelling then followed the general CODEm process.

Section 3.3: CODEm models[2]

Mortality estimates for each cancer were generated using CODEm (#12 in figure S1). Methods describing the CODEm approach have been described previously.[1,16] In brief, the CODEm modelling approach is based on the principles that all types of available data should be used even if data quality varies; that individual models but also ensemble models should be tested for their predictive validity; and that the best model or sets of models should be chosen based on the out of sample predictive validity. Models were run separately for countries with extensive and

complete vital registration data and countries with less VR data to prevent inflation in the uncertainty around the estimates in "data-rich" countries. Covariates were selected based on a possible predictive relationship between the covariate and the specific cancer mortality.

Section 3.4: CoDCorrect[2]

CODEm estimates the individual cause-level mortality without taking into account the all-cause mortality (#13 in figure S1). To ensure that all single causes add up to the all-cause mortality and that all child-causes add up to the parent cause, an algorithm called "CoDCorrect" is used (#14 and #15 in figure S1). Details regarding the algorithm can be found in appendix 1, section 3.3.2 of Supplement 1 to Vos et al.[1]

Section 3.5: Incidence estimation[1,2]

GBD cancer incidence estimates were generated by dividing final mortality estimates (after CoDCorrect adjustment) by the MIR for specific cancer (#1 in figure S2). Final MIR estimates at the 1000-draw level were combined with final mortality estimates (also at the 1000-draw level) to generate 1000 draws of incidence estimates (which provides an estimated mean incidence with 95% uncertainty interval). It was assumed that uncertainty in the MIR is independent of uncertainty in the estimated mortality.

Section 3.6: Prevalence and YLD estimation[1,2]

Prevalence is estimated as 10-year prevalence for all cancers. After transforming the final GBD cancer mortality estimates to incidence estimates (#1 in figure S2), incidence was combined with the relative yearly survival estimates up to 10 years (#7 in figure S2). Our survival estimation methods were first implemented in GBD 2017 to more directly utilise MIRs in generating yearly cancer relative survival estimates; for GBD 2019, we updated these methods to utilise age-specific rather than all-ages survival curves. Previous reports suggest that the value of (1 – MIR)

may serve as a proxy for 5-year relative survival, with the exact correlation varying slightly by cancer type.[17] GBD 2019 used SEER*Stat to obtain national mortality, incidence, and relative survival statistics from the nine SEER registries reporting from 1980 to 2014 (#2 in Appendix Figure 2), by cancer type, sex, 5-year blocks (i.e., 1980–1984, 1985–1989, etc.), and 5-year age groups (except combining 80+). For each cancer, we modelled 5-year relative survival with the SEER MIRs. For GBD 2019, we updated this model from the Poisson regression used in GBD 2017 to using a generalized linear model with a quasibinomial family and logit link, weighted by the number of index cases (#3 in figure S2). To reduce variability due to small samples, only MIRs based on at least 25 incident cases were included (except for the rarer cancers mesothelioma, nasopharyngeal cancer, and acute myeloid leukaemia, where MIRs based on at least 10 cases were included). These models were then applied to the GBD MIR estimates to predict an estimated 5-year survival for each age/sex/year/location (#4 in figure S2). To prevent unrealistic values, predicted 5-year survival values were winsorized to be between 0% and 100% survival. Unlike GBD 2017, we did not require the estimated survival to be greater than the all ages worst-case survival scenario from SurvCan and US 1950 survival data,[18,19] since age-specific survival could be plausibly lower than for these all-ages scenarios.). To generate yearly survival estimates up to 10 years, for GBD 2019, we downloaded SEER sex- and age-specific annual 1- through 10-year relative survival data from patients diagnosed between 2001 and 2010 (compared to GBD 2017, where we downloaded all-ages survival data from 2004).[20] The proportion of the predicted GBD 5-year survival estimate to the SEER 5- year survival statistic was calculated as a scalar, and then used to generate yearly survival estimates by scaling the 1-10 year SEER curve to the GBD survival predictions under the proportional hazard assumption (#5 in figure S2). This change from GBD 2017, where we used SEER all-ages data from 2004 as the

scalar and survival curve, impacts prevalence and YLD estimation. It is generally leading to survival estimates that are higher for younger ages and lower for older ages compared to estimates using the all-ages curve.

To transform relative to absolute survival (adjusting for background mortality), GBD 2019 lifetables were used (#6 and #7 in figure S2) to calculate lambda values:

$$lambda = (ln(nLxn \;/\; nLxn + 1)) \;/\; 5$$

nLx: person years lived between ages x and x+n (from GBD lifetable)

Absolute survival was then calculated using an exponential survival function (absolute survival = relative survival*$e^{lambda*t}$). Absolute survival is combined with incidence to estimate the prevalence at each year after diagnosis, which is then split into the four sequelae (step 8 in Appendix Figure 2).

For our estimation purposes, the population that survived beyond 10 years was considered cured. The survivor population prevalence was divided into two sequelae (1. diagnosis and primary therapy, 2. controlled phase). The yearly prevalence of the population that did not survive beyond 10 years was divided into the four sequelae by assigning the fixed durations for each (1. diagnosis and primary therapy phase, 2. metastatic phase, 3. terminal phase, and assigning the remaining prevalence to the 4. controlled phase) (#8 in figure S2). Duration of these four sequelae remained the same as for GBD 2017. Table S4 lists the duration of each sequela for TBL and larynx cancer, along with the sources used to determine their length. YLDs were calculated by multiplying each phase with the respective disability weight (table S5). Details regarding the disability weights estimation method can be found in appendix 1, section 4.8 in Vos et al.[1] To generate the total YLDs for TBL cancer, the YLDs for each TBL cancer sequela were added.

Additional disability was estimated for larynx cancer (disability due to laryngectomy) (#10 in figure S2). Hospital data were used to estimate the number of cancer patients undergoing laryngectomy. Table S6 lists hospital data sources and procedure codes that were used in calculating the proportion of patients with larynx cancer undergoing laryngectomy. These proportions remained the same as in GBD 2013, GBD 2015, GBD 2016, and GBD 2017. The proportion of patients with larynx cancer that undergo laryngectomy from hospital data was used as input for a proportion model in DisMod-MR 2.1 to estimate the proportions for all locations, by age, and by sex (#9 in figure S2)

The final laryngectomy proportion was applied to the incidence cases of larynx cancer and multiplied with the proportion of the incidence population surviving for 10 years to determine the incident cases of the cancer population that underwent procedures and that survived beyond 10 years. These incident cases were used again as an input for DisMod-MR 2.1, with a remission specification of zero and an excess mortality rate prior of 0 to 0.1, as well as with increasing the age of the population and the year by 10 years to reflect prevalence after that population has survived 10 years. The results from this model are incidence and lifetime prevalent cases of persons with larynx cancer-related sequelae who have survived beyond 10 years.

We assumed that for the population surviving up to 10 years, only the prevalence population being in remission experiences additional disability due to laryngectomy (eg, a patient suffering from metastatic larynx cancer does not experience additional disability due to a laryngectomy during this phase). To estimate the prevalence of the larynx cancer population in remission during the first 10 years after diagnosis with and without laryngectomy-related disability, we multiplied the prevalence of the population with larynx cancer in the remission phase with the proportion of the population undergoing laryngectomy. This step allowed us to estimate

disability during the remission phase for both the population experiencing disability due to the remission phase alone, as well as the population experiencing disability from the remission phase and the additional laryngectomy-related disability.

Lastly, the laryngectomy sequelae prevalence and general sequelae prevalence were multiplied with their respective disability weights (table S5) to obtain the number of YLDs (#11 and #12 in figure S2). The sum of these YLDs is the final YLD estimate associated with larynx cancer.

Section 4: GBD global population age standard[1]

Age-standardised populations were calculated using the GBD world population age standard. For GBD 2019, we used the non-weighted mean of 2019 age-specific proportional distributions from GBD 2019 population estimates published by Wang and colleagues[22] for all national locations with a population greater than 5 million people in 2019.

Section 5: Risk-specific estimation

A detailed description of each risk factor and risk-specific modelling can be found in appendix 1 of Murray et al.[21]

Funding Sources

The Institute for Health Metrics and Evaluation received funding from the Bill & Melinda Gates Foundation. The content is solely the responsibility of the authors and does not necessarily represent the official views of the Bill & Melinda Gates Foundation or the National Institutes of Health.

Table S1. GATHER checklist

#	GATHER checklist item	Description of Compliance	Reference
**Objectives and funding**			
**1**	Define the indicator(s), populations (including age, sex, and geographic entities), and time period(s) for which estimates were made.	Narrative provided in paper and methods appendix (appendix 1) describing indicators, definitions, and populations	Main text (Methods) and methods appendix (Definition of indicator)
**2**	List the funding sources for the work.	Funding sources listed in paper	Main text (Summary)
**Data Inputs**			
For all data inputs from multiple sources that are synthesized as part of the study:			
**3**	Describe how the data were identified and how the data were accessed.	Narrative provided in methods appendix	Method appendix (Data sources)
**4**	Specify the inclusion and exclusion criteria. Identify all ad-hoc exclusions.	Narrative provided in methods appendix	Method appendix (Data sources)
**5**	Provide information on all included data sources and their main characteristics. For each data source used, report reference information or contact name/institution, population represented, data collection method, year(s) of data collection, sex and age range, diagnostic criteria or measurement method, and sample size, as relevant.	Metadata for data sources by component, geography, cause, risk, or impairment is available through an interactive, online data source tool	Online data citation tool: http://ghdx.healthdata.org/gbd-2019
**6**	Identify and describe any categories of input data that have potentially important biases (e.g., based on characteristics listed in item 5).	Summary of known biases included in methods appendix	Methods appendix (Bias of categories of input data)
For data inputs that contribute to the analysis but were not synthesized as part of the study:			
**7**	Describe and give sources for any other data inputs.	Included in online data source tool	Online data citation tool: http://ghdx.healthdata.org/gbd-2019
For all data inputs:			
**8**	Provide all data inputs in a file format from which data can be efficiently extracted (e.g., a spreadsheet rather than a PDF), including all relevant meta-data listed in item 5. For any data inputs that cannot be shared because of ethical or legal reasons, such as third-party ownership, provide a contact name or the name of the institution that retains the right to the data.	Downloads of input data are available through online tools, including data visualisation	Online data visualisation tools, data query tools, and the Global Health Data Exchange: http://ghdx.healthdata.org/gbd-2019
**Data analysis**			
**9**	Provide a conceptual overview of the data analysis method. A diagram may be helpful.	Flow diagrams of the overall methodological processes have been provided	Method appendix (figures S1 and S2)

**10**	Provide a detailed description of all steps of the analysis, including mathematical formulae. This description should cover, as relevant, data cleaning, data pre-processing, data adjustments and weighting of data sources, and mathematical or statistical model(s).	Flow diagrams and corresponding methodological write-ups have been provided	Main text (Methods) and methods appendix (Data Analysis)
**11**	Describe how candidate models were evaluated and how the final model(s) were selected.	Details on model evaluation and finalisation have been provided	Methods appendix
**12**	Provide the results of an evaluation of model performance, if done, as well as the results of any relevant sensitivity analysis.	Details on evaluation of model performance have been provided elsewhere	See figure S6 on p 1446 of appendix 2 in Vos et al."[1]
**13**	Describe methods for calculating uncertainty of the estimates. State which sources of uncertainty were, and were not, accounted for in the uncertainty analysis.	Details on uncertainty calculations have been provided	Methods appendix (Data Analysis)
**14**	State how analytic or statistical source code used to generate estimates can be accessed.	Code is provided in an online repository	http://ghdx.healthdata.org/gbd-2019/code
**Results and Discussion**			
**15**	Provide published estimates in a file format from which data can be efficiently extracted.	GBD 2019 results are available through online data visualization tools, the Global Health Data Exchange, and the online data query tool	http://ghdx.healthdata.org/gbd-2019
**16**	Report a quantitative measure of the uncertainty of the estimates (e.g. uncertainty intervals).	Uncertainty intervals are provided with all results in main text, methods appendix, and online	Main manuscript (Results), results appendix (appendix 2), and data tools: http://ghdx.healthdata.org/gbd-2019
**17**	Interpret results in light of existing evidence. If updating a previous set of estimates, describe the reasons for changes in estimates.	Discussion has been provided in the main text	Main manuscript (Discussion)
**18**	Discuss limitations of the estimates. Include a discussion of any modelling assumptions or data limitations that affect interpretation of the estimates.	Discussion of limitations has been provided	Main manuscript (Discussion) and methods appendix

Table S2. List of International Classification of Diseases codes mapped to Global Burden of Disease cause list for tracheal, bronchus, and lung (TBL), and larynx cancer incidence and mortality[1]

Cause		ICD-10	ICD-9
Tracheal, bronchus, and lung cancer	Incidence	C33, C34–C34.92, Z12.2, Z80.1–Z80.2, Z85.1–Z85.20	162–162.9, 209.21, V10.1–V10.20, V16.1–V16.2, V16.4–V16.40
	Mortality	C33–C34.9, D02.1–D02.3, D14.2–D14.3, D38.1	162–162.9, 212.2–212.3, 231.1–231.2, 235.7
Larynx cancer	Incidence	C32–C32.9, Z85.21	161–161.9, V10.21
	Mortality	C32–C32.9, D02.0, D14.1, D38.0	161–161.9, 212.1, 231.0, 235.6

ICD = International Classification of Diseases.

Table S3. Duration of four prevalence phases by cancer

Cancer	Diagnosis/treatment (months)	Remission	Disseminated/metastatic (months)	Note	Terminal (months)
TBL cancer	3.3[22]	Calculated based on remainder of time after attributing other sequelae.	4.51[23]	SEER Summary Stage 1997 (Distant site/node involved) 1995-2000	1month
Larynx cancer	5.3[24]		8.84[23]	SEER Stage IVc	

Table S4. Disabilities weights

Health state	Lay description	Estimate	95% uncertainty interval
Cancer, diagnosis and primary therapy	Has pain, nausea, fatigue, weight loss and high anxiety.	0.288	0.193-0.399
Cancer, controlled phase	Has a chronic disease that requires medication every day and causes some worry but minimal interference with daily activities.	0.049	0.031-0.072
Cancer, metastatic	Has severe pain, extreme fatigue, weight loss and high anxiety.	0.451	0.307-0.600
Terminal phase, with medication	Has lost a lot of weight and regularly uses strong medication to avoid constant pain. The person has no appetite, feels nauseous, and needs to spend most of the day in bed.	0.540	0.377-0.687
Laryngectomy	Has difficulty speaking, and others find it difficult to understand.	0.051	0.032-0.078

Table S5. Sources of hospital data[25]

Sequela	Cancer	Sources	Procedure code (ICD-9-CM)
Laryngectomy	Larynx cancer	SEER (03-08)[26]<br>Canada hospital data (94-09)[27]<br>Mexico hospital data (01-09)[28]<br>USA hospital data[29]	301. 303, 304, 3029

ICD = International Classification of Diseases.

Table S6. Definition and input sources for risk factors used in Global Burden of Disease estimations for tracheal, bronchus, and lung (TBL), and larynx cancers[21]

Risk		Outcome	Definition	Input data
Metabolic	High fasting plasma glucose	TBL cancer	Mean FPG in a population, where FPG is a continuous exposure in units of mmol/L.	- Estimates of mean FPG in a representative population<br>- Individual-level data of fasting plasma glucose measured from surveys<br>- Estimates of diabetes prevalence in a representative population
Environmental	Ambient particulate matter pollution	TBL cancer	Population-weighted annual average mass concentration of particles with an aerodynamic diameter less than 2.5 micrometers ($PM_{2.5}$) in one cubic meter of air (μg/m^3).	- $PM_{2.5}$ ground measurement database<br>- Satellite-based estimates<br>-Population data<br>- Chemical transport model simulations<br>- land-use data
	Household air pollution from solid fuels	TBL cancer	The proportion of individuals using solid cooking fuels (coal, wood, charcoal, dung, and agricultural residues) and the level of $PM_{2.5}$ air pollution exposure for these individuals	- Multi-country survey series<br>- WHO Energy Database
	Residential radon	TBL cancer	Average daily exposure to indoor air radon gas levels measured in Becquerels (disintegrations per second) per cubic meter (Bq/m^3).	- Literature review<br>- Government agency reports<br>- Monitoring station reports<br>- National surveys
	Occupational exposure to asbestos	TBL cancer/ larynx cancer	Proportion of the population occupationally exposed to asbestos, using mesothelioma death rate as an analogue.	- GBD 2019 cause of death estimates<br>- Published studies
	Occupational exposure to arsenic	TBL cancer	Proportion of the population that was ever occupationally exposed to carcinogens in high or low exposure level, based on population distributions across 17 economic activities	- International Labour Organization (ILO) data<br>- GBD Collaborator Network
	Occupational exposure to beryllium	TBL cancer	Proportion of the population that was ever occupationally exposed to carcinogens in high or low exposure level, based on population distributions across 17 economic activities	- International Labour Organization (ILO) data<br>- GBD Collaborator Network

	Occupational exposure to cadmium	TBL cancer	Proportion of the population that was ever occupationally exposed to carcinogens in high or low exposure level, based on population distributions across 17 economic activities	- International Labour Organization (ILO) data<br>- GBD Collaborator Network
	Occupational exposure to chromium	TBL cancer	Proportion of the population that was ever occupationally exposed to carcinogens in high or low exposure level, based on population distributions across 17 economic activities	- International Labour Organization (ILO) data<br>- GBD Collaborator Network
	Occupational exposure to diesel engine exhaust	TBL cancer	Proportion of the population that was ever occupationally exposed to carcinogens in high or low exposure level, based on population distributions across 17 economic activities	- International Labour Organization (ILO) data<br>- GBD Collaborator Network
	Occupational exposure to nickel	TBL cancer	Proportion of the population that was ever occupationally exposed to carcinogens in high or low exposure level, based on population distributions across 17 economic activities	- International Labour Organization (ILO) data<br>- GBD Collaborator Network
	Occupational exposure to polycyclic aromatic hydrocarbons	TBL cancer	Proportion of the population that was ever occupationally exposed to carcinogens in high or low exposure level, based on population distributions across 17 economic activities	- International Labour Organization (ILO) data<br>- GBD Collaborator Network
	Occupational exposure to silica	TBL cancer	Proportion of the population that was ever occupationally exposed to carcinogens in high or low exposure level, based on population distributions across 17 economic activities	- International Labour Organization (ILO) data<br>- GBD Collaborator Network
	Occupational exposure to sulfuric acid	Larynx cancer	Proportion of the population that was ever occupationally exposed to carcinogens in high or low exposure level, based on population distributions across 17 economic activities	- International Labour Organization (ILO) data<br>- GBD Collaborator Network
**Behavioural**	Smoking	TBL cancer/ larynx cancer	Current smoker: Individuals who currently use any smoked tobacco product on a daily or occasional basis.<br>Former smoker: Individuals who quit using akk smoked tobacco products for at least six months (where possible) or according to the definition used by survey	- Primary data from individual level microdata<br>- Survey report tabulations
	Secondhand smoke	TBL cancer	Current exposure to secondhand tobacco smoke at home, at work, or in other public places. Only non-smokers considered to be exposed to secondhand smoke. Non-smokers are defined as all persons who are not daily smokers. Ex-smokers and occasional smokers are considered non-smokers in this analysis.	- Representative major survey series with a household composition module<br>- Cross-sectional surveys
	Diets low in fruits	TBL cancer	Average daily consumption of less than 310-340 grams per day of fruits (fresh, frozen, cooked, canned, or dried, excluding fruit juices and salted or pickled fruits).	- Dietary recall sources from literature search of PubMed

				- IHME Global Health Data Exchange (GHDx) yearly known survey series<br>- Nationally and sub-nationally representative nutrition surveys<br>- Household budget surveys<br>- Accounts of national sales<br>- United Nations FAO Food Balance Sheets and Supply and Utilization Accounts
	Alcohol use	Larynx cancer	Grams per day of pure alcohol consumed among current drinkers.<br>- Current drinker: Proportion of individuals who have consumed at least one alcoholic beverage in a 12-month period<br>- Alcohol consumption: Grams of alcohol consumed by current drinker, per day, over a 12-month period<br>-Alcohol litres per capita stock: Litres per capita of pure alcohol, over a 12-month period	- Global Health Data Exchange (GHDx)<br>- WHO GISAH database<br>- FAOSTAT<br>- Retail supply (Euromonitor)<br>- Data on the number of tourists and their duration of stay from the UNWTO

Figure S1. Flowchart of GBD 2019 cancer mortality and YLL estimation

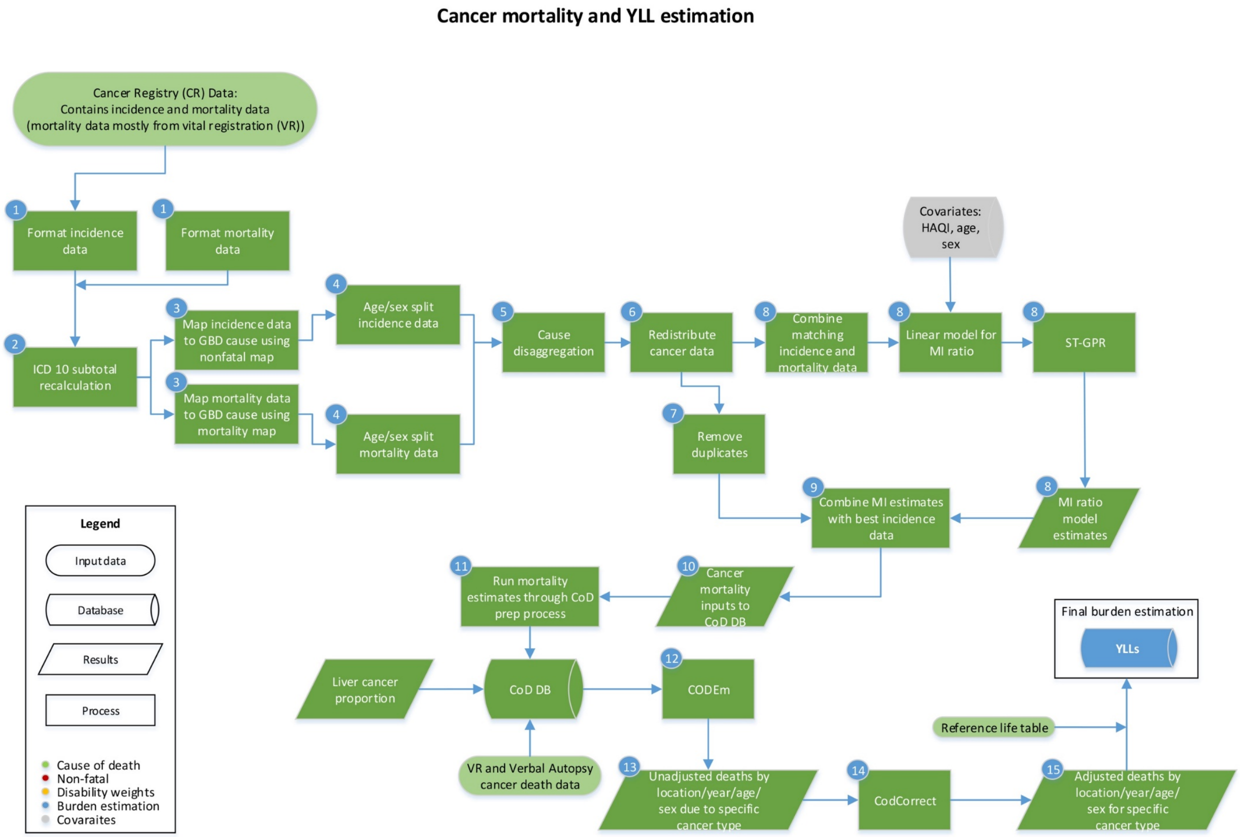

Figure S2. Flowchart of GBD 2019 cancer incidence, prevalence, and YLD estimation

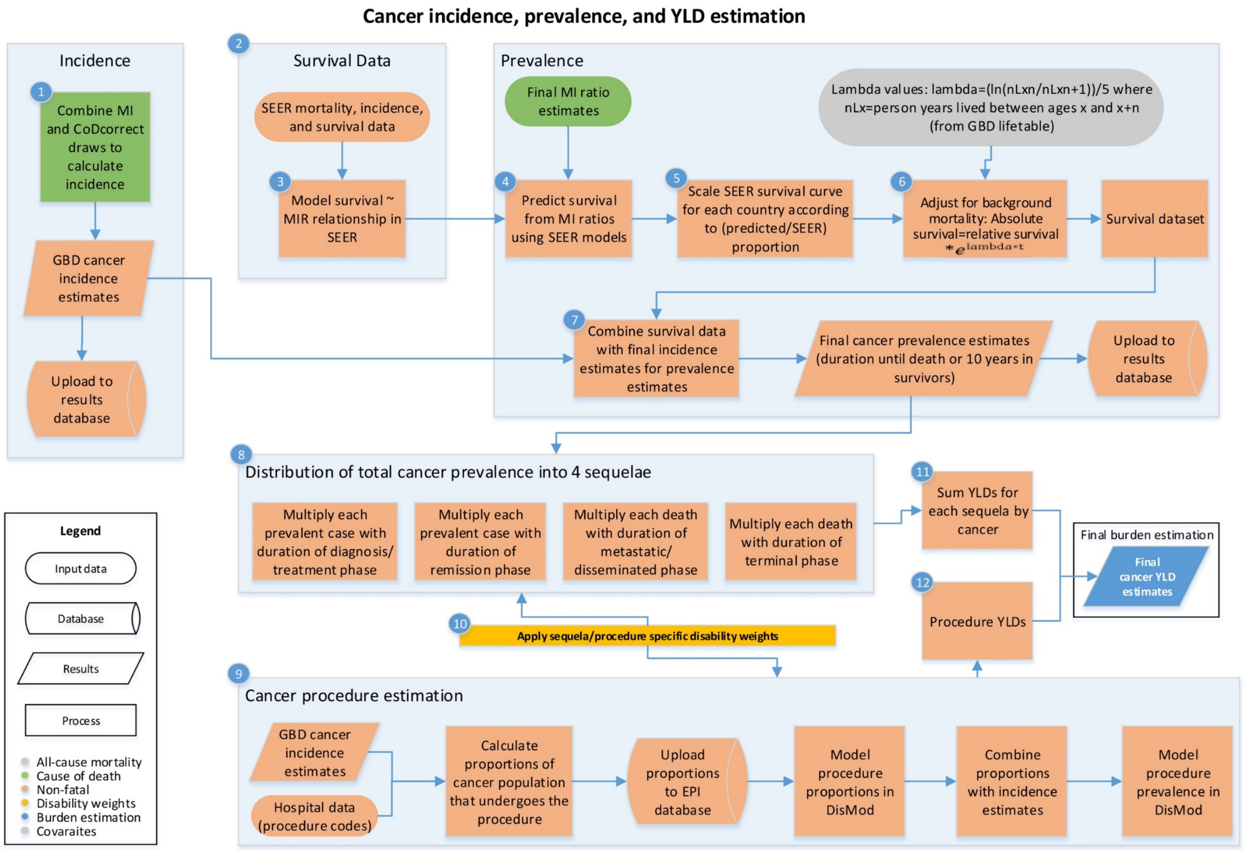

References

1. Global burden of 369 diseases and injuries in 204 countries and territories, 1990-2019: a systematic analysis for the Global Burden of Disease Study 2019. *Lancet (London, England)* 2020; **396**(10258): 1204-22.
2. Doll R, Payne P, Waterhouse J. Cancer Incidence in Five Continents I. Geneva: Union Internationale Contre le Cancer, 1966.
3. Doll R, Muir C, Waterhouse J. Cancer Incidence in Five Continents II. Geneva: Union Internationale Contre le Cancer, 1970.
4. Waterhouse J, Muir C, Correa P, Powell J. Cancer Incidence in Five Continents III. Lyon: IARC, 1976.
5. Waterhouse J, Muir C, Shanmugaratnam K, Powell J. Cancer Incidence in Five Continents IV. Lyon: IARC, 1982.
6. Muir C, Mack T, Powell J, Whelan S. Cancer Incidence in Five Continents V. Lyon: IARC, 1987.
7. Parkin D, Muir C, Whelan S, Gao Y, Ferlay J, Powell J. Cancer Incidence in Five Continents VI. Lyon: IARC, 1992.
8. Parkin D, Whelan S, Ferlay J, Raymond L, Young J. Cancer Incidence in Five Continents VII. Lyon: IARC, 1997.
9. Parkin D, Whelan S, Ferlay J, Teppo L, Thomas D. Cancer Incidence in Five Continents VIII. Lyon: IARC, 2002.
10. Curado M, Edwards B, Shin H, et al. Cancer Incidence in Five Continents IX. Lyon: IARC, 2007.
11. Forman D, Bray F, Brewster D, et al. Cancer Incidence in Five Continents X. Lyon: IARC, 2014.
12. Steliarova-Foucher E, O'Callaghan M, Ferlay J, et al. The European Cancer Observatory: A new data resource. *European journal of cancer (Oxford, England : 1990)* 2015; **51**(9): 1131-43.
13. Engholm G, Ferlay J, Christensen N, et al. NORDCAN--a Nordic tool for cancer information, planning, quality control and research. *Acta oncologica (Stockholm, Sweden)* 2010; **49**(5): 725-36.
14. Global, regional, and national age-sex specific all-cause and cause-specific mortality for 240 causes of death, 1990-2013: a systematic analysis for the Global Burden of Disease Study 2013. *Lancet (London, England)* 2015; **385**(9963): 117-71.
15. Healthcare Access and Quality Index based on mortality from causes amenable to personal health care in 195 countries and territories, 1990-2015: a novel analysis from the Global Burden of Disease Study 2015. *Lancet (London, England)* 2017; **390**(10091): 231-66.
16. Foreman KJ, Lozano R, Lopez AD, Murray CJ. Modeling causes of death: an integrated approach using CODEm. *Population health metrics* 2012; **10**: 1.
17. Asadzadeh Vostakolaei F, Karim-Kos HE, Janssen-Heijnen ML, Visser O, Verbeek AL, Kiemeney LA. The validity of the mortality to incidence ratio as a proxy for site-specific cancer survival. *European journal of public health* 2011; **21**(5): 573-7.
18. Sankaranarayanan R, Swaminathan R, Lucas E. Cancer survival in Africa, Asia, the Caribbean and Central America (SurvCan).
19. National Center for Health Statistics CfDCa, Prevention. US Mortality Files. 61-Year Trends in U.S. Cancer Death Rates. http://seer.cancer.gov/archive/csr/1975_2010/results_merged/topic_historical_mort_trends.pdf.
20. SEER Cancer Statistics Review 1975-2011. http://seer.cancer.gov/csr/1975_2011/results_merged/topic_survival_by_year_dx.pdf.
21. Global burden of 87 risk factors in 204 countries and territories, 1990-2019: a systematic analysis for the Global Burden of Disease Study 2019. *Lancet (London, England)* 2020; **396**(10258): 1223-49.

22. Allgar VL, Neal RD. Delays in the diagnosis of six cancers: analysis of data from the National Survey of NHS Patients: Cancer. *British journal of cancer* 2005; **92**(11): 1959-70.
23. Surveillance, Epidemiology, and End Results (SEER) Program (http://www.seer.cancer.gov/) SEER*StatDatabase: Incidence - SEER 18 Regs Research Data + Hurricane Katrina Impacted Louisiana Cases,Nov 2012 Sub (1973-2010 Varying) - Linked To County Attributes - Total U.S., 1969-2011 Counties: National Cancer Institute, DCCPS, Surveillance Research Program, Surveillance Systems Branch, Released April 2013, Based on the November 2012 Submission.
24. Neal RD, Din NU, Hamilton W, et al. Comparison of cancer diagnostic intervals before and after implementation of NICE guidelines: analysis of data from the UK General Practice Research Database. *British journal of cancer* 2014; **110**(3): 584-92.
25. Fitzmaurice C, Dicker D, Pain A, et al. The Global Burden of Cancer 2013. *JAMA oncology* 2015; **1**(4): 505-27.
26. National Cancer Institute (United States). United States SEER Cancer Data 1973-2010. Bethesda, United States: National Cancer Institute (United States).
27. Canadian Institute for Health Information (CIHI). Canada Discharge Abstract Database 1994-2009. Ottawa, Canada: Canadian Institute for Health Information (CIHI).
28. Ministry of Health (Mexico). Mexico Ministry of Health Hospital Discharges 2000-2012. Mexico City, México: Ministry of Health (Mexico).
29. National Center for Health Statistics (NCHS), Centers for Disease Control and Prevention (CDC), US Census Bureau. United States National Hospital Discharge Survey. Hyattsville, United States: National Center for Health Statistics (NCHS), Centers for Disease Control and Prevention (CDC).
