# Supplementary material for: Global, regional, and national burden of respiratory tract cancers and associated risk factors from 1990 to 2019: a systematic analysis for the Global Burden of Disease Study 2019
Source: Lancet Respir Med. 2021 Sep;9(9):1030–49. doi: 10.1016/S2213-2600(21)00164-8 (PMC8410610; doi:10.1016/S2213-2600(21)00164-8)

THE LANCET
Respiratory Medicine

Supplementary appendix 2

This appendix formed part of the original submission and has been peer reviewed. We post it as supplied by the authors.

Supplement to: GBD 2019 Respiratory Tract Cancers Collaborators. Global, regional, and national burden of respiratory tract cancers and associated risk factors from 1990 to 2019: a systematic analysis for the Global Burden of Disease Study 2019. *Lancet Respir Med* 2021; published online August 16. http://dx.doi.org/10.1016/S2213-2600(21)00164-8.

Supplementary appendix 2

Results appendix to "Global, regional, and national burden of respiratory tract cancers and associated risk factors from 1990 to 2019: a systematic analysis for the Global Burden of Disease Study 2019"

This appendix provides more detailed results for "Global, regional, and national burden of respiratory tract cancers and associated risk factors from 1990 to 2019: a systematic analysis for the Global Burden of Disease Study 2019".

List of Appendix tables and figure

Appendix tables

Table S1. Incidence, deaths, and disability-adjusted life-years (DALYs) in 2019 (counts and age-standardised rates) and percent change in age-standardised rates between 2010 and 2019, at global, regional, national levels, and Socio-demographic Index (SDI) quintiles, by sex and for both sexes combined, for tracheal, bronchus, and lung (TBL) cancer. 3

Table S2. Incidence, deaths, and disability-adjusted life-years (DALYs) in 2019 (counts and age-standardised rates) and percent change in age-standardised rates between 2010 and 2019, at global, regional, national levels, and Socio-demographic Index (SDI) quintiles, by sex and for both sexes combined, for larynx cancer. 41

Table S3. Years lived with disability (YLDs) and years of life lost (YLLs) in 2019 (counts and age-standardised rates), and percent change in age-standardised rates between 2010 and 2019, at global, regional, national levels, and Socio-demographic Index (SDI) quintiles, by sex and for both sexes combined, for tracheal, bronchus, and lung (TBL), and larynx cancer. 78

Table S4. Tracheal, bronchus, and lung (TBL) cancer deaths (counts and age-standardised rates) attributable to specific risk factors in 2019, at global, regional, national levels, and Socio-demographic Index (SDI) quintiles, by sex and for both sexes combined. 137

Table S5. Larynx cancer deaths (counts and age-standardised rates) attributable to specific risk factors in 2019, at global, regional, national levels, and Socio-demographic Index (SDI) quintiles, by sex and for both sexes combined. 204

Appendix figure

Figure S1. Annualised rate of change in the age-standardised death rate of larynx cancer attributable to smoking, 2010–2019. 238

Table S1. Incidence, deaths, and disability-adjusted life-years (DALYs) in 2019 (counts and age-standardised rates) and percent change in age-standardised rates between 2010 and 2019, at global, regional, national levels, and Socio-demographic Index (SDI) quintiles, by sex and for both sexes combined, for tracheal, bronchus, and lung (TBL) cancer.

95% UIs given in parentheses.

Location	Sex	Incidence			Deaths			DALYs		
		Number in 2019	Age-standardised rate per 100,000 in 2019	Percent change in age-standardised rate from 2010 to 2019 (%)	Number in 2019	Age-standardised rate per 100,000 in 2019	Percent change in age-standardised rate from 2010 to 2019 (%)	Number in 2019	Age-standardised rate per 100,000 in 2019	Percent change in age-standardised rate from 2010 to 2019 (%)
Global	Both	2260000 (2070000 to 2450000)	27.7 (25.3 to 30)	-4.6 (-12.5 to 3.3)	2040000 (1880000 to 2190000)	25.2 (23.2 to 27)	-5.7 (-12.6 to 1.5)	45900000 (42300000 to 49300000)	551.6 (509 to 593.1)	-7.3 (-14.6 to 0.4)
	Male	1520000 (1370000 to 1680000)	40.4 (36.5 to 44.4)	-7.4 (-16.8 to 1.6)	1390000 (1260000 to 1510000)	37.4 (34.1 to 40.7)	-8.7 (-16.8 to 0.2)	31600000 (28600000 to 34700000)	802.9 (727.6 to 879.8)	-10 (-18.6 to -0.4)
	Female	737000 (658000 to 814000)	16.8 (15 to 18.6)	0.9 (-8.2 to 10.2)	657000 (590000 to 719000)	15 (13.5 to 16.4)	0.1 (-8.2 to 8.6)	14300000 (13000000 to 15700000)	327.6 (298.5 to 360.4)	-1.3 (-10 to 7.7)
High SDI	Both	709000 (637000 to 773000)	37.4 (33.9 to 40.8)	-8.7 (-15.8 to -0.5)	578000 (534000 to 603000)	29.8 (27.8 to 31)	-8.9 (-11 to -6.9)	11300000 (10800000 to 11700000)	636 (606.9 to 656.8)	-11 (-13.3 to -8.8)
	Male	431000 (389000 to 478000)	49.7 (44.8 to 55)	-12.8 (-20.7 to -3.6)	354000 (333000 to 367000)	40.6 (38.3 to 42.1)	-13.1 (-15 to -11.1)	7030000 (6730000 to 7260000)	835.7 (801.8 to 863.2)	-14.8 (-16.8 to -12.6)
	Female	278000 (244000 to 310000)	27.3 (24.2 to 30.5)	-3.4 (-13.3 to 6.9)	224000 (201000 to 237000)	21.1 (19.4 to 22.1)	-3.7 (-6.7 to -0.7)	4310000 (4010000 to 4510000)	461.9 (436.4 to 481.2)	-5.5 (-8.7 to -2.3)
High-middle SDI	Both	671000 (603000 to 735000)	32.6 (29.3 to 35.7)	-4.9 (-14.7 to 5.8)	614000 (559000 to 670000)	29.9 (27.2 to 32.6)	-7.1 (-15.6 to 2.3)	14000000 (12700000 to 15300000)	680.7 (620.1 to 743.5)	-9.6 (-17.9 to -0.2)
	Male	479000 (422000 to 539000)	51.9 (45.7 to 58.4)	-9.3 (-20.4 to 2.7)	439000 (390000 to 491000)	48.3 (43.1 to 53.9)	-11.4 (-21.2 to 0.1)	10100000 (8960000 to 11300000)	1064.2 (943.6 to 1187.2)	-13.4 (-23.4 to -1.9)
	Female	193000 (168000 to 222000)	17.1 (15 to 19.7)	4.8 (-8.2 to 21.2)	175000 (154000 to 200000)	15.4 (13.6 to 17.6)	2.1 (-10.5 to 17.6)	3870000 (3430000 to 4420000)	355.1 (314.9 to 404.8)	-0.6 (-13.3 to 14.5)

Middle SDI	Both	580000 (509000 to 650000)	23.7 (20.8 to 26.5)	1.2 (-11.1 to 14.1)	630000 (551000 to 712000)	26.3 (23 to 29.7)	-1.7 (-13.8 to 11.6)	14900000 (13000000 to 16900000)	579.4 (506.1 to 655)	-3.1 (-15.5 to 10.3)
	Male	377000 (320000 to 435000)	32.5 (27.6 to 37.4)	-1.5 (-16.5 to 14.8)	436000 (368000 to 509000)	38.7 (32.8 to 45)	-3.4 (-18 to 13.2)	10400000 (8760000 to 12200000)	844.4 (711.1 to 986)	-4.7 (-19.6 to 12.2)
	Female	203000 (172000 to 236000)	15.9 (13.5 to 18.5)	7.5 (-7.5 to 24.1)	194000 (165000 to 224000)	15.5 (13.2 to 17.8)	3.7 (-11.8 to 19.5)	4470000 (3810000 to 5200000)	336.7 (287.3 to 390.8)	2.3 (-13.4 to 19.2)
Low-middle SDI	Both	170000 (153000 to 186000)	12.6 (11.3 to 13.8)	4.8 (-6 to 14.5)	174000 (158000 to 190000)	13.2 (12 to 14.3)	3.7 (-6.4 to 12.7)	4400000 (3980000 to 4830000)	308.2 (278.6 to 337.5)	2.9 (-7.5 to 12.6)
	Male	118000 (106000 to 131000)	18.3 (16.4 to 20.2)	1.6 (-10.9 to 14.5)	122000 (110000 to 134000)	19.3 (17.5 to 21.2)	0.6 (-10.7 to 12)	3080000 (2780000 to 3400000)	447.9 (405.3 to 492.4)	-0.4 (-12 to 11.4)
	Female	51800 (45000 to 58900)	7.4 (6.4 to 8.4)	15.8 (1.9 to 29.5)	52500 (45800 to 59600)	7.7 (6.7 to 8.7)	14.3 (0.3 to 28.3)	1320000 (1140000 to 1510000)	178.5 (154.4 to 204.3)	14 (0 to 28.8)
Low SDI	Both	40800 (35100 to 48600)	8.1 (7 to 9.5)	4.3 (-6.2 to 15.2)	46000 (39600 to 53900)	9.4 (8.1 to 10.9)	3 (-6.9 to 13.4)	1220000 (1040000 to 1450000)	219.9 (188.7 to 259.9)	2.7 (-7.8 to 13.9)
	Male	29800 (24800 to 37200)	12.1 (10.1 to 14.9)	-0.2 (-11.4 to 13)	34700 (29000 to 42200)	14.7 (12.4 to 17.5)	-1.2 (-12.3 to 11.3)	910000 (754000 to 1130000)	337.3 (282.2 to 412.8)	-1.4 (-13.2 to 12.1)
	Female	11000 (9620 to 12400)	4.2 (3.7 to 4.8)	20.4 (6.1 to 35.9)	11300 (9780 to 12700)	4.5 (3.9 to 5.1)	20.2 (4.9 to 36.5)	306000 (264000 to 348000)	106.4 (92.5 to 120.6)	19.6 (4.7 to 35.9)
Central Asia	Both	14300 (13000 to 15900)	18.9 (17.1 to 20.8)	-6.5 (-14.9 to 2.6)	14000 (12700 to 15500)	19 (17.3 to 20.9)	-7 (-15.1 to 1.8)	387000 (349000 to 430000)	473.7 (428.6 to 523.6)	-9 (-17.7 to 0.7)
	Male	11300 (9980 to 12600)	34.4 (30.7 to 38.1)	-8.4 (-17.3 to 1.6)	11000 (9720 to 12200)	35 (31.2 to 38.7)	-8.7 (-17.5 to 1.1)	305000 (270000 to 343000)	845.2 (750.3 to 941)	-11.1 (-20.2 to -1)
	Female	3090 (2770 to 3450)	7.4 (6.7 to 8.3)	3.2 (-7.3 to 14.5)	3040 (2720 to 3380)	7.5 (6.8 to 8.4)	2.1 (-7.9 to 13.1)	82200 (73000 to 92400)	185.3 (165.2 to 207.5)	1 (-10.3 to 12.8)
Central Europe	Both	84000 (74200 to 94800)	40 (35.2 to 45.2)	-6.3 (-17.5 to 5.7)	81300 (71400 to 92200)	38.3 (33.6 to 43.5)	-6.7 (-18.1 to 5.4)	1900000 (1670000 to 2170000)	946 (823.6 to 1081.4)	-9 (-20.8 to 3.5)
	Male	59800 (51800 to 68500)	64.3 (55.6 to 73.4)	-10.9 (-23.2 to 1.7)	58000 (50100 to 66500)	62.3 (54 to 71.4)	-11.3 (-23.1 to 1)	1370000 (1180000 to 1580000)	1491.8 (1283.4 to 1714.8)	-13.4 (-25.5 to -0.9)

	Female	24200 (21000 to 27700)	20.9 (18.1 to 24)	4.2 (-9.4 to 19.7)	23400 (20400 to 26500)	19.6 (17.1 to 22.4)	4 (-9.3 to 18.7)	528000 (459000 to 605000)	490 (424.3 to 563.6)	1.5 (-11.8 to 16.7)
Eastern Europe	Both	86300 (76600 to 97000)	25 (22.2 to 28.1)	-8.4 (-18.8 to 2.5)	78700 (69600 to 88000)	22.7 (20.1 to 25.3)	-10.3 (-20.6 to 0.2)	1980000 (1750000 to 2220000)	588.5 (518.6 to 658.3)	-11.2 (-21.6 to -0.3)
	Male	69200 (59600 to 79400)	51.1 (44.1 to 58.4)	-13.3 (-25 to -1.2)	63100 (54500 to 72000)	47.3 (40.9 to 53.9)	-14.8 (-26.2 to -3.1)	1620000 (1400000 to 1860000)	1176.2 (1015.8 to 1345.3)	-15.4 (-27.1 to -3.2)
	Female	17100 (14700 to 19900)	8.2 (7 to 9.6)	6.3 (-9.5 to 25.3)	15600 (13400 to 18100)	7.3 (6.2 to 8.5)	3.2 (-12 to 20.6)	358000 (305000 to 419000)	183.1 (154.9 to 215.4)	2.7 (-13.3 to 21.2)
Australasia	Both	15300 (12500 to 18700)	30.7 (25 to 37.5)	-8.6 (-25.2 to 11.4)	12000 (11100 to 12800)	23.8 (22.1 to 25.1)	-9 (-13 to -4.7)	237000 (223000 to 250000)	500.9 (473 to 526.3)	-9.7 (-13.6 to -5.5)
	Male	8830 (7170 to 11000)	37.6 (30.4 to 46.8)	-13.2 (-29.9 to 8.6)	7070 (6560 to 7530)	29.9 (27.8 to 31.8)	-12.8 (-17.5 to -7.8)	138000 (129000 to 146000)	605.8 (568.5 to 641.5)	-13.1 (-18.1 to -8.3)
	Female	6470 (5230 to 7780)	24.8 (20.1 to 30)	-2.7 (-19.6 to 18.2)	4970 (4420 to 5380)	18.6 (16.8 to 20)	-4.2 (-9.8 to 2.1)	99700 (91100 to 107000)	407.8 (376.3 to 436.6)	-4.9 (-10.4 to 1.2)
High-income Asia Pacific	Both	151000 (127000 to 173000)	31.6 (27 to 36.3)	-9.5 (-19.5 to 2.4)	111000 (96100 to 119000)	22.2 (19.7 to 23.6)	-10.5 (-12.9 to -8.3)	1830000 (1650000 to 1930000)	427.4 (395.7 to 448.3)	-13.6 (-16.2 to -11)
	Male	106000 (89300 to 125000)	50.6 (42.9 to 59.6)	-14 (-26.5 to 0.6)	76800 (69000 to 81500)	36.2 (32.8 to 38.3)	-15.4 (-18 to -12.9)	1320000 (1220000 to 1390000)	670.8 (624.5 to 704.7)	-17.5 (-20.5 to -14.6)
	Female	45100 (35700 to 53400)	16.5 (13.6 to 19.2)	-2.4 (-15.3 to 11.7)	34200 (27100 to 38200)	11.4 (9.6 to 12.4)	-3.4 (-6.9 to 0.3)	512000 (438000 to 556000)	219.8 (197.3 to 234.7)	-6.6 (-10.4 to -2.9)
High-income North America	Both	285000 (250000 to 325000)	45 (39.5 to 51.4)	-10.2 (-21.3 to 1.7)	230000 (216000 to 239000)	35.9 (33.8 to 37.3)	-9.7 (-12.1 to -7.5)	4670000 (4460000 to 4820000)	762 (729.6 to 787.1)	-11.1 (-13.5 to -8.8)
	Male	154000 (129000 to 184000)	52.9 (44.4 to 63.3)	-11.9 (-25.8 to 4.7)	127000 (121000 to 131000)	43.7 (41.7 to 45.2)	-11.4 (-13.3 to -8.8)	2610000 (2520000 to 2690000)	909.9 (879.9 to 938.5)	-12.5 (-14.4 to -9.7)
	Female	132000 (110000 to 157000)	38.5 (31.9 to 46)	-9 (-24.1 to 8.6)	104000 (94600 to 110000)	29.5 (27.2 to 31.4)	-8.5 (-12.9 to -4.3)	2060000 (1930000 to 2180000)	634.5 (595.6 to 669.5)	-9.7 (-14.4 to -5.4)
Southern Latin America	Both	19700 (15700 to 24500)	23.6 (18.8 to 29.5)	-4.7 (-24.8 to 19.7)	19600 (18500 to 20600)	23.5 (22 to 24.6)	-6 (-10.5 to -1.5)	442000 (418000 to 465000)	540.9 (512.1 to 569)	-8 (-12.8 to -3.4)

	Male	13100 (10300 to 16400)	35.4 (28 to 44.4)	-9.9 (-28.8 to 13)	13100 (12400 to 13800)	35.7 (33.7 to 37.6)	-11.1 (-15.8 to -6.3)	298000 (283000 to 314000)	800.6 (760.3 to 843.3)	-12.9 (-17.4 to -8)
	Female	6580 (5280 to 8200)	14.3 (11.4 to 17.8)	7 (-14.4 to 35)	6560 (5990 to 7010)	14 (12.8 to 14.9)	5.3 (-2.3 to 13.6)	144000 (133000 to 154000)	325.7 (301.6 to 348.7)	3.2 (-4.3 to 11.7)
Western Europe	Both	301000 (263000 to 341000)	34.5 (30.1 to 39.4)	-8.3 (-19.6 to 3.7)	261000 (244000 to 270000)	29 (27.4 to 30)	-8.9 (-11.1 to -6.9)	5270000 (5030000 to 5450000)	654 (628.1 to 675.5)	-11 (-13.5 to -8.7)
	Male	197000 (171000 to 225000)	49.1 (42.5 to 56.2)	-13.9 (-25.5 to -1.6)	174000 (165000 to 180000)	42.5 (40.5 to 44.1)	-14.3 (-16.8 to -12.1)	3520000 (3380000 to 3650000)	920.9 (882.8 to 954.3)	-15.8 (-18.6 to -13.4)
	Female	103000 (90500 to 118000)	22.4 (19.6 to 25.6)	1.4 (-10.5 to 15.4)	87000 (79600 to 91500)	17.9 (16.7 to 18.6)	0.5 (-2 to 3)	1750000 (1640000 to 1820000)	419 (398.5 to 434.6)	-1.6 (-4.3 to 1.2)
Andean Latin America	Both	5970 (4810 to 7310)	10.8 (8.7 to 13.2)	-5.6 (-24.3 to 17)	6240 (5040 to 7620)	11.4 (9.2 to 13.9)	-6.7 (-24.9 to 14.9)	139000 (111000 to 172000)	245.1 (195.4 to 303)	-7.6 (-27 to 15.8)
	Male	3280 (2610 to 4090)	12.5 (9.9 to 15.5)	-9.2 (-29.2 to 14.4)	3450 (2760 to 4290)	13.3 (10.6 to 16.5)	-10.2 (-29.5 to 12.3)	76200 (60300 to 95700)	280.1 (221.2 to 350.6)	-10.6 (-30.8 to 13)
	Female	2690 (2180 to 3300)	9.3 (7.5 to 11.3)	-0.7 (-20.7 to 22.5)	2790 (2250 to 3410)	9.7 (7.8 to 11.8)	-1.9 (-21.5 to 20.2)	62800 (50200 to 77400)	212.7 (170.2 to 262.6)	-3.6 (-23.9 to 21.1)
Caribbean	Both	11500 (9820 to 13500)	22.2 (19 to 26)	0.2 (-14 to 16.6)	11200 (9630 to 13100)	21.7 (18.6 to 25.3)	-1.1 (-14.9 to 14.6)	254000 (216000 to 299000)	489.1 (415.7 to 575.1)	-1.4 (-16.1 to 15.1)
	Male	7520 (6410 to 8820)	31.1 (26.6 to 36.3)	-0.9 (-15.2 to 15.6)	7290 (6240 to 8510)	30.3 (26 to 35.4)	-2.3 (-15.8 to 13.3)	166000 (139000 to 195000)	673.7 (568.5 to 791)	-2 (-16.4 to 15)
	Female	3990 (3370 to 4670)	14.5 (12.2 to 16.9)	3 (-11.9 to 20.2)	3940 (3350 to 4610)	14.2 (12.1 to 16.6)	2 (-12.8 to 18.2)	88400 (74800 to 104000)	323.9 (273.9 to 382)	0.6 (-14.7 to 18.1)
Central Latin America	Both	27200 (23200 to 31800)	11.7 (10 to 13.6)	-2.1 (-16.3 to 13)	27200 (23300 to 31900)	11.8 (10.1 to 13.8)	-3.6 (-17.4 to 12.3)	620000 (525000 to 735000)	259.9 (220.3 to 307.2)	-3.6 (-18.3 to 13.8)
	Male	15900 (13400 to 18800)	15 (12.6 to 17.6)	-5.7 (-19.7 to 10.7)	16400 (13900 to 19400)	15.6 (13.2 to 18.4)	-6.5 (-20.7 to 10.5)	371000 (310000 to 445000)	337 (282.2 to 402.3)	-6.8 (-22.1 to 11.7)
	Female	11300 (9330 to 13700)	8.9 (7.4 to 10.8)	4.7 (-11.8 to 25.2)	10900 (8970 to 12900)	8.6 (7.2 to 10.3)	2.2 (-13.4 to 20.2)	249000 (204000 to 302000)	193.8 (159.2 to 234.2)	2.6 (-14.2 to 22.3)
Tropical Latin America	Both	37300 (35000 to 39100)	15.4 (14.5 to 16.2)	-6.7 (-10.4 to -3)	37900 (35400 to 39800)	15.8 (14.7 to 16.6)	-7 (-10.4 to -3.4)	888000 (844000 to 929000)	359.3 (341 to 376.3)	-9.1 (-12.6 to -5.5)

	Male	21800 (20500 to 23000)	20.3 (19 to 21.4)	-12.6 (-17.1 to -8.2)	22300 (21000 to 23600)	21.1 (19.7 to 22.3)	-12.7 (-17 to -8.3)	519000 (491000 to 547000)	462.6 (437 to 487.7)	-15 (-19.1 to -10.6)
	Female	15500 (14300 to 16400)	11.6 (10.7 to 12.3)	3.7 (-2.1 to 9.5)	15600 (14300 to 16600)	11.7 (10.8 to 12.5)	3.2 (-2.4 to 9)	368000 (344000 to 392000)	275.1 (256.4 to 292.9)	1.1 (-4.6 to 6.7)
North Africa and Middle East	Both	71700 (63400 to 81000)	16.8 (14.9 to 19)	1 (-10.4 to 14)	72500 (64100 to 81900)	17.5 (15.6 to 19.8)	0.6 (-10.5 to 13.3)	1870000 (1650000 to 2120000)	406.7 (359.1 to 459.5)	-1.4 (-12.8 to 11.2)
	Male	55800 (49100 to 63500)	25.8 (22.7 to 29.2)	-3.2 (-14.8 to 10.5)	57100 (50300 to 64700)	27.2 (24 to 30.6)	-3.1 (-14.3 to 10.1)	1470000 (1290000 to 1680000)	629.3 (554.1 to 713.7)	-5.1 (-16.9 to 8.6)
	Female	15800 (13600 to 18200)	7.6 (6.6 to 8.7)	17.9 (2.9 to 32.1)	15400 (13300 to 17700)	7.7 (6.6 to 8.8)	15.5 (0.8 to 29.3)	398000 (343000 to 459000)	174.9 (151.1 to 201.6)	13.9 (-0.9 to 27.8)
South Asia	Both	117000 (100000 to 134000)	8.4 (7.1 to 9.5)	6.6 (-8.9 to 22.4)	120000 (101000 to 137000)	8.8 (7.4 to 10.1)	6 (-9.4 to 21.3)	3090000 (2620000 to 3550000)	208.5 (176.6 to 239.6)	5 (-10.6 to 20.6)
	Male	85400 (69700 to 102000)	12.4 (10.2 to 14.7)	0.3 (-17.8 to 20.3)	87200 (71200 to 103000)	13 (10.7 to 15.3)	-0.2 (-18.1 to 18.4)	2240000 (1840000 to 2650000)	306.7 (251 to 362)	-1.3 (-19.9 to 18)
	Female	31800 (26000 to 38200)	4.5 (3.7 to 5.4)	31.6 (6.5 to 61.1)	32500 (26600 to 39100)	4.7 (3.9 to 5.6)	30.8 (5 to 60.4)	847000 (692000 to 1030000)	112.8 (92.2 to 136.9)	29.9 (3.8 to 60.2)
East Asia	Both	855000 (721000 to 1000000)	41.3 (35 to 48.1)	0.3 (-16.9 to 19.2)	778000 (658000 to 907000)	38.4 (32.7 to 44.6)	-4 (-19.2 to 14.1)	17600000 (14800000 to 20700000)	825.8 (696.8 to 969.5)	-5.3 (-21.4 to 13.7)
	Male	590000 (467000 to 723000)	61.1 (48.9 to 73.9)	-1 (-21.7 to 24)	537000 (427000 to 661000)	57.5 (46.3 to 69.7)	-5.4 (-24.9 to 17.4)	12300000 (9700000 to 15300000)	1195.5 (950 to 1478)	-6.3 (-27.1 to 18.2)
	Female	265000 (213000 to 322000)	24.6 (19.8 to 29.9)	4.2 (-16.1 to 29.6)	242000 (196000 to 289000)	22.7 (18.5 to 27.2)	-0.4 (-20.1 to 22.5)	5330000 (4310000 to 6510000)	489.7 (396.1 to 597.2)	-1.6 (-22.5 to 22.3)
Oceania	Both	1480 (1120 to 2100)	21.6 (16.6 to 30.4)	4.1 (-8.7 to 19.6)	1490 (1130 to 2110)	22.9 (17.8 to 32)	4.2 (-8.6 to 19.5)	41900 (31300 to 59700)	539.6 (407.5 to 764)	3.7 (-9.6 to 21.2)
	Male	1170 (857 to 1720)	33.5 (25.2 to 48.6)	1.9 (-11.3 to 18.6)	1170 (865 to 1730)	35.8 (27.2 to 51.5)	1.8 (-11.2 to 18.1)	33200 (23900 to 49600)	827.3 (611.2 to 1218.6)	1.9 (-12 to 19.6)
	Female	313 (234 to 438)	9.5 (7.1 to 13.2)	11.7 (-4.1 to 31.4)	316 (236 to 443)	10 (7.5 to 13.9)	11.8 (-3.7 to 31.9)	8780 (6490 to 12400)	236 (176.1 to 330.2)	10.7 (-6 to 31)
Southeast Asia	Both	133000 (111000 to 154000)	22 (18.4 to 25.4)	2.4 (-10.1 to 16.3)	135000 (113000 to 156000)	23 (19.2 to 26.6)	1.7 (-10.4 to 13.6)	3430000 (2860000 to 3970000)	532.9 (445.9 to 615.8)	0.4 (-12.1 to 12.9)

	Male	91200 (76900 to 106000)	33.2 (28.2 to 38.4)	0.7 (-13.4 to 16)	92400 (78500 to 107000)	35 (29.8 to 40.4)	0 (-13.3 to 13.6)	2380000 (2000000 to 2770000)	795.2 (672.7 to 921.6)	-1.2 (-15.1 to 13.9)
	Female	41300 (31600 to 51800)	12.8 (9.8 to 15.9)	6.5 (-8.4 to 22.1)	42200 (32100 to 52600)	13.4 (10.2 to 16.6)	6 (-9 to 20.7)	1050000 (797000 to 1320000)	308.5 (233.1 to 387.3)	4.3 (-10.9 to 20.3)
Central Sub-Saharan Africa	Both	6800 (4410 to 11700)	12.7 (8.4 to 21.2)	6.4 (-11 to 29.6)	6890 (4470 to 11700)	13.5 (8.9 to 22.3)	6.4 (-10.3 to 29.7)	195000 (126000 to 339000)	328.9 (212.8 to 560.8)	5.3 (-12.3 to 30)
	Male	5190 (3140 to 9930)	21.9 (13.5 to 41.1)	0.2 (-16 to 22.5)	5250 (3200 to 9990)	23.3 (14.5 to 43.5)	0.2 (-15.4 to 22.7)	150000 (89500 to 289000)	554.4 (338.7 to 1050.2)	-0.7 (-16.8 to 23.1)
	Female	1610 (1210 to 2190)	5.6 (4.2 to 7.8)	24.3 (-1.9 to 56.9)	1640 (1230 to 2240)	6 (4.5 to 8.4)	24.2 (-1.3 to 56)	45100 (33400 to 60800)	142.5 (106.9 to 194.1)	23.1 (-2.9 to 56.2)
Eastern sub-Saharan Africa	Both	10900 (9170 to 13300)	7 (5.9 to 8.5)	3.1 (-7.8 to 14.2)	11400 (9630 to 13900)	7.6 (6.5 to 9.1)	3.1 (-7.7 to 14.3)	298000 (248000 to 366000)	173.3 (145.5 to 211)	2.4 (-9.1 to 14.2)
	Male	7890 (6530 to 9830)	10.9 (9.1 to 13.5)	-0.9 (-12.9 to 11.4)	8290 (6930 to 10300)	11.9 (10 to 14.6)	-0.8 (-12.5 to 11.7)	212000 (175000 to 266000)	263.2 (219.6 to 326.8)	-1.7 (-14.2 to 11.8)
	Female	3050 (2520 to 3630)	3.6 (3 to 4.3)	17.8 (2.4 to 34.7)	3160 (2610 to 3720)	3.9 (3.3 to 4.5)	17.4 (2.6 to 34.4)	86400 (70200 to 104000)	92.2 (75.7 to 109.5)	16.9 (1.2 to 34.5)
Southern sub-Saharan Africa	Both	10300 (9340 to 11500)	18.4 (16.7 to 20.3)	-10.3 (-18.8 to 0.7)	10500 (9520 to 11600)	19.2 (17.5 to 21.2)	-10.2 (-18.2 to 1.2)	271000 (244000 to 304000)	455.7 (411.9 to 509.5)	-13.3 (-22.1 to -1.1)
	Male	6930 (6170 to 7910)	29.4 (26.4 to 33.3)	-13.3 (-22.9 to 0.1)	6980 (6260 to 7960)	30.8 (27.8 to 34.7)	-13.7 (-22.7 to -0.1)	189000 (168000 to 217000)	732.2 (653.7 to 838.6)	-16.3 (-25.7 to -2.1)
	Female	3350 (2980 to 3720)	10.5 (9.4 to 11.7)	-4.4 (-15 to 8.1)	3490 (3100 to 3870)	11.2 (10 to 12.3)	-3.5 (-13.8 to 8.3)	82500 (72300 to 92500)	248 (218.2 to 277.3)	-6.9 (-18.4 to 7)
Western sub-Saharan Africa	Both	16200 (13600 to 18900)	9.2 (7.7 to 10.6)	2.5 (-12.3 to 19.8)	16900 (14200 to 19900)	10 (8.5 to 11.6)	2.4 (-11.5 to 18.5)	427000 (354000 to 512000)	218 (183.1 to 257.6)	1.9 (-12.5 to 19.6)
	Male	11600 (9570 to 13800)	13.9 (11.6 to 16.4)	0.9 (-15.6 to 23.7)	12200 (10000 to 14600)	15.1 (12.6 to 18)	0.6 (-15.5 to 21.4)	302000 (246000 to 368000)	328.1 (269.5 to 396.6)	0.6 (-16.6 to 22.2)
	Female	4580 (3770 to 5450)	4.9 (4.1 to 5.7)	18.5 (-1.9 to 41.4)	4720 (3900 to 5620)	5.2 (4.4 to 6.2)	18.4 (-0.8 to 40.7)	125000 (101000 to 151000)	116.9 (95.9 to 140)	17.8 (-3.2 to 43.1)
Afghanistan	Both	1480 (871 to 2340)	11.7 (7.3 to 18.5)	0.6 (-17.1 to 27.3)	1490 (892 to 2360)	12.5 (8 to 19.7)	0.5 (-17.1 to 26.7)	44600 (25100 to 71300)	302.6 (179.9 to 481.2)	0.9 (-17.6 to 29.7)
	Male	1110 (618 to 1930)	19.2 (11.3 to 33)	-2.7 (-20 to 24.4)	1130 (641 to 1950)	20.6 (12.3 to 35)	-2.4 (-19.3 to 24.4)	32800 (17500 to 57900)	486 (274.1 to 844.6)	-3.6 (-21.1 to 24.9)

	Female	365 (237 to 524)	5.1 (3.6 to 7.1)	18.9 (-8.1 to 53.5)	359 (238 to 512)	5.3 (3.9 to 7.3)	18.8 (-8 to 52.7)	11700 (7200 to 17400)	139.7 (92.8 to 198.6)	18 (-8.5 to 51.4)
Albania	Both	1170 (862 to 1570)	27.1 (20 to 36)	14.7 (-14.9 to 50.8)	1160 (856 to 1530)	26.6 (19.7 to 35.2)	12.9 (-15.8 to 47.8)	25900 (18900 to 34800)	615 (448.4 to 824)	11.4 (-18.2 to 47.9)
	Male	937 (679 to 1280)	45.2 (32.8 to 61)	14.4 (-16.3 to 51.7)	924 (670 to 1250)	44.8 (32.9 to 60.3)	12.4 (-16.7 to 48)	20800 (14800 to 28700)	1016.1 (729.6 to 1393.3)	11.5 (-19.2 to 49.2)
	Female	237 (178 to 308)	10.7 (8 to 13.9)	18.9 (-11.2 to 53.7)	234 (175 to 302)	10.4 (7.8 to 13.4)	16.4 (-12.3 to 50)	5110 (3780 to 6710)	241.8 (179.5 to 319.1)	16 (-14.1 to 50.7)
Algeria	Both	3190 (2460 to 4110)	9.8 (7.6 to 12.5)	-3 (-23.8 to 23)	3290 (2540 to 4240)	10.5 (8.2 to 13.4)	-3.4 (-24 to 22.6)	81400 (61900 to 105000)	231.8 (178 to 298.7)	-4.8 (-25.9 to 22.1)
	Male	2480 (1840 to 3310)	14.8 (11.1 to 19.8)	-7.3 (-29.4 to 20.4)	2590 (1930 to 3460)	15.9 (12 to 21.2)	-7.4 (-28.9 to 20.1)	63200 (46400 to 84700)	355.6 (263 to 474)	-8.4 (-30.7 to 20.6)
	Female	716 (563 to 888)	4.5 (3.6 to 5.4)	11.1 (-12.7 to 38.6)	706 (559 to 867)	4.6 (3.7 to 5.6)	9.1 (-13.6 to 35.5)	18200 (14200 to 22600)	102.8 (80.7 to 127.5)	7.9 (-16.1 to 35.8)
American Samoa	Both	12 (11 to 14)	26.5 (23 to 30.1)	-0.8 (-13.2 to 12.8)	13 (11 to 14)	28 (24.3 to 31.7)	-0.8 (-12.9 to 12.8)	317 (267 to 369)	637.6 (541.4 to 739.5)	-1 (-14 to 13.7)
	Male	9 (7 to 10)	38.6 (33.4 to 43.7)	-5.4 (-18.5 to 8.8)	9 (8 to 10)	41 (35.8 to 46.4)	-5.7 (-18.4 to 7.9)	222 (186 to 259)	920.3 (784.4 to 1063.1)	-5.1 (-18.8 to 10.8)
	Female	4 (3 to 5)	15.7 (12.5 to 19.6)	11.5 (-11.4 to 39.9)	4 (3 to 5)	16.5 (13.1 to 20.6)	11.5 (-11.3 to 40.2)	95 (75 to 120)	374.2 (295.8 to 471)	11.5 (-10.8 to 39.8)
Andorra	Both	55 (43 to 70)	39.5 (30.5 to 49.9)	-4.3 (-22.7 to 18.5)	48 (37 to 60)	34.2 (26.5 to 42.9)	-4.9 (-22.6 to 16.9)	1090 (832 to 1380)	783.3 (599.5 to 994.8)	-5.7 (-24.2 to 16.5)
	Male	44 (34 to 55)	62.1 (48.5 to 77.8)	-6.6 (-23.7 to 14.8)	38 (30 to 48)	55 (43.1 to 68.7)	-7.1 (-24 to 13.4)	862 (666 to 1100)	1212.8 (937.2 to 1542)	-7.7 (-25.2 to 13.7)
	Female	11 (8 to 16)	16.6 (11.7 to 22.8)	9.5 (-19.5 to 48.1)	10 (7 to 13)	13.7 (9.7 to 18.6)	9.1 (-19.9 to 45.4)	224 (158 to 303)	333.4 (235.9 to 451.1)	7.8 (-20.5 to 43.6)
Angola	Both	1570 (1220 to 2010)	14 (11.3 to 17.6)	3.6 (-16 to 30)	1580 (1240 to 2030)	14.9 (12.1 to 18.6)	3.3 (-15.2 to 30.4)	45200 (34400 to 58800)	358.7 (280.5 to 460.2)	2.2 (-17.2 to 31.5)
	Male	1190 (914 to 1540)	23.8 (19.1 to 29.8)	1.2 (-18.5 to 28.5)	1200 (931 to 1550)	25.4 (20.5 to 31.4)	0.9 (-18.1 to 27.2)	34300 (26000 to 44900)	603.8 (471.5 to 772.8)	0.1 (-20.2 to 29.2)
	Female	379 (285 to 511)	6.3 (4.8 to 8.3)	21.4 (-6.4 to 59.2)	382 (290 to 513)	6.7 (5.1 to 8.8)	21.6 (-4.7 to 57.5)	10800 (7960 to 14700)	157.8 (119.4 to 212.7)	19.4 (-7.8 to 57.3)
Antigua and Barbuda	Both	10 (8 to 11)	9.5 (8.2 to 11)	-2.4 (-16 to 14)	9 (8 to 11)	9.6 (8.3 to 11.1)	-3 (-16.4 to 13.7)	220 (186 to 258)	209.8 (178 to 244.8)	-4.2 (-18.5 to 14)
	Male	6 (5 to 7)	12.9 (10.9 to 15.1)	-11.4 (-25.1 to 7.3)	6 (5 to 7)	13.1 (11.1 to 15.3)	-11.9 (-25.3 to 6.6)	138 (116 to 164)	279.2 (234.6 to 329.5)	-13.5 (-27.5 to 5.9)
	Female	4 (3 to 4)	6.6 (5.5 to 7.8)	14.2 (-5.4 to 38.7)	4 (3 to 4)	6.7 (5.6 to 7.9)	13.5 (-4.9 to 36.3)	82 (67 to 98)	148.4 (123.1 to 177.7)	13.1 (-7.1 to 39.1)
Argentina	Both	13900 (11000 to 17400)	25.9 (20.5 to 32.5)	-3.6 (-24.4 to 21.7)	13900 (13000 to 14700)	25.7 (24.1 to 27.3)	-5.1 (-11.2 to 1.2)	319000 (298000 to 339000)	607.6 (569 to 644.8)	-7.2 (-13.3 to -0.8)

	Male	9440 (7380 to 11900)	39.8 (31.2 to 49.9)	-8.2 (-27.8 to 15.4)	9460 (8840 to 10100)	40.1 (37.5 to 42.8)	-9.5 (-15.5 to -2.7)	219000 (204000 to 234000)	915.5 (855.4 to 978.7)	-11.6 (-17.6 to -4.9)
	Female	4420 (3540 to 5590)	14.9 (11.9 to 19)	6.8 (-16.1 to 36.5)	4420 (3990 to 4850)	14.6 (13.2 to 16.1)	5 (-5.7 to 16.1)	100000 (90400 to 110000)	353.4 (320.5 to 388.5)	2.9 (-7.6 to 14)
Armenia	Both	1350 (1130 to 1600)	31.8 (26.7 to 37.7)	-9.5 (-23.7 to 7.3)	1330 (1120 to 1580)	31.6 (26.7 to 37.3)	-10.1 (-23.5 to 6.3)	32500 (27100 to 38700)	772.1 (644 to 919.5)	-12.4 (-26.6 to 4.7)
	Male	1110 (925 to 1320)	60.7 (51 to 72.1)	-10.5 (-25 to 6.5)	1090 (916 to 1300)	60.6 (51 to 71.7)	-11 (-25.1 to 5.3)	27000 (22400 to 32400)	1456.8 (1215.4 to 1753.3)	-13.3 (-27.6 to 3.9)
	Female	241 (201 to 285)	10.2 (8.5 to 12.1)	-4.8 (-21.8 to 13.6)	243 (202 to 285)	10.2 (8.5 to 12)	-5.6 (-22 to 12.5)	5540 (4560 to 6580)	242.6 (198.6 to 288.3)	-8 (-25.1 to 10.8)
Australia	Both	12800 (10000 to 16100)	30.4 (23.9 to 38.4)	-9.4 (-28.7 to 14.2)	10000 (9210 to 10700)	23.5 (21.7 to 25)	-9.4 (-14.2 to -4.5)	196000 (183000 to 208000)	491.6 (461.2 to 520.1)	-10.1 (-14.8 to -5)
	Male	7520 (5760 to 9600)	38 (29.2 to 48.5)	-14 (-33.4 to 9.9)	6010 (5540 to 6450)	30.1 (27.9 to 32.3)	-13.2 (-18.5 to -7.3)	117000 (108000 to 125000)	608.5 (567.6 to 650.2)	-13.5 (-19.2 to -7.7)
	Female	5280 (4110 to 6610)	24 (18.8 to 30.3)	-3.1 (-23.7 to 23)	4030 (3560 to 4400)	17.8 (15.9 to 19.3)	-4.4 (-11.3 to 3.1)	79800 (72400 to 86600)	387.1 (353.3 to 418.9)	-4.9 (-12.2 to 2)
Austria	Both	5120 (4160 to 6260)	30.5 (24.7 to 37.4)	-10.5 (-27.5 to 10.5)	4210 (3920 to 4460)	24.3 (22.9 to 25.7)	-10.6 (-15.6 to -5.7)	90300 (85200 to 95400)	566.3 (533.5 to 596.7)	-13.7 (-19 to -8.7)
	Male	3100 (2500 to 3810)	40 (32.2 to 49.2)	-18.2 (-34.7 to 1.7)	2630 (2440 to 2810)	33.4 (31.2 to 35.7)	-17.8 (-23.6 to -12)	56200 (52400 to 60000)	744.8 (693.6 to 795.4)	-20.5 (-26.5 to -14.7)
	Female	2020 (1650 to 2490)	22.7 (18.6 to 28.1)	2.3 (-17.3 to 25.7)	1590 (1440 to 1730)	17 (15.5 to 18.5)	1.2 (-7.1 to 9.6)	34200 (31200 to 37100)	412.3 (379.5 to 446.5)	-1.3 (-9.6 to 7.6)
Azerbaijan	Both	2380 (1750 to 3040)	23.5 (17.4 to 29.5)	0.2 (-18.8 to 22.7)	2300 (1690 to 2920)	23.6 (17.5 to 29.7)	-0.2 (-18.5 to 22)	67400 (49100 to 86500)	615.5 (453.5 to 778.5)	-1.9 (-21.2 to 20.6)
	Male	1920 (1300 to 2560)	41.4 (28.3 to 54.1)	0.1 (-22.6 to 28.1)	1850 (1250 to 2450)	41.6 (28.5 to 54)	-0.3 (-22.8 to 27)	55000 (37100 to 73900)	1083.2 (739 to 1437)	-1.8 (-24.8 to 26.9)
	Female	454 (350 to 577)	8.9 (7 to 11.4)	2.7 (-18 to 28.6)	444 (346 to 567)	9.2 (7.1 to 11.7)	2.3 (-18.2 to 27)	12400 (9430 to 15600)	222.5 (172.7 to 281.1)	-0.7 (-22.1 to 25.7)
Bahamas	Both	57 (47 to 71)	14.5 (11.9 to 17.9)	-4.9 (-22.1 to 17.1)	56 (46 to 69)	14.5 (11.9 to 17.8)	-4.7 (-21.7 to 17.2)	1480 (1190 to 1860)	354.7 (287 to 443)	-5.1 (-23.1 to 18.9)
	Male	40 (32 to 51)	22.5 (18.3 to 28)	-6.6 (-25.1 to 16.2)	39 (32 to 49)	22.7 (18.5 to 28.1)	-6.4 (-24.9 to 15.2)	1050 (834 to 1330)	543.9 (437.5 to 687.9)	-6.6 (-26.1 to 17.6)
	Female	17 (14 to 21)	8 (6.4 to 9.8)	-1.8 (-20.4 to 21.6)	17 (14 to 21)	8 (6.5 to 9.9)	-2 (-20.1 to 21.1)	436 (344 to 546)	195 (154.3 to 243.7)	-2 (-21.6 to 22.1)
Bahrain	Both	141 (107 to 186)	19.8 (15.2 to 25.1)	-18.1 (-35.6 to 2.3)	142 (107 to 187)	22.1 (17.1 to 27.7)	-18.8 (-36 to 1.2)	3550 (2680 to 4750)	392.1 (295 to 503.8)	-19.2 (-37 to 1.7)
	Male	104 (75 to 142)	26.5 (19.9 to 34.3)	-23.2 (-41.5 to -0.5)	106 (78 to 144)	30.4 (23 to 39.1)	-23.1 (-41.1 to -0.4)	2680 (1930 to 3710)	518.9 (386.9 to 684.8)	-24.5 (-43.2 to -0.7)

	Female	37 (29 to 45)	12.5 (10 to 15.1)	-10.9 (-28.2 to 6.9)	35 (28 to 43)	13 (10.5 to 15.8)	-13.5 (-30.1 to 4.4)	869 (682 to 1070)	238.5 (190 to 289.9)	-12.6 (-30.4 to 6.2)
Bangladesh	Both	9650 (6330 to 15100)	7.4 (4.9 to 11.6)	-2.8 (-28.3 to 26.9)	9970 (6570 to 15600)	7.8 (5.2 to 12.1)	-3.6 (-28.8 to 26)	246000 (158000 to 385000)	181.7 (117.7 to 284.3)	-3.7 (-28.9 to 25.9)
	Male	7500 (4820 to 12000)	11.1 (7.1 to 17.7)	-5.2 (-33.2 to 26.5)	7770 (5020 to 12500)	11.6 (7.6 to 18.6)	-6.2 (-33.5 to 25.8)	189000 (119000 to 310000)	271.2 (172.1 to 441.5)	-5.9 (-34.2 to 26.4)
	Female	2150 (1420 to 3060)	3.4 (2.3 to 4.9)	18.2 (-8.5 to 48.5)	2210 (1450 to 3160)	3.6 (2.4 to 5.2)	17.2 (-9.3 to 45.9)	57100 (38100 to 81400)	85.3 (56.8 to 121.2)	16.8 (-10.6 to 48.4)
Barbados	Both	48 (39 to 57)	9.7 (8 to 11.5)	2.8 (-17 to 23.5)	48 (40 to 57)	9.7 (8 to 11.5)	2.7 (-16.6 to 22.4)	1050 (849 to 1260)	217.4 (175.7 to 261.9)	0.2 (-19.9 to 22.4)
	Male	31 (25 to 37)	13.9 (11.3 to 16.7)	3.6 (-17.3 to 26.2)	31 (25 to 37)	13.9 (11.3 to 16.6)	3.4 (-16.9 to 24.9)	679 (541 to 830)	307 (244.8 to 373.5)	0.7 (-21.2 to 24.1)
	Female	17 (14 to 21)	6.2 (5 to 7.6)	-1.5 (-21.2 to 20.3)	17 (14 to 21)	6.3 (5.1 to 7.6)	-2 (-20.8 to 18.7)	370 (295 to 455)	142 (113.4 to 175.1)	-2.3 (-21.9 to 21.6)
Belarus	Both	3800 (2920 to 4940)	23.9 (18.5 to 31.1)	-13.5 (-33.3 to 12.5)	3540 (2750 to 4590)	22.2 (17.3 to 28.7)	-16.4 (-35.3 to 8.4)	89500 (68300 to 118000)	572 (437.2 to 748.1)	-18 (-37.5 to 7.2)
	Male	3240 (2490 to 4230)	51.9 (40.3 to 67.2)	-16.2 (-35.3 to 8.6)	3020 (2330 to 3910)	49 (38.2 to 63.2)	-18.9 (-37.3 to 4.3)	77900 (59500 to 102000)	1210.7 (929.2 to 1577.7)	-20.3 (-39.2 to 3.7)
	Female	560 (425 to 725)	5.7 (4.3 to 7.5)	-0.4 (-25.6 to 30.2)	527 (403 to 672)	5.3 (4 to 6.9)	-5 (-28.4 to 23.2)	11600 (8660 to 15300)	128.4 (94.9 to 170.9)	-6.1 (-31.1 to 25.7)
Belgium	Both	8680 (6830 to 10900)	39.8 (31 to 50.6)	-6.7 (-27.9 to 17.6)	7900 (7370 to 8360)	35.1 (32.9 to 37.1)	-8.2 (-13 to -3)	162000 (152000 to 171000)	793.4 (745.7 to 840.7)	-11.6 (-16.7 to -6.2)
	Male	6060 (4750 to 7720)	59.8 (46.9 to 76.1)	-12.1 (-31.5 to 10.7)	5600 (5210 to 5950)	54.5 (51 to 57.8)	-13.4 (-18.8 to -7.9)	113000 (106000 to 120000)	1155.6 (1084.1 to 1223.2)	-16.4 (-21.2 to -11.1)
	Female	2620 (2060 to 3290)	23.4 (18.1 to 29.8)	3.6 (-19.9 to 31.4)	2300 (2090 to 2490)	19.5 (17.9 to 21.1)	2.2 (-5.8 to 10.3)	48900 (45100 to 52900)	477.1 (440.2 to 517.4)	-1.3 (-9.2 to 7.4)
Belize	Both	37 (32 to 43)	13.3 (11.4 to 15.3)	0.4 (-14.5 to 16.4)	37 (31 to 42)	13.5 (11.6 to 15.4)	0 (-13.9 to 15.6)	979 (834 to 1140)	328 (280.8 to 378.6)	0 (-14.7 to 16.5)
	Male	27 (23 to 32)	19.4 (16.3 to 22.5)	1.9 (-14.3 to 20.4)	27 (23 to 31)	19.6 (16.7 to 22.8)	1.4 (-14.3 to 20.2)	715 (598 to 840)	474.9 (399 to 556.4)	1.8 (-14.9 to 21.6)
	Female	10 (8 to 11)	7.1 (6 to 8.2)	-2 (-16.6 to 16.1)	10 (8 to 11)	7.2 (6.1 to 8.4)	-2.4 (-16.7 to 14.4)	264 (225 to 312)	176.7 (149.8 to 207.4)	-2 (-17.3 to 16.9)
Benin	Both	485 (370 to 634)	10.5 (8.1 to 13.6)	0.3 (-17.9 to 22.4)	508 (392 to 664)	11.4 (8.9 to 14.6)	0.2 (-17.2 to 21.3)	12800 (9480 to 17100)	252.2 (191.4 to 331.7)	-0.5 (-19 to 21.7)
	Male	351 (263 to 464)	16.7 (12.6 to 21.7)	-3.8 (-21.3 to 16.6)	368 (275 to 483)	18.3 (13.8 to 23.7)	-3.8 (-20.7 to 16.9)	9180 (6760 to 12400)	392.9 (296.2 to 521.2)	-4.9 (-23 to 17.4)
	Female	134 (104 to 175)	5.3 (4.1 to 6.8)	10.5 (-10.4 to 35.2)	140 (108 to 181)	5.7 (4.5 to 7.3)	10.6 (-10.4 to 35.5)	3640 (2680 to 4850)	129.7 (98.3 to 169.3)	10.2 (-11.8 to 36.1)

Bermuda	Both	35 (30 to 42)	26.8 (22.6 to 32.3)	-7.1 (-20.2 to 10.2)	32 (27 to 38)	24 (20.4 to 28.8)	-9.3 (-21.7 to 7.9)	646 (544 to 783)	521.5 (437.4 to 631.1)	-10.5 (-23.7 to 7.1)
	Male	24 (21 to 29)	42.6 (36.5 to 50.8)	-9.8 (-23.1 to 6.8)	22 (19 to 26)	38 (32.4 to 44.9)	-12.1 (-24.7 to 3.6)	460 (390 to 550)	811.4 (684 to 970.7)	-13.3 (-26.4 to 3.6)
	Female	10 (8 to 13)	14.1 (11.3 to 18.1)	-2.8 (-19.5 to 17.6)	10 (8 to 13)	13.1 (10.4 to 16.6)	-5.2 (-20.9 to 14.7)	186 (149 to 237)	275.4 (220.4 to 351.4)	-5.3 (-22.1 to 16.2)
Bhutan	Both	43 (31 to 58)	7.7 (5.6 to 10.6)	6.8 (-11.2 to 27.7)	44 (32 to 61)	8.3 (6 to 11.3)	6.5 (-11.4 to 26.5)	1070 (764 to 1470)	184.2 (132.5 to 253.2)	5 (-13 to 26.7)
	Male	31 (20 to 44)	11.1 (7.1 to 15.8)	4.1 (-14.4 to 25.2)	33 (21 to 46)	11.9 (7.6 to 16.8)	3.8 (-14.2 to 24.7)	774 (504 to 1090)	261.1 (167.9 to 370.9)	2.5 (-15.7 to 24.4)
	Female	12 (8 to 18)	4.3 (2.9 to 6.7)	17.7 (-3.9 to 46.7)	12 (8 to 19)	4.5 (3.1 to 7.2)	17.2 (-4.4 to 46.2)	297 (194 to 475)	103.6 (68.5 to 165)	15.4 (-5.8 to 45.2)
Bolivia	Both	1150 (746 to 1580)	13.4 (8.6 to 18.3)	7.4 (-13.4 to 29.3)	1220 (782 to 1670)	14.5 (9.4 to 19.9)	7.3 (-13.4 to 29.4)	28200 (18600 to 38600)	311.4 (204.6 to 427.4)	5.1 (-16.4 to 28.5)
	Male	693 (424 to 988)	17.2 (10.4 to 24.4)	5.5 (-15.8 to 29.2)	741 (450 to 1050)	18.9 (11.4 to 26.8)	5.3 (-16 to 28.8)	16900 (10700 to 23800)	393.2 (243.8 to 556.9)	3.5 (-18 to 28)
	Female	454 (303 to 623)	10 (6.7 to 13.7)	10 (-10.5 to 32.7)	475 (318 to 653)	10.7 (7.2 to 14.8)	9.9 (-10.3 to 31.8)	11400 (7650 to 15500)	238.2 (160.7 to 324.7)	7.4 (-14.2 to 31.2)
Bosnia and Herzegovina	Both	2440 (1890 to 3060)	40.3 (31.3 to 50.7)	-1.8 (-24.3 to 23.9)	2390 (1860 to 2990)	39.4 (30.9 to 49.4)	-2.5 (-24.7 to 22.7)	56700 (43600 to 71900)	957.9 (736.3 to 1213.4)	-3.9 (-26.6 to 22.5)
	Male	1860 (1420 to 2350)	68.2 (52.3 to 85.8)	-5.7 (-27.7 to 20.4)	1830 (1400 to 2310)	67.5 (52.2 to 84.9)	-6.2 (-27.6 to 19.8)	43800 (33300 to 55300)	1596.5 (1221.5 to 2016.3)	-7.7 (-30.2 to 18.4)
	Female	575 (450 to 725)	17.6 (13.7 to 22.2)	9.8 (-14.7 to 40.8)	565 (446 to 711)	17.1 (13.4 to 21.6)	8.4 (-14.5 to 37.9)	13000 (10100 to 16600)	416.5 (320.9 to 535.8)	8.4 (-16.6 to 39.9)
Botswana	Both	298 (207 to 398)	21.7 (15.6 to 28.6)	-1.2 (-20.6 to 22.3)	298 (209 to 396)	22.6 (16.3 to 29.7)	-1.4 (-20.5 to 21.2)	8330 (5640 to 11300)	546.2 (378.3 to 727.6)	-3.9 (-24.3 to 21)
	Male	209 (146 to 270)	35.5 (25.4 to 44.7)	-5.7 (-23 to 15.6)	208 (146 to 266)	37.1 (26.7 to 46.6)	-5.9 (-22.6 to 14.5)	5970 (4080 to 7830)	885.8 (622.2 to 1132.8)	-8.2 (-27.2 to 14.7)
	Female	89 (56 to 134)	11.8 (7.6 to 17.4)	7.2 (-18.1 to 44.4)	90 (58 to 134)	12.4 (8.2 to 18.2)	6.1 (-19 to 41.6)	2360 (1460 to 3580)	285 (179.9 to 428.6)	5.5 (-21.5 to 46.5)
Brazil	Both	36400 (34200 to 38200)	15.4 (14.4 to 16.2)	-6.9 (-10.6 to -3.2)	37000 (34600 to 38900)	15.8 (14.7 to 16.6)	-7.2 (-10.5 to -3.5)	867000 (825000 to 907000)	359.1 (340.8 to 376)	-9.3 (-12.8 to -5.7)
	Male	21100 (19900 to 22300)	20.1 (18.9 to 21.3)	-12.9 (-17.4 to -8.5)	21600 (20300 to 22800)	20.9 (19.6 to 22.2)	-13 (-17.2 to -8.5)	503000 (476000 to 530000)	459.5 (434.4 to 484.1)	-15.4 (-19.4 to -10.9)
	Female	15300 (14100 to 16200)	11.7 (10.8 to 12.4)	3.6 (-2.1 to 9.3)	15400 (14100 to 16400)	11.8 (10.9 to 12.6)	3.1 (-2.5 to 8.9)	364000 (339000 to 387000)	277.5 (258.9 to 295.6)	1 (-4.6 to 6.7)
Brunei	Both	116 (102 to 130)	46.1 (40.9 to 51.8)	-0.3 (-12.6 to 12.9)	103 (92 to 116)	44.8 (39.7 to 50.2)	-2.3 (-14.1 to 10.8)	2580 (2270 to 2920)	867.7 (768.5 to 975.4)	-1.6 (-14 to 12.9)
	Male	63 (52 to 75)	61 (51.4 to 71.2)	1.1 (-16.1 to 21)	57 (47 to 67)	61.1 (52 to 71.1)	0.3 (-16.1 to 18)	1400 (1160 to 1670)	1086.9 (917.8 to 1259.1)	-2.7 (-18.7 to 15)

	Female	53 (44 to 63)	37.9 (32.3 to 44)	4.7 (-13.1 to 24.7)	47 (40 to 54)	36.1 (30.5 to 42)	2.4 (-13.4 to 20.1)	1180 (980 to 1390)	730.8 (618.7 to 850.6)	3.5 (-12.7 to 21.3)
Bulgaria	Both	4840 (3860 to 6020)	36.4 (28.7 to 45.5)	-3.8 (-25.1 to 22.8)	4610 (3700 to 5710)	34 (26.9 to 42.6)	-4.3 (-25.1 to 21.5)	117000 (91600 to 146000)	934.4 (730.8 to 1181.4)	-3.8 (-25.3 to 23.5)
	Male	3850 (3060 to 4820)	63.8 (50.3 to 79.9)	-7.7 (-28 to 18.4)	3630 (2890 to 4540)	59.7 (47.5 to 74.4)	-8.1 (-28 to 17.4)	93100 (73400 to 117000)	1595.5 (1249.6 to 2010.1)	-7.9 (-28.7 to 19.1)
	Female	986 (772 to 1240)	13.5 (10.5 to 17.1)	9.7 (-15.9 to 39.7)	978 (765 to 1230)	13 (10.1 to 16.4)	8.8 (-16 to 37.6)	23400 (18100 to 29700)	358.5 (274.7 to 456.6)	9.5 (-16.9 to 40.9)
Burkina Faso	Both	876 (683 to 1180)	10 (7.9 to 13.5)	15 (-3.2 to 38.4)	908 (711 to 1230)	10.8 (8.5 to 14.5)	13.8 (-3.6 to 36.7)	23500 (18100 to 31400)	244.1 (189.7 to 329.1)	15.9 (-3.9 to 41.6)
	Male	661 (497 to 920)	17 (12.8 to 23.6)	15.4 (-3.6 to 40.8)	688 (518 to 958)	18.4 (14 to 25.5)	14.2 (-3.4 to 38.9)	17600 (13000 to 24300)	409.4 (306.9 to 569.2)	15.7 (-4.9 to 43.3)
	Female	215 (154 to 295)	4.4 (3.2 to 6)	25.3 (5.2 to 51.5)	221 (159 to 301)	4.7 (3.4 to 6.4)	24.3 (4 to 49.7)	5910 (4140 to 8230)	108.1 (77.1 to 148.7)	27 (4.7 to 56.1)
Burundi	Both	312 (219 to 431)	7.1 (5 to 9.6)	-1.7 (-19.7 to 19.6)	322 (227 to 441)	7.6 (5.4 to 10.3)	-1.6 (-19.3 to 19.5)	8780 (6060 to 12300)	178.1 (124.7 to 243.8)	-2.1 (-21 to 20.1)
	Male	242 (169 to 334)	10.8 (7.7 to 14.6)	-6.2 (-23.7 to 15.4)	251 (177 to 347)	11.7 (8.4 to 15.8)	-6.2 (-23.3 to 15.8)	6720 (4610 to 9380)	266 (186.2 to 367.7)	-6.5 (-24.4 to 15.8)
	Female	70 (47 to 103)	3.2 (2.2 to 4.6)	4.6 (-15.5 to 29.1)	71 (48 to 104)	3.4 (2.3 to 4.9)	4.6 (-15.2 to 28.1)	2060 (1350 to 3020)	82.7 (55.1 to 120.7)	4.3 (-16.5 to 30.4)
Cabo Verde	Both	71 (56 to 86)	17.3 (13.5 to 21.1)	38.1 (6.1 to 71.8)	76 (59 to 92)	18.6 (14.4 to 22.8)	39.4 (5.8 to 73.9)	1690 (1360 to 2030)	394.2 (313.5 to 476.8)	31.4 (2.9 to 63.2)
	Male	45 (33 to 56)	26.4 (18.7 to 33.3)	39.7 (-1.7 to 80.5)	48 (35 to 60)	28.8 (20.3 to 36.4)	41.9 (-1.2 to 83)	1090 (831 to 1360)	594.5 (438.3 to 738.5)	33 (-3.1 to 71.5)
	Female	26 (21 to 32)	10.9 (8.7 to 13.3)	31.8 (1.5 to 68)	28 (22 to 34)	11.6 (9.2 to 14.2)	31.8 (-0.4 to 68.9)	592 (480 to 720)	244.7 (197.7 to 297.1)	25 (-1.8 to 57.5)
Cambodia	Both	2890 (2270 to 3580)	24.7 (19.4 to 30.6)	6.1 (-12.2 to 26.2)	2990 (2340 to 3700)	26.4 (20.8 to 32.6)	5.5 (-12.3 to 24.6)	76000 (58800 to 94300)	606.8 (473.9 to 750.7)	4.8 (-14.2 to 25.3)
	Male	2090 (1610 to 2670)	44.4 (34.4 to 57.8)	3.9 (-14.4 to 24.3)	2170 (1670 to 2790)	48 (37.2 to 62.6)	3.4 (-14.3 to 23.8)	54900 (42100 to 69400)	1062.4 (817.7 to 1358.8)	1.9 (-17 to 23)
	Female	796 (609 to 1020)	11.6 (9 to 14.8)	15.8 (-6.4 to 40.8)	816 (629 to 1050)	12.2 (9.5 to 15.4)	15.6 (-5.8 to 41.3)	21100 (16100 to 27200)	290.7 (222 to 372.6)	14 (-8.3 to 39.8)
Cameroon	Both	1560 (1120 to 2160)	13.7 (10 to 18.6)	1.5 (-19.5 to 25.4)	1610 (1160 to 2200)	14.7 (10.8 to 19.8)	1.4 (-18.2 to 24.8)	42200 (29900 to 59100)	332.6 (238.7 to 458.3)	0 (-21.5 to 25.1)
	Male	1110 (792 to 1510)	20.5 (15 to 27.5)	-3 (-22.4 to 21.7)	1140 (820 to 1540)	22.2 (16.2 to 29.7)	-3 (-21.8 to 20.1)	29700 (21100 to 41000)	490.2 (351.8 to 668.8)	-4.5 (-25.1 to 21.1)
	Female	456 (310 to 657)	7.6 (5.3 to 10.7)	14.9 (-9.6 to 43.5)	468 (324 to 665)	8.2 (5.8 to 11.3)	14.6 (-9.5 to 43.1)	12600 (8300 to 18500)	185.3 (126.2 to 268.1)	13.7 (-11.8 to 44.3)
Canada	Both	30300 (23600 to 38200)	43.6 (34 to 55)	-9.6 (-30 to 14)	24100 (22000 to 25600)	34.1 (31.4 to 36.1)	-10.4 (-14.9 to -5.7)	479000 (448000 to 506000)	716.6 (673 to 755.8)	-11.8 (-16.6 to -7.1)

	Male	15900 (12100 to 20300)	48.8 (37.4 to 62.3)	-12.8 (-33.1 to 11.8)	13000 (12000 to 14000)	40.1 (36.8 to 42.9)	-13.3 (-18.8 to -6.8)	258000 (240000 to 275000)	807 (750.9 to 860.9)	-14.6 (-19.9 to -8.8)
	Female	14400 (11200 to 18100)	39.5 (30.8 to 49.9)	-6.9 (-26.4 to 18.1)	11000 (9830 to 12100)	29.3 (26.4 to 31.8)	-8 (-16 to -0.5)	222000 (200000 to 240000)	639.7 (581.8 to 690.3)	-9.1 (-17 to -1.7)
Central Africa Republic	Both	284 (144 to 498)	12.4 (6.9 to 20.9)	-8.7 (-25.7 to 11.3)	289 (147 to 504)	13.1 (7.5 to 21.9)	-8.7 (-25.7 to 10.7)	8830 (4300 to 15700)	343.9 (178 to 596.5)	-9.2 (-27.1 to 12.6)
	Male	236 (105 to 440)	23 (11.6 to 40.8)	-10 (-26.2 to 9.7)	240 (108 to 444)	24.6 (12.6 to 43.2)	-10 (-26.3 to 9.5)	7400 (3140 to 14000)	627.4 (294.7 to 1146.1)	-10.7 (-27.1 to 9.8)
	Female	49 (34 to 68)	4.1 (2.8 to 5.8)	-1.6 (-25.6 to 27.8)	49 (34 to 68)	4.4 (3 to 6.1)	-1.4 (-25.1 to 27.9)	1430 (976 to 2030)	108.1 (74.4 to 150.7)	-2.4 (-26.6 to 27.7)
Chad	Both	625 (447 to 888)	11.7 (8.5 to 16.5)	4.2 (-14.6 to 27.3)	655 (470 to 926)	12.7 (9.2 to 17.9)	4 (-14.4 to 27.3)	16600 (11700 to 23800)	284.4 (202.9 to 406.3)	2.5 (-16.7 to 26.7)
	Male	507 (355 to 758)	17.6 (12.4 to 26.2)	-0.6 (-18.5 to 23.4)	534 (373 to 800)	19.2 (13.6 to 28.4)	-0.9 (-18.5 to 23)	13300 (9230 to 19800)	425.6 (297.3 to 634.6)	-1.9 (-20.8 to 22.5)
	Female	117 (82 to 174)	4.7 (3.3 to 6.9)	15.1 (-5.2 to 44.2)	120 (85 to 178)	5 (3.5 to 7.4)	15.1 (-5.1 to 43.4)	3270 (2280 to 4910)	114.7 (80.4 to 169.9)	14.1 (-7 to 43.7)
Chile	Both	4050 (3180 to 5100)	16.8 (13.2 to 21.1)	-3.8 (-24.4 to 20.9)	4010 (3700 to 4270)	16.6 (15.3 to 17.7)	-5.1 (-11.3 to 0.8)	83700 (78000 to 88900)	347.2 (323.8 to 368.9)	-6.8 (-13.1 to -0.5)
	Male	2360 (1830 to 3020)	21.8 (16.9 to 27.8)	-9.7 (-29.7 to 15)	2330 (2150 to 2500)	21.7 (20.1 to 23.3)	-10.8 (-17.4 to -3.8)	49800 (46200 to 53500)	452.5 (419.3 to 486.1)	-12.2 (-18.9 to -4.8)
	Female	1700 (1340 to 2120)	12.7 (10 to 15.8)	5.7 (-17.2 to 32.1)	1680 (1510 to 1830)	12.4 (11.2 to 13.6)	4.1 (-4.4 to 13.5)	33800 (30800 to 36600)	259 (236.4 to 280)	2.8 (-6.3 to 12.6)
China	Both	833000 (700000 to 982000)	41.7 (35.2 to 48.8)	0.5 (-17.1 to 20)	757000 (639000 to 888000)	38.7 (32.8 to 45)	-4 (-19.7 to 14.5)	17100000 (14300000 to 20200000)	831.3 (699.1 to 980)	-5.4 (-21.7 to 14.2)
	Male	576000 (451000 to 709000)	61.7 (48.9 to 75.2)	-0.9 (-21.7 to 24.7)	523000 (413000 to 647000)	58.1 (46.5 to 70.9)	-5.4 (-25.3 to 17.9)	12000000 (9370000 to 15000000)	1203.8 (950.2 to 1495.1)	-6.4 (-27.3 to 18.9)
	Female	257000 (206000 to 314000)	24.8 (19.9 to 30.3)	4.3 (-16.6 to 30.4)	234000 (189000 to 283000)	22.9 (18.5 to 27.5)	-0.4 (-20.8 to 23.3)	5160000 (4140000 to 6350000)	492.2 (393.7 to 604.3)	-1.7 (-23.3 to 22.9)
Colombia	Both	6400 (5020 to 8150)	12.1 (9.5 to 15.5)	-6.1 (-26.7 to 20.1)	6350 (4980 to 8060)	12 (9.4 to 15.3)	-8.7 (-28.4 to 16.6)	136000 (106000 to 176000)	259.1 (201.9 to 333.9)	-9.3 (-30.1 to 17.5)
	Male	3340 (2550 to 4330)	14 (10.7 to 18.2)	-15.3 (-35.9 to 10.6)	3430 (2630 to 4450)	14.5 (11 to 18.7)	-16.3 (-36.4 to 8.6)	73600 (55800 to 96800)	307.1 (233.4 to 403.3)	-17.9 (-38.1 to 8.5)
	Female	3070 (2400 to 3870)	10.6 (8.3 to 13.4)	7.7 (-15.2 to 35.5)	2920 (2300 to 3680)	10 (7.9 to 12.6)	3.5 (-18.2 to 29.6)	62600 (49200 to 80300)	219.4 (172.4 to 281.1)	4.6 (-18.5 to 33.1)
Comoros	Both	35 (26 to 47)	7.4 (5.5 to 9.8)	5 (-14.3 to 26.6)	37 (28 to 49)	7.9 (6 to 10.4)	4.2 (-14.8 to 25.6)	921 (662 to 1260)	182.7 (132.5 to 245.9)	6.5 (-14.5 to 29.7)

	Male	23 (16 to 32)	10.7 (7.8 to 15)	1.5 (-17.8 to 24.4)	24 (18 to 34)	11.6 (8.6 to 16.2)	0.9 (-17.7 to 22.9)	586 (406 to 855)	256.8 (182.8 to 369.4)	2.6 (-18.5 to 25.8)
	Female	13 (9 to 16)	4.8 (3.4 to 6.1)	13 (-10.8 to 39.2)	13 (9 to 17)	5 (3.6 to 6.4)	12.5 (-10.2 to 37.4)	335 (233 to 442)	122 (85.3 to 160)	14.5 (-11.5 to 43.6)
Congo	Both	416 (292 to 595)	16 (11.8 to 22.5)	5.9 (-14.4 to 29.9)	422 (301 to 602)	17 (12.8 to 23.6)	6.1 (-13.7 to 29.2)	11900 (8070 to 17400)	406.9 (289 to 583.8)	4.1 (-17.2 to 30.9)
	Male	287 (208 to 410)	23.3 (17.8 to 32.7)	0.1 (-18 to 23.2)	292 (214 to 415)	24.9 (19.2 to 34.6)	0.1 (-17.9 to 21.8)	8190 (5750 to 11900)	582.5 (428.9 to 829.8)	-1.1 (-20.3 to 24.3)
	Female	129 (79 to 198)	9.6 (6.2 to 14.2)	12.5 (-14.8 to 43)	131 (81 to 198)	10.1 (6.7 to 14.8)	12.8 (-13.5 to 42.8)	3690 (2200 to 5780)	244.1 (150.2 to 372.9)	10.3 (-16.6 to 42.9)
Cook Islands	Both	7 (6 to 9)	29.1 (24.9 to 34.3)	-1.3 (-14.9 to 14.5)	7 (6 to 9)	29.8 (25.5 to 34.9)	-2.4 (-15.2 to 12.8)	165 (140 to 197)	668 (562.5 to 798)	-1.8 (-16.5 to 14.6)
	Male	6 (5 to 7)	48.5 (41.5 to 57)	-1.7 (-15.8 to 15.7)	6 (5 to 7)	49.9 (43 to 58.4)	-2.9 (-16.2 to 14.4)	135 (114 to 160)	1115.4 (935.8 to 1331)	-1.9 (-17.3 to 16)
	Female	1 (1 to 2)	10.8 (8.5 to 13.6)	7.2 (-13.6 to 33.7)	1 (1 to 2)	11.1 (8.8 to 13.8)	6.3 (-14 to 32.9)	30 (24 to 39)	240.5 (187.6 to 307.3)	5.6 (-15.9 to 32.7)
Costa Rica	Both	503 (390 to 634)	9.9 (7.7 to 12.4)	-5 (-25.8 to 20.1)	492 (384 to 616)	9.7 (7.6 to 12.1)	-6.8 (-26.9 to 17)	10500 (8140 to 13500)	204.9 (158.1 to 261.6)	-7.2 (-28.9 to 19)
	Male	314 (241 to 401)	13.5 (10.4 to 17.3)	-8.2 (-29.1 to 18.6)	317 (247 to 405)	13.8 (10.7 to 17.6)	-9.4 (-30.1 to 16.4)	6830 (5190 to 8770)	286.5 (218.4 to 366.4)	-9.6 (-31 to 17.1)
	Female	189 (146 to 239)	6.9 (5.3 to 8.7)	3.1 (-19.1 to 30.7)	174 (134 to 219)	6.3 (4.9 to 8)	0.8 (-21 to 26.3)	3720 (2860 to 4740)	134.7 (103.7 to 171.8)	0 (-22.6 to 27.5)
Croatia	Both	3430 (2710 to 4300)	40.4 (31.6 to 51)	-13.2 (-31.7 to 10.6)	2880 (2280 to 3610)	33.4 (26.3 to 42.1)	-15.5 (-33.3 to 7.7)	65000 (50900 to 82400)	811.1 (631.8 to 1038.1)	-17.2 (-35.7 to 6.6)
	Male	2520 (1980 to 3160)	67.3 (52.8 to 84.2)	-17.1 (-35.3 to 4.4)	2150 (1690 to 2680)	57 (45 to 71.2)	-19.2 (-37 to 1.9)	49000 (38000 to 61900)	1332.5 (1030.5 to 1683.4)	-20.6 (-38.9 to 1.8)
	Female	909 (711 to 1160)	19.4 (15 to 24.7)	-6.1 (-27.8 to 21.8)	729 (573 to 929)	15.1 (11.8 to 19.4)	-9.3 (-29.9 to 18)	15900 (12300 to 20500)	374.2 (285.8 to 485.9)	-10.1 (-31.5 to 18.2)
Cuba	Both	6830 (5600 to 8300)	35.9 (29.4 to 43.7)	1 (-17.1 to 21.9)	6560 (5400 to 7940)	34.2 (28.1 to 41.5)	-0.8 (-18.3 to 19.4)	143000 (115000 to 175000)	764.9 (617.3 to 936.6)	-2.4 (-20.8 to 19.1)
	Male	4360 (3560 to 5340)	48.8 (39.9 to 59.7)	0.3 (-17.7 to 22)	4160 (3410 to 5110)	46.5 (38.1 to 57)	-1.8 (-19 to 18.9)	90200 (72600 to 111000)	1016.4 (818.4 to 1250.3)	-2.9 (-21.5 to 18.6)
	Female	2460 (2000 to 2990)	24.6 (19.9 to 29.9)	3.5 (-16.2 to 25.3)	2400 (1950 to 2900)	23.5 (19.1 to 28.5)	2 (-16.9 to 22.9)	52400 (42100 to 64200)	540 (434.8 to 662.1)	-0.5 (-20 to 21.3)
Cyprus	Both	514 (444 to 592)	26 (22.6 to 30)	-7.3 (-20.9 to 7.6)	462 (404 to 525)	23.4 (20.5 to 26.6)	-8.4 (-20.5 to 4.7)	9680 (8510 to 10900)	496.5 (437.3 to 562.2)	-8.7 (-20.2 to 3.1)
	Male	398 (330 to 469)	42.3 (35.1 to 49.9)	-12.2 (-27.6 to 5.5)	358 (309 to 412)	38.4 (33.1 to 44.4)	-14.4 (-27 to 0.1)	7500 (6530 to 8560)	796.9 (695.1 to 911.4)	-12 (-23.7 to 1.5)
	Female	116 (95 to 138)	11.4 (9.4 to 13.6)	-1.9 (-19.8 to 17.1)	104 (88 to 121)	10.2 (8.7 to 11.8)	-1.4 (-17.1 to 13.5)	2180 (1840 to 2520)	219.2 (186.6 to 253.9)	-3.7 (-18.9 to 11.6)

Czechia	Both	6940 (5700 to 8450)	32.9 (26.9 to 40.1)	-16.5 (-31.9 to 2.5)	6240 (5140 to 7580)	29.2 (24 to 35.5)	-17.3 (-32.3 to 1.2)	134000 (109000 to 164000)	664.4 (539.7 to 817.7)	-20 (-35.6 to -1.6)
	Male	4690 (3810 to 5780)	49.8 (40.5 to 61.1)	-22 (-36.6 to -4.2)	4240 (3480 to 5180)	45 (37 to 54.9)	-22.6 (-36.6 to -4.9)	91900 (74900 to 113000)	990 (804.7 to 1219)	-25.2 (-39.7 to -7.5)
	Female	2250 (1810 to 2760)	19.5 (15.7 to 24)	-5.9 (-24 to 16.1)	2000 (1620 to 2430)	16.8 (13.6 to 20.6)	-7 (-24.7 to 14.5)	41600 (33400 to 51000)	389.6 (312.9 to 482.5)	-8.7 (-26.7 to 13.6)
Côte d'Ivoire	Both	1280 (940 to 1650)	12.8 (9.7 to 16.3)	1.1 (-18.8 to 25.8)	1310 (965 to 1690)	13.8 (10.5 to 17.5)	1.2 (-18.7 to 24.8)	35100 (25300 to 46300)	309.3 (228 to 396.9)	0.3 (-20.8 to 27.3)
	Male	986 (711 to 1290)	19.3 (14.2 to 24.8)	-3.1 (-22.2 to 22.5)	1010 (736 to 1320)	21 (15.6 to 26.8)	-2.9 (-21.3 to 21.5)	26900 (19200 to 35300)	459.1 (334 to 597)	-4 (-24.2 to 23.4)
	Female	293 (221 to 377)	6.1 (4.7 to 7.6)	14.9 (-9.9 to 44.5)	300 (231 to 383)	6.5 (5.1 to 8.2)	14.7 (-9.4 to 42.7)	8230 (6000 to 10800)	146.2 (111.2 to 187.4)	14.6 (-11.9 to 48.2)
North Korea	Both	9040 (7010 to 11500)	27.9 (21.8 to 35.2)	-2.1 (-16.6 to 14.4)	9160 (7160 to 11500)	28.5 (22.5 to 35.8)	-2.4 (-16.2 to 13.7)	235000 (178000 to 306000)	712.3 (539.3 to 920.2)	-3.1 (-18 to 14.9)
	Male	6060 (4410 to 8120)	45.7 (33.8 to 59.9)	-7 (-21.2 to 9.6)	6070 (4440 to 8080)	47.5 (35.3 to 61.5)	-7.4 (-20.8 to 9)	162000 (114000 to 221000)	1126.9 (815.7 to 1514.4)	-7.7 (-22.2 to 9.7)
	Female	2980 (2140 to 3880)	15.9 (11.4 to 20.7)	1.5 (-18.7 to 24.3)	3090 (2240 to 3990)	16.3 (11.8 to 21.1)	0.8 (-19 to 21.8)	73700 (52200 to 98900)	403.1 (281.9 to 545.1)	0.9 (-20.4 to 24.6)
Democratic Republic of the Congo	Both	4250 (2250 to 8570)	11.9 (6.3 to 23.3)	8.5 (-11.2 to 38.2)	4310 (2290 to 8650)	12.5 (6.6 to 24.4)	8.4 (-11.2 to 38.5)	122000 (65300 to 247000)	306.9 (162.8 to 617)	7.8 (-12.7 to 39.9)
	Male	3280 (1580 to 7550)	20.7 (10 to 46.7)	0.7 (-17.3 to 29.6)	3320 (1600 to 7590)	22.1 (10.6 to 49.1)	0.7 (-17.4 to 28.7)	95000 (45300 to 221000)	524.4 (253.5 to 1192.4)	0.1 (-18.3 to 31.8)
	Female	974 (609 to 1480)	5.1 (3.1 to 7.8)	28.9 (-1.5 to 73.1)	995 (619 to 1520)	5.4 (3.2 to 8.4)	28.7 (-2 to 72.8)	27000 (17000 to 40500)	128.1 (80.2 to 194.8)	28.4 (-2.7 to 72.8)
Denmark	Both	4930 (3890 to 6080)	42.9 (33.7 to 53.1)	-19 (-36.6 to 1.3)	4360 (4010 to 4720)	37 (34.1 to 40)	-15.9 (-21.8 to -10.1)	85700 (79500 to 92700)	786.6 (730.4 to 848.6)	-17.3 (-23 to -11.6)
	Male	2540 (1980 to 3180)	46.6 (36.3 to 58.7)	-23.4 (-40.6 to -3.4)	2280 (2090 to 2460)	41.5 (38.2 to 44.7)	-20.3 (-26.5 to -14.3)	44500 (41200 to 48200)	845.5 (781.3 to 913)	-21.6 (-27.8 to -15.4)
	Female	2390 (1870 to 3000)	40.1 (31.1 to 50.2)	-14.7 (-33.7 to 7.2)	2080 (1850 to 2330)	33.5 (30 to 37.2)	-11.9 (-20.1 to -3)	41200 (36900 to 45700)	739.1 (664.7 to 813.9)	-12.9 (-21.1 to -4.4)
Djibouti	Both	62 (39 to 104)	10.8 (7.1 to 17.4)	6.1 (-15.6 to 31.3)	63 (40 to 106)	11.6 (7.7 to 18.5)	6.1 (-14.9 to 31.2)	1740 (1060 to 2960)	266.5 (170.5 to 444.2)	4.5 (-18 to 30.2)
	Male	47 (28 to 86)	15.7 (9.6 to 27.2)	1.5 (-20.4 to 26.1)	49 (29 to 88)	17.1 (10.6 to 29.5)	1.3 (-19.4 to 25)	1320 (760 to 2440)	379.3 (227 to 675.2)	0.3 (-21.6 to 25.7)
	Female	14 (10 to 20)	5.2 (3.8 to 7.2)	15.7 (-9.7 to 47.4)	15 (10 to 20)	5.6 (4.2 to 7.6)	15.6 (-8.6 to 47.2)	419 (286 to 605)	133.4 (95.4 to 187.8)	14.2 (-12.8 to 47.5)
Dominica	Both	15 (12 to 18)	16.9 (13.6 to 20.5)	2.5 (-15.2 to 24)	16 (13 to 19)	17.5 (14.2 to 21.1)	2.8 (-14.7 to 23.7)	357 (286 to 439)	402.2 (322.1 to 495.9)	2.2 (-15.4 to 24.7)

	Male	11 (9 to 13)	24.4 (19.6 to 29.7)	-1.1 (-20.7 to 22)	11 (9 to 13)	25.4 (20.4 to 30.9)	-1.4 (-20.1 to 21.2)	254 (200 to 316)	568.7 (450.5 to 702.7)	-0.5 (-20.5 to 24.4)
	Female	5 (4 to 6)	9.9 (7.8 to 12.2)	5.9 (-14.3 to 30)	5 (4 to 6)	10.3 (8.2 to 12.8)	6.1 (-14 to 29.8)	103 (81 to 128)	235.4 (184.5 to 294)	6 (-14.9 to 31.6)
Dominican Republic	Both	1410 (1020 to 1900)	15.2 (11.1 to 20.5)	12.2 (-20.7 to 58.1)	1440 (1050 to 1930)	15.8 (11.6 to 21.1)	10.9 (-20.6 to 55.2)	35100 (24700 to 48000)	367.2 (260.2 to 501.2)	14.4 (-20.8 to 62.6)
	Male	917 (656 to 1250)	20.8 (15.1 to 28.1)	15 (-19.6 to 63.3)	931 (675 to 1270)	21.5 (15.8 to 28.9)	13.4 (-19.7 to 59.1)	22900 (15900 to 31300)	495 (347.3 to 677.7)	18.3 (-19.6 to 69.1)
	Female	487 (331 to 670)	10.2 (6.9 to 14)	8.3 (-24.9 to 53.1)	506 (347 to 689)	10.7 (7.3 to 14.5)	7.5 (-24.6 to 51.1)	12200 (8100 to 17000)	247.2 (165 to 346)	8.7 (-26 to 54.5)
Ecuador	Both	1480 (1170 to 1880)	10.1 (8 to 12.8)	-4.2 (-25 to 21.8)	1550 (1230 to 1970)	10.8 (8.6 to 13.6)	-5.6 (-25.4 to 19.3)	34000 (26500 to 43700)	221.8 (174 to 284.6)	-6 (-27.7 to 20.8)
	Male	820 (635 to 1050)	11.8 (9.2 to 15.2)	-9.1 (-30.4 to 19.2)	867 (677 to 1110)	12.8 (10.1 to 16.5)	-10.5 (-31 to 16.6)	18700 (14300 to 24400)	256.2 (196.7 to 332.8)	-10.6 (-32.7 to 18.7)
	Female	658 (521 to 831)	8.5 (6.7 to 10.7)	3.3 (-19.5 to 31)	685 (542 to 864)	9 (7.1 to 11.3)	2.1 (-19.7 to 28.6)	15300 (11900 to 19400)	190.7 (150.4 to 242.4)	0.8 (-22.4 to 29.3)
Egypt	Both	6120 (4300 to 8310)	9.2 (6.5 to 12.5)	5.1 (-20 to 35.6)	6070 (4270 to 8220)	9.5 (6.7 to 12.9)	4.6 (-19.7 to 34.5)	175000 (123000 to 239000)	240.9 (169.4 to 326.9)	4.4 (-21.3 to 35.8)
	Male	4210 (2910 to 5910)	11.5 (8 to 16.1)	-1.7 (-26.7 to 28.7)	4210 (2910 to 5890)	11.8 (8.3 to 16.7)	-1.9 (-26.7 to 27.9)	122000 (83900 to 172000)	310.4 (213.2 to 437.3)	-1.7 (-26.9 to 29.3)
	Female	1920 (1220 to 2770)	6.6 (4.2 to 9.4)	22.2 (-10.4 to 63.3)	1870 (1180 to 2680)	6.8 (4.3 to 9.7)	20.9 (-11 to 60.8)	53500 (34600 to 76700)	162.4 (103.4 to 233.2)	20.3 (-12.5 to 62.2)
El Salvador	Both	619 (469 to 797)	10.4 (7.9 to 13.5)	9.9 (-17.9 to 42)	632 (478 to 810)	10.6 (8 to 13.6)	8.6 (-18.5 to 39.4)	14100 (10500 to 18200)	240.2 (179.2 to 311.3)	7.4 (-20.1 to 40.1)
	Male	306 (230 to 395)	12.2 (9.1 to 15.8)	7.8 (-20.9 to 40.6)	321 (243 to 413)	12.7 (9.6 to 16.4)	6.9 (-21.4 to 38.4)	7200 (5350 to 9420)	288.1 (213.4 to 377.2)	6.9 (-22.3 to 41.4)
	Female	313 (231 to 406)	9.2 (6.7 to 11.9)	12.9 (-18 to 47.6)	312 (230 to 403)	9 (6.6 to 11.6)	11.4 (-18.6 to 45.3)	6880 (5080 to 9040)	204.5 (150.5 to 268.8)	8.8 (-21.1 to 42.8)
Equatorial Guinea	Both	76 (49 to 117)	16.4 (10.8 to 24.6)	9.9 (-15.3 to 41.1)	78 (50 to 119)	17.5 (11.7 to 25.9)	10.1 (-14.2 to 40.3)	2100 (1280 to 3280)	401 (255.8 to 618.3)	8.6 (-18 to 43.2)
	Male	48 (29 to 74)	24.8 (15.6 to 37.1)	4.5 (-16.6 to 30.1)	49 (30 to 75)	26.6 (16.8 to 39.5)	4.4 (-16.1 to 29.1)	1330 (779 to 2100)	603.8 (370.9 to 928.5)	4 (-18.8 to 33.2)
	Female	28 (17 to 46)	10.6 (6.5 to 16.5)	18.7 (-18.4 to 69.8)	29 (17 to 46)	11.3 (7 to 17.2)	19.1 (-17.3 to 68.6)	773 (438 to 1280)	256.7 (150.2 to 412)	16.1 (-21.8 to 70.7)
Eritrea	Both	226 (173 to 293)	8.4 (6.5 to 10.6)	3.8 (-15 to 25.9)	228 (175 to 293)	8.9 (6.9 to 11.1)	3.8 (-14.4 to 25.4)	6680 (5000 to 8890)	218.5 (167.8 to 280.2)	1.9 (-17.4 to 24.3)
	Male	151 (112 to 198)	13.9 (10.6 to 17.8)	-3.9 (-20.3 to 18.8)	152 (112 to 198)	14.8 (11.2 to 18.8)	-3.8 (-20.3 to 17.6)	4480 (3250 to 5980)	347.2 (259.1 to 448)	-5.7 (-23.6 to 17.5)
	Female	75 (52 to 106)	4.9 (3.5 to 6.8)	16.1 (-9.2 to 45.9)	76 (53 to 106)	5.2 (3.7 to 7.2)	15.9 (-8.3 to 45.8)	2200 (1510 to 3110)	128.1 (89.3 to 179.8)	14.9 (-10.1 to 46)

Estonia	Both	721 (564 to 906)	28.3 (22 to 35.6)	-9.8 (-29.7 to 13.6)	714 (561 to 896)	27.3 (21.3 to 34.5)	-10.6 (-30 to 12.3)	15100 (11700 to 19200)	634.3 (492 to 807.1)	-11.7 (-31.9 to 11.8)
	Male	505 (396 to 644)	51.1 (40 to 65.2)	-19.8 (-37.5 to 0.3)	505 (398 to 639)	51 (40.2 to 64.5)	-20.1 (-37.5 to 0)	11100 (8530 to 14100)	1131.8 (872.9 to 1445.4)	-20.5 (-38.7 to 0.7)
	Female	216 (167 to 273)	13.5 (10.4 to 17.2)	12.1 (-14 to 43.9)	209 (162 to 263)	12.5 (9.6 to 16)	9.3 (-15.7 to 39.6)	4050 (3120 to 5200)	285.6 (217.9 to 370.4)	9.7 (-16.5 to 41.3)
eSwatini	Both	104 (63 to 147)	17.8 (11.1 to 24.9)	-6.9 (-27 to 23)	105 (65 to 148)	18.7 (11.7 to 26.1)	-7.3 (-27.1 to 21.8)	2950 (1750 to 4310)	465.7 (280.4 to 666.3)	-9.6 (-29.9 to 22.3)
	Male	74 (43 to 104)	31.3 (18.9 to 43.3)	-7.3 (-25.9 to 23.4)	74 (44 to 104)	33 (20.1 to 45.3)	-7.8 (-26.1 to 21)	2180 (1240 to 3130)	819.5 (483.8 to 1151.8)	-10.3 (-29.3 to 22.9)
	Female	30 (18 to 48)	9 (5.4 to 14)	-0.5 (-30.4 to 44.5)	31 (18 to 49)	9.7 (6 to 14.9)	-0.4 (-29.4 to 43.5)	775 (445 to 1250)	216.9 (126.5 to 346.8)	-3.4 (-33.9 to 44.7)
Ethiopia	Both	2170 (1510 to 2920)	5.6 (3.9 to 7.5)	3.6 (-15.6 to 28.6)	2310 (1600 to 3130)	6.1 (4.2 to 8.3)	3.6 (-16.9 to 29.7)	55500 (38400 to 75100)	131.8 (91 to 178.9)	2.8 (-16.9 to 27.3)
	Male	1690 (1160 to 2280)	8.6 (5.9 to 11.6)	2.4 (-20.1 to 32.7)	1820 (1240 to 2490)	9.5 (6.5 to 12.9)	2.6 (-22 to 33.5)	42300 (29000 to 58000)	199.5 (136.2 to 272.2)	1.2 (-23.1 to 33.5)
	Female	476 (290 to 764)	2.4 (1.5 to 3.8)	23.4 (0.1 to 55.3)	488 (300 to 796)	2.5 (1.6 to 4.1)	23.7 (2 to 54.4)	13200 (8030 to 21500)	59.7 (36.6 to 98.1)	21.9 (-0.5 to 53.6)
Fiji	Both	73 (57 to 92)	10.1 (8 to 12.5)	6.8 (-14.5 to 33)	74 (59 to 93)	10.7 (8.6 to 13.2)	6.8 (-14.3 to 32.2)	1950 (1510 to 2480)	246 (192.5 to 308.2)	6.2 (-16.2 to 33.3)
	Male	47 (36 to 60)	14.4 (11.5 to 17.8)	5.7 (-15.8 to 31.9)	48 (37 to 61)	15.7 (12.6 to 19.2)	5.6 (-15.6 to 30.6)	1260 (965 to 1610)	339.4 (264.9 to 429.3)	5.1 (-17.5 to 33.5)
	Female	26 (20 to 34)	6.8 (5.2 to 8.6)	10.2 (-17 to 40.3)	27 (20 to 34)	7.2 (5.5 to 9)	10 (-16.7 to 39.7)	693 (530 to 902)	167.6 (128.2 to 215.8)	9.4 (-18.8 to 42.1)
Finland	Both	3110 (2470 to 3910)	25.2 (19.8 to 31.7)	-7.5 (-27.6 to 16.5)	2590 (2380 to 2780)	20.5 (18.9 to 21.9)	-8.1 (-14.2 to -1.8)	49400 (45600 to 52900)	430 (396.7 to 460.1)	-10.8 (-16.9 to -4.4)
	Male	2010 (1560 to 2530)	35.8 (27.7 to 45.2)	-14.9 (-34.2 to 7.6)	1700 (1560 to 1840)	30.1 (27.7 to 32.5)	-15.7 (-22.4 to -9.1)	32700 (30100 to 35200)	605.7 (558.4 to 655.1)	-17.2 (-23.5 to -10.5)
	Female	1100 (872 to 1420)	16.6 (13 to 21.3)	3.5 (-19.4 to 33.1)	891 (791 to 984)	12.9 (11.5 to 14.1)	3.3 (-5.7 to 13.5)	16800 (15100 to 18400)	279.9 (252.5 to 307.3)	0.1 (-8.8 to 10.1)
France	Both	45100 (35100 to 57500)	36.8 (28.6 to 47)	-8.8 (-29.6 to 16.7)	40200 (37100 to 42800)	31.5 (29.4 to 33.4)	-9.7 (-14.9 to -4.7)	880000 (823000 to 934000)	780.6 (731.1 to 828.8)	-11.9 (-17.6 to -6.6)
	Male	32200 (25000 to 41000)	57.1 (44.1 to 72.9)	-13.3 (-33.4 to 11.9)	28900 (26800 to 31000)	50.2 (46.8 to 53.7)	-14 (-19.9 to -7.9)	639000 (594000 to 683000)	1195.2 (1110.8 to 1276)	-15.7 (-22.3 to -9.5)
	Female	12900 (10100 to 16400)	19.7 (15.4 to 25.3)	2.2 (-20.5 to 29.8)	11300 (9900 to 12500)	16.1 (14.6 to 17.6)	0.8 (-6.8 to 9.2)	242000 (220000 to 264000)	415.6 (378.1 to 453.4)	-1.8 (-9.7 to 6.9)
Gabon	Both	202 (135 to 289)	19.3 (13.1 to 26.9)	0.8 (-17 to 24.5)	205 (138 to 292)	20.2 (13.9 to 28.1)	1.1 (-16.4 to 23.9)	5520 (3620 to 8040)	485.7 (322.9 to 695.2)	-1.6 (-20.6 to 23.2)
	Male	148 (99 to 218)	30.2 (21 to 43.2)	-3.7 (-20.7 to 19.1)	149 (101 to 218)	32 (22.4 to 45.4)	-3.7 (-20.2 to 18.4)	4110 (2690 to 6100)	754.8 (511 to 1102.9)	-5.2 (-22.9 to 19.6)

	Female	54 (34 to 77)	9.9 (6.4 to 13.9)	10.1 (-15.1 to 41.1)	56 (36 to 79)	10.5 (6.8 to 14.7)	10.4 (-15.2 to 40.1)	1410 (874 to 2100)	241.3 (150.9 to 350.6)	7.7 (-18.7 to 41.4)
The Gambia	Both	69 (52 to 89)	7.5 (5.7 to 9.6)	9 (-11.1 to 33.1)	72 (55 to 93)	8.1 (6.3 to 10.3)	8.7 (-10.6 to 32.1)	1790 (1340 to 2330)	182 (137 to 236.9)	8.9 (-12.9 to 34.7)
	Male	45 (35 to 58)	10.3 (8.1 to 13.2)	3 (-18.5 to 27.6)	48 (37 to 61)	11.2 (8.9 to 14.3)	2.6 (-17.7 to 25.7)	1170 (878 to 1500)	247.2 (189 to 315.3)	3 (-19.3 to 28.9)
	Female	24 (15 to 34)	4.9 (3 to 7)	24.7 (-6.3 to 61.8)	25 (15 to 35)	5.3 (3.3 to 7.5)	24 (-7.1 to 60.1)	617 (384 to 869)	119.9 (74.5 to 170.8)	25.2 (-6.8 to 65.8)
Georgia	Both	1780 (1490 to 2110)	31.1 (25.9 to 36.9)	6.3 (-13.3 to 28.5)	1770 (1490 to 2080)	30.6 (25.6 to 36)	6 (-13.2 to 27.6)	45700 (37900 to 54200)	828.6 (684 to 980.4)	3.9 (-16 to 26.4)
	Male	1520 (1250 to 1800)	62.1 (51.5 to 73.5)	6.9 (-12.8 to 30)	1500 (1240 to 1760)	61.5 (51.3 to 72.5)	6.7 (-12.7 to 29)	39400 (32600 to 46900)	1623.5 (1343.6 to 1925.2)	4.3 (-15.9 to 27.8)
	Female	269 (224 to 322)	8 (6.7 to 9.7)	2.9 (-17.7 to 25.5)	274 (230 to 329)	8 (6.6 to 9.6)	2.8 (-17.2 to 25)	6390 (5260 to 7670)	207.2 (170.3 to 250.1)	-0.6 (-20.3 to 23.1)
Germany	Both	63500 (49700 to 81900)	35.5 (27.8 to 46.1)	-8.9 (-29.1 to 17.5)	54000 (50500 to 57300)	29 (27.3 to 30.6)	-9.2 (-13.8 to -4.3)	1110000 (1050000 to 1180000)	668.6 (631.4 to 705.6)	-10.4 (-15.3 to -5.5)
	Male	40700 (31200 to 52600)	48.5 (37.4 to 62.9)	-15.7 (-35 to 9.2)	35300 (32800 to 37600)	41.3 (38.4 to 44)	-15.7 (-20.8 to -10.1)	726000 (680000 to 773000)	909 (852.1 to 967.2)	-16 (-21.2 to -10.2)
	Female	22800 (17700 to 29300)	24.6 (18.9 to 31.9)	1.7 (-21.6 to 30.9)	18700 (16800 to 20300)	18.9 (17.3 to 20.4)	0.7 (-7.2 to 8.6)	388000 (356000 to 418000)	455.9 (418.7 to 492.5)	-0.7 (-8.5 to 7.5)
Ghana	Both	1380 (1110 to 1730)	8.7 (7.1 to 10.8)	6.8 (-11.5 to 29)	1410 (1140 to 1760)	9.3 (7.6 to 11.5)	6.2 (-11.3 to 27.7)	37900 (30000 to 47600)	215.6 (173.5 to 270.4)	6.1 (-12.8 to 30.8)
	Male	937 (751 to 1170)	13.9 (11.3 to 17.1)	5.3 (-15.6 to 31.7)	965 (779 to 1200)	15 (12.2 to 18.3)	4.7 (-15.4 to 30.2)	25200 (19900 to 31800)	336.2 (269.3 to 420)	4.7 (-16.8 to 33.2)
	Female	440 (335 to 566)	4.8 (3.7 to 6.1)	18.8 (-4.6 to 47.2)	443 (338 to 570)	5.1 (3.9 to 6.5)	18.4 (-5.2 to 46.1)	12700 (9540 to 16300)	122 (92.8 to 158.5)	17.7 (-7.3 to 46.9)
Greece	Both	9240 (7270 to 11500)	42 (32.7 to 53.1)	-2.8 (-24 to 23.1)	8640 (8030 to 9190)	37.6 (35.3 to 39.8)	-2.6 (-8.3 to 3.3)	172000 (162000 to 182000)	856.9 (806.2 to 908.5)	-4.6 (-10.2 to 1.5)
	Male	7180 (5650 to 9080)	70.3 (54.5 to 89.4)	-5 (-26.4 to 20.6)	6770 (6280 to 7220)	64.1 (60 to 68.2)	-4.5 (-10.9 to 2.2)	136000 (127000 to 144000)	1427.7 (1340.2 to 1517.5)	-7 (-13.1 to -0.5)
	Female	2060 (1610 to 2570)	17.5 (13.5 to 21.9)	6.1 (-17.5 to 35.2)	1870 (1670 to 2050)	14.9 (13.5 to 16.2)	5.4 (-3.6 to 14.8)	36600 (33200 to 39800)	351 (320.9 to 380)	5.3 (-4 to 15)
Greenland	Both	54 (45 to 64)	77.7 (64.4 to 90.6)	-12.8 (-25.7 to -0.1)	53 (43 to 63)	78.2 (63.9 to 92)	-13.2 (-26.9 to 0.5)	1310 (1050 to 1560)	1769 (1436.9 to 2101.5)	-14.7 (-28.9 to 0.1)
	Male	33 (26 to 39)	89.1 (71.9 to 104.1)	-13.3 (-26.9 to 0.2)	33 (26 to 39)	91.4 (72.5 to 107.9)	-13.2 (-28.8 to 2.2)	806 (629 to 976)	2017 (1578 to 2416.9)	-14.9 (-31 to 1.2)
	Female	21 (17 to 26)	65.4 (53.9 to 79.8)	-12.7 (-27.6 to 5.5)	20 (17 to 25)	64.4 (52.8 to 78.4)	-13.8 (-28.6 to 4.3)	500 (402 to 614)	1485 (1205.3 to 1815.2)	-14.7 (-29.8 to 4.3)

Grenada	Both	16 (14 to 18)	14.2 (12.8 to 15.8)	-4.8 (-15.8 to 6.6)	16 (14 to 18)	14.4 (13 to 16)	-6.5 (-17.1 to 4.4)	398 (351 to 450)	338.9 (300.8 to 379.3)	-4.1 (-16 to 8.6)
	Male	11 (10 to 12)	20.8 (18.8 to 23.1)	-22 (-31.7 to -12.2)	11 (10 to 12)	21.2 (19.1 to 23.5)	-25 (-34.1 to -15.9)	276 (245 to 309)	480.4 (429.4 to 536.7)	-17.5 (-27.8 to -6.3)
	Female	5 (4 to 6)	8.7 (7.5 to 10)	7.5 (-8 to 26.2)	5 (4 to 6)	8.9 (7.7 to 10.2)	6.8 (-8.3 to 24.4)	122 (105 to 141)	207.6 (179.2 to 240)	6.1 (-9.9 to 25.5)
Guam	Both	59 (49 to 70)	31.1 (26.1 to 37)	4.2 (-14.6 to 24.8)	60 (50 to 71)	31.9 (26.8 to 38)	3.8 (-14.8 to 23.9)	1460 (1210 to 1740)	760.7 (631.9 to 904.8)	4.8 (-14.2 to 25.8)
	Male	39 (33 to 47)	43.2 (35.8 to 51.7)	-1.1 (-18.6 to 19.5)	40 (33 to 48)	45 (37.6 to 53.5)	-1.5 (-18.9 to 19.3)	984 (816 to 1180)	1038.5 (863.4 to 1244.5)	-0.3 (-18.5 to 21.1)
	Female	20 (16 to 24)	20.2 (16.5 to 24.9)	14.8 (-10.6 to 42.4)	20 (16 to 25)	20.6 (16.8 to 25.4)	15.1 (-10.4 to 44)	477 (384 to 591)	493.3 (397.6 to 610.4)	14.8 (-10 to 42.5)
Guatemala	Both	867 (677 to 1100)	7.9 (6.2 to 9.9)	-9.3 (-31.4 to 18.5)	897 (702 to 1130)	8.3 (6.6 to 10.4)	-9.9 (-31.1 to 15.9)	21700 (16700 to 27500)	184.7 (142.5 to 233.6)	-10.3 (-33 to 18.1)
	Male	508 (393 to 643)	10.4 (8 to 13)	-9.9 (-33.1 to 19.7)	536 (415 to 674)	11.3 (8.8 to 14.1)	-9.9 (-32.6 to 18.5)	12500 (9550 to 15900)	236.8 (181 to 302.2)	-11.6 (-35.9 to 18.5)
	Female	359 (281 to 451)	5.9 (4.6 to 7.3)	-7.2 (-28.7 to 19.9)	361 (284 to 450)	6 (4.8 to 7.5)	-8.6 (-29.7 to 19.3)	9230 (7170 to 11700)	141.6 (109.8 to 179)	-7.2 (-29.7 to 24)
Guinea	Both	549 (421 to 707)	10.1 (7.8 to 12.9)	2.2 (-15.9 to 25.2)	570 (440 to 732)	10.7 (8.4 to 13.7)	1.7 (-16.2 to 24.2)	14900 (11200 to 19400)	256.2 (193.6 to 331)	0.9 (-18.6 to 24.1)
	Male	432 (327 to 562)	15.7 (12 to 20.2)	-1.8 (-20.2 to 21.7)	448 (343 to 581)	16.7 (13 to 21.4)	-2.5 (-20.2 to 21.1)	11700 (8750 to 15400)	398.6 (301.6 to 520.1)	-2.6 (-22.5 to 22.2)
	Female	118 (84 to 154)	4.4 (3.1 to 5.7)	11.6 (-11 to 38.9)	122 (86 to 159)	4.7 (3.3 to 6.1)	11.4 (-9.8 to 38)	3210 (2230 to 4280)	110 (77.5 to 145.8)	11.6 (-10.4 to 40.5)
Guinea-Bissau	Both	92 (59 to 133)	13.1 (8.6 to 18.7)	-0.9 (-17.2 to 21.4)	93 (59 to 134)	13.8 (9.1 to 19.6)	-0.7 (-16.5 to 21.1)	2560 (1640 to 3720)	325.4 (208.5 to 472.8)	-2.2 (-19.3 to 21.1)
	Male	68 (41 to 105)	21.9 (13.6 to 33)	-4.4 (-19.7 to 16.8)	69 (41 to 106)	23.4 (14.7 to 34.9)	-4 (-18.7 to 16.9)	1870 (1100 to 2930)	533.9 (319.8 to 821.4)	-5.4 (-21.2 to 17.8)
	Female	24 (18 to 33)	6.2 (4.6 to 8.2)	11 (-11.6 to 38.9)	24 (18 to 32)	6.4 (4.8 to 8.4)	10.7 (-11.2 to 37.3)	686 (491 to 917)	152.7 (111.4 to 205.6)	8.9 (-13.7 to 37.8)
Guyana	Both	55 (43 to 71)	8.7 (6.8 to 11)	4.8 (-19.1 to 33.2)	55 (43 to 70)	9 (7.1 to 11.3)	4.3 (-19 to 31.6)	1510 (1160 to 1930)	221.5 (171.5 to 281.3)	4 (-21 to 35)
	Male	35 (26 to 44)	11.8 (9.1 to 14.9)	1.9 (-23.3 to 33.2)	35 (26 to 44)	12.3 (9.5 to 15.4)	1.6 (-22.9 to 32.3)	938 (705 to 1220)	293.5 (223.8 to 376.8)	0.6 (-25.3 to 34.7)
	Female	21 (16 to 26)	6.1 (4.8 to 7.7)	12.2 (-14.3 to 45.5)	21 (16 to 26)	6.2 (4.9 to 7.9)	11.4 (-14.3 to 43.4)	567 (431 to 729)	158.1 (121.6 to 201.8)	11.6 (-15.9 to 46.7)
Haiti	Both	780 (481 to 1270)	11.3 (7.2 to 18.4)	0.3 (-16.9 to 22.6)	796 (495 to 1290)	12 (7.6 to 19.4)	0 (-17.2 to 22)	21500 (13200 to 34800)	284.3 (175.6 to 462.2)	-0.3 (-18.3 to 23.4)
	Male	546 (309 to 1010)	17 (9.9 to 31.3)	-0.3 (-19 to 25.3)	560 (320 to 1030)	18.1 (10.6 to 33.2)	-0.6 (-18.9 to 24.5)	14800 (8190 to 27300)	422 (239.6 to 778.7)	-0.6 (-20.4 to 26)
	Female	234 (151 to 332)	6.2 (4.1 to 8.7)	4.7 (-18.1 to 32.6)	236 (153 to 333)	6.5 (4.3 to 9.1)	4.5 (-16.8 to 31.9)	6700 (4360 to 9720)	161.6 (104.1 to 230.9)	3.3 (-18.7 to 32.5)

Honduras	Both	1380 (894 to 1970)	23 (15 to 32.7)	14.5 (-7.9 to 40.8)	1400 (908 to 1980)	24.1 (15.8 to 33.8)	13.5 (-6.8 to 38.9)	35000 (22900 to 50300)	553.2 (362.3 to 791.7)	11.9 (-11 to 42.3)
	Male	556 (344 to 793)	19.9 (12.2 to 28.4)	13.9 (-14.6 to 51.7)	579 (358 to 826)	21.2 (13 to 29.9)	13.8 (-13 to 52)	14100 (8870 to 19900)	480.2 (297.3 to 679.5)	10.8 (-16.3 to 50.3)
	Female	820 (492 to 1220)	25.8 (15.7 to 38)	13.7 (-12.1 to 47.8)	820 (494 to 1210)	26.7 (16.5 to 38.9)	11.8 (-12.7 to 43.9)	20800 (12500 to 31000)	618.3 (369.1 to 915.2)	11.9 (-15 to 48.5)
Hungary	Both	9510 (7850 to 11600)	51.9 (42.6 to 63.6)	-11.8 (-27.7 to 8.1)	8970 (7430 to 10800)	48.1 (39.6 to 58.4)	-12.8 (-28.4 to 5.8)	212000 (173000 to 259000)	1221 (992.7 to 1496)	-16.1 (-31.7 to 3)
	Male	5820 (4740 to 7100)	74.4 (60.6 to 90.6)	-18.1 (-33.6 to 0.9)	5510 (4500 to 6670)	70.3 (57.4 to 85)	-19 (-34 to -0.8)	133000 (107000 to 162000)	1716.8 (1390.4 to 2103.5)	-21.5 (-36.9 to -3.2)
	Female	3690 (3020 to 4510)	35.6 (29.1 to 43.8)	-2.2 (-21.3 to 20.3)	3460 (2860 to 4210)	32.4 (26.6 to 39.6)	-3.5 (-21.5 to 18.2)	79900 (65200 to 97800)	838.9 (680.2 to 1034.5)	-7.4 (-25.5 to 14.8)
Iceland	Both	187 (163 to 215)	34.5 (30.2 to 39.5)	-11.3 (-23.1 to 2.4)	147 (129 to 163)	26.5 (23.5 to 29.4)	-15.3 (-23.5 to -6.3)	3050 (2730 to 3380)	589.1 (529.2 to 653.2)	-13.6 (-22.2 to -4)
	Male	100 (87 to 114)	38.1 (33.3 to 43.5)	-12.1 (-24.5 to 1)	77 (69 to 86)	29.4 (26.2 to 32.6)	-15.8 (-24.9 to -5.1)	1630 (1460 to 1810)	637.4 (568.1 to 705)	-12.3 (-22.3 to -1.4)
	Female	87 (74 to 102)	31.4 (26.7 to 36.7)	-11.4 (-24.9 to 5.2)	69 (60 to 79)	23.9 (20.8 to 26.9)	-15.9 (-25.4 to -4.9)	1420 (1260 to 1600)	544.6 (485.1 to 611)	-15.8 (-25.3 to -5.1)
India	Both	87300 (71900 to 104000)	7.7 (6.4 to 9.1)	10.9 (-8.8 to 30.9)	89200 (73700 to 105000)	8.1 (6.7 to 9.5)	10.4 (-8.9 to 29.6)	2280000 (1870000 to 2690000)	190.2 (156.6 to 224.7)	8.8 (-10.5 to 28.4)
	Male	61300 (48100 to 75900)	11.2 (8.8 to 13.7)	3.4 (-18.9 to 27.2)	62500 (49100 to 76400)	11.7 (9.2 to 14.3)	3.1 (-18.5 to 26.9)	1590000 (1230000 to 1950000)	271.3 (211.9 to 331.8)	1.1 (-20.9 to 25.6)
	Female	26100 (20300 to 32500)	4.5 (3.5 to 5.6)	36.1 (5.6 to 71.2)	26700 (21000 to 33500)	4.7 (3.7 to 5.9)	35.3 (3.7 to 69.7)	689000 (534000 to 874000)	112.9 (87.7 to 142.7)	34.4 (2.3 to 70.8)
Indonesia	Both	48200 (35300 to 59300)	22.9 (16.8 to 28)	7 (-12.5 to 27.6)	49400 (36100 to 61100)	24.4 (17.9 to 30.2)	6.9 (-11.8 to 24.6)	1280000 (928000 to 1600000)	555.8 (404.2 to 685.3)	4.5 (-15.1 to 25.5)
	Male	32900 (24500 to 43300)	33.2 (25.1 to 42.8)	5.5 (-18.3 to 33.4)	33800 (24800 to 44200)	35.8 (27.1 to 45.7)	5.3 (-17.8 to 33.5)	872000 (632000 to 1170000)	796 (584.5 to 1045.6)	3.1 (-21.1 to 33.1)
	Female	15300 (8550 to 22900)	13.9 (7.7 to 20.6)	10.4 (-14.1 to 38.8)	15600 (8700 to 23600)	14.7 (8.2 to 21.6)	10.7 (-13.1 to 36.8)	408000 (227000 to 623000)	339.2 (190 to 516.4)	7.4 (-17.2 to 38.6)
Iran	Both	8710 (8040 to 9370)	12.2 (11.3 to 13.2)	14.2 (6.6 to 22.1)	8920 (8250 to 9600)	12.9 (11.9 to 13.9)	12.7 (5.8 to 20.7)	219000 (203000 to 235000)	286.8 (266.1 to 307.6)	12.2 (4.8 to 20.2)

	Male	5880 (5360 to 6410)	16.6 (15.1 to 18.1)	8 (0.2 to 16.8)	6160 (5640 to 6730)	17.8 (16.2 to 19.4)	7.5 (-0.3 to 16.4)	150000 (137000 to 164000)	396.9 (364.1 to 434.8)	7.4 (-0.7 to 17.1)
	Female	2830 (2550 to 3080)	7.9 (7.1 to 8.7)	32.7 (18.7 to 45.3)	2770 (2490 to 3020)	8 (7.2 to 8.8)	29.7 (15.9 to 42)	68700 (62600 to 74000)	178.2 (161.4 to 192.4)	26.1 (13.2 to 37.4)
Iraq	Both	4150 (3200 to 5130)	18.7 (14.5 to 22.7)	20.6 (0.1 to 45.9)	4230 (3280 to 5190)	19.9 (15.6 to 23.8)	21.1 (1 to 45.9)	111000 (84200 to 140000)	451.6 (346 to 557.5)	16.3 (-4.5 to 41.5)
	Male	3000 (2290 to 3680)	28.4 (22 to 34)	16.3 (-4.7 to 44.7)	3110 (2380 to 3780)	30.9 (24.2 to 36.8)	17.7 (-3.2 to 46.4)	79500 (60500 to 101000)	675.6 (516.7 to 825.1)	12.4 (-9.2 to 41.5)
	Female	1160 (880 to 1480)	9.8 (7.5 to 12.3)	37 (7.6 to 76.2)	1130 (861 to 1430)	9.9 (7.7 to 12.2)	35.8 (7.8 to 74.3)	31200 (23300 to 41000)	239.6 (181 to 307.8)	30.9 (2 to 70.3)
Ireland	Both	2500 (1980 to 3140)	33.2 (26.2 to 41.7)	-8 (-27.9 to 16.4)	2190 (2010 to 2370)	28.8 (26.4 to 31)	-12.3 (-18.6 to -6)	43600 (40200 to 46900)	593.6 (548 to 637.7)	-13.4 (-19.9 to -6.9)
	Male	1360 (1040 to 1730)	38.2 (29.3 to 48.7)	-15.3 (-35.3 to 7.8)	1220 (1100 to 1330)	34.3 (30.9 to 37.4)	-18.9 (-26.1 to -11.6)	24300 (22000 to 26300)	686.3 (623.6 to 743.1)	-19.8 (-27.2 to -12.6)
	Female	1140 (904 to 1460)	29 (23 to 37)	0.9 (-21.4 to 28.2)	974 (866 to 1080)	24.2 (21.6 to 26.9)	-4.4 (-13 to 4.9)	19300 (17300 to 21500)	510.1 (456.9 to 571)	-4.7 (-13.4 to 5.1)
Israel	Both	2670 (2090 to 3390)	23.4 (18.1 to 29.7)	-6.7 (-26.8 to 18.1)	2520 (2310 to 2680)	21.7 (20 to 23.1)	-8.6 (-14 to -2.9)	52600 (49100 to 55900)	478.1 (446.7 to 507.5)	-9.9 (-15.5 to -4.1)
	Male	1750 (1350 to 2240)	33.4 (25.8 to 42.7)	-9.9 (-30.8 to 15.2)	1650 (1530 to 1770)	31.4 (29 to 33.6)	-11.6 (-17.2 to -5.5)	35800 (33300 to 38300)	694.5 (646.7 to 745.2)	-12.4 (-18 to -5.8)
	Female	918 (724 to 1170)	14.6 (11.5 to 18.6)	-2 (-22.4 to 24.1)	867 (769 to 953)	13.4 (12 to 14.7)	-4.4 (-12.5 to 3.9)	16900 (15200 to 18400)	285.3 (258.6 to 310.4)	-6.2 (-14.1 to 2.6)
Italy	Both	41700 (34300 to 49600)	30.2 (24.6 to 36)	-10.8 (-26.4 to 6.6)	37000 (33900 to 38900)	25.6 (23.8 to 26.7)	-11.3 (-13.8 to -9)	699000 (656000 to 729000)	552 (523.3 to 573.3)	-12.8 (-15.2 to -10.5)
	Male	29200 (22600 to 37000)	46.3 (35.9 to 58.9)	-16.8 (-35 to 5.3)	26600 (24600 to 28000)	41.1 (38.5 to 43.2)	-17.2 (-20.1 to -14.5)	500000 (472000 to 524000)	842 (797.1 to 881.3)	-18.1 (-20.8 to -15.5)
	Female	12500 (9730 to 15800)	17.1 (13.3 to 21.6)	1.3 (-20.3 to 28.2)	10400 (9230 to 11200)	13.2 (12.1 to 14)	0.8 (-2.8 to 4.9)	199000 (183000 to 210000)	305 (286 to 319.9)	-0.8 (-4.2 to 3.3)
Jamaica	Both	575 (454 to 718)	19.5 (15.3 to 24.3)	-3.8 (-24.9 to 21.1)	572 (451 to 709)	19.3 (15.2 to 24)	-3.6 (-24.3 to 21.2)	14000 (10800 to 17600)	471.9 (365.3 to 593)	-2.8 (-24.7 to 23.6)
	Male	447 (348 to 560)	31.2 (24.3 to 39)	-8 (-27.9 to 16.1)	441 (345 to 549)	31 (24.3 to 38.6)	-8 (-28 to 15.7)	10900 (8400 to 13800)	750.6 (576.8 to 948.4)	-6.7 (-27.9 to 19.2)
	Female	128 (98 to 165)	8.3 (6.4 to 10.7)	12.6 (-14.7 to 46.1)	131 (101 to 168)	8.3 (6.4 to 10.7)	13.1 (-14 to 46.7)	3070 (2320 to 3990)	201.8 (152.5 to 263)	13 (-15.6 to 49)
Japan	Both	117000 (95800 to 137000)	30.7 (25.5 to 36)	-11.1 (-23.7 to 3.9)	86000 (73400 to 92700)	21.2 (18.9 to 22.5)	-11.4 (-14 to -9.1)	1350000 (1210000 to 1430000)	408 (378.7 to 427.4)	-14.2 (-17 to -11.8)

	Male	81700 (66700 to 99600)	49 (40 to 59.9)	-15.3 (-30.2 to 2.9)	59100 (53000 to 62900)	34.4 (31.3 to 36.4)	-16.2 (-19.3 to -13.7)	966000 (891000 to 1020000)	638.5 (595.3 to 669.4)	-18 (-21.4 to -15.3)
	Female	35100 (26600 to 42600)	15.9 (12.5 to 19.5)	-3.8 (-20.4 to 15.5)	26900 (20600 to 30300)	10.9 (9 to 12)	-3.8 (-7.5 to -0.1)	381000 (316000 to 419000)	210.6 (186.7 to 225.9)	-6.8 (-10.6 to -3)
Jordan	Both	914 (748 to 1110)	14.3 (11.9 to 17.4)	-5.1 (-22.9 to 14.4)	917 (749 to 1110)	15.1 (12.5 to 18.2)	-6.3 (-24.1 to 13.5)	24200 (19800 to 29400)	338 (276.6 to 409.8)	-6.2 (-24.7 to 14.3)
	Male	697 (537 to 882)	20.9 (16.3 to 26.2)	-9.7 (-30.3 to 14.1)	711 (551 to 897)	22.3 (17.5 to 27.9)	-10.2 (-30.4 to 13.3)	18700 (14300 to 23700)	497.8 (385.8 to 628.3)	-10.6 (-31.9 to 14.2)
	Female	218 (171 to 277)	7.2 (5.7 to 9)	12 (-11.5 to 40)	206 (163 to 261)	7.2 (5.7 to 9.1)	10.4 (-12 to 40)	5580 (4370 to 7140)	161.9 (128.3 to 205.7)	8 (-15.2 to 37.1)
Kazakhstan	Both	3830 (3260 to 4410)	21.4 (18.4 to 24.4)	-17.5 (-29.8 to -4.4)	3720 (3180 to 4260)	21.1 (18.2 to 24.1)	-18.9 (-30.9 to -6.1)	99100 (84100 to 115000)	530 (452 to 608.7)	-20.6 (-32.6 to -7.5)
	Male	3050 (2580 to 3560)	41.8 (35.5 to 48.5)	-21 (-33.4 to -7.8)	2950 (2500 to 3440)	41.8 (35.6 to 48.3)	-22.1 (-34.2 to -9.2)	79500 (66600 to 93300)	1011.2 (856.3 to 1179.9)	-24 (-36.4 to -11.1)
	Female	782 (656 to 915)	7.7 (6.5 to 9)	-4.3 (-20.6 to 13)	768 (646 to 894)	7.6 (6.4 to 8.9)	-6.6 (-22.4 to 9.9)	19600 (16300 to 23100)	187.7 (156.5 to 220.9)	-7.3 (-23.9 to 10.4)
Kenya	Both	1180 (977 to 1430)	5.6 (4.7 to 6.7)	0.7 (-16 to 16.9)	1280 (1070 to 1550)	6.3 (5.3 to 7.5)	0.6 (-13.5 to 16.4)	33700 (27700 to 41400)	143.5 (119 to 174.3)	-1.3 (-16 to 15.2)
	Male	895 (723 to 1090)	9.4 (7.7 to 11.4)	-4.9 (-23.3 to 16.3)	958 (771 to 1160)	10.5 (8.7 to 12.5)	-4.6 (-21.4 to 15.2)	24700 (19600 to 30400)	228.4 (184.9 to 276.2)	-6.4 (-24.1 to 15.6)
	Female	286 (195 to 434)	2.5 (1.7 to 3.7)	17.7 (-8 to 58.4)	324 (222 to 483)	2.9 (2 to 4.2)	14.7 (-9.2 to 47.9)	8940 (6020 to 13400)	69.5 (47.3 to 104.4)	13.2 (-11.2 to 48)
Kiribati	Both	14 (10 to 18)	19.2 (14.4 to 24.6)	0.6 (-15.1 to 17.5)	14 (10 to 18)	20.1 (15.3 to 25.6)	1 (-14.1 to 17.6)	413 (300 to 550)	514 (381.2 to 673)	0.1 (-16.6 to 18.6)
	Male	11 (8 to 15)	36.7 (27.2 to 47.5)	0.4 (-16.4 to 19.6)	11 (8 to 15)	38.7 (28.9 to 49.9)	0.7 (-15.7 to 19.4)	341 (244 to 461)	962.1 (697.3 to 1266.5)	0.1 (-18.1 to 21.7)
	Female	3 (2 to 3)	6.7 (4.9 to 8.9)	5.7 (-13.9 to 29.1)	3 (2 to 3)	7.2 (5.3 to 9.5)	6 (-13.1 to 27.9)	71 (51 to 96)	171.2 (124 to 226.9)	4 (-15.8 to 28.1)
Kuwait	Both	225 (185 to 272)	10.1 (8.2 to 12.2)	0.8 (-17.4 to 21.8)	228 (185 to 275)	10.8 (8.7 to 13)	0.4 (-17.8 to 21.3)	5540 (4540 to 6680)	214.8 (174.7 to 260.7)	-3 (-20.7 to 17.8)
	Male	173 (133 to 216)	13.3 (10.3 to 16.6)	6.5 (-16.6 to 33.8)	181 (140 to 225)	14.5 (11.3 to 18.1)	6.5 (-16.3 to 34.1)	4260 (3280 to 5350)	287.6 (222.7 to 360.3)	3.2 (-19.3 to 30.3)
	Female	53 (41 to 66)	5.2 (4.1 to 6.5)	-12.5 (-29.7 to 8.9)	47 (37 to 59)	5 (3.9 to 6.1)	-15.5 (-31.8 to 4.3)	1280 (989 to 1620)	104.2 (82.3 to 130.2)	-17.3 (-33.8 to 3.3)
Kyrgyzstan	Both	569 (493 to 647)	12.2 (10.6 to 13.9)	-6.8 (-18.5 to 5.7)	560 (486 to 636)	12.4 (10.8 to 14)	-7 (-18.6 to 5.3)	15400 (13300 to 17500)	308.1 (266.8 to 350.8)	-9.8 (-21.9 to 2.8)
	Male	430 (371 to 492)	21.3 (18.5 to 24.3)	-9.1 (-21.3 to 4.2)	422 (364 to 483)	21.6 (18.8 to 24.7)	-9.1 (-20.9 to 3.8)	11700 (10000 to 13400)	532.3 (460.1 to 608.8)	-12.2 (-24.5 to 0.5)
	Female	139 (115 to 164)	5.4 (4.5 to 6.3)	3 (-14.1 to 21.2)	139 (115 to 163)	5.5 (4.6 to 6.4)	1.8 (-15 to 19.7)	3660 (3010 to 4340)	133.4 (110.8 to 157.9)	0.6 (-16.8 to 18.9)

Laos	Both	973 (718 to 1260)	22.8 (17 to 29)	3.2 (-15.6 to 25.5)	1000 (742 to 1290)	24.3 (18.3 to 30.7)	3.3 (-15.1 to 25.2)	26500 (19100 to 35100)	566.5 (418.1 to 736.9)	1.2 (-18.4 to 24.3)
	Male	713 (519 to 923)	35.3 (26.3 to 44.8)	-0.6 (-19.7 to 22.4)	736 (538 to 944)	38 (28.3 to 48.3)	-0.6 (-18.6 to 21.5)	19200 (13700 to 25300)	861.3 (624.1 to 1112.8)	-2.5 (-22 to 21.2)
	Female	260 (186 to 372)	11.4 (8.4 to 16.5)	13.9 (-8.8 to 40.6)	264 (191 to 380)	12.1 (8.9 to 17.4)	14.3 (-8.3 to 41.1)	7300 (5150 to 10500)	292.9 (208.9 to 416.9)	11.4 (-11.4 to 37.2)
Latvia	Both	1020 (838 to 1240)	26.9 (22 to 33)	-13.6 (-29.6 to 6.5)	950 (788 to 1150)	24.6 (20.3 to 29.9)	-15.3 (-30.9 to 3.3)	21200 (17400 to 25900)	602 (489.8 to 741.4)	-16.7 (-32.9 to 3.1)
	Male	777 (608 to 987)	53.6 (41.9 to 68)	-20 (-37.9 to 2.3)	718 (564 to 913)	49.4 (38.8 to 62.7)	-21.7 (-38.8 to -0.2)	16500 (12800 to 21200)	1156.9 (899.1 to 1483)	-22.6 (-40.8 to -0.1)
	Female	241 (181 to 317)	10 (7.4 to 13.3)	2.9 (-23.7 to 37.1)	232 (176 to 304)	9.3 (6.9 to 12.4)	0.3 (-25.3 to 33.2)	4690 (3480 to 6280)	220.9 (160.2 to 297.7)	-0.3 (-27.2 to 34.5)
Lebanon	Both	1420 (1170 to 1870)	27.3 (22.4 to 35.8)	-1.8 (-17.3 to 13.6)	1430 (1180 to 1900)	27.5 (22.7 to 36.4)	-5.1 (-19 to 10.1)	32700 (26400 to 42600)	629.6 (507.3 to 817.1)	-3.7 (-18.7 to 13.1)
	Male	914 (729 to 1200)	38.9 (30.9 to 51.4)	-5.2 (-20.4 to 12.5)	961 (768 to 1280)	40.9 (32.7 to 54.7)	-6.6 (-20.8 to 11)	21800 (17000 to 28500)	926 (725.1 to 1215.4)	-6.1 (-20.7 to 11.3)
	Female	507 (375 to 703)	17.8 (13.1 to 24.5)	14 (-13.6 to 39.1)	472 (347 to 656)	16.5 (12.1 to 22.8)	8.5 (-16.7 to 30.5)	10900 (8040 to 14800)	385 (281.4 to 520.5)	9.7 (-16.4 to 34.5)
Lesotho	Both	233 (158 to 326)	18.1 (12.4 to 25)	4 (-16.9 to 27.6)	240 (163 to 333)	19.1 (13.1 to 26.4)	3.4 (-17 to 26.6)	6740 (4490 to 9600)	487.4 (326.5 to 686.5)	2.1 (-19.5 to 26.3)
	Male	174 (119 to 251)	33.1 (23 to 46.6)	1.4 (-16.4 to 22)	179 (122 to 256)	35.2 (24.5 to 49.1)	0.5 (-16.8 to 20.8)	5200 (3470 to 7510)	887.6 (607.4 to 1274)	-0.4 (-18.3 to 20.9)
	Female	59 (35 to 93)	8.1 (4.8 to 12.5)	13.6 (-20.5 to 51.5)	61 (36 to 96)	8.7 (5.3 to 13.2)	13.5 (-20 to 50.7)	1540 (883 to 2450)	199.4 (116.3 to 317.6)	12.5 (-23.4 to 54.6)
Liberia	Both	175 (118 to 241)	9.2 (6.4 to 12.6)	-0.5 (-20.3 to 22.6)	182 (125 to 250)	10 (7 to 13.6)	-0.6 (-19.9 to 22.8)	4690 (3140 to 6500)	218.8 (148 to 302.7)	-0.4 (-21.1 to 25.2)
	Male	125 (81 to 181)	13.2 (8.6 to 18.7)	-4.1 (-23.1 to 19.5)	131 (86 to 188)	14.4 (9.6 to 20.2)	-4 (-22.7 to 19.9)	3310 (2110 to 4830)	308.4 (201.1 to 445.7)	-4.4 (-24 to 20.7)
	Female	49 (32 to 66)	5.1 (3.3 to 6.7)	11 (-12.9 to 37.7)	51 (33 to 67)	5.5 (3.6 to 7.2)	10.9 (-12.7 to 37.2)	1380 (885 to 1840)	124.8 (81.2 to 165.3)	10.9 (-13.7 to 39.8)
Libya	Both	925 (688 to 1180)	18.5 (13.7 to 23.5)	-5.5 (-27.8 to 19.9)	946 (703 to 1200)	19.4 (14.4 to 24.5)	-5.6 (-27.8 to 19.8)	25000 (18500 to 32000)	457.8 (341.3 to 584)	-5.3 (-28.2 to 20.6)
	Male	797 (584 to 1020)	31.4 (23 to 40.2)	-5.1 (-27.9 to 22.4)	821 (601 to 1050)	33.2 (24.3 to 42.5)	-5.3 (-27.8 to 21.6)	21600 (15900 to 27800)	778.8 (569.1 to 1000.8)	-4.9 (-28.1 to 22.9)
	Female	128 (82 to 175)	5.1 (3.2 to 6.9)	-2.4 (-28.6 to 26.7)	125 (80 to 169)	5.1 (3.2 to 6.9)	-2.3 (-28.8 to 26.2)	3390 (2130 to 4710)	123.7 (78.8 to 170.1)	-2 (-29.4 to 26.7)
Lithuania	Both	1400 (1140 to 1690)	25.4 (20.6 to 31)	-14.7 (-30.5 to 4.4)	1310 (1070 to 1590)	23.6 (19.1 to 28.5)	-15.6 (-30.7 to 2.7)	29600 (23900 to 36000)	575.8 (462.7 to 706.5)	-17 (-33.3 to 2.2)
	Male	1080 (866 to 1320)	51 (40.9 to 62.1)	-20.1 (-35 to -2.9)	1020 (818 to 1230)	47.9 (38.6 to 58.1)	-20.8 (-35.6 to -3.8)	23600 (18700 to 29000)	1123.6 (893.4 to 1381)	-21.9 (-37.5 to -4.1)
	Female	312 (253 to 384)	9 (7.2 to 11.2)	6 (-14.6 to 31.5)	295 (237 to 359)	8.2 (6.6 to 10.1)	4.1 (-16 to 28.8)	5990 (4800 to 7380)	197.1 (155.9 to 243.6)	3 (-18.6 to 28.8)

Luxembourg	Both	318 (261 to 386)	32.5 (26.7 to 39.6)	-17.1 (-32.3 to 2)	289 (254 to 328)	29.1 (25.5 to 32.9)	-15.8 (-25.8 to -4)	6210 (5480 to 7040)	655.2 (577.5 to 742.7)	-17.3 (-27.3 to -5.6)
	Male	217 (177 to 264)	47 (38.4 to 57.2)	-22.1 (-37 to -3.6)	198 (173 to 226)	43.1 (37.7 to 49.2)	-22.1 (-32.4 to -10.4)	4250 (3700 to 4870)	922.3 (804 to 1054.9)	-22.5 (-33.5 to -10.6)
	Female	101 (80 to 129)	19.9 (15.8 to 25.2)	-11.8 (-31 to 12.9)	91 (76 to 108)	17.3 (14.5 to 20.4)	-8.2 (-22.7 to 9.6)	1960 (1650 to 2310)	408 (342.4 to 482.2)	-8.9 (-22.8 to 7.8)
Madagascar	Both	678 (495 to 899)	6.4 (4.7 to 8.3)	0.3 (-19.9 to 24.1)	690 (507 to 912)	6.8 (5.1 to 8.8)	0.3 (-19.3 to 23.2)	19600 (14100 to 26000)	161.1 (118.6 to 212.2)	0.1 (-20.8 to 25.9)
	Male	460 (326 to 626)	9.3 (6.7 to 12.4)	-4.8 (-24.3 to 19.1)	473 (337 to 640)	10 (7.3 to 13.3)	-4.7 (-23.8 to 18.8)	13000 (9130 to 17900)	228.2 (163.7 to 308.3)	-5.2 (-26.2 to 19.4)
	Female	217 (151 to 297)	3.7 (2.6 to 5)	13.7 (-12.7 to 45.3)	218 (152 to 296)	3.9 (2.8 to 5.2)	13.6 (-12.2 to 44.9)	6560 (4570 to 8970)	98.7 (69 to 134.1)	12.9 (-13.3 to 45.3)
Malawi	Both	416 (326 to 521)	5.9 (4.7 to 7.3)	-3.1 (-20.6 to 15.1)	432 (341 to 537)	6.4 (5.1 to 7.8)	-2.7 (-19.7 to 14.8)	11100 (8510 to 14200)	145.3 (113.2 to 182.3)	-3.9 (-22.8 to 16.4)
	Male	293 (221 to 378)	9.8 (7.6 to 12.5)	-8.3 (-26.3 to 12.1)	304 (233 to 391)	10.6 (8.3 to 13.4)	-8 (-25.7 to 11.6)	7830 (5810 to 10300)	233.3 (177.4 to 300.7)	-8.9 (-27.9 to 13.7)
	Female	123 (90 to 156)	3.1 (2.3 to 4)	12.5 (-12.9 to 41)	128 (95 to 165)	3.3 (2.5 to 4.3)	12.9 (-12.2 to 41.2)	3300 (2380 to 4280)	76.7 (55.9 to 97.7)	11.9 (-15.1 to 42.3)
Malaysia	Both	5170 (4000 to 6560)	19.8 (15.4 to 25.1)	-7.3 (-27.5 to 16.3)	5220 (4060 to 6640)	20.6 (16 to 26)	-9.3 (-28.9 to 13.3)	125000 (95800 to 159000)	453 (349 to 573.3)	-8 (-28.8 to 16.1)
	Male	3640 (2790 to 4630)	28 (21.6 to 35.6)	-6.8 (-28.2 to 18.3)	3690 (2830 to 4680)	29.2 (22.6 to 37.2)	-8.8 (-29.3 to 15.1)	88700 (67500 to 113000)	640.7 (490.4 to 816.7)	-7.8 (-29.1 to 17.1)
	Female	1520 (1160 to 1930)	11.7 (9 to 14.8)	-9 (-29.1 to 14)	1530 (1180 to 1950)	12.1 (9.3 to 15.4)	-11.7 (-30.8 to 10.1)	36800 (28100 to 47100)	266.5 (203.4 to 339.4)	-8.6 (-29.5 to 16.4)
Maldives	Both	26 (22 to 32)	9.6 (7.9 to 11.4)	-3 (-19.8 to 16.6)	27 (22 to 32)	10.2 (8.4 to 12.1)	-4.9 (-21.7 to 14)	620 (511 to 742)	199.4 (163.9 to 237.7)	-4.5 (-21.6 to 14.7)
	Male	20 (17 to 24)	13.7 (11.1 to 16.6)	-3.5 (-22.2 to 17.6)	21 (17 to 25)	14.7 (11.9 to 17.7)	-4.8 (-23.1 to 15.9)	478 (389 to 577)	283.5 (229.7 to 342)	-6.3 (-25.2 to 14.5)
	Female	6 (5 to 8)	4.8 (3.9 to 5.9)	6.2 (-14.1 to 31.5)	6 (5 to 8)	5.1 (4 to 6.2)	4.3 (-15.8 to 29.9)	142 (114 to 174)	101.3 (81.2 to 123.7)	3.1 (-17 to 27.6)
Mali	Both	595 (454 to 776)	7.2 (5.5 to 9.3)	4 (-12.1 to 23.8)	618 (473 to 805)	7.7 (5.9 to 9.9)	3.6 (-12.6 to 22.2)	15800 (11800 to 21000)	175.8 (133.5 to 230.1)	4.3 (-13.3 to 25)
	Male	419 (289 to 559)	9.9 (6.9 to 13.1)	1.3 (-17.7 to 23.2)	438 (304 to 585)	10.6 (7.5 to 14)	0.8 (-17.6 to 21.8)	11000 (7460 to 14900)	239.3 (164.9 to 320.3)	1.6 (-17.7 to 23.8)
	Female	176 (128 to 227)	4.3 (3.2 to 5.6)	10.7 (-9.6 to 34.5)	180 (133 to 232)	4.6 (3.4 to 5.9)	10.3 (-9.5 to 34)	4820 (3480 to 6330)	107.3 (78.5 to 138.9)	10.5 (-10.4 to 35.8)
Malta	Both	210 (180 to 248)	23 (19.6 to 27.1)	-8.3 (-22.2 to 8.7)	194 (171 to 218)	20.7 (18.4 to 23.2)	-10.6 (-20.5 to -0.5)	3980 (3520 to 4460)	464.4 (412.9 to 521.8)	-9.5 (-19.8 to 1.5)
	Male	168 (142 to 198)	39 (33.3 to 45.9)	-15.2 (-28.4 to 0.9)	157 (139 to 176)	36.2 (32.3 to 40.6)	-16.7 (-25.5 to -6.6)	3200 (2840 to 3580)	771.8 (685.9 to 859.9)	-15 (-24.3 to -4.5)
	Female	42 (34 to 52)	9.1 (7.5 to 11.2)	10.1 (-9.6 to 33.8)	37 (31 to 44)	7.7 (6.5 to 9)	4.1 (-11.4 to 21.4)	778 (656 to 914)	185 (157.8 to 217)	4.5 (-11.4 to 21.4)

Marshall Islands	Both	10 (6 to 15)	30.4 (19.1 to 44.3)	1.3 (-15.9 to 22.7)	10 (6 to 15)	32.4 (20.7 to 47.2)	1.6 (-15.6 to 23.3)	290 (172 to 436)	758.5 (467.1 to 1119.1)	0.1 (-17.6 to 21.5)
	Male	7 (4 to 12)	43.8 (25.7 to 69.1)	-0.6 (-18.1 to 21.1)	7 (4 to 12)	46.7 (28 to 73.4)	-0.6 (-17.8 to 21.1)	215 (116 to 350)	1095.6 (615.9 to 1764.9)	-1.3 (-19.3 to 20.9)
	Female	3 (2 to 4)	15.9 (10.7 to 22.3)	8 (-13.3 to 32.4)	3 (2 to 4)	16.9 (11.5 to 23.5)	8 (-13.7 to 32.1)	75 (49 to 108)	400.4 (268.2 to 571)	6.7 (-14.1 to 31)
Mauritania	Both	213 (149 to 297)	10.7 (7.6 to 14.6)	3.4 (-19.5 to 27.2)	225 (158 to 309)	11.6 (8.3 to 15.6)	3.5 (-18.6 to 26.5)	5340 (3540 to 7710)	249.9 (170.4 to 353.2)	1 (-23.1 to 25.3)
	Male	140 (91 to 217)	14 (9.4 to 21.1)	0.4 (-22.8 to 23.9)	149 (98 to 227)	15.3 (10.3 to 22.7)	0.4 (-21.9 to 23.8)	3450 (2120 to 5530)	325.4 (204.4 to 511.1)	-1.8 (-27 to 22.5)
	Female	73 (51 to 100)	7.2 (5.2 to 9.8)	9.8 (-16.5 to 35.2)	76 (54 to 103)	7.8 (5.6 to 10.5)	9.8 (-15.1 to 34.9)	1890 (1270 to 2610)	172.7 (119.2 to 237.5)	7.2 (-19.5 to 33.6)
Mauritius	Both	207 (169 to 250)	11.8 (9.7 to 14.3)	-1.5 (-20 to 20.2)	209 (171 to 252)	12.2 (10 to 14.6)	-2.3 (-20.3 to 18.6)	4950 (3990 to 6030)	277.9 (225.1 to 337.2)	-3 (-22.6 to 19.4)
	Male	146 (118 to 179)	18.7 (15.2 to 22.6)	-5.9 (-24.7 to 16.2)	148 (120 to 181)	19.5 (15.8 to 23.5)	-6.7 (-25.3 to 14.5)	3480 (2780 to 4280)	422.5 (340.9 to 517.1)	-7 (-25.7 to 15.7)
	Female	61 (49 to 74)	6.5 (5.2 to 7.8)	6 (-14.8 to 29.4)	61 (49 to 74)	6.5 (5.3 to 7.9)	5.1 (-15 to 27.8)	1470 (1170 to 1800)	158 (126.1 to 193.5)	4.1 (-17.4 to 28.5)
Mexico	Both	10900 (9400 to 12600)	9.5 (8.2 to 11)	-2.6 (-15.5 to 11.6)	11000 (9430 to 12700)	9.7 (8.4 to 11.2)	-4.1 (-17.1 to 10)	247000 (211000 to 285000)	208.3 (178.4 to 240.6)	-3.2 (-16.7 to 11.9)
	Male	6930 (5680 to 8480)	13.2 (10.8 to 16)	-3.6 (-20.7 to 17.2)	7180 (5880 to 8670)	13.8 (11.3 to 16.7)	-4.5 (-21.1 to 14.6)	159000 (131000 to 194000)	290.4 (238.5 to 353.1)	-3.9 (-21.3 to 16.4)
	Female	3960 (3270 to 4800)	6.4 (5.3 to 7.7)	0.6 (-17 to 21.2)	3830 (3120 to 4660)	6.3 (5.1 to 7.6)	-1.8 (-19.1 to 19.4)	87400 (70800 to 107000)	137.4 (111.5 to 168.4)	-0.7 (-19.1 to 22)
Federatead States of Micronesia	Both	22 (13 to 33)	31.3 (19.2 to 45.6)	5.4 (-15 to 26)	22 (13 to 32)	32.9 (20.5 to 47.2)	5.1 (-14 to 25.2)	619 (354 to 948)	793.7 (469.7 to 1183.1)	4.7 (-18.1 to 27.5)
	Male	16 (9 to 26)	48.4 (28.8 to 75.9)	3 (-18.5 to 24.5)	16 (9 to 25)	51.4 (31.3 to 79.4)	2.7 (-17.4 to 22.7)	453 (239 to 763)	1210.4 (685 to 1944.2)	2.3 (-21.6 to 24.9)
	Female	6 (4 to 9)	17 (11 to 24.1)	10.4 (-11.6 to 36.9)	6 (4 to 9)	18 (11.8 to 25.4)	10.1 (-11.5 to 35.9)	166 (103 to 247)	423.8 (271.3 to 615.2)	9.4 (-13.7 to 36)
Monaco	Both	68 (55 to 80)	75.6 (61.4 to 90.8)	-5.3 (-21.8 to 12.4)	60 (49 to 71)	64.2 (52.3 to 76.9)	-6.4 (-22.1 to 10.9)	1210 (976 to 1450)	1483.3 (1181.9 to 1811.7)	-7.1 (-24.1 to 11.4)
	Male	43 (36 to 50)	101.2 (83.8 to 120)	-6.2 (-21.2 to 11.4)	38 (32 to 45)	88.4 (74 to 104.5)	-7.3 (-22.1 to 8.8)	781 (645 to 929)	1986.6 (1610.3 to 2390.2)	-7.7 (-24 to 9.8)
	Female	25 (19 to 31)	52.8 (39.2 to 66.4)	-4.5 (-25.3 to 18.8)	22 (16 to 27)	43.1 (32.1 to 53.8)	-5.7 (-25.8 to 17.1)	425 (315 to 534)	1020.6 (751.9 to 1296.9)	-6.6 (-26.9 to 17.2)
Mongolia	Both	662 (512 to 863)	31.2 (24.7 to 39.7)	1.3 (-19.1 to 26.6)	672 (523 to 869)	33.4 (26.5 to 42)	1.3 (-18.7 to 25.2)	17500 (13400 to 23200)	727.7 (568 to 941.3)	-0.6 (-21.8 to 26.6)
	Male	521 (391 to 686)	58.1 (45 to 74.3)	2.1 (-20.6 to 29.4)	525 (397 to 686)	62.8 (49.2 to 79.4)	1.9 (-19.8 to 27.6)	14000 (10400 to 18700)	1335.3 (1008.7 to 1737.4)	0.7 (-22.2 to 30.2)

	Female	141 (109 to 182)	12.5 (9.9 to 15.8)	0.4 (-21.8 to 28.1)	146 (114 to 188)	13.7 (10.9 to 17.3)	0.1 (-21.1 to 26.2)	3520 (2710 to 4620)	277.4 (216.3 to 357.4)	-0.7 (-23.3 to 26.4)
Montenegro	Both	563 (461 to 685)	56.7 (46.5 to 68.9)	-0.4 (-17.6 to 19.9)	531 (437 to 643)	53.4 (43.8 to 64.4)	-1.7 (-18 to 17.7)	13100 (10700 to 16000)	1343.6 (1092.2 to 1633.4)	-3.9 (-20.6 to 16.3)
	Male	422 (341 to 522)	92.8 (75.3 to 114.3)	-0.6 (-19.6 to 21.9)	400 (323 to 493)	88.6 (71.9 to 108.8)	-1.6 (-19.6 to 20.3)	9870 (7880 to 12300)	2157.3 (1730 to 2682)	-4.3 (-22.3 to 17.2)
	Female	141 (113 to 173)	26.7 (21.5 to 32.7)	-0.1 (-18.4 to 21.5)	131 (105 to 161)	24.6 (19.8 to 30.2)	-2 (-20.1 to 19.3)	3210 (2570 to 3940)	639.2 (510.8 to 784.4)	-3.2 (-21.4 to 17.6)
Morocco	Both	5280 (3790 to 6810)	16.4 (11.7 to 21)	11.3 (-12.2 to 41.9)	5340 (3830 to 6880)	17 (12.2 to 21.7)	12.4 (-11.2 to 42.9)	143000 (102000 to 186000)	418.9 (302.6 to 542.1)	9.6 (-14.5 to 39.9)
	Male	4620 (3280 to 6060)	28.7 (20.4 to 37.4)	9.7 (-14.6 to 42.2)	4680 (3320 to 6100)	29.8 (21.3 to 38.8)	11.4 (-12.8 to 44.5)	125000 (89000 to 166000)	736.8 (528.3 to 963.9)	7.9 (-16.9 to 39.6)
	Female	656 (475 to 844)	4.2 (3 to 5.3)	17.3 (-6.6 to 46.8)	661 (480 to 845)	4.3 (3.2 to 5.5)	15.6 (-7.4 to 44.7)	17200 (12500 to 22500)	102.8 (74.5 to 132.8)	14.9 (-9.4 to 45.9)
Mozambique	Both	862 (643 to 1140)	8.3 (6.3 to 10.9)	6.5 (-13.3 to 31)	900 (674 to 1190)	9 (6.9 to 11.7)	6.4 (-12.9 to 30.3)	23500 (17200 to 31800)	203.9 (151.4 to 271.7)	6.1 (-14.4 to 31.4)
	Male	597 (457 to 782)	13.6 (10.6 to 17.6)	1.3 (-17.3 to 23.1)	624 (479 to 815)	14.9 (11.6 to 19.2)	1.2 (-17.2 to 22)	16100 (12200 to 21500)	324.2 (249.4 to 421.4)	0.7 (-18.5 to 23.8)
	Female	265 (169 to 398)	4.5 (2.9 to 6.6)	23.4 (-10.2 to 67)	276 (176 to 409)	4.8 (3.1 to 7.1)	23.4 (-10 to 67.2)	7340 (4620 to 11100)	111.1 (70 to 167.4)	23.3 (-12.6 to 68.5)
Myanmar	Both	10300 (7610 to 14100)	22.4 (16.8 to 30.2)	-0.2 (-17.6 to 21.8)	10600 (7900 to 14400)	23.8 (17.8 to 31.9)	-0.6 (-17.3 to 21.7)	272000 (200000 to 375000)	556.1 (409.2 to 761.9)	-2.5 (-19.9 to 20.6)
	Male	6980 (4960 to 10300)	35.4 (25.2 to 51.9)	-0.8 (-19.6 to 22.8)	7200 (5110 to 10600)	37.8 (27 to 55.3)	-1.2 (-19.2 to 22.3)	185000 (130000 to 275000)	865 (613.9 to 1281)	-3.1 (-21.9 to 23.1)
	Female	3310 (2560 to 4360)	12.8 (9.9 to 16.7)	4.8 (-12.8 to 27.1)	3420 (2640 to 4500)	13.5 (10.5 to 17.7)	4.9 (-12.7 to 28.7)	87000 (66600 to 116000)	318.7 (245.7 to 419.5)	2.3 (-16.6 to 26.3)
Namibia	Both	107 (83 to 135)	7.8 (6.2 to 9.7)	7.3 (-11 to 29.1)	111 (88 to 139)	8.3 (6.7 to 10.2)	6.9 (-10.4 to 27.8)	2760 (2090 to 3610)	189.3 (146.2 to 242.9)	5.6 (-13.8 to 29.4)
	Male	72 (56 to 90)	12.7 (10.1 to 15.5)	5 (-12.2 to 24)	75 (59 to 92)	13.7 (11 to 16.6)	4.6 (-12.1 to 23)	1880 (1440 to 2410)	304.7 (235.8 to 381.8)	3.3 (-15.3 to 24.7)
	Female	35 (24 to 48)	4.3 (3.1 to 6)	17 (-13.3 to 56.7)	36 (25 to 50)	4.6 (3.3 to 6.3)	16.6 (-12.8 to 55.1)	880 (598 to 1260)	105.3 (72.1 to 149.4)	14.8 (-16.5 to 57.6)
Nauru	Both	2 (1 to 2)	38.4 (25.3 to 50.7)	-1 (-22.2 to 21.4)	1 (1 to 2)	40.4 (27.1 to 52.8)	-1.5 (-22.7 to 20.1)	48 (28 to 67)	955.3 (605.6 to 1278.4)	-2.8 (-24 to 20.2)
	Male	1 (1 to 2)	58.5 (37.5 to 79.6)	0.3 (-21.4 to 25.5)	1 (1 to 1)	61.5 (40.2 to 83.2)	-0.1 (-21.3 to 24.6)	34 (19 to 49)	1458.5 (892.1 to 2036.2)	-1.9 (-24.4 to 24.1)
	Female	0 (0 to 1)	21.6 (13.6 to 31.5)	20.6 (-3.3 to 51)	0 (0 to 1)	22.5 (14.2 to 32.4)	19.7 (-4.1 to 49.9)	15 (8 to 22)	542 (329 to 797.6)	16.4 (-7.4 to 46.4)

Nepal	Both	1760 (1260 to 2280)	8 (5.9 to 10.4)	14 (-10.3 to 40.5)	1840 (1330 to 2370)	8.6 (6.3 to 11.2)	14.1 (-10.4 to 40.3)	45200 (32300 to 59200)	195.1 (139.5 to 253.5)	12.8 (-11.6 to 40.6)
	Male	1200 (873 to 1540)	11.5 (8.4 to 14.6)	13.4 (-12 to 39.1)	1260 (917 to 1610)	12.4 (9.1 to 15.7)	13.5 (-11.5 to 38.6)	30400 (21700 to 39500)	277.2 (198.2 to 357.2)	12.2 (-12.6 to 38.7)
	Female	557 (371 to 803)	4.8 (3.2 to 7)	21.9 (-4 to 57.4)	574 (382 to 830)	5.1 (3.4 to 7.6)	21.8 (-4.7 to 57.7)	14800 (9890 to 21000)	119.8 (80.4 to 171.5)	20.5 (-5.6 to 57.2)
Netherlands	Both	15200 (12000 to 19000)	44.8 (35.4 to 56.6)	-9.1 (-28.2 to 14.6)	13200 (12200 to 14000)	38.1 (35.4 to 40.5)	-10.5 (-14.9 to -5.6)	266000 (249000 to 282000)	825.4 (776.8 to 875.8)	-13.3 (-17.7 to -8.2)
	Male	8910 (6980 to 11200)	55.5 (43.4 to 70)	-16.9 (-34.8 to 5.5)	8010 (7420 to 8600)	49.8 (46.2 to 53.4)	-17.7 (-22.9 to -12.3)	155000 (145000 to 166000)	987.8 (923.7 to 1052.8)	-19.4 (-24.5 to -14.1)
	Female	6250 (4910 to 7840)	36.6 (28.8 to 46.1)	-0.1 (-21.7 to 26.7)	5160 (4680 to 5600)	29.1 (26.6 to 31.5)	-2.2 (-9.4 to 5.9)	110000 (101000 to 119000)	691 (634.4 to 746)	-6 (-13.2 to 2)
New Zealand	Both	2510 (2080 to 2950)	32 (26.4 to 37.8)	-4.3 (-20.4 to 13.2)	2000 (1860 to 2130)	25.3 (23.5 to 26.9)	-6.9 (-11.9 to -2.1)	41000 (38500 to 43300)	550.3 (519 to 580.4)	-7.6 (-12.6 to -2.5)
	Male	1310 (1010 to 1670)	35.6 (27.3 to 45.4)	-8.2 (-29.4 to 17.5)	1060 (979 to 1140)	28.5 (26.5 to 30.6)	-10.8 (-16.9 to -4.7)	21100 (19700 to 22500)	591.2 (552.2 to 629.2)	-10.9 (-16.7 to -5.1)
	Female	1190 (932 to 1490)	29 (22.6 to 36.4)	-0.7 (-22.3 to 25.2)	946 (859 to 1040)	22.7 (20.7 to 24.7)	-3.2 (-10.8 to 5)	19900 (18300 to 21600)	515.8 (476.2 to 558.3)	-4.3 (-11.7 to 4.3)
Nicaragua	Both	364 (295 to 442)	8.7 (7.1 to 10.4)	-1.8 (-18.2 to 17.7)	371 (303 to 444)	9.1 (7.5 to 10.8)	-4.5 (-19.9 to 13.6)	8450 (6830 to 10300)	188.2 (153.3 to 227.7)	-3.2 (-21.1 to 18.1)
	Male	217 (171 to 266)	11.8 (9.3 to 14.3)	-2.4 (-21.7 to 20.3)	227 (179 to 276)	12.8 (10.2 to 15.4)	-4.2 (-22.8 to 17.8)	5120 (3980 to 6350)	257.3 (201.5 to 315.3)	-3.4 (-23.2 to 20.6)
	Female	147 (121 to 177)	6.2 (5.2 to 7.5)	2.6 (-13.7 to 22.3)	144 (120 to 171)	6.3 (5.3 to 7.4)	-1.5 (-16.5 to 16.3)	3330 (2700 to 4040)	133.2 (109.6 to 160.1)	0.5 (-17.5 to 21.5)
Niger	Both	654 (415 to 950)	9.1 (5.8 to 13)	7.8 (-10.8 to 31.7)	682 (432 to 988)	9.9 (6.3 to 14.1)	7.3 (-11.2 to 30.1)	17400 (10800 to 25400)	214.9 (136.2 to 310.9)	8.6 (-11.9 to 35.6)
	Male	515 (317 to 789)	14.8 (9.2 to 22.5)	8.5 (-10.4 to 32.6)	540 (333 to 824)	16.3 (10.2 to 24.3)	8.1 (-10.6 to 32)	13600 (8400 to 21200)	348.7 (214.9 to 530.6)	8.9 (-11.9 to 36.6)
	Female	139 (86 to 219)	3.6 (2.3 to 5.6)	14.2 (-6.7 to 38.8)	142 (88 to 223)	3.9 (2.5 to 6.1)	13.7 (-6.2 to 37.5)	3850 (2340 to 6140)	88 (54.3 to 139.1)	14.7 (-6.8 to 41.8)
Nigeria	Both	5890 (4570 to 7550)	7.5 (5.9 to 9.5)	-2.2 (-27.2 to 33.7)	6240 (4780 to 8210)	8.3 (6.4 to 10.7)	-1.9 (-26.6 to 33.5)	151000 (113000 to 201000)	172.7 (131.6 to 228.6)	-2.7 (-28.7 to 34.4)
	Male	4030 (2940 to 5520)	10.9 (8.1 to 14.7)	-4 (-34.8 to 45.5)	4310 (3090 to 6120)	12 (8.8 to 16.7)	-4 (-33.7 to 43.5)	101000 (70300 to 147000)	250.3 (178.1 to 358)	-4.1 (-35.1 to 47.6)
	Female	1860 (1300 to 2540)	4.4 (3.1 to 5.7)	22.9 (-15.8 to 74.3)	1930 (1370 to 2610)	4.7 (3.4 to 6.2)	23 (-13.4 to 77.8)	49800 (34500 to 70200)	102.2 (72.1 to 139.2)	22.6 (-16.9 to 84.9)
Niue	Both	1 (1 to 1)	30.5 (24.6 to 37.5)	-0.2 (-12.5 to 14.7)	1 (1 to 1)	31.4 (25.4 to 38.5)	-0.8 (-13.2 to 13.9)	16 (12 to 19)	717.4 (565 to 897.6)	-0.8 (-14.3 to 14.7)

	Male	0 (0 to 1)	44.8 (37.2 to 52.7)	-1.5 (-13.2 to 13.1)	0 (0 to 1)	46.8 (39.1 to 55.1)	-1.9 (-13.4 to 13)	10 (8 to 13)	1036 (840 to 1244.3)	-2.3 (-14.9 to 13)
	Female	0 (0 to 0)	19.1 (13.8 to 25.8)	3.1 (-15.6 to 26.8)	0 (0 to 0)	19.6 (14.1 to 26.4)	2.5 (-15.9 to 24.3)	5 (4 to 7)	445.9 (315.2 to 603.1)	2.1 (-17.5 to 26.7)
North Macedonia	Both	1340 (1030 to 1700)	40.4 (31 to 51.2)	-4.2 (-26.8 to 21.4)	1280 (985 to 1630)	38.9 (30 to 49.1)	-4.9 (-27.1 to 20.2)	33100 (25200 to 42400)	1000.4 (760.5 to 1285.2)	-5.9 (-29.3 to 20.5)
	Male	1090 (819 to 1400)	67.5 (51.3 to 86.3)	-6.9 (-29.9 to 18.4)	1040 (787 to 1330)	65.5 (49.9 to 83.1)	-7.7 (-29.9 to 16.6)	26900 (20200 to 35000)	1651 (1240.1 to 2140.9)	-8.4 (-31.7 to 17.4)
	Female	254 (198 to 322)	15.3 (11.9 to 19.4)	2.7 (-20.6 to 30.8)	241 (189 to 304)	14.5 (11.4 to 18.3)	1.6 (-21 to 28.9)	6160 (4750 to 7920)	377.6 (290.8 to 487.5)	0.6 (-23.3 to 29.7)
Northern Mariana Islands	Both	22 (19 to 25)	44.8 (39 to 50)	-4.4 (-15.5 to 8.3)	22 (19 to 25)	46.2 (40.4 to 51.5)	-4.5 (-15.4 to 7.3)	572 (480 to 658)	1021.7 (877.2 to 1161.8)	-5.3 (-17.6 to 8.2)
	Male	17 (15 to 19)	68.7 (60.4 to 76.6)	-2.8 (-15.5 to 10.7)	17 (14 to 19)	71.6 (63.3 to 79.9)	-2.5 (-14.9 to 10.9)	436 (367 to 502)	1546.9 (1336.2 to 1746.2)	-4.3 (-17.2 to 10.5)
	Female	5 (4 to 7)	21.4 (17.3 to 26.5)	3.7 (-15.2 to 27)	5 (4 to 6)	21.7 (17.6 to 26.8)	3.4 (-15.4 to 26.5)	136 (107 to 171)	486.9 (390.7 to 609.4)	3.6 (-15.5 to 27.7)
Norway	Both	2960 (2520 to 3450)	31 (26.3 to 36.1)	-16 (-28.3 to -2.2)	2390 (2220 to 2520)	24.5 (22.9 to 25.9)	-14.3 (-17.8 to -11.2)	47300 (44400 to 49800)	519.5 (489.8 to 546.5)	-17.3 (-20.9 to -14.2)
	Male	1600 (1280 to 1970)	35.2 (28.2 to 43.4)	-23.3 (-38.5 to -5.5)	1300 (1220 to 1380)	28.6 (26.7 to 30.4)	-22.6 (-26.6 to -18.7)	25800 (24000 to 27300)	580.3 (540.3 to 615.9)	-25 (-29.3 to -21.2)
	Female	1360 (1090 to 1660)	27.6 (22.1 to 33.7)	-7.3 (-24.1 to 13.4)	1090 (986 to 1170)	21.3 (19.6 to 22.8)	-3.9 (-8.9 to 1.6)	21500 (20000 to 23000)	466.9 (435.4 to 497.1)	-7.3 (-12.4 to -1.7)
Oman	Both	147 (117 to 192)	9.9 (8.2 to 11.9)	-17.6 (-31.8 to 2.4)	144 (115 to 187)	10.4 (8.7 to 12.6)	-17.1 (-30.6 to 2)	3880 (3000 to 5250)	211.7 (170.7 to 267.8)	-23.1 (-37.7 to -0.4)
	Male	102 (76 to 145)	12.9 (10.2 to 16.6)	-20.5 (-37.1 to 3.8)	103 (77 to 143)	14.2 (11.4 to 18)	-19 (-35.5 to 3.9)	2760 (2020 to 4080)	274.3 (211.8 to 366.4)	-25.8 (-42.5 to 1.2)
	Female	45 (33 to 54)	6.7 (5.1 to 8.1)	-3.5 (-26.2 to 16.6)	42 (30 to 50)	6.6 (5 to 8)	-4.5 (-26.4 to 15.4)	1120 (800 to 1380)	141.8 (104.1 to 169.6)	-10.1 (-32.7 to 12)
Pakistan	Both	18400 (14000 to 24300)	16.4 (12.5 to 21.5)	-5.5 (-28.4 to 27.3)	18600 (14200 to 24000)	17.2 (13.1 to 22)	-5.8 (-28.3 to 25.3)	523000 (400000 to 681000)	425.7 (327.1 to 550.8)	-6.2 (-29.7 to 26.8)
	Male	15400 (11200 to 21000)	26.7 (19.5 to 36.1)	-7.2 (-33.8 to 29.4)	15600 (11400 to 20900)	27.9 (20.6 to 37.1)	-7.6 (-33.5 to 27.2)	437000 (318000 to 592000)	689.9 (504.5 to 928)	-8 (-35.1 to 30.4)
	Female	2980 (2160 to 3930)	5.5 (4 to 7.2)	11.4 (-16.8 to 50.5)	3000 (2200 to 3980)	5.8 (4.2 to 7.6)	11.3 (-15.5 to 49.1)	85700 (62100 to 114000)	141.8 (104.1 to 189)	10.4 (-17.7 to 50.9)
Palau	Both	9 (7 to 12)	44.5 (36 to 55)	1.1 (-14.8 to 22.5)	9 (7 to 11)	45.8 (37.3 to 56.3)	0.7 (-14.9 to 21.6)	246 (195 to 311)	1071.8 (855.7 to 1337.8)	0.4 (-15.4 to 22.1)
	Male	5 (4 to 6)	47.7 (38.9 to 58.7)	-1.6 (-17.8 to 18.8)	5 (4 to 6)	49.1 (40.2 to 60.2)	-2.1 (-18.1 to 17.4)	138 (109 to 176)	1166 (941.2 to 1464.6)	-2.2 (-19 to 18.9)
	Female	4 (3 to 5)	41.2 (31.6 to 51.6)	4.1 (-15.6 to 31.2)	4 (3 to 5)	42.4 (32.6 to 53.1)	3.6 (-16.1 to 29.4)	108 (82 to 138)	965 (738.5 to 1223.8)	3.2 (-16.6 to 30.6)

Palestine	Both	523 (444 to 613)	22.5 (19.1 to 26.1)	17 (-3.1 to 38.2)	530 (448 to 618)	23.8 (20.2 to 27.6)	16.2 (-4 to 37.7)	14200 (12000 to 16700)	546.4 (462.8 to 639.4)	16.3 (-4.1 to 38.2)
	Male	410 (349 to 478)	38 (32.5 to 44)	10.5 (-9.2 to 31.8)	420 (358 to 487)	41.2 (35.3 to 47.7)	10.1 (-9.3 to 31.2)	11200 (9470 to 13100)	901.5 (768 to 1046.3)	10.7 (-9.2 to 32.7)
	Female	113 (92 to 137)	9.1 (7.5 to 11)	27.5 (0.5 to 59.2)	110 (90 to 133)	9.3 (7.6 to 11.1)	25.7 (-0.6 to 57.4)	3040 (2490 to 3670)	219.7 (178.8 to 265)	23.1 (-2.7 to 54)
Panama	Both	447 (346 to 572)	10.9 (8.4 to 13.8)	0.4 (-22.1 to 28.5)	447 (347 to 570)	10.8 (8.4 to 13.8)	-2.1 (-23.8 to 24.8)	9760 (7430 to 12500)	235.8 (179.7 to 302.7)	-1.2 (-24.3 to 28.4)
	Male	279 (213 to 359)	14 (10.7 to 18)	-7.1 (-29.7 to 20.8)	287 (218 to 368)	14.5 (11 to 18.5)	-8.3 (-30.3 to 18.7)	6230 (4680 to 8130)	308.3 (232.4 to 402.3)	-7.3 (-30.7 to 22.5)
	Female	168 (131 to 215)	7.9 (6.1 to 10.1)	16.8 (-9.4 to 51.7)	160 (125 to 204)	7.4 (5.8 to 9.4)	11.9 (-12 to 44.7)	3530 (2740 to 4540)	166.9 (129.4 to 214.9)	12.7 (-13.6 to 47.7)
Papua New Guinea	Both	996 (694 to 1510)	21.8 (15.6 to 33)	3.6 (-12.9 to 25)	1000 (701 to 1520)	23.2 (16.6 to 34.8)	3.8 (-12.5 to 24.9)	28600 (19600 to 43900)	544.7 (383.5 to 826.8)	2.9 (-14.1 to 25.4)
	Male	809 (558 to 1270)	33.8 (23.9 to 52.3)	1.4 (-15.7 to 23.7)	811 (560 to 1270)	36.2 (26 to 55.5)	1.3 (-15.4 to 23.4)	23200 (15900 to 36500)	837.1 (580 to 1307.9)	1.3 (-16.8 to 24.8)
	Female	188 (117 to 299)	8.7 (5.4 to 13.9)	11.4 (-9.7 to 39)	189 (118 to 301)	9.1 (5.7 to 14.7)	11.4 (-9.3 to 38.1)	5410 (3380 to 8660)	218.2 (137.4 to 349.6)	10.5 (-12.2 to 39.6)
Paraguay	Both	863 (653 to 1110)	15.8 (12 to 20.3)	2 (-23.4 to 33.5)	881 (672 to 1130)	16.3 (12.5 to 20.9)	0.7 (-24.6 to 31.5)	20900 (15700 to 27000)	368.1 (277.1 to 475)	-0.1 (-26.2 to 31.8)
	Male	657 (499 to 853)	25.4 (19.4 to 32.9)	0.4 (-26.6 to 32.6)	670 (510 to 867)	26.4 (20.3 to 34.2)	-0.8 (-26.4 to 31.6)	16000 (11900 to 20900)	584.9 (440 to 765.1)	-1.6 (-28 to 31.1)
	Female	206 (155 to 268)	7.2 (5.4 to 9.3)	8.1 (-20.1 to 44.2)	211 (160 to 274)	7.4 (5.6 to 9.5)	6.4 (-21.4 to 41.4)	4890 (3640 to 6370)	166.2 (123.6 to 216.6)	6.3 (-22.3 to 42.8)
Peru	Both	3340 (2510 to 4400)	10.5 (7.9 to 13.8)	-10 (-36.6 to 26.3)	3470 (2610 to 4560)	10.9 (8.2 to 14.3)	-11.3 (-37.1 to 23.7)	76900 (57000 to 102000)	238.2 (176.8 to 316.6)	-12.2 (-38.8 to 23.8)
	Male	1770 (1280 to 2390)	11.6 (8.4 to 15.7)	-13.7 (-41.3 to 22.8)	1840 (1340 to 2500)	12.1 (8.8 to 16.4)	-14.9 (-41.9 to 20.8)	40700 (28800 to 55200)	261.5 (185.4 to 355.6)	-15.3 (-43.5 to 22.3)
	Female	1580 (1200 to 2040)	9.5 (7.2 to 12.3)	-5.3 (-32.2 to 29.9)	1630 (1240 to 2120)	9.8 (7.4 to 12.7)	-6.9 (-32.7 to 26.8)	36200 (27300 to 47800)	216.4 (163.9 to 285)	-8.4 (-35.1 to 28.7)
Philippines	Both	13800 (11000 to 17100)	17.7 (14.2 to 21.8)	2.1 (-19.1 to 26.6)	14000 (11300 to 17100)	18.5 (15.1 to 22.4)	2 (-17.8 to 24.4)	373000 (301000 to 459000)	443.6 (359.6 to 543.9)	1.3 (-18.9 to 24.5)
	Male	9520 (7210 to 12400)	26.6 (20.4 to 34.2)	-2.9 (-29.1 to 26.6)	9570 (7260 to 12300)	27.8 (21.3 to 35.4)	-2.9 (-26.1 to 25.4)	258000 (195000 to 335000)	658.2 (499.8 to 848.9)	-3.8 (-27.5 to 25.2)
	Female	4310 (3200 to 5620)	10.4 (7.8 to 13.4)	16.3 (-14.8 to 55.9)	4390 (3360 to 5630)	10.9 (8.4 to 13.9)	16.2 (-13.3 to 52.8)	115000 (86800 to 150000)	259.6 (197.5 to 336.2)	15.5 (-15.3 to 54.6)
Poland	Both	30000 (25200 to 35700)	43.1 (36 to 51.4)	-6.1 (-21.5 to 11.9)	31200 (26100 to 37000)	44.3 (37 to 52.6)	-5.6 (-20.9 to 12.4)	709000 (586000 to 847000)	1051.7 (866.5 to 1258.5)	-8.4 (-24.3 to 9.9)

	Male	20900 (16300 to 26300)	69.5 (54.3 to 87.3)	-12.1 (-31 to 9.9)	21800 (17100 to 27100)	72.9 (57.3 to 90.6)	-11.9 (-30.2 to 9.5)	499000 (389000 to 627000)	1656 (1292 to 2080.6)	-14.2 (-32.9 to 8)
	Female	9130 (7110 to 11500)	23.4 (18.1 to 29.7)	6.4 (-15.6 to 34.7)	9450 (7500 to 11900)	23.6 (18.6 to 29.9)	7.7 (-14.5 to 35.7)	210000 (165000 to 267000)	571.1 (448.2 to 729.4)	3.9 (-18 to 32.7)
Portugal	Both	4680 (3660 to 5920)	21.4 (16.5 to 27.3)	-10.7 (-30.9 to 14.2)	4710 (4370 to 5020)	20.7 (19.3 to 22.1)	-9.4 (-15.4 to -3.4)	102000 (94600 to 108000)	505.6 (470.9 to 539.4)	-12.1 (-18.7 to -5.7)
	Male	3550 (2770 to 4490)	36.2 (28.1 to 46.1)	-14.4 (-33.3 to 10)	3560 (3320 to 3810)	35.6 (33.2 to 38)	-12.9 (-19.3 to -6.5)	78000 (72700 to 83200)	844.5 (785.3 to 902.6)	-15.7 (-22.2 to -9.2)
	Female	1130 (889 to 1430)	9.2 (7.1 to 11.8)	3.4 (-20.3 to 31.6)	1150 (1020 to 1260)	8.9 (8 to 9.6)	3.5 (-5.8 to 13.3)	23600 (21400 to 25600)	219.2 (200 to 238.2)	2.8 (-6.6 to 13.3)
Puerto Rico	Both	881 (690 to 1110)	12.4 (9.7 to 15.7)	-8.7 (-28.7 to 15.9)	845 (663 to 1060)	11.5 (9 to 14.6)	-10.3 (-29.3 to 13.5)	16500 (12900 to 21100)	252.7 (196.3 to 324)	-9.6 (-30.1 to 16)
	Male	542 (421 to 689)	17.3 (13.4 to 22.1)	-12.6 (-32.1 to 13)	508 (396 to 643)	15.9 (12.4 to 20.2)	-14.4 (-33.8 to 10.4)	10200 (7890 to 13200)	347.4 (267 to 451.3)	-13.3 (-33.9 to 13.2)
	Female	339 (264 to 428)	8.5 (6.6 to 10.8)	-1.9 (-23.3 to 24.5)	336 (261 to 420)	8 (6.3 to 10.1)	-3.5 (-24.2 to 21.8)	6290 (4930 to 7980)	174.6 (135.1 to 222.9)	-3.3 (-25.8 to 23.5)
Qatar	Both	125 (89 to 174)	18.8 (14.4 to 23.9)	-14.3 (-34.8 to 10.5)	119 (85 to 165)	20.7 (16 to 26.1)	-12.9 (-32.5 to 10.8)	3500 (2440 to 4860)	373.9 (279.2 to 491.3)	-18.3 (-39 to 8.4)
	Male	103 (71 to 149)	21.2 (16 to 27.6)	-19.5 (-40.6 to 9.4)	101 (70 to 144)	23.8 (18.1 to 30.4)	-17.8 (-38.6 to 9.9)	2930 (1990 to 4180)	426.9 (315.7 to 573)	-23.1 (-45.1 to 5.6)
	Female	21 (16 to 27)	11.8 (9.2 to 14.7)	-3.9 (-23.4 to 17.4)	18 (14 to 23)	11.8 (9.3 to 14.7)	-5.9 (-24.5 to 15.7)	572 (421 to 734)	220.5 (170.7 to 275.2)	-8.6 (-26.9 to 13.4)
South Korea	Both	31800 (26500 to 37500)	35.6 (29.6 to 41.9)	-3.5 (-18.9 to 14.1)	23300 (20900 to 25600)	26.3 (23.5 to 28.9)	-7.7 (-15.1 to -0.1)	448000 (407000 to 490000)	498.4 (452.3 to 545)	-12.9 (-20 to -5.3)
	Male	22700 (18600 to 27100)	59.1 (48.6 to 70.4)	-9.4 (-25.4 to 8.1)	16600 (14800 to 18400)	44.7 (39.5 to 49.7)	-13.5 (-21.9 to -4.2)	330000 (296000 to 365000)	812.1 (726.9 to 896.9)	-18 (-25.9 to -8.8)
	Female	9170 (7490 to 10900)	18.3 (15 to 21.8)	2.7 (-13.7 to 21.2)	6690 (5680 to 7580)	13.1 (11.2 to 14.9)	-1.8 (-11.8 to 8.7)	118000 (104000 to 131000)	245.2 (216.8 to 270.4)	-6.8 (-15.3 to 1.6)
Moldova	Both	1070 (920 to 1230)	18.3 (15.7 to 21)	-16.8 (-29.7 to -3.5)	1030 (892 to 1190)	17.6 (15.2 to 20.2)	-17.6 (-30 to -4.6)	27400 (23500 to 31600)	477.4 (409.1 to 549.8)	-20.7 (-33.6 to -7.3)
	Male	842 (718 to 973)	34 (28.9 to 39.3)	-18.7 (-32.1 to -5)	811 (693 to 936)	33 (28.2 to 37.9)	-19.3 (-32.5 to -5.9)	22000 (18600 to 25600)	883.1 (746.8 to 1022)	-22.5 (-36 to -9.2)
	Female	226 (185 to 275)	6.7 (5.5 to 8.2)	-6.1 (-25.6 to 16.7)	222 (182 to 268)	6.5 (5.3 to 7.9)	-7.5 (-26.6 to 14.6)	5360 (4350 to 6560)	168 (135.4 to 205.9)	-9.7 (-29.4 to 13.6)
Romania	Both	11500 (9480 to 14000)	33.2 (27.2 to 40.5)	-0.2 (-18.2 to 20.3)	11000 (9120 to 13400)	31.2 (25.7 to 37.8)	-1.6 (-19 to 19.1)	273000 (224000 to 333000)	826.9 (675.5 to 1012.4)	-4.2 (-21.6 to 16.6)

	Male	8890 (7320 to 10800)	56.9 (46.8 to 69.6)	-4.4 (-21.2 to 16.1)	8440 (6960 to 10300)	53.7 (44.2 to 65.6)	-5.5 (-21.9 to 14.5)	213000 (174000 to 261000)	1395.6 (1137.7 to 1711.5)	-8.5 (-25.2 to 11.9)
	Female	2650 (2170 to 3190)	13.7 (11.1 to 16.5)	16.4 (-5.1 to 41)	2580 (2130 to 3100)	12.8 (10.5 to 15.5)	14.1 (-6.7 to 37.7)	59800 (48600 to 72300)	337.1 (271.8 to 410.7)	12.8 (-8.7 to 37.6)
Russia	Both	58200 (49700 to 67800)	24.5 (21 to 28.6)	-13.9 (-26.1 to -0.1)	54100 (46100 to 63100)	22.8 (19.4 to 26.6)	-15.2 (-27.7 to -1.3)	1350000 (1140000 to 1580000)	578.7 (489.4 to 679.6)	-17.2 (-30 to -2.8)
	Male	46000 (37800 to 55600)	49.8 (41.1 to 60.1)	-19.7 (-33.8 to -3.7)	42800 (35300 to 51200)	47.4 (39.4 to 56.6)	-20.6 (-34.3 to -5.4)	1090000 (891000 to 1310000)	1150.4 (944.7 to 1379.6)	-22.2 (-35.9 to -6.5)
	Female	12200 (10100 to 14800)	8.5 (7 to 10.4)	5.6 (-14.1 to 30.2)	11300 (9370 to 13500)	7.7 (6.4 to 9.3)	2.2 (-16.8 to 24)	258000 (210000 to 313000)	190 (153.4 to 232.9)	0.9 (-19.6 to 24.3)
Rwanda	Both	472 (326 to 685)	8.2 (5.7 to 11.7)	9.7 (-8.9 to 32.4)	488 (336 to 710)	8.8 (6.2 to 12.6)	9.7 (-8.6 to 32)	12700 (8710 to 18700)	199.5 (137.4 to 288.9)	9 (-11 to 32.8)
	Male	312 (189 to 487)	13.4 (8.3 to 20.7)	4.7 (-15.2 to 25.4)	324 (197 to 504)	14.6 (9.2 to 22.5)	4.8 (-14.5 to 24.9)	8360 (5060 to 13300)	317.5 (193.7 to 492.7)	3.9 (-16.7 to 25.9)
	Female	160 (120 to 206)	4.7 (3.6 to 6)	18.8 (-3.3 to 46.1)	165 (124 to 211)	5 (3.8 to 6.4)	18.9 (-2.7 to 45.6)	4360 (3210 to 5690)	117 (87 to 150.9)	17.7 (-5.9 to 45.7)
Saint Kitts and Nevis	Both	8 (6 to 9)	11.4 (9.7 to 13.4)	15.1 (-1 to 35.1)	7 (6 to 9)	11.2 (9.6 to 13.1)	12.7 (-2.3 to 31.6)	183 (148 to 223)	256.4 (210.1 to 309.3)	14.5 (-4.4 to 35.5)
	Male	5 (4 to 6)	15.7 (13.4 to 18.4)	16.7 (-0.7 to 36.9)	5 (4 to 6)	15.3 (13.1 to 17.7)	11.8 (-4.1 to 30.5)	123 (98 to 147)	347.5 (283.1 to 412.2)	18.3 (-2.9 to 41.7)
	Female	3 (2 to 3)	7.5 (6 to 9.2)	4.9 (-14.2 to 29.2)	3 (2 to 3)	7.6 (6.2 to 9.2)	5.5 (-11.9 to 27.5)	61 (48 to 76)	169.7 (133 to 209.7)	3.2 (-16.5 to 27.1)
Saint Lucia	Both	26 (22 to 31)	12.2 (10.4 to 14.3)	16 (-2.4 to 37.3)	26 (23 to 31)	12.4 (10.6 to 14.5)	16.1 (-2 to 36.5)	636 (535 to 757)	289.7 (243.7 to 342.9)	13.4 (-5.6 to 34.7)
	Male	18 (15 to 21)	17.8 (15 to 21.1)	11.8 (-6.2 to 34.5)	18 (15 to 21)	18.1 (15.4 to 21.4)	11.4 (-6.2 to 32.9)	433 (358 to 520)	411 (342.9 to 491.6)	9.9 (-9.1 to 33.2)
	Female	8 (7 to 10)	7.4 (6.2 to 8.9)	23.5 (1.5 to 50.1)	9 (7 to 10)	7.6 (6.3 to 9.1)	23.9 (2.3 to 50.2)	203 (168 to 245)	178.8 (147.7 to 215.5)	19.8 (-2.4 to 46.2)
Saint Vincent and the Grenadines	Both	14 (12 to 16)	10.2 (8.9 to 11.7)	11.6 (-3.7 to 29.1)	14 (12 to 16)	10.5 (9.2 to 12)	11.4 (-3.6 to 28.6)	341 (295 to 396)	247.3 (214.2 to 287)	10.5 (-5.9 to 30)
	Male	9 (8 to 10)	12.9 (11.2 to 15)	8 (-7.8 to 27.5)	9 (8 to 11)	13.3 (11.5 to 15.4)	7.3 (-8.1 to 25.7)	222 (190 to 261)	310.5 (266.2 to 363.8)	7.7 (-8.9 to 28.6)
	Female	5 (4 to 6)	7.4 (6.2 to 8.6)	13.5 (-6 to 36.2)	5 (4 to 6)	7.7 (6.5 to 8.9)	13.4 (-5.9 to 35.6)	119 (100 to 140)	179.8 (150.2 to 211.9)	12.7 (-8.2 to 36.5)
Samoa	Both	16 (13 to 21)	11.2 (8.7 to 13.9)	1.6 (-12.7 to 19.1)	16 (13 to 21)	11.6 (9.1 to 14.4)	1.4 (-12.7 to 18.5)	427 (329 to 556)	280 (216.3 to 358.6)	0.9 (-14.5 to 20)
	Male	11 (8 to 14)	15.4 (11.1 to 19.8)	-2.3 (-16.5 to 14.1)	11 (8 to 14)	16 (11.6 to 20.5)	-2.4 (-16.4 to 13.4)	289 (206 to 388)	379.7 (273.9 to 498.7)	-2.9 (-18.2 to 15)

	Female	5 (4 to 7)	7.3 (5.5 to 9.5)	7.7 (-14.1 to 37)	5 (4 to 7)	7.5 (5.7 to 9.9)	7.7 (-14.2 to 36.7)	139 (101 to 184)	182.4 (136.3 to 240.4)	7.3 (-14.7 to 36.5)
San Marino	Both	23 (17 to 30)	36.8 (27.7 to 48.6)	-0.7 (-20.4 to 23)	21 (14 to 29)	32.5 (21.2 to 47)	-3.2 (-29.4 to 31.8)	417 (268 to 614)	731.1 (463.2 to 1090)	-2.7 (-30.2 to 34.2)
	Male	16 (12 to 21)	55.1 (41.6 to 71.4)	-3.3 (-22.8 to 20.2)	15 (10 to 21)	49.1 (31.9 to 69.1)	-5.7 (-31.4 to 26.7)	294 (187 to 423)	1056.9 (665 to 1536.7)	-4.4 (-31.5 to 30.7)
	Female	6 (5 to 9)	20.3 (14.8 to 28.8)	3.3 (-21.4 to 34.3)	6 (4 to 9)	17.9 (11.5 to 28)	-0.2 (-30.5 to 43.4)	123 (78 to 193)	426.6 (266.8 to 673.1)	0.2 (-31.5 to 45.1)
São Tome and Príncipe	Both	19 (15 to 24)	18.9 (14.9 to 23.4)	11.3 (-6.3 to 31)	20 (15 to 24)	20.3 (16 to 25.1)	11 (-6.5 to 30.7)	504 (384 to 643)	452.2 (350.1 to 568.7)	10.5 (-8.2 to 29.9)
	Male	13 (11 to 16)	27.9 (22.9 to 34)	8 (-10.5 to 29)	14 (11 to 17)	30.4 (25 to 37)	7.8 (-10.2 to 28.3)	340 (268 to 431)	652.8 (526 to 804.8)	7.1 (-11.7 to 28.9)
	Female	6 (3 to 8)	10.8 (6.2 to 15.5)	19.3 (-5.6 to 44.6)	6 (3 to 9)	11.5 (6.5 to 16.6)	19.2 (-5.7 to 44.2)	164 (94 to 238)	268.5 (153.1 to 386.1)	17.4 (-7.7 to 43)
Saudi Arabia	Both	1550 (1190 to 1900)	8.8 (7 to 10.6)	-3.9 (-22.3 to 15.7)	1490 (1150 to 1830)	9.1 (7.3 to 11.1)	-5.5 (-22.8 to 13.2)	45500 (34500 to 57200)	208 (162.6 to 253.2)	-5.3 (-23.8 to 15.2)
	Male	1120 (841 to 1390)	10.9 (8.5 to 13.7)	-9.9 (-28.5 to 11.1)	1110 (842 to 1380)	11.7 (9.1 to 14.5)	-10.8 (-28.8 to 9.8)	32800 (24500 to 41900)	260.5 (199.9 to 325)	-9.9 (-28.8 to 12)
	Female	429 (321 to 551)	5.5 (4.2 to 6.9)	7.4 (-17.2 to 35)	382 (286 to 493)	5.3 (4.1 to 6.7)	2.9 (-20.5 to 29)	12700 (9340 to 16500)	127.2 (95.8 to 162.8)	3.2 (-20.3 to 31.2)
Senegal	Both	875 (662 to 1140)	12.2 (9.4 to 15.8)	9.6 (-11.1 to 34.7)	921 (701 to 1200)	13.2 (10.2 to 17.2)	9.2 (-11.1 to 34.6)	22500 (16600 to 29900)	291 (218.1 to 382.1)	9.4 (-13.4 to 35)
	Male	655 (485 to 891)	19.1 (14.2 to 25.8)	6.7 (-14.8 to 32.8)	691 (513 to 942)	20.8 (15.7 to 28.2)	6.5 (-14.2 to 32.3)	16800 (12100 to 23000)	452.4 (332.6 to 617.3)	6.5 (-16.1 to 34.8)
	Female	220 (167 to 291)	5.8 (4.5 to 7.6)	22 (-1.3 to 52.6)	230 (176 to 302)	6.3 (4.9 to 8.1)	21.5 (-2 to 51.4)	5790 (4280 to 7810)	140.7 (106.2 to 187.4)	21.9 (-3.2 to 54.2)
Serbia	Both	7700 (6060 to 9690)	49.4 (38.8 to 62.4)	-3.5 (-24.3 to 21.5)	7260 (5730 to 9070)	46 (36.1 to 57.5)	-4.3 (-24.8 to 19.8)	177000 (138000 to 223000)	1183.5 (919.2 to 1497.6)	-6.2 (-27.1 to 18.9)
	Male	5500 (4290 to 6930)	75.3 (58.5 to 95)	-7.7 (-28 to 16.2)	5250 (4110 to 6570)	71.5 (56 to 89.7)	-7.9 (-28 to 15.3)	128000 (99600 to 163000)	1799.3 (1392.8 to 2300)	-10 (-30 to 14.4)
	Female	2200 (1720 to 2790)	26.9 (21.1 to 34.3)	6.9 (-16.6 to 37.1)	2010 (1580 to 2530)	24.1 (18.9 to 30.5)	5 (-18.1 to 33.5)	48700 (37700 to 62300)	634.4 (488.5 to 818.6)	3.8 (-19.8 to 34)
Seychelles	Both	19 (17 to 22)	17.7 (15.4 to 20.3)	4.2 (-8.9 to 20.3)	19 (17 to 22)	18.4 (16 to 21.2)	3.4 (-9.5 to 18.8)	477 (414 to 554)	416.9 (363.4 to 483.4)	3.2 (-9.8 to 20.1)
	Male	14 (12 to 17)	28.6 (24.4 to 33.7)	-1.7 (-16.4 to 16.2)	15 (12 to 17)	30.1 (25.7 to 35.5)	-2.8 (-17.2 to 14.5)	367 (311 to 444)	661.4 (561.6 to 790.9)	-1.9 (-17.1 to 18.1)
	Female	5 (4 to 6)	8.3 (6.8 to 9.9)	9.7 (-11 to 32.7)	5 (4 to 6)	8.8 (7.2 to 10.5)	8.5 (-11.7 to 31.4)	109 (89 to 131)	189.3 (155.7 to 228.2)	8.4 (-12.4 to 31.3)
Sierra Leone	Both	361 (257 to 483)	10.5 (7.5 to 13.9)	6.7 (-13.4 to 34.1)	378 (271 to 501)	11.4 (8.1 to 15.1)	6 (-13.2 to 32.5)	9510 (6690 to 13000)	253.8 (180.1 to 343.3)	6.6 (-14.6 to 35.1)

	Male	270 (183 to 370)	16 (10.9 to 21.8)	1.7 (-18.3 to 29.2)	285 (192 to 389)	17.5 (11.9 to 23.8)	1.2 (-18.3 to 27.6)	7040 (4730 to 9780)	380.4 (259.3 to 522.8)	1.3 (-19.8 to 30.4)
	Female	90 (68 to 118)	5.1 (3.9 to 6.7)	18.8 (-3.9 to 47.9)	93 (71 to 122)	5.5 (4.1 to 7.1)	18.2 (-4.4 to 46.4)	2470 (1800 to 3250)	126.2 (93.9 to 166.8)	18.6 (-5.5 to 49.1)
Singapore	Both	2160 (1720 to 2710)	28.4 (22.7 to 35.4)	-14.5 (-31 to 6)	1570 (1410 to 1680)	20.9 (18.7 to 22.5)	-18.5 (-23.8 to -13.3)	32000 (29500 to 34300)	406.2 (373.2 to 435)	-20 (-25.1 to -14.8)
	Male	1370 (1080 to 1720)	38.7 (30.8 to 48.3)	-20.1 (-36.8 to 0.3)	1010 (915 to 1090)	29.4 (26.4 to 31.7)	-23.4 (-30 to -17.6)	20900 (19000 to 22400)	549.3 (499.2 to 590.6)	-25 (-31.6 to -19.2)
	Female	790 (627 to 991)	19.7 (15.6 to 24.8)	-7.3 (-25 to 15.1)	556 (476 to 621)	13.9 (11.9 to 15.5)	-12.4 (-21.1 to -3.2)	11100 (9750 to 12400)	277 (242.9 to 308.5)	-13.1 (-21.6 to -3.7)
Slovakia	Both	3130 (2430 to 4050)	33.6 (26.1 to 43.4)	-5.3 (-26.6 to 21.7)	2530 (1980 to 3280)	27 (21 to 35)	-8.9 (-29.2 to 17.4)	59400 (45600 to 77500)	649.9 (497.9 to 844.8)	-9.8 (-30.6 to 17.1)
	Male	2370 (1800 to 3100)	59.2 (45.3 to 77.2)	-9 (-30.2 to 17.3)	1920 (1470 to 2520)	48.2 (37.2 to 63)	-12.8 (-32.8 to 13.1)	45600 (34700 to 59700)	1110.8 (845.1 to 1445.9)	-13.4 (-34 to 13.6)
	Female	759 (596 to 954)	14.6 (11.4 to 18.4)	-1.5 (-24.1 to 26.6)	609 (483 to 764)	11.6 (9.1 to 14.5)	-4.2 (-25.7 to 21.7)	13800 (10600 to 17500)	281.9 (216.5 to 358.4)	-5.4 (-27.8 to 21.7)
Slovenia	Both	1400 (1080 to 1820)	33.9 (26 to 44.2)	-10.3 (-30.8 to 16.1)	1270 (984 to 1640)	29.9 (23 to 38.9)	-10.4 (-30.1 to 15)	27700 (21200 to 36000)	705.3 (539.2 to 919.5)	-12.3 (-32.6 to 14.7)
	Male	971 (749 to 1260)	51.4 (39.6 to 66.8)	-16.1 (-35.2 to 7.7)	889 (685 to 1160)	46.9 (36.2 to 61)	-16.2 (-34.7 to 7)	19700 (15000 to 25900)	1054.1 (801.6 to 1395)	-17.4 (-36.7 to 6.5)
	Female	424 (324 to 555)	19.2 (14.6 to 25.3)	-2 (-26.6 to 29.2)	380 (290 to 498)	16.3 (12.4 to 21.4)	-3.2 (-26.7 to 27.1)	8010 (6020 to 10400)	396.8 (295.8 to 521.2)	-3.7 (-28.1 to 28.1)
Solomon Islands	Both	102 (53 to 167)	32.7 (18.6 to 51.4)	7 (-10.6 to 31)	101 (54 to 163)	34.1 (19.8 to 53)	6.5 (-11 to 29.1)	3090 (1550 to 5150)	859.2 (460.5 to 1392.4)	6.3 (-11.8 to 31.1)
	Male	83 (40 to 145)	52.3 (28 to 88.6)	5.9 (-11.9 to 30.1)	82 (40 to 142)	54.6 (29.9 to 91.7)	5.6 (-11.7 to 28.7)	2510 (1180 to 4430)	1374.8 (684 to 2378.4)	5 (-13 to 30.2)
	Female	19 (12 to 29)	12.3 (8.3 to 17.9)	24.1 (0.6 to 52.8)	19 (12 to 28)	12.8 (8.9 to 18.6)	23.6 (0.2 to 51)	574 (359 to 871)	324.5 (211.7 to 481.7)	22.3 (-1.8 to 51.2)
Somalia	Both	365 (205 to 607)	5.6 (3.1 to 9.1)	-4.9 (-23.4 to 17.8)	377 (215 to 620)	6 (3.4 to 9.8)	-4.8 (-22.9 to 17.6)	10800 (6110 to 18300)	146.2 (83 to 240.5)	-5.3 (-24.5 to 17.3)
	Male	291 (159 to 502)	10.9 (6.1 to 18.4)	-5.9 (-23.9 to 15.2)	301 (165 to 514)	11.8 (6.5 to 19.8)	-5.7 (-23.1 to 15)	8620 (4720 to 15100)	276.7 (152.9 to 474.8)	-6.5 (-24.6 to 15.8)
	Female	74 (40 to 126)	1.9 (1.1 to 3.4)	2 (-22 to 29.2)	77 (42 to 133)	2.1 (1.1 to 3.6)	2.1 (-21 to 27.5)	2230 (1220 to 3920)	52.3 (28.5 to 90.4)	1.3 (-22.7 to 28.1)
South Africa	Both	8520 (7630 to 9700)	19.1 (17.2 to 21.6)	-12.6 (-21.9 to -0.1)	8690 (7810 to 9910)	20 (18 to 22.6)	-12.6 (-21.8 to -0.7)	222000 (198000 to 256000)	471.6 (421.8 to 542.9)	-15.9 (-25.6 to -3.1)
	Male	5830 (5110 to 6810)	31 (27.5 to 36)	-15.8 (-26.2 to -1.4)	5870 (5160 to 6850)	32.4 (28.8 to 37.4)	-16.2 (-25.9 to -1.9)	157000 (137000 to 185000)	767.8 (672.1 to 901.5)	-18.8 (-29.1 to -3.3)
	Female	2690 (2370 to 3070)	10.6 (9.4 to 12.1)	-6.9 (-18 to 6.6)	2830 (2490 to 3200)	11.3 (10 to 12.8)	-6.4 (-17 to 6.5)	64800 (56300 to 74700)	247.3 (215.7 to 284.6)	-10.6 (-22.4 to 3.9)

South Sudan	Both	318 (208 to 454)	8.8 (5.9 to 12.5)	-4.1 (-21 to 16.7)	334 (216 to 480)	9.6 (6.4 to 13.6)	-4.5 (-21.8 to 16.6)	8640 (5500 to 12700)	215 (138.9 to 309)	-4.8 (-23.3 to 17.9)
	Male	247 (150 to 355)	13.1 (8.1 to 18.6)	-4.1 (-22 to 17.6)	261 (158 to 376)	14.4 (8.9 to 20.5)	-4.5 (-22.7 to 17.4)	6570 (3860 to 9670)	315.6 (189.7 to 457.2)	-4.9 (-23.7 to 17.4)
	Female	71 (46 to 131)	3.9 (2.6 to 7.1)	8.2 (-13 to 35.9)	73 (47 to 133)	4.2 (2.8 to 7.6)	8.2 (-13.6 to 37.9)	2070 (1270 to 3780)	99.9 (63.6 to 182.7)	8 (-15.1 to 41.4)
Spain	Both	29500 (23100 to 37500)	33.2 (25.9 to 42.5)	-7.2 (-27.5 to 18.8)	24500 (22800 to 26000)	26.7 (25 to 28.2)	-7.7 (-12.7 to -2.7)	524000 (490000 to 553000)	633.5 (594.8 to 668.8)	-10 (-15.2 to -5.1)
	Male	23600 (18500 to 30100)	57.5 (44.9 to 73.6)	-11.7 (-31.5 to 13.5)	19500 (18000 to 20700)	46.7 (43.2 to 49.5)	-12.6 (-18.2 to -7.2)	411000 (382000 to 436000)	1045.2 (973.1 to 1110.9)	-14.7 (-20.5 to -9.2)
	Female	5930 (4560 to 7640)	12.9 (9.9 to 16.8)	11.6 (-14.4 to 44.7)	5060 (4480 to 5580)	10.4 (9.3 to 11.5)	12 (1.8 to 23.3)	114000 (101000 to 125000)	273.2 (245.5 to 301.4)	8.9 (-1.8 to 20.9)
Sri Lanka	Both	2510 (1820 to 3410)	9.7 (7.1 to 13.1)	-3.9 (-29.3 to 29.5)	2480 (1800 to 3370)	9.8 (7.2 to 13.2)	-5.7 (-30.2 to 26.5)	61100 (44100 to 83500)	231.4 (167.5 to 315.6)	-6 (-31.8 to 26.8)
	Male	1730 (1200 to 2450)	15 (10.5 to 20.9)	-8.4 (-34.4 to 28.8)	1700 (1180 to 2410)	15.2 (10.7 to 21)	-10 (-35.6 to 25.2)	42900 (29700 to 60600)	356 (248.4 to 498.9)	-10.4 (-37 to 25.3)
	Female	775 (571 to 1030)	5.5 (4.1 to 7.3)	10.8 (-18.6 to 44.4)	774 (570 to 1020)	5.6 (4.1 to 7.3)	8.4 (-19.8 to 40.4)	18300 (13300 to 24300)	128.6 (94.2 to 170.8)	7.9 (-21.2 to 42.8)
Sudan	Both	1530 (983 to 2360)	8.3 (5.4 to 12.9)	3.4 (-16.5 to 29.6)	1550 (1020 to 2390)	8.8 (5.9 to 13.7)	3.3 (-16.2 to 29.7)	41400 (26800 to 64400)	203.8 (131.7 to 318.1)	1.9 (-18.4 to 29.5)
	Male	1120 (683 to 1920)	11.4 (6.9 to 19.6)	-2 (-22.5 to 26)	1160 (703 to 1980)	12.2 (7.5 to 20.9)	-1.9 (-22 to 26.4)	29800 (17900 to 51300)	279.4 (168.4 to 479.8)	-3.3 (-23.7 to 26.9)
	Female	403 (279 to 552)	4.5 (3.2 to 5.9)	24.6 (-5.4 to 60.3)	394 (279 to 537)	4.6 (3.3 to 6.1)	23.7 (-5.6 to 58.7)	11600 (7770 to 16700)	112.6 (79 to 155.1)	22.3 (-7.6 to 58.9)
Suriname	Both	91 (75 to 110)	15.1 (12.5 to 18.2)	7.9 (-11.3 to 30.8)	92 (76 to 111)	15.5 (12.8 to 18.6)	7.3 (-12.2 to 29.5)	2310 (1880 to 2810)	370.5 (302 to 450.4)	7.4 (-12.1 to 31.3)
	Male	59 (48 to 73)	21.4 (17.4 to 26.5)	5.2 (-15.2 to 29.9)	60 (48 to 74)	22 (17.9 to 27.2)	4.5 (-15.5 to 28.8)	1510 (1210 to 1870)	515.6 (416.4 to 636.6)	4.3 (-16.2 to 30.1)
	Female	32 (26 to 39)	9.9 (7.9 to 12)	15.2 (-8 to 42.5)	33 (26 to 40)	10.2 (8.2 to 12.4)	14.7 (-8.5 to 41.7)	801 (641 to 984)	244 (195.1 to 297.4)	15.1 (-9.6 to 43.9)
Sweden	Both	4340 (3700 to 5020)	20.8 (17.7 to 24.2)	-6 (-19.8 to 8.9)	4340 (4010 to 4590)	20 (18.7 to 21.2)	-6.9 (-11.5 to -2.5)	81100 (76300 to 85500)	416.1 (392.9 to 437.3)	-10.9 (-15.2 to -6.6)
	Male	2110 (1690 to 2590)	20.9 (16.8 to 25.8)	-13.5 (-30.2 to 8.2)	2160 (2010 to 2310)	21 (19.6 to 22.4)	-13.9 (-19 to -8.7)	39900 (37400 to 42400)	417.4 (392.5 to 442.2)	-16.8 (-21.6 to -11.7)
	Female	2240 (1810 to 2710)	20.9 (16.9 to 25.3)	1.1 (-17.4 to 23.1)	2180 (1950 to 2340)	19.4 (17.8 to 20.8)	-0.2 (-5.9 to 5.7)	41200 (37900 to 44100)	418.5 (389.3 to 447.2)	-5.3 (-10.7 to 0.5)
Switzerland	Both	4370 (3410 to 5520)	26.2 (20.3 to 33.1)	-14.8 (-33.9 to 8.3)	3830 (3480 to 4100)	22.2 (20.5 to 23.7)	-15.1 (-19.9 to -10.1)	77900 (72300 to 82800)	494.7 (460.5 to 525.5)	-17.7 (-22.5 to -12.9)
	Male	2640 (2060 to 3350)	33.6 (26.1 to 42.8)	-20 (-38.9 to 2.5)	2370 (2170 to 2570)	29.8 (27.3 to 32.1)	-20.4 (-26.2 to -14.5)	47900 (44200 to 51600)	631.5 (582.5 to 678.9)	-22.3 (-28.2 to -16.4)

	Female	1730 (1350 to 2200)	19.9 (15.4 to 25.4)	-8.2 (-28.3 to 17.1)	1460 (1300 to 1600)	16.1 (14.5 to 17.5)	-8 (-15.6 to -0.1)	30000 (27300 to 32600)	373.1 (341.6 to 404.1)	-11.3 (-18.6 to -3.7)
Syria	Both	1370 (1010 to 1810)	11.1 (8.2 to 14.5)	-5.3 (-28.1 to 22.1)	1370 (1010 to 1810)	11.6 (8.7 to 15.1)	-5.8 (-27.6 to 20.5)	37000 (26900 to 49500)	274.4 (203 to 363.5)	-5.4 (-29.3 to 24)
	Male	991 (720 to 1310)	15.4 (11.3 to 20.1)	-6.8 (-31.4 to 23.1)	1010 (735 to 1330)	16.2 (11.9 to 21)	-6.9 (-31 to 22.2)	26800 (19300 to 36100)	389.3 (283.4 to 517.1)	-6.9 (-32.3 to 25.1)
	Female	382 (262 to 519)	6.6 (4.6 to 8.8)	3.3 (-25.5 to 35.8)	368 (251 to 496)	6.8 (4.8 to 8.9)	1.9 (-26 to 32.7)	10100 (6960 to 14000)	155.3 (106.3 to 210.2)	4 (-25.2 to 39.6)
Taiwan (Province of China)	Both	12600 (9960 to 16200)	31.9 (25.2 to 40.9)	-7.1 (-27 to 18)	12100 (9530 to 15300)	30.3 (24.1 to 38.6)	-4.4 (-24 to 21.2)	250000 (195000 to 323000)	638.7 (500 to 824.7)	-3.7 (-24.9 to 24.1)
	Male	7800 (6140 to 9890)	42.6 (33.5 to 54.2)	-7.4 (-27.5 to 18.7)	7530 (5920 to 9580)	41.3 (32.5 to 52.5)	-4.7 (-25.2 to 21.1)	156000 (122000 to 204000)	847.3 (665.2 to 1101.7)	-4.7 (-26.4 to 23.8)
	Female	4820 (3750 to 6190)	22.8 (17.8 to 29.3)	-2.4 (-22.7 to 24.6)	4530 (3520 to 5770)	21.2 (16.5 to 27.1)	1.3 (-19.7 to 28.7)	93900 (73500 to 121000)	457.2 (355.9 to 592)	1.2 (-21 to 30.9)
Tajikistan	Both	593 (479 to 740)	12 (9.8 to 14.8)	-6.9 (-26.8 to 17.4)	586 (476 to 732)	12.6 (10.3 to 15.5)	-5.6 (-25.5 to 18.7)	17300 (13800 to 21800)	300.3 (243.9 to 374)	-10.4 (-29.7 to 13.9)
	Male	411 (323 to 527)	17.8 (14.3 to 22.4)	-9 (-29.5 to 16.4)	408 (322 to 522)	18.8 (15.2 to 23.5)	-7.3 (-28 to 17.7)	11800 (9320 to 15200)	436.7 (347 to 555.2)	-13.7 (-33.7 to 11)
	Female	182 (146 to 229)	6.8 (5.5 to 8.5)	4.7 (-17.8 to 32.3)	178 (143 to 224)	7.1 (5.7 to 8.8)	5.1 (-16.9 to 31.8)	5470 (4330 to 6890)	175.3 (141 to 219.8)	3.4 (-19.4 to 32.9)
Thailand	Both	22500 (17000 to 29600)	22.2 (16.8 to 29)	-9.5 (-32.8 to 20.5)	23100 (17500 to 30100)	23 (17.4 to 29.8)	-10.4 (-33.2 to 18.1)	524000 (390000 to 699000)	512.9 (382.7 to 681.9)	-10.9 (-34.6 to 20.2)
	Male	14700 (10800 to 19400)	32 (23.7 to 42.1)	-10.2 (-34.4 to 22.8)	15000 (11000 to 19700)	33.1 (24.7 to 43.5)	-11.1 (-34.7 to 20.8)	348000 (254000 to 466000)	735.8 (538.4 to 977.9)	-11.4 (-36.1 to 22.1)
	Female	7850 (5970 to 10300)	14.2 (10.8 to 18.6)	-8.2 (-32.6 to 21.9)	8130 (6110 to 10600)	14.7 (11 to 19.1)	-9.3 (-32.7 to 19.9)	176000 (131000 to 235000)	321.7 (238.8 to 427.9)	-10.1 (-35.6 to 20.9)
Timor-Leste	Both	158 (113 to 207)	19.5 (13.9 to 25.3)	12.2 (-12 to 38.9)	165 (116 to 216)	20.9 (15 to 27.1)	11.1 (-12.5 to 36.8)	4100 (2890 to 5400)	479.5 (341.3 to 629.5)	13.9 (-11.2 to 44.9)
	Male	113 (75 to 148)	28.1 (18.7 to 36.5)	11.6 (-12.5 to 41.1)	118 (78 to 154)	30.3 (20 to 39.3)	10.6 (-13.5 to 39.3)	2890 (1900 to 3900)	682.8 (456.4 to 904.2)	12.7 (-12.8 to 45.4)
	Female	46 (32 to 66)	11.1 (7.9 to 16)	17.4 (-10.8 to 49.3)	47 (33 to 67)	11.8 (8.4 to 16.8)	16.6 (-11.3 to 47.5)	1210 (840 to 1740)	280.2 (196.8 to 400.2)	19.1 (-10.1 to 54)
Togo	Both	392 (287 to 523)	11.2 (8.3 to 14.7)	0.2 (-17.6 to 21.7)	400 (294 to 530)	11.9 (9 to 15.6)	0.4 (-17.1 to 21.5)	10800 (7720 to 14500)	273.5 (201.1 to 363.3)	-0.3 (-19.4 to 22.5)
	Male	275 (194 to 381)	19.1 (13.7 to 25.9)	-3.9 (-20.8 to 16.9)	279 (197 to 386)	20.5 (14.8 to 27.7)	-3.3 (-20 to 17.4)	7570 (5220 to 10600)	454.2 (322.1 to 625.5)	-5 (-22.9 to 17.2)
	Female	118 (84 to 152)	5.9 (4.3 to 7.5)	15.9 (-8.1 to 44.8)	121 (86 to 156)	6.3 (4.6 to 8)	15.8 (-6.7 to 43.9)	3220 (2280 to 4220)	142.6 (101.7 to 184.9)	15 (-10.3 to 46.1)

Tokelau	Both	0 (0 to 0)	24.4 (19.2 to 31.4)	3.4 (-13 to 23.8)	0 (0 to 0)	25.7 (20.3 to 33.1)	3.1 (-13.4 to 22.9)	8 (6 to 10)	582.9 (449 to 762.1)	1.9 (-15 to 23.6)
	Male	0 (0 to 0)	31 (23.4 to 42.7)	-3.1 (-19.6 to 16.9)	0 (0 to 0)	32.9 (25 to 45.2)	-3.5 (-19.7 to 15.9)	5 (4 to 7)	725.7 (531.4 to 1023.9)	-3.9 (-21.1 to 17.2)
	Female	0 (0 to 0)	17.9 (12 to 25.1)	10.1 (-11.1 to 36.8)	0 (0 to 0)	18.7 (12.7 to 26.2)	9.6 (-11.7 to 36)	3 (2 to 4)	439.9 (288.9 to 619.1)	8.6 (-13.5 to 37.1)
Tonga	Both	22 (18 to 26)	27.8 (22.8 to 33.5)	0.1 (-15 to 18.4)	23 (19 to 27)	29.5 (24.3 to 35.4)	-0.2 (-14.9 to 18.4)	528 (425 to 647)	664.1 (536.7 to 811.6)	-0.4 (-16.5 to 18.6)
	Male	17 (13 to 20)	46.6 (38 to 56.8)	-2.3 (-16.9 to 16.8)	17 (14 to 21)	50.2 (41.1 to 60.7)	-2.7 (-16.8 to 15.3)	406 (323 to 501)	1090.1 (877.9 to 1348.2)	-2.8 (-18.5 to 16.9)
	Female	5 (4 to 7)	12.1 (9.1 to 15.8)	11.6 (-12.7 to 40.8)	5 (4 to 7)	12.8 (9.7 to 16.6)	11.4 (-12.7 to 40.3)	122 (91 to 160)	291.6 (218.2 to 380.9)	10.8 (-13.3 to 41.9)
Trinidad and Tobago	Both	192 (145 to 250)	10.3 (7.8 to 13.4)	-7.6 (-31.4 to 20.5)	191 (144 to 248)	10.3 (7.8 to 13.4)	-7.9 (-31.1 to 20.3)	4740 (3530 to 6240)	253.4 (189 to 333.3)	-8.6 (-32.9 to 21)
	Male	144 (107 to 190)	16 (11.9 to 21)	-9.6 (-33 to 19)	141 (105 to 186)	15.9 (11.8 to 20.8)	-9.9 (-32.5 to 18.2)	3580 (2640 to 4790)	389.9 (288.6 to 518.4)	-10.5 (-34.7 to 19.8)
	Female	48 (36 to 63)	5 (3.7 to 6.6)	-1.6 (-27 to 29.8)	50 (37 to 65)	5.1 (3.8 to 6.7)	-2.1 (-27 to 28.1)	1160 (835 to 1540)	122.5 (88.4 to 164.5)	-2.4 (-28.2 to 31.1)
Tunisia	Both	2460 (1740 to 3390)	19.4 (13.9 to 26.5)	-1.1 (-25.3 to 31.6)	2520 (1800 to 3460)	20.2 (14.4 to 27.6)	-2 (-25.8 to 29.6)	61900 (43100 to 86300)	471.8 (328.9 to 654.3)	-2.5 (-27.5 to 30.7)
	Male	2200 (1530 to 3060)	35.5 (24.9 to 49.2)	-1.6 (-26.1 to 31.9)	2270 (1580 to 3150)	37.5 (26.5 to 51.5)	-2.1 (-26.3 to 29.8)	55600 (38400 to 78300)	865.2 (600.5 to 1209.8)	-3 (-28.2 to 31.4)
	Female	266 (193 to 358)	4.1 (3 to 5.5)	10.1 (-16.3 to 46.1)	257 (186 to 344)	4 (2.9 to 5.4)	6.9 (-18.4 to 41)	6240 (4480 to 8410)	94.1 (67.5 to 126.6)	6.6 (-19.5 to 43.7)
Turkey	Both	29500 (23400 to 36800)	33.1 (26.2 to 41.1)	-3.6 (-25.2 to 25.2)	29800 (23800 to 37000)	33.8 (26.9 to 41.8)	-4.3 (-25.4 to 23.3)	744000 (585000 to 929000)	814.6 (643.8 to 1017)	-6.2 (-27.4 to 22.7)
	Male	24300 (19200 to 30400)	57.7 (45.6 to 72)	-6.5 (-27.7 to 21.9)	24800 (19600 to 31000)	59.7 (47.3 to 74.1)	-6.7 (-27.7 to 20.8)	626000 (493000 to 787000)	1431.9 (1132.2 to 1796.1)	-8.5 (-29.8 to 20)
	Female	5190 (4130 to 6460)	11.1 (8.9 to 13.8)	11.7 (-12.7 to 43.1)	5050 (4030 to 6270)	10.9 (8.7 to 13.5)	8.6 (-15 to 38.5)	118000 (93400 to 147000)	250.3 (198.4 to 311.9)	7.3 (-17 to 39.9)
Turkmenistan	Both	413 (326 to 521)	9.9 (7.8 to 12.4)	16.3 (-7.6 to 45.7)	399 (316 to 504)	9.9 (7.8 to 12.2)	15.7 (-7.9 to 44.6)	11900 (9360 to 15100)	265.8 (209.7 to 336.1)	14.6 (-9.5 to 45.4)
	Male	311 (242 to 395)	16.6 (13.2 to 20.9)	14.7 (-9.9 to 44)	300 (234 to 380)	16.7 (13.3 to 20.9)	14.1 (-9.8 to 43)	9040 (7010 to 11500)	438.6 (343.7 to 552.6)	13.1 (-11.8 to 44.3)
	Female	102 (79 to 130)	4.6 (3.6 to 5.8)	18.5 (-6.4 to 48.4)	99 (77 to 126)	4.6 (3.6 to 5.7)	17.4 (-6.8 to 47.1)	2880 (2200 to 3720)	122.6 (94.5 to 158.1)	17.1 (-8.8 to 47.9)
Tuvalu	Both	3 (2 to 4)	26 (19.4 to 35)	1.6 (-16.5 to 22.4)	3 (2 to 4)	27.5 (20.7 to 36.8)	1.5 (-16.8 to 22.2)	69 (50 to 95)	645.3 (471.1 to 887.8)	1.3 (-17.4 to 22.3)
	Male	2 (1 to 3)	38.6 (28.8 to 54.8)	-2 (-19.2 to 18.2)	2 (1 to 3)	41.2 (30.6 to 58.1)	-2.1 (-19 to 17.6)	48 (34 to 69)	948 (686.2 to 1361.3)	-2.3 (-20.3 to 19.1)

	Female	1 (1 to 1)	15.2 (10.7 to 20.6)	6.2 (-15.2 to 31.1)	1 (1 to 1)	16 (11.4 to 21.6)	6.3 (-15.5 to 31.2)	21 (15 to 29)	375.2 (263.6 to 520.2)	5.3 (-16.6 to 30.8)
Uganda	Both	1010 (801 to 1210)	7.3 (6 to 8.7)	6.2 (-12.1 to 25.3)	1040 (828 to 1240)	7.8 (6.4 to 9.2)	6.6 (-11.3 to 25.1)	27800 (21800 to 34000)	182.5 (144.7 to 219.9)	6.2 (-13.3 to 27.8)
	Male	626 (491 to 765)	10.8 (8.6 to 12.8)	-1 (-19 to 18.3)	646 (510 to 785)	11.6 (9.3 to 13.8)	-0.3 (-17.9 to 18.6)	17200 (13200 to 21600)	260.6 (204.9 to 317.7)	-1 (-20.2 to 21)
	Female	381 (287 to 486)	4.9 (3.7 to 6.2)	23.4 (-5.3 to 57.4)	390 (295 to 498)	5.1 (3.9 to 6.5)	23.1 (-4.9 to 57)	10600 (7860 to 13600)	122.6 (91.5 to 156.9)	23.6 (-7.1 to 58.9)
Ukraine	Both	20100 (16500 to 24400)	27.2 (22.4 to 33.1)	13.9 (-6.6 to 37.4)	17000 (14100 to 20200)	22.8 (18.9 to 27)	11.7 (-8 to 33.5)	452000 (371000 to 539000)	631.2 (518.4 to 755.2)	15.3 (-6.1 to 39.1)
	Male	16800 (13200 to 20900)	56.9 (44.9 to 70.8)	12.6 (-12 to 39.5)	14200 (11300 to 17400)	48.2 (38.5 to 58.8)	10.5 (-12.2 to 36)	383000 (304000 to 469000)	1289.6 (1025.2 to 1581)	14.3 (-10 to 42.8)
	Female	3320 (2630 to 4180)	7.4 (5.8 to 9.3)	10.7 (-13.3 to 39.6)	2850 (2290 to 3550)	6.2 (4.9 to 7.8)	8.3 (-13.9 to 36)	69100 (54700 to 86600)	168.3 (131.7 to 213.1)	11.7 (-13 to 42.9)
United Arab Emirates	Both	542 (393 to 721)	18.6 (13.5 to 25.7)	-18.5 (-37 to 6.3)	523 (380 to 697)	20.3 (14.7 to 28.2)	-19.5 (-37.9 to 5.9)	16700 (12100 to 22200)	406.5 (295.1 to 559.1)	-17.3 (-36.4 to 7.1)
	Male	434 (310 to 593)	21.5 (15.8 to 29.9)	-15 (-33.3 to 8.9)	423 (303 to 580)	23.7 (17.4 to 32.9)	-15.4 (-33.5 to 8.7)	13400 (9530 to 18300)	468.7 (343.4 to 645.7)	-14.7 (-33.2 to 9.1)
	Female	108 (72 to 145)	12.8 (7.5 to 19.3)	-30.6 (-57.7 to 9.6)	100 (66 to 135)	13.6 (7.9 to 20.6)	-33.3 (-59.9 to 7.7)	3290 (2250 to 4370)	274.9 (164.7 to 396.5)	-28.4 (-54.3 to 7.8)
United Kingdom	Both	51500 (43200 to 60700)	40.2 (33.5 to 47.3)	-3.7 (-19 to 13.3)	42800 (39900 to 44800)	32.8 (30.8 to 34.3)	-4.9 (-7.5 to -2.3)	796000 (756000 to 831000)	666.4 (636.1 to 693.5)	-7.1 (-9.7 to -4.5)
	Male	27800 (21900 to 34700)	47 (36.9 to 58.7)	-8.2 (-27.1 to 15)	23300 (22000 to 24200)	39.2 (37 to 40.8)	-9.2 (-11.4 to -7.1)	438000 (419000 to 453000)	773.6 (743.3 to 800.4)	-10.8 (-13 to -8.6)
	Female	23700 (19000 to 29600)	34.6 (27.6 to 43.6)	0.7 (-19.1 to 26.6)	19600 (17800 to 20900)	27.7 (25.5 to 29.5)	-0.7 (-5.4 to 4)	358000 (333000 to 381000)	572.6 (538 to 607.2)	-3 (-7.6 to 1.7)
Tanzania	Both	2130 (1530 to 3070)	9 (6.6 to 12.8)	6.5 (-12 to 27.1)	2220 (1610 to 3190)	9.7 (7.1 to 13.6)	6.6 (-11.4 to 26.9)	56900 (40200 to 83500)	221 (158.4 to 320.9)	5.7 (-14.8 to 27.7)
	Male	1530 (1040 to 2380)	13.7 (9.5 to 20.9)	1.1 (-18 to 22)	1600 (1090 to 2470)	14.9 (10.4 to 22.4)	1.2 (-17.9 to 21.9)	40300 (26900 to 63400)	331.1 (225.3 to 513.5)	0.5 (-20.2 to 23.1)
	Female	605 (483 to 737)	4.8 (3.8 to 5.7)	17.8 (-3.7 to 44.6)	622 (499 to 754)	5 (4.1 to 6)	17.7 (-3.2 to 43.5)	16600 (12800 to 20900)	119.7 (94.7 to 146.8)	17.3 (-6.2 to 46.4)
Virgin Islands	Both	45 (38 to 52)	24.1 (20.1 to 27.8)	-4.8 (-18.5 to 8.3)	45 (38 to 52)	24 (20.3 to 27.6)	-4.7 (-17.7 to 8)	1010 (837 to 1180)	552.5 (453.9 to 647.2)	-4.7 (-19 to 9.5)
	Male	29 (24 to 35)	35.1 (29 to 41.5)	-4.6 (-18.4 to 11.9)	29 (24 to 34)	34.8 (29.1 to 40.7)	-4.6 (-18.2 to 10.8)	669 (537 to 801)	808.2 (649.4 to 973.6)	-4.2 (-19.4 to 12.9)
	Female	16 (13 to 19)	15.2 (12.5 to 18.2)	-2.2 (-19.6 to 15.1)	16 (13 to 19)	15.5 (12.7 to 18.4)	-2 (-19.5 to 14.6)	341 (278 to 413)	339.9 (273.3 to 413.5)	-2.6 (-21 to 17.2)

United States of America	Both	255000 (221000 to 294000)	45.1 (39.1 to 52.2)	-10.3 (-22 to 3.3)	206000 (194000 to 214000)	36.1 (34.1 to 37.5)	-9.6 (-12.1 to -7.3)	4190000 (4000000 to 4330000)	767.4 (735.2 to 792)	-11 (-13.6 to -8.5)
	Male	138000 (112000 to 167000)	53.4 (43.6 to 64.7)	-11.8 (-27.5 to 6.8)	114000 (108000 to 118000)	44.1 (42.1 to 45.7)	-11.2 (-13.2 to -8.4)	2350000 (2270000 to 2420000)	922.2 (890.9 to 951.8)	-12.2 (-14.2 to -9.3)
	Female	117000 (95900 to 141000)	38.3 (31.3 to 46.4)	-9.2 (-25.3 to 9.8)	92600 (84700 to 98900)	29.6 (27.3 to 31.5)	-8.5 (-13.1 to -4.3)	1840000 (1720000 to 1950000)	633.8 (595.3 to 669.9)	-9.8 (-14.4 to -5.4)
Uruguay	Both	1750 (1380 to 2190)	34.1 (26.6 to 42.9)	-4.4 (-25.8 to 20.3)	1750 (1640 to 1860)	33.5 (31.3 to 35.5)	-5.1 (-11.4 to 1)	39500 (36800 to 42000)	813.1 (755.2 to 866.3)	-6.3 (-13.2 to 0.6)
	Male	1290 (1000 to 1610)	57.7 (45 to 72.4)	-10 (-30.3 to 13)	1290 (1200 to 1380)	57.4 (53.5 to 61.2)	-10.6 (-17.2 to -4.2)	29400 (27300 to 31400)	1344.7 (1252 to 1437)	-11.6 (-18.2 to -5.1)
	Female	461 (365 to 581)	16 (12.6 to 20.2)	15.2 (-9.9 to 47.1)	463 (417 to 507)	15.4 (14 to 16.8)	13.8 (3.5 to 25.1)	10200 (9290 to 11100)	383.4 (350.8 to 419.6)	12 (1.3 to 22.5)
Uzbekistan	Both	2770 (2280 to 3320)	12.7 (10.7 to 14.9)	7.7 (-9.7 to 27)	2660 (2190 to 3180)	12.9 (11 to 15.2)	6.9 (-9.9 to 25.6)	80600 (66300 to 96800)	320.9 (266.5 to 380.7)	6.8 (-11.2 to 27.1)
	Male	1990 (1620 to 2420)	20.5 (17 to 24.3)	4.2 (-13.8 to 24.1)	1910 (1560 to 2320)	21 (17.5 to 24.9)	3.2 (-14.5 to 22.7)	57800 (47000 to 70200)	510.3 (421.6 to 610.4)	4 (-14.8 to 25.6)
	Female	779 (638 to 942)	6.7 (5.6 to 8)	15.8 (-4 to 39.2)	744 (612 to 898)	6.9 (5.7 to 8.1)	14.3 (-5 to 36.8)	22700 (18500 to 27600)	169.5 (140.4 to 203.1)	14.9 (-5.7 to 38.9)
Vanuatu	Both	42 (28 to 63)	24.7 (16.4 to 36.3)	4.1 (-11.4 to 24.7)	43 (29 to 64)	26.4 (17.5 to 38.6)	3.8 (-11.8 to 24.2)	1130 (741 to 1700)	615.5 (407 to 925.7)	4.1 (-12.2 to 26.3)
	Male	34 (20 to 53)	38.1 (23 to 59.2)	2.2 (-12.6 to 22.3)	35 (21 to 54)	40.7 (24.6 to 62.8)	2 (-12.7 to 21.8)	911 (549 to 1460)	948.7 (572.2 to 1517.7)	2 (-13.4 to 23.2)
	Female	8 (5 to 12)	10 (6.6 to 14.3)	12.1 (-9.8 to 38.9)	8 (5 to 12)	10.7 (7.1 to 15.3)	11.8 (-9.7 to 38.9)	220 (138 to 320)	250.8 (161.3 to 362.7)	11.6 (-10.7 to 39.2)
Venezuela	Both	5770 (4260 to 7450)	19.8 (14.7 to 25.4)	-1.9 (-26.3 to 25.9)	5660 (4210 to 7250)	19.6 (14.7 to 25.1)	-2.5 (-26.6 to 25.2)	138000 (102000 to 180000)	460.1 (338.3 to 594.7)	-2.9 (-27.7 to 26.2)
	Male	3500 (2590 to 4520)	25.7 (19.2 to 33.1)	-5.8 (-29.8 to 22.3)	3520 (2620 to 4550)	26.3 (19.7 to 33.9)	-6.2 (-29.8 to 21.6)	86700 (63500 to 113000)	609.3 (450.4 to 790.9)	-6.1 (-30.8 to 22.9)
	Female	2270 (1630 to 3110)	14.6 (10.6 to 20)	3.5 (-25.2 to 41.2)	2140 (1550 to 2930)	13.9 (10.1 to 18.9)	2.7 (-25.6 to 39.7)	51500 (36800 to 71100)	327.2 (233.8 to 452.3)	2 (-26.9 to 41.3)
Vietnam	Both	25600 (19700 to 32400)	26.7 (20.8 to 33.5)	7.6 (-13 to 29.7)	25200 (19500 to 31700)	27 (21.1 to 33.6)	6.2 (-13.7 to 27.6)	677000 (515000 to 874000)	668.8 (512 to 853.2)	4.6 (-16.1 to 27.7)
	Male	18500 (14100 to 23700)	44.8 (34.8 to 56.2)	5 (-15.2 to 27.6)	18200 (13900 to 23000)	45.8 (35.8 to 57)	3.9 (-15.6 to 25)	499000 (375000 to 644000)	1102.4 (838 to 1402.6)	2.1 (-18.8 to 26)
	Female	7020 (5270 to 9140)	13.3 (10 to 17.2)	12.5 (-12 to 40.6)	7010 (5290 to 9120)	13.5 (10.2 to 17.4)	10.9 (-12.6 to 37.9)	178000 (133000 to 235000)	325.3 (245.2 to 426.6)	8.9 (-15.3 to 36.8)

Yemen	Both	1300 (885 to 1930)	9.9 (6.8 to 14.7)	-1.8 (-21.3 to 21.5)	1340 (912 to 1970)	10.6 (7.3 to 15.6)	-1.8 (-21.4 to 21)	36200 (24400 to 53800)	248.8 (169.3 to 368.9)	-1.9 (-21.3 to 21.9)
	Male	1010 (658 to 1600)	15.9 (10.4 to 24.9)	-3.1 (-23.8 to 21.4)	1050 (681 to 1640)	17.1 (11.2 to 26.7)	-3.2 (-23.7 to 21.3)	27700 (18000 to 44300)	393.9 (254.6 to 622.7)	-2.9 (-23.8 to 22)
	Female	289 (212 to 391)	4.2 (3.1 to 5.6)	8.6 (-20.7 to 36)	289 (214 to 390)	4.3 (3.3 to 5.8)	8.2 (-21.4 to 35)	8460 (5990 to 11600)	107.4 (78.7 to 145.4)	8.7 (-19.9 to 38.2)
Zambia	Both	699 (500 to 911)	10.7 (7.9 to 13.8)	0.9 (-20.1 to 27.1)	717 (517 to 929)	11.4 (8.5 to 14.6)	0 (-19.5 to 24.7)	19700 (14000 to 26100)	270 (192.9 to 353.1)	0.1 (-22 to 27)
	Male	484 (316 to 659)	16 (10.8 to 21.4)	-3.2 (-24.3 to 24.8)	499 (329 to 675)	17.2 (11.8 to 22.7)	-3.9 (-24.5 to 22.6)	13500 (8830 to 18800)	394.9 (259.9 to 535.2)	-4.7 (-26.9 to 24.4)
	Female	215 (153 to 294)	6.1 (4.4 to 8.3)	17.5 (-9.2 to 52.3)	218 (156 to 297)	6.5 (4.8 to 8.8)	17.2 (-9.7 to 52.1)	6220 (4270 to 8620)	157.8 (111.2 to 215.9)	15.8 (-12.3 to 52.2)
Zimbabwe	Both	1020 (786 to 1250)	14.5 (11.3 to 17.5)	0.7 (-16.7 to 19.5)	1030 (792 to 1250)	15.1 (11.8 to 18.2)	1.6 (-15.8 to 20.7)	28800 (21800 to 35900)	372.2 (284.7 to 455.6)	0.6 (-18.3 to 21.4)
	Male	572 (451 to 699)	19.3 (15.6 to 23.2)	0.3 (-19.2 to 22.2)	581 (458 to 706)	20.4 (16.5 to 24.3)	-1.6 (-20.3 to 19.6)	16700 (12900 to 20900)	504.3 (396.1 to 614.6)	-2.9 (-23 to 20.1)
	Female	452 (321 to 591)	11.2 (7.9 to 14.5)	2.8 (-22.1 to 35.6)	445 (313 to 584)	11.4 (8.1 to 14.8)	8.2 (-18.4 to 44.2)	12100 (8470 to 15900)	275.5 (192.9 to 367.2)	7.8 (-19.5 to 45.3)

Table S2. Incidence, deaths, and disability-adjusted life-years (DALYs) in 2019 (counts and age-standardised rates) and percent change in age-standardised rates between 2010 and 2019, at global, regional, national levels, and Socio-demographic Index (SDI) quintiles, by sex and for both sexes combined, for larynx cancer.

95% UIs given in parentheses.

Location	Sex	Incidence			Deaths			DALYs		
		Number in 2019	Age-standardised rate per 100,000 in 2019	Percent change in age-standardised rate from 2010 to 2019 (%)	Number in 2019	Age-standardised rate per 100,000 in 2019	Percent change in age-standardised rate from 2010 to 2019 (%)	Number in 2019	Age-standardised rate per 100,000 in 2019	Percent change in age-standardised rate from 2010 to 2019 (%)
Global	Both	209000 (194000 to 225000)	2.5 (2.3 to 2.7)	-2.5 (-9.3 to 4.8)	123000 (115000 to 133000)	1.5 (1.4 to 1.6)	-8.5 (-14.3 to -2.3)	3260000 (3030000 to 3510000)	38.8 (36.1 to 41.8)	-9.3 (-15.3 to -2.9)
	Male	181000 (166000 to 196000)	4.6 (4.2 to 5)	-3 (-10.5 to 5)	106000 (97800 to 115000)	2.7 (2.5 to 3)	-9.3 (-15.9 to -2.8)	2800000 (2590000 to 3030000)	69.3 (64.1 to 75.2)	-10 (-16.9 to -3.2)
	Female	28500 (26100 to 31300)	0.7 (0.6 to 0.7)	-0.5 (-8.4 to 8.1)	17800 (16200 to 19700)	0.4 (0.4 to 0.5)	-4.9 (-12.8 to 4.1)	464000 (421000 to 512000)	10.7 (9.7 to 11.8)	-5.2 (-13.3 to 4)
High SDI	Both	43700 (39300 to 48500)	2.5 (2.2 to 2.8)	-5.5 (-15 to 5)	14600 (13700 to 15300)	0.8 (0.7 to 0.8)	-8.9 (-11.8 to -5.6)	342000 (324000 to 359000)	20.1 (19.1 to 21.2)	-10.3 (-13.6 to -6.5)
	Male	37500 (33500 to 41900)	4.5 (4 to 5)	-6.9 (-16.9 to 4.1)	12400 (11700 to 12900)	1.5 (1.4 to 1.5)	-10.7 (-14.1 to -7.2)	290000 (274000 to 305000)	35.6 (33.7 to 37.5)	-11.8 (-15.4 to -7.7)
	Female	6230 (5390 to 7140)	0.7 (0.6 to 0.8)	-4.4 (-14.9 to 7.2)	2280 (2030 to 2490)	0.2 (0.2 to 0.2)	-7.5 (-11.1 to -2.9)	52100 (47300 to 56800)	6 (5.5 to 6.5)	-7.8 (-11.8 to -2.9)
High-middle SDI	Both	57800 (52500 to 63500)	2.8 (2.5 to 3.1)	-7.8 (-16.1 to 1.2)	30400 (28200 to 32500)	1.5 (1.4 to 1.6)	-16.9 (-22.7 to -11.4)	780000 (723000 to 836000)	38 (35.2 to 40.7)	-18.3 (-24.2 to -12.5)
	Male	51900 (46800 to 57200)	5.4 (4.9 to 6)	-9.3 (-17.9 to 0)	27200 (25100 to 29200)	2.9 (2.7 to 3.2)	-18.4 (-24.5 to -12.7)	703000 (649000 to 756000)	72.9 (67.4 to 78.4)	-19.5 (-25.7 to -13.5)
	Female	5990 (5360 to 6730)	0.5 (0.5 to 0.6)	-3.3 (-12.8 to 8.8)	3250 (2940 to 3600)	0.3 (0.3 to 0.3)	-11.9 (-19.5 to -2.8)	77800 (70800 to 85900)	7.3 (6.6 to 8)	-12.9 (-20.6 to -4)

Middle SDI	Both	51400 (46600 to 56800)	2 (1.8 to 2.2)	3.7 (-6 to 14.6)	34900 (31600 to 38200)	1.4 (1.3 to 1.6)	-7.8 (-15.7 to 0.2)	912000 (829000 to 1000000)	34.7 (31.5 to 38.1)	-8.5 (-16.5 to -0.3)
	Male	43200 (38700 to 48300)	3.5 (3.2 to 3.9)	5.2 (-5.8 to 18.4)	29500 (26500 to 32600)	2.5 (2.3 to 2.8)	-6.5 (-15.6 to 2.8)	777000 (699000 to 860000)	61.1 (55.2 to 67.5)	-7.2 (-16.7 to 2.3)
	Female	8260 (7280 to 9360)	0.6 (0.6 to 0.7)	0.5 (-11.3 to 12.4)	5380 (4820 to 6070)	0.4 (0.4 to 0.5)	-10.2 (-19.8 to 0.1)	135000 (121000 to 151000)	10.1 (9.1 to 11.3)	-10.9 (-20.4 to -0.6)
Low-middle SDI	Both	37600 (33500 to 42100)	2.7 (2.4 to 3)	0.5 (-10.3 to 13.1)	32100 (28700 to 36200)	2.3 (2.1 to 2.6)	-4.5 (-14.8 to 7)	900000 (801000 to 1020000)	61.3 (54.6 to 69.4)	-5.2 (-16 to 7.3)
	Male	31700 (27800 to 36100)	4.7 (4.1 to 5.3)	0.8 (-11.8 to 15.2)	27100 (23800 to 31000)	4.1 (3.7 to 4.7)	-4 (-15.8 to 9)	759000 (662000 to 868000)	106.8 (93.4 to 122)	-5.1 (-17.5 to 8.8)
	Female	5890 (5110 to 6810)	0.8 (0.7 to 0.9)	5.6 (-7.3 to 22.5)	4970 (4260 to 5780)	0.7 (0.6 to 0.8)	0.2 (-12.5 to 15.6)	141000 (120000 to 165000)	18.6 (15.8 to 21.7)	0.4 (-13 to 16.8)
Low SDI	Both	11200 (9760 to 12700)	2.1 (1.8 to 2.3)	-4.5 (-15.9 to 8.7)	11200 (9790 to 12800)	2.2 (1.9 to 2.5)	-7.4 (-17.7 to 5)	326000 (285000 to 376000)	56.3 (49.2 to 64.6)	-8.7 (-19.7 to 4.3)
	Male	9020 (7670 to 10500)	3.4 (2.9 to 4)	-4.8 (-18.1 to 11.4)	9320 (7970 to 11000)	3.7 (3.2 to 4.3)	-7.6 (-20 to 6.8)	268000 (228000 to 319000)	94.5 (80.7 to 111.4)	-8.8 (-21.7 to 6.6)
	Female	2150 (1850 to 2450)	0.8 (0.7 to 0.9)	-1.1 (-12.9 to 13.7)	1910 (1640 to 2210)	0.7 (0.6 to 0.8)	-3.6 (-14.7 to 10.3)	57900 (49600 to 66900)	19.1 (16.5 to 22.1)	-5.7 (-17.4 to 8.9)
Central Asia	Both	1790 (1610 to 2010)	2.2 (2 to 2.5)	-16.1 (-24.4 to -6.8)	1340 (1200 to 1510)	1.8 (1.6 to 2)	-20.5 (-28 to -12)	39100 (34900 to 44100)	46.7 (41.8 to 52.4)	-21.9 (-29.7 to -12.8)
	Male	1430 (1270 to 1630)	4.2 (3.7 to 4.7)	-14.9 (-23.2 to -5)	1080 (964 to 1220)	3.4 (3 to 3.8)	-19 (-26.8 to -9.8)	31000 (27600 to 35200)	84.5 (75.2 to 95.8)	-20.9 (-28.7 to -11.4)
	Female	362 (309 to 428)	0.8 (0.7 to 1)	-19.2 (-31 to -2.7)	259 (222 to 305)	0.6 (0.5 to 0.7)	-24.2 (-34.8 to -9.8)	8060 (6860 to 9540)	17.4 (14.8 to 20.5)	-24.6 (-35.9 to -9.1)
Central Europe	Both	9160 (7920 to 10400)	4.7 (4 to 5.3)	-6.7 (-19.3 to 6.7)	5260 (4550 to 5990)	2.6 (2.2 to 2.9)	-13.4 (-25.2 to -1.6)	138000 (119000 to 157000)	71.8 (61.8 to 81.8)	-14.8 (-26.9 to -3)
	Male	8260 (7130 to 9430)	9.1 (7.8 to 10.4)	-8.5 (-21.2 to 4.6)	4780 (4130 to 5470)	5.2 (4.5 to 6)	-14.8 (-26.7 to -2.9)	126000 (108000 to 145000)	140.5 (120.6 to 161.2)	-16.2 (-28.2 to -4.2)

	Female	902 (750 to 1050)	0.9 (0.7 to 1)	-2.7 (-15.8 to 13.6)	482 (403 to 560)	0.4 (0.3 to 0.5)	-10.8 (-22.2 to 3)	11900 (9760 to 13900)	11.6 (9.6 to 13.7)	-11.6 (-23.1 to 2.6)
Eastern Europe	Both	11000 (9480 to 12600)	3.3 (2.8 to 3.8)	-7.3 (-19.9 to 6.7)	6560 (5650 to 7480)	1.9 (1.7 to 2.2)	-16.5 (-27.6 to -4.6)	185000 (160000 to 212000)	56.3 (48.7 to 64.4)	-16.3 (-27.4 to -4.6)
	Male	10400 (8910 to 11900)	7.5 (6.4 to 8.6)	-10 (-22.5 to 4.2)	6210 (5320 to 7140)	4.6 (3.9 to 5.2)	-18.7 (-29.8 to -6.9)	176000 (151000 to 202000)	126.8 (109.2 to 145.8)	-18.4 (-29.6 to -6.5)
	Female	607 (514 to 727)	0.3 (0.3 to 0.4)	2.1 (-13.2 to 21.1)	351 (301 to 415)	0.2 (0.1 to 0.2)	-10.3 (-22.9 to 7.1)	9100 (7750 to 10900)	5 (4.2 to 6)	-8.9 (-22.2 to 9.3)
Australasia	Both	897 (706 to 1130)	1.9 (1.5 to 2.4)	-8.6 (-28.3 to 15.6)	293 (267 to 321)	0.6 (0.5 to 0.6)	-13.1 (-20.6 to -5.1)	6540 (5910 to 7160)	14.1 (12.8 to 15.4)	-13.3 (-21.1 to -5.1)
	Male	775 (607 to 984)	3.4 (2.7 to 4.4)	-9.2 (-29 to 16.1)	253 (228 to 280)	1.1 (1 to 1.2)	-14.2 (-22.6 to -5.6)	5630 (5040 to 6220)	25.4 (22.8 to 28)	-13.6 (-22.4 to -4.3)
	Female	122 (92 to 161)	0.5 (0.4 to 0.7)	-6.3 (-28.5 to 20.5)	40 (34 to 46)	0.2 (0.1 to 0.2)	-11.6 (-22.3 to 0.1)	905 (773 to 1060)	3.8 (3.3 to 4.5)	-11 (-21.8 to 1)
High-income Asia Pacific	Both	7040 (6040 to 8190)	1.7 (1.5 to 2)	-8.3 (-20.6 to 6)	1770 (1570 to 1910)	0.4 (0.3 to 0.4)	-12.9 (-17.2 to -7.8)	35000 (31800 to 38400)	8.6 (7.9 to 9.5)	-14.1 (-18.8 to -8.3)
	Male	6320 (5370 to 7410)	3.3 (2.8 to 3.8)	-10.6 (-23.7 to 4.6)	1560 (1410 to 1690)	0.7 (0.7 to 0.8)	-15.5 (-19.9 to -10.4)	31100 (28400 to 33900)	16.3 (14.9 to 17.9)	-16.1 (-21.3 to -10.3)
	Female	725 (584 to 884)	0.3 (0.3 to 0.4)	-6.5 (-20.8 to 10.3)	202 (163 to 234)	0.1 (0.1 to 0.1)	-13.7 (-18.6 to -7.5)	3910 (3380 to 4500)	2 (1.8 to 2.2)	-12.8 (-18 to -6.4)
High-income North America	Both	17700 (14900 to 21000)	2.9 (2.4 to 3.5)	-5.3 (-20.6 to 12.5)	5440 (5160 to 5680)	0.9 (0.8 to 0.9)	-5.3 (-8.2 to -2)	129000 (123000 to 135000)	21.6 (20.6 to 22.6)	-7.2 (-10.3 to -3.6)
	Male	14800 (12200 to 18000)	5.2 (4.3 to 6.3)	-5.5 (-22.1 to 14.5)	4380 (4150 to 4580)	1.5 (1.4 to 1.6)	-5.5 (-9 to -1.8)	104000 (98600 to 109000)	36.9 (35 to 38.7)	-7.1 (-10.7 to -3.3)
	Female	2920 (2420 to 3570)	0.9 (0.7 to 1.1)	-8.3 (-23.3 to 11.1)	1070 (974 to 1170)	0.3 (0.3 to 0.3)	-8.9 (-13 to -4.1)	25000 (23200 to 27400)	8.1 (7.5 to 8.8)	-9.6 (-13.9 to -4.5)
Southern Latin America	Both	2160 (1680 to 2720)	2.6 (2 to 3.3)	-6.7 (-27.3 to 18.2)	1370 (1270 to 1480)	1.6 (1.5 to 1.8)	-13.4 (-19.6 to -6)	32900 (30500 to 35700)	40.4 (37.5 to 43.9)	-14.8 (-21.6 to -7.3)
	Male	1880 (1450 to 2380)	5.1 (3.9 to 6.4)	-7.9 (-28.7 to 17.9)	1190 (1100 to 1290)	3.2 (3 to 3.5)	-14.4 (-21.3 to -6.5)	28800 (26600 to 31600)	77.4 (71.4 to 84.5)	-15.8 (-23.1 to -7.6)
	Female	278 (218 to 354)	0.6 (0.5 to 0.8)	-0.8 (-22.4 to 25.2)	180 (158 to 200)	0.4 (0.3 to 0.4)	-8.1 (-17.7 to 2.5)	4050 (3580 to 4510)	9.2 (8.1 to 10.2)	-9.1 (-19.1 to 1.4)

Western Europe	Both	24200 (20900 to 28000)	3 (2.6 to 3.5)	-8.8 (-22.3 to 6.7)	9690 (9050 to 10200)	1.1 (1 to 1.2)	-13.4 (-16.9 to -9.6)	220000 (207000 to 232000)	28.2 (26.7 to 29.7)	-15.3 (-19.4 to -10.9)
	Male	21200 (18000 to 24700)	5.6 (4.7 to 6.6)	-10.7 (-24.3 to 5.2)	8510 (8010 to 8950)	2.1 (2 to 2.2)	-15.7 (-19.5 to -11.8)	194000 (183000 to 204000)	52.1 (49.2 to 55)	-17.1 (-21.3 to -12.4)
	Female	2960 (2400 to 3480)	0.7 (0.6 to 0.8)	-1.2 (-14.7 to 13.2)	1180 (943 to 1310)	0.2 (0.2 to 0.3)	-6.1 (-11.1 to 0.3)	26000 (20800 to 28800)	6.5 (5.3 to 7.2)	-6.6 (-11.9 to 0.6)
Andean Latin America	Both	481 (391 to 590)	0.9 (0.7 to 1.1)	-4.1 (-22.6 to 16.7)	398 (325 to 484)	0.7 (0.6 to 0.9)	-9.9 (-27.1 to 9.1)	8830 (7100 to 10900)	15.6 (12.6 to 19.2)	-12 (-29.2 to 6.9)
	Male	382 (307 to 472)	1.5 (1.2 to 1.8)	-2.9 (-22.8 to 19.4)	320 (260 to 393)	1.2 (1 to 1.5)	-8.6 (-26.7 to 11.5)	6940 (5550 to 8610)	25.8 (20.7 to 31.9)	-10.5 (-29 to 9.5)
	Female	99 (81 to 122)	0.3 (0.3 to 0.4)	-6.7 (-24.5 to 14.2)	78 (64 to 95)	0.3 (0.2 to 0.3)	-13 (-28.5 to 5.3)	1880 (1520 to 2330)	6.3 (5.1 to 7.8)	-15.5 (-31.9 to 4.3)
Caribbean	Both	2340 (1980 to 2770)	4.5 (3.8 to 5.3)	6.1 (-10.6 to 25.9)	1550 (1320 to 1790)	3 (2.5 to 3.4)	1 (-14 to 17.2)	37800 (32100 to 44100)	72.5 (61.5 to 84.3)	1.2 (-14.3 to 18.2)
	Male	2050 (1720 to 2430)	8.3 (7 to 9.9)	7.7 (-9.9 to 28.1)	1350 (1150 to 1570)	5.6 (4.7 to 6.5)	2.4 (-12.8 to 19.3)	33200 (28100 to 38900)	133.8 (113.3 to 156.1)	2.7 (-13.5 to 20.4)
	Female	295 (247 to 354)	1.1 (0.9 to 1.3)	0.8 (-15.2 to 19.9)	198 (168 to 233)	0.7 (0.6 to 0.8)	-3.8 (-17.6 to 11.9)	4580 (3920 to 5420)	16.8 (14.4 to 19.9)	-3.8 (-18.1 to 13.3)
Central Latin America	Both	3620 (3020 to 4290)	1.5 (1.3 to 1.8)	-4.4 (-19.5 to 12.1)	2660 (2250 to 3130)	1.2 (1 to 1.4)	-9.5 (-23 to 6)	61700 (51800 to 73400)	25.8 (21.8 to 30.7)	-9.4 (-24 to 7.2)
	Male	3030 (2500 to 3610)	2.8 (2.3 to 3.3)	-3.4 (-19.5 to 13.8)	2220 (1850 to 2630)	2.1 (1.8 to 2.5)	-8.4 (-23.1 to 7.7)	51600 (43000 to 61800)	46.8 (39 to 55.9)	-8.5 (-24.1 to 9.1)
	Female	597 (493 to 760)	0.5 (0.4 to 0.6)	-4 (-19.1 to 12.5)	441 (370 to 557)	0.4 (0.3 to 0.4)	-9.4 (-23.1 to 5.4)	10000 (8380 to 13200)	7.8 (6.5 to 10.3)	-9.2 (-23.3 to 6.3)
Tropical Latin America	Both	7790 (7350 to 8220)	3.1 (3 to 3.3)	-8.4 (-13.4 to -3.5)	5540 (5220 to 5810)	2.3 (2.1 to 2.4)	-12.8 (-17.1 to -8.2)	149000 (141000 to 156000)	59.1 (56 to 62.1)	-14.2 (-18.7 to -9.4)
	Male	6780 (6350 to 7210)	6 (5.6 to 6.3)	-8.4 (-13.9 to -2.7)	4800 (4510 to 5070)	4.3 (4 to 4.6)	-12.8 (-17.7 to -7.6)	131000 (123000 to 138000)	112 (105.5 to 118.2)	-14.2 (-19.2 to -8.9)
	Female	1010 (906 to 1120)	0.8 (0.7 to 0.8)	-6.3 (-13 to 2.5)	733 (649 to 811)	0.5 (0.5 to 0.6)	-10.9 (-17 to -3)	18200 (16600 to 20200)	13.6 (12.4 to 15)	-12.6 (-18.9 to -4.3)

North Africa and Middle East	Both	11200 (10100 to 12600)	2.5 (2.3 to 2.8)	1.8 (-6 to 11.8)	7640 (6860 to 8590)	1.8 (1.6 to 2)	-7.2 (-13.8 to 1.1)	209000 (184000 to 237000)	44.3 (39.5 to 50)	-8.3 (-15.6 to -0.2)
	Male	9450 (8450 to 10700)	4.2 (3.8 to 4.7)	-0.1 (-8.7 to 10.4)	6440 (5760 to 7300)	3 (2.7 to 3.4)	-8.7 (-15.7 to -0.1)	174000 (153000 to 200000)	72.8 (64.8 to 82.6)	-9.9 (-17.3 to -1)
	Female	1800 (1610 to 2020)	0.8 (0.7 to 0.9)	11.1 (3.3 to 21.2)	1200 (1070 to 1350)	0.6 (0.5 to 0.6)	0.4 (-6.6 to 9.3)	34700 (30400 to 40000)	14.6 (12.9 to 16.5)	-1.5 (-9.3 to 8.3)
South Asia	Both	46200 (39900 to 53600)	3.2 (2.7 to 3.7)	-3.2 (-17.1 to 13)	39900 (34400 to 46300)	2.8 (2.4 to 3.2)	-7.7 (-20.4 to 7.2)	1130000 (979000 to 1320000)	74.7 (64.4 to 86.6)	-8.2 (-21.3 to 7)
	Male	39200 (33300 to 46400)	5.4 (4.6 to 6.4)	-3.8 (-19.7 to 14.3)	33800 (28500 to 40000)	4.9 (4.1 to 5.7)	-8.1 (-22.6 to 8.4)	958000 (806000 to 1140000)	127.3 (107.1 to 151.6)	-8.7 (-23.5 to 8.6)
	Female	7060 (5880 to 8400)	0.9 (0.8 to 1.1)	6.7 (-10.1 to 28.6)	6020 (5030 to 7210)	0.8 (0.7 to 1)	1.5 (-15.2 to 21.7)	176000 (147000 to 211000)	22.8 (19 to 27.3)	1.1 (-16 to 21.4)
East Asia	Both	46600 (37800 to 56600)	2.2 (1.8 to 2.6)	11.6 (-8.4 to 38.3)	20900 (17400 to 24800)	1 (0.8 to 1.2)	-8.6 (-24.5 to 10)	514000 (428000 to 615000)	23.8 (19.9 to 28.3)	-8.7 (-24.9 to 10.9)
	Male	40000 (31500 to 49800)	3.9 (3.1 to 4.8)	14.8 (-8.3 to 45.8)	17400 (14000 to 21200)	1.8 (1.5 to 2.2)	-6.4 (-24.7 to 14.4)	435000 (351000 to 534000)	41.5 (33.7 to 50.7)	-6 (-25.1 to 15.4)
	Female	6580 (5250 to 7980)	0.6 (0.5 to 0.7)	-2.1 (-21.3 to 20.8)	3500 (2820 to 4210)	0.3 (0.3 to 0.4)	-16.8 (-33.7 to 1.2)	78400 (63600 to 94600)	7.3 (5.9 to 8.8)	-18 (-35 to -0.2)
Oceania	Both	61 (47 to 77)	0.9 (0.7 to 1.1)	-2.8 (-14.7 to 11)	54 (41 to 68)	0.9 (0.7 to 1)	-3.8 (-15.6 to 9.6)	1490 (1140 to 1910)	19.7 (15.1 to 24.9)	-4.8 (-17.8 to 9.8)
	Male	48 (36 to 62)	1.5 (1.1 to 1.8)	-3 (-16.1 to 12.4)	42 (32 to 54)	1.4 (1.1 to 1.8)	-4.2 (-16.6 to 11.4)	1150 (844 to 1510)	30.5 (23.1 to 39)	-4.6 (-18.7 to 11.9)
	Female	13 (10 to 17)	0.4 (0.3 to 0.5)	-4.1 (-20.9 to 16.9)	11 (8 to 15)	0.3 (0.2 to 0.4)	-5.5 (-21.9 to 14.6)	342 (248 to 456)	8.6 (6.4 to 11.5)	-6 (-23.7 to 16.2)
Southeast Asia	Both	10300 (8890 to 12100)	1.7 (1.4 to 1.9)	6.9 (-6.1 to 21.1)	7130 (6180 to 8260)	1.2 (1 to 1.4)	-3 (-13.6 to 7.9)	190000 (163000 to 221000)	29.1 (25.2 to 33.8)	-3.7 (-14.8 to 8)
	Male	9070 (7710 to 10700)	3.2 (2.7 to 3.7)	9.3 (-4.8 to 25.2)	6100 (5200 to 7190)	2.3 (1.9 to 2.7)	-0.7 (-12.4 to 11.7)	164000 (139000 to 194000)	53.8 (45.8 to 63.5)	-1.8 (-13.9 to 11.1)
	Female	1280 (1110 to 1470)	0.4 (0.3 to 0.4)	-6.7 (-17.8 to 6.6)	1030 (895 to 1170)	0.3 (0.3 to 0.4)	-12.3 (-23.3 to -1.5)	26200 (22300 to 30200)	7.7 (6.6 to 8.8)	-13.7 (-25.5 to -1.9)

Central Sub-Saharan Africa	Both	751 (585 to 955)	1.4 (1.1 to 1.7)	-2 (-17.7 to 17)	703 (550 to 888)	1.3 (1.1 to 1.7)	-3.9 (-18.3 to 14)	20700 (16000 to 26500)	34 (26.6 to 42.9)	-4.6 (-20.7 to 15.1)
	Male	629 (486 to 796)	2.6 (2 to 3.2)	-2.3 (-18.4 to 16.7)	590 (457 to 746)	2.6 (2 to 3.2)	-4 (-19.3 to 14.4)	17300 (13300 to 22400)	62.7 (48.8 to 79.3)	-4.9 (-21.7 to 14.9)
	Female	122 (93 to 158)	0.4 (0.3 to 0.5)	-7.8 (-24.7 to 14.2)	113 (87 to 146)	0.4 (0.3 to 0.5)	-9.2 (-25.6 to 11.6)	3360 (2520 to 4390)	10.1 (7.7 to 13.1)	-10.7 (-27.9 to 11.2)
Eastern sub-Saharan Africa	Both	2210 (1890 to 2730)	1.3 (1.1 to 1.6)	-6.2 (-15.1 to 4)	2050 (1750 to 2570)	1.2 (1.1 to 1.5)	-8 (-16.6 to 1.3)	61500 (52100 to 77000)	32.6 (27.8 to 40.8)	-9 (-18.3 to 1.1)
	Male	1780 (1490 to 2300)	2.2 (1.8 to 2.8)	-6.3 (-16 to 5)	1650 (1400 to 2130)	2.1 (1.8 to 2.7)	-8 (-17.3 to 2.5)	49400 (41500 to 63900)	54.6 (46.2 to 70.6)	-9.1 (-18.8 to 2.3)
	Female	428 (363 to 500)	0.5 (0.4 to 0.5)	-3.6 (-13.5 to 7.3)	393 (336 to 457)	0.4 (0.4 to 0.5)	-5.7 (-15.5 to 4.4)	12100 (10100 to 14300)	12 (10.2 to 14)	-6.9 (-17.2 to 4.3)
Southern sub-Saharan Africa	Both	1060 (968 to 1180)	1.8 (1.6 to 2)	-18.4 (-25.1 to -10.2)	900 (827 to 995)	1.6 (1.5 to 1.7)	-21.7 (-27.4 to -13.7)	25700 (23400 to 28600)	41.8 (38.2 to 46.3)	-23.3 (-29.6 to -14.8)
	Male	889 (805 to 995)	3.6 (3.3 to 4)	-18.6 (-25.7 to -9.6)	756 (690 to 841)	3.2 (2.9 to 3.5)	-22 (-27.8 to -13.3)	21700 (19700 to 24300)	81.8 (74.5 to 91.2)	-23.8 (-30.2 to -14.6)
	Female	169 (150 to 191)	0.5 (0.5 to 0.6)	-18.5 (-27.4 to -7)	144 (127 to 163)	0.4 (0.4 to 0.5)	-20.6 (-29.3 to -9.9)	4010 (3490 to 4600)	11.6 (10.1 to 13.2)	-22.6 (-32.1 to -11)
Western sub-Saharan Africa	Both	2480 (2010 to 2970)	1.3 (1.1 to 1.6)	-7.6 (-23.3 to 11.6)	2310 (1870 to 2800)	1.3 (1.1 to 1.5)	-8.6 (-23.6 to 10.9)	63700 (50700 to 78600)	30.9 (25 to 37.7)	-10.1 (-26.2 to 10.4)
	Male	2290 (1840 to 2770)	2.5 (2.1 to 3)	-3.2 (-20.4 to 17.8)	2130 (1710 to 2610)	2.5 (2 to 3)	-4.6 (-21 to 17.4)	58700 (46000 to 73000)	59.9 (47.9 to 73.5)	-5.3 (-22.9 to 18.1)
	Female	195 (163 to 228)	0.2 (0.2 to 0.2)	-4.8 (-16.9 to 8.5)	180 (152 to 212)	0.2 (0.2 to 0.2)	-6 (-17.6 to 7.8)	5020 (4150 to 6010)	4.6 (3.8 to 5.4)	-7.4 (-20.3 to 7.9)
Afghanistan	Both	459 (316 to 616)	3.3 (2.3 to 4.3)	-5.9 (-22.6 to 15)	423 (293 to 562)	3.3 (2.3 to 4.2)	-7.5 (-23.9 to 12.7)	13600 (8930 to 18800)	85.4 (59.2 to 113.6)	-8.1 (-25.7 to 13.5)
	Male	323 (204 to 461)	5.1 (3.4 to 7)	-6.7 (-25.3 to 16.7)	302 (195 to 425)	5.1 (3.4 to 6.9)	-8 (-25.9 to 14.1)	9370 (5620 to 13700)	127.7 (82.9 to 178.7)	-9.4 (-28.1 to 14.6)
	Female	136 (89 to 190)	1.7 (1.2 to 2.3)	-3.2 (-25 to 25.9)	121 (81 to 169)	1.6 (1.1 to 2.2)	-4.6 (-26.3 to 23.9)	4240 (2700 to 6020)	47.3 (31.8 to 65.1)	-6.9 (-28.5 to 23.1)
Albania	Both	133 (96 to 181)	3.1 (2.3 to 4.3)	11.5 (-17.7 to 47.9)	88 (63 to 119)	2.1 (1.5 to 2.8)	3.5 (-23.2 to 34.9)	2100 (1500 to 2890)	50.8 (36.2 to 70)	1.6 (-25.1 to 34.6)

	Male	117 (83 to 160)	5.7 (4.1 to 7.9)	12.3 (-18.1 to 49.2)	77 (55 to 105)	3.8 (2.8 to 5.2)	4 (-23.7 to 36.3)	1870 (1320 to 2570)	93.2 (66.1 to 128.2)	2.7 (-25 to 36.8)
	Female	16 (12 to 21)	0.8 (0.6 to 1)	14.6 (-14.4 to 49.9)	11 (8 to 14)	0.5 (0.4 to 0.6)	4.3 (-19.9 to 33.8)	236 (176 to 309)	11.4 (8.5 to 15.1)	4.6 (-21.5 to 36.3)
Algeria	Both	646 (499 to 830)	1.9 (1.5 to 2.4)	1.9 (-20.9 to 29.5)	439 (342 to 563)	1.4 (1.1 to 1.8)	-8.2 (-27.5 to 15.5)	11700 (8960 to 15000)	32.6 (25.3 to 41.8)	-9.6 (-30.7 to 15.9)
	Male	573 (439 to 741)	3.3 (2.6 to 4.3)	1 (-22.9 to 30.3)	393 (303 to 508)	2.4 (1.9 to 3.1)	-9.4 (-29.1 to 15.4)	10400 (7860 to 13400)	56.9 (43.8 to 73.1)	-10 (-31.4 to 17.4)
	Female	73 (56 to 93)	0.4 (0.3 to 0.5)	0.7 (-23.5 to 28)	46 (36 to 59)	0.3 (0.2 to 0.4)	-10.5 (-30.4 to 12.7)	1350 (1030 to 1740)	7.3 (5.6 to 9.3)	-11.4 (-33.3 to 13.3)
American Samoa	Both	0 (0 to 0)	0.7 (0.6 to 0.8)	-24.4 (-36.5 to -7.4)	0 (0 to 0)	0.6 (0.5 to 0.7)	-24.8 (-36 to -8)	6 (5 to 7)	12.4 (10.4 to 14.9)	-25.3 (-37.5 to -8.2)
	Male	0 (0 to 0)	1.3 (1.1 to 1.6)	-26.7 (-38.7 to -8.2)	0 (0 to 0)	1.1 (0.9 to 1.3)	-27.5 (-39.8 to -9.1)	5 (4 to 6)	22.8 (19.1 to 27.7)	-27.5 (-39.4 to -8.8)
	Female	0 (0 to 0)	0.1 (0.1 to 0.2)	-0.7 (-22 to 26.4)	0 (0 to 0)	0.1 (0.1 to 0.1)	-1.5 (-22.2 to 25.4)	1 (1 to 1)	2.9 (2.3 to 3.7)	-2 (-23 to 25.5)
Andorra	Both	4 (3 to 6)	3.1 (2.3 to 4.1)	-11.6 (-31.9 to 16.2)	1 (1 to 2)	1 (0.8 to 1.3)	-13.5 (-32.6 to 13)	38 (28 to 50)	26.9 (20 to 35.6)	-14.1 (-33.9 to 13.7)
	Male	4 (3 to 6)	5.8 (4.3 to 7.7)	-10.7 (-31.2 to 17.3)	1 (1 to 2)	1.9 (1.4 to 2.5)	-13 (-32 to 13)	36 (27 to 47)	49.8 (36.9 to 65.9)	-13.2 (-33.1 to 15.2)
	Female	0 (0 to 0)	0.2 (0.2 to 0.3)	6.6 (-23 to 47.9)	0 (0 to 0)	0.1 (0.1 to 0.1)	5 (-23.2 to 44.6)	2 (1 to 2)	2.5 (1.7 to 3.4)	3.6 (-24.9 to 41.5)
Angola	Both	192 (147 to 251)	1.7 (1.3 to 2.1)	-1.2 (-19.7 to 23.8)	178 (138 to 230)	1.6 (1.3 to 2.1)	-3.7 (-21.4 to 20.8)	5240 (3960 to 6830)	40.7 (31.7 to 52.5)	-4.6 (-23.5 to 21.2)
	Male	167 (127 to 220)	3.2 (2.5 to 4.2)	2.6 (-17 to 28.4)	155 (119 to 202)	3.2 (2.5 to 4.1)	0 (-19.1 to 26.9)	4540 (3420 to 5960)	78.5 (60.8 to 103)	-0.9 (-21.7 to 27.8)
	Female	25 (19 to 35)	0.4 (0.3 to 0.5)	-7.8 (-29.4 to 21.5)	23 (17 to 32)	0.4 (0.3 to 0.5)	-9.7 (-29.9 to 17.6)	701 (502 to 970)	9.7 (7.1 to 13.3)	-12.1 (-33.2 to 17)
Antigua and Barbuda	Both	2 (2 to 3)	2.2 (1.8 to 2.6)	-3 (-21.6 to 18.8)	2 (1 to 2)	1.6 (1.3 to 1.9)	-6 (-23.1 to 14.4)	39 (32 to 48)	37.1 (30.4 to 45)	-7.1 (-24.8 to 14.6)
	Male	2 (2 to 3)	4.5 (3.8 to 5.5)	-5.8 (-23.9 to 15.4)	2 (1 to 2)	3.4 (2.8 to 4)	-8.5 (-25.5 to 11.6)	38 (31 to 47)	76.3 (62.5 to 92.5)	-9.8 (-27.1 to 11.1)
	Female	0 (0 to 0)	0.1 (0.1 to 0.1)	3.8 (-16 to 27.7)	0 (0 to 0)	0.1 (0.1 to 0.1)	0.1 (-17.7 to 21.9)	1 (1 to 1)	1.9 (1.6 to 2.4)	-0.5 (-19.1 to 22.8)
Argentina	Both	1620 (1260 to 2060)	3.1 (2.4 to 3.9)	-5.4 (-27 to 21.9)	1050 (965 to 1150)	2 (1.8 to 2.1)	-12 (-19.6 to -2.5)	25700 (23500 to 28300)	49.3 (44.9 to 54.3)	-13.6 (-22.1 to -4.3)
	Male	1400 (1080 to 1790)	5.9 (4.5 to 7.5)	-6.8 (-28.4 to 20.7)	913 (827 to 1010)	3.9 (3.5 to 4.3)	-13.1 (-21.8 to -3.2)	22500 (20400 to 25100)	94.1 (85.4 to 104.8)	-14.9 (-24 to -4.5)
	Female	211 (164 to 273)	0.7 (0.6 to 0.9)	1.5 (-22 to 31.4)	140 (121 to 159)	0.5 (0.4 to 0.5)	-5.8 (-17.7 to 7.3)	3220 (2770 to 3660)	11.4 (9.7 to 12.9)	-7.1 (-19.7 to 6.3)

Armenia	Both	150 (122 to 182)	3.5 (2.9 to 4.2)	-10.5 (-26.8 to 8.5)	107 (88 to 129)	2.5 (2.1 to 3)	-16.3 (-31.1 to 0.8)	2780 (2260 to 3370)	65.6 (53.9 to 79.1)	-17.4 (-32.8 to 0.1)
	Male	135 (109 to 164)	7.3 (5.9 to 8.8)	-10.8 (-27.7 to 9.2)	96 (78 to 117)	5.3 (4.3 to 6.4)	-16.4 (-31.5 to 1.4)	2520 (2040 to 3060)	135.4 (110.2 to 163.6)	-17.5 (-33.1 to 0.4)
	Female	15 (12 to 18)	0.6 (0.5 to 0.8)	-9.9 (-27.7 to 10.4)	11 (9 to 13)	0.5 (0.4 to 0.5)	-16.3 (-31.5 to 1.1)	252 (201 to 306)	10.9 (8.7 to 13.4)	-16.8 (-32.8 to 0.9)
Australia	Both	788 (602 to 1030)	2 (1.5 to 2.6)	-9.2 (-30.4 to 19.1)	257 (231 to 284)	0.6 (0.6 to 0.7)	-13.4 (-21.9 to -4.6)	5710 (5110 to 6320)	14.7 (13.2 to 16.2)	-13.6 (-22.4 to -4.3)
	Male	675 (514 to 881)	3.6 (2.7 to 4.7)	-9.8 (-31.8 to 19.2)	225 (200 to 250)	1.1 (1 to 1.3)	-14.6 (-23.4 to -4.9)	4980 (4410 to 5550)	26.7 (23.7 to 29.6)	-14 (-23.7 to -3.3)
	Female	113 (83 to 151)	0.5 (0.4 to 0.7)	-6.2 (-29.7 to 23)	32 (26 to 38)	0.1 (0.1 to 0.2)	-11.7 (-24.3 to 1.8)	725 (604 to 864)	3.6 (3.1 to 4.3)	-10.8 (-23.5 to 3.5)
Austria	Both	318 (252 to 399)	2 (1.6 to 2.6)	-13.4 (-33.6 to 10)	144 (131 to 158)	0.9 (0.8 to 1)	-16.1 (-24.3 to -6.6)	3500 (3160 to 3880)	22.8 (20.5 to 25.3)	-19.3 (-27.6 to -9.4)
	Male	280 (219 to 352)	3.8 (2.9 to 4.8)	-15 (-35.2 to 8.8)	126 (113 to 139)	1.6 (1.5 to 1.8)	-17.9 (-26.6 to -7.8)	3070 (2760 to 3430)	41.9 (37.6 to 47)	-20.7 (-29.7 to -10.5)
	Female	37 (28 to 49)	0.5 (0.3 to 0.6)	-7.5 (-29 to 21.9)	18 (15 to 21)	0.2 (0.2 to 0.2)	-12.4 (-24.4 to 1.8)	426 (361 to 504)	5.4 (4.5 to 6.4)	-12.8 (-25 to 2.3)
Azerbaijan	Both	312 (242 to 418)	3 (2.4 to 4)	-1.7 (-20.4 to 23.3)	238 (185 to 320)	2.4 (1.9 to 3.2)	-6.5 (-24.1 to 16.3)	7160 (5510 to 9480)	64.7 (50.7 to 85.9)	-8.6 (-27.1 to 15)
	Male	259 (192 to 358)	5.4 (4.1 to 7.4)	-0.2 (-21.6 to 30)	198 (149 to 270)	4.4 (3.4 to 6)	-4.9 (-24.9 to 22.6)	5980 (4450 to 8220)	116.8 (88 to 160.1)	-7 (-27.5 to 21.8)
	Female	54 (41 to 71)	1 (0.8 to 1.3)	-6.1 (-28.3 to 20.9)	40 (30 to 53)	0.8 (0.6 to 1.1)	-10.4 (-30.1 to 15.5)	1180 (890 to 1570)	21 (16 to 27.8)	-14.3 (-35.2 to 12.4)
Bahamas	Both	13 (10 to 17)	3.2 (2.6 to 4)	0.9 (-19.5 to 26.5)	10 (8 to 12)	2.5 (2 to 3.1)	0.3 (-19.1 to 24.5)	266 (211 to 336)	62.9 (50.1 to 78.7)	-0.1 (-21 to 26.3)
	Male	12 (10 to 15)	6.6 (5.2 to 8.2)	0.5 (-19.7 to 26.7)	9 (7 to 11)	5.1 (4.1 to 6.3)	-0.3 (-19.5 to 24.4)	247 (195 to 313)	126.8 (100.4 to 159)	-0.3 (-21.3 to 26.2)
	Female	1 (1 to 1)	0.5 (0.4 to 0.6)	0 (-20.3 to 24.6)	1 (1 to 1)	0.4 (0.3 to 0.4)	-1.2 (-20.6 to 21.1)	19 (15 to 24)	8.6 (6.8 to 10.8)	-1 (-21.7 to 23.3)
Bahrain	Both	13 (9 to 16)	1.6 (1.2 to 2.1)	-14.4 (-33.9 to 9.3)	7 (6 to 10)	1.2 (0.8 to 1.5)	-22.5 (-39.8 to -1.5)	187 (141 to 243)	21.5 (16 to 27.6)	-23 (-40.3 to -1.7)
	Male	12 (9 to 15)	2.9 (2 to 3.7)	-19.3 (-38.3 to 4.1)	7 (5 to 9)	2.1 (1.5 to 2.8)	-25.9 (-42.9 to -5.3)	173 (130 to 226)	36.6 (26.8 to 47.3)	-27.5 (-44.9 to -6.6)
	Female	1 (1 to 1)	0.2 (0.2 to 0.3)	-13.2 (-32.9 to 8.9)	0 (0 to 1)	0.1 (0.1 to 0.2)	-23.9 (-40.5 to -5)	14 (11 to 18)	3.2 (2.5 to 4)	-21.9 (-38.9 to -1.2)
Bangladesh	Both	3410 (2510 to 4910)	2.6 (1.9 to 3.7)	-13.4 (-33.1 to 11.9)	2930 (2170 to 4220)	2.2 (1.7 to 3.2)	-18.9 (-37.2 to 4.6)	79200 (57800 to 115000)	57.7 (42.2 to 83.4)	-18.8 (-37.4 to 5.1)
	Male	2970 (2140 to 4410)	4.3 (3.1 to 6.4)	-11.5 (-32.3 to 15.6)	2560 (1840 to 3790)	3.8 (2.8 to 5.6)	-17.3 (-36.5 to 7.3)	68600 (48700 to 102000)	97.9 (69.8 to 146.2)	-16.5 (-36.8 to 9.4)

	Female	442 (332 to 575)	0.7 (0.5 to 0.9)	-5.6 (-27.4 to 21.7)	368 (279 to 481)	0.6 (0.4 to 0.8)	-11.7 (-32 to 13.8)	10600 (7860 to 13800)	15.4 (11.5 to 20.1)	-12.2 (-34.1 to 13.6)
Barbados	Both	10 (8 to 12)	2 (1.6 to 2.4)	10 (-11 to 33.5)	7 (6 to 8)	1.4 (1.2 to 1.7)	9 (-10.5 to 30.6)	162 (132 to 196)	33.4 (27.1 to 40.5)	5 (-15.2 to 27.5)
	Male	9 (7 to 11)	3.9 (3.2 to 4.7)	8.9 (-12.8 to 32.9)	6 (5 to 7)	2.8 (2.3 to 3.4)	7.3 (-12.6 to 29.6)	144 (116 to 175)	64.7 (52.2 to 78.4)	4.3 (-16.2 to 27.5)
	Female	1 (1 to 1)	0.4 (0.4 to 0.5)	2 (-17.2 to 24.4)	1 (1 to 1)	0.3 (0.2 to 0.4)	-0.8 (-18.4 to 19.8)	18 (15 to 22)	6.9 (5.6 to 8.3)	-1 (-19.8 to 21.1)
Belarus	Both	603 (460 to 803)	3.9 (2.9 to 5.1)	-6.8 (-29.9 to 24.5)	344 (262 to 454)	2.2 (1.7 to 2.9)	-20.1 (-39.4 to 5.6)	9870 (7490 to 13100)	64.5 (48.9 to 85.2)	-21 (-40.5 to 6.6)
	Male	585 (446 to 782)	9 (6.9 to 11.9)	-7.9 (-30.4 to 22.3)	334 (254 to 441)	5.3 (4 to 6.9)	-20.8 (-39.5 to 3.7)	9610 (7290 to 12800)	147.5 (112.2 to 196)	-21.6 (-40.7 to 4.5)
	Female	18 (13 to 30)	0.2 (0.1 to 0.3)	-3.7 (-31.1 to 30.9)	10 (8 to 17)	0.1 (0.1 to 0.2)	-18.7 (-40.3 to 8.3)	252 (179 to 424)	3 (2.1 to 4.9)	-20.1 (-42.3 to 8.4)
Belgium	Both	628 (475 to 802)	3.1 (2.4 to 4)	-12.9 (-34.3 to 14.3)	241 (218 to 264)	1.1 (1 to 1.2)	-19.6 (-27.1 to -11.4)	5760 (5190 to 6410)	29.8 (26.8 to 33.2)	-20.9 (-29.2 to -11.5)
	Male	553 (418 to 717)	5.8 (4.4 to 7.5)	-14.2 (-35.6 to 13)	204 (183 to 226)	2.1 (1.9 to 2.3)	-21.5 (-29.9 to -12.7)	4900 (4360 to 5500)	52.5 (46.8 to 58.8)	-22.2 (-31.4 to -12.1)
	Female	75 (56 to 98)	0.7 (0.5 to 1)	-11.7 (-34.3 to 16.7)	37 (32 to 43)	0.3 (0.3 to 0.4)	-17.2 (-28.1 to -3.5)	852 (732 to 999)	8.7 (7.5 to 10.2)	-17.8 (-29.5 to -3)
Belize	Both	6 (5 to 7)	2.1 (1.8 to 2.4)	3.7 (-11.5 to 21.2)	5 (4 to 5)	1.7 (1.5 to 2)	1.8 (-12.3 to 17.6)	129 (110 to 150)	42.9 (36.8 to 50)	1.3 (-13.3 to 18.6)
	Male	5 (5 to 6)	3.7 (3.2 to 4.4)	5.9 (-10.2 to 24.8)	4 (4 to 5)	3.1 (2.6 to 3.6)	3.7 (-11.4 to 20.4)	117 (100 to 138)	77.1 (65.8 to 90.6)	3.8 (-11.9 to 22)
	Female	1 (0 to 1)	0.4 (0.3 to 0.5)	-3.5 (-18.2 to 13.6)	0 (0 to 1)	0.3 (0.3 to 0.4)	-5.7 (-20 to 10.3)	11 (10 to 14)	7.7 (6.6 to 9.2)	-5.1 (-19.8 to 11.8)
Benin	Both	60 (45 to 78)	1.3 (1 to 1.6)	-3.6 (-21.7 to 17.2)	57 (43 to 74)	1.2 (1 to 1.6)	-4.6 (-22.4 to 15.1)	1520 (1110 to 2020)	29.6 (22 to 38.8)	-5.3 (-23.6 to 16.6)
	Male	54 (39 to 71)	2.4 (1.8 to 3.2)	-4 (-22.3 to 17.9)	51 (37 to 67)	2.5 (1.9 to 3.2)	-4.8 (-22.6 to 15.7)	1360 (981 to 1810)	56.4 (41.1 to 74.1)	-5.9 (-24.5 to 16.6)
	Female	6 (5 to 8)	0.2 (0.2 to 0.3)	-5.9 (-26.2 to 18.5)	6 (5 to 8)	0.2 (0.2 to 0.3)	-6.5 (-26 to 17)	162 (118 to 218)	5.8 (4.3 to 7.7)	-7.8 (-28.4 to 16.9)
Bermuda	Both	4 (3 to 5)	3.3 (2.7 to 4)	-1.6 (-18.9 to 20.2)	2 (2 to 2)	1.6 (1.3 to 1.9)	-8.9 (-23.9 to 9.2)	47 (38 to 57)	37.8 (30.9 to 46.3)	-9.3 (-26.2 to 11)
	Male	4 (3 to 5)	6.7 (5.5 to 8.2)	-3.5 (-20.7 to 18)	2 (2 to 2)	3.4 (2.8 to 4)	-10.7 (-25.7 to 6.9)	44 (36 to 53)	77.1 (63.1 to 94)	-11.1 (-27.5 to 9.2)
	Female	0 (0 to 0)	0.4 (0.3 to 0.5)	0.7 (-19.1 to 26)	0 (0 to 0)	0.2 (0.1 to 0.2)	-7.4 (-24.4 to 16.3)	3 (2 to 4)	4.1 (3.2 to 5.2)	-6.8 (-24.1 to 18.8)
Bhutan	Both	14 (10 to 20)	2.5 (1.7 to 3.6)	0.6 (-18.5 to 23.6)	12 (8 to 17)	2.1 (1.5 to 3.1)	-4.7 (-21.9 to 15.4)	310 (212 to 463)	52.5 (35.9 to 77.7)	-6.5 (-24.7 to 16.5)
	Male	11 (7 to 17)	3.9 (2.6 to 5.9)	1.1 (-20.1 to 26.3)	10 (6 to 14)	3.5 (2.3 to 5.1)	-4.3 (-22.9 to 17.5)	252 (165 to 393)	83.3 (54.6 to 128.3)	-5.7 (-26.3 to 19.2)

	Female	3 (2 to 4)	0.9 (0.7 to 1.3)	2.5 (-20 to 33.2)	2 (2 to 3)	0.8 (0.6 to 1)	-3.5 (-23.8 to 24.7)	58 (41 to 82)	19.9 (14.3 to 27.4)	-5.5 (-27.2 to 23.6)
Bolivia	Both	120 (92 to 155)	1.4 (1.1 to 1.8)	5.7 (-15.3 to 31.2)	110 (84 to 140)	1.3 (1 to 1.7)	1.8 (-18.2 to 24.8)	2560 (1950 to 3370)	28.3 (21.6 to 36.9)	-0.9 (-21.6 to 23.9)
	Male	95 (70 to 123)	2.3 (1.7 to 3)	7.1 (-16.1 to 34.8)	88 (65 to 113)	2.2 (1.7 to 2.9)	3 (-18.9 to 28.7)	2000 (1460 to 2650)	46.9 (34.5 to 61.5)	1 (-21.7 to 28.2)
	Female	25 (19 to 33)	0.6 (0.4 to 0.7)	-0.5 (-20.4 to 24.4)	23 (17 to 29)	0.5 (0.4 to 0.6)	-4.3 (-23.1 to 18.9)	558 (421 to 739)	11.6 (8.8 to 15.3)	-7.5 (-26.3 to 17.8)
Bosnia and Herzegovina	Both	222 (171 to 287)	3.7 (2.8 to 4.8)	4.2 (-19.4 to 34.7)	155 (120 to 196)	2.6 (2 to 3.3)	0.1 (-22.3 to 27.8)	3720 (2850 to 4820)	62.7 (47.8 to 81.3)	-1.4 (-24.1 to 27.3)
	Male	198 (151 to 258)	7.3 (5.5 to 9.4)	3.4 (-19.9 to 34)	139 (107 to 177)	5.2 (4 to 6.6)	-0.6 (-22.9 to 27.6)	3350 (2550 to 4330)	122.8 (93.8 to 159.6)	-2 (-25 to 27.4)
	Female	24 (18 to 30)	0.7 (0.6 to 1)	3.2 (-21.3 to 33.2)	17 (13 to 21)	0.5 (0.4 to 0.6)	-1.8 (-23 to 24.7)	365 (284 to 466)	11.7 (9.1 to 15.1)	-2.4 (-25.7 to 26.3)
Botswana	Both	38 (27 to 53)	2.6 (1.9 to 3.5)	-8.8 (-28.1 to 13.9)	30 (22 to 41)	2.1 (1.6 to 2.8)	-13.9 (-31.5 to 6.2)	909 (630 to 1270)	57.1 (40.5 to 78.4)	-15.9 (-35 to 6.4)
	Male	32 (23 to 44)	5 (3.7 to 6.9)	-10.7 (-29.9 to 9.8)	25 (18 to 35)	4.2 (3.1 to 5.8)	-15.5 (-32.7 to 3.4)	761 (526 to 1070)	109.1 (78 to 150.7)	-17.6 (-36.1 to 3.3)
	Female	6 (4 to 9)	0.8 (0.5 to 1.1)	-2.6 (-30.2 to 39.1)	5 (3 to 7)	0.6 (0.4 to 0.9)	-9 (-34 to 27.4)	148 (93 to 222)	16.7 (10.7 to 24.6)	-9.4 (-35.9 to 30.3)
Brazil	Both	7680 (7250 to 8110)	3.2 (3 to 3.3)	-8.7 (-13.7 to -3.8)	5450 (5140 to 5730)	2.3 (2.1 to 2.4)	-13.1 (-17.4 to -8.4)	147000 (139000 to 154000)	59.6 (56.4 to 62.6)	-14.5 (-19.1 to -9.7)
	Male	6680 (6250 to 7110)	6 (5.6 to 6.4)	-8.7 (-14.3 to -3)	4730 (4430 to 4990)	4.4 (4.1 to 4.6)	-13.1 (-18.1 to -7.9)	128000 (121000 to 136000)	113 (106.1 to 119.2)	-14.5 (-19.6 to -9.2)
	Female	1000 (899 to 1110)	0.8 (0.7 to 0.8)	-6.5 (-13.2 to 2.4)	726 (641 to 805)	0.6 (0.5 to 0.6)	-11 (-17.1 to -3.1)	18100 (16400 to 20000)	13.8 (12.5 to 15.3)	-12.7 (-19.1 to -4.4)
Brunei	Both	6 (5 to 7)	2.1 (1.7 to 2.6)	-8.8 (-24.8 to 8.7)	3 (2 to 3)	1.1 (0.9 to 1.3)	-15.8 (-28.5 to -0.7)	67 (56 to 82)	22.3 (18.8 to 26.8)	-13.6 (-27.3 to 2.8)
	Male	5 (4 to 6)	3.9 (3.2 to 4.9)	-3.2 (-22.5 to 17.7)	2 (2 to 3)	2.2 (1.8 to 2.7)	-5.7 (-22.3 to 13.9)	53 (43 to 67)	40.5 (33.3 to 49.7)	-9.3 (-25.6 to 9.4)
	Female	1 (1 to 1)	0.8 (0.6 to 0.9)	-11.5 (-29 to 7.7)	1 (0 to 1)	0.4 (0.3 to 0.5)	-17.9 (-31.9 to -0.7)	14 (11 to 17)	8.7 (7.3 to 10.3)	-15.2 (-29.7 to 2.4)
Bulgaria	Both	702 (530 to 907)	5.6 (4.2 to 7.3)	2.9 (-23.7 to 34.5)	401 (309 to 509)	3.1 (2.3 to 3.9)	-7.1 (-29.8 to 19.7)	10900 (8280 to 13900)	90 (68.6 to 116)	-6.5 (-30 to 22.3)
	Male	666 (500 to 863)	11.4 (8.6 to 14.9)	0.8 (-25.8 to 32.5)	377 (289 to 480)	6.3 (4.8 to 8.1)	-8.5 (-30.8 to 18.4)	10300 (7820 to 13200)	180.5 (137.1 to 231.5)	-8.4 (-32.1 to 20.1)
	Female	36 (27 to 47)	0.5 (0.4 to 0.7)	12 (-16.9 to 48.5)	24 (18 to 31)	0.3 (0.2 to 0.4)	0.7 (-23.1 to 30)	579 (432 to 756)	9.1 (6.7 to 12.1)	2.3 (-24.6 to 35.8)

Burkina Faso	Both	135 (104 to 170)	1.5 (1.2 to 1.9)	8.7 (-10.8 to 34.2)	128 (99 to 161)	1.5 (1.2 to 1.9)	6.8 (-11.8 to 30.6)	3470 (2660 to 4460)	35.5 (27.3 to 44.9)	8.5 (-11.8 to 34.9)
	Male	121 (92 to 156)	3 (2.3 to 3.8)	12.1 (-9.1 to 38.8)	115 (88 to 147)	3 (2.3 to 3.8)	10.2 (-9.9 to 35.2)	3110 (2330 to 4040)	70.4 (53.5 to 90.7)	11.5 (-10.5 to 40)
	Female	14 (11 to 18)	0.3 (0.2 to 0.3)	8.1 (-13.5 to 38.3)	13 (10 to 16)	0.3 (0.2 to 0.3)	7 (-13.7 to 35)	360 (270 to 460)	6.6 (5 to 8.4)	7.4 (-15.9 to 38.7)
Burundi	Both	77 (55 to 109)	1.6 (1.1 to 2.2)	-6.6 (-24.3 to 17.9)	73 (52 to 102)	1.5 (1.1 to 2.1)	-6.8 (-24 to 14.8)	2230 (1570 to 3160)	41.1 (29.4 to 57.5)	-7.3 (-25.6 to 17.2)
	Male	62 (43 to 91)	2.4 (1.7 to 3.5)	-8.4 (-26.6 to 15.5)	59 (41 to 85)	2.4 (1.7 to 3.4)	-8.8 (-26.2 to 14.7)	1790 (1220 to 2640)	63.2 (43.9 to 91.8)	-8.8 (-28.3 to 15.9)
	Female	15 (11 to 21)	0.6 (0.4 to 0.8)	-9.3 (-30.8 to 16.9)	14 (10 to 19)	0.6 (0.4 to 0.8)	-9.5 (-31.1 to 15.8)	443 (313 to 618)	16.4 (11.7 to 22.6)	-10.3 (-31.5 to 17.9)
Cabo Verde	Both	7 (6 to 8)	1.6 (1.4 to 1.9)	53.1 (25.9 to 84.1)	6 (5 to 7)	1.4 (1.2 to 1.6)	46.3 (21.8 to 74.2)	133 (110 to 163)	30.4 (25.4 to 37.1)	36.9 (11.1 to 66.5)
	Male	6 (5 to 8)	3.5 (2.9 to 4.2)	48.2 (20.6 to 80.2)	5 (4 to 6)	3 (2.6 to 3.6)	42.4 (16.9 to 71.5)	122 (101 to 151)	64.6 (53.7 to 79)	32.8 (7.1 to 62.6)
	Female	1 (0 to 1)	0.2 (0.2 to 0.3)	44 (13.6 to 84.3)	1 (0 to 1)	0.2 (0.2 to 0.3)	38.3 (9.1 to 77.2)	10 (8 to 13)	4.3 (3.5 to 5.4)	27.1 (0.4 to 62.1)
Cambodia	Both	222 (165 to 292)	1.9 (1.4 to 2.4)	14.2 (-6.4 to 36.1)	187 (140 to 242)	1.6 (1.2 to 2.1)	6.4 (-12 to 26.7)	4860 (3600 to 6370)	38.6 (28.9 to 50.5)	5.3 (-14.6 to 27.8)
	Male	183 (133 to 247)	3.8 (2.7 to 5.1)	18.3 (-4 to 44.6)	152 (111 to 204)	3.3 (2.4 to 4.5)	10.3 (-10 to 34.6)	3960 (2860 to 5290)	75.8 (55.2 to 102.4)	8.3 (-12.5 to 33.1)
	Female	39 (30 to 50)	0.6 (0.4 to 0.7)	0.6 (-19 to 26.2)	34 (26 to 44)	0.5 (0.4 to 0.7)	-4 (-22 to 18.9)	892 (665 to 1160)	12.3 (9.3 to 15.8)	-6.6 (-25.8 to 19.6)
Cameroon	Both	200 (145 to 272)	1.7 (1.2 to 2.2)	-4.8 (-25 to 19.2)	184 (134 to 248)	1.6 (1.2 to 2.1)	-7.3 (-25.3 to 16.2)	5070 (3580 to 7000)	39.3 (28.5 to 53.6)	-8.9 (-28.7 to 18.5)
	Male	183 (129 to 250)	3.2 (2.4 to 4.3)	-4 (-24.7 to 20.1)	168 (120 to 228)	3.1 (2.3 to 4.1)	-6.4 (-25.9 to 17.5)	4630 (3240 to 6410)	74.2 (53.2 to 100.9)	-8 (-28.5 to 19.9)
	Female	17 (12 to 24)	0.3 (0.2 to 0.4)	-7.5 (-30.9 to 21.4)	16 (11 to 22)	0.3 (0.2 to 0.4)	-9.9 (-32 to 16.7)	431 (298 to 608)	6.4 (4.5 to 8.9)	-12 (-34 to 16.8)
Canada	Both	1510 (1140 to 1930)	2.3 (1.7 to 2.9)	-5.9 (-29 to 20.4)	508 (459 to 556)	0.7 (0.7 to 0.8)	-8.8 (-17.2 to -0.3)	11300 (10100 to 12500)	17.2 (15.4 to 19)	-10.2 (-19 to -0.9)
	Male	1240 (936 to 1610)	3.9 (3 to 5)	-6.4 (-30.1 to 20.4)	421 (377 to 466)	1.3 (1.2 to 1.4)	-9.8 (-19.2 to -1)	9360 (8260 to 10400)	30 (26.6 to 33.2)	-10.6 (-20.7 to -0.6)
	Female	269 (199 to 357)	0.8 (0.6 to 1)	-8.1 (-31.8 to 21.4)	87 (73 to 102)	0.2 (0.2 to 0.3)	-12.5 (-23.9 to 1.3)	1940 (1630 to 2320)	5.8 (4.9 to 6.9)	-12.3 (-24.2 to 2.1)
Central Africa Republic	Both	43 (30 to 59)	1.8 (1.3 to 2.4)	-9.1 (-26 to 10.1)	42 (30 to 57)	1.8 (1.3 to 2.4)	-9.2 (-25.9 to 10.2)	1310 (911 to 1840)	49.5 (35.4 to 67.9)	-9.4 (-27.6 to 11.4)
	Male	36 (25 to 50)	3.4 (2.5 to 4.5)	-8.8 (-26.8 to 10.9)	35 (25 to 49)	3.5 (2.6 to 4.6)	-8.9 (-26.3 to 11)	1120 (758 to 1570)	91.9 (65.7 to 125.1)	-9.4 (-28.4 to 11.8)
	Female	6 (4 to 9)	0.5 (0.3 to 0.7)	-10.1 (-32.9 to 19.1)	6 (4 to 9)	0.5 (0.3 to 0.7)	-9.8 (-32.1 to 19)	195 (128 to 285)	13.8 (9.2 to 20)	-11 (-34.6 to 18.9)

Chad	Both	75 (57 to 96)	1.4 (1.1 to 1.7)	-2.5 (-20.8 to 20.4)	74 (56 to 93)	1.4 (1.1 to 1.7)	-3.4 (-21 to 18.4)	1960 (1460 to 2540)	33.2 (25 to 42.5)	-5.2 (-23.4 to 18.4)
	Male	68 (51 to 87)	2.3 (1.7 to 2.9)	-4.8 (-23.6 to 19.3)	66 (50 to 85)	2.3 (1.8 to 3)	-5.9 (-23.6 to 17.1)	1760 (1300 to 2300)	55.3 (41.2 to 71.6)	-7.1 (-26.1 to 17.9)
	Female	7 (5 to 10)	0.3 (0.2 to 0.4)	-4.7 (-25.5 to 20)	7 (5 to 9)	0.3 (0.2 to 0.4)	-5.3 (-25.1 to 19)	197 (144 to 264)	6.9 (5.2 to 9.2)	-7.3 (-27.4 to 18.8)
Chile	Both	321 (248 to 408)	1.3 (1 to 1.7)	-5.6 (-26.9 to 19.2)	179 (162 to 197)	0.7 (0.7 to 0.8)	-14.3 (-23.1 to -5.3)	3920 (3540 to 4330)	16.2 (14.6 to 17.9)	-14.6 (-23.5 to -5)
	Male	275 (209 to 352)	2.5 (1.9 to 3.2)	-5.9 (-28.1 to 19.3)	153 (137 to 171)	1.4 (1.3 to 1.6)	-14.7 (-24.4 to -4.7)	3380 (3020 to 3760)	30.6 (27.5 to 34.1)	-14.8 (-24.5 to -4.2)
	Female	46 (34 to 59)	0.3 (0.3 to 0.4)	-4.6 (-28 to 25.4)	26 (22 to 30)	0.2 (0.2 to 0.2)	-13.4 (-24.8 to 0.8)	540 (461 to 628)	4.2 (3.6 to 4.9)	-13.3 (-25.1 to 0.7)
China	Both	45400 (36800 to 55400)	2.2 (1.8 to 2.7)	12 (-8.8 to 39.5)	20300 (16800 to 24200)	1 (0.8 to 1.2)	-8.6 (-24.9 to 10.4)	498000 (412000 to 600000)	23.9 (19.8 to 28.6)	-8.7 (-25.4 to 11.5)
	Male	38900 (30400 to 48700)	3.9 (3.1 to 4.8)	15.2 (-8.8 to 46.5)	16800 (13500 to 20700)	1.8 (1.5 to 2.2)	-6.4 (-25.6 to 15.1)	422000 (338000 to 520000)	41.5 (33.5 to 51)	-6 (-26 to 16.3)
	Female	6450 (5130 to 7880)	0.6 (0.5 to 0.8)	-2.1 (-21.7 to 21.3)	3420 (2740 to 4130)	0.3 (0.3 to 0.4)	-16.8 (-34.1 to 1.8)	76400 (61400 to 92800)	7.3 (5.9 to 8.9)	-18.2 (-35.5 to 0.4)
Colombia	Both	773 (583 to 1000)	1.5 (1.1 to 1.9)	-8.9 (-32 to 17)	550 (422 to 701)	1 (0.8 to 1.3)	-16.6 (-36.7 to 6.7)	11800 (8880 to 15500)	22.4 (16.9 to 29.4)	-17.3 (-38.8 to 7.2)
	Male	600 (450 to 789)	2.5 (1.9 to 3.3)	-9.3 (-33.1 to 20.3)	428 (322 to 556)	1.8 (1.4 to 2.3)	-16.5 (-38 to 8.9)	9240 (6860 to 12200)	38.5 (28.6 to 50.6)	-17.8 (-39.6 to 9.5)
	Female	173 (129 to 238)	0.6 (0.4 to 0.8)	-3.4 (-26.1 to 23.2)	122 (93 to 165)	0.4 (0.3 to 0.6)	-12.5 (-32.8 to 9.4)	2580 (1930 to 3680)	9 (6.7 to 12.9)	-11.8 (-32.9 to 11.8)
Comoros	Both	5 (4 to 7)	1.1 (0.8 to 1.4)	1.5 (-20.9 to 26.6)	5 (4 to 7)	1 (0.8 to 1.3)	-0.8 (-21.6 to 22.5)	141 (100 to 192)	26.8 (19.5 to 36.1)	1.5 (-22.4 to 29.5)
	Male	4 (3 to 5)	1.7 (1.3 to 2.3)	2.1 (-21.9 to 30)	4 (3 to 5)	1.6 (1.2 to 2.2)	-0.1 (-23.1 to 25.7)	103 (71 to 143)	42.1 (30.2 to 57.8)	1.8 (-23.4 to 31.6)
	Female	1 (1 to 2)	0.5 (0.4 to 0.7)	-1.4 (-24 to 29.3)	1 (1 to 2)	0.5 (0.4 to 0.6)	-3.4 (-24.7 to 25.8)	38 (27 to 52)	13.6 (9.8 to 18.2)	-1.7 (-26.7 to 31)
Congo	Both	43 (32 to 56)	1.6 (1.2 to 2)	-0.4 (-19 to 22.8)	39 (30 to 51)	1.5 (1.2 to 2)	-1.9 (-20 to 20.6)	1150 (841 to 1520)	38.6 (29.3 to 50.2)	-4.2 (-23.7 to 20.3)
	Male	36 (27 to 48)	2.8 (2.2 to 3.7)	-1.7 (-21 to 22.9)	33 (25 to 44)	2.8 (2.2 to 3.6)	-3.4 (-21.6 to 19.8)	952 (700 to 1280)	66.7 (50.8 to 87.5)	-5 (-24.8 to 19.8)
	Female	7 (5 to 10)	0.5 (0.4 to 0.7)	-11 (-32.2 to 17.4)	6 (5 to 9)	0.5 (0.3 to 0.6)	-12.3 (-33.1 to 14.9)	194 (131 to 271)	12.3 (8.6 to 17.1)	-14.9 (-37 to 13.4)
Cook Islands	Both	0 (0 to 0)	0.8 (0.7 to 1)	-10.7 (-25.8 to 6.3)	0 (0 to 0)	0.5 (0.4 to 0.6)	-17.5 (-30.4 to -2)	3 (2 to 3)	11.1 (9.1 to 13.9)	-16.4 (-30.7 to 0.5)

	Male	0 (0 to 0)	1.5 (1.2 to 1.8)	-9.5 (-25.2 to 9.9)	0 (0 to 0)	0.9 (0.8 to 1.2)	-16.4 (-30 to 0.9)	2 (2 to 3)	19.5 (15.9 to 24.4)	-15.3 (-30.3 to 3.5)
	Female	0 (0 to 0)	0.2 (0.2 to 0.3)	-6.1 (-28.5 to 25.6)	0 (0 to 0)	0.1 (0.1 to 0.2)	-12 (-31.8 to 15.3)	0 (0 to 1)	3.4 (2.5 to 4.5)	-12.5 (-33.4 to 16.4)
Costa Rica	Both	87 (66 to 112)	1.7 (1.3 to 2.2)	-5.9 (-28.4 to 20.9)	56 (43 to 71)	1.1 (0.8 to 1.4)	-11.2 (-31.5 to 13.1)	1240 (937 to 1600)	24 (18.2 to 30.9)	-12.1 (-33.4 to 13)
	Male	77 (58 to 100)	3.3 (2.5 to 4.2)	-3.8 (-27.3 to 24.4)	50 (38 to 64)	2.2 (1.6 to 2.8)	-9.3 (-30.3 to 16.8)	1110 (831 to 1430)	46.3 (35 to 59.5)	-10.2 (-32.5 to 17)
	Female	10 (7 to 12)	0.3 (0.3 to 0.4)	-7.6 (-29 to 21.2)	6 (5 to 8)	0.2 (0.2 to 0.3)	-12.2 (-31.8 to 14.2)	131 (100 to 172)	4.7 (3.6 to 6.2)	-13.9 (-33.9 to 11.4)
Croatia	Both	345 (264 to 445)	4.3 (3.3 to 5.6)	-10.6 (-32.7 to 17.6)	193 (150 to 246)	2.3 (1.8 to 3)	-16.1 (-35.8 to 9.2)	4660 (3590 to 6060)	59.9 (45.9 to 78.1)	-17.9 (-37.8 to 7.9)
	Male	321 (245 to 415)	8.8 (6.7 to 11.4)	-12.2 (-34.4 to 14.9)	180 (139 to 230)	4.9 (3.8 to 6.2)	-17.8 (-36.9 to 6.7)	4390 (3350 to 5730)	121.7 (93.4 to 158.6)	-19.1 (-38.8 to 6.6)
	Female	24 (18 to 32)	0.6 (0.4 to 0.7)	-13.9 (-35.3 to 17.8)	13 (10 to 17)	0.3 (0.2 to 0.3)	-21.4 (-39.8 to 4.2)	276 (205 to 365)	6.6 (4.9 to 8.6)	-21.9 (-41.8 to 5.9)
Cuba	Both	1600 (1290 to 1980)	8.5 (6.8 to 10.5)	7.4 (-14.6 to 33.4)	962 (781 to 1180)	5 (4.1 to 6.2)	-0.2 (-19.7 to 22.5)	23000 (18300 to 28600)	123.1 (97.9 to 152.8)	-0.8 (-21.6 to 23.3)
	Male	1400 (1120 to 1750)	15.7 (12.5 to 19.5)	9.4 (-13.8 to 38)	844 (679 to 1040)	9.4 (7.5 to 11.5)	1.7 (-18.3 to 25.8)	20400 (16200 to 25400)	228.3 (181 to 284.2)	0.9 (-20.5 to 26)
	Female	196 (153 to 244)	2 (1.5 to 2.5)	0.8 (-20.9 to 27)	118 (93 to 146)	1.2 (0.9 to 1.4)	-6.2 (-24.5 to 16.2)	2620 (2070 to 3290)	27 (21.4 to 34)	-7.1 (-26.8 to 16.9)
Cyprus	Both	40 (32 to 48)	2 (1.6 to 2.5)	-9.8 (-26.8 to 12.3)	16 (13 to 19)	0.8 (0.7 to 1)	-17.5 (-30.4 to -1.4)	358 (303 to 422)	18.7 (15.8 to 22)	-16.4 (-29.2 to -0.1)
	Male	37 (29 to 45)	4 (3.2 to 4.9)	-12.3 (-29.7 to 9.9)	14 (12 to 17)	1.6 (1.3 to 1.9)	-22.5 (-34.8 to -6.3)	325 (271 to 386)	35.5 (29.8 to 41.9)	-18.2 (-31.4 to -1.5)
	Female	3 (2 to 3)	0.3 (0.2 to 0.3)	-15 (-32.4 to 7.4)	1 (1 to 2)	0.1 (0.1 to 0.2)	-16 (-29.3 to 1.8)	32 (27 to 39)	3.4 (2.8 to 4.1)	-18.3 (-31.9 to -0.9)
Czechia	Both	533 (423 to 665)	2.8 (2.2 to 3.5)	-11 (-29.5 to 12.9)	267 (215 to 330)	1.4 (1.1 to 1.7)	-16 (-32.5 to 5)	6760 (5410 to 8460)	36.8 (29.3 to 46.3)	-18.3 (-35.2 to 3.2)
	Male	470 (374 to 593)	5.3 (4.2 to 6.6)	-14.6 (-33.2 to 8.3)	242 (193 to 299)	2.7 (2.1 to 3.3)	-19 (-35.4 to 0.9)	6170 (4920 to 7720)	70.5 (55.5 to 88.3)	-21 (-37.5 to -0.1)
	Female	63 (46 to 82)	0.6 (0.5 to 0.8)	4.8 (-20.1 to 37.3)	26 (19 to 32)	0.2 (0.2 to 0.3)	-2.6 (-23.9 to 22.9)	597 (438 to 767)	6.2 (4.5 to 7.9)	-3.4 (-25 to 24.6)
Côte d'Ivoire	Both	166 (122 to 215)	1.6 (1.2 to 2)	-7.6 (-26.6 to 15.9)	155 (115 to 199)	1.6 (1.2 to 2)	-8.2 (-26.3 to 13.9)	4340 (3150 to 5710)	37.3 (27.6 to 48)	-9.4 (-29.3 to 16.2)
	Male	154 (112 to 200)	2.9 (2.1 to 3.6)	-8.2 (-27.2 to 15.2)	144 (105 to 186)	2.8 (2.2 to 3.6)	-8.7 (-26.8 to 13.9)	4040 (2900 to 5320)	66.2 (48.4 to 85.5)	-10.1 (-30.2 to 15.8)
	Female	12 (9 to 16)	0.2 (0.2 to 0.3)	-7.4 (-29.6 to 22.3)	11 (8 to 15)	0.2 (0.2 to 0.3)	-8 (-29.1 to 21.6)	309 (222 to 413)	5.5 (4.1 to 7.3)	-9.6 (-32.3 to 19.1)

North Korea	Both	453 (353 to 577)	1.4 (1.1 to 1.7)	-3.9 (-20.3 to 14.1)	321 (256 to 400)	1 (0.8 to 1.2)	-8.5 (-22.8 to 7.8)	8860 (6790 to 11400)	26.5 (20.5 to 34.1)	-8.3 (-25.1 to 10)
	Male	371 (280 to 482)	2.6 (2 to 3.4)	-6.3 (-23 to 13)	255 (197 to 328)	1.9 (1.5 to 2.4)	-10.9 (-25.9 to 6.5)	7250 (5400 to 9550)	48.7 (37.4 to 62.9)	-10.4 (-26.9 to 7.9)
	Female	82 (61 to 110)	0.4 (0.3 to 0.6)	-9.7 (-27.9 to 15.4)	66 (50 to 88)	0.4 (0.3 to 0.5)	-13.5 (-30.5 to 8.7)	1620 (1180 to 2210)	8.9 (6.4 to 12.2)	-12.8 (-31.6 to 11.8)
Democratic Republic of the Congo	Both	447 (333 to 593)	1.2 (0.9 to 1.6)	-2.4 (-21.8 to 21.8)	421 (314 to 555)	1.2 (0.9 to 1.5)	-4.1 (-22.9 to 18.4)	12300 (9090 to 16600)	30.4 (22.7 to 40.1)	-4.4 (-24.4 to 20.2)
	Male	367 (265 to 497)	2.2 (1.7 to 2.9)	-3.9 (-24.1 to 21.7)	346 (252 to 468)	2.2 (1.7 to 2.9)	-5.3 (-24.6 to 19.4)	10200 (7250 to 14000)	55.1 (40 to 73.9)	-5.9 (-27 to 21)
	Female	80 (59 to 107)	0.4 (0.3 to 0.5)	-7 (-27.2 to 19.8)	75 (55 to 100)	0.4 (0.3 to 0.5)	-8.4 (-28.6 to 17.8)	2190 (1570 to 2970)	10 (7.2 to 13.4)	-9.5 (-30.5 to 17.5)
Denmark	Both	276 (213 to 358)	2.6 (2 to 3.4)	-14.1 (-34.1 to 11.7)	102 (92 to 113)	0.9 (0.8 to 1)	-20.2 (-28.1 to -11.5)	2360 (2130 to 2630)	23.1 (20.8 to 25.8)	-21.4 (-29.7 to -11.9)
	Male	229 (174 to 299)	4.5 (3.4 to 5.9)	-15.5 (-35.8 to 10.9)	83 (73 to 92)	1.6 (1.4 to 1.8)	-21.8 (-30.7 to -12.7)	1930 (1700 to 2160)	38.9 (34.6 to 43.6)	-22.7 (-32 to -12.6)
	Female	47 (35 to 63)	0.9 (0.7 to 1.2)	-11.9 (-34.8 to 20)	20 (16 to 23)	0.3 (0.3 to 0.4)	-18.6 (-30.1 to -6.1)	439 (366 to 522)	8.3 (7 to 9.9)	-18 (-30 to -4.5)
Djibouti	Both	10 (7 to 14)	1.5 (1.1 to 2.1)	-5.5 (-25.7 to 20.5)	9 (6 to 12)	1.4 (1 to 1.9)	-8.3 (-27.5 to 16.7)	264 (178 to 386)	36.2 (25.6 to 52.5)	-10 (-30.9 to 16)
	Male	8 (5 to 12)	2.2 (1.6 to 3.3)	-6.9 (-26.8 to 18.3)	7 (5 to 11)	2.1 (1.5 to 3.1)	-9.7 (-28.6 to 13.6)	220 (144 to 326)	55.2 (37.7 to 81.9)	-10.8 (-31.4 to 15.2)
	Female	2 (1 to 2)	0.5 (0.4 to 0.7)	-4.6 (-27.7 to 29.2)	1 (1 to 2)	0.5 (0.4 to 0.7)	-7.8 (-29.6 to 24.4)	44 (29 to 66)	13 (8.9 to 18.8)	-9.2 (-33.7 to 25.5)
Dominica	Both	3 (2 to 3)	2.9 (2.2 to 3.6)	4.2 (-15.8 to 30.3)	2 (2 to 3)	2.4 (1.9 to 3)	5.1 (-14.3 to 30)	52 (40 to 65)	57.8 (45.1 to 72.4)	4.4 (-16.1 to 31.2)
	Male	2 (2 to 3)	5 (3.9 to 6.4)	2.3 (-18.8 to 29.2)	2 (1 to 2)	4.3 (3.3 to 5.4)	2.2 (-18.3 to 27.8)	46 (35 to 58)	101.3 (77.7 to 128.5)	3.4 (-18.2 to 32.1)
	Female	0 (0 to 0)	0.7 (0.6 to 0.9)	-0.8 (-20.7 to 25.6)	0 (0 to 0)	0.6 (0.5 to 0.8)	0.2 (-19.2 to 25.6)	6 (5 to 8)	13.5 (10.6 to 17.1)	-0.2 (-19.9 to 26.2)
Dominican Republic	Both	231 (174 to 304)	2.5 (1.9 to 3.2)	26.7 (-10.1 to 76.2)	190 (145 to 245)	2.1 (1.6 to 2.7)	21.8 (-11 to 67.8)	4810 (3570 to 6370)	50.1 (37.5 to 66.2)	25.8 (-11.3 to 77.3)
	Male	190 (142 to 253)	4.2 (3.2 to 5.6)	30.8 (-7.8 to 85.1)	155 (117 to 204)	3.5 (2.7 to 4.6)	25.8 (-10.3 to 74.9)	3970 (2940 to 5370)	85.3 (63.6 to 115)	30 (-9.3 to 86.4)
	Female	41 (31 to 55)	0.9 (0.7 to 1.1)	15.4 (-15.9 to 60.7)	35 (27 to 45)	0.7 (0.6 to 1)	10.9 (-17.5 to 52.1)	835 (615 to 1120)	16.9 (12.5 to 22.7)	13.1 (-19.4 to 58.6)
Ecuador	Both	132 (103 to 172)	0.9 (0.7 to 1.2)	-1.1 (-24.8 to 29.4)	108 (84 to 140)	0.7 (0.6 to 1)	-6.1 (-27.4 to 21.6)	2410 (1850 to 3150)	15.7 (12.2 to 20.6)	-9.6 (-31.8 to 19.4)
	Male	107 (81 to 142)	1.5 (1.2 to 2)	-0.5 (-25 to 32.2)	88 (68 to 115)	1.3 (1 to 1.7)	-5.2 (-28.3 to 24.4)	1950 (1480 to 2590)	26.7 (20.3 to 35.5)	-9.2 (-32.5 to 22.1)

	Female	25 (19 to 33)	0.3 (0.2 to 0.4)	0.6 (-22.8 to 31.7)	20 (16 to 26)	0.3 (0.2 to 0.3)	-4.9 (-26.3 to 23.7)	462 (350 to 601)	5.7 (4.4 to 7.5)	-8.1 (-31.2 to 20.8)
Egypt	Both	912 (676 to 1250)	1.3 (1 to 1.8)	-1.2 (-24.9 to 28.9)	663 (492 to 926)	1 (0.8 to 1.4)	-8.5 (-29.6 to 18.2)	20300 (14900 to 27500)	27.2 (20.1 to 37.4)	-9.2 (-31.4 to 19.2)
	Male	756 (544 to 1060)	2 (1.5 to 2.9)	-3.1 (-27.2 to 28.3)	555 (398 to 802)	1.5 (1.1 to 2.3)	-10.6 (-32.4 to 17)	16800 (12000 to 23200)	42.3 (30.4 to 59.2)	-10.7 (-33.2 to 18.1)
	Female	156 (112 to 214)	0.5 (0.3 to 0.6)	3.5 (-23.8 to 35.2)	108 (79 to 148)	0.4 (0.3 to 0.5)	-5.3 (-28.8 to 23.5)	3520 (2530 to 4850)	9.9 (7.1 to 13.5)	-4.7 (-30.7 to 26.9)
El Salvador	Both	60 (45 to 78)	1 (0.8 to 1.3)	7.5 (-19.6 to 40.9)	46 (35 to 60)	0.8 (0.6 to 1)	1.3 (-23.6 to 31.1)	1080 (806 to 1430)	18.5 (13.7 to 24.5)	0.7 (-25.3 to 32.5)
	Male	47 (35 to 62)	1.9 (1.4 to 2.5)	12.7 (-16.8 to 48.7)	37 (28 to 48)	1.5 (1.1 to 1.9)	5.7 (-21.6 to 38.6)	859 (630 to 1140)	34.6 (25.3 to 45.9)	6.2 (-21.7 to 41.8)
	Female	12 (9 to 17)	0.4 (0.3 to 0.5)	-1.2 (-26.5 to 30.3)	10 (7 to 12)	0.3 (0.2 to 0.4)	-5.9 (-28.7 to 21.6)	224 (163 to 300)	6.6 (4.8 to 8.8)	-9.3 (-33.3 to 20.2)
Equatorial Guinea	Both	6 (4 to 9)	1.3 (0.9 to 1.9)	10.7 (-13.5 to 42.9)	5 (4 to 8)	1.2 (0.8 to 1.8)	7.3 (-15 to 35.9)	145 (94 to 225)	27.8 (18.7 to 42.8)	5.9 (-18.7 to 40)
	Male	5 (3 to 8)	2.5 (1.7 to 3.9)	14.2 (-11.1 to 46.3)	4 (3 to 7)	2.4 (1.6 to 3.7)	10.3 (-12.4 to 39.7)	119 (76 to 188)	55.1 (36.7 to 86.4)	9.7 (-15.8 to 43.9)
	Female	1 (1 to 2)	0.4 (0.2 to 0.6)	-6.4 (-31.6 to 30.8)	1 (1 to 1)	0.3 (0.2 to 0.5)	-9.1 (-32.5 to 25.3)	26 (16 to 42)	8.4 (5.3 to 13.1)	-12.1 (-37.8 to 27.4)
Eritrea	Both	45 (31 to 62)	1.4 (1 to 2)	-5.3 (-21.2 to 15.4)	41 (29 to 57)	1.4 (1 to 1.9)	-7.2 (-22.3 to 12.3)	1340 (922 to 1880)	39.2 (27.7 to 53.9)	-8.4 (-24.4 to 12.7)
	Male	33 (23 to 47)	2.5 (1.7 to 3.4)	-9 (-25.2 to 12.3)	30 (21 to 43)	2.4 (1.7 to 3.3)	-10.7 (-26.1 to 10.2)	1000 (669 to 1460)	65.9 (45.2 to 92.5)	-12.1 (-28.6 to 9.8)
	Female	12 (8 to 16)	0.7 (0.5 to 1)	-1.7 (-24.5 to 24.7)	11 (7 to 15)	0.7 (0.5 to 0.9)	-3.5 (-25.5 to 22.5)	338 (228 to 484)	18.4 (12.7 to 25.8)	-4.7 (-27.1 to 22.6)
Estonia	Both	89 (67 to 116)	3.9 (2.9 to 5.1)	1 (-25.1 to 32)	32 (24 to 41)	1.3 (1 to 1.7)	-13 (-34.4 to 12.1)	819 (620 to 1060)	37 (28.1 to 47.9)	-13.3 (-35.1 to 13)
	Male	85 (64 to 112)	8.8 (6.6 to 11.5)	-2.8 (-27.6 to 26.6)	30 (23 to 38)	3.1 (2.4 to 4)	-16.3 (-36.6 to 7.7)	769 (580 to 997)	80.6 (61.1 to 104.3)	-16.3 (-37.4 to 8.7)
	Female	4 (3 to 5)	0.3 (0.2 to 0.4)	-9.5 (-33.8 to 20.3)	2 (2 to 3)	0.1 (0.1 to 0.2)	-19.4 (-39.6 to 5.2)	50 (38 to 67)	3.9 (2.9 to 5.3)	-19.6 (-41.2 to 6.6)
eSwatini	Both	14 (11 to 20)	2.4 (1.8 to 3.2)	-14.1 (-30.7 to 8.3)	13 (10 to 18)	2.2 (1.6 to 2.9)	-17.6 (-32.9 to 3.1)	390 (278 to 543)	59.9 (43.7 to 82.7)	-18.9 (-35.5 to 5.2)
	Male	12 (9 to 17)	5 (3.8 to 6.6)	-10.3 (-27 to 11.9)	11 (8 to 15)	4.7 (3.6 to 6.1)	-13.7 (-29.5 to 6.8)	336 (243 to 469)	123.6 (92.1 to 167.2)	-16 (-32.9 to 7.5)
	Female	2 (1 to 3)	0.6 (0.4 to 0.9)	-20.6 (-43.1 to 16.2)	2 (1 to 3)	0.6 (0.4 to 0.9)	-22.7 (-43.9 to 11.8)	54 (31 to 85)	14.5 (8.6 to 22.8)	-25.8 (-48.6 to 12.2)
Ethiopia	Both	407 (326 to 546)	1 (0.8 to 1.3)	-9.2 (-25.3 to 12.3)	382 (304 to 514)	0.9 (0.7 to 1.3)	-11.4 (-27.8 to 8.5)	10900 (8600 to 14400)	23.6 (18.8 to 31.5)	-13.2 (-29.8 to 7.2)

	Male	344 (262 to 483)	1.6 (1.2 to 2.2)	-7 (-26.6 to 18.3)	324 (246 to 454)	1.5 (1.2 to 2.2)	-9.2 (-28.7 to 14.3)	9120 (6890 to 12700)	39.1 (29.6 to 54.5)	-11.2 (-30.8 to 13.2)
	Female	64 (50 to 77)	0.3 (0.2 to 0.4)	-8.2 (-25.3 to 13.9)	58 (46 to 69)	0.3 (0.2 to 0.3)	-10.4 (-27 to 7.6)	1750 (1370 to 2130)	7.4 (5.9 to 8.9)	-13.5 (-30.9 to 6.1)
Fiji	Both	6 (5 to 8)	0.9 (0.7 to 1.1)	3.1 (-18.9 to 30.7)	5 (4 to 7)	0.8 (0.6 to 1)	1.6 (-19 to 27.7)	136 (103 to 176)	17.6 (13.6 to 22.4)	-1.7 (-23.2 to 25.6)
	Male	5 (4 to 7)	1.8 (1.3 to 2.2)	8.4 (-14.3 to 39)	4 (3 to 6)	1.7 (1.3 to 2.1)	6.4 (-15 to 35.1)	113 (84 to 146)	32.9 (25 to 41.9)	3.9 (-18.9 to 34.4)
	Female	1 (1 to 1)	0.2 (0.2 to 0.3)	-16.8 (-38.5 to 12.8)	1 (1 to 1)	0.2 (0.1 to 0.3)	-18.9 (-39.1 to 8.2)	23 (17 to 32)	5.4 (4 to 7.2)	-19.5 (-40.5 to 9)
Finland	Both	159 (120 to 206)	1.5 (1.1 to 1.9)	-6.7 (-30.1 to 24.2)	45 (41 to 51)	0.4 (0.3 to 0.4)	-11.7 (-21 to -1.3)	1040 (916 to 1170)	10 (8.8 to 11.3)	-13.6 (-24.1 to -1.7)
	Male	135 (100 to 176)	2.7 (2 to 3.5)	-9.2 (-32.9 to 20.8)	40 (35 to 45)	0.7 (0.7 to 0.8)	-14.9 (-24.7 to -3.8)	909 (798 to 1030)	18.3 (16.1 to 20.9)	-15.8 (-26.8 to -3.3)
	Female	24 (18 to 32)	0.4 (0.3 to 0.6)	-3 (-28.1 to 28.9)	6 (4 to 7)	0.1 (0.1 to 0.1)	-7.2 (-19.7 to 8)	128 (103 to 155)	2.4 (2 to 2.9)	-7.9 (-20.5 to 8.3)
France	Both	4930 (3740 to 6460)	4.3 (3.3 to 5.7)	-2.2 (-26.4 to 28.6)	1810 (1630 to 1990)	1.5 (1.3 to 1.6)	-8.1 (-16.8 to 2.4)	44300 (39600 to 49400)	40.1 (35.8 to 45)	-9.5 (-18.8 to 2.2)
	Male	4470 (3360 to 5880)	8.3 (6.2 to 11)	-3.5 (-27.8 to 27.6)	1580 (1420 to 1760)	2.8 (2.5 to 3.1)	-10.1 (-19.5 to 1)	39000 (34700 to 44100)	74.5 (66.1 to 84.3)	-10.9 (-21.4 to 2.1)
	Female	467 (323 to 615)	0.8 (0.5 to 1)	5 (-21.2 to 40.4)	228 (175 to 265)	0.3 (0.2 to 0.4)	-1.4 (-13.6 to 13.6)	5230 (3720 to 6170)	9.2 (6.5 to 10.9)	-1.6 (-15 to 15.8)
Gabon	Both	21 (15 to 28)	1.9 (1.4 to 2.6)	-3.9 (-22.5 to 20.8)	18 (13 to 25)	1.7 (1.3 to 2.4)	-6.7 (-24.4 to 16.8)	510 (368 to 687)	44 (32.3 to 59.2)	-9.4 (-28.7 to 17.1)
	Male	19 (13 to 25)	3.7 (2.7 to 5.1)	-3.6 (-22.3 to 20.2)	16 (12 to 22)	3.4 (2.6 to 4.8)	-6.5 (-23.8 to 16.6)	460 (326 to 628)	83.4 (60.4 to 113.6)	-8.4 (-27.5 to 17.2)
	Female	2 (1 to 3)	0.4 (0.3 to 0.5)	-12.4 (-34.3 to 15.6)	2 (1 to 3)	0.3 (0.2 to 0.5)	-14.8 (-35.2 to 10.6)	50 (34 to 72)	8.3 (5.8 to 11.8)	-17.6 (-40.2 to 11)
The Gambia	Both	7 (5 to 8)	0.7 (0.6 to 0.9)	5.1 (-15.8 to 31.8)	6 (5 to 8)	0.7 (0.5 to 0.9)	3.4 (-16.2 to 29)	172 (131 to 221)	16.9 (13 to 21.2)	2.9 (-19.1 to 31.3)
	Male	6 (4 to 7)	1.3 (1 to 1.6)	5.3 (-16.6 to 34.1)	6 (4 to 7)	1.3 (1 to 1.6)	3.5 (-17 to 30.8)	147 (111 to 190)	29.7 (22.8 to 37.4)	3.3 (-19.1 to 33.3)
	Female	1 (1 to 1)	0.2 (0.1 to 0.3)	8.4 (-18.5 to 43.5)	1 (1 to 1)	0.2 (0.1 to 0.2)	6.1 (-19.6 to 39.1)	25 (18 to 34)	4.6 (3.3 to 6.3)	6.3 (-22.1 to 42.7)
Georgia	Both	261 (211 to 318)	4.6 (3.8 to 5.6)	2.1 (-19.1 to 27.5)	200 (164 to 243)	3.5 (2.8 to 4.2)	-1.1 (-21.2 to 21.4)	5400 (4370 to 6600)	98.7 (80.2 to 120.7)	-1.9 (-22.7 to 22.6)
	Male	246 (198 to 301)	10.1 (8.2 to 12.3)	3.5 (-18.3 to 29.7)	189 (153 to 229)	7.7 (6.3 to 9.4)	0.5 (-20.3 to 24.4)	5130 (4120 to 6300)	212.2 (171.9 to 258.9)	-0.8 (-22.4 to 24.2)
	Female	14 (11 to 23)	0.5 (0.4 to 0.7)	-21.9 (-38.3 to -0.2)	12 (9 to 19)	0.3 (0.3 to 0.5)	-24.3 (-40.4 to -3.7)	271 (212 to 439)	9 (7 to 14.3)	-25.5 (-42.2 to -4.6)

Germany	Both	3520 (2740 to 4610)	2.1 (1.6 to 2.8)	-7.3 (-29.2 to 23)	1670 (1530 to 1830)	0.9 (0.9 to 1)	-11.9 (-19.5 to -2.6)	38200 (34900 to 41900)	23.7 (21.6 to 25.9)	-14.3 (-22.1 to -5)
	Male	3090 (2390 to 4070)	3.9 (3 to 5.1)	-9.2 (-30.6 to 21)	1440 (1310 to 1580)	1.7 (1.6 to 1.9)	-14.3 (-22.5 to -4.9)	33000 (29800 to 36500)	42.6 (38.4 to 46.9)	-16.3 (-24.9 to -6.4)
	Female	436 (311 to 570)	0.5 (0.4 to 0.7)	-0.4 (-25.1 to 32.1)	235 (174 to 269)	0.2 (0.2 to 0.3)	-4.6 (-15.7 to 8.4)	5220 (3760 to 5940)	6.4 (4.6 to 7.3)	-5.2 (-16.9 to 8.1)
Ghana	Both	261 (201 to 335)	1.6 (1.3 to 2.1)	0.5 (-18.3 to 25)	237 (183 to 301)	1.5 (1.2 to 1.9)	-2.4 (-20.4 to 20.4)	6430 (4880 to 8360)	37.2 (28.8 to 47.8)	-3.3 (-22.5 to 22.4)
	Male	240 (183 to 310)	3.5 (2.7 to 4.4)	5.6 (-14.8 to 33.3)	217 (167 to 278)	3.3 (2.6 to 4.1)	2.6 (-16.4 to 27.6)	5900 (4460 to 7720)	77.6 (59.4 to 100.1)	1.7 (-18.8 to 29.7)
	Female	22 (16 to 28)	0.2 (0.2 to 0.3)	-5.4 (-24.8 to 22.6)	19 (15 to 25)	0.2 (0.2 to 0.3)	-7.8 (-26.3 to 19.2)	525 (391 to 690)	5.4 (4.1 to 7)	-9.9 (-29.3 to 17.6)
Greece	Both	897 (693 to 1140)	4.3 (3.3 to 5.4)	-9.2 (-30.9 to 16.6)	407 (372 to 441)	1.7 (1.6 to 1.9)	-8.4 (-15.7 to -0.7)	8300 (7600 to 9060)	41 (37.5 to 44.8)	-9.4 (-17.3 to -0.9)
	Male	833 (641 to 1060)	8.5 (6.5 to 10.8)	-9.6 (-31.4 to 17)	368 (334 to 401)	3.4 (3.1 to 3.7)	-8.5 (-16.1 to -0.2)	7570 (6890 to 8310)	79.8 (72.4 to 87.6)	-10 (-18.2 to -0.8)
	Female	64 (48 to 82)	0.6 (0.4 to 0.7)	-2.5 (-24.7 to 26.8)	39 (31 to 45)	0.3 (0.2 to 0.3)	-3.3 (-13 to 7.8)	722 (621 to 814)	6.7 (5.9 to 7.5)	-2.1 (-12.2 to 9.9)
Greenland	Both	2 (1 to 2)	2.1 (1.7 to 2.6)	-9.2 (-25.3 to 9.7)	1 (1 to 1)	1.5 (1.1 to 1.8)	-14 (-30.1 to 4.2)	28 (21 to 35)	36.4 (28.3 to 44.9)	-16.3 (-32.6 to 2.3)
	Male	1 (1 to 2)	3.7 (2.9 to 4.5)	-9.1 (-25.6 to 9.5)	1 (1 to 1)	2.6 (2.1 to 3.2)	-14.1 (-30.9 to 4.4)	26 (20 to 33)	63.1 (48.9 to 78.1)	-15.5 (-32.4 to 4.5)
	Female	0 (0 to 0)	0.3 (0.2 to 0.4)	-17.2 (-35.1 to 5.9)	0 (0 to 0)	0.2 (0.2 to 0.2)	-22.9 (-39.9 to -2.3)	2 (1 to 2)	5.1 (4 to 6.5)	-23.8 (-41.3 to -3.1)
Grenada	Both	2 (2 to 2)	1.6 (1.4 to 1.8)	-20.8 (-32.3 to -6.9)	1 (1 to 2)	1.2 (1.1 to 1.4)	-24.5 (-34.8 to -12.2)	36 (31 to 41)	30.3 (26.3 to 34.7)	-23.7 (-35.8 to -9.9)
	Male	2 (1 to 2)	2.9 (2.6 to 3.4)	-31.4 (-41.7 to -19)	1 (1 to 1)	2.4 (2.1 to 2.7)	-36.3 (-45.3 to -26)	32 (27 to 37)	55.1 (47.9 to 63.4)	-31.8 (-42.5 to -19.1)
	Female	0 (0 to 0)	0.4 (0.3 to 0.5)	-16.7 (-31.7 to 4.8)	0 (0 to 0)	0.3 (0.3 to 0.4)	-19.7 (-34.1 to 0.5)	4 (3 to 5)	7.1 (5.9 to 8.6)	-20.2 (-35.2 to 0.2)
Guam	Both	1 (1 to 1)	0.5 (0.4 to 0.7)	-28.7 (-43.5 to -6)	1 (1 to 1)	0.4 (0.3 to 0.5)	-28.9 (-43.4 to -7.2)	17 (14 to 22)	9.1 (7.3 to 11.7)	-28 (-42.7 to -5.9)
	Male	1 (1 to 1)	0.9 (0.7 to 1.2)	-33.4 (-47.6 to -7.3)	1 (0 to 1)	0.7 (0.5 to 0.9)	-33.6 (-47.6 to -7.4)	13 (11 to 17)	14.5 (11.5 to 18.8)	-32.9 (-47.6 to -6.8)
	Female	0 (0 to 0)	0.2 (0.2 to 0.3)	-5.7 (-27.8 to 21.8)	0 (0 to 0)	0.1 (0.1 to 0.2)	-3.5 (-25.3 to 24.6)	4 (3 to 5)	4.1 (3.2 to 5.2)	-6.7 (-28 to 20.8)
Guatemala	Both	91 (72 to 116)	0.8 (0.7 to 1)	-15.3 (-35.9 to 11.6)	81 (64 to 101)	0.8 (0.6 to 0.9)	-16.6 (-36.4 to 7.4)	1990 (1560 to 2520)	16.9 (13.3 to 21.5)	-17.9 (-38.8 to 8.9)
	Male	65 (51 to 83)	1.3 (1 to 1.7)	-14.4 (-36.8 to 13.3)	58 (46 to 74)	1.2 (1 to 1.6)	-15.2 (-36.4 to 11)	1400 (1080 to 1780)	26.7 (20.7 to 33.9)	-17.2 (-39.4 to 10.5)

	Female	26 (20 to 35)	0.4 (0.3 to 0.6)	-13.8 (-34.5 to 15.4)	22 (17 to 29)	0.4 (0.3 to 0.5)	-16.1 (-35.2 to 9.2)	590 (455 to 783)	9 (7 to 11.9)	-16.2 (-36.6 to 11)
Guinea	Both	74 (54 to 99)	1.4 (1 to 1.8)	1.2 (-18.5 to 27.9)	73 (53 to 96)	1.4 (1 to 1.8)	0 (-19 to 23.5)	1890 (1350 to 2530)	33.1 (23.8 to 44.1)	-1.5 (-22.2 to 24.1)
	Male	66 (47 to 89)	2.4 (1.7 to 3.2)	-0.3 (-20.8 to 26.4)	65 (46 to 87)	2.4 (1.8 to 3.2)	-1.7 (-21.5 to 22.5)	1690 (1170 to 2290)	58.3 (40.8 to 78.4)	-2.4 (-23.9 to 24.3)
	Female	8 (6 to 10)	0.3 (0.2 to 0.4)	-2.1 (-24 to 23.4)	8 (5 to 10)	0.3 (0.2 to 0.4)	-2.7 (-23.3 to 22.6)	205 (146 to 274)	7 (5 to 9.3)	-4.2 (-25.6 to 21.3)
Guinea-Bissau	Both	14 (10 to 18)	1.9 (1.3 to 2.4)	-6.7 (-24.9 to 16.6)	13 (9 to 17)	1.8 (1.3 to 2.3)	-7.2 (-25.1 to 15.5)	371 (257 to 487)	46.6 (32.4 to 60.5)	-8.8 (-27.8 to 15.6)
	Male	12 (8 to 16)	3.8 (2.7 to 4.8)	-6.2 (-24.4 to 17.6)	12 (8 to 15)	3.7 (2.6 to 4.7)	-6.6 (-23.9 to 16.8)	333 (223 to 444)	91.8 (63 to 119.3)	-8.2 (-27.2 to 17.6)
	Female	1 (1 to 2)	0.3 (0.3 to 0.5)	-7.8 (-28.7 to 19.2)	1 (1 to 2)	0.3 (0.2 to 0.4)	-8.4 (-28.4 to 17.8)	38 (27 to 50)	8.5 (6.2 to 11.1)	-11.4 (-31.8 to 16.3)
Guyana	Both	9 (7 to 12)	1.4 (1.1 to 1.8)	0.3 (-25.6 to 32.6)	8 (6 to 10)	1.2 (0.9 to 1.6)	-3.4 (-27.9 to 27.4)	223 (165 to 293)	32.1 (24 to 41.8)	-3.3 (-28.8 to 30)
	Male	8 (6 to 11)	2.7 (2 to 3.5)	1.5 (-25 to 34.3)	7 (5 to 9)	2.4 (1.8 to 3)	-1.9 (-27.2 to 29.6)	201 (148 to 265)	60.6 (45.1 to 79.3)	-2.2 (-28.7 to 32.6)
	Female	1 (1 to 1)	0.3 (0.2 to 0.4)	1.4 (-25.2 to 36.5)	1 (1 to 1)	0.3 (0.2 to 0.3)	-2.5 (-26.4 to 29.4)	23 (17 to 30)	6.3 (4.7 to 8.3)	-2.4 (-28.7 to 32.7)
Haiti	Both	174 (103 to 252)	2.5 (1.5 to 3.5)	0.3 (-19.4 to 25.2)	165 (98 to 237)	2.5 (1.5 to 3.4)	-1.3 (-19.9 to 22.5)	4530 (2650 to 6610)	59.9 (35.4 to 86.8)	-1.4 (-21.2 to 24.4)
	Male	152 (88 to 219)	4.6 (2.7 to 6.6)	2.9 (-18.8 to 33.2)	144 (84 to 208)	4.6 (2.7 to 6.5)	1.2 (-19.9 to 29.1)	3930 (2250 to 5730)	110.9 (64.6 to 160.1)	1.4 (-21 to 32.5)
	Female	23 (14 to 33)	0.6 (0.4 to 0.9)	-6.7 (-27.8 to 21.9)	21 (13 to 30)	0.6 (0.4 to 0.8)	-8.1 (-28.1 to 18.2)	601 (373 to 891)	14.5 (9.1 to 21.3)	-9.4 (-29.7 to 17.2)
Honduras	Both	87 (66 to 108)	1.5 (1.1 to 1.8)	12.4 (-8.3 to 41.5)	76 (58 to 94)	1.3 (1 to 1.6)	8.5 (-9.8 to 34.3)	1860 (1410 to 2370)	29.9 (22.9 to 37.6)	6.8 (-14.7 to 36.3)
	Male	62 (46 to 77)	2.2 (1.6 to 2.7)	18.9 (-8.8 to 59.8)	54 (40 to 67)	2 (1.5 to 2.4)	15.8 (-10.4 to 52.2)	1330 (981 to 1680)	45.3 (33.7 to 57)	12.3 (-16.2 to 52.2)
	Female	25 (19 to 33)	0.8 (0.6 to 1.1)	2.6 (-20 to 31.2)	22 (17 to 29)	0.8 (0.6 to 1)	-2.6 (-23.7 to 24.6)	535 (392 to 723)	16.2 (12 to 21.5)	-1.2 (-24.6 to 29)
Hungary	Both	899 (723 to 1110)	5.4 (4.3 to 6.7)	-19.5 (-36.2 to 1.3)	488 (398 to 594)	2.8 (2.3 to 3.4)	-25.1 (-39.8 to -7)	13300 (10800 to 16500)	82.2 (65.8 to 102.2)	-26.9 (-41.6 to -8.2)
	Male	773 (618 to 955)	10.3 (8.2 to 12.8)	-22 (-38.6 to -1.9)	424 (345 to 518)	5.6 (4.5 to 6.8)	-27.1 (-41.5 to -9.3)	11700 (9350 to 14400)	157 (124.7 to 195.6)	-28.9 (-43.8 to -10.6)
	Female	126 (93 to 160)	1.4 (1 to 1.8)	-11.4 (-32.1 to 12.5)	64 (48 to 79)	0.6 (0.5 to 0.8)	-19.4 (-37.1 to 0.7)	1690 (1200 to 2120)	19.3 (13.3 to 24.3)	-19.3 (-38.3 to 0.8)
Iceland	Both	7 (6 to 9)	1.4 (1.2 to 1.7)	-2.2 (-18.7 to 17.9)	3 (2 to 3)	0.5 (0.4 to 0.5)	-8.7 (-20.3 to 5.2)	60 (53 to 70)	11.9 (10.4 to 13.8)	-5.4 (-18.4 to 9.9)

	Male	6 (5 to 8)	2.5 (2.1 to 3)	-3.4 (-20.8 to 17.7)	2 (2 to 2)	0.8 (0.7 to 0.9)	-10.9 (-23.6 to 4)	50 (43 to 58)	20 (17.2 to 23.3)	-6.3 (-20.3 to 11)
	Female	1 (1 to 1)	0.4 (0.3 to 0.5)	-1.2 (-22.1 to 23.9)	0 (0 to 1)	0.2 (0.1 to 0.2)	-6.1 (-20.8 to 11)	11 (8 to 13)	4.2 (3.4 to 5)	-5 (-20.5 to 14.7)
India	Both	34800 (28700 to 41800)	2.9 (2.4 to 3.5)	-1.2 (-18.1 to 19.3)	29800 (25000 to 35600)	2.6 (2.2 to 3.1)	-5.7 (-21.8 to 13.3)	840000 (705000 to 1010000)	68.5 (57.5 to 82.2)	-6.8 (-23.1 to 12.6)
	Male	29400 (23600 to 36200)	5.1 (4.1 to 6.3)	-2.2 (-20.8 to 20.2)	25200 (20300 to 30700)	4.5 (3.7 to 5.5)	-6.4 (-25 to 14.9)	708000 (574000 to 865000)	117.3 (94.9 to 143.1)	-8 (-26.7 to 13.7)
	Female	5430 (4350 to 6670)	0.9 (0.7 to 1.1)	10.8 (-10.6 to 38.9)	4610 (3660 to 5730)	0.8 (0.6 to 1)	5.4 (-15.4 to 30.3)	133000 (104000 to 166000)	21.3 (16.8 to 26.5)	4.7 (-16.2 to 30.4)
Indonesia	Both	2590 (1950 to 3640)	1.2 (0.9 to 1.7)	0.9 (-18.2 to 22.6)	2130 (1640 to 2850)	1.1 (0.8 to 1.4)	-3.8 (-21.3 to 15.5)	55500 (42500 to 74300)	24.2 (18.7 to 32.3)	-6 (-24.3 to 15.4)
	Male	2130 (1510 to 3170)	2.1 (1.5 to 3.1)	3.5 (-19.1 to 30.4)	1730 (1250 to 2470)	1.8 (1.4 to 2.6)	-1.4 (-22.7 to 23.7)	45000 (32400 to 64600)	41.2 (29.9 to 58.5)	-3.8 (-25.5 to 24.4)
	Female	466 (369 to 585)	0.4 (0.3 to 0.5)	-8.3 (-27.5 to 15.6)	405 (321 to 497)	0.4 (0.3 to 0.5)	-10.8 (-28.8 to 10.7)	10500 (8170 to 13200)	8.9 (7 to 10.9)	-14.1 (-33.2 to 9.6)
Iran	Both	2280 (2030 to 2530)	3.1 (2.8 to 3.4)	13.6 (6.6 to 28.1)	1450 (1320 to 1610)	2.1 (1.9 to 2.3)	-1.2 (-7 to 10.6)	37100 (34100 to 41100)	48.1 (44.2 to 53.3)	-2.2 (-7.8 to 8.8)
	Male	1760 (1560 to 1980)	4.8 (4.3 to 5.4)	7.8 (-0.3 to 22.4)	1130 (1020 to 1270)	3.2 (2.9 to 3.6)	-6.2 (-12.5 to 6.1)	28800 (26000 to 32500)	75.1 (68 to 84.5)	-7 (-13.2 to 4.9)
	Female	517 (467 to 569)	1.4 (1.3 to 1.6)	44.3 (32.8 to 59.7)	322 (296 to 352)	0.9 (0.8 to 1)	26.4 (18.2 to 40)	8240 (7650 to 8950)	21.3 (19.8 to 23.3)	22.4 (14.5 to 33.8)
Iraq	Both	750 (563 to 948)	3.2 (2.4 to 3.9)	6.3 (-14.7 to 32.2)	530 (404 to 661)	2.4 (1.9 to 2.9)	-2.2 (-20.4 to 20.3)	14800 (11000 to 19000)	58.4 (44.2 to 73.4)	-7.3 (-25.8 to 14.6)
	Male	562 (425 to 707)	5 (3.8 to 6.2)	8.3 (-14.7 to 36.5)	405 (310 to 504)	3.9 (3 to 4.7)	0.4 (-19.7 to 25.6)	10900 (8190 to 13800)	90.5 (69.1 to 113.1)	-5.6 (-26.5 to 19.7)
	Female	188 (137 to 252)	1.5 (1.1 to 1.9)	2.6 (-21.8 to 36.5)	124 (92 to 163)	1 (0.8 to 1.3)	-7.4 (-27.8 to 21.8)	3870 (2780 to 5260)	27.8 (20.4 to 36.9)	-10.3 (-31.8 to 20.7)
Ireland	Both	176 (133 to 232)	2.4 (1.8 to 3.2)	-10.2 (-32.1 to 18.6)	62 (55 to 69)	0.8 (0.7 to 0.9)	-17.7 (-26.5 to -9.1)	1450 (1290 to 1640)	20.3 (18 to 23)	-19.5 (-28.6 to -9.8)
	Male	155 (116 to 205)	4.4 (3.3 to 5.9)	-11 (-33.4 to 17.3)	52 (46 to 59)	1.5 (1.3 to 1.7)	-19 (-28.6 to -9.2)	1240 (1080 to 1410)	35.7 (31.1 to 40.5)	-20.2 (-30.3 to -9.2)

	Female	21 (15 to 28)	0.6 (0.4 to 0.8)	-8.4 (-32 to 20.5)	9 (8 to 12)	0.2 (0.2 to 0.3)	-17 (-28.9 to -1.4)	215 (180 to 263)	5.9 (4.9 to 7.2)	-17 (-29.9 to -1.9)
Israel	Both	201 (153 to 259)	1.8 (1.4 to 2.3)	-7.4 (-30.2 to 20.4)	97 (87 to 107)	0.8 (0.7 to 0.9)	-12.7 (-21.5 to -3.2)	2080 (1860 to 2300)	18.9 (16.9 to 21)	-14.3 (-23.5 to -4.5)
	Male	180 (136 to 233)	3.5 (2.6 to 4.5)	-10 (-32.5 to 18.1)	84 (74 to 93)	1.6 (1.4 to 1.8)	-15.3 (-24.9 to -4.6)	1810 (1600 to 2020)	35.2 (31 to 39.4)	-16.9 (-27.1 to -6.4)
	Female	21 (16 to 28)	0.4 (0.3 to 0.5)	6.4 (-20.2 to 39)	13 (11 to 16)	0.2 (0.2 to 0.2)	-0.1 (-12.7 to 15)	266 (229 to 315)	4.5 (3.9 to 5.3)	-0.3 (-12.8 to 14.8)
Italy	Both	4060 (3190 to 5070)	3.3 (2.5 to 4.1)	-12.4 (-31.3 to 10.1)	1720 (1590 to 1830)	1.2 (1.1 to 1.3)	-15.3 (-18.8 to -11.1)	35700 (33500 to 38000)	29 (27.4 to 31)	-16.5 (-20.2 to -12)
	Male	3580 (2710 to 4570)	6.2 (4.7 to 7.9)	-14.4 (-35.1 to 10.3)	1540 (1430 to 1640)	2.5 (2.3 to 2.6)	-18.1 (-21.6 to -13.8)	32100 (30100 to 34200)	55.8 (52.7 to 59.5)	-18.3 (-22.4 to -13.7)
	Female	483 (363 to 619)	0.7 (0.5 to 0.9)	-5.6 (-27.6 to 23)	178 (147 to 204)	0.2 (0.2 to 0.3)	-10.6 (-16.2 to -1.8)	3620 (3120 to 4140)	5.6 (5 to 6.4)	-10.5 (-16.5 to -1.7)
Jamaica	Both	50 (38 to 64)	1.7 (1.3 to 2.2)	-7.4 (-29.6 to 19.5)	38 (29 to 49)	1.3 (1 to 1.7)	-7.1 (-29.1 to 18)	913 (696 to 1180)	31 (23.6 to 40.3)	-6.9 (-29.2 to 21.3)
	Male	46 (35 to 60)	3.3 (2.5 to 4.2)	-9 (-31.6 to 17.7)	36 (27 to 46)	2.5 (1.9 to 3.2)	-9 (-30.9 to 17.7)	849 (645 to 1110)	58.9 (44.6 to 76.8)	-8.4 (-30.9 to 20)
	Female	4 (3 to 5)	0.2 (0.2 to 0.3)	14.9 (-11.6 to 48.9)	3 (2 to 4)	0.2 (0.1 to 0.2)	16.3 (-9.5 to 49)	64 (49 to 86)	4.2 (3.2 to 5.6)	14.3 (-12.4 to 49)
Japan	Both	4650 (3800 to 5610)	1.5 (1.2 to 1.8)	-12.5 (-27.9 to 5.7)	1210 (1070 to 1300)	0.3 (0.3 to 0.3)	-14.9 (-18.1 to -10)	22500 (20400 to 24100)	7.2 (6.7 to 7.8)	-16.2 (-20 to -11.4)
	Male	4140 (3330 to 5070)	2.8 (2.2 to 3.4)	-14.7 (-31.9 to 5.6)	1070 (951 to 1140)	0.6 (0.6 to 0.7)	-17.5 (-20.9 to -12.4)	19800 (18100 to 21300)	13.5 (12.4 to 14.5)	-18.3 (-22.7 to -13.1)
	Female	514 (395 to 653)	0.3 (0.3 to 0.4)	-6.1 (-24.5 to 17.3)	144 (112 to 172)	0.1 (0.1 to 0.1)	-11.9 (-16.2 to -4.7)	2690 (2270 to 3140)	1.8 (1.6 to 2.1)	-10.9 (-15.6 to -3.7)
Jordan	Both	73 (56 to 96)	1.1 (0.8 to 1.5)	-11.5 (-32.2 to 14.2)	45 (35 to 59)	0.7 (0.6 to 1)	-21.2 (-39.1 to 0.2)	1230 (946 to 1610)	17 (13.1 to 22.4)	-21.3 (-40.6 to 2)
	Male	64 (47 to 86)	1.8 (1.4 to 2.5)	-14.3 (-36.4 to 13.4)	40 (29 to 53)	1.2 (0.9 to 1.7)	-23.1 (-42.9 to 0.5)	1080 (793 to 1450)	28.4 (21.1 to 38.5)	-24.1 (-44.1 to 1)
	Female	9 (7 to 12)	0.3 (0.2 to 0.4)	6.5 (-17.4 to 36)	5 (4 to 7)	0.2 (0.1 to 0.2)	-3.6 (-24.9 to 20.5)	152 (116 to 197)	4.4 (3.4 to 5.6)	-6.9 (-28 to 18.4)
Kazakhstan	Both	381 (323 to 447)	2 (1.7 to 2.4)	-14.6 (-28 to 0.2)	269 (228 to 314)	1.5 (1.3 to 1.7)	-23.8 (-35.7 to -10.8)	7600 (6400 to 8900)	39.8 (33.7 to 46.7)	-25.1 (-37.3 to -12.3)
	Male	341 (287 to 400)	4.4 (3.7 to 5.2)	-16.5 (-30.3 to -1.5)	242 (205 to 284)	3.3 (2.8 to 3.9)	-24.8 (-36.9 to -12.2)	6830 (5720 to 8020)	84.6 (71.6 to 99.1)	-26.4 (-38.6 to -13.2)
	Female	40 (33 to 54)	0.4 (0.3 to 0.5)	-8 (-23.8 to 14.5)	27 (22 to 35)	0.3 (0.2 to 0.3)	-19 (-32.8 to 0.5)	776 (636 to 1020)	7.4 (6 to 9.8)	-21.4 (-35.2 to -1.7)

Kenya	Both	354 (274 to 474)	1.5 (1.2 to 2)	-8.3 (-25.1 to 11.1)	326 (252 to 445)	1.4 (1.1 to 1.9)	-10.5 (-25.6 to 5.8)	9950 (7690 to 13400)	38.1 (29.5 to 51.9)	-12.5 (-27.8 to 4.1)
	Male	306 (231 to 422)	2.7 (2.1 to 3.7)	-10.1 (-28.7 to 10.7)	280 (209 to 395)	2.6 (1.9 to 3.6)	-12 (-28.6 to 6.2)	8520 (6350 to 12100)	67.8 (50.8 to 95.6)	-13.8 (-30.8 to 6)
	Female	48 (37 to 63)	0.4 (0.3 to 0.5)	0 (-20.8 to 29.2)	46 (35 to 60)	0.4 (0.3 to 0.5)	-4.7 (-23.8 to 17.4)	1430 (1070 to 1870)	10.3 (7.8 to 13.4)	-6.7 (-26.7 to 17.1)
Kiribati	Both	1 (0 to 1)	0.8 (0.6 to 0.9)	-2.5 (-17 to 14.1)	0 (0 to 1)	0.7 (0.6 to 0.9)	-2.5 (-16.6 to 13.4)	13 (10 to 17)	17.7 (13.9 to 22.5)	-4.1 (-19.3 to 14)
	Male	0 (0 to 0)	1.4 (1 to 1.7)	1.2 (-17 to 22.4)	0 (0 to 0)	1.4 (1.1 to 1.8)	1.2 (-17 to 22.3)	9 (7 to 12)	29.6 (22.5 to 38.1)	0 (-18.5 to 21.7)
	Female	0 (0 to 0)	0.4 (0.3 to 0.5)	-8.1 (-28 to 17.3)	0 (0 to 0)	0.4 (0.3 to 0.5)	-8.5 (-27.9 to 16.4)	4 (3 to 6)	9.8 (7.3 to 12.8)	-9.8 (-29.2 to 16.5)
Kuwait	Both	30 (24 to 37)	1.2 (1 to 1.6)	4 (-18.2 to 29.8)	15 (12 to 18)	0.7 (0.6 to 0.9)	-7.1 (-25.3 to 14.3)	369 (290 to 454)	14.1 (11.2 to 17.5)	-11.3 (-29.7 to 11.1)
	Male	26 (20 to 33)	1.8 (1.4 to 2.3)	11.3 (-14.8 to 42.1)	13 (10 to 17)	1.1 (0.8 to 1.3)	-1.2 (-22.9 to 24.6)	317 (242 to 403)	20.8 (16.1 to 26.4)	-4.7 (-27.2 to 22)
	Female	4 (3 to 6)	0.4 (0.3 to 0.5)	-22.4 (-42 to 5.3)	2 (1 to 2)	0.2 (0.1 to 0.2)	-33.5 (-49.8 to -10.8)	52 (38 to 75)	4.1 (3.1 to 5.8)	-33.9 (-50.1 to -10.8)
Kyrgyzstan	Both	51 (43 to 60)	1 (0.9 to 1.2)	-13 (-27.1 to 4.6)	39 (33 to 47)	0.8 (0.7 to 1)	-17.5 (-30.5 to -1.5)	1140 (964 to 1370)	22.2 (18.8 to 26.4)	-18.8 (-32.4 to -2.5)
	Male	44 (37 to 53)	2.1 (1.7 to 2.5)	-14.9 (-29.7 to 3.2)	34 (29 to 41)	1.7 (1.4 to 2)	-19 (-32.2 to -2.2)	995 (830 to 1200)	43.8 (36.8 to 52.6)	-20.4 (-34.3 to -3.2)
	Female	7 (5 to 8)	0.2 (0.2 to 0.3)	1.9 (-18 to 26.6)	5 (4 to 6)	0.2 (0.2 to 0.2)	-4.9 (-23.5 to 18.8)	149 (119 to 184)	5.2 (4.2 to 6.4)	-5.7 (-25.4 to 17.7)
Laos	Both	57 (41 to 78)	1.3 (1 to 1.8)	-1.7 (-19.2 to 21.8)	51 (37 to 69)	1.2 (0.9 to 1.6)	-5.5 (-21.2 to 16.5)	1370 (960 to 1870)	29.2 (20.6 to 39.7)	-8 (-24.8 to 15.6)
	Male	46 (32 to 66)	2.2 (1.6 to 3.1)	-0.5 (-19.6 to 26.2)	41 (29 to 58)	2.1 (1.5 to 2.8)	-4.5 (-22.4 to 19.7)	1090 (743 to 1560)	48.5 (33.6 to 68.8)	-6.7 (-25.3 to 19.6)
	Female	11 (8 to 14)	0.5 (0.4 to 0.6)	-8.3 (-27.3 to 17)	10 (7 to 13)	0.5 (0.4 to 0.6)	-10.4 (-29 to 13.3)	278 (202 to 366)	11.2 (8.2 to 14.5)	-14.2 (-32.7 to 11.9)
Latvia	Both	107 (83 to 138)	3.1 (2.4 to 4.1)	-14.6 (-34.7 to 12.2)	69 (53 to 86)	1.9 (1.5 to 2.5)	-21.7 (-39.8 to 0.4)	1780 (1360 to 2290)	54.1 (41.1 to 69.7)	-22.2 (-40.6 to 1.2)
	Male	101 (77 to 131)	7.1 (5.5 to 9.4)	-17.7 (-37.7 to 8.5)	65 (49 to 82)	4.5 (3.5 to 5.8)	-24.2 (-42.3 to -1.8)	1690 (1270 to 2190)	121.2 (91.3 to 157.7)	-24.8 (-43.3 to -1.9)
	Female	6 (4 to 8)	0.3 (0.2 to 0.4)	-11.6 (-35.7 to 21)	4 (3 to 5)	0.2 (0.1 to 0.2)	-20.1 (-40.8 to 6.2)	87 (64 to 116)	4.4 (3.2 to 5.9)	-20.5 (-41.9 to 8.3)
Lebanon	Both	293 (236 to 375)	5.6 (4.6 to 7.2)	6.4 (-13.3 to 29.8)	150 (123 to 192)	2.9 (2.4 to 3.7)	-10.5 (-26.2 to 7.4)	3690 (2980 to 4710)	70.9 (57.2 to 90.7)	-7.8 (-24.9 to 13.6)
	Male	250 (199 to 329)	10.7 (8.5 to 14)	11.1 (-11.7 to 38.2)	130 (106 to 169)	5.6 (4.5 to 7.2)	-5.6 (-23 to 14.7)	3160 (2530 to 4090)	134.2 (107.8 to 174.7)	-4.1 (-23.3 to 19.1)
	Female	43 (32 to 59)	1.5 (1.1 to 2.1)	8 (-17.9 to 41.4)	20 (15 to 28)	0.7 (0.5 to 1)	-9.9 (-29 to 15.4)	530 (399 to 720)	18.7 (14 to 25.4)	-7.4 (-29.5 to 21.2)

Lesotho	Both	39 (29 to 51)	2.9 (2.2 to 3.7)	-2.6 (-21.9 to 21.3)	36 (28 to 48)	2.8 (2.1 to 3.6)	-5.1 (-23.1 to 17.2)	1100 (818 to 1460)	77.5 (58 to 101.9)	-5.7 (-25.5 to 19.8)
	Male	33 (25 to 43)	5.9 (4.6 to 7.5)	-1.3 (-19.5 to 23.4)	31 (23 to 40)	5.7 (4.5 to 7.3)	-3.9 (-21.1 to 19.5)	934 (699 to 1230)	154.6 (117.2 to 201)	-4.7 (-23.9 to 21.1)
	Female	6 (4 to 9)	0.8 (0.5 to 1.2)	-4.5 (-34.4 to 30.9)	6 (4 to 8)	0.8 (0.5 to 1.1)	-6.3 (-34.6 to 28.6)	166 (98 to 245)	20.9 (12.5 to 30.6)	-7.3 (-37.4 to 29.2)
Liberia	Both	25 (18 to 34)	1.3 (0.9 to 1.7)	-6.6 (-25.5 to 18.7)	24 (17 to 32)	1.3 (0.9 to 1.7)	-7.2 (-25.3 to 17.1)	635 (453 to 879)	29.3 (21.3 to 39.4)	-7.3 (-26.9 to 20.1)
	Male	22 (16 to 31)	2.2 (1.6 to 3)	-6.2 (-25.8 to 20.3)	21 (15 to 29)	2.2 (1.6 to 3)	-6.6 (-25.6 to 19)	565 (395 to 792)	50.7 (36.2 to 69.4)	-7.1 (-27.8 to 21.5)
	Female	3 (2 to 4)	0.3 (0.2 to 0.4)	-10.6 (-31.4 to 16.1)	2 (2 to 3)	0.3 (0.2 to 0.3)	-10.8 (-30.9 to 14.5)	70 (49 to 96)	6.3 (4.5 to 8.6)	-12 (-32.9 to 16)
Libya	Both	204 (154 to 265)	3.8 (2.9 to 4.9)	3.7 (-20.8 to 34.4)	141 (108 to 184)	2.8 (2.1 to 3.6)	4.2 (-19.7 to 34.6)	4050 (3010 to 5310)	70.2 (52.6 to 91.9)	4.8 (-20.7 to 37.2)
	Male	178 (133 to 235)	6.5 (4.9 to 8.6)	5 (-21.1 to 38.6)	124 (94 to 164)	4.8 (3.7 to 6.3)	5.2 (-20.6 to 36.4)	3530 (2610 to 4700)	120.6 (89.9 to 160.6)	6.1 (-20.6 to 41.5)
	Female	26 (19 to 35)	1 (0.7 to 1.3)	-0.8 (-25 to 31)	17 (13 to 23)	0.7 (0.5 to 0.8)	0.1 (-23 to 31.6)	517 (377 to 701)	17.7 (13 to 23.7)	0.1 (-24.3 to 31.7)
Lithuania	Both	166 (132 to 207)	3.3 (2.6 to 4.2)	-19.5 (-37 to 1.6)	115 (92 to 142)	2.2 (1.8 to 2.8)	-23 (-39 to -3.9)	3030 (2410 to 3790)	63.3 (50.2 to 80)	-24.6 (-41 to -5)
	Male	156 (123 to 195)	7.5 (5.9 to 9.3)	-21.2 (-38.2 to -0.7)	109 (86 to 135)	5.2 (4.1 to 6.5)	-24.3 (-39.9 to -5.8)	2870 (2270 to 3610)	140.5 (110.8 to 177.4)	-26.1 (-42.1 to -7)
	Female	10 (8 to 13)	0.4 (0.3 to 0.5)	-3.7 (-26 to 24.1)	7 (5 to 8)	0.2 (0.2 to 0.3)	-10.4 (-30.4 to 13.8)	158 (121 to 200)	5.7 (4.3 to 7.3)	-10.4 (-31.3 to 15.1)
Luxembourg	Both	26 (21 to 32)	2.7 (2.2 to 3.4)	-19.4 (-36.3 to 1.1)	9 (8 to 11)	1 (0.8 to 1.1)	-20.8 (-31.2 to -7.8)	236 (204 to 275)	25.2 (21.8 to 29.4)	-21.9 (-32.9 to -8.9)
	Male	23 (18 to 29)	4.9 (3.9 to 6.2)	-21.7 (-38.3 to -1.5)	8 (7 to 9)	1.7 (1.5 to 2)	-24.4 (-34.8 to -11.6)	201 (173 to 237)	43.6 (37.4 to 51.3)	-24.5 (-35.7 to -10.7)
	Female	3 (2 to 4)	0.6 (0.5 to 0.8)	-18.4 (-36.8 to 7.3)	2 (1 to 2)	0.3 (0.2 to 0.3)	-16.9 (-29.8 to 2.2)	35 (28 to 43)	7.3 (6 to 9.1)	-17 (-30 to 1.8)
Madagascar	Both	124 (90 to 167)	1 (0.8 to 1.4)	-6.6 (-27.7 to 17.6)	115 (84 to 154)	1 (0.7 to 1.3)	-7.3 (-27.7 to 16.2)	3630 (2600 to 4900)	26.9 (19.7 to 36)	-7.9 (-29.2 to 17.9)
	Male	91 (64 to 126)	1.6 (1.1 to 2.1)	-7.2 (-28.9 to 18.2)	85 (60 to 117)	1.5 (1.1 to 2.1)	-7.8 (-29.3 to 16.5)	2630 (1830 to 3660)	40.6 (29 to 55.8)	-8.3 (-31 to 19.1)
	Female	33 (24 to 45)	0.5 (0.4 to 0.7)	-5.3 (-28.7 to 23.8)	30 (22 to 41)	0.5 (0.4 to 0.7)	-6.1 (-28.9 to 21.4)	996 (721 to 1380)	13.7 (10.1 to 18.9)	-7.2 (-31.2 to 22.2)
Malawi	Both	42 (33 to 54)	0.6 (0.4 to 0.7)	-8.1 (-25.6 to 12.9)	39 (31 to 50)	0.5 (0.4 to 0.7)	-8.8 (-25.5 to 11.4)	1160 (883 to 1520)	14.1 (11 to 18.3)	-9.9 (-28.9 to 13.3)
	Male	32 (25 to 42)	1 (0.8 to 1.2)	-8.7 (-26.8 to 13.9)	30 (23 to 39)	0.9 (0.8 to 1.2)	-9.3 (-26.5 to 12.6)	878 (664 to 1180)	23.7 (18.4 to 31.1)	-10.6 (-29.6 to 13.6)
	Female	10 (8 to 14)	0.2 (0.2 to 0.3)	-5.7 (-28.9 to 21.6)	10 (7 to 13)	0.2 (0.2 to 0.3)	-6.6 (-29 to 20.7)	282 (201 to 391)	6.2 (4.5 to 8.5)	-7.6 (-31.1 to 22.5)

Malaysia	Both	534 (406 to 681)	2 (1.5 to 2.5)	28.1 (-1.7 to 64.8)	331 (254 to 422)	1.3 (1 to 1.6)	13.7 (-11.5 to 45.1)	8320 (6300 to 10700)	29.9 (22.8 to 38.1)	15.9 (-10.7 to 48)
	Male	462 (346 to 596)	3.4 (2.6 to 4.4)	29 (-2.7 to 68.3)	283 (212 to 365)	2.2 (1.7 to 2.8)	13.9 (-12.3 to 48.1)	7090 (5340 to 9260)	50.9 (38.5 to 66.3)	16.7 (-11.5 to 51.3)
	Female	72 (54 to 93)	0.5 (0.4 to 0.7)	17.8 (-10.4 to 55.8)	48 (37 to 63)	0.4 (0.3 to 0.5)	4.8 (-19.6 to 36.5)	1240 (924 to 1630)	8.8 (6.7 to 11.5)	9.2 (-17.1 to 43.7)
Maldives	Both	4 (3 to 5)	1.4 (1.1 to 1.7)	5.4 (-16.1 to 31.9)	2 (2 to 3)	0.8 (0.7 to 1)	-9.3 (-27.7 to 13.4)	55 (45 to 69)	17.3 (14 to 21.5)	-6.9 (-25.6 to 16.8)
	Male	4 (3 to 5)	2.4 (1.9 to 3)	7.1 (-15.8 to 34.5)	2 (2 to 3)	1.5 (1.2 to 1.8)	-6.2 (-25.4 to 17.4)	52 (42 to 65)	30 (24.1 to 37.4)	-6.9 (-26.3 to 18.3)
	Female	0 (0 to 0)	0.2 (0.1 to 0.2)	-1.9 (-23.8 to 25.2)	0 (0 to 0)	0.1 (0.1 to 0.1)	-12.5 (-31.8 to 11.4)	3 (3 to 4)	2.3 (1.8 to 2.9)	-13.2 (-32.6 to 10.5)
Mali	Both	92 (70 to 123)	1 (0.8 to 1.3)	-0.1 (-18.2 to 22.4)	86 (65 to 113)	1 (0.8 to 1.3)	-1.6 (-18.8 to 19.8)	2480 (1860 to 3330)	25.8 (19.6 to 34.6)	-1.2 (-21.1 to 23)
	Male	71 (52 to 97)	1.6 (1.2 to 2.1)	0.3 (-19.6 to 26.6)	67 (49 to 90)	1.5 (1.1 to 2)	-1.3 (-20.3 to 23.4)	1890 (1350 to 2610)	38.7 (28.2 to 52.8)	-0.6 (-22.3 to 28.1)
	Female	21 (16 to 28)	0.5 (0.4 to 0.6)	-2.7 (-23.1 to 23.9)	19 (14 to 25)	0.4 (0.3 to 0.6)	-4.1 (-23.6 to 20)	587 (429 to 805)	12 (8.9 to 16.2)	-4.5 (-25.9 to 22.2)
Malta	Both	22 (18 to 27)	2.6 (2.2 to 3.2)	-2.6 (-20.3 to 19.1)	9 (8 to 10)	1 (0.9 to 1.1)	-13.7 (-25.2 to -0.4)	207 (178 to 240)	26.2 (22.3 to 30.3)	-11.1 (-23.5 to 3.5)
	Male	20 (16 to 24)	5 (4 to 6.1)	-5.3 (-22.7 to 16.4)	8 (7 to 9)	1.9 (1.6 to 2.2)	-16.6 (-27.9 to -2.7)	183 (156 to 214)	47.7 (40.3 to 55.8)	-13.2 (-26.2 to 1.7)
	Female	2 (2 to 3)	0.5 (0.4 to 0.6)	-3 (-22.8 to 22.7)	1 (1 to 1)	0.2 (0.2 to 0.3)	-12.9 (-27.2 to 3.1)	23 (19 to 29)	5.9 (4.9 to 7.2)	-11.7 (-27.3 to 5.2)
Marshall Islands	Both	0 (0 to 1)	1.3 (0.9 to 1.7)	-3.7 (-19.4 to 17.8)	0 (0 to 0)	1.2 (0.9 to 1.6)	-5.3 (-20.5 to 14.5)	10 (7 to 14)	27.8 (19.6 to 36.9)	-7.1 (-23.5 to 14.4)
	Male	0 (0 to 0)	1.9 (1.3 to 2.6)	-3 (-20.4 to 19.8)	0 (0 to 0)	1.9 (1.3 to 2.5)	-5.1 (-22.1 to 17.2)	8 (5 to 11)	41 (27.3 to 55.9)	-5.8 (-23.9 to 17.8)
	Female	0 (0 to 0)	0.5 (0.4 to 0.7)	-8.7 (-29.5 to 15.4)	0 (0 to 0)	0.5 (0.4 to 0.7)	-10.5 (-29.7 to 12.7)	3 (2 to 4)	13.4 (9.5 to 18.4)	-11.4 (-32.1 to 12.4)
Mauritania	Both	20 (14 to 27)	1 (0.7 to 1.3)	-0.7 (-23.3 to 23.1)	18 (13 to 25)	0.9 (0.7 to 1.2)	-3.4 (-24.7 to 18.3)	446 (304 to 624)	20.9 (14.6 to 28.5)	-6.7 (-29.7 to 17.5)
	Male	17 (12 to 24)	1.7 (1.2 to 2.3)	0.4 (-23.1 to 25.6)	16 (11 to 22)	1.6 (1.2 to 2.2)	-2.7 (-24.2 to 21.1)	386 (257 to 551)	36.2 (24.7 to 50.9)	-5.2 (-29.2 to 20.8)
	Female	3 (2 to 3)	0.2 (0.2 to 0.3)	-7.4 (-31.8 to 18.4)	2 (2 to 3)	0.2 (0.2 to 0.3)	-10.4 (-33.1 to 13.9)	60 (42 to 82)	5.4 (3.9 to 7.4)	-13.3 (-37.6 to 11.7)
Mauritius	Both	32 (25 to 40)	1.8 (1.4 to 2.2)	-0.3 (-21.3 to 23.5)	19 (15 to 24)	1.1 (0.9 to 1.4)	-8.2 (-26.6 to 12.8)	494 (388 to 617)	27.2 (21.4 to 33.9)	-8.7 (-27.7 to 13.2)
	Male	30 (23 to 37)	3.6 (2.8 to 4.4)	1.6 (-20.2 to 26.6)	18 (14 to 22)	2.3 (1.8 to 2.8)	-6.2 (-24.7 to 15.8)	448 (351 to 558)	52.9 (41.5 to 65.8)	-6.4 (-26.1 to 16.5)
	Female	3 (2 to 5)	0.3 (0.2 to 0.6)	-27.9 (-43.7 to 0.7)	2 (1 to 4)	0.2 (0.2 to 0.4)	-32.5 (-46.8 to -6.3)	46 (35 to 89)	4.9 (3.8 to 9.6)	-32.9 (-47.7 to -7.4)

Mexico	Both	1490 (1240 to 1790)	1.3 (1.1 to 1.5)	-5.3 (-20.5 to 12.2)	1120 (926 to 1330)	1 (0.8 to 1.2)	-10.8 (-24.4 to 4.9)	25500 (21300 to 30700)	21.6 (18 to 25.9)	-9.7 (-24.8 to 6.9)
	Male	1280 (1030 to 1560)	2.4 (1.9 to 2.9)	-3.9 (-21.1 to 16.6)	957 (775 to 1160)	1.8 (1.5 to 2.2)	-9.4 (-25.5 to 9.6)	21800 (17600 to 26700)	39.7 (32.2 to 48.3)	-8.5 (-25.5 to 11.6)
	Female	216 (174 to 297)	0.3 (0.3 to 0.5)	-7.3 (-23.1 to 10)	162 (131 to 223)	0.3 (0.2 to 0.4)	-12 (-26.6 to 5.2)	3710 (3000 to 5410)	5.9 (4.8 to 8.4)	-11.6 (-26.8 to 6.7)
Federatead States of Micronesia	Both	1 (1 to 1)	1.2 (0.9 to 1.6)	2.6 (-18.3 to 28)	1 (0 to 1)	1.1 (0.8 to 1.4)	-1.5 (-21 to 22.2)	20 (13 to 28)	26.5 (18.1 to 35.1)	-2.1 (-24.9 to 23.3)
	Male	1 (0 to 1)	2.1 (1.5 to 2.8)	2.4 (-20.3 to 29.9)	1 (0 to 1)	1.9 (1.4 to 2.5)	-1.3 (-22.2 to 23.9)	15 (9 to 21)	43 (28.5 to 58.1)	-2.6 (-28.4 to 25.1)
	Female	0 (0 to 0)	0.5 (0.4 to 0.8)	2.8 (-21.6 to 33.4)	0 (0 to 0)	0.5 (0.3 to 0.6)	-0.8 (-23.6 to 27)	5 (4 to 8)	12.7 (9 to 17.8)	-1.5 (-25.7 to 29.4)
Monaco	Both	8 (6 to 10)	10 (7.8 to 12.4)	-6.6 (-26.7 to 18.1)	3 (2 to 3)	3.2 (2.6 to 4)	-10.3 (-28 to 11.2)	67 (52 to 82)	87.8 (68.4 to 109.6)	-10.5 (-29.6 to 13.1)
	Male	7 (6 to 9)	19.1 (15 to 23.8)	-6.8 (-26.9 to 18.6)	3 (2 to 3)	6.2 (4.9 to 7.6)	-10.7 (-28.6 to 11.7)	61 (48 to 74)	164.4 (127.8 to 205.2)	-10.5 (-30.2 to 13.5)
	Female	1 (0 to 1)	1.4 (1 to 1.9)	-10.2 (-33.7 to 20.7)	0 (0 to 0)	0.6 (0.4 to 0.8)	-13 (-34.9 to 14.6)	6 (4 to 8)	15.6 (11.3 to 20.6)	-12.8 (-35 to 17.1)
Mongolia	Both	34 (26 to 45)	1.7 (1.3 to 2.2)	-3.4 (-23.9 to 21.3)	31 (24 to 41)	1.7 (1.2 to 2.2)	-4.6 (-24.2 to 19.7)	749 (572 to 983)	34.4 (26.2 to 44.5)	-8.3 (-27.9 to 17.4)
	Male	30 (23 to 39)	3.9 (2.7 to 5)	-0.6 (-21.4 to 24.6)	27 (21 to 35)	4 (2.9 to 5.1)	-2.3 (-22.8 to 21.7)	648 (485 to 854)	73 (54.6 to 94.7)	-4.6 (-25.4 to 22.1)
	Female	5 (3 to 6)	0.4 (0.3 to 0.6)	-19.7 (-41.1 to 8.9)	4 (3 to 6)	0.4 (0.3 to 0.5)	-22.1 (-42.6 to 6.3)	100 (74 to 135)	8.3 (6.1 to 11.4)	-22.7 (-43.1 to 5.6)
Montenegro	Both	82 (67 to 101)	8.5 (6.9 to 10.4)	-0.8 (-18.7 to 21.5)	46 (38 to 57)	4.7 (3.9 to 5.8)	-7.5 (-23.7 to 13.7)	1240 (1010 to 1540)	130 (104.8 to 161.1)	-9.4 (-26.1 to 11.9)
	Male	64 (52 to 81)	14.1 (11.3 to 17.7)	0.7 (-19.2 to 26.3)	37 (30 to 46)	8.2 (6.6 to 10.2)	-5.2 (-23.5 to 18.6)	995 (792 to 1260)	218.5 (173.3 to 275.1)	-8.1 (-26.1 to 15.6)
	Female	18 (14 to 22)	3.6 (2.9 to 4.4)	-6.6 (-25.1 to 15.9)	10 (8 to 11)	1.8 (1.5 to 2.2)	-14.9 (-29.9 to 2.2)	248 (200 to 301)	51 (41.2 to 62.3)	-14.6 (-31.6 to 5.4)
Morocco	Both	1230 (874 to 1570)	3.7 (2.7 to 4.7)	15.8 (-9.7 to 53)	948 (686 to 1190)	3 (2.2 to 3.7)	8.5 (-14.6 to 41.9)	26100 (18400 to 33800)	76.2 (54.6 to 96.8)	4.7 (-18.4 to 37)
	Male	1130 (798 to 1450)	6.9 (5 to 8.8)	16.8 (-9.7 to 56.5)	876 (628 to 1100)	5.6 (4.1 to 7)	10.2 (-13.8 to 47)	24000 (16700 to 31100)	140.9 (99.8 to 180.4)	5.4 (-18.9 to 40.5)
	Female	97 (73 to 128)	0.6 (0.4 to 0.7)	0.5 (-23.7 to 33.6)	72 (55 to 93)	0.4 (0.3 to 0.6)	-8.8 (-29.6 to 19.7)	2140 (1560 to 2880)	12.2 (9 to 16)	-9.3 (-31 to 21)
Mozambique	Both	210 (159 to 272)	1.8 (1.4 to 2.3)	-2.7 (-23.4 to 20.8)	196 (150 to 253)	1.7 (1.3 to 2.2)	-4.3 (-23.9 to 18)	6020 (4460 to 7850)	47 (35.7 to 60.9)	-4.5 (-27 to 19.8)

	Male	173 (132 to 225)	3.3 (2.5 to 4.2)	-2.5 (-23.2 to 20.6)	162 (124 to 210)	3.2 (2.5 to 4.1)	-3.9 (-23.7 to 18.1)	4960 (3730 to 6480)	85.9 (65.7 to 112)	-4.4 (-27 to 20.1)
	Female	37 (25 to 53)	0.6 (0.4 to 0.8)	0.7 (-27 to 36.8)	34 (23 to 49)	0.5 (0.4 to 0.8)	-0.7 (-27.5 to 35.5)	1060 (679 to 1510)	14.8 (9.8 to 21.1)	-1.2 (-29.9 to 38.1)
Myanmar	Both	557 (446 to 732)	1.2 (1 to 1.6)	-5.1 (-21.9 to 15.2)	477 (386 to 631)	1.1 (0.9 to 1.4)	-10.8 (-26.2 to 7.4)	12400 (9750 to 16300)	25.4 (20.1 to 33.5)	-13 (-29.3 to 6.1)
	Male	434 (337 to 588)	2.2 (1.7 to 2.9)	0.1 (-19.4 to 23.7)	366 (288 to 488)	1.9 (1.5 to 2.5)	-6.4 (-23.5 to 15)	9600 (7440 to 13000)	44.7 (35.2 to 59.9)	-8.4 (-26.5 to 14.1)
	Female	123 (94 to 161)	0.5 (0.4 to 0.6)	-14.8 (-31.5 to 5.2)	111 (86 to 144)	0.4 (0.3 to 0.6)	-18 (-34.2 to 1.8)	2810 (2140 to 3720)	10.4 (7.9 to 13.7)	-21.8 (-38.5 to -0.5)
Namibia	Both	46 (35 to 61)	3.1 (2.4 to 4.1)	3.1 (-17.3 to 27.4)	39 (30 to 51)	2.8 (2.2 to 3.5)	-2.9 (-20.8 to 18.5)	1150 (857 to 1540)	74.4 (56.6 to 98.2)	-3.6 (-24.3 to 21.8)
	Male	38 (29 to 50)	6.1 (4.8 to 7.8)	5.7 (-13.8 to 29.6)	33 (25 to 42)	5.4 (4.2 to 6.8)	-0.2 (-18 to 22)	953 (718 to 1270)	141.6 (108.8 to 184.3)	-1.5 (-21.3 to 23.6)
	Female	8 (5 to 12)	1 (0.7 to 1.4)	-1.7 (-29.6 to 35.1)	7 (5 to 10)	0.9 (0.6 to 1.2)	-7.5 (-33.2 to 26.1)	194 (128 to 288)	22.5 (14.9 to 33.4)	-8.9 (-36.5 to 28.2)
Nauru	Both	0 (0 to 0)	1.6 (1.2 to 2)	-13.4 (-28.6 to 7.5)	0 (0 to 0)	1.3 (1 to 1.7)	-20.3 (-33.9 to -0.7)	1 (1 to 2)	30.5 (23.4 to 38.9)	-22.1 (-36.1 to -3.1)
	Male	0 (0 to 0)	2.7 (2 to 3.6)	-5.7 (-24 to 19)	0 (0 to 0)	2.3 (1.7 to 2.9)	-14.3 (-30.5 to 7.4)	1 (1 to 2)	50.9 (36.3 to 67)	-16 (-32.7 to 6.8)
	Female	0 (0 to 0)	0.6 (0.4 to 0.8)	-8.7 (-29.7 to 21.8)	0 (0 to 0)	0.5 (0.3 to 0.7)	-16.7 (-35.5 to 11.1)	0 (0 to 1)	13 (9.2 to 18)	-18.2 (-37.1 to 10.7)
Nepal	Both	619 (467 to 835)	2.7 (2.1 to 3.6)	15.3 (-7.7 to 41)	554 (421 to 737)	2.5 (1.9 to 3.3)	11.5 (-10.5 to 35.5)	14800 (11100 to 20300)	62.7 (47.1 to 84.6)	9.2 (-13.4 to 34.6)
	Male	489 (357 to 679)	4.5 (3.3 to 6.1)	20.7 (-5.4 to 51.4)	442 (324 to 606)	4.2 (3.1 to 5.7)	16.7 (-8 to 44.7)	11600 (8400 to 16300)	103.9 (75.3 to 145.3)	14.7 (-11.4 to 44.8)
	Female	129 (94 to 171)	1.1 (0.8 to 1.4)	8.6 (-14.5 to 38.1)	112 (82 to 148)	1 (0.7 to 1.2)	4.6 (-17.8 to 32.5)	3200 (2330 to 4260)	25.1 (18.4 to 33.3)	2.6 (-20.2 to 32.6)
Netherlands	Both	733 (557 to 940)	2.3 (1.7 to 2.9)	-6 (-29.9 to 21.5)	267 (242 to 292)	0.8 (0.7 to 0.9)	-10.1 (-18.1 to -1.1)	5950 (5360 to 6560)	18.9 (17.1 to 20.9)	-11.9 (-20.7 to -2.3)
	Male	622 (468 to 806)	4 (3 to 5.2)	-8.3 (-31.9 to 20.2)	216 (195 to 240)	1.4 (1.3 to 1.5)	-13.6 (-22.3 to -3.5)	4820 (4310 to 5360)	31.7 (28.3 to 35.3)	-13.9 (-23.2 to -2.7)
	Female	111 (72 to 146)	0.7 (0.4 to 0.9)	-3.9 (-29.5 to 25.9)	51 (33 to 60)	0.3 (0.2 to 0.3)	-7.8 (-19.7 to 5.7)	1130 (734 to 1330)	7.2 (4.7 to 8.4)	-9.9 (-21.8 to 3.6)
New Zealand	Both	109 (85 to 139)	1.4 (1.1 to 1.8)	-4.9 (-28.3 to 23.8)	37 (33 to 40)	0.5 (0.4 to 0.5)	-10.4 (-18 to -2)	830 (755 to 908)	11.3 (10.4 to 12.3)	-11.3 (-18.8 to -3.1)
	Male	100 (75 to 130)	2.8 (2.1 to 3.6)	-5 (-29.9 to 26.6)	29 (26 to 32)	0.8 (0.7 to 0.9)	-11.3 (-20.4 to -1.2)	650 (586 to 721)	18.6 (16.8 to 20.6)	-11.1 (-20.6 to -1)
	Female	9 (7 to 12)	0.2 (0.2 to 0.3)	-8.4 (-30.1 to 19.1)	8 (7 to 10)	0.2 (0.2 to 0.2)	-10.9 (-21.3 to 1.6)	180 (155 to 213)	4.8 (4.2 to 5.6)	-11.7 (-22.3 to 0.8)

Nicaragua	Both	67 (53 to 85)	1.6 (1.2 to 2)	0.8 (-20.9 to 28.1)	52 (41 to 65)	1.3 (1 to 1.6)	-8.4 (-28 to 14.9)	1180 (912 to 1490)	26.6 (20.7 to 33.7)	-6.4 (-26.7 to 19)
	Male	59 (45 to 76)	3.2 (2.5 to 4)	5.9 (-18.1 to 36.5)	45 (35 to 58)	2.6 (2 to 3.2)	-3.3 (-24.4 to 22.5)	1040 (793 to 1330)	52.6 (40.5 to 67.3)	-1.8 (-24.7 to 26.4)
	Female	8 (7 to 10)	0.3 (0.3 to 0.4)	-10.4 (-27.8 to 11.9)	6 (5 to 7)	0.3 (0.2 to 0.3)	-19.1 (-34 to -0.7)	142 (113 to 177)	5.7 (4.6 to 7)	-16.8 (-33.2 to 5)
Niger	Both	80 (58 to 106)	1.1 (0.8 to 1.4)	2.7 (-16.6 to 29.4)	77 (56 to 102)	1.1 (0.8 to 1.4)	1.3 (-17.3 to 26.9)	2070 (1480 to 2760)	25 (18 to 32.8)	2.6 (-17.4 to 30.6)
	Male	70 (49 to 94)	1.9 (1.4 to 2.5)	4.9 (-16 to 33.9)	68 (47 to 90)	2 (1.4 to 2.6)	3.5 (-16.6 to 32.3)	1800 (1260 to 2450)	44.8 (31.6 to 59.5)	4.6 (-17 to 33.6)
	Female	10 (7 to 14)	0.3 (0.2 to 0.3)	0.7 (-21.9 to 29.4)	10 (7 to 13)	0.3 (0.2 to 0.3)	-0.2 (-21.7 to 27.1)	267 (187 to 359)	6.1 (4.3 to 8.1)	-0.5 (-23.5 to 28.5)
Nigeria	Both	1070 (778 to 1490)	1.2 (0.9 to 1.7)	-15.8 (-41.5 to 23.7)	992 (713 to 1380)	1.2 (0.9 to 1.6)	-15.9 (-41.8 to 25.4)	27900 (19600 to 40200)	28.6 (20.5 to 40)	-17.8 (-44.2 to 24.2)
	Male	1020 (724 to 1440)	2.4 (1.8 to 3.4)	-7.3 (-36.7 to 38.8)	946 (669 to 1330)	2.4 (1.7 to 3.3)	-8.3 (-37.5 to 39.6)	26600 (18400 to 38600)	58.4 (41.1 to 83.1)	-8.8 (-39.7 to 41.9)
	Female	50 (35 to 67)	0.1 (0.1 to 0.1)	-4.3 (-33.7 to 36.4)	46 (33 to 63)	0.1 (0.1 to 0.1)	-5.2 (-31.5 to 34.9)	1280 (892 to 1800)	2.5 (1.8 to 3.4)	-5.2 (-32.9 to 41.5)
Niue	Both	0 (0 to 0)	1.1 (0.9 to 1.4)	-3.9 (-17.8 to 11.2)	0 (0 to 0)	0.8 (0.6 to 0.9)	-8.2 (-21.2 to 6)	0 (0 to 0)	17.5 (13.9 to 21.7)	-8.1 (-22 to 7.2)
	Male	0 (0 to 0)	2.1 (1.7 to 2.6)	-2.2 (-17.5 to 15.8)	0 (0 to 0)	1.5 (1.2 to 1.8)	-6 (-21.3 to 11.8)	0 (0 to 0)	30.5 (24.1 to 38.1)	-6.6 (-21.3 to 10.6)
	Female	0 (0 to 0)	0.4 (0.3 to 0.5)	-6.6 (-29.3 to 22.5)	0 (0 to 0)	0.3 (0.2 to 0.4)	-10.4 (-30.9 to 15.2)	0 (0 to 0)	7.1 (5.1 to 9.6)	-10.4 (-32.1 to 18.1)
North Macedonia	Both	183 (139 to 237)	5.6 (4.3 to 7.2)	-8.2 (-29.4 to 17.1)	118 (91 to 151)	3.6 (2.8 to 4.6)	-12.5 (-31.5 to 10.1)	3150 (2390 to 4060)	96 (73.2 to 123.3)	-13.4 (-33.5 to 10.5)
	Male	167 (126 to 216)	10.4 (8 to 13.4)	-9.8 (-31 to 15.8)	109 (83 to 139)	7 (5.4 to 8.9)	-14 (-32.4 to 8.1)	2880 (2180 to 3740)	179.3 (135.8 to 230.7)	-14.7 (-34.6 to 9.7)
	Female	16 (13 to 21)	1 (0.8 to 1.3)	-5.5 (-28.2 to 23)	10 (8 to 12)	0.6 (0.5 to 0.8)	-11 (-31 to 13.8)	263 (201 to 340)	16.3 (12.5 to 21)	-11.4 (-32.3 to 14.8)
Northern Mariana Islands	Both	1 (0 to 1)	1 (0.8 to 1.3)	-37.7 (-48.5 to -18.7)	0 (0 to 0)	0.6 (0.6 to 0.8)	-37.7 (-47.8 to -22.9)	8 (7 to 10)	14.4 (12 to 18.5)	-38.9 (-49.1 to -21.3)
	Male	0 (0 to 0)	1.6 (1.3 to 2)	-36.8 (-49.2 to -17.4)	0 (0 to 0)	1 (0.9 to 1.3)	-36.3 (-47.8 to -19.7)	6 (4 to 7)	21.6 (17.8 to 28.1)	-38.5 (-50.1 to -20.5)
	Female	0 (0 to 0)	0.5 (0.4 to 0.6)	-27.2 (-43.9 to -4.2)	0 (0 to 0)	0.3 (0.2 to 0.4)	-26.7 (-42.6 to -5)	3 (2 to 3)	7.8 (6 to 10)	-28.9 (-45.1 to -6.5)
Norway	Both	138 (111 to 168)	1.5 (1.2 to 1.9)	-12.8 (-29 to 7.7)	40 (37 to 43)	0.4 (0.4 to 0.4)	-14.6 (-18.6 to -7.4)	886 (826 to 965)	10 (9.3 to 10.9)	-17.4 (-21.6 to -9.8)
	Male	121 (95 to 151)	2.8 (2.2 to 3.4)	-14.9 (-32.6 to 7.1)	31 (29 to 34)	0.7 (0.7 to 0.8)	-18 (-22.4 to -10.5)	709 (658 to 781)	16.4 (15.2 to 18)	-20 (-25 to -11.7)

	Female	17 (13 to 22)	0.4 (0.3 to 0.5)	-8.7 (-27.7 to 15)	8 (7 to 9)	0.2 (0.1 to 0.2)	-9.1 (-15.9 to 0.1)	177 (150 to 197)	4 (3.5 to 4.5)	-11.5 (-18.1 to -2.1)
Oman	Both	19 (15 to 24)	1.1 (0.9 to 1.4)	-24.3 (-38 to -8.3)	10 (8 to 12)	0.7 (0.6 to 0.8)	-28.4 (-40.9 to -14.7)	298 (237 to 374)	15.1 (12.4 to 18.3)	-35.6 (-47.5 to -22.1)
	Male	17 (13 to 22)	1.8 (1.5 to 2.2)	-22.5 (-37.9 to -4.2)	9 (7 to 11)	1.2 (1 to 1.4)	-25.4 (-39.5 to -9.4)	261 (202 to 336)	23.9 (19.3 to 29.3)	-34.6 (-47.7 to -19.2)
	Female	3 (2 to 3)	0.3 (0.3 to 0.4)	-20.2 (-36.7 to 0.9)	1 (1 to 2)	0.2 (0.1 to 0.2)	-27 (-41.7 to -8.9)	37 (30 to 45)	4.4 (3.6 to 5.4)	-31.4 (-45.6 to -13.7)
Pakistan	Both	7380 (5640 to 9720)	6.2 (4.8 to 8.1)	-7.5 (-31.6 to 25.7)	6570 (5040 to 8600)	5.7 (4.5 to 7.4)	-10.3 (-32.1 to 21)	200000 (153000 to 265000)	155.1 (119.6 to 203.7)	-10.5 (-33.5 to 22.7)
	Male	6320 (4520 to 8580)	10.3 (7.4 to 14)	-6.9 (-35.6 to 31.8)	5640 (4120 to 7670)	9.6 (7.1 to 12.9)	-9.6 (-34.7 to 26.3)	170000 (124000 to 234000)	257.2 (188 to 350.3)	-9.9 (-35.5 to 29.1)
	Female	1060 (816 to 1400)	1.8 (1.4 to 2.3)	-5.4 (-29.3 to 31.8)	923 (709 to 1220)	1.6 (1.3 to 2.1)	-8.2 (-29.8 to 23.7)	29700 (22200 to 39600)	45.5 (34.8 to 60)	-9 (-32.3 to 24.5)
Palau	Both	0 (0 to 0)	0.9 (0.7 to 1.1)	-1.2 (-19 to 22.9)	0 (0 to 0)	0.6 (0.4 to 0.7)	-5.9 (-22.2 to 16.2)	3 (3 to 5)	14.3 (11 to 18.3)	-5.4 (-22.8 to 18.5)
	Male	0 (0 to 0)	0.8 (0.6 to 1.1)	0.4 (-18.8 to 25.3)	0 (0 to 0)	0.6 (0.4 to 0.7)	-4 (-22.5 to 19)	1 (1 to 2)	12.5 (9.7 to 15.8)	-4 (-21.8 to 19.2)
	Female	0 (0 to 0)	0.9 (0.7 to 1.3)	-2.2 (-25.8 to 32.1)	0 (0 to 0)	0.6 (0.4 to 0.8)	-6.4 (-28.3 to 23.6)	2 (1 to 3)	16.6 (11.9 to 22.6)	-6.4 (-29.1 to 25.8)
Palestine	Both	30 (25 to 36)	1.3 (1.1 to 1.5)	7.5 (-12.3 to 30)	21 (18 to 26)	1 (0.8 to 1.2)	-3.2 (-20.8 to 16.5)	562 (459 to 679)	22.4 (18.5 to 27.1)	-2.9 (-21.2 to 18.2)
	Male	28 (23 to 33)	2.5 (2.1 to 3)	3.1 (-16.7 to 26)	20 (16 to 24)	2 (1.6 to 2.4)	-6.5 (-23.3 to 13.3)	515 (417 to 626)	43.1 (35.5 to 52.2)	-6.6 (-24.4 to 14.5)
	Female	2 (2 to 3)	0.2 (0.2 to 0.2)	7.7 (-15.5 to 37.6)	2 (1 to 2)	0.1 (0.1 to 0.2)	-3.1 (-23.7 to 22.4)	46 (37 to 57)	3.4 (2.8 to 4.2)	-4.4 (-24.4 to 19.6)
Panama	Both	61 (45 to 80)	1.5 (1.1 to 1.9)	-13.9 (-36.4 to 14.9)	43 (32 to 55)	1 (0.8 to 1.3)	-21.4 (-41.1 to 2.6)	983 (726 to 1310)	23.7 (17.5 to 31.5)	-22.3 (-43.1 to 3.4)
	Male	54 (40 to 72)	2.7 (2 to 3.6)	-13.5 (-36.7 to 15.6)	38 (28 to 50)	1.9 (1.4 to 2.5)	-21 (-41.4 to 4)	879 (641 to 1170)	43.3 (31.6 to 57.6)	-21.8 (-43.4 to 4.6)
	Female	7 (5 to 9)	0.3 (0.2 to 0.4)	-11.5 (-33.5 to 15.9)	5 (3 to 6)	0.2 (0.2 to 0.3)	-19.2 (-38.9 to 4.1)	104 (78 to 139)	4.9 (3.7 to 6.5)	-21.3 (-41.1 to 3.4)
Papua New Guinea	Both	39 (28 to 52)	0.9 (0.6 to 1.2)	-2.1 (-17.6 to 17)	35 (26 to 48)	0.9 (0.6 to 1.1)	-3.3 (-18.3 to 15.5)	993 (712 to 1360)	19.5 (14.2 to 26.2)	-4.5 (-20.6 to 14.7)
	Male	31 (21 to 42)	1.4 (1 to 1.8)	-2.8 (-20.4 to 19.2)	28 (20 to 38)	1.4 (1 to 1.8)	-4.3 (-21.2 to 16.8)	766 (533 to 1070)	29.5 (21.2 to 39.9)	-4.6 (-23 to 18.5)
	Female	8 (6 to 12)	0.3 (0.2 to 0.5)	-3.7 (-26.1 to 26.8)	7 (5 to 11)	0.3 (0.2 to 0.5)	-5 (-26.2 to 24.3)	227 (153 to 326)	8.7 (5.9 to 12.4)	-5.7 (-28.7 to 26.7)
Paraguay	Both	113 (83 to 149)	2 (1.5 to 2.6)	15.2 (-16.3 to 56.5)	84 (63 to 110)	1.5 (1.1 to 2)	5.4 (-22.5 to 41.9)	2230 (1640 to 2960)	38.6 (28.5 to 51)	5.7 (-23.4 to 43.3)

	Male	104 (76 to 138)	3.8 (2.8 to 5)	15.4 (-16.4 to 57.2)	77 (57 to 101)	2.9 (2.2 to 3.8)	5.7 (-22.2 to 42.3)	2070 (1510 to 2760)	73.3 (53.8 to 97.4)	6.1 (-23.5 to 44.3)
	Female	9 (7 to 12)	0.3 (0.2 to 0.4)	14.8 (-16.3 to 54.2)	7 (5 to 9)	0.2 (0.2 to 0.3)	5.4 (-22 to 40.6)	163 (121 to 214)	5.5 (4.1 to 7.2)	5 (-23.6 to 42.1)
Peru	Both	229 (168 to 309)	0.7 (0.5 to 1)	-9.5 (-36.3 to 25.4)	180 (132 to 242)	0.6 (0.4 to 0.8)	-17.1 (-41.4 to 14)	3860 (2810 to 5260)	12.1 (8.8 to 16.4)	-18.8 (-43.8 to 14.2)
	Male	180 (131 to 248)	1.2 (0.9 to 1.6)	-8.2 (-36.5 to 29.6)	145 (105 to 197)	1 (0.7 to 1.3)	-15.7 (-40.7 to 18.3)	3000 (2150 to 4180)	19.5 (14.1 to 27.1)	-17 (-43.2 to 17.9)
	Female	49 (36 to 65)	0.3 (0.2 to 0.4)	-12.4 (-38.8 to 23.8)	36 (26 to 47)	0.2 (0.2 to 0.3)	-20.7 (-43.7 to 10.4)	863 (630 to 1160)	5.1 (3.7 to 6.9)	-22.6 (-47.4 to 10.2)
Philippines	Both	874 (694 to 1110)	1.1 (0.9 to 1.4)	-1.5 (-22.7 to 24.1)	691 (551 to 869)	0.9 (0.7 to 1.1)	-5.1 (-24.1 to 17)	18800 (15000 to 24000)	22.4 (17.8 to 28.3)	-5.1 (-24.7 to 18.3)
	Male	725 (553 to 954)	2 (1.5 to 2.6)	0.7 (-24.9 to 31.1)	562 (424 to 737)	1.6 (1.2 to 2.1)	-2.7 (-27.1 to 25.8)	15500 (11700 to 20500)	39.2 (29.9 to 51.5)	-3.5 (-27.7 to 25.5)
	Female	149 (115 to 190)	0.4 (0.3 to 0.5)	-8.2 (-29.1 to 17.8)	129 (100 to 163)	0.3 (0.3 to 0.4)	-10.7 (-30.1 to 12.5)	3360 (2560 to 4280)	7.7 (5.9 to 9.7)	-10.7 (-31.5 to 15)
Poland	Both	2740 (2200 to 3440)	4.2 (3.3 to 5.3)	-8.6 (-27.1 to 15.5)	1780 (1430 to 2200)	2.6 (2.1 to 3.3)	-14 (-31.5 to 6.4)	45600 (36500 to 56400)	71.1 (56.6 to 88.1)	-15.8 (-33.2 to 4.4)
	Male	2400 (1870 to 3110)	8.1 (6.3 to 10.4)	-11 (-30.5 to 15.2)	1590 (1230 to 2020)	5.4 (4.2 to 6.8)	-15.8 (-34.6 to 6.4)	41000 (31700 to 52100)	138.9 (107.4 to 176.9)	-17.5 (-36.7 to 4.4)
	Female	337 (245 to 435)	1 (0.7 to 1.2)	-3.6 (-25.8 to 24)	189 (142 to 237)	0.5 (0.4 to 0.6)	-11.1 (-30.5 to 11.8)	4660 (3490 to 5880)	13.5 (10.1 to 17.1)	-12.8 (-32.1 to 9.2)
Portugal	Both	557 (423 to 723)	2.8 (2.1 to 3.6)	-18.7 (-39.3 to 6.8)	392 (355 to 429)	1.8 (1.7 to 2)	-22.4 (-30.4 to -13.7)	9600 (8610 to 10600)	50.4 (45.2 to 55.7)	-25.8 (-34.3 to -16.3)
	Male	521 (394 to 678)	5.7 (4.3 to 7.4)	-19.5 (-40 to 6.1)	369 (333 to 405)	3.9 (3.5 to 4.2)	-22.9 (-31.1 to -14)	9180 (8190 to 10100)	104.2 (93.3 to 115.6)	-26.1 (-34.6 to -16.3)
	Female	36 (27 to 50)	0.3 (0.2 to 0.4)	0.8 (-24.5 to 32.9)	23 (19 to 28)	0.2 (0.1 to 0.2)	-8 (-19.7 to 5.2)	426 (360 to 573)	3.8 (3.2 to 5.4)	-7.2 (-19.9 to 7.3)
Puerto Rico	Both	113 (85 to 147)	1.7 (1.3 to 2.2)	-2.5 (-26.5 to 27.9)	64 (49 to 82)	0.9 (0.7 to 1.2)	-7.7 (-29.8 to 19.6)	1400 (1050 to 1820)	22.2 (16.6 to 29.2)	-7.6 (-31.1 to 21.1)
	Male	99 (74 to 130)	3.4 (2.5 to 4.4)	-2.8 (-27.4 to 27.6)	56 (43 to 73)	1.8 (1.4 to 2.4)	-8 (-30.7 to 19.3)	1250 (936 to 1640)	43.7 (32.2 to 58)	-7.9 (-31.6 to 21.4)
	Female	13 (10 to 20)	0.4 (0.3 to 0.5)	-2.6 (-26.1 to 27.2)	8 (6 to 11)	0.2 (0.1 to 0.3)	-7.7 (-29.6 to 19.8)	155 (114 to 225)	4.4 (3.2 to 6.4)	-8 (-31.2 to 21)
Qatar	Both	30 (20 to 43)	4.5 (2.9 to 6.1)	-0.4 (-27.4 to 38.7)	14 (9 to 19)	2.9 (2 to 4)	-3 (-27.8 to 34.5)	414 (273 to 581)	49.6 (32.4 to 67.5)	-14.2 (-37.4 to 19.6)
	Male	29 (18 to 41)	5.7 (3.7 to 7.8)	-7.8 (-33.5 to 29.7)	13 (8 to 18)	3.7 (2.5 to 5.1)	-10.6 (-33.7 to 25.4)	389 (253 to 551)	63.5 (41.2 to 87.2)	-20.4 (-42.3 to 11.6)

	Female	2 (1 to 2)	0.6 (0.4 to 0.8)	-12.2 (-32.8 to 12.9)	1 (0 to 1)	0.3 (0.2 to 0.4)	-23 (-39.8 to -1.4)	26 (18 to 35)	6.9 (5.2 to 9)	-22.8 (-40.3 to -1.3)
South Korea	Both	2230 (1770 to 2800)	2.5 (1.9 to 3.1)	-5.2 (-24.2 to 16.2)	518 (450 to 606)	0.6 (0.5 to 0.7)	-15.3 (-25.8 to -3.7)	11700 (10100 to 13800)	12.9 (11.3 to 15.2)	-16.3 (-26.9 to -3.6)
	Male	2040 (1600 to 2590)	5 (3.9 to 6.2)	-9.5 (-28 to 11.8)	465 (402 to 550)	1.3 (1.1 to 1.5)	-19 (-30.3 to -6.8)	10500 (9090 to 12600)	25.8 (22.3 to 30.7)	-19.9 (-31.1 to -7.4)
	Female	195 (153 to 243)	0.4 (0.3 to 0.5)	-10.2 (-28.9 to 12.7)	53 (43 to 63)	0.1 (0.1 to 0.1)	-23.2 (-35.1 to -9.6)	1120 (943 to 1340)	2.4 (2.1 to 2.9)	-20 (-30.8 to -6.3)
Moldova	Both	202 (171 to 235)	3.5 (2.9 to 4)	-17 (-31 to -1.9)	140 (119 to 163)	2.4 (2 to 2.8)	-23.9 (-36.2 to -10.7)	4170 (3530 to 4870)	73.3 (62 to 85.5)	-25.5 (-37.9 to -12)
	Male	194 (164 to 227)	7.7 (6.5 to 9)	-16.4 (-30.6 to -1.3)	135 (114 to 158)	5.4 (4.6 to 6.3)	-23.2 (-35.8 to -10.1)	4040 (3400 to 4720)	160.5 (135.6 to 187.2)	-25.1 (-37.7 to -11.8)
	Female	7 (6 to 9)	0.2 (0.2 to 0.3)	-27 (-41.5 to -4.4)	5 (4 to 6)	0.1 (0.1 to 0.2)	-33.6 (-46.3 to -14.2)	134 (110 to 170)	4.4 (3.6 to 5.5)	-35.2 (-47.8 to -14.7)
Romania	Both	1900 (1510 to 2320)	5.9 (4.6 to 7.2)	1.7 (-20.1 to 26.4)	1030 (834 to 1250)	3.1 (2.4 to 3.7)	-7.8 (-26.9 to 12.3)	28600 (22600 to 34900)	89.8 (70.5 to 110.4)	-9.4 (-29 to 11.9)
	Male	1800 (1420 to 2210)	11.9 (9.3 to 14.7)	0.4 (-21.1 to 24.9)	972 (782 to 1180)	6.3 (5 to 7.7)	-8.4 (-27.1 to 11.6)	27100 (21400 to 33200)	180.3 (141.4 to 221)	-10.5 (-29.8 to 10.6)
	Female	99 (77 to 124)	0.6 (0.4 to 0.7)	3.5 (-18.8 to 32.3)	61 (48 to 74)	0.3 (0.2 to 0.4)	-7 (-26.5 to 16.6)	1510 (1180 to 1890)	9 (6.9 to 11.3)	-6.4 (-27.2 to 18.7)
Russia	Both	6930 (5720 to 8310)	3 (2.5 to 3.6)	-14.1 (-28.9 to 3.3)	4110 (3400 to 4920)	1.8 (1.5 to 2.1)	-23.8 (-37.2 to -9.1)	114000 (93700 to 136000)	50.1 (41.2 to 59.8)	-24.6 (-38 to -9.9)
	Male	6500 (5290 to 7870)	6.8 (5.6 to 8.3)	-17 (-32.4 to 0.7)	3850 (3150 to 4660)	4.2 (3.4 to 5)	-26.2 (-39.4 to -11.1)	107000 (87700 to 129000)	112.4 (92.1 to 135.6)	-26.9 (-40.3 to -11.5)
	Female	434 (349 to 532)	0.3 (0.3 to 0.4)	0.5 (-19.3 to 25.5)	264 (216 to 321)	0.2 (0.2 to 0.2)	-12.3 (-28.3 to 8.7)	6800 (5540 to 8350)	5.4 (4.3 to 6.6)	-11.6 (-28.6 to 10.5)
Rwanda	Both	89 (68 to 120)	1.4 (1.1 to 1.9)	-1.8 (-19.3 to 20.2)	81 (62 to 110)	1.3 (1 to 1.8)	-4.6 (-20.8 to 15.8)	2390 (1780 to 3230)	34.6 (26.4 to 46.3)	-5.2 (-23.5 to 18.2)
	Male	69 (51 to 99)	2.6 (1.9 to 3.6)	-2.3 (-20.9 to 20.8)	63 (47 to 90)	2.5 (1.9 to 3.4)	-4.9 (-22.3 to 16.3)	1860 (1350 to 2650)	63 (46.7 to 89)	-5.7 (-25.2 to 18.5)
	Female	20 (15 to 26)	0.5 (0.4 to 0.7)	-4.2 (-25.5 to 23.2)	18 (14 to 23)	0.5 (0.4 to 0.7)	-6.9 (-27 to 19)	527 (389 to 704)	13.4 (10.2 to 17.7)	-7.8 (-29.1 to 20.3)
Saint Kitts and Nevis	Both	2 (1 to 2)	2.5 (2.1 to 3)	30.6 (10.9 to 53.7)	1 (1 to 1)	1.7 (1.4 to 2)	21.3 (4.4 to 41.1)	31 (25 to 37)	41.9 (34.1 to 50.1)	25 (4.4 to 48.1)
	Male	2 (1 to 2)	5.1 (4.3 to 6.1)	26.2 (7.9 to 48.5)	1 (1 to 1)	3.5 (3 to 4.1)	15.6 (-0.1 to 34.5)	30 (24 to 37)	84.1 (68.9 to 100.2)	21.9 (2.1 to 44.3)

	Female	0 (0 to 0)	0.1 (0.1 to 0.1)	-4.5 (-22.2 to 18.1)	0 (0 to 0)	0.1 (0.1 to 0.1)	-6.9 (-22.8 to 11.9)	1 (0 to 1)	1.6 (1.2 to 2)	-8.5 (-26.6 to 12)
Saint Lucia	Both	6 (5 to 7)	2.8 (2.3 to 3.3)	13.4 (-6.9 to 35.2)	5 (4 to 5)	2.1 (1.8 to 2.5)	11.5 (-8 to 32.2)	116 (96 to 139)	52.3 (43.5 to 62.5)	9.1 (-10.5 to 30.8)
	Male	6 (5 to 7)	5.4 (4.5 to 6.4)	11.6 (-8.6 to 34.2)	4 (4 to 5)	4.2 (3.5 to 5)	9.4 (-9.8 to 30.6)	107 (89 to 129)	100.6 (83.3 to 120.5)	7.7 (-12.2 to 29.4)
	Female	0 (0 to 1)	0.4 (0.3 to 0.5)	20.8 (-2.2 to 45.9)	0 (0 to 0)	0.3 (0.3 to 0.4)	19 (-1.9 to 42.9)	9 (7 to 10)	7.5 (6.1 to 9.1)	15 (-6.9 to 40.3)
Saint Vincent and the Grenadines	Both	5 (4 to 6)	3.5 (3 to 4)	17.7 (-0.5 to 38.8)	4 (3 to 5)	2.9 (2.5 to 3.3)	17.1 (-0.3 to 36.9)	99 (85 to 117)	71.2 (61.3 to 83.2)	15.2 (-3.6 to 37.3)
	Male	5 (4 to 5)	6.4 (5.6 to 7.5)	13.7 (-3.8 to 34.1)	4 (3 to 4)	5.4 (4.7 to 6.2)	12.2 (-4.6 to 31.1)	95 (81 to 111)	131.4 (113.1 to 153.6)	12.5 (-6.1 to 34.7)
	Female	0 (0 to 0)	0.3 (0.3 to 0.4)	9 (-7.9 to 28.9)	0 (0 to 0)	0.3 (0.2 to 0.3)	7.7 (-8.5 to 26.8)	4 (4 to 5)	6.8 (5.7 to 8.1)	7.7 (-9.3 to 28.4)
Samoa	Both	1 (1 to 2)	0.9 (0.8 to 1.1)	-2.7 (-18 to 16.7)	1 (1 to 1)	0.8 (0.6 to 0.9)	-5.2 (-19.3 to 11.9)	26 (21 to 32)	17.6 (14.3 to 21.4)	-5.5 (-20.8 to 14.1)
	Male	1 (1 to 1)	1.4 (1.2 to 1.8)	-3.8 (-19.9 to 16.4)	1 (1 to 1)	1.2 (1 to 1.5)	-6.3 (-21.6 to 12.1)	18 (15 to 23)	25.9 (20.9 to 31.8)	-6.5 (-22 to 13.9)
	Female	0 (0 to 0)	0.5 (0.3 to 0.6)	-2.5 (-26.6 to 29.3)	0 (0 to 0)	0.4 (0.3 to 0.5)	-4.6 (-26.6 to 26.2)	8 (6 to 11)	10.1 (7.4 to 13.6)	-5.3 (-28.8 to 26.3)
San Marino	Both	2 (2 to 3)	4 (2.8 to 5.4)	5.9 (-19 to 35)	1 (1 to 1)	1.3 (0.8 to 1.9)	0.3 (-29.4 to 36.4)	19 (12 to 28)	34.2 (21.2 to 50.8)	0.7 (-30.3 to 40.3)
	Male	2 (2 to 3)	8 (5.7 to 10.8)	6.6 (-18.8 to 35.7)	1 (1 to 1)	2.7 (1.8 to 4)	-0.1 (-29.4 to 35.5)	18 (12 to 27)	68.4 (42.8 to 101.3)	2 (-29.1 to 41.6)
	Female	0 (0 to 0)	0.2 (0.1 to 0.3)	0.1 (-27.4 to 33.8)	0 (0 to 0)	0.1 (0.1 to 0.1)	-6 (-37.7 to 38.7)	1 (0 to 1)	2.3 (1.4 to 3.6)	-4.2 (-36.3 to 41.3)
São Tome and Príncipe	Both	1 (1 to 2)	1.2 (1 to 1.5)	9.7 (-10.7 to 33.3)	1 (1 to 1)	1.2 (1 to 1.4)	6.1 (-13.1 to 28.7)	28 (22 to 36)	26.1 (20.6 to 32.7)	5.5 (-15.5 to 30.1)
	Male	1 (1 to 1)	2.4 (1.9 to 3)	10.3 (-11.3 to 35.3)	1 (1 to 1)	2.4 (1.9 to 2.9)	6.9 (-13.1 to 29.8)	26 (20 to 33)	50.2 (39.7 to 62.9)	5.7 (-15.9 to 30.5)
	Female	0 (0 to 0)	0.2 (0.1 to 0.2)	0.1 (-20.8 to 26)	0 (0 to 0)	0.2 (0.1 to 0.2)	-3.3 (-23 to 21.2)	2 (2 to 3)	3.9 (3 to 5.3)	-4.5 (-24.9 to 21.3)
Saudi Arabia	Both	222 (167 to 297)	1.1 (0.8 to 1.4)	-3 (-21.6 to 19.5)	115 (88 to 153)	0.6 (0.5 to 0.8)	-19.4 (-34 to -2.3)	3770 (2800 to 5090)	16 (12.6 to 21.1)	-18.5 (-35 to 0.9)
	Male	192 (142 to 256)	1.6 (1.2 to 2.1)	-4.3 (-23.3 to 18.6)	101 (77 to 135)	1 (0.8 to 1.3)	-20.7 (-35.4 to -2.5)	3250 (2380 to 4430)	23.3 (18.4 to 30.9)	-18.9 (-35.9 to 1)
	Female	30 (21 to 42)	0.3 (0.2 to 0.4)	-11.9 (-34.3 to 15.2)	14 (10 to 19)	0.2 (0.1 to 0.2)	-28.5 (-44.8 to -7)	518 (367 to 708)	4.7 (3.5 to 6.4)	-27.3 (-44.8 to -3.7)
Senegal	Both	91 (69 to 118)	1.2 (0.9 to 1.6)	4 (-19.4 to 32.1)	87 (67 to 112)	1.2 (0.9 to 1.6)	2 (-19.7 to 28)	2240 (1650 to 2950)	28.5 (21.5 to 37.1)	2.3 (-22.5 to 32.4)
	Male	81 (60 to 107)	2.3 (1.7 to 3)	5 (-18.8 to 34.5)	78 (58 to 101)	2.3 (1.8 to 3)	3 (-19.5 to 30.3)	2000 (1450 to 2660)	53.1 (39.3 to 69.9)	3.3 (-21.8 to 34.9)
	Female	9 (7 to 12)	0.2 (0.2 to 0.3)	4.5 (-22.2 to 37.7)	9 (7 to 11)	0.2 (0.2 to 0.3)	2.7 (-22.5 to 33.5)	233 (169 to 306)	5.6 (4.1 to 7.4)	2 (-24.9 to 33.6)

Serbia	Both	984 (747 to 1260)	6.5 (5 to 8.4)	-5.1 (-25.9 to 22.4)	459 (352 to 581)	2.9 (2.3 to 3.7)	-14.5 (-32.9 to 9.2)	11700 (8930 to 15000)	79.1 (60.4 to 101.8)	-14.9 (-34.2 to 9.8)
	Male	873 (657 to 1130)	12.3 (9.3 to 16)	-6.2 (-27.4 to 22.2)	415 (318 to 531)	5.7 (4.4 to 7.3)	-15.1 (-33.6 to 8.9)	10600 (8020 to 13700)	151.6 (114.6 to 195.5)	-15.7 (-35 to 9.5)
	Female	111 (83 to 145)	1.4 (1 to 1.8)	-2.5 (-27.9 to 29.1)	44 (34 to 56)	0.5 (0.4 to 0.7)	-14.3 (-35 to 10.6)	1070 (803 to 1380)	13.8 (10.3 to 17.8)	-13.8 (-36.1 to 13.7)
Seychelles	Both	9 (7 to 11)	7.6 (6.3 to 9.1)	8.5 (-10.6 to 30.8)	6 (5 to 7)	5.1 (4.3 to 6.1)	0.8 (-15.5 to 19.5)	151 (125 to 180)	127.5 (106 to 152.1)	0.9 (-17.5 to 21.9)
	Male	8 (7 to 10)	15.1 (12.5 to 18.2)	3.6 (-15.7 to 25.8)	5 (4 to 6)	10.4 (8.8 to 12.4)	-4.3 (-20.5 to 14.7)	142 (116 to 172)	245.1 (203 to 295)	-3 (-20.5 to 18.1)
	Female	1 (0 to 1)	0.9 (0.7 to 1.1)	8.5 (-13.2 to 34.7)	0 (0 to 0)	0.7 (0.6 to 0.8)	2.3 (-18.1 to 26.9)	9 (7 to 11)	15.8 (12.7 to 19.3)	2 (-18.4 to 27.3)
Sierra Leone	Both	47 (35 to 63)	1.3 (1 to 1.8)	-1.9 (-22.1 to 24.5)	45 (34 to 60)	1.3 (1 to 1.8)	-4.4 (-23.6 to 20.7)	1200 (871 to 1630)	31.7 (23.5 to 42.9)	-4.1 (-24.7 to 22.1)
	Male	42 (31 to 57)	2.4 (1.8 to 3.2)	-3.4 (-23.9 to 23.9)	40 (30 to 54)	2.4 (1.8 to 3.2)	-5.6 (-25.1 to 19.6)	1060 (759 to 1460)	56 (40.8 to 76.6)	-5.6 (-26.7 to 21.3)
	Female	5 (4 to 7)	0.3 (0.2 to 0.4)	-5.7 (-26.4 to 21.5)	5 (4 to 7)	0.3 (0.2 to 0.4)	-7.6 (-27.8 to 17.6)	133 (94 to 185)	6.9 (4.9 to 9.5)	-8.8 (-30 to 19.7)
Singapore	Both	148 (114 to 190)	1.9 (1.4 to 2.4)	-22.3 (-39.3 to -1.5)	35 (30 to 40)	0.5 (0.4 to 0.5)	-27.8 (-36.2 to -17.3)	797 (693 to 916)	10.1 (8.8 to 11.6)	-29.2 (-37.2 to -18.4)
	Male	133 (101 to 171)	3.5 (2.7 to 4.5)	-26.6 (-43.3 to -6.5)	31 (27 to 35)	0.9 (0.8 to 1.1)	-31.8 (-40.5 to -21)	706 (608 to 820)	18.6 (16 to 21.6)	-33 (-41 to -22.1)
	Female	16 (12 to 21)	0.4 (0.3 to 0.5)	-3.8 (-26.4 to 24.7)	4 (3 to 5)	0.1 (0.1 to 0.1)	-13.2 (-25.8 to 1.6)	91 (76 to 112)	2.3 (1.9 to 2.8)	-13.8 (-25.2 to 0)
Slovakia	Both	314 (236 to 417)	3.5 (2.6 to 4.6)	-8.9 (-32.1 to 22.5)	177 (134 to 233)	1.9 (1.5 to 2.5)	-16.1 (-36.4 to 10.5)	4840 (3610 to 6380)	55.1 (40.9 to 72.9)	-16.9 (-38 to 10.7)
	Male	295 (220 to 396)	7.2 (5.4 to 9.5)	-11.9 (-34.3 to 18.5)	167 (125 to 221)	4.1 (3.1 to 5.4)	-18.6 (-38.4 to 7.5)	4590 (3400 to 6080)	112.4 (83.5 to 148.6)	-19.3 (-39.6 to 7.6)
	Female	19 (14 to 25)	0.4 (0.3 to 0.5)	-2.5 (-29.6 to 32.1)	10 (7 to 13)	0.2 (0.1 to 0.3)	-11.6 (-34.3 to 14.8)	250 (183 to 332)	5.4 (4 to 7.3)	-12.2 (-35.8 to 17.1)
Slovenia	Both	128 (98 to 170)	3.3 (2.5 to 4.4)	-7 (-29.4 to 25.2)	54 (41 to 70)	1.3 (1 to 1.7)	-16.4 (-35.6 to 11.5)	1300 (989 to 1750)	34.6 (26.2 to 46.3)	-18 (-38.2 to 10.5)
	Male	116 (88 to 155)	6.3 (4.8 to 8.4)	-10.4 (-31.9 to 19.9)	48 (37 to 63)	2.6 (2 to 3.4)	-19.8 (-38.1 to 6.9)	1170 (885 to 1570)	65 (49.1 to 86.7)	-20.7 (-39.9 to 7.4)
	Female	13 (9 to 17)	0.6 (0.4 to 0.9)	-1 (-27.8 to 36.4)	6 (4 to 8)	0.3 (0.2 to 0.3)	-11.5 (-34.2 to 17.5)	131 (91 to 179)	6.7 (4.7 to 9.2)	-11.2 (-34.8 to 20.8)
Solomon Islands	Both	5 (3 to 6)	1.6 (1.1 to 2)	2 (-16.3 to 23)	4 (3 to 5)	1.4 (1 to 1.8)	-2.3 (-18.9 to 17.4)	127 (78 to 171)	35.5 (23.3 to 46.5)	-2.4 (-20.4 to 19.7)
	Male	4 (2 to 5)	2.4 (1.5 to 3.1)	2.9 (-17.2 to 27.1)	3 (2 to 4)	2.2 (1.4 to 2.8)	-1.2 (-19.5 to 20.4)	94 (49 to 130)	52.6 (30.5 to 69.7)	-1.9 (-21.2 to 22.9)

	Female	1 (1 to 2)	0.7 (0.5 to 0.9)	6.9 (-17.6 to 42.2)	1 (1 to 1)	0.6 (0.4 to 0.8)	3 (-19.5 to 35.4)	33 (20 to 46)	17.8 (11.6 to 23.8)	1.8 (-22.3 to 36.2)
Somalia	Both	100 (69 to 141)	1.4 (0.9 to 1.9)	-7.7 (-25.1 to 14.8)	97 (68 to 136)	1.4 (1 to 1.9)	-8.2 (-25 to 13.4)	3140 (2160 to 4470)	38.4 (26.8 to 53.9)	-8.4 (-26.5 to 16.1)
	Male	75 (49 to 106)	2.4 (1.6 to 3.3)	-8.5 (-26.9 to 15.8)	73 (48 to 102)	2.4 (1.6 to 3.3)	-8.9 (-26.6 to 13.5)	2360 (1510 to 3360)	66.1 (43.2 to 92.2)	-9.3 (-28.1 to 16.2)
	Female	25 (16 to 37)	0.6 (0.4 to 0.9)	-4.5 (-26.8 to 25.7)	24 (16 to 36)	0.6 (0.4 to 0.9)	-4.9 (-26.4 to 24)	782 (503 to 1170)	17.1 (11.3 to 25.7)	-5.9 (-28.1 to 25.1)
South Africa	Both	738 (663 to 824)	1.6 (1.4 to 1.8)	-23.4 (-30.4 to -14.2)	616 (560 to 680)	1.4 (1.2 to 1.5)	-26.8 (-32.9 to -18.9)	17100 (15400 to 19000)	35.5 (32 to 39.3)	-29.3 (-35.6 to -20.7)
	Male	629 (561 to 716)	3.2 (2.9 to 3.6)	-23.9 (-31.2 to -14.3)	524 (472 to 589)	2.8 (2.6 to 3.1)	-27.2 (-33.5 to -18.2)	14600 (13000 to 16500)	70.4 (63.2 to 79.4)	-29.7 (-36.5 to -20.4)
	Female	109 (97 to 124)	0.4 (0.4 to 0.5)	-22.8 (-31.7 to -13.1)	92 (82 to 104)	0.4 (0.3 to 0.4)	-25.7 (-33.9 to -16.6)	2450 (2170 to 2790)	9.1 (8 to 10.3)	-29.2 (-38.5 to -18.9)
South Sudan	Both	39 (26 to 57)	1 (0.7 to 1.4)	-9 (-26.5 to 13.9)	37 (25 to 54)	1 (0.7 to 1.4)	-9.7 (-27.1 to 13.8)	1100 (726 to 1630)	24.7 (16.8 to 36.1)	-10.2 (-29 to 14.8)
	Male	31 (20 to 48)	1.5 (1 to 2.3)	-7.2 (-25.7 to 17.5)	30 (20 to 46)	1.5 (1 to 2.3)	-7.9 (-26.5 to 16.9)	862 (557 to 1350)	37.4 (24.6 to 58.5)	-8.2 (-28.1 to 18.8)
	Female	8 (5 to 11)	0.4 (0.3 to 0.6)	-5.5 (-26.5 to 23.1)	7 (5 to 11)	0.4 (0.3 to 0.5)	-5.9 (-27.7 to 23.7)	234 (151 to 342)	10.4 (7 to 15)	-6.3 (-28.6 to 25.5)
Spain	Both	3740 (2810 to 4850)	4.5 (3.4 to 5.8)	-16.5 (-37 to 11.5)	1500 (1370 to 1650)	1.6 (1.5 to 1.8)	-19.1 (-26.8 to -10.4)	34400 (31100 to 38100)	41.8 (37.7 to 46.3)	-21.7 (-29.8 to -12.2)
	Male	3550 (2680 to 4600)	9 (6.7 to 11.7)	-17.5 (-37.5 to 10.1)	1410 (1280 to 1560)	3.4 (3.1 to 3.8)	-20.4 (-28 to -11.6)	32300 (29100 to 35900)	82.9 (74.3 to 92.3)	-22.7 (-31.3 to -13)
	Female	190 (135 to 252)	0.4 (0.3 to 0.6)	-1.1 (-26.7 to 32.6)	90 (68 to 105)	0.2 (0.1 to 0.2)	-6.2 (-17.7 to 7.5)	2070 (1540 to 2400)	5 (3.7 to 5.8)	-6.7 (-19.5 to 7.9)
Sri Lanka	Both	318 (228 to 433)	1.2 (0.9 to 1.6)	-8.1 (-33.5 to 22.6)	180 (130 to 242)	0.7 (0.5 to 1)	-20.8 (-41.6 to 4.3)	4530 (3190 to 6190)	17.1 (12.2 to 23.2)	-21 (-42.9 to 5.8)
	Male	261 (183 to 362)	2.2 (1.6 to 3.1)	-9 (-34.9 to 22.4)	143 (100 to 195)	1.3 (0.9 to 1.7)	-21.7 (-42.8 to 2.9)	3660 (2530 to 5090)	30.5 (21.3 to 42)	-22.4 (-44.8 to 4.5)
	Female	57 (41 to 78)	0.4 (0.3 to 0.5)	1.7 (-27.1 to 36.2)	38 (27 to 51)	0.3 (0.2 to 0.4)	-10.6 (-35.7 to 19.1)	871 (620 to 1180)	6.1 (4.4 to 8.3)	-10.6 (-36.6 to 20.1)
Sudan	Both	410 (270 to 553)	2.2 (1.5 to 2.9)	1.2 (-16.8 to 24.7)	345 (240 to 462)	1.9 (1.4 to 2.5)	-4 (-21.1 to 17.5)	9570 (6340 to 13300)	46.5 (31.9 to 63.1)	-6.4 (-23.9 to 16.3)
	Male	327 (211 to 446)	3.2 (2.1 to 4.4)	0.6 (-18 to 26.4)	279 (189 to 377)	2.9 (2 to 3.9)	-4.6 (-22 to 19.1)	7540 (4880 to 10500)	69.5 (46.4 to 95.6)	-6.7 (-24.6 to 18.2)
	Female	83 (54 to 118)	0.9 (0.6 to 1.2)	4.7 (-17.8 to 33.6)	65 (45 to 92)	0.7 (0.5 to 1)	-1.6 (-21.8 to 25.6)	2040 (1320 to 3010)	19.3 (12.9 to 27.4)	-3.8 (-25.4 to 25.7)

Suriname	Both	6 (5 to 8)	1 (0.8 to 1.3)	5.9 (-15.9 to 32.5)	5 (4 to 6)	0.9 (0.7 to 1.1)	2.3 (-18.7 to 26.8)	138 (110 to 171)	21.8 (17.4 to 26.9)	2.4 (-19 to 28.4)
	Male	5 (4 to 7)	1.9 (1.5 to 2.3)	6.1 (-17 to 34.2)	4 (4 to 6)	1.6 (1.3 to 2)	2.7 (-19 to 27.9)	119 (93 to 150)	39.8 (31.4 to 49.7)	2.3 (-20.2 to 29.6)
	Female	1 (1 to 1)	0.3 (0.2 to 0.4)	10.8 (-11.2 to 41.5)	1 (1 to 1)	0.2 (0.2 to 0.3)	7 (-14 to 36.2)	19 (15 to 24)	5.8 (4.7 to 7.3)	7.5 (-13.8 to 38.5)
Sweden	Both	232 (187 to 286)	1.2 (1 to 1.5)	-4.1 (-22.8 to 19.4)	72 (66 to 80)	0.3 (0.3 to 0.4)	-7.4 (-14.8 to 1.7)	1500 (1370 to 1650)	8.1 (7.4 to 8.8)	-9.7 (-17.6 to -0.7)
	Male	204 (160 to 255)	2.2 (1.7 to 2.7)	-6 (-26.4 to 19.8)	58 (52 to 65)	0.6 (0.5 to 0.6)	-9.7 (-18.7 to 0.5)	1210 (1090 to 1340)	13.2 (11.8 to 14.6)	-11.4 (-20.8 to -0.4)
	Female	28 (22 to 35)	0.3 (0.2 to 0.4)	-1.7 (-22.7 to 25)	14 (12 to 16)	0.1 (0.1 to 0.1)	-6.4 (-16.2 to 5.1)	296 (261 to 336)	3.3 (2.9 to 3.8)	-7.4 (-17.3 to 4.5)
Switzerland	Both	289 (216 to 381)	1.8 (1.4 to 2.4)	-16.5 (-38.1 to 10.9)	103 (92 to 114)	0.6 (0.5 to 0.7)	-17.3 (-25.8 to -8)	2300 (2050 to 2590)	14.9 (13.3 to 16.8)	-19.3 (-28.7 to -8.9)
	Male	253 (188 to 335)	3.4 (2.5 to 4.4)	-18.7 (-40 to 7.2)	85 (75 to 96)	1.1 (1 to 1.2)	-20.3 (-29.6 to -10)	1930 (1690 to 2180)	25.9 (22.7 to 29.2)	-21.6 (-32.2 to -11.2)
	Female	36 (27 to 48)	0.4 (0.3 to 0.6)	-10.6 (-33.7 to 19.1)	18 (15 to 21)	0.2 (0.2 to 0.2)	-11.8 (-23.8 to 2.7)	373 (317 to 444)	4.8 (4.1 to 5.7)	-13 (-25.5 to 2)
Syria	Both	148 (110 to 196)	1.2 (0.9 to 1.6)	-2.8 (-26.7 to 28)	101 (75 to 133)	0.9 (0.7 to 1.1)	-7.3 (-29.3 to 19.4)	2700 (1980 to 3590)	20.3 (15.1 to 26.9)	-5.5 (-29.6 to 24.8)
	Male	125 (92 to 168)	1.9 (1.4 to 2.6)	-0.5 (-26.3 to 33)	86 (63 to 115)	1.4 (1.1 to 1.9)	-5 (-29.4 to 25.3)	2280 (1670 to 3060)	33.6 (24.8 to 45)	-3.3 (-29 to 29.7)
	Female	23 (17 to 31)	0.4 (0.3 to 0.5)	-1.3 (-26 to 31)	15 (11 to 20)	0.3 (0.2 to 0.4)	-6.8 (-27.7 to 20)	419 (310 to 569)	6.5 (4.9 to 8.6)	-3.7 (-28.1 to 29.3)
Taiwan (Province of China)	Both	798 (609 to 1050)	2 (1.5 to 2.7)	1.8 (-23 to 34.5)	276 (213 to 360)	0.7 (0.5 to 0.9)	-6.6 (-27.8 to 21.6)	6800 (5160 to 9020)	17.5 (13.3 to 23.1)	-5.6 (-28 to 26.2)
	Male	757 (576 to 1010)	4 (3.1 to 5.4)	5 (-20.6 to 38.8)	258 (198 to 337)	1.4 (1.1 to 1.8)	-2.2 (-24.9 to 27)	6390 (4800 to 8520)	34.5 (26.2 to 45.8)	-2.9 (-26.2 to 29.6)
	Female	41 (30 to 56)	0.2 (0.1 to 0.3)	-0.7 (-25.2 to 31.7)	18 (14 to 24)	0.1 (0.1 to 0.1)	-6.5 (-28.2 to 20.8)	409 (309 to 554)	2.1 (1.6 to 2.8)	-6.7 (-29.5 to 21.4)
Tajikistan	Both	57 (44 to 79)	1.1 (0.8 to 1.4)	-26 (-43.3 to -4.5)	48 (37 to 66)	1 (0.8 to 1.3)	-26.3 (-43 to -5.9)	1490 (1140 to 2080)	24.8 (19.2 to 33.5)	-28.9 (-46 to -7)
	Male	37 (27 to 54)	1.5 (1.2 to 2.2)	-27.2 (-45.1 to -3.6)	32 (24 to 47)	1.5 (1.1 to 2.1)	-26.6 (-44.3 to -3.3)	938 (695 to 1370)	34.4 (26 to 51)	-31 (-48.4 to -7.7)
	Female	20 (15 to 27)	0.7 (0.5 to 0.9)	-18.2 (-38.4 to 6.5)	16 (12 to 21)	0.6 (0.4 to 0.7)	-20 (-39 to 4)	550 (409 to 725)	16.1 (12.4 to 20.9)	-20.2 (-41.6 to 5.3)
Thailand	Both	2070 (1520 to 2820)	2 (1.5 to 2.7)	2.2 (-27.9 to 41)	1190 (870 to 1600)	1.2 (0.9 to 1.6)	-9 (-34.7 to 24.1)	30200 (21700 to 41400)	29.1 (21 to 39.6)	-8.4 (-35.9 to 26.9)
	Male	1910 (1380 to 2620)	4 (2.9 to 5.5)	4 (-27.2 to 43.3)	1080 (789 to 1470)	2.3 (1.7 to 3.2)	-7 (-33.8 to 27)	27700 (19800 to 38100)	57.3 (41.1 to 78.3)	-6.7 (-35.2 to 28.9)

	Female	161 (118 to 214)	0.3 (0.2 to 0.4)	-16.1 (-41.1 to 15.9)	110 (82 to 144)	0.2 (0.1 to 0.3)	-24.1 (-45.2 to 2.8)	2480 (1810 to 3320)	4.6 (3.3 to 6.1)	-23.6 (-46.7 to 5.7)
Timor-Leste	Both	9 (6 to 13)	1.1 (0.8 to 1.5)	23.3 (-1.3 to 51.8)	8 (6 to 11)	1.1 (0.8 to 1.4)	17.7 (-5.3 to 44)	204 (137 to 281)	24.3 (16.5 to 32.9)	20.5 (-4.7 to 51.1)
	Male	7 (5 to 11)	1.8 (1.2 to 2.6)	28.9 (0.5 to 63.5)	7 (4 to 9)	1.7 (1.1 to 2.4)	23 (-3.4 to 54.4)	161 (99 to 230)	38.3 (24.2 to 54.7)	25.2 (-2.6 to 62)
	Female	2 (1 to 2)	0.5 (0.3 to 0.6)	9.6 (-12.6 to 36)	2 (1 to 2)	0.4 (0.3 to 0.6)	6 (-15.1 to 30)	44 (30 to 57)	10.3 (7.3 to 13.3)	7.8 (-14.7 to 37.7)
Togo	Both	53 (38 to 71)	1.4 (1 to 1.9)	-5.5 (-22.9 to 20.1)	48 (35 to 64)	1.3 (1 to 1.8)	-7.6 (-24.1 to 16)	1380 (994 to 1850)	33.7 (24.5 to 45.2)	-7.7 (-26.1 to 19)
	Male	48 (34 to 65)	3 (2.3 to 4)	-4.9 (-22.6 to 20.6)	43 (31 to 59)	3 (2.2 to 3.9)	-6.3 (-22.7 to 17.4)	1250 (879 to 1690)	70.5 (51.1 to 94.9)	-7.9 (-26.1 to 19.2)
	Female	5 (4 to 7)	0.2 (0.2 to 0.3)	-3.3 (-25 to 25)	5 (3 to 6)	0.2 (0.2 to 0.3)	-5.1 (-26 to 20.9)	130 (94 to 177)	5.7 (4.2 to 7.7)	-6.8 (-28.4 to 24.2)
Tokelau	Both	0 (0 to 0)	0.8 (0.6 to 1)	-3.1 (-19.4 to 15.9)	0 (0 to 0)	0.6 (0.5 to 0.8)	-8 (-22.6 to 9.6)	0 (0 to 0)	14.3 (11.1 to 18.2)	-10.4 (-26.2 to 6.7)
	Male	0 (0 to 0)	1.1 (0.9 to 1.4)	-6.3 (-23.6 to 15.8)	0 (0 to 0)	0.9 (0.7 to 1.2)	-11.9 (-27.4 to 8.3)	0 (0 to 0)	19.2 (14.8 to 24.3)	-12.2 (-30 to 8.8)
	Female	0 (0 to 0)	0.5 (0.3 to 0.6)	-5.8 (-28.8 to 22.9)	0 (0 to 0)	0.4 (0.3 to 0.5)	-11.1 (-31.8 to 14.6)	0 (0 to 0)	9.7 (6.9 to 13.1)	-11.8 (-33.9 to 16.5)
Tonga	Both	1 (0 to 1)	0.8 (0.6 to 1)	-3.4 (-19.5 to 16.2)	1 (0 to 1)	0.7 (0.5 to 0.8)	-6.6 (-21.3 to 11.8)	11 (9 to 14)	14.5 (11.2 to 18.2)	-6.8 (-22.9 to 12.6)
	Male	0 (0 to 1)	1.4 (1.1 to 1.8)	-3.4 (-19.4 to 17.3)	0 (0 to 1)	1.2 (0.9 to 1.6)	-6.6 (-21.8 to 13)	9 (7 to 12)	25 (19.1 to 32.1)	-6.8 (-23 to 13.2)
	Female	0 (0 to 0)	0.3 (0.2 to 0.3)	0.5 (-23.7 to 30.9)	0 (0 to 0)	0.2 (0.2 to 0.3)	-2.4 (-24.9 to 26.3)	2 (2 to 3)	5.6 (4.1 to 7.4)	-2.9 (-27.1 to 27.5)
Trinidad and Tobago	Both	23 (17 to 30)	1.2 (0.9 to 1.6)	-6.4 (-32 to 25.2)	18 (13 to 23)	1 (0.7 to 1.3)	-9 (-33 to 19.9)	436 (321 to 576)	23.5 (17.4 to 30.9)	-9.1 (-34.1 to 21.7)
	Male	21 (15 to 28)	2.4 (1.7 to 3.1)	-7.2 (-32.7 to 24)	16 (12 to 21)	1.9 (1.4 to 2.5)	-9.6 (-33.8 to 18.9)	408 (299 to 540)	45.2 (33.3 to 59.6)	-9.8 (-34.9 to 20.8)
	Female	2 (1 to 2)	0.2 (0.1 to 0.2)	1.9 (-23.8 to 37.1)	1 (1 to 2)	0.1 (0.1 to 0.2)	-1 (-25.7 to 31.5)	28 (21 to 38)	2.9 (2.2 to 4)	-1.2 (-27.3 to 32.4)
Tunisia	Both	509 (361 to 710)	3.9 (2.8 to 5.5)	6.2 (-19.9 to 45.2)	303 (216 to 420)	2.4 (1.7 to 3.3)	-7.5 (-29.9 to 24.3)	7810 (5500 to 10900)	59.3 (41.9 to 82.4)	-6.7 (-29.9 to 29.1)
	Male	474 (332 to 666)	7.5 (5.3 to 10.6)	7 (-20.1 to 46.3)	284 (201 to 396)	4.7 (3.3 to 6.6)	-6.4 (-29.2 to 26)	7290 (5100 to 10200)	113.2 (79.5 to 158)	-6 (-30.5 to 30.4)
	Female	35 (25 to 47)	0.5 (0.4 to 0.7)	4.4 (-23.5 to 42.4)	19 (14 to 26)	0.3 (0.2 to 0.4)	-9.6 (-33.2 to 21.1)	520 (371 to 703)	7.8 (5.6 to 10.5)	-8.9 (-33.5 to 24.8)
Turkey	Both	2380 (1840 to 3010)	2.7 (2.1 to 3.4)	-11.2 (-31.7 to 15.7)	1440 (1130 to 1800)	1.6 (1.3 to 2.1)	-21.2 (-38.3 to 1.3)	35500 (27400 to 44900)	39.2 (30.3 to 49.3)	-22.8 (-40.5 to 1)

	Male	2110 (1620 to 2680)	5 (3.9 to 6.4)	-12.1 (-33.3 to 15.3)	1270 (985 to 1610)	3.1 (2.5 to 4)	-21.8 (-39.8 to 1.2)	31500 (24000 to 40200)	73.1 (56.1 to 92.1)	-23.4 (-41.5 to 1.1)
	Female	279 (215 to 361)	0.6 (0.5 to 0.8)	-5.1 (-27.8 to 27)	167 (131 to 215)	0.4 (0.3 to 0.5)	-16.3 (-35.8 to 9.5)	4050 (3150 to 5210)	8.6 (6.7 to 11.1)	-17.8 (-37.7 to 10.2)
Turkmenistan	Both	51 (40 to 65)	1.2 (0.9 to 1.5)	-3.6 (-23.9 to 22.2)	39 (31 to 49)	1 (0.8 to 1.2)	-8.8 (-28.1 to 15)	1170 (910 to 1500)	26.3 (20.7 to 33.6)	-8.8 (-28.8 to 15.3)
	Male	36 (28 to 46)	1.9 (1.5 to 2.4)	5.8 (-17.2 to 34.5)	28 (22 to 36)	1.6 (1.2 to 2)	0.4 (-20.7 to 27)	853 (666 to 1090)	42 (32.9 to 53.2)	0 (-22.4 to 28.1)
	Female	14 (11 to 20)	0.6 (0.5 to 0.8)	-21.4 (-39.6 to 5.3)	11 (8 to 14)	0.5 (0.4 to 0.6)	-26.4 (-43.3 to -2.8)	318 (241 to 435)	13.4 (10.2 to 18.1)	-26.5 (-44.1 to -2.7)
Tuvalu	Both	0 (0 to 0)	1.1 (0.8 to 1.5)	-2.4 (-19.7 to 20.4)	0 (0 to 0)	1 (0.7 to 1.3)	-5.3 (-21.3 to 16.2)	2 (2 to 3)	22.7 (16.3 to 30.7)	-5.6 (-23.2 to 17.3)
	Male	0 (0 to 0)	1.9 (1.3 to 2.5)	-2.4 (-21.8 to 22.3)	0 (0 to 0)	1.7 (1.2 to 2.2)	-5.4 (-23.7 to 17.9)	2 (1 to 2)	36.5 (25 to 49.7)	-5.5 (-25.1 to 18.8)
	Female	0 (0 to 0)	0.5 (0.3 to 0.6)	-7.6 (-30.1 to 22)	0 (0 to 0)	0.4 (0.3 to 0.5)	-10.1 (-31.7 to 17.7)	1 (0 to 1)	10.6 (7.5 to 14.8)	-10.9 (-32.9 to 18.6)
Uganda	Both	230 (178 to 290)	1.5 (1.2 to 1.9)	-4.9 (-23.5 to 16.5)	209 (162 to 262)	1.4 (1.1 to 1.8)	-6.4 (-24.2 to 14.2)	6330 (4790 to 8070)	38.5 (29.8 to 48.7)	-6.8 (-25.6 to 16.4)
	Male	175 (135 to 228)	2.6 (2.1 to 3.4)	-6 (-25.1 to 16.9)	159 (123 to 206)	2.5 (2 to 3.2)	-7.1 (-25.6 to 14.4)	4830 (3680 to 6280)	65.6 (51 to 85)	-8 (-27.6 to 16.7)
	Female	55 (41 to 71)	0.7 (0.5 to 0.8)	1.5 (-21.2 to 31.3)	50 (37 to 63)	0.6 (0.5 to 0.8)	-0.6 (-22.1 to 27.2)	1500 (1100 to 1960)	16.4 (12.2 to 21)	-0.5 (-23.7 to 30.8)
Ukraine	Both	2890 (2260 to 3660)	4.1 (3.2 to 5.2)	16 (-10.6 to 49.3)	1750 (1360 to 2170)	2.4 (1.9 to 3)	10.3 (-14.5 to 37.3)	51200 (39800 to 63900)	73.2 (56.9 to 91.2)	14.6 (-11.1 to 44.5)
	Male	2760 (2140 to 3540)	9.3 (7.2 to 11.9)	13.8 (-12.9 to 46.2)	1690 (1300 to 2120)	5.7 (4.4 to 7.1)	8.1 (-16.7 to 35.2)	49600 (38200 to 62600)	167.2 (128.8 to 210)	12.6 (-13.1 to 42.2)
	Female	127 (97 to 175)	0.3 (0.2 to 0.4)	12.6 (-15 to 47.3)	59 (45 to 80)	0.1 (0.1 to 0.2)	3.7 (-19.9 to 33.5)	1620 (1250 to 2190)	4.3 (3.3 to 5.8)	10.4 (-15.8 to 43.8)
United Arab Emirates	Both	228 (148 to 346)	4 (2.8 to 5.6)	-11.1 (-29.7 to 13.2)	145 (94 to 219)	3.1 (2.2 to 4.2)	-18.5 (-34.4 to 3.4)	5470 (3460 to 8480)	76.4 (52.8 to 108)	-14.4 (-33 to 10.4)
	Male	213 (134 to 327)	5.2 (3.5 to 7.2)	-8.4 (-28.4 to 19.2)	136 (87 to 207)	3.9 (2.8 to 5.5)	-14.9 (-32.6 to 9.5)	5110 (3170 to 8090)	96.5 (65.8 to 138.4)	-12.9 (-32.8 to 13.7)
	Female	15 (10 to 22)	1.1 (0.8 to 1.6)	-44.5 (-57.9 to -25.9)	9 (6 to 13)	0.8 (0.5 to 1.1)	-52 (-63.3 to -36.9)	361 (242 to 521)	21.1 (14.3 to 29.2)	-43.1 (-56.7 to -24.5)
United Kingdom	Both	3180 (2610 to 3850)	2.7 (2.2 to 3.3)	0.2 (-18 to 22)	962 (900 to 1000)	0.8 (0.7 to 0.8)	-4.6 (-7.2 to -2)	21200 (20000 to 22200)	18.6 (17.7 to 19.5)	-6.8 (-9.7 to -3.8)
	Male	2330 (1820 to 2960)	4.2 (3.3 to 5.3)	-1.1 (-22.8 to 25.5)	774 (733 to 805)	1.3 (1.3 to 1.4)	-6.3 (-9.3 to -3.3)	16900 (16100 to 17700)	31 (29.6 to 32.5)	-8.3 (-11.7 to -4.9)

	Female	857 (646 to 1070)	1.4 (1.1 to 1.8)	0.9 (-20.5 to 26.6)	188 (146 to 203)	0.3 (0.2 to 0.3)	-3.4 (-7.3 to 0.4)	4300 (3510 to 4710)	7.4 (6.3 to 8)	-3.6 (-8.4 to 1.3)
Tanzania	Both	351 (266 to 493)	1.4 (1 to 1.9)	-3.6 (-22.6 to 19.1)	322 (245 to 451)	1.3 (1 to 1.8)	-5.1 (-23 to 16.6)	9390 (7010 to 13300)	34 (25.7 to 47.7)	-6 (-25.6 to 17.6)
	Male	277 (202 to 411)	2.3 (1.7 to 3.3)	-5.1 (-24.3 to 18.5)	255 (189 to 377)	2.2 (1.6 to 3.1)	-6.6 (-25.1 to 15.9)	7410 (5330 to 11000)	55.8 (40.7 to 82.6)	-7 (-26.8 to 18.1)
	Female	74 (57 to 94)	0.5 (0.4 to 0.7)	-1.4 (-23.9 to 26.4)	67 (52 to 85)	0.5 (0.4 to 0.6)	-3.1 (-24.6 to 23.3)	1980 (1490 to 2600)	13.5 (10.3 to 17.4)	-3.5 (-26.7 to 25.4)
Virgin Islands	Both	5 (4 to 7)	2.9 (2.4 to 3.5)	-4.1 (-20.9 to 14.2)	4 (3 to 5)	2.1 (1.7 to 2.4)	-4.4 (-19.7 to 12.8)	94 (76 to 112)	51.6 (41.6 to 61.7)	-3.4 (-20.9 to 15.5)
	Male	5 (4 to 6)	6.1 (4.9 to 7.3)	-1 (-19.1 to 18.7)	4 (3 to 4)	4.3 (3.5 to 5.1)	-1.3 (-17.8 to 17.3)	88 (71 to 105)	106.1 (84.9 to 127.8)	-0.5 (-18.5 to 19.6)
	Female	0 (0 to 0)	0.4 (0.3 to 0.5)	-8.5 (-25.6 to 11.9)	0 (0 to 0)	0.3 (0.2 to 0.3)	-8 (-24.6 to 11.3)	6 (5 to 8)	6.1 (4.9 to 7.6)	-8.6 (-25.8 to 12.2)
United States of America	Both	16200 (13500 to 19500)	3 (2.5 to 3.6)	-5.3 (-21.1 to 13.9)	4930 (4700 to 5150)	0.9 (0.8 to 0.9)	-4.9 (-7.8 to -1.8)	118000 (112000 to 123000)	22.1 (21.1 to 23.2)	-6.8 (-10 to -3.3)
	Male	13600 (11000 to 16700)	5.3 (4.3 to 6.6)	-5.4 (-23.2 to 15.9)	3950 (3760 to 4140)	1.5 (1.5 to 1.6)	-5.1 (-8.6 to -1.5)	94700 (89900 to 99600)	37.7 (35.8 to 39.6)	-6.8 (-10.3 to -2.6)
	Female	2650 (2170 to 3270)	0.9 (0.8 to 1.1)	-8.3 (-24.6 to 12.1)	982 (896 to 1070)	0.3 (0.3 to 0.4)	-8.5 (-12.4 to -3.8)	23100 (21500 to 25200)	8.3 (7.8 to 9.1)	-9.4 (-13.5 to -4.2)
Uruguay	Both	221 (170 to 283)	4.4 (3.4 to 5.7)	-1.6 (-25.8 to 26.5)	137 (124 to 151)	2.6 (2.4 to 2.9)	-6.8 (-16.5 to 3.8)	3260 (2920 to 3610)	67.5 (60.2 to 74.9)	-7.6 (-18 to 5.3)
	Male	200 (152 to 256)	9 (6.9 to 11.6)	-2.4 (-26.6 to 26.4)	123 (110 to 137)	5.5 (4.9 to 6.1)	-7.5 (-17.8 to 4)	2970 (2630 to 3310)	136.6 (121.3 to 152.6)	-8.1 (-19 to 5.1)
	Female	21 (16 to 28)	0.7 (0.5 to 1)	1.6 (-22.5 to 32.4)	14 (12 to 17)	0.4 (0.4 to 0.5)	-3.4 (-16.5 to 11.9)	292 (240 to 343)	10.6 (8.7 to 12.5)	-5.6 (-19.3 to 11.2)
Uzbekistan	Both	497 (414 to 594)	2.1 (1.8 to 2.5)	-28 (-40.4 to -14.3)	367 (306 to 436)	1.7 (1.5 to 2)	-31.6 (-43.4 to -18.9)	11600 (9630 to 13900)	45.1 (37.8 to 53.4)	-31.3 (-43.7 to -18)
	Male	305 (248 to 368)	3 (2.5 to 3.5)	-26.8 (-39.3 to -11.2)	233 (191 to 281)	2.5 (2.1 to 3)	-30.2 (-41.9 to -16.6)	7130 (5820 to 8600)	62.9 (52.2 to 75.1)	-29.7 (-42.1 to -14.4)
	Female	192 (155 to 237)	1.5 (1.2 to 1.8)	-29.7 (-44 to -5.6)	134 (109 to 163)	1.1 (0.9 to 1.4)	-33.9 (-46.5 to -11.9)	4470 (3600 to 5460)	31.3 (25.6 to 38)	-33.4 (-46.7 to -10.7)
Vanuatu	Both	2 (1 to 2)	1.1 (0.7 to 1.5)	1 (-15.9 to 21.6)	2 (1 to 2)	1.1 (0.7 to 1.4)	-0.8 (-17 to 18.7)	43 (29 to 60)	24.1 (16.5 to 33.2)	-0.5 (-19.1 to 20.9)
	Male	1 (1 to 2)	1.7 (1.1 to 2.3)	1.2 (-17.5 to 22.6)	1 (1 to 2)	1.7 (1.1 to 2.2)	-0.5 (-18.5 to 21.1)	35 (22 to 49)	37.2 (24.3 to 51.5)	-0.6 (-20.4 to 22)
	Female	0 (0 to 0)	0.4 (0.3 to 0.6)	0.7 (-22 to 29.6)	0 (0 to 0)	0.4 (0.2 to 0.5)	-0.8 (-22.4 to 27.2)	9 (5 to 13)	9.7 (6.1 to 14.1)	-0.9 (-24 to 29.6)

Venezuela	Both	905 (673 to 1200)	3 (2.3 to 4)	-1.4 (-27 to 30.7)	639 (480 to 837)	2.2 (1.7 to 2.9)	-3.3 (-28 to 27.3)	16000 (11600 to 21300)	52.9 (38.8 to 70.1)	-4.1 (-30 to 28.1)
	Male	784 (577 to 1040)	5.6 (4.2 to 7.5)	-2.7 (-28.4 to 29.7)	552 (411 to 728)	4.1 (3.1 to 5.4)	-4.7 (-29 to 25.7)	13900 (10100 to 18700)	97.5 (71.7 to 129.7)	-5.3 (-31.3 to 27.4)
	Female	120 (90 to 160)	0.8 (0.6 to 1)	2.1 (-23.6 to 34.5)	87 (66 to 115)	0.6 (0.4 to 0.7)	-0.3 (-24.6 to 29.5)	2020 (1500 to 2710)	12.9 (9.6 to 17.2)	-0.1 (-26 to 31.2)
Vietnam	Both	3050 (2310 to 3890)	3.1 (2.3 to 3.9)	17.8 (-5.8 to 45.7)	1840 (1420 to 2320)	1.9 (1.5 to 2.4)	2.7 (-16.2 to 24.9)	52800 (39800 to 68000)	51 (38.9 to 64.9)	2.1 (-19 to 27.1)
	Male	2860 (2140 to 3680)	6.5 (5 to 8.2)	17.4 (-6.3 to 44.8)	1700 (1300 to 2170)	4.2 (3.3 to 5.2)	3.1 (-16 to 25.4)	49200 (36900 to 63900)	106.2 (80.9 to 135.9)	1.6 (-19.6 to 26)
	Female	197 (144 to 260)	0.4 (0.3 to 0.5)	5.1 (-20.1 to 37.5)	137 (101 to 177)	0.3 (0.2 to 0.3)	-6.1 (-27.9 to 21.8)	3580 (2600 to 4770)	6.5 (4.8 to 8.6)	-6.7 (-29 to 22.2)
Yemen	Both	376 (259 to 518)	2.7 (1.9 to 3.7)	4.8 (-16.3 to 30.4)	326 (225 to 444)	2.5 (1.8 to 3.4)	2.9 (-17.4 to 26.9)	9410 (6320 to 13100)	62.7 (43 to 86.1)	2.6 (-18.5 to 28.2)
	Male	299 (197 to 423)	4.5 (3 to 6.2)	5.3 (-17.6 to 31.6)	261 (173 to 367)	4.1 (2.8 to 5.7)	3.1 (-18.8 to 28.1)	7400 (4750 to 10600)	101.5 (66.6 to 143.7)	3.3 (-20 to 31.1)
	Female	77 (53 to 109)	1.1 (0.7 to 1.5)	7.5 (-16.9 to 39)	65 (45 to 91)	0.9 (0.7 to 1.3)	5.2 (-18.2 to 34.4)	2010 (1320 to 2850)	24.9 (17.1 to 35.2)	5.2 (-19.1 to 38)
Zambia	Both	125 (92 to 169)	1.7 (1.3 to 2.3)	-8.7 (-29 to 16.3)	112 (83 to 149)	1.6 (1.2 to 2.1)	-12.9 (-31.4 to 10.4)	3510 (2550 to 4820)	43.8 (32.1 to 58.8)	-13 (-33.5 to 13.2)
	Male	101 (73 to 140)	2.9 (2.1 to 3.9)	-8 (-29.6 to 18.1)	91 (66 to 125)	2.7 (2 to 3.6)	-11.9 (-32.1 to 14.2)	2840 (2030 to 3980)	73 (52.8 to 100.5)	-12.8 (-34.5 to 16.4)
	Female	24 (17 to 33)	0.6 (0.5 to 0.9)	-10.7 (-32.3 to 19.6)	21 (15 to 29)	0.6 (0.4 to 0.8)	-14.2 (-34.7 to 14.4)	673 (468 to 939)	15.9 (11.3 to 22.1)	-15.9 (-37.5 to 14.1)
Zimbabwe	Both	182 (146 to 227)	2.4 (2 to 2.9)	-4.2 (-21.1 to 16.1)	165 (133 to 206)	2.3 (1.8 to 2.7)	-6.7 (-23.5 to 12.4)	5130 (3990 to 6540)	63 (50.2 to 78.9)	-7.2 (-26.2 to 14.8)
	Male	145 (115 to 181)	4.5 (3.7 to 5.5)	0.1 (-19.6 to 22.9)	132 (106 to 163)	4.3 (3.5 to 5.2)	-4 (-22.2 to 17.2)	4130 (3240 to 5280)	118.1 (94.2 to 147)	-5.3 (-25.1 to 18.5)
	Female	37 (26 to 51)	0.9 (0.6 to 1.2)	-12.7 (-34.8 to 17.9)	33 (23 to 45)	0.8 (0.6 to 1.1)	-9.9 (-31.7 to 21)	996 (688 to 1380)	21.6 (15.1 to 29.5)	-10.6 (-34.2 to 23.7)

Table S3. Years lived with disability (YLDs) and years of life lost (YLLs) in 2019 (counts and age-standardised rates), and percent change in age-standardised rates between 2010 and 2019, at global, regional, national levels, and Socio-demographic Index (SDI) quintiles, by sex and for both sexes combined, for tracheal, bronchus, and lung (TBL), and larynx cancer.

95% UIs given in parentheses.

Location	Cause	Sex	YLDs			YLLs		
			Number in 2019	Age-standardised rate per 100,000 in 2019	Percent change in age-standardised rate from 2010 to 2019 (%)	Number in 2019	Age-standardised rate per 100,000 in 2019	Percent change in age-standardised rate from 2010 to 2019 (%)
Global	TBL cancer	Both	544000 (396000 to 700000)	6.6 (4.8 to 8.5)	-4.2 (-12.2 to 3.6)	45300000 (41900000 to 48800000)	545 (503.5 to 587.2)	-7.3 (-14.6 to 0.5)
		Male	365000 (266000 to 469000)	9.6 (7 to 12.3)	-6.8 (-16.1 to 3.1)	31200000 (28300000 to 34300000)	793.3 (718.8 to 869)	-10 (-18.7 to -0.3)
		Female	179000 (129000 to 232000)	4.1 (2.9 to 5.3)	0.9 (-8.7 to 10.6)	14100000 (12800000 to 15500000)	323.5 (294.1 to 356.6)	-1.3 (-10.1 to 7.8)
	Larynx cancer	Both	117000 (86200 to 152000)	1.4 (1 to 1.8)	-1.4 (-8.1 to 6.1)	3150000 (2930000 to 3400000)	37.4 (34.8 to 40.4)	-9.6 (-15.9 to -3.1)
		Male	98300 (72700 to 128000)	2.5 (1.8 to 3.2)	-1.8 (-9.2 to 6.6)	2700000 (2500000 to 2940000)	66.9 (61.9 to 72.8)	-10.3 (-17.4 to -3.3)
		Female	18500 (13100 to 24600)	0.4 (0.3 to 0.6)	0 (-6.8 to 7.6)	445000 (404000 to 493000)	10.3 (9.3 to 11.4)	-5.4 (-13.7 to 4.1)
High SDI	TBL cancer	Both	178000 (130000 to 227000)	9.6 (6.9 to 12.2)	-8.9 (-16.2 to -0.7)	11200000 (10600000 to 11500000)	626.4 (598.5 to 647)	-11 (-13.3 to -8.8)
		Male	108000 (78100 to 138000)	12.6 (9.1 to 16.1)	-12.8 (-21.1 to -3.3)	6920000 (6630000 to 7140000)	823.2 (790 to 849)	-14.8 (-16.8 to -12.6)
		Female	69900 (51000 to 89900)	7.1 (5.2 to 9.2)	-3.9 (-14 to 7)	4240000 (3950000 to 4430000)	454.8 (429.7 to 473.5)	-5.6 (-8.7 to -2.4)
	Larynx cancer	Both	27300 (19500 to 35700)	1.6 (1.1 to 2.1)	-5.3 (-14.1 to 4.9)	315000 (300000 to 329000)	18.5 (17.7 to 19.4)	-10.7 (-13.8 to -7.1)
		Male	22800 (16300 to 29800)	2.8 (2 to 3.6)	-6.8 (-16.6 to 4.4)	267000 (255000 to 280000)	32.8 (31.2 to 34.4)	-12.2 (-15.8 to -8.3)

		Female	4510 (3210 to 6180)	0.5 (0.4 to 0.7)	-3.4 (-12.2 to 6.6)	47600 (43600 to 51700)	5.4 (5 to 5.9)	-8.2 (-12.2 to -3.5)
High-middle SDI	TBL cancer	Both	161000 (116000 to 207000)	7.8 (5.6 to 10.1)	-3.4 (-14 to 7.5)	13800000 (12600000 to 15100000)	672.9 (612.4 to 736.2)	-9.7 (-18.1 to -0.2)
		Male	114000 (81000 to 148000)	12.3 (8.7 to 15.9)	-7.7 (-19.5 to 5.4)	10000000 (8860000 to 11200000)	1052 (931.6 to 1175.3)	-13.4 (-23.5 to -1.9)
		Female	46300 (32700 to 60600)	4.2 (2.9 to 5.4)	6 (-8.6 to 23.2)	3830000 (3380000 to 4360000)	350.9 (310.4 to 399.9)	-0.7 (-13.5 to 14.7)
	Larynx cancer	Both	32900 (23700 to 43300)	1.6 (1.2 to 2.1)	-5.4 (-13.8 to 3.5)	748000 (692000 to 801000)	36.4 (33.7 to 39)	-18.7 (-24.7 to -12.9)
		Male	28800 (20600 to 37900)	3 (2.2 to 4)	-6.9 (-15.8 to 2.9)	674000 (620000 to 726000)	69.9 (64.5 to 75.3)	-19.9 (-26.4 to -13.9)
		Female	4060 (2880 to 5420)	0.4 (0.3 to 0.5)	-1.2 (-10 to 9.2)	73700 (67100 to 81500)	6.9 (6.3 to 7.6)	-13.4 (-21.4 to -4.3)
Middle SDI	TBL cancer	Both	155000 (110000 to 202000)	6.2 (4.4 to 8.1)	3.4 (-10.3 to 19.1)	14700000 (12900000 to 16700000)	573.2 (501.1 to 647.8)	-3.2 (-15.7 to 10.5)
		Male	108000 (75900 to 142000)	9.1 (6.4 to 12)	1.7 (-14.9 to 21.1)	10300000 (8650000 to 12100000)	835.3 (704.2 to 975.8)	-4.7 (-19.8 to 12.3)
		Female	47600 (32700 to 63900)	3.7 (2.5 to 4.9)	9.1 (-7.9 to 26.8)	4420000 (3760000 to 5160000)	333.1 (284 to 387.6)	2.3 (-13.6 to 19.3)
	Larynx cancer	Both	31600 (22900 to 41900)	1.2 (0.9 to 1.6)	8.3 (-2.8 to 21.3)	880000 (798000 to 967000)	33.4 (30.3 to 36.7)	-9.1 (-17.3 to -0.6)
		Male	26200 (19000 to 34800)	2.1 (1.5 to 2.8)	10.3 (-2.5 to 25.8)	750000 (673000 to 832000)	59 (53.1 to 65.4)	-7.7 (-17.3 to 2)
		Female	5420 (3780 to 7310)	0.4 (0.3 to 0.6)	3.2 (-7.7 to 14.5)	129000 (116000 to 145000)	9.7 (8.7 to 10.9)	-11.4 (-21.3 to -0.7)
Low-middle SDI	TBL cancer	Both	39600 (28400 to 51400)	2.9 (2.1 to 3.7)	4.9 (-6 to 15)	4360000 (3940000 to 4780000)	305.3 (276.2 to 334.6)	2.8 (-7.5 to 12.5)
		Male	27400 (19700 to 35600)	4.2 (3 to 5.4)	1.9 (-11.3 to 16.3)	3050000 (2760000 to 3370000)	443.8 (401.4 to 489)	-0.5 (-12.1 to 11.4)
		Female	12200 (8440 to 16300)	1.7 (1.2 to 2.3)	15 (0.2 to 29.2)	1310000 (1130000 to 1500000)	176.8 (153 to 202.7)	14 (-0.1 to 28.8)
	Larynx cancer	Both	18800 (13700 to 24800)	1.3 (1 to 1.7)	2.5 (-8.4 to 15.3)	881000 (782000 to 1000000)	60 (53.4 to 68)	-5.4 (-16.2 to 7.4)

		Male	15500 (11400 to 20600)	2.2 (1.6 to 3)	2.8 (-10 to 17.6)	743000 (648000 to 853000)	104.6 (91.3 to 119.7)	-5.3 (-17.8 to 8.9)
		Female	3310 (2320 to 4530)	0.4 (0.3 to 0.6)	7 (-5.9 to 22.7)	138000 (117000 to 162000)	18.1 (15.4 to 21.2)	0.3 (-13.6 to 17.1)
Low SDI	TBL cancer	Both	10400 (7320 to 14000)	2 (1.4 to 2.7)	3.5 (-7.9 to 16.5)	1210000 (1030000 to 1440000)	217.9 (187.3 to 257.5)	2.7 (-7.8 to 14)
		Male	7740 (5260 to 10600)	3.1 (2.1 to 4.1)	-0.5 (-13.5 to 14.6)	902000 (748000 to 1120000)	334.3 (279.6 to 409.1)	-1.4 (-13.2 to 12.2)
		Female	2680 (1870 to 3620)	1 (0.7 to 1.4)	19 (4.3 to 35.7)	303000 (262000 to 345000)	105.4 (91.4 to 119.5)	19.6 (4.7 to 35.9)
	Larynx cancer	Both	6050 (4300 to 8090)	1.1 (0.8 to 1.4)	-3.5 (-15.3 to 10.5)	320000 (278000 to 370000)	55.3 (48.1 to 63.4)	-8.8 (-19.9 to 4.3)
		Male	4890 (3450 to 6570)	1.8 (1.3 to 2.4)	-3.7 (-17.7 to 12.5)	264000 (223000 to 313000)	92.7 (79 to 109.5)	-8.9 (-22.1 to 6.7)
		Female	1160 (819 to 1580)	0.4 (0.3 to 0.5)	0.1 (-11.8 to 13.8)	56700 (48500 to 65800)	18.7 (16.1 to 21.6)	-5.8 (-17.7 to 8.8)
Central Asia	TBL cancer	Both	3370 (2410 to 4440)	4.3 (3.1 to 5.7)	-6.3 (-17.6 to 4.5)	384000 (346000 to 426000)	469.4 (424.9 to 518.1)	-9 (-17.7 to 0.7)
		Male	2620 (1860 to 3440)	7.8 (5.5 to 10.1)	-8.3 (-20 to 3.8)	303000 (267000 to 340000)	837.4 (743.2 to 931.9)	-11.1 (-20.2 to -1)
		Female	743 (524 to 992)	1.8 (1.2 to 2.3)	3 (-12.5 to 21.8)	81400 (72400 to 91600)	183.5 (163.7 to 205.3)	1 (-10.3 to 12.8)
	Larynx cancer	Both	988 (708 to 1320)	1.2 (0.9 to 1.6)	-13.1 (-22.6 to -2.9)	38100 (34000 to 42900)	45.4 (40.7 to 51.2)	-22.2 (-30 to -13.1)
		Male	754 (542 to 1010)	2.1 (1.5 to 2.8)	-12.3 (-23.5 to -0.7)	30300 (26900 to 34400)	82.4 (73.5 to 93.5)	-21.1 (-28.8 to -11.5)
		Female	233 (164 to 320)	0.5 (0.4 to 0.7)	-14.5 (-26.4 to 0.3)	7830 (6650 to 9250)	16.9 (14.4 to 19.9)	-24.9 (-36.2 to -9.4)
Central Europe	TBL cancer	Both	19100 (13400 to 25000)	9.2 (6.5 to 12.1)	-6.1 (-18 to 6.7)	1880000 (1650000 to 2150000)	936.8 (814.1 to 1070.7)	-9 (-20.8 to 3.5)
		Male	13500 (9500 to 17800)	14.6 (10.2 to 19.2)	-10.7 (-23.9 to 2.4)	1360000 (1170000 to 1570000)	1477.2 (1272.9 to 1699.4)	-13.5 (-25.5 to -0.8)
		Female	5600 (3910 to 7450)	4.9 (3.4 to 6.6)	3.7 (-11.6 to 20.1)	523000 (454000 to 599000)	485.1 (420 to 557.7)	1.4 (-11.9 to 16.7)
	Larynx cancer	Both	4930 (3530 to 6600)	2.6 (1.8 to 3.4)	-4.7 (-18.1 to 9.1)	133000 (114000 to 152000)	69.3 (59.5 to 79.1)	-15.1 (-27.2 to -3.1)
		Male	4360 (3110 to 5820)	4.9 (3.5 to 6.5)	-6.6 (-19.6 to 7.7)	122000 (104000 to 140000)	135.7 (116.3 to 156)	-16.6 (-28.5 to -4.2)
		Female	574 (404 to 794)	0.6 (0.4 to 0.8)	-0.7 (-13 to 14.1)	11300 (9260 to 13200)	11.1 (9.1 to 13)	-12.1 (-23.8 to 2.4)

Eastern Europe	TBL cancer	Both	20600 (14500 to 26800)	6 (4.3 to 7.8)	-6.4 (-17.7 to 5.1)	1960000 (1720000 to 2200000)	582.5 (512.4 to 652)	-11.2 (-21.7 to -0.3)
		Male	16300 (11400 to 21500)	12 (8.4 to 15.7)	-11.4 (-23.7 to 1.9)	1610000 (1380000 to 1840000)	1164.2 (1004.2 to 1334.3)	-15.4 (-27.2 to -3.1)
		Female	4210 (2880 to 5590)	2.1 (1.4 to 2.7)	8.3 (-9.4 to 28.6)	354000 (301000 to 415000)	181 (153.2 to 213.4)	2.6 (-13.5 to 21.4)
	Larynx cancer	Both	5900 (4100 to 8000)	1.8 (1.2 to 2.4)	-3.5 (-16.2 to 10.6)	179000 (154000 to 205000)	54.5 (47 to 62.4)	-16.6 (-27.9 to -4.6)
		Male	5480 (3810 to 7420)	4 (2.8 to 5.4)	-6.3 (-19.7 to 8.4)	170000 (146000 to 196000)	122.8 (105.6 to 141.1)	-18.7 (-30.2 to -6.5)
		Female	417 (288 to 589)	0.2 (0.2 to 0.3)	5 (-7.3 to 20.1)	8680 (7370 to 10400)	4.8 (4 to 5.7)	-9.5 (-23.4 to 9.3)
Australasia	TBL cancer	Both	3910 (2700 to 5350)	8 (5.5 to 11)	-7.9 (-26.1 to 15.2)	233000 (219000 to 246000)	492.9 (465.2 to 517.7)	-9.7 (-13.6 to -5.6)
		Male	2240 (1520 to 3130)	9.6 (6.6 to 13.5)	-12.6 (-31.6 to 11.1)	135000 (127000 to 144000)	596.1 (559.4 to 631.4)	-13.1 (-17.9 to -8.3)
		Female	1670 (1130 to 2340)	6.6 (4.5 to 9.2)	-1.6 (-24.1 to 27.7)	98000 (89700 to 105000)	401.3 (370.1 to 429.1)	-4.9 (-10.2 to 1.2)
	Larynx cancer	Both	575 (374 to 807)	1.2 (0.8 to 1.7)	-7.7 (-27.4 to 17.9)	5960 (5440 to 6470)	12.9 (11.8 to 14)	-13.8 (-21.2 to -6.2)
		Male	490 (321 to 685)	2.2 (1.4 to 3.1)	-8.1 (-28.8 to 18.3)	5140 (4660 to 5640)	23.2 (21 to 25.5)	-14.1 (-22.6 to -5.1)
		Female	86 (55 to 126)	0.4 (0.2 to 0.5)	-5.2 (-26.2 to 21.7)	819 (702 to 953)	3.5 (3 to 4)	-11.6 (-22.1 to 0.1)
High-income Asia Pacific	TBL cancer	Both	41500 (29200 to 54300)	9.1 (6.5 to 11.9)	-9 (-20 to 3.3)	1790000 (1620000 to 1890000)	418.3 (388.1 to 438.4)	-13.7 (-16.2 to -11.2)
		Male	29300 (20800 to 39100)	14.3 (10.2 to 19.1)	-13.2 (-26.5 to 2.6)	1290000 (1190000 to 1360000)	656.5 (612.8 to 689)	-17.6 (-20.5 to -14.8)
		Female	12200 (8490 to 16600)	4.8 (3.3 to 6.4)	-2 (-16 to 13.8)	500000 (426000 to 543000)	215 (193.3 to 229.7)	-6.7 (-10.5 to -3)
	Larynx cancer	Both	4670 (3330 to 6340)	1.2 (0.8 to 1.6)	-6.9 (-18.8 to 6.9)	30400 (27800 to 32700)	7.4 (6.9 to 8.1)	-15.1 (-19.7 to -9.9)
		Male	4070 (2840 to 5550)	2.2 (1.5 to 2.9)	-9.3 (-22.5 to 5.9)	27100 (24900 to 29100)	14.1 (13.1 to 15.2)	-17.1 (-21.9 to -11.7)
		Female	604 (416 to 867)	0.3 (0.2 to 0.5)	-3.7 (-14.3 to 8.3)	3310 (2860 to 3780)	1.6 (1.5 to 1.8)	-14.4 (-19.1 to -8.1)
High-income North America	TBL cancer	Both	69600 (50200 to 90000)	11.1 (8 to 14.4)	-11.2 (-22.4 to 0.8)	4600000 (4400000 to 4750000)	750.9 (719.8 to 774.8)	-11.1 (-13.6 to -8.8)

		Male	37100 (25800 to 49000)	12.8 (8.9 to 17)	-12.7 (-27.1 to 4.7)	2570000 (2490000 to 2650000)	897.1 (867.2 to 925.3)	-12.5 (-14.3 to -9.7)
		Female	32500 (23600 to 43000)	9.7 (7 to 12.9)	-10.1 (-25.7 to 7.5)	2030000 (1900000 to 2140000)	624.8 (586.8 to 659)	-9.7 (-14.3 to -5.4)
	Larynx cancer	Both	10800 (7480 to 14500)	1.8 (1.3 to 2.4)	-5.5 (-19.4 to 10.9)	118000 (114000 to 123000)	19.8 (19 to 20.6)	-7.3 (-10.2 to -4)
		Male	8720 (5920 to 11800)	3.1 (2.1 to 4.2)	-5.8 (-22 to 13.6)	95300 (91100 to 99600)	33.8 (32.3 to 35.3)	-7.2 (-10.9 to -3.3)
		Female	2060 (1420 to 2820)	0.7 (0.5 to 0.9)	-7 (-20.4 to 9.5)	23000 (21300 to 25000)	7.4 (6.9 to 8)	-9.9 (-14 to -4.7)
Southern Latin America	TBL cancer	Both	4490 (2940 to 6440)	5.4 (3.5 to 7.8)	-4.5 (-26.7 to 24.1)	438000 (414000 to 460000)	535.4 (507.2 to 562.5)	-8.1 (-12.7 to -3.5)
		Male	2970 (1910 to 4260)	8 (5.2 to 11.5)	-9.8 (-32 to 17.2)	295000 (280000 to 311000)	792.6 (753.2 to 834.1)	-12.9 (-17.5 to -8.1)
		Female	1530 (984 to 2160)	3.4 (2.2 to 4.8)	7.2 (-20.9 to 44.7)	143000 (132000 to 152000)	322.4 (298.8 to 344.6)	3.2 (-4.4 to 11.3)
	Larynx cancer	Both	1210 (804 to 1720)	1.5 (1 to 2.1)	-4.9 (-27.2 to 23.9)	31700 (29300 to 34400)	38.9 (36 to 42.3)	-15.1 (-21.8 to -7.6)
		Male	1050 (694 to 1490)	2.8 (1.9 to 4)	-6.1 (-28.8 to 23.7)	27800 (25600 to 30300)	74.6 (68.7 to 81.2)	-16.1 (-23.4 to -8.2)
		Female	168 (111 to 245)	0.4 (0.2 to 0.5)	0.7 (-22 to 28.6)	3880 (3420 to 4320)	8.8 (7.8 to 9.8)	-9.4 (-19.4 to 0.8)
Western Europe	TBL cancer	Both	73600 (52200 to 95200)	8.7 (6.2 to 11.3)	-8 (-20.5 to 4.5)	5200000 (4960000 to 5370000)	645.3 (619.4 to 665.7)	-11 (-13.5 to -8.9)
		Male	47900 (33600 to 62400)	12.1 (8.5 to 15.9)	-13.5 (-26.3 to 0)	3480000 (3330000 to 3600000)	908.7 (872.5 to 940.7)	-15.8 (-18.6 to -13.5)
		Female	25700 (18100 to 33600)	5.8 (4.1 to 7.6)	1.4 (-11.7 to 17.2)	1720000 (1620000 to 1790000)	413.2 (393.4 to 428.3)	-1.7 (-4.3 to 1.1)
	Larynx cancer	Both	15100 (10800 to 20400)	1.9 (1.4 to 2.6)	-8.1 (-21.3 to 7.9)	205000 (194000 to 214000)	26.2 (25 to 27.4)	-15.8 (-19.4 to -11.7)
		Male	12900 (9250 to 17600)	3.5 (2.5 to 4.7)	-10.1 (-23.6 to 6.6)	181000 (171000 to 190000)	48.6 (46.2 to 51)	-17.6 (-21.5 to -13.2)
		Female	2140 (1480 to 2910)	0.6 (0.4 to 0.8)	-0.6 (-11.6 to 12.1)	23900 (18900 to 26300)	5.9 (4.9 to 6.5)	-7.1 (-12.3 to -0.1)
Andean Latin America	TBL cancer	Both	1380 (932 to 1930)	2.5 (1.7 to 3.5)	-4.9 (-27.3 to 19.6)	138000 (110000 to 170000)	242.6 (193.5 to 299.3)	-7.7 (-27 to 15.8)
		Male	752 (506 to 1060)	2.8 (1.9 to 4)	-8.3 (-31.2 to 19.2)	75500 (59700 to 94700)	277.3 (219.4 to 347.9)	-10.6 (-30.9 to 13)

		Female	626 (412 to 881)	2.2 (1.4 to 3)	-0.2 (-25.5 to 29.3)	62200 (49700 to 76700)	210.6 (168.6 to 259.4)	-3.6 (-23.8 to 21)
	Larynx cancer	Both	240 (160 to 333)	0.4 (0.3 to 0.6)	-1.9 (-21.2 to 20.8)	8590 (6880 to 10600)	15.2 (12.3 to 18.7)	-12.3 (-29.5 to 6.5)
		Male	187 (125 to 262)	0.7 (0.5 to 1)	-0.7 (-21.4 to 23.9)	6760 (5390 to 8370)	25.1 (20.1 to 31)	-10.8 (-29.2 to 9.2)
		Female	52 (36 to 73)	0.2 (0.1 to 0.2)	-4.4 (-22.9 to 17.6)	1830 (1480 to 2260)	6.1 (5 to 7.6)	-15.8 (-32.2 to 4)
Caribbean	TBL cancer	Both	2650 (1870 to 3570)	5.1 (3.6 to 6.9)	0.5 (-15.7 to 19.6)	251000 (214000 to 296000)	484 (411.5 to 569.3)	-1.4 (-16.1 to 15.1)
		Male	1740 (1200 to 2330)	7.1 (4.9 to 9.5)	-0.2 (-17.3 to 20.5)	164000 (138000 to 193000)	666.5 (561.9 to 782.7)	-2 (-16.4 to 14.9)
		Female	920 (630 to 1250)	3.3 (2.3 to 4.6)	2.6 (-19 to 27.4)	87500 (73900 to 103000)	320.6 (270.8 to 378.2)	0.6 (-14.8 to 17.9)
	Larynx cancer	Both	1160 (803 to 1570)	2.2 (1.5 to 3)	6.6 (-12.9 to 30.7)	36600 (31100 to 42800)	70.3 (59.7 to 82)	1.1 (-14.5 to 18.1)
		Male	991 (686 to 1360)	4 (2.8 to 5.5)	8.3 (-13.3 to 34.9)	32200 (27300 to 37800)	129.8 (110 to 152.1)	2.5 (-13.4 to 19.9)
		Female	167 (115 to 229)	0.6 (0.4 to 0.8)	1.5 (-16.4 to 21.8)	4420 (3780 to 5220)	16.2 (13.9 to 19.1)	-4 (-18.4 to 13.2)
Central Latin America	TBL cancer	Both	6340 (4400 to 8620)	2.7 (1.9 to 3.7)	-1.3 (-16.5 to 16.1)	614000 (519000 to 728000)	257.2 (217.7 to 304.3)	-3.6 (-18.3 to 13.8)
		Male	3640 (2510 to 4920)	3.4 (2.3 to 4.6)	-5.2 (-21.2 to 12.9)	368000 (306000 to 440000)	333.7 (279.4 to 398.5)	-6.8 (-22.1 to 11.8)
		Female	2710 (1840 to 3760)	2.1 (1.5 to 3)	5.5 (-13.1 to 30.7)	246000 (202000 to 298000)	191.7 (157.3 to 231.9)	2.6 (-14.3 to 22.4)
	Larynx cancer	Both	1980 (1400 to 2720)	0.8 (0.6 to 1.1)	-2.3 (-17.5 to 13.9)	59700 (49900 to 71200)	25 (20.9 to 29.7)	-9.6 (-24.3 to 7)
		Male	1500 (1050 to 2090)	1.4 (1 to 1.9)	-1.8 (-18.8 to 16.7)	50100 (41700 to 60000)	45.4 (37.8 to 54.1)	-8.7 (-24.5 to 8.9)
		Female	475 (329 to 667)	0.4 (0.3 to 0.5)	-0.7 (-12.3 to 12)	9560 (7940 to 12700)	7.4 (6.2 to 9.9)	-9.6 (-24 to 6.3)
Tropical Latin America	TBL cancer	Both	8540 (6130 to 11000)	3.5 (2.5 to 4.5)	-6.4 (-12.3 to 0.5)	879000 (836000 to 921000)	355.8 (337.7 to 372.5)	-9.2 (-12.6 to -5.5)
		Male	4950 (3550 to 6350)	4.5 (3.2 to 5.8)	-12.2 (-19.7 to -4.5)	514000 (486000 to 541000)	458 (432.6 to 482.7)	-15 (-19.2 to -10.6)
		Female	3590 (2560 to 4700)	2.7 (1.9 to 3.5)	3.6 (-6.6 to 15.3)	365000 (340000 to 389000)	272.4 (254 to 290)	1 (-4.6 to 6.7)
	Larynx cancer	Both	3960 (2890 to 5230)	1.6 (1.2 to 2.1)	-6.2 (-13.3 to 0.8)	145000 (137000 to 152000)	57.5 (54.5 to 60.4)	-14.4 (-19 to -9.4)
		Male	3320 (2410 to 4390)	2.9 (2.1 to 3.8)	-6.6 (-14.9 to 2.2)	127000 (120000 to 134000)	109.1 (102.8 to 115.3)	-14.4 (-19.5 to -8.9)

		Female	642 (454 to 860)	0.5 (0.3 to 0.7)	-3.2 (-10.4 to 5.3)	17600 (16000 to 19500)	13.1 (11.9 to 14.5)	-12.9 (-19.3 to -4.6)
North Africa and Middle East	TBL cancer	Both	16600 (11800 to 22100)	3.8 (2.7 to 5)	1.1 (-11.5 to 15.1)	1850000 (1630000 to 2100000)	402.9 (355.4 to 455.9)	-1.4 (-12.9 to 11.2)
		Male	12700 (9030 to 16900)	5.7 (4.1 to 7.6)	-3.3 (-16 to 12.1)	1460000 (1280000 to 1660000)	623.5 (548.6 to 707.3)	-5.2 (-16.9 to 8.7)
		Female	3840 (2660 to 5160)	1.8 (1.2 to 2.4)	17.8 (0.2 to 37.9)	394000 (340000 to 456000)	173.1 (149.5 to 199.5)	13.8 (-0.9 to 27.8)
	Larynx cancer	Both	6080 (4420 to 8110)	1.3 (1 to 1.8)	4.4 (-4.5 to 14.9)	203000 (179000 to 231000)	42.9 (38.3 to 48.6)	-8.7 (-15.9 to -0.6)
		Male	4990 (3610 to 6680)	2.2 (1.6 to 2.9)	2.3 (-7.3 to 13.8)	169000 (149000 to 194000)	70.6 (62.9 to 80.2)	-10.2 (-17.7 to -1.4)
		Female	1090 (781 to 1450)	0.5 (0.3 to 0.6)	12.8 (4.5 to 23.1)	33600 (29400 to 38700)	14.1 (12.5 to 16.1)	-1.9 (-9.7 to 7.9)
South Asia	TBL cancer	Both	27300 (18900 to 36400)	1.9 (1.3 to 2.6)	6.5 (-9.3 to 22.9)	3060000 (2590000 to 3520000)	206.6 (174.9 to 237.4)	5 (-10.6 to 20.8)
		Male	19700 (13500 to 26900)	2.8 (1.9 to 3.8)	0.7 (-17.9 to 21.3)	2220000 (1820000 to 2630000)	303.9 (248.5 to 358.8)	-1.3 (-20 to 18)
		Female	7680 (5130 to 10600)	1.1 (0.7 to 1.5)	28.2 (3.3 to 56.5)	839000 (684000 to 1020000)	111.7 (91.2 to 135.7)	29.9 (3.7 to 60.3)
	Larynx cancer	Both	22700 (16300 to 30000)	1.5 (1.1 to 2)	-0.8 (-14.5 to 15.4)	1110000 (957000 to 1300000)	73.2 (63 to 85.1)	-8.4 (-21.8 to 7.2)
		Male	18800 (13400 to 25300)	2.6 (1.8 to 3.4)	-1.5 (-17.8 to 17.3)	940000 (790000 to 1120000)	124.7 (105 to 148.5)	-8.9 (-23.8 to 8.7)
		Female	3880 (2670 to 5320)	0.5 (0.4 to 0.7)	8.4 (-7.9 to 29.2)	172000 (143000 to 207000)	22.3 (18.6 to 26.7)	0.9 (-16.5 to 21.4)
East Asia	TBL cancer	Both	204000 (143000 to 270000)	9.7 (6.8 to 12.8)	2.7 (-15.5 to 23.4)	17400000 (14600000 to 20500000)	816.1 (686.2 to 960.1)	-5.4 (-21.5 to 13.9)
		Male	142000 (96900 to 193000)	14.3 (9.9 to 19.4)	1.4 (-19.8 to 29.1)	12100000 (9580000 to 15100000)	1181.2 (937.1 to 1464)	-6.4 (-27.3 to 18.4)
		Female	62700 (41900 to 84500)	5.8 (3.9 to 7.8)	6.5 (-15.2 to 32.7)	5270000 (4250000 to 6440000)	484 (391.1 to 590.9)	-1.7 (-22.8 to 22.6)
	Larynx cancer	Both	27500 (19000 to 38000)	1.3 (0.9 to 1.8)	13.9 (-6.1 to 39.3)	486000 (401000 to 587000)	22.5 (18.7 to 27.1)	-9.7 (-26.7 to 10.8)
		Male	23000 (15700 to 32300)	2.2 (1.5 to 3.1)	17.5 (-6 to 47.5)	412000 (329000 to 510000)	39.3 (31.6 to 48.3)	-7.1 (-27 to 15.3)

		Female	4480 (3000 to 6140)	0.4 (0.3 to 0.6)	1.3 (-15.9 to 21.2)	73900 (59100 to 90000)	6.8 (5.5 to 8.3)	-19 (-36.7 to -0.1)
Oceania	TBL cancer	Both	339 (208 to 513)	4.7 (3 to 7.1)	4.1 (-12.3 to 23.3)	41600 (31000 to 59100)	534.8 (404.1 to 758.9)	3.6 (-9.6 to 21.3)
		Male	266 (159 to 418)	7.3 (4.5 to 11.3)	2.1 (-15.2 to 24.3)	32900 (23700 to 49200)	820 (605.4 to 1209.8)	1.9 (-12 to 19.6)
		Female	73 (43 to 110)	2.1 (1.3 to 3.2)	11.4 (-13.5 to 42.8)	8700 (6440 to 12300)	233.9 (174.6 to 327.3)	10.7 (-6 to 30.9)
	Larynx cancer	Both	32 (22 to 45)	0.4 (0.3 to 0.6)	-2.3 (-14.9 to 11.4)	1460 (1110 to 1870)	19.2 (14.8 to 24.3)	-4.8 (-18 to 9.7)
		Male	24 (16 to 35)	0.7 (0.5 to 1)	-2.4 (-16.6 to 13.8)	1130 (827 to 1470)	29.8 (22.5 to 38.2)	-4.6 (-18.8 to 11.9)
		Female	7 (5 to 11)	0.2 (0.1 to 0.3)	-3.5 (-19.6 to 16.7)	335 (242 to 448)	8.4 (6.3 to 11.2)	-6.1 (-23.8 to 16.2)
Southeast Asia	TBL cancer	Both	30300 (20700 to 40600)	4.9 (3.4 to 6.6)	2.3 (-10.8 to 16.1)	3400000 (2840000 to 3930000)	528 (442.1 to 610.4)	0.4 (-12.1 to 12.9)
		Male	20700 (14400 to 27700)	7.3 (5.1 to 9.8)	0.5 (-14.3 to 17.5)	2360000 (1970000 to 2740000)	787.9 (665.4 to 914.6)	-1.2 (-15.1 to 13.9)
		Female	9540 (6050 to 14000)	2.9 (1.8 to 4.3)	6.6 (-10.3 to 24.4)	1040000 (789000 to 1310000)	305.6 (230.7 to 383.5)	4.3 (-10.9 to 20.3)
	Larynx cancer	Both	5680 (3960 to 7790)	0.9 (0.6 to 1.2)	8.7 (-4.7 to 24.8)	184000 (158000 to 215000)	28.2 (24.4 to 32.8)	-4.1 (-15.2 to 7.7)
		Male	4800 (3330 to 6610)	1.6 (1.1 to 2.2)	11.3 (-3.6 to 29.6)	159000 (135000 to 189000)	52.2 (44.4 to 61.6)	-2.1 (-14.2 to 10.9)
		Female	880 (620 to 1200)	0.3 (0.2 to 0.4)	-2.9 (-12.4 to 7.9)	25300 (21600 to 29200)	7.4 (6.4 to 8.6)	-14 (-26 to -1.7)
Central Sub-Saharan Africa	TBL cancer	Both	1590 (886 to 2960)	2.9 (1.6 to 5.2)	5.5 (-15.3 to 36)	194000 (125000 to 337000)	326 (211.1 to 557.1)	5.3 (-12.3 to 30.2)
		Male	1200 (621 to 2470)	4.8 (2.6 to 9.7)	0.1 (-20.8 to 35.4)	149000 (88700 to 287000)	549.5 (336.1 to 1042.4)	-0.7 (-16.8 to 23.1)
		Female	385 (242 to 566)	1.3 (0.8 to 1.9)	19.9 (-9.6 to 58.9)	44700 (33100 to 60200)	141.2 (105.8 to 192.1)	23.1 (-2.9 to 56.2)
	Larynx cancer	Both	375 (250 to 523)	0.7 (0.4 to 0.9)	-1.2 (-17.7 to 17.6)	20300 (15700 to 26000)	33.4 (26.1 to 42.2)	-4.7 (-20.8 to 15)
		Male	312 (207 to 438)	1.2 (0.8 to 1.7)	-1.5 (-18.7 to 18.9)	17000 (13000 to 21900)	61.5 (47.9 to 77.9)	-5 (-21.8 to 14.8)
		Female	64 (41 to 91)	0.2 (0.1 to 0.3)	-6.5 (-23.5 to 14.8)	3290 (2480 to 4310)	9.9 (7.6 to 12.8)	-10.8 (-28 to 11.1)
Eastern sub-Saharan Africa	TBL cancer	Both	2580 (1730 to 3650)	1.6 (1.1 to 2.3)	3.3 (-9.4 to 16.8)	296000 (246000 to 362000)	171.7 (144.2 to 208.7)	2.4 (-9.1 to 14.3)
		Male	1840 (1220 to 2630)	2.5 (1.6 to 3.5)	-0.6 (-14.9 to 15.1)	210000 (173000 to 264000)	260.7 (217.5 to 323.9)	-1.7 (-14.2 to 11.9)

		Female	749 (503 to 1050)	0.9 (0.6 to 1.2)	16.7 (0.6 to 36.1)	85700 (69500 to 103000)	91.3 (75 to 108.5)	16.9 (1.2 to 34.5)
	Larynx cancer	Both	1130 (791 to 1540)	0.6 (0.4 to 0.9)	-4.9 (-13.7 to 5.7)	60400 (51100 to 75600)	32 (27.2 to 40.1)	-9.1 (-18.3 to 1)
		Male	893 (620 to 1230)	1 (0.7 to 1.4)	-5 (-14.8 to 6.7)	48500 (40700 to 62800)	53.6 (45.2 to 69.3)	-9.2 (-19 to 2.3)
		Female	239 (165 to 329)	0.2 (0.2 to 0.3)	-2.4 (-11.8 to 8.2)	11800 (9910 to 14000)	11.8 (10 to 13.8)	-7 (-17.2 to 4.2)
Southern sub-Saharan Africa	TBL cancer	Both	2330 (1640 to 3070)	4.1 (2.9 to 5.4)	-10.1 (-20.2 to 3.5)	269000 (242000 to 301000)	451.6 (408 to 505.5)	-13.3 (-22.1 to -1.1)
		Male	1570 (1100 to 2110)	6.5 (4.6 to 8.6)	-13 (-24.5 to 2.7)	187000 (166000 to 215000)	725.7 (648.1 to 831.1)	-16.3 (-25.8 to -2.1)
		Female	757 (529 to 991)	2.3 (1.6 to 3.1)	-4.5 (-18 to 11)	81700 (71600 to 91600)	245.7 (216.3 to 274.8)	-6.9 (-18.4 to 7)
	Larynx cancer	Both	548 (396 to 733)	0.9 (0.7 to 1.2)	-15.2 (-22.5 to -6.1)	25200 (22900 to 28000)	40.9 (37.3 to 45.3)	-23.5 (-29.8 to -14.9)
		Male	451 (328 to 605)	1.8 (1.3 to 2.4)	-15.5 (-23.3 to -5)	21300 (19300 to 23800)	80 (72.9 to 89.3)	-24 (-30.4 to -14.7)
		Female	97 (68 to 130)	0.3 (0.2 to 0.4)	-14.9 (-23.9 to -3.9)	3910 (3400 to 4500)	11.3 (9.9 to 12.9)	-22.8 (-32.2 to -11.1)
Western sub-Saharan Africa	TBL cancer	Both	3770 (2570 to 5040)	2.1 (1.4 to 2.7)	2.7 (-12.2 to 20.4)	423000 (351000 to 508000)	215.9 (180.5 to 255.5)	1.8 (-12.6 to 19.7)
		Male	2660 (1790 to 3600)	3.1 (2.1 to 4.1)	1.3 (-16.6 to 25.7)	299000 (243000 to 364000)	325 (267.1 to 393.3)	0.6 (-16.6 to 22.5)
		Female	1100 (743 to 1510)	1.1 (0.8 to 1.5)	17.4 (-4.4 to 41.1)	124000 (99900 to 150000)	115.7 (95 to 138.5)	17.8 (-3.4 to 43.2)
	Larynx cancer	Both	1250 (858 to 1690)	0.6 (0.4 to 0.9)	-6.8 (-22.3 to 12.4)	62500 (49700 to 77300)	30.3 (24.4 to 37)	-10.1 (-26.5 to 10.6)
		Male	1130 (774 to 1540)	1.2 (0.8 to 1.6)	-2.3 (-19.6 to 19)	57600 (45200 to 71700)	58.6 (46.7 to 72.1)	-5.4 (-23.2 to 18.8)
		Female	120 (81 to 166)	0.1 (0.1 to 0.1)	-3.7 (-15.2 to 8.5)	4900 (4050 to 5870)	4.5 (3.8 to 5.3)	-7.5 (-20.4 to 8.2)
Afghanistan	TBL cancer	Both	346 (177 to 584)	2.6 (1.4 to 4.4)	0 (-23 to 40.5)	44200 (24900 to 70900)	299.9 (178 to 477.2)	0.9 (-17.7 to 29.4)
		Male	254 (120 to 471)	4.2 (2.1 to 7.7)	-3.9 (-29.5 to 42.9)	32600 (17400 to 57400)	481.8 (271.3 to 837.8)	-3.6 (-21.3 to 25)
		Female	91 (52 to 148)	1.2 (0.7 to 1.9)	16.8 (-13.4 to 52.3)	11600 (7140 to 17300)	138.5 (91.8 to 197.1)	18 (-8.5 to 51.5)
	Larynx cancer	Both	224 (135 to 342)	1.5 (0.9 to 2.2)	-4.7 (-24.9 to 20.9)	13400 (8800 to 18500)	83.9 (58.2 to 111.9)	-8.2 (-25.7 to 13.2)
		Male	154 (88 to 234)	2.3 (1.3 to 3.4)	-6 (-29.7 to 23.9)	9220 (5530 to 13500)	125.4 (81.4 to 175.7)	-9.5 (-28.2 to 14.5)

		Female	70 (40 to 107)	0.9 (0.5 to 1.3)	-2.4 (-25.1 to 29.1)	4170 (2650 to 5920)	46.5 (31.3 to 63.9)	-6.9 (-28.5 to 23.2)
Albania	TBL cancer	Both	269 (166 to 405)	6.3 (3.9 to 9.5)	15.7 (-18.5 to 58.2)	25700 (18700 to 34500)	608.8 (444.2 to 816.8)	11.3 (-18.2 to 47.6)
		Male	213 (130 to 328)	10.3 (6.3 to 15.7)	15.3 (-20.4 to 60.2)	20600 (14700 to 28400)	1005.8 (722.3 to 1381)	11.4 (-19.2 to 49.1)
		Female	56 (34 to 87)	2.6 (1.6 to 3.9)	20.4 (-18.6 to 73.1)	5060 (3740 to 6630)	239.2 (177.9 to 315.5)	15.9 (-14.1 to 50.4)
	Larynx cancer	Both	73 (46 to 107)	1.7 (1.1 to 2.6)	11.9 (-18.7 to 50.5)	2030 (1450 to 2790)	49 (35.1 to 67.6)	1.3 (-25.4 to 34.4)
		Male	62 (39 to 92)	3.1 (1.9 to 4.6)	13.1 (-19.3 to 55)	1800 (1270 to 2490)	90.1 (63.9 to 123.9)	2.4 (-25.3 to 36.8)
		Female	10 (6 to 15)	0.5 (0.3 to 0.7)	13.3 (-13.6 to 47.3)	226 (167 to 296)	10.9 (8.1 to 14.5)	4.2 (-22 to 35.8)
Algeria	TBL cancer	Both	750 (487 to 1100)	2.2 (1.5 to 3.3)	-2.9 (-27.9 to 31)	80600 (61300 to 104000)	229.6 (176.2 to 295.9)	-4.9 (-25.9 to 22.1)
		Male	573 (355 to 867)	3.4 (2.1 to 5)	-7.3 (-35 to 32.3)	62700 (46000 to 84000)	352.3 (261.1 to 470.4)	-8.5 (-30.7 to 20.5)
		Female	177 (120 to 254)	1.1 (0.7 to 1.5)	11.6 (-17.2 to 48.3)	18000 (14000 to 22400)	101.7 (79.8 to 126)	7.8 (-16.2 to 35.8)
	Larynx cancer	Both	367 (245 to 524)	1 (0.7 to 1.5)	4.8 (-18.6 to 35.4)	11300 (8670 to 14600)	31.6 (24.4 to 40.4)	-10 (-31.2 to 15.1)
		Male	318 (208 to 456)	1.8 (1.2 to 2.6)	4.1 (-20.3 to 38)	10000 (7620 to 13000)	55.1 (42.3 to 70.9)	-10.4 (-31.6 to 17.2)
		Female	49 (33 to 70)	0.3 (0.2 to 0.4)	4 (-17.6 to 28.3)	1300 (989 to 1670)	7 (5.4 to 8.9)	-11.9 (-33.8 to 12.8)
American Samoa	TBL cancer	Both	3 (2 to 4)	5.8 (4.1 to 7.7)	-0.9 (-18.6 to 19.9)	314 (265 to 366)	631.8 (535.6 to 733)	-1 (-14 to 13.7)
		Male	2 (1 to 3)	8.3 (5.8 to 11.3)	-5.2 (-23.9 to 18.5)	220 (184 to 257)	912 (776.9 to 1054.1)	-5.1 (-18.8 to 10.7)
		Female	1 (1 to 1)	3.4 (2.3 to 5)	10.8 (-20.8 to 56)	94 (75 to 119)	370.8 (292.7 to 467.3)	11.5 (-10.9 to 39.8)
	Larynx cancer	Both	0 (0 to 0)	0.4 (0.2 to 0.5)	-21.4 (-35.3 to -3.9)	6 (5 to 7)	12 (10.1 to 14.4)	-25.4 (-37.6 to -8.2)
		Male	0 (0 to 0)	0.7 (0.5 to 0.9)	-23.5 (-38.1 to -5.7)	5 (4 to 6)	22.2 (18.5 to 26.9)	-27.7 (-39.6 to -8.8)
		Female	0 (0 to 0)	0.1 (0.1 to 0.1)	-0.4 (-19.3 to 23.3)	1 (1 to 1)	2.8 (2.2 to 3.5)	-2 (-23.2 to 25.5)
Andorra	TBL cancer	Both	14 (9 to 19)	9.9 (6.4 to 13.9)	-3.8 (-24.4 to 22.6)	1070 (821 to 1360)	773.4 (591.4 to 981.3)	-5.7 (-24.3 to 16.5)
		Male	11 (7 to 15)	15.3 (10.2 to 21.5)	-6.1 (-26.8 to 20.4)	851 (658 to 1080)	1197.5 (925.9 to 1520.8)	-7.7 (-25.4 to 13.7)
		Female	3 (2 to 5)	4.4 (2.7 to 6.7)	9.5 (-25.2 to 56.4)	221 (156 to 299)	329 (232.8 to 444.5)	7.8 (-20.6 to 43.6)
	Larynx cancer	Both	3 (2 to 4)	2 (1.3 to 2.9)	-10.6 (-33.5 to 20)	35 (26 to 46)	24.9 (18.5 to 32.8)	-14.4 (-34.4 to 13.1)
		Male	3 (2 to 4)	3.7 (2.4 to 5.4)	-9.6 (-32.9 to 21.4)	33 (25 to 44)	46.2 (34.3 to 60.8)	-13.5 (-33.9 to 15)
		Female	0 (0 to 0)	0.2 (0.1 to 0.3)	5.6 (-19.6 to 38.6)	2 (1 to 2)	2.3 (1.6 to 3.1)	3.4 (-25.3 to 42.2)
Angola	TBL cancer	Both	367 (238 to 542)	3.2 (2.1 to 4.6)	3.8 (-23.4 to 41.4)	44800 (34200 to 58300)	355.5 (278.1 to 456.2)	2.2 (-17.3 to 31.7)

		Male	276 (177 to 407)	5.3 (3.4 to 7.6)	1.4 (-26.5 to 42.7)	34100 (25800 to 44600)	598.6 (467.6 to 766.8)	0 (-20.2 to 29.2)
		Female	92 (57 to 141)	1.5 (0.9 to 2.2)	20.4 (-17.3 to 82.7)	10700 (7890 to 14600)	156.3 (118.4 to 210.6)	19.4 (-7.8 to 57)
	Larynx cancer	Both	95 (63 to 134)	0.8 (0.5 to 1.1)	-0.7 (-19.8 to 26.1)	5140 (3880 to 6710)	39.9 (31 to 51.5)	-4.7 (-23.6 to 21.2)
		Male	82 (54 to 116)	1.5 (1 to 2.1)	2.8 (-18.1 to 31.6)	4460 (3350 to 5830)	77 (59.6 to 101.1)	-1 (-21.7 to 27.8)
		Female	14 (9 to 20)	0.2 (0.1 to 0.3)	-6.2 (-27.4 to 22.6)	687 (491 to 950)	9.5 (7 to 13)	-12.2 (-33.4 to 16.9)
Antigua and Barbuda	TBL cancer	Both	2 (2 to 3)	2.2 (1.5 to 3)	-2.5 (-24.8 to 25.4)	218 (183 to 255)	207.6 (176.1 to 242.3)	-4.2 (-18.5 to 14.1)
		Male	1 (1 to 2)	3 (1.9 to 4.1)	-12 (-37.2 to 20.2)	137 (114 to 162)	276.3 (231.6 to 325.7)	-13.5 (-27.5 to 6)
		Female	1 (1 to 1)	1.6 (1 to 2.2)	15 (-14.9 to 55.3)	81 (67 to 97)	146.8 (121.6 to 175.6)	13.1 (-7.4 to 39.1)
	Larynx cancer	Both	1 (1 to 2)	1.1 (0.7 to 1.5)	-1.9 (-23.5 to 24.2)	38 (31 to 47)	36.1 (29.5 to 43.9)	-7.2 (-24.9 to 14.4)
		Male	1 (1 to 2)	2.2 (1.5 to 3.1)	-4.7 (-25.7 to 20.9)	37 (30 to 46)	74.1 (60.7 to 89.9)	-9.9 (-27.2 to 11)
		Female	0 (0 to 0)	0.1 (0.1 to 0.1)	3.9 (-13.1 to 24.5)	1 (1 to 1)	1.9 (1.5 to 2.3)	-0.6 (-19.5 to 22.7)
Argentina	TBL cancer	Both	3170 (2060 to 4560)	6 (3.8 to 8.6)	-3.6 (-28.2 to 27.7)	316000 (296000 to 334000)	601.6 (563.9 to 637.5)	-7.3 (-13.3 to -1)
		Male	2140 (1360 to 3100)	9 (5.7 to 13)	-8.3 (-32.8 to 21.7)	217000 (203000 to 231000)	906.5 (848 to 968.2)	-11.6 (-17.5 to -5)
		Female	1030 (643 to 1490)	3.5 (2.2 to 5.1)	7 (-25.2 to 50.2)	99100 (89600 to 109000)	349.8 (316.8 to 384.7)	2.9 (-7.6 to 13.9)
	Larynx cancer	Both	896 (587 to 1270)	1.7 (1.1 to 2.4)	-3.9 (-28.8 to 28.5)	24800 (22700 to 27300)	47.6 (43.4 to 52.4)	-14 (-22.3 to -4.8)
		Male	770 (500 to 1120)	3.2 (2.1 to 4.7)	-5.5 (-31 to 28.9)	21700 (19700 to 24200)	90.9 (82.4 to 101.2)	-15.2 (-24.3 to -5.2)
		Female	126 (81 to 185)	0.4 (0.3 to 0.6)	2.8 (-23.1 to 35.4)	3090 (2670 to 3510)	10.9 (9.4 to 12.4)	-7.5 (-20.1 to 5.7)
Armenia	TBL cancer	Both	307 (214 to 416)	7.3 (5 to 9.9)	-9.5 (-29.2 to 12.9)	32200 (26800 to 38300)	764.8 (638.2 to 910.9)	-12.5 (-26.7 to 4.8)
		Male	251 (172 to 340)	13.7 (9.4 to 18.6)	-10.6 (-31.5 to 14.1)	26700 (22200 to 32000)	1443.1 (1204.7 to 1731.7)	-13.3 (-27.7 to 3.8)
		Female	56 (37 to 80)	2.4 (1.6 to 3.4)	-4.2 (-30.2 to 26.8)	5480 (4510 to 6520)	240.2 (196.6 to 286.1)	-8.1 (-25.1 to 10.9)
	Larynx cancer	Both	78 (52 to 107)	1.8 (1.2 to 2.5)	-7.1 (-28.8 to 21.1)	2700 (2200 to 3280)	63.7 (52.2 to 77)	-17.6 (-33.1 to 0.2)
		Male	69 (46 to 95)	3.7 (2.5 to 5.1)	-7.4 (-30.3 to 23)	2460 (1990 to 2990)	131.7 (107.2 to 159.4)	-17.7 (-33.1 to 0.4)
		Female	9 (6 to 13)	0.4 (0.3 to 0.6)	-6.7 (-25.3 to 13.7)	243 (193 to 295)	10.5 (8.4 to 12.9)	-17.1 (-33 to 0.8)
Australia	TBL cancer	Both	3290 (2240 to 4680)	8 (5.4 to 11.4)	-8.6 (-30.5 to 18.5)	193000 (180000 to 204000)	483.6 (453.6 to 511)	-10.1 (-14.6 to -5.1)

		Male	1920 (1270 to 2720)	9.8 (6.5 to 14)	-13.4 (-36 to 15.2)	115000 (107000 to 122000)	598.7 (557.8 to 638.4)	-13.5 (-19.2 to -7.8)
		Female	1370 (912 to 2000)	6.4 (4.3 to 9.3)	-2 (-29.2 to 34.1)	78400 (71100 to 85000)	380.7 (347.9 to 412.1)	-5 (-12.1 to 2)
	Larynx cancer	Both	506 (325 to 720)	1.3 (0.8 to 1.9)	-8.1 (-30.3 to 19.9)	5200 (4680 to 5710)	13.4 (12.1 to 14.6)	-14.1 (-22.2 to -5.3)
		Male	426 (271 to 613)	2.3 (1.4 to 3.3)	-8.7 (-32.6 to 20.7)	4560 (4070 to 5030)	24.4 (21.9 to 27)	-14.4 (-23.9 to -4.1)
		Female	80 (51 to 118)	0.4 (0.3 to 0.6)	-5.2 (-27.8 to 23.9)	645 (542 to 763)	3.2 (2.7 to 3.8)	-11.4 (-24.3 to 2.6)
Austria	TBL cancer	Both	1300 (875 to 1790)	7.9 (5.3 to 10.9)	-10.2 (-29.6 to 13.1)	89000 (84000 to 93900)	558.3 (526.3 to 586.7)	-13.7 (-18.9 to -8.8)
		Male	769 (508 to 1070)	10.1 (6.7 to 14)	-17.7 (-37.9 to 5.8)	55400 (51600 to 59200)	734.7 (684.3 to 784.1)	-20.5 (-26.6 to -14.8)
		Female	527 (356 to 756)	6.1 (4.1 to 8.7)	2 (-24.7 to 36.7)	33600 (30800 to 36500)	406.2 (374.2 to 439.2)	-1.4 (-9.5 to 7.6)
	Larynx cancer	Both	204 (133 to 289)	1.3 (0.9 to 1.9)	-12 (-30.8 to 12.1)	3290 (2990 to 3630)	21.4 (19.5 to 23.7)	-19.7 (-27.8 to -10.2)
		Male	177 (114 to 253)	2.4 (1.6 to 3.4)	-13.8 (-34 to 10.7)	2900 (2600 to 3230)	39.5 (35.5 to 44.2)	-21.1 (-30 to -10.9)
		Female	27 (18 to 39)	0.4 (0.2 to 0.5)	-5.5 (-23.7 to 18.7)	399 (339 to 469)	5 (4.2 to 5.9)	-13.2 (-25.2 to 1.2)
Azerbaijan	TBL cancer	Both	563 (346 to 817)	5.4 (3.4 to 7.7)	0.5 (-24.5 to 29.7)	66800 (48800 to 85700)	610.1 (449.5 to 772)	-1.9 (-21.2 to 20.5)
		Male	453 (259 to 676)	9.5 (5.6 to 14)	0.3 (-29 to 38.1)	54600 (36700 to 73100)	1073.7 (731.9 to 1424.9)	-1.9 (-24.9 to 27.1)
		Female	109 (67 to 161)	2.1 (1.3 to 3)	2.9 (-30.1 to 45.8)	12300 (9360 to 15500)	220.4 (171.1 to 278.1)	-0.7 (-22.2 to 25.6)
	Larynx cancer	Both	165 (108 to 240)	1.5 (1 to 2.2)	-0.5 (-23 to 26.7)	7000 (5390 to 9250)	63.2 (49.5 to 84)	-8.8 (-27.3 to 15)
		Male	132 (84 to 199)	2.7 (1.7 to 3.9)	0.7 (-25 to 37.1)	5850 (4330 to 7990)	114.2 (86 to 155.5)	-7.1 (-27.7 to 21.3)
		Female	33 (22 to 48)	0.6 (0.4 to 0.9)	-3.8 (-24.4 to 21.8)	1150 (864 to 1530)	20.4 (15.5 to 27.1)	-14.6 (-35.4 to 12.4)
Bahamas	TBL cancer	Both	14 (9 to 19)	3.4 (2.3 to 4.7)	-4.6 (-26.8 to 25.5)	1470 (1180 to 1840)	351.3 (284 to 439)	-5.1 (-23.2 to 18.9)
		Male	10 (6 to 14)	5.2 (3.4 to 7.4)	-6 (-31.2 to 26.8)	1040 (827 to 1320)	538.6 (433.5 to 680.8)	-6.6 (-26.2 to 17.6)
		Female	4 (3 to 6)	1.9 (1.2 to 2.7)	-2 (-31.9 to 41.5)	432 (341 to 540)	193.1 (153.1 to 241.4)	-2 (-21.6 to 22)
	Larynx cancer	Both	6 (4 to 9)	1.6 (1 to 2.3)	0.8 (-22.6 to 31.2)	260 (206 to 328)	61.3 (48.7 to 76.8)	-0.1 (-21.3 to 26.2)
		Male	6 (4 to 9)	3.1 (2 to 4.5)	0.5 (-23.9 to 33.5)	241 (190 to 306)	123.7 (98 to 155.6)	-0.3 (-21.6 to 26.4)
		Female	1 (0 to 1)	0.3 (0.2 to 0.4)	0.3 (-18.8 to 22.9)	19 (15 to 23)	8.3 (6.6 to 10.6)	-1 (-21.8 to 23.3)
Bahrain	TBL cancer	Both	33 (21 to 49)	4.2 (2.8 to 5.9)	-17 (-37.8 to 7.6)	3510 (2640 to 4710)	387.9 (292 to 498.4)	-19.3 (-37 to 1.7)

		Male	24 (15 to 37)	5.4 (3.5 to 7.9)	-22.5 (-45.6 to 5.2)	2650 (1910 to 3670)	513.5 (382.4 to 678)	-24.6 (-43.3 to -0.7)
		Female	9 (6 to 12)	2.7 (1.8 to 3.8)	-9.2 (-35.8 to 24.3)	861 (675 to 1060)	235.8 (188 to 286.4)	-12.6 (-30.6 to 6.2)
	Larynx cancer	Both	9 (6 to 12)	0.9 (0.6 to 1.3)	-9.3 (-33 to 17.8)	179 (135 to 233)	20.6 (15.3 to 26.6)	-23.5 (-40.9 to -2.4)
		Male	8 (5 to 11)	1.5 (1 to 2.2)	-14.5 (-39.1 to 15)	166 (124 to 217)	35.1 (25.8 to 45.4)	-28 (-45.1 to -7)
		Female	1 (1 to 1)	0.2 (0.1 to 0.3)	-6.7 (-22.2 to 10.6)	13 (10 to 17)	3 (2.4 to 3.8)	-22.7 (-39.9 to -1.8)
Bangladesh	TBL cancer	Both	2280 (1250 to 4050)	1.7 (1 to 3.1)	-2.7 (-34 to 36.8)	244000 (157000 to 382000)	180 (116.6 to 281.8)	-3.7 (-28.9 to 25.9)
		Male	1750 (870 to 3180)	2.6 (1.3 to 4.6)	-4.9 (-39.8 to 40.9)	187000 (118000 to 306000)	268.7 (170.3 to 436.7)	-5.9 (-34.2 to 26.2)
		Female	534 (306 to 871)	0.8 (0.5 to 1.4)	15.6 (-12.6 to 48)	56500 (37800 to 80600)	84.5 (56.3 to 120)	16.8 (-10.6 to 48.4)
	Larynx cancer	Both	1720 (1100 to 2590)	1.3 (0.8 to 1.9)	-8.9 (-31.5 to 20.6)	77400 (56400 to 112000)	56.5 (41.3 to 81.6)	-19 (-37.6 to 5)
		Male	1480 (926 to 2330)	2.1 (1.3 to 3.3)	-6.7 (-30.9 to 24.9)	67100 (47700 to 100000)	95.8 (68.2 to 143.3)	-16.7 (-37 to 9.4)
		Female	237 (158 to 350)	0.4 (0.2 to 0.5)	-2.7 (-24.9 to 24.5)	10300 (7650 to 13500)	15 (11.2 to 19.6)	-12.4 (-34.3 to 13.4)
Barbados	TBL cancer	Both	11 (7 to 16)	2.3 (1.5 to 3.2)	2.2 (-24.2 to 33.7)	1040 (840 to 1250)	215.1 (173.9 to 259.3)	0.2 (-19.8 to 22.4)
		Male	7 (5 to 10)	3.3 (2.1 to 4.6)	2.9 (-26.5 to 42.4)	672 (535 to 821)	303.7 (242.2 to 370.2)	0.7 (-21.3 to 24.1)
		Female	4 (3 to 6)	1.5 (1 to 2.1)	-1.4 (-31 to 39.7)	366 (292 to 450)	140.6 (112.2 to 173.2)	-2.3 (-21.9 to 21.5)
	Larynx cancer	Both	5 (3 to 7)	1 (0.7 to 1.5)	9 (-13 to 34.4)	157 (127 to 190)	32.4 (26.2 to 39.2)	4.9 (-15.4 to 27.2)
		Male	4 (3 to 6)	2 (1.3 to 2.8)	8.4 (-15.5 to 36.8)	140 (112 to 170)	62.7 (50.5 to 76.2)	4.2 (-16.4 to 27.4)
		Female	1 (0 to 1)	0.3 (0.2 to 0.4)	2.4 (-14.3 to 22.4)	17 (14 to 21)	6.6 (5.3 to 8)	-1.1 (-20.2 to 21)
Belarus	TBL cancer	Both	890 (575 to 1270)	5.6 (3.6 to 8.1)	-11.3 (-35.4 to 19.7)	88600 (67600 to 116000)	566.4 (432.7 to 740.2)	-18.1 (-37.5 to 7.3)
		Male	753 (480 to 1100)	11.9 (7.6 to 17.3)	-14.1 (-39.1 to 16.9)	77100 (58800 to 101000)	1198.8 (920.7 to 1559.6)	-20.3 (-39.2 to 3.4)
		Female	136 (84 to 203)	1.4 (0.9 to 2.1)	1.9 (-33 to 48.1)	11500 (8570 to 15100)	127 (93.8 to 169.2)	-6.1 (-31.2 to 25.5)
	Larynx cancer	Both	323 (202 to 475)	2.1 (1.3 to 3.1)	-2.1 (-30 to 39)	9540 (7250 to 12700)	62.4 (47.5 to 82.5)	-21.5 (-40.7 to 5.8)
		Male	312 (195 to 462)	4.8 (3 to 7.1)	-3.3 (-31.1 to 36.6)	9300 (7040 to 12500)	142.7 (108.8 to 190.4)	-22.1 (-41.1 to 4.5)
		Female	11 (7 to 18)	0.1 (0.1 to 0.2)	0 (-26.2 to 33)	241 (171 to 404)	2.9 (2 to 4.6)	-20.8 (-42.9 to 7.5)
Belgium	TBL cancer	Both	2060 (1360 to 2940)	9.7 (6.4 to 14)	-5.8 (-28 to 23.3)	160000 (150000 to 169000)	783.7 (737.7 to 829.7)	-11.6 (-16.6 to -6.3)
		Male	1420 (937 to 2040)	14.2 (9.4 to 20.4)	-11 (-33.4 to 16.7)	112000 (105000 to 118000)	1141.4 (1071.4 to 1207.8)	-16.5 (-21.2 to -11.3)

		Female	637 (420 to 924)	5.9 (3.9 to 8.6)	4.2 (-23.3 to 41.9)	48300 (44600 to 52300)	471.2 (434.8 to 510.1)	-1.4 (-9.3 to 7.2)
	Larynx cancer	Both	394 (255 to 571)	2 (1.3 to 2.9)	-11.3 (-35.1 to 19.4)	5360 (4860 to 5910)	27.7 (25 to 30.6)	-21.5 (-29.4 to -12.6)
		Male	341 (218 to 493)	3.6 (2.3 to 5.2)	-12.7 (-37.6 to 20)	4560 (4080 to 5080)	48.9 (43.7 to 54.4)	-22.9 (-31.7 to -13.4)
		Female	52 (34 to 77)	0.6 (0.4 to 0.8)	-9 (-30.5 to 17.2)	800 (688 to 936)	8.1 (7 to 9.5)	-18.3 (-29.7 to -3.4)
Belize	TBL cancer	Both	9 (6 to 12)	3.1 (2.1 to 4.3)	0.9 (-23 to 27.9)	970 (826 to 1130)	324.9 (277.9 to 374.9)	0 (-14.7 to 16.5)
		Male	6 (4 to 9)	4.5 (3 to 6.2)	2.5 (-25.2 to 37.7)	708 (593 to 834)	470.4 (395.3 to 551.6)	1.8 (-14.9 to 21.5)
		Female	2 (2 to 3)	1.7 (1.1 to 2.4)	-1.3 (-30 to 38.1)	262 (223 to 309)	175 (148.4 to 205.2)	-2 (-17.3 to 16.7)
	Larynx cancer	Both	3 (2 to 4)	1 (0.7 to 1.4)	3.5 (-14.1 to 25.8)	126 (108 to 147)	41.9 (35.9 to 48.9)	1.3 (-13.4 to 18.4)
		Male	3 (2 to 4)	1.8 (1.3 to 2.5)	5.8 (-13.4 to 30)	114 (97 to 134)	75.3 (64.3 to 88.4)	3.8 (-12 to 22)
		Female	0 (0 to 0)	0.2 (0.2 to 0.3)	-2.3 (-16.2 to 13.6)	11 (9 to 13)	7.5 (6.4 to 8.9)	-5.2 (-19.8 to 11.8)
Benin	TBL cancer	Both	112 (72 to 165)	2.4 (1.5 to 3.4)	0 (-24.2 to 31.4)	12700 (9410 to 16900)	249.8 (189.6 to 328.4)	-0.5 (-19 to 21.9)
		Male	80 (49 to 121)	3.7 (2.2 to 5.4)	-4.1 (-29.4 to 32.9)	9090 (6700 to 12300)	389.2 (293.5 to 516.1)	-4.9 (-22.9 to 17.3)
		Female	32 (21 to 47)	1.2 (0.8 to 1.8)	10.2 (-19.4 to 49.3)	3610 (2660 to 4820)	128.4 (97.5 to 167.9)	10.2 (-11.9 to 36.2)
	Larynx cancer	Both	30 (19 to 43)	0.6 (0.4 to 0.9)	-1.7 (-21 to 24.1)	1490 (1090 to 1980)	29 (21.5 to 38.1)	-5.4 (-23.7 to 16.6)
		Male	27 (17 to 38)	1.2 (0.8 to 1.6)	-2.1 (-22.5 to 25.2)	1330 (960 to 1770)	55.2 (40.3 to 72.6)	-6 (-24.7 to 16.6)
		Female	3 (2 to 5)	0.1 (0.1 to 0.2)	-5.3 (-25 to 18.8)	158 (116 to 214)	5.7 (4.2 to 7.6)	-7.9 (-28.4 to 16.8)
Bermuda	TBL cancer	Both	8 (6 to 11)	6.5 (4.5 to 8.9)	-5.9 (-23 to 15.8)	638 (537 to 773)	514.9 (432.5 to 623.9)	-10.5 (-23.8 to 6.9)
		Male	6 (4 to 8)	10.3 (7.1 to 14.3)	-8.7 (-27.8 to 14.8)	454 (385 to 544)	801.1 (674.5 to 957.9)	-13.4 (-26.5 to 3.6)
		Female	2 (2 to 4)	3.4 (2.3 to 4.9)	-1.5 (-25.4 to 33)	184 (147 to 234)	271.9 (217.8 to 346.4)	-5.3 (-22.1 to 16.2)
	Larynx cancer	Both	2 (1 to 3)	1.7 (1.2 to 2.5)	-0.4 (-23.6 to 28.9)	44 (36 to 54)	36 (29.5 to 44.1)	-9.7 (-26.3 to 10.4)
		Male	2 (1 to 3)	3.5 (2.3 to 4.9)	-2.4 (-26.1 to 28)	42 (34 to 51)	73.6 (60.2 to 89.7)	-11.4 (-27.8 to 8.5)
		Female	0 (0 to 0)	0.3 (0.2 to 0.4)	1.9 (-15.5 to 24.4)	3 (2 to 3)	3.8 (3 to 4.9)	-7.3 (-24.7 to 18.3)
Bhutan	TBL cancer	Both	10 (6 to 15)	1.8 (1.1 to 2.7)	6.4 (-18.5 to 38.8)	1060 (756 to 1460)	182.5 (131 to 251.3)	4.9 (-13 to 26.6)
		Male	7 (4 to 11)	2.5 (1.5 to 4)	4.2 (-23.8 to 41.1)	767 (499 to 1080)	258.6 (166.5 to 367.9)	2.5 (-15.9 to 24.5)
		Female	3 (2 to 5)	1 (0.6 to 1.7)	15.4 (-13.9 to 63.9)	295 (192 to 470)	102.6 (67.6 to 163.6)	15.4 (-5.8 to 45)
	Larynx cancer	Both	7 (4 to 11)	1.2 (0.7 to 1.8)	2.4 (-20.2 to 29.3)	303 (207 to 453)	51.3 (35.2 to 76)	-6.7 (-25.1 to 16.5)
		Male	6 (3 to 9)	1.9 (1.1 to 2.9)	2.9 (-22 to 34.3)	247 (161 to 385)	81.4 (53.6 to 125.5)	-5.8 (-26.6 to 19.4)
		Female	1 (1 to 2)	0.5 (0.3 to 0.7)	4.4 (-21.1 to 38)	57 (40 to 80)	19.4 (13.9 to 26.7)	-5.7 (-27.4 to 23.2)

Bolivia	TBL cancer	Both	261 (152 to 401)	3 (1.7 to 4.6)	7.2 (-20.5 to 40.6)	28000 (18500 to 38200)	308.4 (202.6 to 423.3)	5.1 (-16.4 to 28.4)
		Male	156 (85 to 249)	3.8 (2 to 6)	5.4 (-23.9 to 46.9)	16700 (10600 to 23600)	389.4 (240.9 to 552)	3.5 (-18.1 to 28)
		Female	105 (59 to 163)	2.3 (1.3 to 3.6)	9.7 (-24.7 to 50.7)	11300 (7560 to 15400)	236 (159.1 to 321.9)	7.4 (-14.3 to 31.3)
	Larynx cancer	Both	57 (37 to 83)	0.6 (0.4 to 0.9)	6.9 (-17 to 34.4)	2500 (1900 to 3290)	27.7 (21.1 to 36.1)	-1.1 (-21.7 to 23.7)
		Male	44 (28 to 66)	1.1 (0.7 to 1.6)	8.6 (-18.8 to 42.3)	1950 (1430 to 2580)	45.8 (33.6 to 60.2)	0.8 (-21.9 to 28.2)
		Female	13 (8 to 19)	0.3 (0.2 to 0.4)	0.6 (-19.6 to 25.9)	545 (412 to 722)	11.3 (8.6 to 14.9)	-7.7 (-26.4 to 17.5)
Bosnia and Herzegovina	TBL cancer	Both	548 (350 to 786)	9.1 (5.8 to 13)	-2.1 (-28.5 to 28.3)	56200 (43100 to 71200)	948.8 (728.7 to 1202.7)	-4 (-26.6 to 22.5)
		Male	415 (264 to 598)	15.2 (9.7 to 21.7)	-6.1 (-33.3 to 24.3)	43400 (33100 to 54700)	1581.3 (1211.9 to 1998.7)	-7.7 (-30.2 to 18.3)
		Female	133 (84 to 195)	4.1 (2.6 to 6.1)	10.1 (-24.2 to 55.4)	12800 (9980 to 16500)	412.3 (316.9 to 530.9)	8.4 (-16.6 to 39.9)
	Larynx cancer	Both	115 (75 to 163)	1.9 (1.3 to 2.8)	5.3 (-22.1 to 37.6)	3600 (2750 to 4670)	60.7 (46.2 to 78.9)	-1.6 (-24.4 to 27.2)
		Male	100 (64 to 144)	3.7 (2.4 to 5.3)	4.6 (-24.2 to 38.6)	3250 (2460 to 4200)	119.1 (90.7 to 155.1)	-2.2 (-25.2 to 27.1)
		Female	15 (10 to 21)	0.5 (0.3 to 0.7)	3.9 (-17.2 to 32.7)	350 (272 to 451)	11.2 (8.7 to 14.5)	-2.7 (-25.9 to 26.4)
Botswana	TBL cancer	Both	68 (42 to 104)	4.8 (3 to 7.2)	-1.1 (-26.1 to 31.1)	8260 (5580 to 11300)	541.3 (374.3 to 721.8)	-3.9 (-24.4 to 21.1)
		Male	48 (29 to 72)	7.7 (4.8 to 11.3)	-5.6 (-29.7 to 26.2)	5920 (4040 to 7780)	878.1 (615.7 to 1121.1)	-8.2 (-27.2 to 14.8)
		Female	21 (11 to 35)	2.7 (1.4 to 4.4)	7.3 (-29.2 to 63.6)	2340 (1450 to 3550)	282.3 (178 to 424.6)	5.5 (-21.4 to 46.6)
	Larynx cancer	Both	20 (13 to 30)	1.3 (0.8 to 1.9)	-6.3 (-29.9 to 22.3)	889 (616 to 1240)	55.9 (39.5 to 76.8)	-16.1 (-35.2 to 6.1)
		Male	16 (10 to 25)	2.4 (1.5 to 3.7)	-8.3 (-31.5 to 22.3)	745 (513 to 1050)	106.7 (76.1 to 147.4)	-17.7 (-36.4 to 3.1)
		Female	4 (2 to 6)	0.4 (0.2 to 0.7)	-0.5 (-29.6 to 42.3)	145 (90 to 217)	16.3 (10.4 to 24)	-9.7 (-36.1 to 30)
Brazil	TBL cancer	Both	8340 (5980 to 10700)	3.5 (2.5 to 4.5)	-6.6 (-12.4 to 0.3)	858000 (816000 to 898000)	355.6 (337.5 to 372.4)	-9.4 (-12.8 to -5.7)
		Male	4810 (3440 to 6180)	4.5 (3.2 to 5.8)	-12.5 (-20.1 to -4.7)	498000 (472000 to 525000)	455 (429.7 to 480)	-15.4 (-19.5 to -10.8)
		Female	3540 (2520 to 4620)	2.7 (1.9 to 3.5)	3.5 (-7 to 15.1)	360000 (336000 to 384000)	274.8 (256.3 to 292.9)	1 (-4.6 to 6.7)
	Larynx cancer	Both	3910 (2840 to 5160)	1.6 (1.2 to 2.1)	-6.5 (-13.7 to 0.8)	143000 (135000 to 150000)	58 (54.9 to 60.9)	-14.7 (-19.3 to -9.8)
		Male	3270 (2370 to 4320)	2.9 (2.1 to 3.9)	-6.9 (-15.3 to 1.8)	125000 (118000 to 132000)	110.1 (103.6 to 116.2)	-14.7 (-19.9 to -9.2)
		Female	637 (451 to 854)	0.5 (0.3 to 0.7)	-3.3 (-10.5 to 5.3)	17400 (15800 to 19300)	13.3 (12.1 to 14.7)	-13 (-19.5 to -4.8)

Brunei	TBL cancer	Both	27 (19 to 36)	10.2 (7.3 to 13.4)	1.1 (-13.7 to 18.8)	2550 (2250 to 2890)	857.4 (759.8 to 962.9)	-1.6 (-14 to 12.8)
		Male	15 (10 to 20)	13.2 (9.2 to 17.7)	1.8 (-18.5 to 26.8)	1390 (1150 to 1650)	1073.8 (907.5 to 1245.9)	-2.8 (-18.8 to 14.9)
		Female	13 (9 to 18)	8.6 (5.9 to 11.7)	6 (-19.1 to 33.7)	1160 (970 to 1370)	722.2 (612.4 to 838.7)	3.4 (-12.9 to 21.3)
	Larynx cancer	Both	4 (3 to 6)	1.4 (1 to 1.9)	-5.6 (-22.9 to 14.1)	62 (52 to 76)	20.9 (17.6 to 25.1)	-14.1 (-27.7 to 2.4)
		Male	3 (2 to 5)	2.3 (1.6 to 3.3)	-1.9 (-24.9 to 24.8)	50 (40 to 63)	38.2 (31.4 to 46.9)	-9.7 (-25.9 to 9.7)
		Female	1 (1 to 2)	0.7 (0.5 to 1)	-6 (-24.7 to 15.5)	13 (10 to 15)	8 (6.7 to 9.5)	-16 (-30.5 to 1.9)
Bulgaria	TBL cancer	Both	1120 (743 to 1570)	8.5 (5.6 to 12.1)	-3.4 (-27.4 to 28.6)	115000 (90800 to 145000)	925.8 (724.1 to 1171)	-3.8 (-25.3 to 23.6)
		Male	889 (580 to 1260)	14.9 (9.7 to 20.9)	-7.1 (-31.4 to 25.7)	92200 (72700 to 116000)	1580.6 (1236.9 to 1993)	-7.9 (-28.7 to 19)
		Female	228 (144 to 329)	3.2 (2 to 4.7)	8.6 (-26.7 to 59.6)	23200 (17900 to 29400)	355.3 (272 to 452.3)	9.5 (-16.8 to 41)
	Larynx cancer	Both	371 (239 to 531)	3 (1.9 to 4.3)	4.8 (-24.5 to 42.9)	10500 (8000 to 13500)	87 (66.2 to 111.9)	-6.8 (-30.3 to 21.7)
		Male	348 (223 to 501)	6.1 (3.9 to 8.7)	2.7 (-26.7 to 40.7)	9980 (7570 to 12800)	174.5 (132.8 to 224.2)	-8.7 (-32.1 to 19.7)
		Female	22 (15 to 32)	0.4 (0.2 to 0.5)	12.7 (-12.4 to 45.1)	557 (415 to 729)	8.7 (6.4 to 11.6)	1.9 (-25 to 35.6)
Burkina Faso	TBL cancer	Both	203 (128 to 302)	2.3 (1.4 to 3.3)	15 (-13.2 to 51.4)	23300 (17900 to 31100)	241.8 (187.9 to 325.7)	15.9 (-4 to 41.6)
		Male	151 (92 to 232)	3.8 (2.3 to 5.7)	15.6 (-16 to 60.5)	17500 (12900 to 24100)	405.6 (303.7 to 563.1)	15.7 (-4.9 to 43.3)
		Female	52 (31 to 80)	1 (0.6 to 1.6)	23.2 (-7.3 to 65.4)	5860 (4110 to 8160)	107 (76.5 to 147.4)	27 (4.9 to 56)
	Larynx cancer	Both	66 (43 to 93)	0.7 (0.5 to 1)	9.4 (-13.7 to 39.8)	3400 (2600 to 4370)	34.8 (26.7 to 44)	8.5 (-11.8 to 35.1)
		Male	58 (38 to 84)	1.4 (0.9 to 2)	12.6 (-12.8 to 45.6)	3050 (2280 to 3960)	69.1 (52.5 to 89.2)	11.5 (-10.6 to 40.1)
		Female	7 (5 to 11)	0.1 (0.1 to 0.2)	8.4 (-13 to 38.1)	352 (265 to 451)	6.4 (4.9 to 8.2)	7.4 (-16 to 38.7)
Burundi	TBL cancer	Both	75 (45 to 117)	1.7 (1 to 2.5)	-2 (-25 to 29.8)	8700 (6010 to 12200)	176.4 (123.6 to 241.9)	-2.1 (-21 to 20.1)
		Male	57 (34 to 88)	2.5 (1.5 to 3.7)	-6.6 (-31.2 to 28.4)	6670 (4570 to 9310)	263.5 (184.8 to 364.1)	-6.5 (-24.5 to 15.8)
		Female	18 (10 to 29)	0.8 (0.5 to 1.3)	4.7 (-15.4 to 29.5)	2040 (1340 to 3000)	81.9 (54.6 to 119.7)	4.3 (-16.5 to 30.4)
	Larynx cancer	Both	39 (24 to 58)	0.8 (0.5 to 1.1)	-6 (-25.1 to 18.5)	2190 (1540 to 3110)	40.3 (28.9 to 56.4)	-7.3 (-25.7 to 17)
		Male	31 (19 to 48)	1.2 (0.7 to 1.8)	-7.8 (-27.6 to 17.8)	1760 (1200 to 2600)	62 (43.1 to 90.3)	-8.9 (-28.2 to 15.9)
		Female	8 (5 to 12)	0.3 (0.2 to 0.5)	-8.4 (-29.3 to 16.5)	435 (306 to 609)	16.1 (11.4 to 22.2)	-10.3 (-31.6 to 18)

Cabo Verde	TBL cancer	Both	16 (10 to 22)	3.8 (2.5 to 5.3)	35.7 (-1.3 to 79)	1670 (1340 to 2010)	390.4 (309.9 to 472.2)	31.3 (2.9 to 63)
		Male	10 (6 to 15)	5.8 (3.4 to 8.3)	36.8 (-13.3 to 92.4)	1080 (823 to 1340)	588.7 (433.4 to 730.9)	33 (-3.1 to 71.5)
		Female	6 (4 to 8)	2.5 (1.6 to 3.5)	30.2 (-9.5 to 82.8)	586 (474 to 713)	242.2 (196 to 293.8)	25 (-2 to 57.6)
	Larynx cancer	Both	3 (2 to 5)	0.8 (0.6 to 1.1)	50.7 (21.6 to 85.7)	129 (107 to 160)	29.6 (24.8 to 36.1)	36.6 (10.9 to 65.8)
		Male	3 (2 to 4)	1.7 (1.2 to 2.3)	45.7 (16 to 81.9)	119 (98 to 148)	62.9 (52.3 to 76.8)	32.4 (6.8 to 62.1)
		Female	0 (0 to 0)	0.1 (0.1 to 0.2)	43.9 (14.4 to 82.8)	10 (8 to 12)	4.2 (3.4 to 5.2)	26.6 (0 to 61.5)
Cambodia	TBL cancer	Both	650 (420 to 907)	5.4 (3.6 to 7.6)	6 (-16.1 to 33.9)	75400 (58300 to 93400)	601.4 (470.8 to 744.1)	4.8 (-14.2 to 25.3)
		Male	465 (296 to 669)	9.6 (6.2 to 13.9)	3.3 (-20.8 to 34.4)	54400 (41800 to 68900)	1052.8 (810.3 to 1344.7)	1.9 (-17 to 23.1)
		Female	184 (114 to 265)	2.6 (1.6 to 3.7)	15.5 (-16.6 to 59.2)	21000 (16000 to 27000)	288.1 (219.8 to 369.4)	14 (-8.3 to 39.7)
	Larynx cancer	Both	112 (73 to 163)	0.9 (0.6 to 1.3)	14.7 (-9 to 42.5)	4740 (3530 to 6240)	37.7 (28.2 to 49.2)	5.1 (-15 to 27.7)
		Male	89 (57 to 136)	1.8 (1.1 to 2.7)	18.4 (-9.3 to 53.2)	3870 (2810 to 5200)	74.1 (54 to 99.5)	8.1 (-12.7 to 32.8)
		Female	22 (15 to 32)	0.3 (0.2 to 0.4)	2.6 (-16.1 to 27.4)	870 (651 to 1130)	12 (9 to 15.4)	-6.8 (-26 to 19.5)
Cameroon	TBL cancer	Both	362 (217 to 558)	3.1 (1.9 to 4.6)	2.9 (-23.3 to 35.2)	41900 (29600 to 58600)	329.5 (236.3 to 454.2)	0 (-21.5 to 25.1)
		Male	253 (152 to 398)	4.5 (2.7 to 6.9)	-1.7 (-28.3 to 36)	29400 (21000 to 40700)	485.7 (348.7 to 661.9)	-4.5 (-25 to 21.1)
		Female	109 (63 to 174)	1.8 (1 to 2.8)	16.1 (-20.6 to 70.4)	12500 (8220 to 18300)	183.6 (124.9 to 266)	13.7 (-11.7 to 44.4)
	Larynx cancer	Both	99 (61 to 145)	0.8 (0.5 to 1.2)	-3.7 (-26.9 to 25.9)	4970 (3490 to 6870)	38.5 (28 to 52.7)	-9 (-28.8 to 18.4)
		Male	89 (55 to 133)	1.5 (0.9 to 2.2)	-2.9 (-26.5 to 27.9)	4550 (3190 to 6300)	72.7 (51.9 to 98.9)	-8.1 (-28.6 to 19.7)
		Female	9 (6 to 14)	0.1 (0.1 to 0.2)	-5.8 (-29.3 to 22.9)	422 (292 to 595)	6.3 (4.4 to 8.7)	-12.1 (-34.1 to 16.8)
Canada	TBL cancer	Both	7740 (5130 to 10900)	11.3 (7.4 to 16)	-10.1 (-31.2 to 18)	471000 (441000 to 498000)	705.3 (663.3 to 743.6)	-11.9 (-16.5 to -7.1)
		Male	3970 (2600 to 5770)	12.2 (8 to 17.7)	-13.3 (-35.8 to 16.8)	254000 (236000 to 270000)	794.8 (742.2 to 846.4)	-14.6 (-19.7 to -8.9)
		Female	3770 (2520 to 5290)	10.6 (7 to 15)	-7.3 (-32.3 to 23.9)	218000 (197000 to 236000)	629.1 (570.2 to 680.1)	-9.1 (-17 to -1.7)
	Larynx cancer	Both	1010 (664 to 1460)	1.6 (1 to 2.2)	-5.7 (-28.4 to 20.8)	10300 (9270 to 11300)	15.7 (14.2 to 17.2)	-10.7 (-19.1 to -1.7)
		Male	809 (523 to 1190)	2.6 (1.7 to 3.8)	-6.1 (-31 to 24)	8550 (7610 to 9490)	27.4 (24.5 to 30.2)	-11 (-20.6 to -1.4)
		Female	204 (134 to 300)	0.6 (0.4 to 0.9)	-7.1 (-28 to 20.1)	1730 (1470 to 2090)	5.1 (4.4 to 6.2)	-12.9 (-24.7 to 1.8)

Central African Republic	TBL cancer	Both	67 (30 to 130)	2.8 (1.4 to 5.3)	-8.1 (-31.6 to 21.6)	8770 (4260 to 15600)	341.1 (176.6 to 591.3)	-9.2 (-27.1 to 12.6)
		Male	55 (21 to 112)	5.2 (2.3 to 10.2)	-9.3 (-34.7 to 25.6)	7350 (3120 to 13900)	622.3 (292.5 to 1136.1)	-10.7 (-27.1 to 9.6)
		Female	12 (7 to 19)	1 (0.6 to 1.6)	-2.2 (-26 to 27)	1420 (967 to 2020)	107.1 (73.8 to 149.4)	-2.4 (-26.6 to 27.7)
	Larynx cancer	Both	21 (13 to 31)	0.8 (0.5 to 1.2)	-8.2 (-25.3 to 13.2)	1290 (900 to 1810)	48.7 (34.9 to 66.6)	-9.4 (-27.6 to 11.4)
		Male	17 (10 to 26)	1.6 (1 to 2.3)	-8 (-24.8 to 14.9)	1100 (746 to 1550)	90.3 (64.7 to 123.2)	-9.4 (-28.4 to 11.8)
		Female	3 (2 to 5)	0.3 (0.1 to 0.4)	-9.5 (-32.1 to 19)	191 (126 to 280)	13.5 (9 to 19.6)	-11.1 (-34.7 to 18.9)
Chad	TBL cancer	Both	143 (86 to 223)	2.6 (1.6 to 4)	3.9 (-21.4 to 39.1)	16500 (11600 to 23600)	281.8 (201 to 402.2)	2.5 (-16.6 to 26.7)
		Male	115 (66 to 187)	3.9 (2.2 to 6.3)	-0.8 (-27.2 to 40.4)	13200 (9150 to 19700)	421.7 (295.1 to 629.8)	-1.9 (-20.7 to 22.5)
		Female	28 (17 to 44)	1.1 (0.7 to 1.7)	14.7 (-17 to 61)	3250 (2260 to 4870)	113.7 (79.8 to 169.1)	14.1 (-7 to 43.7)
	Larynx cancer	Both	37 (23 to 53)	0.6 (0.4 to 0.9)	-1.9 (-21.4 to 24.4)	1920 (1430 to 2490)	32.6 (24.5 to 41.6)	-5.2 (-23.5 to 18.4)
		Male	33 (21 to 48)	1.1 (0.7 to 1.6)	-4.1 (-24.4 to 23.1)	1730 (1280 to 2260)	54.2 (40.2 to 69.9)	-7.1 (-26.1 to 17.9)
		Female	4 (2 to 6)	0.1 (0.1 to 0.2)	-4.1 (-24.4 to 19.8)	193 (142 to 260)	6.8 (5 to 9)	-7.4 (-27.6 to 18.9)
Chile	TBL cancer	Both	930 (604 to 1330)	3.8 (2.5 to 5.5)	-3.4 (-28.7 to 28.2)	82700 (77200 to 87900)	343.4 (320.5 to 364.7)	-6.8 (-13.1 to -0.7)
		Male	538 (339 to 790)	4.9 (3.1 to 7.2)	-9 (-36.2 to 23.9)	49300 (45700 to 52900)	447.6 (415.3 to 480.2)	-12.2 (-18.8 to -5.1)
		Female	392 (251 to 564)	3 (1.9 to 4.2)	5.8 (-24 to 51.3)	33400 (30500 to 36200)	256.1 (234 to 277.2)	2.8 (-6.4 to 12.4)
	Larynx cancer	Both	196 (128 to 279)	0.8 (0.5 to 1.2)	-1.7 (-26 to 26.8)	3720 (3380 to 4100)	15.4 (14 to 17)	-15.2 (-23.9 to -6)
		Male	166 (109 to 237)	1.5 (1 to 2.2)	-1.6 (-28.6 to 29.9)	3210 (2880 to 3590)	29.1 (26.2 to 32.5)	-15.4 (-24.8 to -4.9)
		Female	29 (19 to 42)	0.2 (0.1 to 0.3)	-2.3 (-24.9 to 26.2)	511 (438 to 594)	3.9 (3.4 to 4.6)	-13.9 (-25.2 to 0.6)
China	TBL cancer	Both	199000 (139000 to 264000)	9.8 (6.9 to 13)	2.9 (-15.5 to 24.2)	16900000 (14100000 to 20000000)	821.4 (688.6 to 970.5)	-5.4 (-21.8 to 14.4)
		Male	138000 (94600 to 189000)	14.5 (10 to 19.6)	1.7 (-19.8 to 30.2)	11800000 (9260000 to 14800000)	1189.3 (935.9 to 1481.5)	-6.5 (-27.7 to 19)
		Female	60900 (40600 to 82900)	5.8 (3.9 to 7.9)	6.8 (-15.8 to 33.7)	5100000 (4080000 to 6290000)	486.3 (389.2 to 597.9)	-1.8 (-23.5 to 23.5)

	Larynx cancer	Both	26800 (18500 to 36900)	1.3 (0.9 to 1.8)	14.3 (-6.1 to 40.7)	471000 (386000 to 574000)	22.6 (18.6 to 27.4)	-9.7 (-27.2 to 11.2)
		Male	22400 (15200 to 31600)	2.2 (1.5 to 3.1)	18.1 (-5.9 to 49.9)	399000 (315000 to 497000)	39.3 (31.4 to 48.7)	-7.1 (-27.9 to 16.1)
		Female	4410 (2940 to 6060)	0.4 (0.3 to 0.6)	1.4 (-16.1 to 21.6)	72000 (57000 to 88100)	6.9 (5.5 to 8.4)	-19.1 (-37.2 to 0.2)
Colombia	TBL cancer	Both	1510 (962 to 2200)	2.9 (1.8 to 4.1)	-3.8 (-30.6 to 29.5)	135000 (105000 to 174000)	256.3 (199.5 to 330.3)	-9.4 (-30.1 to 17.4)
		Male	768 (468 to 1120)	3.2 (2 to 4.7)	-13.6 (-41.9 to 23.6)	72800 (55300 to 95800)	303.8 (231.2 to 399.7)	-18 (-38.2 to 8.4)
		Female	738 (473 to 1080)	2.6 (1.6 to 3.8)	10 (-22.4 to 58.3)	61800 (48500 to 79300)	216.8 (170.1 to 277.7)	4.5 (-18.5 to 33)
	Larynx cancer	Both	416 (274 to 593)	0.8 (0.5 to 1.1)	-6 (-29.4 to 23)	11400 (8540 to 14900)	21.6 (16.2 to 28.4)	-17.6 (-39.2 to 6.9)
		Male	303 (194 to 443)	1.3 (0.8 to 1.8)	-6.9 (-33.3 to 24.7)	8940 (6660 to 11800)	37.2 (27.7 to 49.2)	-18.1 (-39.9 to 8.9)
		Female	113 (75 to 166)	0.4 (0.3 to 0.6)	-0.1 (-22.6 to 26.6)	2470 (1840 to 3520)	8.6 (6.4 to 12.3)	-12.3 (-33.2 to 11.6)
Comoros	TBL cancer	Both	8 (5 to 13)	1.7 (1.1 to 2.6)	4.9 (-20.9 to 39)	913 (655 to 1250)	180.9 (131.3 to 243.3)	6.5 (-14.6 to 29.8)
		Male	5 (3 to 9)	2.4 (1.4 to 3.9)	1.8 (-29 to 40.5)	580 (402 to 847)	254.4 (181 to 366)	2.6 (-18.4 to 26)
		Female	3 (2 to 4)	1.1 (0.7 to 1.7)	11.8 (-22.7 to 52.8)	332 (230 to 437)	120.9 (84.5 to 158.3)	14.5 (-11.6 to 43.7)
	Larynx cancer	Both	3 (2 to 4)	0.5 (0.3 to 0.8)	2.3 (-19.4 to 29.6)	138 (98 to 188)	26.3 (19.1 to 35.3)	1.5 (-22.5 to 29.7)
		Male	2 (1 to 3)	0.8 (0.5 to 1.3)	2.8 (-21.8 to 34.5)	101 (70 to 140)	41.3 (29.5 to 56.8)	1.8 (-23.4 to 31.7)
		Female	1 (0 to 1)	0.3 (0.2 to 0.4)	-0.5 (-22.9 to 28.8)	37 (27 to 51)	13.3 (9.6 to 17.8)	-1.8 (-26.7 to 31.2)
Congo	TBL cancer	Both	97 (57 to 154)	3.6 (2.2 to 5.6)	5.6 (-20.7 to 39.2)	11800 (8000 to 17300)	403.3 (286.5 to 577.6)	4.1 (-17.2 to 30.9)
		Male	66 (39 to 107)	5.2 (3.2 to 8.1)	-0.3 (-25.2 to 36.7)	8120 (5710 to 11800)	577.3 (425.2 to 821.9)	-1.1 (-20.3 to 24.3)
		Female	31 (16 to 51)	2.2 (1.1 to 3.5)	12.8 (-24.9 to 60.4)	3650 (2180 to 5740)	241.9 (148.9 to 369.7)	10.2 (-16.5 to 42.8)
	Larynx cancer	Both	21 (14 to 31)	0.8 (0.5 to 1.1)	0.2 (-18.9 to 24.2)	1120 (826 to 1490)	37.8 (28.7 to 49.2)	-4.3 (-23.8 to 20.4)
		Male	18 (12 to 25)	1.3 (0.9 to 1.9)	-1.1 (-22 to 24.1)	934 (684 to 1260)	65.3 (49.7 to 86)	-5.1 (-24.9 to 19.7)
		Female	4 (2 to 6)	0.3 (0.2 to 0.4)	-9.7 (-31 to 18.2)	190 (130 to 266)	12 (8.5 to 16.7)	-15 (-37.1 to 13.4)
Cook Islands	TBL cancer	Both	2 (1 to 2)	6.4 (4.4 to 8.7)	-0.9 (-20.6 to 21.4)	164 (138 to 194)	661.6 (557.5 to 790.9)	-1.8 (-16.5 to 14.7)
		Male	1 (1 to 2)	10.6 (7.4 to 14.4)	-1.4 (-22.3 to 25.1)	134 (113 to 159)	1104.8 (928.1 to 1317.9)	-1.9 (-17.3 to 16)
		Female	0 (0 to 0)	2.5 (1.5 to 3.6)	7.4 (-22.1 to 50.5)	30 (23 to 38)	238 (185.4 to 304.4)	5.5 (-15.9 to 32.6)
	Larynx cancer	Both	0 (0 to 0)	0.5 (0.3 to 0.7)	-7.2 (-24.6 to 14)	3 (2 to 3)	10.7 (8.7 to 13.3)	-16.8 (-30.8 to 0.1)
		Male	0 (0 to 0)	0.8 (0.6 to 1.2)	-5.8 (-25.3 to 19.1)	2 (2 to 3)	18.7 (15.2 to 23.4)	-15.7 (-30.7 to 3)
		Female	0 (0 to 0)	0.1 (0.1 to 0.2)	-3.9 (-23.9 to 24.5)	0 (0 to 1)	3.3 (2.4 to 4.3)	-12.9 (-33.9 to 16)

Costa Rica	TBL cancer	Both	119 (75 to 173)	2.3 (1.5 to 3.4)	-3.8 (-29.2 to 28.6)	10400 (8040 to 13300)	202.5 (156.1 to 258.2)	-7.3 (-28.9 to 19)
		Male	72 (44 to 108)	3.1 (1.9 to 4.6)	-7.4 (-35 to 32.6)	6760 (5130 to 8660)	283.4 (215.9 to 361.8)	-9.7 (-31.1 to 17.2)
		Female	47 (29 to 68)	1.7 (1.1 to 2.5)	4.4 (-27.1 to 44.2)	3670 (2820 to 4680)	133 (102.3 to 169.3)	-0.1 (-22.6 to 27.6)
	Larynx cancer	Both	46 (30 to 66)	0.9 (0.6 to 1.3)	-4.6 (-28.2 to 24.6)	1190 (901 to 1550)	23.1 (17.5 to 29.8)	-12.4 (-33.7 to 12.9)
		Male	39 (25 to 56)	1.6 (1.1 to 2.4)	-2.7 (-28.5 to 29.3)	1070 (799 to 1380)	44.7 (33.5 to 57.8)	-10.4 (-32.6 to 16.5)
		Female	7 (5 to 10)	0.2 (0.2 to 0.3)	-5.2 (-22.8 to 17.7)	125 (95 to 163)	4.5 (3.4 to 5.9)	-14.3 (-34.5 to 11.4)
Croatia	TBL cancer	Both	836 (552 to 1170)	10 (6.6 to 14.2)	-11.9 (-33.4 to 14.3)	64100 (50100 to 81400)	801.1 (623.6 to 1025.5)	-17.3 (-35.7 to 6.5)
		Male	605 (406 to 861)	16.2 (10.8 to 23.1)	-16 (-37.8 to 8.2)	48400 (37500 to 61200)	1316.2 (1017 to 1664.8)	-20.6 (-39 to 1.8)
		Female	231 (150 to 334)	5.1 (3.3 to 7.4)	-4.5 (-32.8 to 33.5)	15700 (12100 to 20200)	369.1 (282 to 480.2)	-10.2 (-31.7 to 18.2)
	Larynx cancer	Both	187 (123 to 269)	2.4 (1.6 to 3.5)	-9.1 (-32.5 to 21.3)	4480 (3440 to 5830)	57.4 (44 to 74.8)	-18.3 (-38.3 to 7.8)
		Male	170 (111 to 247)	4.8 (3.1 to 6.9)	-10.5 (-33.8 to 19.1)	4220 (3220 to 5510)	117 (89 to 152.4)	-19.4 (-39.1 to 6.2)
		Female	16 (11 to 24)	0.4 (0.3 to 0.6)	-10.4 (-31.5 to 17.8)	260 (193 to 341)	6.2 (4.6 to 8.2)	-22.5 (-42.5 to 5.1)
Cuba	TBL cancer	Both	1560 (1060 to 2170)	8.3 (5.6 to 11.6)	1.3 (-21 to 26.3)	141000 (114000 to 173000)	756.7 (611.4 to 926.5)	-2.4 (-20.9 to 19.1)
		Male	1000 (658 to 1390)	11.2 (7.4 to 15.6)	1.1 (-22.7 to 29.4)	89200 (71800 to 110000)	1005.1 (806.8 to 1237.9)	-2.9 (-21.5 to 18.5)
		Female	562 (369 to 800)	5.7 (3.7 to 8.1)	3 (-26.5 to 41.9)	51800 (41700 to 63500)	534.3 (431 to 655.8)	-0.5 (-20.1 to 21.3)
	Larynx cancer	Both	791 (526 to 1120)	4.2 (2.8 to 6)	8.1 (-18 to 42.5)	22200 (17700 to 27600)	118.9 (94.6 to 148.1)	-1.1 (-21.9 to 23)
		Male	680 (446 to 971)	7.6 (5 to 10.8)	10.2 (-19 to 49.3)	19700 (15600 to 24600)	220.7 (175.2 to 275.3)	0.6 (-20.8 to 25.5)
		Female	111 (73 to 157)	1.2 (0.8 to 1.6)	2.1 (-20.7 to 30.6)	2510 (1990 to 3150)	25.9 (20.4 to 32.6)	-7.5 (-27.1 to 16.5)
Cyprus	TBL cancer	Both	124 (88 to 168)	6.3 (4.5 to 8.5)	-6.2 (-24.4 to 15)	9550 (8390 to 10800)	490.2 (431.8 to 555.1)	-8.7 (-20.3 to 3.3)
		Male	95 (66 to 130)	10.1 (7 to 13.7)	-10.6 (-30.7 to 14.6)	7400 (6440 to 8460)	786.8 (685.8 to 899.8)	-12 (-23.7 to 1.5)
		Female	29 (19 to 42)	2.9 (2 to 4.1)	-0.1 (-26.2 to 31.8)	2150 (1820 to 2490)	216.3 (183.8 to 250.7)	-3.7 (-19 to 11.5)
	Larynx cancer	Both	27 (18 to 37)	1.4 (1 to 1.9)	-5.4 (-23.6 to 18.8)	331 (279 to 390)	17.3 (14.6 to 20.3)	-17.2 (-30 to -0.7)
		Male	23 (16 to 33)	2.5 (1.7 to 3.6)	-7.3 (-27.4 to 20)	302 (252 to 360)	33 (27.6 to 39)	-18.9 (-32.2 to -1.8)
		Female	3 (2 to 5)	0.4 (0.2 to 0.5)	-6.6 (-17.6 to 6.6)	29 (24 to 36)	3 (2.5 to 3.7)	-19.6 (-34 to -1.4)

Czechia	TBL cancer	Both	1600 (1070 to 2220)	7.7 (5.2 to 10.7)	-16.3 (-34.8 to 8.7)	132000 (107000 to 162000)	656.7 (533.2 to 807.8)	-20 (-35.6 to -1.6)
		Male	1070 (721 to 1490)	11.4 (7.7 to 15.8)	-22 (-40.9 to 2.3)	90900 (74000 to 112000)	978.6 (794 to 1206.8)	-25.3 (-39.7 to -7.6)
		Female	533 (338 to 755)	4.8 (3.1 to 6.8)	-5.3 (-31.8 to 31.4)	41000 (33000 to 50400)	384.9 (309 to 476.6)	-8.8 (-26.8 to 13.6)
	Larynx cancer	Both	303 (204 to 437)	1.6 (1.1 to 2.4)	-9.2 (-31.7 to 18.8)	6460 (5160 to 8040)	35.2 (27.9 to 44.1)	-18.7 (-35.5 to 2.6)
		Male	261 (172 to 376)	3 (2 to 4.3)	-12.8 (-35.3 to 16.1)	5900 (4690 to 7340)	67.5 (53.3 to 84.3)	-21.3 (-37.9 to -0.7)
		Female	42 (27 to 62)	0.5 (0.3 to 0.7)	4.7 (-21.6 to 42.5)	555 (406 to 712)	5.7 (4.2 to 7.3)	-4 (-25.8 to 24.4)
Côte d’Ivoire	TBL cancer	Both	297 (192 to 434)	2.9 (1.8 to 4.2)	1.4 (-24.3 to 34.2)	34800 (25200 to 45900)	306.5 (225.7 to 393.8)	0.3 (-20.7 to 27.3)
		Male	227 (142 to 342)	4.3 (2.7 to 6.4)	-2.6 (-29.4 to 35)	26600 (19000 to 35100)	454.8 (331.1 to 592.8)	-4 (-24 to 23.5)
		Female	70 (44 to 103)	1.4 (0.9 to 2)	14 (-19.9 to 63.5)	8160 (5940 to 10700)	144.8 (110.2 to 185.3)	14.6 (-11.9 to 48.2)
	Larynx cancer	Both	82 (52 to 117)	0.7 (0.5 to 1.1)	-6.6 (-27.1 to 22)	4260 (3090 to 5600)	36.6 (27 to 47.1)	-9.5 (-29.4 to 16.2)
		Male	76 (48 to 109)	1.3 (0.9 to 1.9)	-7.2 (-28.9 to 22.7)	3960 (2840 to 5230)	64.9 (47.4 to 83.7)	-10.2 (-30.2 to 15.8)
		Female	6 (4 to 9)	0.1 (0.1 to 0.2)	-6.5 (-29 to 21.8)	302 (216 to 405)	5.4 (4 to 7.2)	-9.7 (-32.3 to 19.1)
North Korea	TBL cancer	Both	2080 (1350 to 2990)	6.4 (4.2 to 9.1)	-1.9 (-21 to 23)	233000 (176000 to 304000)	705.9 (535 to 912.1)	-3.2 (-18 to 14.8)
		Male	1400 (848 to 2100)	10.3 (6.5 to 15.4)	-6.9 (-28.5 to 22)	160000 (113000 to 219000)	1116.6 (807.6 to 1500.4)	-7.7 (-22.1 to 9.8)
		Female	685 (413 to 996)	3.7 (2.2 to 5.3)	2.2 (-24.4 to 35.2)	73000 (51700 to 97800)	399.4 (279.2 to 540.6)	0.9 (-20.4 to 24.6)
	Larynx cancer	Both	249 (163 to 362)	0.8 (0.5 to 1.1)	-2.5 (-19.9 to 17.7)	8620 (6600 to 11100)	25.7 (19.8 to 32.9)	-8.4 (-25.3 to 9.9)
		Male	203 (132 to 298)	1.4 (0.9 to 2.1)	-4.9 (-23.2 to 16.2)	7050 (5240 to 9270)	47.3 (36.3 to 61.3)	-10.5 (-27 to 7.9)
		Female	46 (29 to 69)	0.3 (0.2 to 0.4)	-7.7 (-24.9 to 16.4)	1570 (1140 to 2150)	8.6 (6.2 to 11.8)	-13 (-31.8 to 11.7)
Democratic Republic of the Congo	TBL cancer	Both	989 (459 to 2130)	2.7 (1.2 to 5.6)	7 (-19.4 to 48.2)	121000 (64700 to 245000)	304.3 (161.3 to 612.7)	7.8 (-12.6 to 39.9)
		Male	757 (303 to 1870)	4.6 (1.9 to 11)	0.4 (-26.8 to 54.9)	94300 (44900 to 218000)	519.9 (251.3 to 1184.4)	0.1 (-18.3 to 31.5)
		Female	232 (131 to 380)	1.2 (0.6 to 2)	22.1 (-13.1 to 74.3)	26700 (16800 to 40200)	126.9 (79.5 to 193.1)	28.4 (-2.7 to 73.1)
	Larynx cancer	Both	224 (143 to 324)	0.6 (0.4 to 0.8)	-1.5 (-21.9 to 23.6)	12100 (8910 to 16300)	29.8 (22.2 to 39.4)	-4.5 (-24.6 to 20.4)

		Male	183 (114 to 271)	1.1 (0.7 to 1.6)	-2.9 (-25 to 23.3)	9970 (7100 to 13800)	54 (39.2 to 72.2)	-5.9 (-27.1 to 20.8)
		Female	41 (26 to 60)	0.2 (0.1 to 0.3)	-5.8 (-26.2 to 20.1)	2150 (1550 to 2910)	9.8 (7.1 to 13.2)	-9.6 (-30.6 to 17.5)
Denmark	TBL cancer	Both	1180 (796 to 1660)	10.5 (7 to 14.8)	-20.9 (-39.8 to 2.1)	84500 (78400 to 91300)	776.1 (722 to 836)	-17.3 (-23 to -11.7)
		Male	609 (397 to 871)	11.3 (7.3 to 16.2)	-25 (-44.6 to 0.7)	43900 (40600 to 47400)	834.2 (772.3 to 899.2)	-21.5 (-27.6 to -15.5)
		Female	571 (378 to 812)	9.9 (6.5 to 14.1)	-16.8 (-38.9 to 9)	40600 (36500 to 44900)	729.3 (657.4 to 802)	-12.8 (-21 to -4.3)
	Larynx cancer	Both	178 (114 to 262)	1.7 (1.1 to 2.6)	-11.3 (-32.7 to 19)	2190 (1980 to 2410)	21.4 (19.4 to 23.6)	-22.1 (-30 to -13)
		Male	143 (91 to 211)	2.9 (1.8 to 4.2)	-12.7 (-35.8 to 20)	1780 (1580 to 1990)	36.1 (32.1 to 40.2)	-23.4 (-32.3 to -13.6)
		Female	34 (23 to 50)	0.7 (0.5 to 1)	-9.3 (-30.8 to 19.1)	405 (340 to 482)	7.6 (6.4 to 9.1)	-18.8 (-30.5 to -5.4)
Djibouti	TBL cancer	Both	15 (7 to 26)	2.4 (1.3 to 4.3)	5.6 (-24 to 43.5)	1730 (1050 to 2940)	264 (168.7 to 441.2)	4.5 (-18 to 30.4)
		Male	11 (5 to 22)	3.5 (1.7 to 6.7)	1.6 (-31.6 to 41.8)	1310 (752 to 2420)	375.8 (224.6 to 670.2)	0.3 (-21.6 to 25.8)
		Female	3 (2 to 5)	1.2 (0.7 to 1.9)	12.8 (-23.9 to 66.6)	415 (284 to 600)	132.2 (94.4 to 186.1)	14.2 (-12.8 to 47.5)
	Larynx cancer	Both	5 (3 to 8)	0.7 (0.5 to 1.1)	-4.1 (-25.1 to 23.9)	259 (175 to 378)	35.4 (25 to 51.3)	-10.1 (-31 to 15.8)
		Male	4 (2 to 7)	1.1 (0.7 to 1.8)	-5.5 (-27.2 to 22.9)	216 (142 to 319)	54.1 (36.9 to 79.7)	-10.9 (-31.5 to 15.1)
		Female	1 (1 to 1)	0.3 (0.2 to 0.4)	-2.9 (-25.7 to 29.8)	44 (28 to 65)	12.8 (8.6 to 18.3)	-9.3 (-33.9 to 25.4)
Dominica	TBL cancer	Both	3 (2 to 5)	3.8 (2.5 to 5.4)	2.1 (-21.3 to 33.1)	354 (283 to 435)	398.4 (319 to 490.9)	2.2 (-15.4 to 24.5)
		Male	2 (2 to 3)	5.4 (3.5 to 7.7)	-1.4 (-26.9 to 35.1)	251 (199 to 313)	563.2 (446.7 to 696.1)	-0.5 (-20.4 to 24.2)
		Female	1 (1 to 2)	2.3 (1.4 to 3.3)	6 (-25.8 to 48.5)	102 (81 to 127)	233.1 (183 to 291)	6 (-14.8 to 31.5)
	Larynx cancer	Both	1 (1 to 2)	1.4 (0.9 to 2)	2.7 (-20.8 to 31.7)	51 (39 to 63)	56.5 (43.9 to 70.8)	4.5 (-16 to 31.1)
		Male	1 (1 to 2)	2.3 (1.5 to 3.4)	1.5 (-23.6 to 31.9)	45 (34 to 56)	98.9 (75.9 to 125.2)	3.5 (-18.2 to 32.8)
		Female	0 (0 to 0)	0.4 (0.3 to 0.5)	-1.7 (-24.7 to 25.7)	6 (5 to 7)	13.1 (10.3 to 16.7)	-0.1 (-19.8 to 26.4)
Dominican Republic	TBL cancer	Both	327 (202 to 494)	3.5 (2.2 to 5.3)	12.6 (-23.3 to 67)	34700 (24400 to 47600)	363.7 (257.6 to 496.7)	14.5 (-20.7 to 62.9)
		Male	213 (128 to 332)	4.8 (2.9 to 7.4)	15.7 (-27.6 to 76.9)	22700 (15700 to 31100)	490.3 (344 to 672.2)	18.4 (-19.7 to 69.4)
		Female	114 (69 to 181)	2.4 (1.4 to 3.7)	8.2 (-32.3 to 67.8)	12100 (8020 to 16900)	244.8 (163.3 to 342.4)	8.7 (-26 to 54.3)
	Larynx cancer	Both	113 (73 to 164)	1.2 (0.8 to 1.7)	25.6 (-11.4 to 77.4)	4700 (3490 to 6220)	48.9 (36.5 to 64.6)	25.8 (-11.4 to 77.1)
		Male	90 (57 to 134)	2 (1.2 to 2.9)	29.7 (-11.5 to 87.6)	3880 (2850 to 5240)	83.3 (61.8 to 112)	30 (-9.2 to 86.4)
		Female	23 (15 to 33)	0.5 (0.3 to 0.7)	15 (-15.7 to 56.4)	812 (599 to 1090)	16.4 (12.2 to 22.1)	13.1 (-19.7 to 58.5)
Ecuador	TBL cancer	Both	339 (221 to 482)	2.3 (1.5 to 3.2)	-3.6 (-29.8 to 29.8)	33600 (26200 to 43300)	219.5 (172 to 281.9)	-6 (-27.7 to 20.9)

		Male	187 (118 to 273)	2.7 (1.7 to 3.9)	-8.3 (-38 to 33)	18500 (14200 to 24300)	253.6 (195 to 329.4)	-10.6 (-32.7 to 18.7)
		Female	152 (99 to 216)	1.9 (1.3 to 2.8)	3.2 (-25.9 to 45)	15100 (11800 to 19200)	188.7 (148.6 to 240)	0.7 (-22.3 to 29.4)
	Larynx cancer	Both	67 (43 to 96)	0.4 (0.3 to 0.6)	0.6 (-23.3 to 32.9)	2340 (1790 to 3070)	15.3 (11.8 to 20.1)	-9.9 (-32 to 19.4)
		Male	54 (34 to 77)	0.7 (0.5 to 1.1)	1 (-25.2 to 36.7)	1890 (1430 to 2520)	25.9 (19.7 to 34.5)	-9.5 (-32.8 to 22.2)
		Female	14 (9 to 20)	0.2 (0.1 to 0.3)	2.6 (-21.1 to 33.1)	448 (340 to 583)	5.6 (4.2 to 7.3)	-8.4 (-31.5 to 20.5)
Egypt	TBL cancer	Both	1480 (859 to 2280)	2.2 (1.3 to 3.3)	5 (-27.3 to 48.3)	174000 (122000 to 237000)	238.7 (167.9 to 324.4)	4.3 (-21.3 to 35.9)
		Male	1010 (557 to 1630)	2.7 (1.5 to 4.3)	-1.2 (-36.9 to 44.9)	120000 (83200 to 170000)	307.7 (211.6 to 433.7)	-1.7 (-27 to 29.3)
		Female	467 (266 to 747)	1.5 (0.8 to 2.5)	20.4 (-19.8 to 68.1)	53000 (34200 to 76100)	160.8 (102.1 to 230.9)	20.3 (-12.5 to 62.1)
	Larynx cancer	Both	499 (317 to 745)	0.7 (0.4 to 1)	1.3 (-24 to 31.9)	19800 (14500 to 26700)	26.5 (19.7 to 36.2)	-9.4 (-31.7 to 19)
		Male	402 (245 to 615)	1 (0.6 to 1.6)	-0.6 (-27.8 to 34.4)	16400 (11800 to 22600)	41.2 (29.7 to 58.1)	-10.9 (-33.4 to 17.8)
		Female	97 (64 to 144)	0.3 (0.2 to 0.4)	5.8 (-19.8 to 35.2)	3420 (2450 to 4720)	9.6 (6.9 to 13.1)	-4.9 (-31.1 to 26.8)
El Salvador	TBL cancer	Both	144 (91 to 209)	2.4 (1.5 to 3.6)	10.2 (-22.2 to 48.1)	13900 (10400 to 18000)	237.7 (177.4 to 308.1)	7.3 (-20.2 to 40)
		Male	71 (44 to 105)	2.8 (1.8 to 4.2)	8.1 (-26.2 to 51.8)	7130 (5300 to 9330)	285.3 (211.3 to 374)	6.9 (-22.3 to 41.4)
		Female	74 (45 to 110)	2.2 (1.3 to 3.2)	13 (-25 to 59.6)	6810 (5030 to 8960)	202.3 (149.1 to 266.4)	8.8 (-21.2 to 42.8)
	Larynx cancer	Both	31 (20 to 45)	0.5 (0.3 to 0.8)	8.8 (-19.6 to 42.7)	1050 (779 to 1390)	18 (13.3 to 23.8)	0.5 (-25.4 to 32.3)
		Male	23 (15 to 34)	0.9 (0.6 to 1.4)	14.4 (-18.8 to 54.1)	835 (614 to 1110)	33.7 (24.7 to 45)	6 (-21.9 to 41.7)
		Female	7 (5 to 11)	0.2 (0.1 to 0.3)	0.5 (-22 to 29)	217 (158 to 290)	6.4 (4.6 to 8.5)	-9.6 (-33.7 to 19.9)
Equatorial Guinea	TBL cancer	Both	18 (10 to 29)	3.6 (2 to 5.8)	10.3 (-20.3 to 49.9)	2080 (1270 to 3250)	397.3 (253.3 to 612.8)	8.6 (-18.2 to 43.4)
		Male	11 (6 to 18)	5.4 (3 to 8.8)	5.3 (-26 to 46.1)	1310 (773 to 2070)	598.3 (368.2 to 921)	4 (-18.9 to 33.3)
		Female	7 (4 to 11)	2.4 (1.3 to 4)	18.2 (-23.6 to 91.2)	766 (435 to 1270)	254.3 (148.5 to 407.8)	16.1 (-21.8 to 70.8)
	Larynx cancer	Both	3 (2 to 5)	0.6 (0.4 to 1)	11.7 (-14.2 to 44.2)	142 (92 to 221)	27.2 (18.3 to 42)	5.7 (-18.8 to 40.3)
		Male	3 (2 to 4)	1.2 (0.7 to 1.9)	15.1 (-12.2 to 50.5)	116 (75 to 185)	53.8 (36 to 84.7)	9.5 (-15.9 to 43.8)
		Female	1 (0 to 1)	0.2 (0.1 to 0.3)	-4.7 (-30.2 to 32.5)	26 (15 to 41)	8.2 (5.1 to 12.9)	-12.3 (-37.9 to 27.3)
Eritrea	TBL cancer	Both	54 (35 to 79)	2 (1.3 to 2.8)	2.7 (-20.8 to 36.3)	6620 (4960 to 8810)	216.5 (166.5 to 277.6)	1.9 (-17.4 to 24.2)

		Male	36 (23 to 54)	3.1 (2 to 4.8)	-4 (-30.3 to 37.6)	4440 (3230 to 5940)	344 (256.8 to 443.9)	-5.7 (-23.5 to 17.4)
		Female	19 (11 to 29)	1.2 (0.7 to 1.8)	12.9 (-18.5 to 49.3)	2180 (1500 to 3090)	126.9 (88.5 to 177.8)	15 (-10.1 to 46)
	Larynx cancer	Both	23 (14 to 34)	0.7 (0.4 to 1.1)	-4 (-21.7 to 19.1)	1320 (906 to 1840)	38.5 (27.2 to 53.1)	-8.5 (-24.5 to 12.7)
		Male	16 (10 to 25)	1.2 (0.7 to 1.8)	-7.6 (-26.1 to 17)	985 (657 to 1440)	64.7 (44.2 to 90.8)	-12.1 (-28.7 to 9.5)
		Female	6 (4 to 9)	0.4 (0.2 to 0.5)	-0.6 (-22.7 to 25.8)	331 (223 to 476)	18 (12.4 to 25.3)	-4.8 (-27.2 to 22.5)
Estonia	TBL cancer	Both	163 (108 to 233)	6.5 (4.3 to 9.4)	-8.7 (-31.1 to 19.3)	14900 (11600 to 19000)	627.8 (486.2 to 798.6)	-11.8 (-31.9 to 11.6)
		Male	112 (75 to 162)	11.4 (7.6 to 16.5)	-19.2 (-40.5 to 6.5)	10900 (8440 to 14000)	1120.4 (863.3 to 1430.5)	-20.5 (-38.7 to 0.7)
		Female	51 (31 to 74)	3.3 (2 to 4.8)	14.4 (-20.7 to 63.7)	4000 (3070 to 5140)	282.3 (215.2 to 366.3)	9.7 (-16.6 to 41.4)
	Larynx cancer	Both	51 (33 to 74)	2.3 (1.5 to 3.3)	3.2 (-26.1 to 42.6)	768 (583 to 991)	34.7 (26.3 to 44.9)	-14.2 (-35.7 to 11.5)
		Male	49 (31 to 71)	5.1 (3.2 to 7.3)	-0.4 (-29.3 to 38.1)	721 (545 to 934)	75.5 (57.2 to 97.6)	-17.2 (-37.9 to 7.1)
		Female	2 (2 to 4)	0.2 (0.1 to 0.3)	-6.9 (-30.6 to 22.7)	48 (36 to 63)	3.7 (2.8 to 5)	-20.1 (-41.5 to 6.1)
eSwatini	TBL cancer	Both	23 (12 to 37)	3.9 (2.1 to 6.1)	-6.6 (-30.4 to 34.2)	2930 (1730 to 4280)	461.7 (277.8 to 660.5)	-9.6 (-29.9 to 22.1)
		Male	17 (8 to 27)	6.8 (3.4 to 10.7)	-7.1 (-31.2 to 37.6)	2160 (1230 to 3100)	812.7 (479.4 to 1142.9)	-10.3 (-29.4 to 22.7)
		Female	7 (3 to 12)	2 (1 to 3.5)	-0.6 (-34.9 to 60.8)	769 (441 to 1240)	214.9 (125.5 to 344)	-3.4 (-33.8 to 44.6)
	Larynx cancer	Both	7 (4 to 10)	1.1 (0.7 to 1.6)	-11.3 (-33.2 to 21.2)	383 (275 to 534)	58.8 (42.8 to 81.4)	-19.1 (-35.6 to 5.1)
		Male	6 (4 to 9)	2.3 (1.4 to 3.3)	-7.8 (-32.6 to 28.8)	330 (239 to 462)	121.3 (90.3 to 164.3)	-16.2 (-33 to 7.4)
		Female	1 (1 to 2)	0.3 (0.2 to 0.5)	-17.5 (-40.5 to 19.2)	53 (30 to 84)	14.2 (8.4 to 22.3)	-26 (-48.8 to 12.1)
Ethiopia	TBL cancer	Both	510 (298 to 762)	1.3 (0.7 to 1.9)	4.4 (-17.9 to 34.9)	55000 (37900 to 74500)	130.5 (89.9 to 177.3)	2.8 (-17 to 27.5)
		Male	390 (231 to 591)	1.9 (1.1 to 2.9)	2.9 (-24.2 to 40.8)	42000 (28700 to 57500)	197.5 (134.5 to 270.8)	1.2 (-23.2 to 33.6)
		Female	120 (61 to 211)	0.6 (0.3 to 1)	23 (-0.1 to 55.6)	13000 (7930 to 21300)	59.1 (36.2 to 97.1)	21.9 (-0.8 to 53.8)
	Larynx cancer	Both	215 (143 to 306)	0.5 (0.3 to 0.7)	-7.7 (-23.5 to 12.6)	10700 (8420 to 14100)	23.1 (18.3 to 31)	-13.3 (-30.1 to 7.3)
		Male	175 (114 to 261)	0.8 (0.5 to 1.2)	-5.8 (-25.1 to 18.9)	8950 (6730 to 12400)	38.3 (28.8 to 53.5)	-11.3 (-31.3 to 13.6)
		Female	40 (27 to 56)	0.2 (0.1 to 0.2)	-6.2 (-22.2 to 12.9)	1710 (1330 to 2090)	7.2 (5.7 to 8.7)	-13.7 (-31.2 to 6.3)
Fiji	TBL cancer	Both	17 (11 to 24)	2.3 (1.5 to 3.2)	6.2 (-22.2 to 44.8)	1930 (1500 to 2460)	243.7 (190.8 to 305.2)	6.2 (-16.5 to 33.4)
		Male	11 (7 to 16)	3.2 (2 to 4.5)	4.7 (-26.6 to 48.9)	1250 (956 to 1600)	336.2 (262.8 to 425.7)	5.1 (-17.5 to 33.5)
		Female	6 (4 to 9)	1.6 (1 to 2.3)	10 (-26 to 55.4)	687 (525 to 894)	166 (127 to 213.8)	9.4 (-18.7 to 42.2)
		Both	3 (2 to 5)	0.5 (0.3 to 0.6)	2.8 (-19.3 to 29.4)	133 (100 to 171)	17.2 (13.3 to 21.9)	-1.8 (-23.4 to 25.7)

	Larynx cancer	Male	3 (2 to 4)	0.9 (0.6 to 1.2)	7.9 (-16.9 to 37.5)	110 (81 to 143)	32 (24.3 to 40.8)	3.8 (-19.1 to 34.4)
		Female	1 (0 to 1)	0.1 (0.1 to 0.2)	-14 (-33.4 to 12.8)	23 (17 to 31)	5.2 (3.9 to 7)	-19.7 (-40.6 to 9)
Finland	TBL cancer	Both	771 (513 to 1090)	6.4 (4.2 to 9)	-6.7 (-29.7 to 23.1)	48700 (44900 to 52000)	423.6 (391.7 to 452.7)	-10.9 (-16.9 to -4.6)
		Male	492 (320 to 695)	8.9 (5.8 to 12.6)	-13.5 (-36.5 to 18.2)	32200 (29700 to 34600)	596.8 (551.9 to 643.4)	-17.2 (-23.5 to -10.9)
		Female	279 (185 to 404)	4.3 (2.9 to 6.4)	2.9 (-26 to 39.3)	16500 (14800 to 18100)	275.6 (249.2 to 302.5)	0.1 (-8.9 to 10.1)
	Larynx cancer	Both	108 (71 to 156)	1 (0.7 to 1.5)	-5.3 (-28.7 to 25.4)	930 (832 to 1040)	8.9 (8 to 10)	-14.4 (-24.3 to -3.6)
		Male	89 (58 to 128)	1.8 (1.2 to 2.6)	-7.7 (-32 to 23.1)	820 (723 to 922)	16.6 (14.6 to 18.6)	-16.6 (-27 to -5.1)
		Female	19 (13 to 28)	0.4 (0.2 to 0.5)	-2.1 (-23.5 to 25.3)	109 (89 to 130)	2.1 (1.7 to 2.4)	-8.8 (-21.1 to 6.5)
France	TBL cancer	Both	11200 (7340 to 15700)	9.4 (6.2 to 13.4)	-7.5 (-30.9 to 20.8)	869000 (814000 to 921000)	771.2 (722.8 to 818.4)	-12 (-17.6 to -6.8)
		Male	7910 (5120 to 11400)	14.3 (9.3 to 20.5)	-12 (-35.2 to 17.5)	631000 (587000 to 674000)	1180.9 (1098.1 to 1260.1)	-15.8 (-22.2 to -9.7)
		Female	3260 (2130 to 4700)	5.2 (3.4 to 7.6)	3.2 (-26.3 to 43.4)	239000 (217000 to 261000)	410.4 (374.4 to 447.7)	-1.9 (-9.7 to 6.7)
	Larynx cancer	Both	3060 (1990 to 4480)	2.7 (1.8 to 4)	-3.1 (-31.6 to 36.1)	41200 (37000 to 45500)	37.3 (33.5 to 41.3)	-9.9 (-19.1 to 1.1)
		Male	2740 (1770 to 4050)	5.2 (3.3 to 7.6)	-4.6 (-34.2 to 34.4)	36300 (32500 to 40600)	69.3 (61.8 to 77.8)	-11.3 (-21.6 to 0.7)
		Female	319 (196 to 474)	0.6 (0.4 to 0.9)	4.9 (-19.7 to 38.1)	4920 (3480 to 5750)	8.6 (6.1 to 10.1)	-2 (-15.4 to 14.6)
Gabon	TBL cancer	Both	46 (27 to 73)	4.3 (2.5 to 6.6)	0.8 (-22.2 to 32.4)	5480 (3590 to 7970)	481.4 (320.4 to 687.9)	-1.7 (-20.7 to 23.2)
		Male	34 (19 to 55)	6.7 (3.9 to 10.6)	-3.3 (-27 to 33.7)	4070 (2670 to 6040)	748.1 (506.3 to 1092.9)	-5.2 (-22.8 to 19.5)
		Female	13 (7 to 20)	2.3 (1.3 to 3.5)	9.6 (-25.9 to 59.9)	1400 (863 to 2080)	239.1 (149.7 to 347.5)	7.7 (-18.7 to 41.6)
	Larynx cancer	Both	10 (7 to 15)	0.9 (0.6 to 1.3)	-2.6 (-23.4 to 24.5)	499 (360 to 673)	43.1 (31.6 to 58)	-9.5 (-28.8 to 16.7)
		Male	9 (6 to 13)	1.7 (1.1 to 2.5)	-2.4 (-23.8 to 26.7)	450 (320 to 616)	81.7 (59.3 to 110.9)	-8.5 (-27.4 to 17.2)
		Female	1 (1 to 2)	0.2 (0.1 to 0.3)	-10.2 (-31.9 to 17.5)	49 (33 to 70)	8.1 (5.6 to 11.5)	-17.8 (-40.4 to 10.8)
The Gambia	TBL cancer	Both	16 (10 to 24)	1.7 (1.1 to 2.5)	9.1 (-20 to 45.6)	1770 (1330 to 2310)	180.3 (135.8 to 234.3)	8.9 (-12.8 to 34.7)
		Male	11 (7 to 16)	2.4 (1.5 to 3.5)	4 (-28.6 to 46.9)	1160 (871 to 1490)	244.8 (187.4 to 312.4)	3 (-19.4 to 28.8)
		Female	6 (3 to 9)	1.1 (0.6 to 1.8)	22 (-15.2 to 69.2)	611 (379 to 862)	118.8 (73.6 to 169.2)	25.2 (-6.7 to 65.7)
	Larynx cancer	Both	3 (2 to 5)	0.3 (0.2 to 0.5)	6 (-17.5 to 33.3)	168 (128 to 217)	16.6 (12.7 to 20.8)	2.8 (-19.2 to 31.4)
		Male	3 (2 to 4)	0.6 (0.4 to 0.9)	6.2 (-17.4 to 36)	144 (109 to 186)	29.1 (22.3 to 36.7)	3.2 (-19.1 to 33.4)
		Female	0 (0 to 1)	0.1 (0.1 to 0.1)	9.2 (-18 to 44.2)	25 (17 to 34)	4.5 (3.2 to 6.2)	6.3 (-22.2 to 42.7)
Georgia	TBL cancer	Both	406 (276 to 552)	7.2 (4.8 to 9.8)	6 (-19 to 38.4)	45300 (37600 to 53700)	821.4 (679.1 to 973)	3.9 (-16 to 26.3)

		Male	343 (233 to 471)	14.1 (9.6 to 19.3)	6.4 (-20.9 to 40.2)	39000 (32300 to 46500)	1609.5 (1331.3 to 1909.8)	4.3 (-15.9 to 27.8)
		Female	63 (39 to 94)	1.9 (1.2 to 2.8)	2.5 (-30.9 to 46.3)	6320 (5210 to 7590)	205.3 (168.7 to 248.4)	-0.6 (-20.3 to 23.1)
	Larynx cancer	Both	128 (87 to 177)	2.3 (1.6 to 3.2)	1.8 (-23.1 to 32.9)	5270 (4260 to 6450)	96.4 (78.4 to 118.5)	-1.9 (-22.7 to 22.6)
		Male	119 (80 to 166)	4.9 (3.3 to 6.7)	3.2 (-23.5 to 36.9)	5010 (4030 to 6140)	207.3 (168 to 252.9)	-0.9 (-22.2 to 24.7)
		Female	9 (6 to 14)	0.3 (0.2 to 0.5)	-17.5 (-31.7 to 0.9)	262 (205 to 427)	8.7 (6.8 to 13.9)	-25.7 (-42.7 to -4.8)
Germany	TBL cancer	Both	15700 (10500 to 22100)	9.1 (6.1 to 12.9)	-9.1 (-32.5 to 20.8)	1100000 (1040000 to 1160000)	659.6 (624.2 to 695.3)	-10.4 (-15.1 to -5.6)
		Male	9920 (6540 to 14100)	12.1 (7.9 to 17.2)	-15.5 (-38.6 to 13.2)	716000 (671000 to 761000)	896.9 (841.1 to 952.9)	-16 (-21.1 to -10.3)
		Female	5750 (3730 to 8190)	6.5 (4.2 to 9.4)	0.9 (-26.2 to 37.3)	382000 (351000 to 412000)	449.4 (414.1 to 485)	-0.7 (-8.6 to 7.2)
	Larynx cancer	Both	2210 (1430 to 3210)	1.4 (0.9 to 2)	-6.2 (-28.2 to 21.6)	36000 (33000 to 39300)	22.4 (20.5 to 24.4)	-14.8 (-22.2 to -5.6)
		Male	1920 (1230 to 2780)	2.5 (1.6 to 3.6)	-8 (-29.9 to 20.8)	31100 (28300 to 34200)	40.1 (36.5 to 44.1)	-16.8 (-25.3 to -7)
		Female	296 (183 to 437)	0.4 (0.2 to 0.6)	-0.1 (-22.1 to 27.9)	4930 (3520 to 5610)	6 (4.3 to 6.9)	-5.5 (-16.9 to 8.1)
Ghana	TBL cancer	Both	327 (210 to 469)	2 (1.3 to 2.9)	6.5 (-18.2 to 41.8)	37600 (29800 to 47100)	213.6 (171.7 to 268.3)	6.1 (-12.7 to 30.9)
		Male	219 (135 to 324)	3.2 (2 to 4.6)	5.4 (-24.4 to 50.3)	25000 (19700 to 31600)	333 (266.7 to 415.7)	4.7 (-16.9 to 33.3)
		Female	108 (68 to 158)	1.1 (0.7 to 1.7)	16.9 (-14.9 to 56)	12600 (9440 to 16200)	120.9 (92 to 156.9)	17.7 (-7.4 to 46.8)
	Larynx cancer	Both	129 (87 to 185)	0.8 (0.5 to 1.1)	1.6 (-20 to 29.8)	6300 (4770 to 8170)	36.4 (28.2 to 46.6)	-3.4 (-22.6 to 22.4)
		Male	118 (78 to 170)	1.6 (1.1 to 2.3)	6.6 (-17.5 to 38.9)	5790 (4340 to 7550)	75.9 (58 to 97.5)	1.6 (-18.9 to 29.8)
		Female	12 (8 to 17)	0.1 (0.1 to 0.2)	-3.9 (-23.2 to 23.4)	513 (383 to 672)	5.3 (4 to 6.9)	-10.1 (-29.4 to 17.5)
Greece	TBL cancer	Both	2170 (1420 to 3070)	10.2 (6.7 to 14.6)	-3.3 (-28.6 to 25.4)	170000 (160000 to 179000)	846.7 (798 to 895.3)	-4.6 (-10.1 to 1.5)
		Male	1680 (1100 to 2380)	16.9 (10.9 to 24.1)	-5.8 (-30.7 to 22.1)	134000 (126000 to 142000)	1410.8 (1326.4 to 1498.8)	-7 (-13.1 to -0.7)
		Female	495 (312 to 706)	4.4 (2.8 to 6.4)	6.3 (-25.8 to 46.5)	36100 (32800 to 39200)	346.6 (316.6 to 374.9)	5.2 (-3.9 to 14.9)
	Larynx cancer	Both	526 (344 to 748)	2.6 (1.7 to 3.7)	-9 (-32.3 to 22.6)	7770 (7130 to 8400)	38.4 (35.3 to 41.6)	-9.5 (-17.3 to -1.7)
		Male	481 (311 to 690)	5 (3.2 to 7.2)	-9.6 (-34 to 22.9)	7090 (6490 to 7710)	74.7 (68.2 to 81.2)	-10 (-18.1 to -1.8)

		Female	45 (31 to 65)	0.5 (0.3 to 0.7)	-1.3 (-18.6 to 21.4)	677 (577 to 760)	6.2 (5.5 to 7)	-2.1 (-12.3 to 9.3)
Greenland	TBL cancer	Both	12 (8 to 16)	17 (11.6 to 22.7)	-12.8 (-27.8 to 4.3)	1290 (1040 to 1540)	1752 (1422.3 to 2080.2)	-14.7 (-28.9 to 0)
		Male	7 (5 to 10)	19.4 (13.1 to 26.3)	-13.3 (-29.8 to 6.1)	798 (623 to 967)	1997.6 (1563 to 2393.8)	-14.9 (-31.1 to 1.3)
		Female	5 (3 to 6)	14.3 (9.5 to 19.4)	-12.5 (-32.3 to 10.7)	496 (398 to 607)	1470.7 (1195.8 to 1801.1)	-14.7 (-30 to 4.3)
	Larynx cancer	Both	1 (1 to 1)	1.2 (0.8 to 1.6)	-6.6 (-26.7 to 17.8)	27 (21 to 33)	35.3 (27.3 to 43.4)	-16.5 (-32.9 to 2)
		Male	1 (1 to 1)	2 (1.3 to 2.8)	-6.2 (-26.9 to 20.2)	25 (19 to 31)	61.1 (47.3 to 75.8)	-15.8 (-32.8 to 4.1)
		Female	0 (0 to 0)	0.2 (0.1 to 0.3)	-14.9 (-32.5 to 8.2)	2 (1 to 2)	5 (3.9 to 6.3)	-24 (-41.5 to -3.5)
Grenada	TBL cancer	Both	4 (3 to 5)	3.3 (2.3 to 4.4)	-3.5 (-22.2 to 19.5)	394 (348 to 445)	335.6 (298 to 375.7)	-4.2 (-16.1 to 8.4)
		Male	3 (2 to 4)	4.7 (3.2 to 6.4)	-19 (-37.1 to 3.1)	273 (243 to 306)	475.7 (426 to 531.4)	-17.5 (-27.8 to -6.4)
		Female	1 (1 to 2)	2 (1.3 to 2.8)	6.8 (-25.2 to 51.8)	121 (105 to 141)	205.6 (177.6 to 237.9)	6 (-9.9 to 25.6)
	Larynx cancer	Both	1 (1 to 1)	0.8 (0.6 to 1.1)	-15.6 (-29.7 to 2.2)	35 (30 to 40)	29.5 (25.7 to 33.7)	-23.9 (-35.9 to -10.1)
		Male	1 (1 to 1)	1.5 (1.1 to 2)	-24.6 (-38.9 to -5.5)	31 (27 to 36)	53.6 (46.5 to 61.6)	-31.9 (-42.6 to -19.2)
		Female	0 (0 to 0)	0.2 (0.2 to 0.3)	-13.7 (-28.2 to 5.7)	4 (3 to 5)	6.9 (5.7 to 8.4)	-20.4 (-35.4 to 0)
Guam	TBL cancer	Both	13 (9 to 19)	7 (4.8 to 9.7)	4.5 (-17.9 to 31.7)	1450 (1200 to 1730)	753.6 (625 to 895.6)	4.8 (-14.1 to 25.9)
		Male	9 (6 to 12)	9.7 (6.5 to 13.4)	-0.4 (-24.1 to 28.8)	975 (807 to 1170)	1028.8 (853.6 to 1234.2)	-0.3 (-18.6 to 21.2)
		Female	4 (3 to 6)	4.6 (3.1 to 6.6)	14.1 (-20.1 to 56.5)	473 (381 to 586)	488.7 (393.6 to 604.7)	14.8 (-10.1 to 42.7)
	Larynx cancer	Both	1 (0 to 1)	0.3 (0.2 to 0.5)	-26 (-40.8 to -3.7)	17 (13 to 21)	8.7 (7.1 to 11.2)	-28.1 (-42.8 to -5.9)
		Male	0 (0 to 1)	0.5 (0.4 to 0.8)	-30.4 (-45.3 to -7.5)	13 (10 to 17)	14 (11.1 to 18.1)	-33 (-48 to -6.7)
		Female	0 (0 to 0)	0.1 (0.1 to 0.2)	-5.9 (-25.9 to 19.8)	4 (3 to 5)	4 (3.1 to 5)	-6.7 (-28.1 to 20.9)
Guatemala	TBL cancer	Both	202 (131 to 294)	1.8 (1.2 to 2.6)	-9.3 (-36 to 26.8)	21500 (16500 to 27200)	182.9 (141.2 to 231.3)	-10.3 (-33 to 18.1)
		Male	116 (74 to 175)	2.3 (1.5 to 3.4)	-9.7 (-40 to 37.4)	12400 (9460 to 15700)	234.5 (179.2 to 298.5)	-11.6 (-35.9 to 18.3)
		Female	86 (53 to 125)	1.4 (0.8 to 2)	-7.8 (-35.8 to 33.8)	9150 (7100 to 11600)	140.3 (108.7 to 177.2)	-7.2 (-29.8 to 24.5)
	Larynx cancer	Both	61 (41 to 85)	0.5 (0.3 to 0.7)	-12.1 (-30.4 to 11)	1920 (1500 to 2450)	16.5 (12.9 to 21)	-18.1 (-39.1 to 8.9)
		Male	35 (23 to 49)	0.7 (0.4 to 1)	-12.6 (-34.2 to 17.1)	1360 (1050 to 1730)	26 (20.1 to 33.1)	-17.3 (-39.5 to 10.5)
		Female	26 (17 to 38)	0.3 (0.2 to 0.5)	-8.3 (-23.9 to 11.2)	565 (434 to 749)	8.7 (6.7 to 11.5)	-16.5 (-37.2 to 11.4)
Guinea	TBL cancer	Both	129 (83 to 189)	2.3 (1.5 to 3.4)	2.5 (-25 to 38.4)	14800 (11100 to 19300)	253.9 (191.6 to 328.3)	0.9 (-18.6 to 24.3)
		Male	100 (62 to 151)	3.6 (2.2 to 5.4)	-1.2 (-31.4 to 40.3)	11600 (8680 to 15200)	395 (299.1 to 516.3)	-2.7 (-22.4 to 22.1)
		Female	28 (17 to 42)	1 (0.6 to 1.6)	11.2 (-22 to 58.5)	3180 (2210 to 4240)	109 (76.9 to 144.5)	11.6 (-10.5 to 40.5)

	Larynx cancer	Both	36 (22 to 53)	0.6 (0.4 to 0.9)	1.7 (-19.9 to 31.3)	1860 (1330 to 2470)	32.5 (23.2 to 43.2)	-1.5 (-22.2 to 24)
		Male	32 (19 to 47)	1.1 (0.7 to 1.7)	0.3 (-22.6 to 31.5)	1660 (1150 to 2240)	57.1 (39.8 to 76.9)	-2.5 (-23.9 to 24.3)
		Female	4 (3 to 6)	0.1 (0.1 to 0.2)	-1.6 (-22.8 to 23.7)	201 (142 to 270)	6.9 (4.9 to 9.1)	-4.2 (-25.7 to 21.3)
Guinea-Bissau	TBL cancer	Both	21 (12 to 35)	2.9 (1.7 to 4.7)	-1.8 (-26.4 to 32.8)	2530 (1630 to 3700)	322.4 (206.6 to 467.8)	-2.2 (-19.3 to 21.1)
		Male	16 (8 to 27)	4.8 (2.5 to 8.3)	-5.1 (-31.1 to 35.8)	1850 (1100 to 2910)	529 (317.5 to 815.5)	-5.4 (-21.3 to 17.6)
		Female	6 (4 to 9)	1.4 (0.9 to 2.1)	8.6 (-23.6 to 49)	680 (487 to 909)	151.3 (110.3 to 203.8)	8.9 (-13.7 to 37.7)
	Larynx cancer	Both	7 (4 to 10)	0.9 (0.5 to 1.2)	-5.3 (-26.7 to 22.4)	365 (253 to 479)	45.8 (31.9 to 59.2)	-8.9 (-27.8 to 15.5)
		Male	6 (4 to 9)	1.7 (1.1 to 2.5)	-5 (-26.9 to 24.9)	328 (218 to 435)	90.1 (61.7 to 116.9)	-8.3 (-27.3 to 17.5)
		Female	1 (0 to 1)	0.2 (0.1 to 0.3)	-7.1 (-27.5 to 19.6)	37 (27 to 49)	8.3 (6 to 10.9)	-11.5 (-31.9 to 16.3)
Guyana	TBL cancer	Both	13 (8 to 19)	2 (1.2 to 2.9)	4.5 (-23.8 to 40)	1490 (1140 to 1910)	219.5 (169.7 to 279.1)	4 (-21.2 to 35)
		Male	8 (5 to 12)	2.7 (1.6 to 4)	1.2 (-31.3 to 46.6)	930 (699 to 1210)	290.8 (221 to 373.1)	0.6 (-25.3 to 34.8)
		Female	5 (3 to 7)	1.4 (0.9 to 2.1)	12.3 (-23 to 62.4)	562 (427 to 721)	156.7 (120.5 to 199.9)	11.6 (-15.7 to 46.7)
	Larynx cancer	Both	5 (3 to 7)	0.7 (0.4 to 1)	1.4 (-25.2 to 34.4)	218 (162 to 287)	31.4 (23.5 to 40.9)	-3.4 (-29.1 to 30)
		Male	4 (3 to 6)	1.3 (0.8 to 1.8)	2.5 (-25.8 to 38.5)	197 (145 to 260)	59.3 (44.2 to 77.8)	-2.3 (-28.8 to 32.6)
		Female	1 (0 to 1)	0.2 (0.1 to 0.2)	2.4 (-21 to 33.6)	22 (16 to 29)	6.1 (4.6 to 8.1)	-2.5 (-28.9 to 32.6)
Haiti	TBL cancer	Both	183 (101 to 315)	2.6 (1.4 to 4.4)	0.7 (-23.4 to 31.5)	21400 (13100 to 34500)	281.8 (174.3 to 457.5)	-0.3 (-18.3 to 23.3)
		Male	127 (63 to 249)	3.8 (1.9 to 7.5)	0.3 (-26.7 to 40.1)	14700 (8090 to 27100)	418.2 (236.6 to 771.2)	-0.7 (-20.3 to 26)
		Female	56 (32 to 88)	1.5 (0.8 to 2.3)	4.4 (-26.8 to 44.9)	6640 (4320 to 9640)	160.2 (103.1 to 228.7)	3.3 (-18.7 to 32.4)
	Larynx cancer	Both	80 (44 to 121)	1.1 (0.6 to 1.7)	2.7 (-21.9 to 34.5)	4450 (2610 to 6500)	58.8 (34.8 to 85.2)	-1.5 (-21.3 to 24.5)
		Male	68 (36 to 103)	2 (1 to 3)	5.5 (-22.9 to 44.5)	3860 (2210 to 5640)	108.9 (63.4 to 157.5)	1.4 (-21.2 to 32.3)
		Female	12 (7 to 19)	0.3 (0.2 to 0.5)	-4.2 (-24.8 to 22)	589 (366 to 872)	14.2 (9 to 20.9)	-9.5 (-29.7 to 17.1)
Honduras	TBL cancer	Both	313 (178 to 492)	5.1 (2.9 to 8)	14.1 (-12.5 to 50.4)	34600 (22600 to 49900)	548.1 (359 to 784.6)	11.9 (-11.1 to 42.2)
		Male	127 (65 to 203)	4.5 (2.3 to 7.1)	13 (-23.2 to 62.5)	14000 (8790 to 19700)	475.7 (294.9 to 672.1)	10.8 (-16.4 to 50.3)
		Female	186 (96 to 312)	5.8 (3 to 9.5)	13.9 (-20.3 to 60.7)	20600 (12400 to 30700)	612.5 (366.8 to 907)	11.9 (-15.1 to 48.6)
	Larynx cancer	Both	47 (32 to 67)	0.7 (0.5 to 1)	12.5 (-8.2 to 41.6)	1810 (1370 to 2310)	29.2 (22.4 to 36.6)	6.7 (-14.8 to 36.5)
		Male	30 (19 to 43)	1 (0.7 to 1.5)	19.4 (-10.8 to 66.9)	1300 (957 to 1650)	44.3 (32.8 to 55.7)	12.2 (-16.2 to 52.4)

		Female	17 (11 to 25)	0.5 (0.3 to 0.7)	4.2 (-16.5 to 29.5)	518 (380 to 702)	15.7 (11.6 to 20.8)	-1.3 (-25 to 29.1)
Hungary	TBL cancer	Both	2170 (1500 to 2960)	12.1 (8.2 to 16.6)	-11.4 (-29.7 to 9.9)	210000 (172000 to 257000)	1208.9 (980 to 1477.7)	-16.2 (-31.8 to 2.9)
		Male	1330 (899 to 1810)	17.1 (11.5 to 23.6)	-17.3 (-34.7 to 4.5)	131000 (106000 to 161000)	1699.7 (1376.4 to 2082.2)	-21.6 (-36.9 to -3.1)
		Female	840 (566 to 1180)	8.3 (5.6 to 11.8)	-2.6 (-26.8 to 29.4)	79100 (64600 to 96800)	830.5 (672.7 to 1023.3)	-7.5 (-25.5 to 14.8)
	Larynx cancer	Both	493 (329 to 700)	3 (2 to 4.2)	-17.6 (-37.8 to 7.6)	12900 (10400 to 15900)	79.2 (63.4 to 98.2)	-27.2 (-41.9 to -9)
		Male	414 (271 to 592)	5.6 (3.7 to 7.9)	-20.3 (-41.4 to 7.1)	11200 (9010 to 13900)	151.5 (120 to 187.9)	-29.2 (-44 to -10.8)
		Female	79 (50 to 115)	0.9 (0.6 to 1.3)	-8.8 (-31.6 to 21.4)	1610 (1150 to 2010)	18.4 (12.7 to 23.2)	-19.8 (-38.9 to 0.5)
Iceland	TBL cancer	Both	48 (33 to 65)	9.1 (6.2 to 12.1)	-10.8 (-25.7 to 6.5)	3010 (2690 to 3330)	580 (521.1 to 643.7)	-13.7 (-22.2 to -4.1)
		Male	26 (18 to 36)	10 (6.8 to 13.5)	-11.6 (-29 to 7.4)	1610 (1430 to 1780)	627.4 (560.9 to 693.4)	-12.3 (-22.1 to -1.6)
		Female	22 (15 to 30)	8.2 (5.7 to 11.3)	-10.9 (-28.5 to 11.2)	1400 (1230 to 1570)	536.3 (478.5 to 601.2)	-15.8 (-25.3 to -5.2)
	Larynx cancer	Both	5 (3 to 7)	1 (0.7 to 1.4)	-1 (-19.2 to 20.6)	55 (48 to 63)	11 (9.6 to 12.6)	-5.8 (-18.8 to 9.4)
		Male	4 (3 to 6)	1.7 (1.1 to 2.3)	-1.9 (-22 to 22.3)	46 (39 to 53)	18.3 (15.8 to 21.3)	-6.7 (-20.4 to 10.1)
		Female	1 (1 to 1)	0.3 (0.2 to 0.5)	-0.8 (-18.7 to 20.3)	10 (8 to 12)	3.9 (3.1 to 4.6)	-5.3 (-20.7 to 14.6)
India	TBL cancer	Both	20400 (14200 to 27500)	1.8 (1.2 to 2.4)	10.4 (-9.3 to 30.7)	2250000 (1850000 to 2670000)	188.4 (155 to 222.8)	8.8 (-10.7 to 28.6)
		Male	14100 (9440 to 19700)	2.5 (1.7 to 3.5)	3.5 (-19.4 to 29)	1570000 (1220000 to 1940000)	268.8 (209.9 to 329.1)	1 (-21 to 25.9)
		Female	6290 (4080 to 8930)	1.1 (0.7 to 1.5)	32.2 (2 to 66.8)	683000 (529000 to 866000)	111.9 (86.8 to 141.6)	34.5 (2.2 to 71.1)
	Larynx cancer	Both	17200 (12200 to 23100)	1.4 (1 to 1.9)	1 (-15.5 to 21.1)	823000 (688000 to 990000)	67.1 (56.2 to 80.6)	-7 (-23.6 to 12.6)
		Male	14200 (9850 to 19300)	2.4 (1.7 to 3.3)	-0.3 (-19 to 22.7)	693000 (559000 to 851000)	114.9 (92.8 to 140.8)	-8.1 (-27.2 to 14.3)
		Female	3010 (2010 to 4230)	0.5 (0.3 to 0.7)	12.1 (-8 to 38.6)	130000 (102000 to 162000)	20.8 (16.4 to 26)	4.5 (-17.1 to 30.7)
Indonesia	TBL cancer	Both	10900 (6940 to 15200)	5 (3.2 to 7)	6.5 (-13.5 to 28.8)	1270000 (919000 to 1590000)	550.8 (399.7 to 679.2)	4.5 (-15.3 to 25.7)
		Male	7400 (4570 to 10900)	7.2 (4.6 to 10.4)	5 (-20.3 to 35.8)	865000 (625000 to 1160000)	788.8 (578.8 to 1038.6)	3.1 (-21.3 to 33.7)
		Female	3520 (1690 to 5940)	3.1 (1.5 to 5.2)	9.7 (-16.7 to 41.6)	404000 (224000 to 619000)	336.1 (187.8 to 511)	7.4 (-17.3 to 38.8)

	Larynx cancer	Both	1440 (940 to 2140)	0.6 (0.4 to 1)	2.4 (-15.7 to 22.8)	54100 (41200 to 72500)	23.6 (18.2 to 31.6)	-6.2 (-24.8 to 15.8)
		Male	1100 (673 to 1740)	1.1 (0.6 to 1.7)	4.9 (-17.6 to 32)	43900 (31500 to 63100)	40.1 (29.1 to 57.4)	-4 (-25.7 to 24.3)
		Female	340 (227 to 478)	0.3 (0.2 to 0.4)	-4.8 (-20.4 to 14)	10200 (7870 to 12900)	8.6 (6.7 to 10.6)	-14.4 (-34.1 to 10)
Iran	TBL cancer	Both	2020 (1450 to 2620)	2.8 (2 to 3.6)	14.5 (5.6 to 23.8)	217000 (202000 to 232000)	284 (263.3 to 304.5)	12.1 (4.8 to 20.3)
		Male	1340 (957 to 1760)	3.7 (2.7 to 4.8)	8 (-2.2 to 19.2)	149000 (136000 to 163000)	393.2 (360.8 to 430.7)	7.4 (-0.8 to 17.1)
		Female	679 (482 to 882)	1.9 (1.3 to 2.4)	32.6 (16.6 to 48.1)	68000 (61900 to 73300)	176.3 (159.6 to 190.5)	26 (13.1 to 37.4)
	Larynx cancer	Both	1250 (892 to 1680)	1.7 (1.2 to 2.2)	17.3 (9.3 to 30.8)	35800 (32700 to 39800)	46.4 (42.8 to 51.6)	-2.8 (-8.4 to 8.3)
		Male	940 (668 to 1260)	2.5 (1.8 to 3.3)	11.3 (2.3 to 25.5)	27900 (25000 to 31500)	72.6 (65.5 to 81.9)	-7.5 (-13.8 to 4.3)
		Female	314 (218 to 421)	0.8 (0.6 to 1.1)	43.5 (31.1 to 58.2)	7920 (7360 to 8610)	20.5 (19 to 22.3)	21.6 (13.7 to 33.1)
Iraq	TBL cancer	Both	957 (630 to 1390)	4.2 (2.7 to 5.9)	20.1 (-8.9 to 57.1)	110000 (83400 to 139000)	447.4 (343.5 to 552.8)	16.3 (-4.5 to 41.5)
		Male	681 (429 to 986)	6.2 (4 to 8.9)	15.6 (-14.4 to 57.4)	78800 (60000 to 99600)	669.4 (511.7 to 817.7)	12.4 (-9.2 to 41.6)
		Female	277 (172 to 413)	2.3 (1.4 to 3.4)	36.1 (-7.3 to 92.8)	31000 (23100 to 40600)	237.3 (179.2 to 305.5)	30.8 (1.9 to 70.2)
	Larynx cancer	Both	398 (260 to 568)	1.6 (1.1 to 2.3)	8.4 (-16.2 to 40.3)	14400 (10700 to 18500)	56.8 (43 to 71.2)	-7.7 (-26.1 to 14.3)
		Male	289 (184 to 418)	2.5 (1.6 to 3.5)	10.2 (-18.2 to 49.1)	10600 (7970 to 13500)	88.1 (67.1 to 109.8)	-6 (-26.8 to 19)
		Female	109 (71 to 163)	0.8 (0.5 to 1.2)	5.4 (-20.8 to 43.9)	3760 (2700 to 5100)	27 (19.8 to 35.7)	-10.7 (-32.1 to 20.2)
Ireland	TBL cancer	Both	604 (403 to 849)	8.1 (5.4 to 11.4)	-6.3 (-29.4 to 21.3)	43000 (39800 to 46100)	585.5 (540.4 to 627.6)	-13.5 (-19.8 to -7.2)
		Male	327 (212 to 464)	9.2 (6 to 13.1)	-13.9 (-37.3 to 15.7)	24000 (21800 to 25900)	677.1 (615.4 to 732.4)	-19.9 (-27.1 to -12.9)
		Female	277 (181 to 389)	7.2 (4.6 to 10.1)	3 (-23.5 to 41.6)	19000 (17000 to 21200)	502.9 (450.4 to 562)	-4.8 (-13.4 to 4.9)
	Larynx cancer	Both	113 (76 to 161)	1.6 (1.1 to 2.3)	-7.9 (-31.2 to 22.7)	1340 (1190 to 1500)	18.7 (16.7 to 21)	-20.4 (-29 to -11.3)
		Male	98 (64 to 142)	2.8 (1.9 to 4.1)	-8.6 (-33.1 to 24.9)	1140 (1000 to 1290)	32.9 (28.7 to 37.1)	-21 (-30.6 to -10.7)
		Female	16 (10 to 23)	0.4 (0.3 to 0.7)	-5.8 (-26.7 to 19.5)	199 (166 to 243)	5.4 (4.6 to 6.6)	-17.8 (-30.5 to -2.9)
Israel	TBL cancer	Both	634 (414 to 902)	5.6 (3.7 to 8)	-5.1 (-29.5 to 23.8)	52000 (48600 to 55300)	472.5 (442.2 to 501)	-9.9 (-15.5 to -4.2)

		Male	417 (267 to 601)	8 (5.1 to 11.5)	-8.2 (-33.6 to 23.1)	35400 (33000 to 37900)	686.5 (639.6 to 735.1)	-12.4 (-18 to -5.8)
		Female	217 (142 to 310)	3.5 (2.3 to 5.1)	-0.6 (-29.9 to 35.9)	16700 (15000 to 18100)	281.8 (255.6 to 306.3)	-6.2 (-14.2 to 2.4)
	Larynx cancer	Both	125 (81 to 179)	1.1 (0.7 to 1.6)	-5.5 (-29 to 22.6)	1950 (1750 to 2150)	17.8 (15.9 to 19.6)	-14.8 (-23.8 to -5.3)
		Male	110 (71 to 158)	2.1 (1.4 to 3.1)	-8 (-32 to 21.3)	1700 (1510 to 1900)	33.1 (29.3 to 37)	-17.5 (-27.4 to -7.4)
		Female	15 (10 to 22)	0.3 (0.2 to 0.4)	5.9 (-15.9 to 32.1)	251 (215 to 296)	4.3 (3.7 to 5)	-0.7 (-13 to 14.6)
Italy	TBL cancer	Both	10100 (7040 to 13500)	7.6 (5.2 to 10.2)	-10.6 (-27.1 to 7.6)	689000 (646000 to 718000)	544.4 (516.5 to 565.5)	-12.8 (-15.3 to -10.5)
		Male	6880 (4660 to 9760)	11.2 (7.6 to 15.9)	-16.7 (-36.3 to 7.9)	493000 (465000 to 517000)	830.9 (788 to 869)	-18.1 (-20.8 to -15.5)
		Female	3190 (2150 to 4420)	4.6 (3 to 6.4)	1.1 (-21.7 to 31.5)	196000 (180000 to 207000)	300.4 (282 to 315.1)	-0.9 (-4.3 to 3.1)
	Larynx cancer	Both	2510 (1670 to 3460)	2.1 (1.4 to 2.9)	-10.8 (-28.3 to 11.4)	33200 (31200 to 35100)	27 (25.5 to 28.4)	-16.9 (-20.5 to -12.6)
		Male	2150 (1410 to 2990)	3.8 (2.5 to 5.3)	-12.9 (-32.7 to 10.5)	30000 (28200 to 31800)	52 (49.2 to 55)	-18.7 (-22.4 to -14.3)
		Female	360 (234 to 515)	0.6 (0.4 to 0.8)	-4 (-22 to 18.4)	3260 (2830 to 3720)	5.1 (4.5 to 5.7)	-11.2 (-17.2 to -2.5)
Jamaica	TBL cancer	Both	132 (85 to 191)	4.5 (2.9 to 6.5)	-4.4 (-32 to 28.8)	13800 (10700 to 17400)	467.4 (361.3 to 586.8)	-2.8 (-24.7 to 23.7)
		Male	103 (65 to 148)	7.1 (4.5 to 10.2)	-8.3 (-36.1 to 26.7)	10800 (8300 to 13700)	743.5 (570.6 to 939.2)	-6.7 (-27.9 to 19.2)
		Female	30 (18 to 45)	1.9 (1.2 to 2.9)	10.7 (-26.7 to 62.9)	3040 (2300 to 3940)	199.9 (151 to 260.1)	13 (-15.7 to 49.2)
	Larynx cancer	Both	25 (16 to 36)	0.8 (0.5 to 1.2)	-6.9 (-33.2 to 24.7)	888 (678 to 1150)	30.1 (23 to 39)	-6.9 (-29.4 to 21.2)
		Male	23 (14 to 34)	1.6 (1 to 2.3)	-8.5 (-34.9 to 23.7)	826 (629 to 1080)	57.4 (43.5 to 74.6)	-8.4 (-31 to 20)
		Female	2 (1 to 3)	0.1 (0.1 to 0.2)	12.1 (-10.4 to 41.5)	62 (48 to 83)	4.1 (3.1 to 5.4)	14.4 (-12.5 to 49.4)
Japan	TBL cancer	Both	32100 (22500 to 42600)	8.9 (6.3 to 11.8)	-11 (-24.1 to 4.3)	1320000 (1180000 to 1390000)	399.1 (370.5 to 417.5)	-14.3 (-17.1 to -11.9)
		Male	22700 (15700 to 30700)	14.1 (9.8 to 19)	-14.8 (-30.6 to 4.7)	943000 (871000 to 993000)	624.5 (583.7 to 653.7)	-18.1 (-21.4 to -15.4)
		Female	9400 (6340 to 13100)	4.7 (3.2 to 6.5)	-4.1 (-21.3 to 16)	372000 (307000 to 409000)	205.9 (183.2 to 221.5)	-6.9 (-10.7 to -3)
	Larynx cancer	Both	3080 (2100 to 4230)	1 (0.7 to 1.4)	-10.9 (-24.7 to 5.9)	19400 (17700 to 20700)	6.2 (5.8 to 6.6)	-17 (-20.3 to -12.2)
		Male	2670 (1800 to 3700)	1.8 (1.3 to 2.6)	-13.2 (-29.1 to 5.9)	17200 (15800 to 18200)	11.7 (10.9 to 12.3)	-19.1 (-22.8 to -14.1)
		Female	410 (275 to 611)	0.3 (0.2 to 0.4)	-4.5 (-18.9 to 12.9)	2280 (1930 to 2620)	1.5 (1.4 to 1.7)	-12.1 (-16.2 to -4.9)

Jordan	TBL cancer	Both	216 (142 to 306)	3.3 (2.2 to 4.6)	-3.7 (-28.7 to 28.7)	24000 (19600 to 29100)	334.8 (274 to 405.7)	-6.2 (-24.8 to 14.3)
		Male	163 (102 to 235)	4.7 (2.9 to 6.8)	-8.5 (-37.6 to 27.6)	18500 (14200 to 23500)	493.1 (382.4 to 622.3)	-10.6 (-31.9 to 14.2)
		Female	54 (35 to 78)	1.7 (1.1 to 2.5)	12.4 (-18.6 to 58.6)	5530 (4310 to 7060)	160.2 (126.9 to 203.8)	8 (-15.4 to 36.7)
	Larynx cancer	Both	45 (30 to 66)	0.6 (0.4 to 0.9)	-7.8 (-28.6 to 18.6)	1190 (908 to 1560)	16.4 (12.6 to 21.6)	-21.8 (-41.1 to 1.6)
		Male	39 (25 to 57)	1 (0.7 to 1.6)	-10.9 (-33.1 to 18)	1040 (763 to 1400)	27.4 (20.4 to 37)	-24.5 (-44.7 to 0.7)
		Female	7 (4 to 10)	0.2 (0.1 to 0.3)	8.1 (-12.9 to 32.3)	145 (111 to 190)	4.2 (3.2 to 5.4)	-7.5 (-28.7 to 18.1)
Kazakhstan	TBL cancer	Both	887 (608 to 1200)	4.9 (3.3 to 6.6)	-16.6 (-33.4 to 4.1)	98200 (83200 to 113000)	525.1 (448.1 to 603.6)	-20.6 (-32.7 to -7.5)
		Male	702 (475 to 986)	9.4 (6.4 to 13)	-20.1 (-38.2 to 1.1)	78800 (66000 to 92400)	1001.8 (849.9 to 1168.3)	-24.1 (-36.5 to -11.1)
		Female	184 (120 to 258)	1.8 (1.2 to 2.5)	-3.7 (-34.9 to 40.8)	19400 (16100 to 22900)	185.9 (155 to 218.7)	-7.3 (-24.1 to 10.3)
	Larynx cancer	Both	206 (140 to 284)	1.1 (0.7 to 1.5)	-10.4 (-30.5 to 13.6)	7400 (6240 to 8660)	38.8 (32.8 to 45.4)	-25.4 (-37.7 to -12.6)
		Male	179 (120 to 248)	2.3 (1.5 to 3.1)	-12.7 (-34.6 to 14.3)	6650 (5580 to 7800)	82.4 (69.5 to 96.7)	-26.7 (-39.1 to -13.7)
		Female	27 (19 to 40)	0.3 (0.2 to 0.4)	-3.3 (-17.2 to 16.4)	748 (613 to 992)	7.1 (5.8 to 9.5)	-22 (-35.8 to -2.2)
Kenya	TBL cancer	Both	281 (190 to 392)	1.3 (0.9 to 1.8)	0.9 (-17 to 18.4)	33400 (27400 to 41100)	142.2 (117.7 to 172.6)	-1.3 (-16.1 to 15.3)
		Male	209 (140 to 290)	2.1 (1.4 to 2.9)	-5 (-24.9 to 16.8)	24500 (19500 to 30100)	226.3 (182.3 to 273.6)	-6.4 (-24.2 to 15.7)
		Female	72 (42 to 118)	0.6 (0.4 to 1)	17.6 (-8.4 to 58.3)	8860 (5950 to 13400)	68.9 (46.7 to 103.7)	13.2 (-11.7 to 48.3)
	Larynx cancer	Both	181 (124 to 259)	0.7 (0.5 to 1)	-6.7 (-23.8 to 11.5)	9770 (7510 to 13200)	37.3 (28.8 to 51.1)	-12.6 (-28.2 to 4.4)
		Male	154 (103 to 226)	1.3 (0.9 to 1.9)	-8.5 (-27 to 11.8)	8370 (6210 to 11800)	66.5 (49.7 to 94)	-13.9 (-31.1 to 6.4)
		Female	28 (18 to 39)	0.2 (0.1 to 0.3)	1.1 (-18.5 to 28.6)	1400 (1040 to 1840)	10.1 (7.6 to 13.2)	-6.9 (-27.1 to 17.2)
Kiribati	TBL cancer	Both	3 (2 to 5)	4.3 (2.8 to 6.3)	0.6 (-20 to 28.3)	410 (298 to 547)	509.7 (378.6 to 665.9)	0.1 (-16.6 to 18.6)
		Male	3 (2 to 4)	8 (5 to 12)	0.1 (-23 to 35.2)	339 (241 to 457)	954 (690.5 to 1254.5)	0.1 (-18.1 to 21.8)
		Female	1 (0 to 1)	1.5 (0.9 to 2.4)	6.1 (-23.6 to 46.8)	71 (50 to 95)	169.6 (122.9 to 225)	4 (-15.8 to 28.2)
	Larynx cancer	Both	0 (0 to 0)	0.4 (0.3 to 0.5)	-1.9 (-16.4 to 15.6)	13 (10 to 17)	17.4 (13.6 to 22)	-4.1 (-19.4 to 14)
		Male	0 (0 to 0)	0.6 (0.4 to 0.9)	2 (-17.7 to 25.5)	9 (6 to 12)	29 (22.1 to 37.1)	0 (-18.6 to 21.7)
		Female	0 (0 to 0)	0.2 (0.1 to 0.3)	-7.3 (-25.4 to 15.9)	4 (3 to 6)	9.5 (7.1 to 12.5)	-9.8 (-29.3 to 16.5)
Kuwait	TBL cancer	Both	54 (36 to 75)	2.3 (1.5 to 3.2)	-0.2 (-25.5 to 30.8)	5490 (4500 to 6620)	212.5 (173 to 258.2)	-3 (-20.7 to 17.6)

		Male	40 (26 to 58)	2.9 (1.9 to 4.3)	5 (-25.6 to 47.4)	4220 (3250 to 5290)	284.7 (220.3 to 354.9)	3.2 (-19.4 to 30.2)
		Female	14 (9 to 20)	1.2 (0.8 to 1.9)	-11.5 (-38.9 to 25.6)	1270 (980 to 1600)	103 (81.2 to 128.8)	-17.3 (-34 to 3)
	Larynx cancer	Both	19 (13 to 27)	0.7 (0.5 to 1)	5.1 (-17.6 to 31.5)	350 (274 to 431)	13.4 (10.6 to 16.5)	-12 (-30.5 to 10.4)
		Male	16 (11 to 23)	1 (0.7 to 1.5)	12.4 (-14.8 to 43.5)	301 (230 to 382)	19.8 (15.3 to 24.9)	-5.5 (-27.6 to 21.4)
		Female	3 (2 to 5)	0.3 (0.2 to 0.4)	-16.8 (-36.2 to 10.3)	49 (36 to 71)	3.8 (2.8 to 5.5)	-34.8 (-50.8 to -11.6)
Kyrgyzstan	TBL cancer	Both	136 (92 to 187)	2.8 (1.9 to 3.9)	-6.6 (-28.7 to 20.3)	15200 (13200 to 17400)	305.3 (264.4 to 347.7)	-9.8 (-22 to 2.9)
		Male	102 (67 to 144)	4.9 (3.2 to 6.9)	-8.7 (-33.1 to 22.9)	11600 (9950 to 13300)	527.4 (455.8 to 603.6)	-12.2 (-24.5 to 0.6)
		Female	34 (22 to 47)	1.3 (0.8 to 1.8)	2.1 (-27.2 to 41.1)	3620 (2980 to 4290)	132.2 (109.5 to 156.4)	0.6 (-16.8 to 18.8)
	Larynx cancer	Both	29 (20 to 39)	0.6 (0.4 to 0.8)	-10.1 (-25.4 to 10.3)	1120 (940 to 1330)	21.6 (18.3 to 25.7)	-19 (-32.6 to -2.8)
		Male	24 (17 to 33)	1.1 (0.8 to 1.5)	-12.1 (-28.6 to 9.2)	971 (810 to 1170)	42.7 (35.8 to 51.2)	-20.5 (-34.4 to -3.5)
		Female	4 (3 to 6)	0.2 (0.1 to 0.2)	3.6 (-13.8 to 25.3)	144 (116 to 179)	5.1 (4.1 to 6.3)	-6 (-25.7 to 17.5)
Laos	TBL cancer	Both	220 (136 to 319)	5 (3.1 to 7.2)	2.2 (-23.2 to 31.4)	26300 (19000 to 34800)	561.5 (413.5 to 730.4)	1.2 (-18.4 to 24.3)
		Male	160 (96 to 237)	7.7 (4.7 to 11.2)	-1.5 (-27.9 to 29.6)	19100 (13600 to 25100)	853.6 (618.8 to 1102.9)	-2.5 (-22.1 to 21.2)
		Female	60 (36 to 95)	2.6 (1.6 to 4)	12 (-21.6 to 57.5)	7240 (5110 to 10400)	290.3 (206.9 to 412.9)	11.4 (-11.5 to 37.2)
	Larynx cancer	Both	30 (19 to 45)	0.7 (0.4 to 1)	-0.1 (-19.5 to 23.1)	1340 (940 to 1830)	28.5 (20.2 to 38.9)	-8.1 (-25 to 15.4)
		Male	23 (14 to 36)	1.1 (0.7 to 1.7)	1.3 (-20.3 to 29.8)	1070 (725 to 1530)	47.4 (33 to 67.1)	-6.8 (-25.4 to 19.6)
		Female	7 (4 to 9)	0.3 (0.2 to 0.4)	-6.4 (-24 to 16.3)	271 (197 to 357)	10.9 (8 to 14.1)	-14.4 (-32.9 to 11.7)
Latvia	TBL cancer	Both	236 (162 to 332)	6.4 (4.4 to 9)	-12.2 (-32.4 to 13.6)	21000 (17200 to 25700)	595.6 (483.7 to 733.1)	-16.7 (-32.9 to 3)
		Male	179 (119 to 257)	12.4 (8.2 to 17.8)	-18.8 (-40.6 to 9)	16400 (12700 to 21000)	1144.5 (890.1 to 1467.3)	-22.6 (-40.9 to -0.1)
		Female	57 (33 to 86)	2.4 (1.4 to 3.7)	4.3 (-30.5 to 52.7)	4640 (3430 to 6190)	218.5 (158.8 to 294.2)	-0.3 (-27.3 to 34.6)
	Larynx cancer	Both	56 (37 to 84)	1.7 (1.1 to 2.5)	-10.7 (-34.6 to 21.3)	1720 (1310 to 2210)	52.4 (39.8 to 67.8)	-22.6 (-41.1 to 0.6)
		Male	53 (35 to 79)	3.8 (2.5 to 5.6)	-13.8 (-37.5 to 19.6)	1640 (1230 to 2120)	117.4 (88.7 to 152.9)	-25.1 (-43.5 to -2.5)
		Female	4 (2 to 5)	0.2 (0.1 to 0.3)	-8.6 (-32.1 to 23)	84 (61 to 112)	4.2 (3 to 5.7)	-21 (-42.4 to 7.8)
Lebanon	TBL cancer	Both	320 (214 to 461)	6.1 (4.1 to 8.9)	-0.4 (-20.1 to 23.5)	32400 (26100 to 42200)	623.4 (501.8 to 809.2)	-3.7 (-18.7 to 13.1)
		Male	200 (132 to 296)	8.5 (5.6 to 12.7)	-4.8 (-25.2 to 23.4)	21600 (16900 to 28200)	917.5 (717 to 1204.8)	-6.1 (-20.8 to 11.2)

		Female	120 (76 to 179)	4.2 (2.7 to 6.3)	16 (-20.7 to 58.8)	10800 (7940 to 14600)	380.8 (278.3 to 514.1)	9.6 (-16.5 to 34.4)
	Larynx cancer	Both	158 (109 to 223)	3 (2.1 to 4.3)	9.4 (-14.5 to 39.5)	3530 (2850 to 4510)	67.9 (54.8 to 86.7)	-8.5 (-25.3 to 12.8)
		Male	131 (90 to 189)	5.6 (3.8 to 8)	13.7 (-14.1 to 49)	3020 (2420 to 3910)	128.6 (102.7 to 168.1)	-4.7 (-23.6 to 18.6)
		Female	27 (17 to 40)	0.9 (0.6 to 1.4)	10.7 (-17.7 to 47)	503 (378 to 685)	17.7 (13.3 to 24.3)	-8.2 (-30.1 to 20.1)
Lesotho	TBL cancer	Both	53 (30 to 81)	4 (2.3 to 6.1)	4.2 (-23.7 to 40.9)	6680 (4460 to 9510)	483.4 (324.2 to 680.9)	2.1 (-19.4 to 26.1)
		Male	39 (23 to 61)	7.2 (4.2 to 11.3)	1.6 (-25.3 to 41.4)	5160 (3440 to 7460)	880.4 (602.2 to 1262.5)	-0.4 (-18.3 to 21)
		Female	13 (7 to 23)	1.8 (0.9 to 3)	13.4 (-29.2 to 70.5)	1530 (874 to 2430)	197.6 (115.1 to 314.6)	12.5 (-23.3 to 54.7)
	Larynx cancer	Both	18 (12 to 26)	1.3 (0.8 to 1.9)	-0.7 (-26.9 to 32.6)	1080 (806 to 1440)	76.1 (57.2 to 100.5)	-5.8 (-25.6 to 19.9)
		Male	15 (9 to 22)	2.6 (1.6 to 3.8)	0.4 (-28.2 to 37.6)	919 (686 to 1210)	152 (115 to 198.3)	-4.8 (-24 to 20.9)
		Female	3 (2 to 5)	0.4 (0.2 to 0.6)	-2.7 (-32 to 32.1)	163 (96 to 241)	20.5 (12.2 to 30)	-7.4 (-37.6 to 29.1)
Liberia	TBL cancer	Both	41 (24 to 62)	2.1 (1.2 to 3.2)	-0.3 (-25.9 to 32.5)	4650 (3110 to 6450)	216.7 (146.9 to 299.7)	-0.4 (-21.2 to 25.1)
		Male	29 (16 to 46)	2.9 (1.6 to 4.7)	-4 (-30.9 to 33)	3280 (2090 to 4790)	305.5 (199.2 to 442)	-4.4 (-24 to 20.6)
		Female	12 (7 to 18)	1.2 (0.7 to 1.8)	10.7 (-20.7 to 53.2)	1370 (878 to 1830)	123.6 (80.3 to 163.4)	10.9 (-13.7 to 39.8)
	Larynx cancer	Both	13 (8 to 18)	0.6 (0.4 to 0.9)	-5.5 (-25.9 to 22.4)	623 (445 to 861)	28.7 (20.8 to 38.7)	-7.3 (-27 to 20)
		Male	11 (7 to 16)	1.1 (0.7 to 1.5)	-5.2 (-26.4 to 24.4)	554 (388 to 776)	49.7 (35.4 to 68)	-7.1 (-27.8 to 21.5)
		Female	1 (1 to 2)	0.1 (0.1 to 0.2)	-9.8 (-30 to 16.1)	69 (48 to 94)	6.1 (4.4 to 8.4)	-12.1 (-32.9 to 16)
Libya	TBL cancer	Both	212 (138 to 306)	4.1 (2.7 to 5.9)	-5.5 (-32.6 to 28.6)	24800 (18400 to 31700)	453.7 (338.4 to 579)	-5.3 (-28.1 to 20.6)
		Male	181 (116 to 262)	6.9 (4.4 to 10)	-5 (-33.8 to 33.3)	21400 (15700 to 27500)	771.9 (565 to 992.6)	-4.9 (-28.1 to 23.2)
		Female	31 (18 to 48)	1.2 (0.7 to 1.9)	-3 (-37.3 to 38.4)	3360 (2110 to 4670)	122.5 (77.9 to 168.2)	-2 (-29.4 to 27.1)
	Larynx cancer	Both	107 (68 to 156)	1.9 (1.3 to 2.8)	3.1 (-23.8 to 38.2)	3940 (2940 to 5190)	68.3 (51.4 to 89.1)	4.9 (-20.7 to 37.3)
		Male	91 (58 to 135)	3.3 (2.1 to 4.8)	4.4 (-24.8 to 43.5)	3440 (2550 to 4610)	117.3 (87.2 to 155.8)	6.1 (-20.6 to 41.6)
		Female	15 (10 to 22)	0.6 (0.3 to 0.8)	-0.8 (-25.9 to 34.3)	502 (366 to 681)	17.1 (12.6 to 23)	0.2 (-24.1 to 31.6)
Lithuania	TBL cancer	Both	320 (219 to 443)	5.9 (4 to 8.3)	-13.9 (-34.6 to 11.2)	29200 (23600 to 35600)	569.8 (458.1 to 698.1)	-17 (-33.3 to 2.1)
		Male	246 (166 to 347)	11.6 (7.8 to 16.4)	-19.5 (-39.8 to 7)	23300 (18500 to 28700)	1111.9 (883.8 to 1367.8)	-22 (-37.6 to -4.1)
		Female	74 (47 to 108)	2.2 (1.4 to 3.2)	6.6 (-23.8 to 49.6)	5920 (4740 to 7290)	194.9 (154.4 to 240.9)	2.9 (-18.7 to 28.8)

	Larynx cancer	Both	85 (57 to 125)	1.7 (1.2 to 2.5)	-16.9 (-38.8 to 11)	2950 (2350 to 3680)	61.6 (49 to 77.8)	-24.8 (-41.2 to -5.3)
		Male	79 (52 to 115)	3.8 (2.5 to 5.6)	-18.8 (-40.7 to 9.7)	2790 (2210 to 3510)	136.7 (107.7 to 172.2)	-26.3 (-42.3 to -7.3)
		Female	6 (4 to 9)	0.2 (0.1 to 0.3)	-1.8 (-23.6 to 25.4)	152 (116 to 192)	5.5 (4.2 to 7.1)	-10.7 (-31.6 to 14.8)
Luxembourg	TBL cancer	Both	78 (52 to 107)	8.1 (5.4 to 11.1)	-16.1 (-34.5 to 6.2)	6140 (5400 to 6960)	647.1 (569.2 to 733.3)	-17.3 (-27.3 to -5.7)
		Male	53 (35 to 73)	11.4 (7.6 to 15.8)	-20.9 (-39.6 to 2.7)	4200 (3660 to 4800)	910.9 (793 to 1040.7)	-22.6 (-33.6 to -10.8)
		Female	25 (17 to 36)	5.1 (3.3 to 7.2)	-10.6 (-35.6 to 20.8)	1930 (1630 to 2290)	402.9 (338.6 to 475.8)	-8.9 (-22.7 to 7.7)
	Larynx cancer	Both	17 (12 to 24)	1.8 (1.2 to 2.6)	-17.3 (-36.2 to 9)	219 (189 to 254)	23.4 (20.2 to 27.2)	-22.2 (-32.9 to -9.2)
		Male	15 (10 to 21)	3.2 (2.1 to 4.6)	-19.7 (-39.1 to 7.7)	187 (161 to 219)	40.4 (34.8 to 47.3)	-24.8 (-35.7 to -11.4)
		Female	2 (1 to 3)	0.5 (0.3 to 0.7)	-14.6 (-32.9 to 10.1)	32 (27 to 40)	6.8 (5.6 to 8.4)	-17.2 (-30.1 to 1.7)
Madagascar	TBL cancer	Both	165 (104 to 247)	1.5 (0.9 to 2.3)	0.4 (-25.1 to 34.9)	19400 (13900 to 25800)	159.6 (117.6 to 210.8)	0.1 (-20.9 to 25.9)
		Male	109 (65 to 170)	2.1 (1.3 to 3.3)	-5.2 (-35.2 to 36.6)	12900 (9060 to 17700)	226.1 (162.2 to 305.5)	-5.2 (-26.3 to 19.7)
		Female	55 (34 to 83)	0.9 (0.6 to 1.4)	13.7 (-12.7 to 45.5)	6500 (4530 to 8900)	97.8 (68.4 to 132.8)	12.9 (-13.3 to 45.3)
	Larynx cancer	Both	64 (41 to 95)	0.5 (0.3 to 0.7)	-5.7 (-25.6 to 20.3)	3570 (2560 to 4810)	26.4 (19.3 to 35.3)	-8 (-29.2 to 18)
		Male	46 (29 to 70)	0.8 (0.5 to 1.1)	-6.3 (-28.8 to 22.7)	2590 (1800 to 3600)	39.9 (28.3 to 54.9)	-8.4 (-31.1 to 19.2)
		Female	18 (11 to 27)	0.3 (0.2 to 0.4)	-4.6 (-26.6 to 23.3)	978 (706 to 1360)	13.5 (9.9 to 18.5)	-7.3 (-31.2 to 22.3)
Malawi	TBL cancer	Both	99 (64 to 144)	1.4 (0.9 to 2)	-2.3 (-25.5 to 25.7)	11000 (8450 to 14100)	143.9 (112.1 to 180.5)	-3.9 (-22.8 to 16.4)
		Male	69 (41 to 104)	2.2 (1.4 to 3.3)	-7.7 (-34.8 to 29.3)	7760 (5750 to 10200)	231.1 (175.3 to 298)	-8.9 (-28 to 13.7)
		Female	31 (20 to 45)	0.8 (0.5 to 1.1)	12.6 (-13.1 to 41.4)	3270 (2360 to 4240)	75.9 (55.4 to 96.8)	11.9 (-15.1 to 42.3)
	Larynx cancer	Both	22 (15 to 31)	0.3 (0.2 to 0.4)	-6.9 (-25.3 to 15.7)	1140 (865 to 1490)	13.8 (10.7 to 17.9)	-10 (-29 to 13.3)
		Male	16 (11 to 24)	0.5 (0.3 to 0.7)	-7.4 (-26.5 to 17.3)	862 (651 to 1160)	23.2 (18 to 30.5)	-10.7 (-29.8 to 13.7)
		Female	5 (4 to 8)	0.1 (0.1 to 0.2)	-4.9 (-27.4 to 21.7)	277 (197 to 382)	6.1 (4.4 to 8.3)	-7.7 (-31.1 to 22.5)
Malaysia	TBL cancer	Both	1180 (742 to 1700)	4.4 (2.8 to 6.3)	-6.8 (-30.3 to 25.2)	124000 (94800 to 157000)	448.5 (345.8 to 566.8)	-8 (-28.8 to 16.1)
		Male	822 (521 to 1200)	6.2 (4 to 9)	-6.5 (-32.8 to 30.8)	87900 (66800 to 112000)	634.5 (485.3 to 808.2)	-7.8 (-29.1 to 17)
		Female	355 (216 to 527)	2.7 (1.6 to 3.9)	-7.9 (-36.5 to 26.7)	36400 (27800 to 46600)	263.8 (201.6 to 335.9)	-8.7 (-29.6 to 16.5)
	Larynx cancer	Both	296 (196 to 433)	1.1 (0.7 to 1.6)	26.8 (-5.5 to 67.9)	8030 (6090 to 10300)	28.8 (22 to 36.7)	15.5 (-11.2 to 47.3)

		Male	249 (160 to 371)	1.8 (1.2 to 2.7)	28.1 (-7.1 to 73.7)	6840 (5110 to 8910)	49.1 (37 to 63.7)	16.3 (-11.9 to 50.4)
		Female	47 (32 to 67)	0.3 (0.2 to 0.5)	17.7 (-7.2 to 50.5)	1190 (890 to 1570)	8.5 (6.4 to 11.1)	8.9 (-17.6 to 43.8)
Maldives	TBL cancer	Both	6 (4 to 9)	2.2 (1.5 to 3)	-2 (-24.5 to 26.7)	614 (507 to 735)	197.2 (162.3 to 235.5)	-4.6 (-21.7 to 14.9)
		Male	5 (3 to 7)	3.1 (2.1 to 4.3)	-3.2 (-30.7 to 30.9)	474 (385 to 571)	280.4 (227.1 to 338)	-6.4 (-25.1 to 14.6)
		Female	1 (1 to 2)	1.1 (0.7 to 1.6)	7.1 (-24.3 to 47.9)	140 (113 to 172)	100.2 (80.2 to 122.1)	3 (-17 to 27.5)
	Larynx cancer	Both	2 (2 to 3)	0.8 (0.5 to 1.1)	9.6 (-14.8 to 39.9)	53 (43 to 66)	16.6 (13.4 to 20.6)	-7.5 (-26.1 to 15.9)
		Male	2 (2 to 3)	1.3 (0.9 to 1.9)	10.4 (-15.1 to 42.2)	50 (40 to 62)	28.7 (22.9 to 35.7)	-7.5 (-27.2 to 17.5)
		Female	0 (0 to 0)	0.1 (0.1 to 0.1)	1.3 (-18.9 to 26.5)	3 (3 to 4)	2.2 (1.7 to 2.7)	-13.8 (-33.1 to 9.9)
Mali	TBL cancer	Both	140 (89 to 205)	1.7 (1.1 to 2.4)	3.3 (-21.7 to 33.4)	15700 (11700 to 20800)	174.2 (132.3 to 227.7)	4.3 (-13.5 to 25)
		Male	98 (59 to 150)	2.2 (1.4 to 3.4)	0.7 (-29.2 to 42)	10900 (7380 to 14800)	237 (163.2 to 317.4)	1.6 (-17.8 to 24)
		Female	43 (27 to 62)	1 (0.7 to 1.5)	9.1 (-19.5 to 46.4)	4780 (3460 to 6280)	106.2 (77.8 to 137.7)	10.5 (-10.5 to 35.7)
	Larynx cancer	Both	46 (30 to 67)	0.5 (0.3 to 0.7)	0.9 (-17.9 to 26.9)	2430 (1820 to 3270)	25.3 (19.2 to 33.9)	-1.3 (-21.2 to 23)
		Male	35 (22 to 52)	0.7 (0.5 to 1.1)	1.4 (-20.3 to 32.8)	1860 (1330 to 2570)	37.9 (27.7 to 51.8)	-0.6 (-22.4 to 28.4)
		Female	11 (7 to 17)	0.2 (0.2 to 0.4)	-1.8 (-22.5 to 23.9)	575 (422 to 789)	11.8 (8.7 to 15.8)	-4.5 (-25.9 to 22.1)
Malta	TBL cancer	Both	49 (33 to 66)	5.5 (3.7 to 7.4)	-8 (-27 to 16.1)	3930 (3490 to 4390)	458.9 (408 to 515.5)	-9.5 (-19.8 to 1.4)
		Male	38 (26 to 53)	9 (6.2 to 12.4)	-15.3 (-34.2 to 7.5)	3160 (2810 to 3530)	762.7 (679.2 to 848.9)	-15 (-24.3 to -4.5)
		Female	10 (7 to 15)	2.3 (1.5 to 3.3)	11.6 (-21.1 to 50.8)	767 (649 to 902)	182.6 (155.6 to 214.1)	4.4 (-11.4 to 21.2)
	Larynx cancer	Both	14 (9 to 19)	1.7 (1.2 to 2.4)	-0.3 (-21.8 to 25.6)	193 (166 to 225)	24.5 (20.9 to 28.2)	-11.8 (-24.1 to 2.7)
		Male	12 (8 to 17)	3.1 (2.1 to 4.4)	-2.8 (-25.1 to 24.3)	171 (146 to 200)	44.6 (37.9 to 52.1)	-13.9 (-26.7 to 0.9)
		Female	1 (1 to 2)	0.4 (0.2 to 0.5)	-1.4 (-18.8 to 21)	22 (18 to 27)	5.5 (4.6 to 6.8)	-12.3 (-27.9 to 4.6)
Marshall Islands	TBL cancer	Both	2 (1 to 4)	6.6 (3.6 to 10.5)	0.5 (-22.5 to 30.1)	287 (171 to 434)	751.9 (463.1 to 1109.4)	0.1 (-17.5 to 21.5)
		Male	2 (1 to 3)	9.4 (4.6 to 16.3)	-1 (-25.5 to 32.6)	213 (115 to 347)	1086.2 (610.6 to 1750)	-1.3 (-19.2 to 20.9)
		Female	1 (0 to 1)	3.5 (2 to 5.4)	6.2 (-23.6 to 44.9)	74 (49 to 107)	396.9 (265.7 to 566)	6.7 (-14.2 to 31)
	Larynx cancer	Both	0 (0 to 0)	0.6 (0.4 to 0.9)	-3 (-20.8 to 19.3)	10 (7 to 14)	27.2 (19.2 to 36.2)	-7.2 (-23.6 to 14.5)
		Male	0 (0 to 0)	0.9 (0.5 to 1.3)	-2 (-22.4 to 25)	8 (5 to 10)	40.1 (26.8 to 54.5)	-5.9 (-24 to 18.1)
		Female	0 (0 to 0)	0.3 (0.2 to 0.5)	-7.2 (-27 to 15.1)	3 (2 to 4)	13.1 (9.3 to 17.9)	-11.5 (-32.2 to 12.3)
Mauritania	TBL cancer	Both	49 (28 to 75)	2.4 (1.4 to 3.6)	3.4 (-24.5 to 34.5)	5290 (3510 to 7620)	247.5 (169.2 to 349.5)	1 (-23.1 to 25.2)
		Male	32 (17 to 55)	3.2 (1.7 to 5.3)	0.9 (-29.8 to 35.3)	3420 (2100 to 5480)	322.2 (202.6 to 506)	-1.8 (-27.1 to 22.6)

		Female	17 (10 to 26)	1.7 (1 to 2.5)	8.5 (-25.5 to 52.7)	1870 (1250 to 2590)	171.1 (118.1 to 235.8)	7.2 (-19.4 to 33.7)
	Larynx cancer	Both	10 (6 to 14)	0.5 (0.3 to 0.7)	0.5 (-22.6 to 25.5)	436 (297 to 604)	20.5 (14.2 to 27.8)	-6.8 (-30.1 to 17.4)
		Male	9 (5 to 13)	0.8 (0.5 to 1.2)	1.7 (-23 to 30.6)	377 (251 to 539)	35.4 (24.2 to 49.8)	-5.4 (-29.3 to 20.7)
		Female	1 (1 to 2)	0.1 (0.1 to 0.2)	-5.8 (-30.1 to 19.7)	58 (41 to 80)	5.3 (3.8 to 7.2)	-13.5 (-37.7 to 11.5)
Mauritius	TBL cancer	Both	47 (31 to 67)	2.7 (1.8 to 3.8)	-0.8 (-25.5 to 28.9)	4900 (3950 to 5970)	275.2 (222.9 to 333.8)	-3.1 (-22.7 to 19.4)
		Male	33 (21 to 47)	4.1 (2.7 to 5.8)	-5.3 (-32 to 27.6)	3450 (2760 to 4240)	418.4 (337.7 to 510.9)	-7 (-25.6 to 15.6)
		Female	14 (9 to 21)	1.5 (1 to 2.2)	7.3 (-24 to 45.8)	1450 (1160 to 1780)	156.4 (124.8 to 191.7)	4.1 (-17.5 to 28.3)
	Larynx cancer	Both	18 (12 to 26)	1 (0.7 to 1.4)	1.3 (-19.5 to 26.8)	476 (373 to 593)	26.2 (20.5 to 32.7)	-9.1 (-28 to 12.9)
		Male	16 (11 to 23)	1.9 (1.3 to 2.7)	3.5 (-19.1 to 32.4)	432 (337 to 541)	51 (40 to 63.4)	-6.7 (-26.5 to 16.3)
		Female	2 (1 to 3)	0.2 (0.1 to 0.4)	-20.5 (-33.5 to 1.6)	44 (33 to 86)	4.7 (3.6 to 9.3)	-33.4 (-48.4 to -7.8)
Mexico	TBL cancer	Both	2530 (1760 to 3330)	2.2 (1.5 to 2.9)	-1.9 (-15.6 to 13.6)	244000 (208000 to 282000)	206.2 (176.4 to 238.1)	-3.3 (-16.9 to 12)
		Male	1570 (1080 to 2150)	2.9 (2 to 4)	-3.1 (-20.9 to 17.8)	158000 (129000 to 192000)	287.4 (235.7 to 350.4)	-3.9 (-21.5 to 16.6)
		Female	954 (640 to 1300)	1.5 (1 to 2.1)	1.5 (-16.9 to 23)	86500 (69800 to 106000)	135.9 (109.9 to 166.7)	-0.8 (-19.3 to 22.2)
	Larynx cancer	Both	862 (603 to 1170)	0.7 (0.5 to 1)	-2.6 (-15.7 to 12.1)	24700 (20500 to 29700)	20.9 (17.3 to 25)	-10 (-25.3 to 7.1)
		Male	639 (440 to 884)	1.2 (0.8 to 1.6)	-1.7 (-19.5 to 18.6)	21200 (17000 to 26100)	38.5 (31 to 47.3)	-8.7 (-26.5 to 12.2)
		Female	223 (150 to 324)	0.3 (0.2 to 0.5)	-1.7 (-11.6 to 9.2)	3490 (2800 to 5120)	5.5 (4.4 to 8)	-12.1 (-27.9 to 6.8)
Federatead States of Micronesia	TBL cancer	Both	5 (3 to 8)	6.9 (3.7 to 11.1)	5.3 (-18.2 to 34)	614 (352 to 941)	786.8 (465.9 to 1174.9)	4.7 (-18.1 to 27.5)
		Male	4 (2 to 7)	10.6 (5.4 to 18.5)	3.2 (-24.5 to 33.9)	449 (236 to 756)	1199.8 (678.2 to 1925.1)	2.3 (-21.7 to 24.7)
		Female	1 (1 to 2)	3.7 (2 to 6)	9.5 (-22.5 to 51.9)	165 (103 to 245)	420.1 (268.2 to 609.4)	9.4 (-13.9 to 36)
	Larynx cancer	Both	0 (0 to 1)	0.6 (0.4 to 0.9)	3.9 (-18.1 to 29.6)	20 (13 to 27)	25.8 (17.7 to 34.2)	-2.2 (-25.1 to 23.3)
		Male	0 (0 to 1)	1 (0.6 to 1.5)	3.5 (-22.6 to 33.1)	15 (9 to 21)	41.9 (27.7 to 56.6)	-2.8 (-28.6 to 25.4)
		Female	0 (0 to 0)	0.3 (0.2 to 0.5)	3.8 (-18.9 to 31.9)	5 (4 to 8)	12.4 (8.8 to 17.4)	-1.6 (-25.8 to 29.3)
Monaco	TBL cancer	Both	16 (11 to 22)	18.8 (12.6 to 25.6)	-4.8 (-23.4 to 16.7)	1190 (963 to 1430)	1464.5 (1166.8 to 1793.9)	-7.1 (-24.2 to 11.4)
		Male	10 (7 to 14)	24.8 (16.9 to 33.7)	-5.7 (-24.3 to 15.7)	771 (637 to 918)	1961.7 (1590.8 to 2361.7)	-7.8 (-24.1 to 9.7)
		Female	6 (4 to 8)	13.3 (8.4 to 18.8)	-4 (-28.4 to 26.5)	419 (311 to 526)	1007.3 (741.8 to 1279.5)	-6.6 (-26.9 to 17.2)
	Larynx cancer	Both	5 (3 to 7)	6.1 (4 to 8.6)	-6.8 (-27.8 to 20.3)	62 (49 to 76)	81.7 (63.7 to 102)	-10.8 (-29.8 to 13.1)
		Male	4 (3 to 6)	11.4 (7.5 to 16.2)	-7 (-28.4 to 21.6)	56 (45 to 69)	153 (119.1 to 191.1)	-10.7 (-30.4 to 13.8)

		Female	0 (0 to 1)	1 (0.6 to 1.5)	-8.5 (-31.3 to 22.5)	6 (4 to 8)	14.6 (10.5 to 19.3)	-13.1 (-35.4 to 17.2)
Mongolia	TBL cancer	Both	151 (98 to 218)	6.8 (4.5 to 9.7)	1.2 (-23.4 to 33.7)	17400 (13300 to 23000)	720.8 (561.8 to 932.4)	-0.6 (-21.9 to 26.6)
		Male	118 (75 to 176)	12.6 (8.1 to 18)	2.3 (-24.1 to 38.6)	13900 (10300 to 18500)	1322.7 (997.5 to 1725.7)	0.7 (-22.3 to 30.1)
		Female	32 (20 to 47)	2.8 (1.8 to 4)	0.3 (-33 to 46.2)	3480 (2680 to 4570)	274.6 (214.1 to 353.8)	-0.7 (-23.2 to 26.8)
	Larynx cancer	Both	18 (12 to 26)	0.9 (0.6 to 1.2)	-2.2 (-25.9 to 27)	730 (556 to 959)	33.5 (25.5 to 43.4)	-8.4 (-28 to 17.5)
		Male	15 (10 to 22)	1.8 (1.1 to 2.5)	1 (-25.5 to 35.5)	633 (474 to 834)	71.2 (53.3 to 92.2)	-4.7 (-25.7 to 22.1)
		Female	3 (2 to 4)	0.2 (0.2 to 0.4)	-15.3 (-38.4 to 16)	97 (72 to 132)	8 (5.9 to 11.1)	-22.9 (-43.3 to 5.6)
Montenegro	TBL cancer	Both	130 (86 to 180)	13.2 (8.7 to 18.2)	-0.4 (-20.3 to 23.6)	13000 (10500 to 15800)	1330.4 (1083 to 1618.7)	-3.9 (-20.7 to 16.3)
		Male	97 (64 to 137)	21.2 (14.1 to 29.9)	-0.9 (-24.6 to 28)	9770 (7800 to 12200)	2136.1 (1709.4 to 2653.8)	-4.3 (-22.4 to 17.2)
		Female	33 (22 to 48)	6.4 (4.2 to 9.2)	0.7 (-23.5 to 31.3)	3180 (2550 to 3900)	632.8 (506 to 775.5)	-3.2 (-21.5 to 17.6)
	Larynx cancer	Both	44 (30 to 62)	4.6 (3.2 to 6.4)	-0.1 (-22.4 to 28.3)	1200 (968 to 1480)	125.4 (101.2 to 155.6)	-9.7 (-26.4 to 11.7)
		Male	34 (22 to 48)	7.3 (4.9 to 10.4)	1.2 (-25.5 to 35.7)	961 (762 to 1220)	211.1 (167.3 to 265.9)	-8.4 (-26.5 to 15.3)
		Female	11 (7 to 15)	2.2 (1.5 to 3.1)	-4.7 (-26 to 19.2)	238 (193 to 288)	48.8 (39.5 to 59.6)	-15 (-31.7 to 5)
Morocco	TBL cancer	Both	1220 (747 to 1810)	3.7 (2.3 to 5.5)	9.9 (-20.3 to 47.5)	141000 (102000 to 184000)	415.2 (300 to 537.1)	9.6 (-14.5 to 40.1)
		Male	1060 (643 to 1600)	6.4 (3.9 to 9.5)	8.1 (-24.2 to 50.4)	124000 (88400 to 165000)	730.4 (523.3 to 953.7)	7.9 (-16.7 to 39.5)
		Female	162 (102 to 237)	1 (0.6 to 1.5)	17.1 (-10.1 to 55.3)	17100 (12400 to 22300)	101.7 (73.8 to 131.6)	14.9 (-9.5 to 45.8)
	Larynx cancer	Both	611 (387 to 888)	1.8 (1.2 to 2.6)	16.3 (-13.9 to 60.9)	25500 (17900 to 32900)	74.4 (52.8 to 94.8)	4.4 (-18.5 to 36.6)
		Male	555 (348 to 808)	3.3 (2.1 to 4.8)	17.2 (-14.8 to 67)	23400 (16300 to 30500)	137.5 (97.1 to 176.4)	5.1 (-19.1 to 40.5)
		Female	56 (35 to 83)	0.3 (0.2 to 0.5)	4 (-19.3 to 35.1)	2080 (1510 to 2810)	11.8 (8.7 to 15.6)	-9.6 (-31.3 to 20.7)
Mozambique	TBL cancer	Both	200 (122 to 302)	1.9 (1.2 to 2.8)	6.6 (-22.3 to 40.5)	23300 (17100 to 31500)	202 (149.8 to 268.8)	6.1 (-14.5 to 31.3)
		Male	136 (83 to 207)	3 (1.8 to 4.5)	1.6 (-28.5 to 41)	16000 (12100 to 21300)	321.2 (247 to 418)	0.7 (-18.5 to 23.9)
		Female	64 (35 to 106)	1.1 (0.6 to 1.7)	22 (-16.9 to 74.3)	7280 (4590 to 11000)	110.1 (69.3 to 165.7)	23.3 (-12.8 to 68.7)
	Larynx cancer	Both	104 (67 to 150)	0.9 (0.5 to 1.2)	-1.5 (-23.2 to 24.1)	5910 (4390 to 7710)	46.2 (35 to 59.9)	-4.6 (-27 to 19.7)
		Male	84 (54 to 122)	1.6 (1 to 2.2)	-1.4 (-23.3 to 24.9)	4880 (3660 to 6380)	84.4 (64.7 to 109.6)	-4.5 (-27.1 to 20.1)

		Female	20 (12 to 30)	0.3 (0.2 to 0.4)	1.8 (-25 to 36.2)	1040 (666 to 1480)	14.5 (9.5 to 20.7)	-1.3 (-30 to 38.2)
Myanmar	TBL cancer	Both	2330 (1470 to 3600)	5 (3.2 to 7.6)	-0.1 (-23.4 to 28.3)	269000 (198000 to 372000)	551.1 (405.1 to 754.8)	-2.5 (-20 to 20.6)
		Male	1570 (938 to 2610)	7.7 (4.7 to 12.8)	-0.8 (-27.3 to 33)	183000 (129000 to 273000)	857.2 (608.7 to 1269.2)	-3.1 (-21.9 to 23.2)
		Female	758 (472 to 1130)	2.9 (1.8 to 4.2)	5 (-26.5 to 43.5)	86300 (66100 to 115000)	315.8 (243.4 to 416.4)	2.2 (-16.8 to 26.6)
	Larynx cancer	Both	291 (197 to 416)	0.6 (0.4 to 0.9)	-2.7 (-20.1 to 18.2)	12100 (9520 to 15900)	24.7 (19.7 to 32.6)	-13.2 (-29.6 to 6.2)
		Male	220 (145 to 320)	1.1 (0.7 to 1.5)	2.5 (-19 to 31.3)	9380 (7280 to 12800)	43.6 (34.3 to 58.7)	-8.6 (-26.8 to 13.6)
		Female	71 (48 to 100)	0.3 (0.2 to 0.4)	-11.9 (-27.9 to 7.1)	2730 (2080 to 3630)	10.1 (7.7 to 13.4)	-22 (-38.9 to -0.7)
Namibia	TBL cancer	Both	25 (15 to 36)	1.8 (1.1 to 2.5)	6.8 (-17.7 to 42.3)	2740 (2070 to 3580)	187.6 (144.8 to 240.5)	5.6 (-13.8 to 29.5)
		Male	17 (10 to 24)	2.8 (1.8 to 4.2)	4.9 (-23.5 to 44.9)	1870 (1420 to 2380)	301.9 (233.8 to 377.5)	3.2 (-15.4 to 24.8)
		Female	8 (5 to 13)	1 (0.6 to 1.6)	14.6 (-22.5 to 64.8)	872 (593 to 1250)	104.3 (71.4 to 147.6)	14.8 (-16.5 to 57.9)
	Larynx cancer	Both	23 (15 to 33)	1.5 (1 to 2.2)	5.2 (-19.4 to 36)	1130 (843 to 1510)	72.9 (55.4 to 96.1)	-3.8 (-24.4 to 21.7)
		Male	18 (12 to 27)	2.8 (1.8 to 4.1)	7.6 (-20 to 43.1)	935 (704 to 1240)	138.7 (106.6 to 180.5)	-1.6 (-21.4 to 23.1)
		Female	4 (3 to 7)	0.5 (0.3 to 0.8)	0.8 (-27.2 to 40.5)	190 (124 to 282)	22 (14.6 to 32.6)	-9.1 (-36.7 to 28.1)
Nauru	TBL cancer	Both	0 (0 to 1)	8.4 (4.7 to 12.6)	-1.4 (-25.3 to 27.6)	48 (28 to 67)	947 (598.7 to 1267.8)	-2.8 (-24.1 to 20.2)
		Male	0 (0 to 0)	12.7 (7 to 19.7)	-0.2 (-26.2 to 35.2)	34 (19 to 49)	1445.8 (883.9 to 2021.1)	-1.9 (-24.3 to 24)
		Female	0 (0 to 0)	4.7 (2.5 to 7.8)	19 (-14.6 to 69.8)	14 (8 to 22)	537.3 (326.9 to 790.5)	16.4 (-7.6 to 46.3)
	Larynx cancer	Both	0 (0 to 0)	0.8 (0.5 to 1.2)	-10.3 (-28 to 13.1)	1 (1 to 2)	29.7 (22.8 to 37.7)	-22.4 (-36.2 to -3.4)
		Male	0 (0 to 0)	1.4 (0.9 to 2)	-3 (-24.4 to 27.2)	1 (1 to 2)	49.6 (35.4 to 65.3)	-16.3 (-33 to 6.4)
		Female	0 (0 to 0)	0.4 (0.2 to 0.6)	-5.3 (-26.5 to 23.6)	0 (0 to 1)	12.7 (8.9 to 17.5)	-18.5 (-37.4 to 10.5)
Nepal	TBL cancer	Both	413 (254 to 614)	1.9 (1.1 to 2.7)	14.2 (-15 to 53.2)	44800 (32000 to 58700)	193.2 (138.5 to 251.1)	12.8 (-11.6 to 40.7)
		Male	279 (163 to 418)	2.6 (1.6 to 3.9)	13.5 (-20.6 to 59.9)	30200 (21500 to 39200)	274.5 (196.7 to 354.1)	12.2 (-12.7 to 38.7)
		Female	134 (75 to 216)	1.1 (0.6 to 1.8)	22.1 (-13.2 to 74.8)	14600 (9820 to 20800)	118.7 (79.7 to 169.8)	20.5 (-5.7 to 57.2)
	Larynx cancer	Both	296 (193 to 448)	1.3 (0.8 to 1.9)	13.6 (-10.3 to 48.3)	14500 (10900 to 19900)	61.4 (46.1 to 82.9)	9.1 (-13.4 to 34.6)
		Male	229 (145 to 357)	2.1 (1.3 to 3.2)	18.2 (-10.5 to 59.9)	11400 (8220 to 16000)	101.8 (73.9 to 142.3)	14.6 (-11.5 to 44.8)

		Female	66 (43 to 97)	0.5 (0.4 to 0.8)	9.6 (-15.6 to 42.8)	3130 (2280 to 4150)	24.6 (18 to 32.5)	2.4 (-20.2 to 32.5)
Netherlands	TBL cancer	Both	3680 (2490 to 5160)	11.1 (7.4 to 15.7)	-9.1 (-31.3 to 18.8)	262000 (246000 to 278000)	814.2 (767.6 to 862.1)	-13.3 (-17.5 to -8.3)
		Male	2130 (1420 to 3010)	13.4 (8.9 to 19)	-16.8 (-38 to 10.3)	153000 (143000 to 163000)	974.4 (912.2 to 1037.7)	-19.5 (-24.5 to -14.2)
		Female	1560 (1040 to 2230)	9.4 (6.2 to 13.5)	-0.1 (-26 to 33.6)	109000 (99900 to 118000)	681.6 (627 to 736.4)	-6.1 (-13.3 to 1.7)
	Larynx cancer	Both	475 (314 to 686)	1.5 (1 to 2.2)	-4.9 (-28.7 to 21.7)	5480 (4960 to 6000)	17.4 (15.8 to 19.1)	-12.5 (-20.7 to -3.3)
		Male	397 (259 to 581)	2.6 (1.7 to 3.8)	-7 (-31.2 to 22)	4430 (3970 to 4890)	29.1 (26.3 to 32.3)	-14.5 (-23.3 to -3.9)
		Female	78 (46 to 115)	0.5 (0.3 to 0.8)	-2.8 (-26.8 to 25.6)	1050 (685 to 1220)	6.6 (4.4 to 7.7)	-10.4 (-22.2 to 2.8)
New Zealand	TBL cancer	Both	624 (439 to 833)	8.1 (5.7 to 10.8)	-3.7 (-21.8 to 17.2)	40300 (38000 to 42600)	542.3 (511 to 571.1)	-7.6 (-12.7 to -2.6)
		Male	326 (218 to 453)	8.9 (6 to 12.4)	-7.6 (-32.6 to 22.5)	20700 (19400 to 22100)	582.3 (543.6 to 619.7)	-11 (-16.8 to -5.1)
		Female	298 (200 to 414)	7.4 (5 to 10.3)	0.1 (-26.2 to 30.4)	19600 (18000 to 21300)	508.4 (469.9 to 550.8)	-4.3 (-11.7 to 4.2)
	Larynx cancer	Both	70 (46 to 100)	0.9 (0.6 to 1.3)	-4.5 (-28.8 to 24.3)	760 (694 to 826)	10.4 (9.6 to 11.2)	-11.8 (-19.1 to -3.9)
		Male	64 (40 to 93)	1.8 (1.1 to 2.6)	-4.7 (-30.9 to 26.1)	586 (529 to 645)	16.8 (15.3 to 18.5)	-11.7 (-20.8 to -1.7)
		Female	6 (4 to 9)	0.2 (0.1 to 0.2)	-5.4 (-23.3 to 15.6)	175 (150 to 205)	4.7 (4 to 5.4)	-11.9 (-22.5 to 0.4)
Nicaragua	TBL cancer	Both	85 (57 to 119)	2 (1.3 to 2.8)	-1.2 (-25.7 to 26.7)	8370 (6760 to 10200)	186.2 (151.7 to 225.7)	-3.2 (-21.1 to 18.1)
		Male	50 (32 to 71)	2.6 (1.7 to 3.8)	-1.7 (-31.1 to 37.1)	5070 (3940 to 6290)	254.7 (199.6 to 312.1)	-3.5 (-23.4 to 20.4)
		Female	36 (23 to 50)	1.5 (1 to 2.1)	2.6 (-24.8 to 40.2)	3300 (2680 to 4000)	131.7 (108.2 to 158.6)	0.4 (-17.4 to 21.7)
	Larynx cancer	Both	36 (24 to 50)	0.8 (0.5 to 1.1)	4 (-22 to 36.1)	1140 (884 to 1450)	25.8 (20.1 to 32.7)	-6.7 (-27.1 to 18.8)
		Male	29 (19 to 42)	1.5 (1 to 2.2)	9.3 (-21.2 to 48.8)	1010 (769 to 1290)	51.1 (39.4 to 65.4)	-2.1 (-24.5 to 26.4)
		Female	6 (4 to 8)	0.2 (0.2 to 0.3)	-5.3 (-20.7 to 14.5)	136 (108 to 170)	5.5 (4.4 to 6.8)	-17.2 (-33.9 to 4.8)
Niger	TBL cancer	Both	154 (84 to 243)	2.1 (1.1 to 3.2)	7.1 (-18.4 to 40.4)	17300 (10700 to 25100)	212.9 (134.6 to 307.9)	8.6 (-12 to 35.5)
		Male	119 (64 to 198)	3.3 (1.8 to 5.5)	7.9 (-20.7 to 46.9)	13500 (8320 to 20900)	345.4 (212.7 to 525.5)	8.9 (-11.9 to 36.7)
		Female	34 (18 to 59)	0.9 (0.4 to 1.5)	11.6 (-14.2 to 43.7)	3820 (2320 to 6090)	87.2 (53.6 to 137.6)	14.7 (-6.8 to 41.7)
	Larynx cancer	Both	40 (25 to 58)	0.5 (0.3 to 0.7)	3.4 (-17.1 to 31.7)	2030 (1440 to 2700)	24.5 (17.6 to 32.2)	2.5 (-17.4 to 30.6)

		Male	35 (21 to 51)	0.9 (0.6 to 1.3)	5.4 (-17.5 to 37.9)	1770 (1230 to 2410)	43.9 (31 to 58.4)	4.6 (-17.1 to 33.6)
		Female	5 (3 to 8)	0.1 (0.1 to 0.2)	1.2 (-20.9 to 29.2)	262 (183 to 350)	6 (4.2 to 7.9)	-0.6 (-23.5 to 28.5)
Nigeria	TBL cancer	Both	1380 (880 to 1970)	1.7 (1.1 to 2.4)	-1.7 (-28 to 34.8)	150000 (112000 to 199000)	171 (130.1 to 226.6)	-2.7 (-28.9 to 34.8)
		Male	926 (577 to 1410)	2.4 (1.5 to 3.6)	-3.3 (-35 to 48.1)	100000 (69700 to 146000)	247.8 (174.9 to 355)	-4.1 (-35.5 to 48)
		Female	449 (275 to 671)	1 (0.6 to 1.5)	21.4 (-17.1 to 73.7)	49400 (34100 to 69600)	101.2 (71.1 to 137.9)	22.6 (-17.2 to 85.2)
	Larynx cancer	Both	557 (363 to 823)	0.6 (0.4 to 0.9)	-14.8 (-39.4 to 22.4)	27300 (19100 to 39600)	28 (20 to 39.2)	-17.9 (-44.7 to 25.8)
		Male	514 (330 to 775)	1.2 (0.8 to 1.8)	-6.3 (-35.1 to 38.3)	26100 (18000 to 38000)	57.2 (40.2 to 81.5)	-8.9 (-40 to 42.6)
		Female	43 (26 to 64)	0.1 (0 to 0.1)	-3 (-27.1 to 27.6)	1240 (857 to 1750)	2.4 (1.7 to 3.3)	-5.2 (-33.6 to 42.8)
Niue	TBL cancer	Both	0 (0 to 0)	6.6 (4.5 to 9.2)	-0.6 (-18.7 to 21.4)	15 (12 to 19)	710.7 (560.4 to 890.1)	-0.8 (-14.3 to 14.7)
		Male	0 (0 to 0)	9.6 (6.6 to 13)	-2 (-20.6 to 23.3)	10 (8 to 13)	1026.4 (832.1 to 1232.7)	-2.3 (-14.9 to 13)
		Female	0 (0 to 0)	4.2 (2.6 to 6.3)	2.7 (-25.5 to 38)	5 (4 to 7)	441.7 (312.4 to 597.6)	2.1 (-17.6 to 26.6)
	Larynx cancer	Both	0 (0 to 0)	0.6 (0.4 to 0.9)	-2.7 (-19.1 to 15.4)	0 (0 to 0)	16.8 (13.4 to 20.9)	-8.3 (-22.1 to 7)
		Male	0 (0 to 0)	1.1 (0.7 to 1.5)	-1.2 (-20.1 to 20.8)	0 (0 to 0)	29.4 (23.3 to 36.8)	-6.8 (-21.3 to 10.4)
		Female	0 (0 to 0)	0.2 (0.2 to 0.4)	-5.2 (-26.3 to 21.6)	0 (0 to 0)	6.8 (5 to 9.3)	-10.6 (-32.4 to 18)
North Macedonia	TBL cancer	Both	310 (204 to 443)	9.3 (6.1 to 13.3)	-3.6 (-29.8 to 26.7)	32800 (25000 to 42100)	991.1 (753.4 to 1273.7)	-5.9 (-29.3 to 20.4)
		Male	249 (162 to 355)	15.4 (10 to 21.9)	-6.5 (-32.8 to 25.1)	26700 (20000 to 34700)	1635.6 (1229.1 to 2122)	-8.5 (-31.8 to 17.4)
		Female	60 (38 to 88)	3.7 (2.3 to 5.3)	3.4 (-26.8 to 43.3)	6100 (4710 to 7860)	373.9 (287.8 to 482.4)	0.6 (-23.4 to 29.6)
	Larynx cancer	Both	97 (64 to 138)	3 (2 to 4.2)	-6.4 (-30.8 to 24.1)	3050 (2320 to 3940)	93 (70.8 to 120.1)	-13.6 (-33.8 to 10.6)
		Male	86 (55 to 123)	5.3 (3.5 to 7.6)	-8.2 (-33.1 to 24)	2800 (2120 to 3630)	173.9 (132.1 to 225.2)	-14.9 (-34.8 to 9.5)
		Female	12 (8 to 17)	0.8 (0.5 to 1.1)	-2.3 (-22.3 to 24.8)	252 (192 to 326)	15.6 (11.9 to 20.3)	-11.8 (-32.8 to 14.7)
Northern Mariana Islands	TBL cancer	Both	5 (4 to 7)	9.8 (6.9 to 13)	-5.1 (-20.3 to 13.4)	567 (476 to 654)	1011.9 (867.5 to 1150.3)	-5.3 (-17.6 to 8.2)
		Male	4 (3 to 5)	15 (10.6 to 19.8)	-3.5 (-20.3 to 16.6)	433 (363 to 498)	1531.9 (1323.8 to 1727.6)	-4.3 (-17.2 to 10.4)
		Female	1 (1 to 2)	4.7 (3.1 to 6.8)	3 (-25.6 to 40.4)	134 (106 to 170)	482.2 (386.9 to 603.7)	3.6 (-15.4 to 27.6)
	Larynx cancer	Both	0 (0 to 0)	0.6 (0.4 to 0.9)	-34.2 (-46.7 to -14.2)	8 (6 to 10)	13.8 (11.4 to 17.8)	-39.1 (-49.3 to -22.2)
		Male	0 (0 to 0)	0.9 (0.6 to 1.4)	-33.6 (-48.3 to -6.7)	5 (4 to 7)	20.7 (17.1 to 27)	-38.7 (-50.4 to -21.3)

		Female	0 (0 to 0)	0.3 (0.2 to 0.4)	-24.4 (-44.2 to 1.7)	2 (2 to 3)	7.5 (5.8 to 9.6)	-29.1 (-45.3 to -6.6)
Norway	TBL cancer	Both	754 (529 to 996)	8 (5.6 to 10.6)	-16.1 (-29.4 to -1.8)	46500 (43800 to 48900)	511.5 (482.4 to 537.2)	-17.3 (-20.9 to -14.3)
		Male	406 (278 to 555)	9 (6.1 to 12.4)	-22.8 (-38.9 to -3.1)	25400 (23700 to 26900)	571.3 (532.4 to 606.5)	-25 (-29.3 to -21.1)
		Female	347 (237 to 474)	7.2 (4.9 to 10)	-8.2 (-26.3 to 13.5)	21200 (19700 to 22600)	459.6 (428.5 to 488.9)	-7.2 (-12.4 to -1.7)
	Larynx cancer	Both	94 (64 to 129)	1.1 (0.7 to 1.5)	-10.8 (-26.5 to 7.8)	792 (746 to 860)	8.9 (8.4 to 9.7)	-18.1 (-22.1 to -10.7)
		Male	80 (54 to 113)	1.9 (1.2 to 2.6)	-13.2 (-30.6 to 8.5)	629 (590 to 690)	14.5 (13.6 to 15.9)	-20.8 (-25.4 to -13)
		Female	13 (9 to 19)	0.3 (0.2 to 0.5)	-5.7 (-20.5 to 12.1)	163 (139 to 182)	3.7 (3.2 to 4.1)	-11.9 (-18.6 to -2.5)
Oman	TBL cancer	Both	35 (23 to 53)	2.2 (1.5 to 3.2)	-17.4 (-38.8 to 11.1)	3840 (2970 to 5200)	209.5 (168.4 to 265.1)	-23.1 (-37.7 to -0.6)
		Male	24 (15 to 38)	2.8 (1.8 to 4.2)	-20.9 (-45.9 to 15.5)	2730 (2000 to 4040)	271.5 (209.5 to 363)	-25.8 (-42.5 to 1.1)
		Female	11 (7 to 16)	1.6 (1 to 2.3)	-3 (-34.5 to 40.1)	1110 (794 to 1360)	140.2 (103 to 167.8)	-10.2 (-32.7 to 11.9)
	Larynx cancer	Both	14 (9 to 19)	0.7 (0.5 to 0.9)	-20.6 (-34.2 to -3.4)	284 (225 to 356)	14.4 (11.9 to 17.6)	-36.2 (-47.9 to -22.6)
		Male	12 (8 to 17)	1.1 (0.7 to 1.5)	-19.6 (-34.9 to 0.1)	249 (191 to 322)	22.8 (18.4 to 28.2)	-35.2 (-48.3 to -19.9)
		Female	2 (1 to 3)	0.2 (0.1 to 0.3)	-16.9 (-32.7 to 3.2)	35 (28 to 43)	4.2 (3.4 to 5.2)	-32 (-46 to -14.1)
Pakistan	TBL cancer	Both	4260 (2800 to 6130)	3.7 (2.4 to 5.3)	-4.9 (-31.2 to 33.5)	518000 (396000 to 675000)	422 (322.8 to 547.2)	-6.2 (-29.8 to 26.9)
		Male	3540 (2210 to 5260)	5.9 (3.7 to 8.8)	-6.6 (-36.2 to 36.8)	433000 (315000 to 588000)	684 (499.6 to 921.8)	-8 (-35.3 to 30.1)
		Female	717 (437 to 1040)	1.3 (0.8 to 1.9)	10.1 (-23.2 to 58.3)	85000 (61500 to 113000)	140.5 (102.9 to 187.7)	10.4 (-17.9 to 51.2)
	Larynx cancer	Both	3520 (2320 to 4920)	2.9 (1.9 to 4)	-5.6 (-31.3 to 28.8)	197000 (150000 to 261000)	152.2 (116.6 to 199.9)	-10.6 (-33.7 to 23)
		Male	2950 (1880 to 4220)	4.7 (3 to 6.6)	-5.1 (-35.6 to 36.3)	167000 (122000 to 231000)	252.5 (183.7 to 345.1)	-10 (-36 to 29.5)
		Female	570 (373 to 826)	0.9 (0.6 to 1.3)	-3.8 (-27.7 to 33.4)	29100 (21700 to 39000)	44.5 (33.9 to 59)	-9.1 (-32.8 to 25.2)
Palau	TBL cancer	Both	2 (1 to 3)	9.7 (6.7 to 13.5)	1.3 (-18.5 to 26.3)	244 (193 to 309)	1062.1 (847.7 to 1327.2)	0.4 (-15.4 to 22)
		Male	1 (1 to 2)	10.5 (7 to 15.1)	-1 (-23.9 to 27.7)	137 (108 to 175)	1155.5 (930.4 to 1450.9)	-2.2 (-19.1 to 18.9)
		Female	1 (1 to 1)	8.8 (5.8 to 12.4)	3.7 (-22.4 to 37.6)	107 (82 to 137)	956.2 (732.3 to 1212.1)	3.2 (-16.6 to 30.5)
	Larynx cancer	Both	0 (0 to 0)	0.5 (0.3 to 0.7)	-0.4 (-18.8 to 24.5)	3 (3 to 4)	13.8 (10.6 to 17.7)	-5.5 (-23 to 18.4)
		Male	0 (0 to 0)	0.5 (0.3 to 0.7)	1.7 (-18.3 to 26.7)	1 (1 to 2)	12 (9.4 to 15.1)	-4.2 (-22 to 19.1)
		Female	0 (0 to 0)	0.6 (0.3 to 0.8)	-1.5 (-26.8 to 35.2)	2 (1 to 3)	16.1 (11.4 to 22)	-6.6 (-29.4 to 25.3)
Palestine	TBL cancer	Both	120 (82 to 163)	5 (3.4 to 6.7)	16.4 (-8.3 to 48.8)	14100 (11900 to 16500)	541.4 (458.6 to 634.4)	16.3 (-4.2 to 38.1)

		Male	92 (63 to 127)	8.2 (5.5 to 11.1)	9.7 (-17.6 to 42.7)	11100 (9390 to 13000)	893.4 (760.7 to 1035)	10.7 (-9.2 to 32.6)
		Female	27 (19 to 39)	2.1 (1.4 to 3)	27.5 (-7.4 to 79.1)	3020 (2470 to 3640)	217.5 (177 to 262.5)	23.1 (-2.7 to 53.7)
	Larynx cancer	Both	18 (12 to 24)	0.7 (0.5 to 1)	10.6 (-12.5 to 36.2)	544 (444 to 657)	21.7 (17.9 to 26.3)	-3.3 (-21.5 to 17.8)
		Male	16 (11 to 22)	1.3 (0.9 to 1.8)	6.2 (-16.8 to 32.6)	499 (403 to 606)	41.8 (34.2 to 50.5)	-7 (-24.9 to 14.2)
		Female	2 (1 to 2)	0.1 (0.1 to 0.2)	9.8 (-11.1 to 34.2)	45 (36 to 55)	3.3 (2.7 to 4.1)	-4.8 (-24.7 to 19.2)
Panama	TBL cancer	Both	105 (67 to 154)	2.6 (1.6 to 3.7)	2.1 (-25.5 to 38.8)	9650 (7360 to 12400)	233.3 (177.8 to 299.2)	-1.2 (-24.3 to 28.4)
		Male	64 (40 to 95)	3.2 (2 to 4.8)	-6.1 (-34.9 to 32.6)	6160 (4640 to 8040)	305.1 (229.9 to 397.6)	-7.3 (-30.8 to 22.4)
		Female	41 (26 to 60)	1.9 (1.2 to 2.8)	18.9 (-19.8 to 70)	3490 (2690 to 4490)	165 (127.4 to 212.3)	12.7 (-13.7 to 47.4)
	Larynx cancer	Both	32 (20 to 46)	0.8 (0.5 to 1.1)	-10.4 (-33.9 to 19.2)	951 (701 to 1260)	22.9 (16.9 to 30.3)	-22.6 (-43.4 to 3)
		Male	28 (18 to 40)	1.4 (0.9 to 2)	-10.1 (-35.2 to 21.6)	852 (620 to 1130)	41.9 (30.6 to 55.8)	-22.1 (-43.7 to 4)
		Female	4 (3 to 6)	0.2 (0.1 to 0.3)	-7.5 (-26.3 to 16.2)	100 (74 to 133)	4.7 (3.5 to 6.3)	-21.9 (-41.8 to 2.9)
Papua New Guinea	TBL cancer	Both	228 (131 to 364)	4.8 (2.8 to 7.5)	3.8 (-18.1 to 29.9)	28400 (19400 to 43500)	539.9 (379.9 to 819.6)	2.9 (-14.1 to 25.4)
		Male	184 (103 to 305)	7.3 (4.2 to 12.1)	1.7 (-21.7 to 33)	23000 (15700 to 36300)	829.8 (574.9 to 1296.7)	1.3 (-16.8 to 24.7)
		Female	44 (23 to 76)	1.9 (1 to 3.4)	11.5 (-24.8 to 72.1)	5370 (3360 to 8590)	216.2 (136.2 to 345.9)	10.5 (-12.2 to 39.2)
	Larynx cancer	Both	20 (13 to 29)	0.4 (0.3 to 0.6)	-1.2 (-18.8 to 18.4)	973 (698 to 1330)	19.1 (13.9 to 25.6)	-4.5 (-20.7 to 14.6)
		Male	15 (10 to 22)	0.6 (0.4 to 0.9)	-1.8 (-22.3 to 22.1)	751 (521 to 1050)	28.9 (20.6 to 39.2)	-4.6 (-23 to 18.5)
		Female	5 (3 to 7)	0.2 (0.1 to 0.3)	-2.7 (-25.1 to 27)	222 (150 to 318)	8.5 (5.8 to 12.2)	-5.8 (-28.8 to 26.6)
Paraguay	TBL cancer	Both	197 (124 to 290)	3.6 (2.3 to 5.2)	1.8 (-28.5 to 42.5)	20700 (15500 to 26700)	364.5 (273.9 to 470.7)	-0.1 (-26.2 to 31.6)
		Male	149 (91 to 220)	5.7 (3.5 to 8.3)	0.1 (-32.4 to 43.9)	15800 (11800 to 20700)	579.3 (435.7 to 756.3)	-1.6 (-28.1 to 31.2)
		Female	49 (30 to 73)	1.7 (1.1 to 2.5)	8.6 (-26.3 to 58.5)	4850 (3610 to 6310)	164.5 (122.5 to 214.2)	6.3 (-22.4 to 42.8)
	Larynx cancer	Both	56 (36 to 83)	1 (0.6 to 1.5)	16.9 (-15 to 59.7)	2180 (1600 to 2890)	37.6 (27.7 to 49.9)	5.5 (-23.7 to 42.9)
		Male	51 (33 to 76)	1.9 (1.2 to 2.8)	17.1 (-15.6 to 62.1)	2020 (1470 to 2690)	71.5 (52.4 to 94.9)	5.9 (-23.8 to 44.3)
		Female	5 (3 to 7)	0.2 (0.1 to 0.2)	17.3 (-13.1 to 55.8)	158 (117 to 206)	5.3 (4 to 7)	4.7 (-23.8 to 41.9)
Peru	TBL cancer	Both	777 (485 to 1160)	2.4 (1.5 to 3.6)	-8.9 (-38.9 to 29.9)	76100 (56400 to 101000)	235.8 (175 to 313.4)	-12.3 (-38.8 to 23.9)
		Male	408 (247 to 617)	2.7 (1.6 to 4)	-12.6 (-44.3 to 31.9)	40300 (28500 to 54500)	258.9 (183.6 to 350.8)	-15.3 (-43.5 to 22.5)
		Female	369 (225 to 559)	2.2 (1.3 to 3.4)	-4.2 (-36.6 to 40.8)	35800 (27000 to 47300)	214.2 (162.2 to 282.1)	-8.4 (-35.3 to 28.8)

	Larynx cancer	Both	116 (73 to 170)	0.4 (0.2 to 0.5)	-6.6 (-34 to 30.6)	3750 (2720 to 5110)	11.7 (8.5 to 16)	-19.1 (-44 to 13.6)
		Male	90 (57 to 133)	0.6 (0.4 to 0.9)	-5.2 (-35.1 to 36.5)	2910 (2090 to 4070)	18.9 (13.6 to 26.4)	-17.4 (-43.4 to 17.9)
		Female	26 (16 to 38)	0.2 (0.1 to 0.2)	-9.4 (-36.1 to 27.8)	837 (609 to 1130)	5 (3.6 to 6.7)	-23 (-47.7 to 9.8)
Philippines	TBL cancer	Both	3190 (2120 to 4430)	4 (2.7 to 5.5)	2.1 (-19.8 to 26.6)	370000 (297000 to 455000)	439.6 (355.2 to 539.5)	1.3 (-19 to 24.8)
		Male	2180 (1410 to 3180)	5.9 (3.9 to 8.5)	-2.8 (-28.6 to 27.9)	256000 (193000 to 332000)	652.3 (494.2 to 842.3)	-3.8 (-27.7 to 25.5)
		Female	1000 (646 to 1450)	2.4 (1.5 to 3.4)	15.8 (-15.4 to 55.2)	114000 (85800 to 149000)	257.2 (195.2 to 333.9)	15.5 (-15.6 to 54.8)
	Larynx cancer	Both	517 (353 to 724)	0.6 (0.4 to 0.9)	-0.3 (-19.8 to 22.2)	18300 (14500 to 23400)	21.7 (17.3 to 27.5)	-5.2 (-25.5 to 19.2)
		Male	385 (257 to 557)	1 (0.7 to 1.5)	1.7 (-22.7 to 31.7)	15100 (11300 to 20000)	38.2 (28.8 to 50.2)	-3.6 (-28.6 to 26.2)
		Female	132 (87 to 192)	0.3 (0.2 to 0.4)	-4.5 (-19.3 to 13.1)	3230 (2450 to 4160)	7.4 (5.6 to 9.4)	-10.9 (-32.3 to 15.5)
Poland	TBL cancer	Both	6570 (4450 to 8870)	9.5 (6.5 to 12.9)	-6.3 (-22.5 to 12)	703000 (580000 to 840000)	1042.2 (857.8 to 1248.5)	-8.5 (-24.4 to 10)
		Male	4530 (2950 to 6370)	15.1 (9.8 to 21.2)	-12.1 (-31.5 to 10.2)	495000 (385000 to 622000)	1641 (1276.6 to 2068.6)	-14.2 (-33 to 8.3)
		Female	2040 (1370 to 2860)	5.3 (3.5 to 7.5)	4.9 (-19.3 to 33.9)	208000 (163000 to 265000)	565.8 (442.8 to 724.5)	3.9 (-18.2 to 32.9)
	Larynx cancer	Both	1440 (972 to 1950)	2.2 (1.5 to 3)	-6.2 (-24.7 to 16.2)	44200 (35000 to 55000)	68.9 (54.5 to 86)	-16.1 (-33.9 to 4.8)
		Male	1230 (810 to 1710)	4.2 (2.7 to 5.7)	-8.7 (-28.5 to 16)	39800 (30400 to 50900)	134.8 (103.4 to 172.6)	-17.8 (-37.3 to 4.8)
		Female	211 (139 to 304)	0.6 (0.4 to 0.9)	-1.6 (-22.1 to 24.8)	4440 (3300 to 5650)	12.9 (9.5 to 16.4)	-13.2 (-33.1 to 9.9)
Portugal	TBL cancer	Both	1070 (691 to 1550)	5 (3.2 to 7.3)	-10.9 (-34.7 to 19)	101000 (93700 to 107000)	500.6 (466.8 to 532.4)	-12.1 (-18.6 to -5.7)
		Male	804 (516 to 1180)	8.4 (5.3 to 12.2)	-14.7 (-38 to 15.9)	77200 (72000 to 82400)	836.1 (778.6 to 894.8)	-15.7 (-22.2 to -9.2)
		Female	263 (162 to 391)	2.2 (1.4 to 3.3)	3.4 (-30 to 44.6)	23300 (21100 to 25200)	217 (198.3 to 235.5)	2.8 (-6.6 to 13.3)
	Larynx cancer	Both	311 (206 to 455)	1.6 (1 to 2.3)	-15.7 (-38.4 to 14.6)	9290 (8370 to 10200)	48.8 (43.9 to 53.9)	-26.1 (-34.3 to -16.7)
		Male	286 (188 to 423)	3.2 (2.1 to 4.7)	-16.7 (-39.8 to 14.6)	8890 (7990 to 9770)	101 (90.6 to 111.8)	-26.4 (-34.6 to -16.7)
		Female	25 (16 to 38)	0.2 (0.2 to 0.4)	2.1 (-18.3 to 27.7)	401 (339 to 544)	3.6 (3 to 5.1)	-7.8 (-20.4 to 7)
Puerto Rico	TBL cancer	Both	206 (133 to 293)	3 (1.9 to 4.3)	-7.1 (-31.5 to 22.1)	16300 (12700 to 20700)	249.7 (193.6 to 320.2)	-9.6 (-30 to 16)

		Male	128 (79 to 184)	4.2 (2.6 to 6)	-10.6 (-38 to 23.2)	10100 (7780 to 13000)	343.2 (263.8 to 446.1)	-13.3 (-33.9 to 13.1)
		Female	79 (50 to 114)	2 (1.3 to 2.9)	-1.3 (-27.7 to 36.7)	6210 (4880 to 7870)	172.5 (133.8 to 220)	-3.3 (-25.9 to 23.6)
	Larynx cancer	Both	62 (40 to 90)	1 (0.6 to 1.4)	-1.4 (-25.5 to 29.7)	1340 (1010 to 1740)	21.3 (15.9 to 28)	-7.8 (-31.5 to 20.8)
		Male	54 (35 to 78)	1.8 (1.2 to 2.7)	-1.7 (-26.8 to 31.3)	1190 (893 to 1570)	41.8 (30.8 to 55.1)	-8.1 (-32 to 20.8)
		Female	9 (5 to 13)	0.3 (0.2 to 0.4)	-1.1 (-21 to 23.8)	146 (107 to 216)	4.1 (3 to 6.1)	-8.4 (-31.7 to 20.5)
Qatar	TBL cancer	Both	30 (19 to 48)	4 (2.6 to 5.8)	-14.3 (-37.8 to 19.3)	3470 (2420 to 4830)	369.9 (276.6 to 486.9)	-18.3 (-39.1 to 8.5)
		Male	25 (15 to 40)	4.4 (2.8 to 6.5)	-19.6 (-45.2 to 15.6)	2900 (1970 to 4150)	422.5 (312.7 to 568.2)	-23.2 (-45.2 to 5.6)
		Female	6 (4 to 8)	2.6 (1.7 to 3.8)	-2.9 (-29.3 to 30.8)	566 (417 to 726)	217.9 (168.7 to 271.8)	-8.6 (-27.1 to 13.3)
	Larynx cancer	Both	21 (13 to 32)	2.3 (1.3 to 3.4)	0.5 (-27.4 to 41.5)	393 (258 to 552)	47.3 (30.9 to 64.6)	-14.8 (-38 to 18.6)
		Male	20 (12 to 29)	2.9 (1.6 to 4.4)	-6.2 (-33.9 to 34.5)	369 (239 to 521)	60.6 (39.4 to 83.4)	-20.9 (-43.1 to 10.9)
		Female	2 (1 to 2)	0.4 (0.3 to 0.6)	-7.2 (-26.8 to 17.7)	24 (17 to 33)	6.5 (4.9 to 8.4)	-23.6 (-41.1 to -1.9)
South Korea	TBL cancer	Both	8770 (6060 to 12000)	9.8 (6.8 to 13.3)	-0.2 (-19.7 to 23.1)	439000 (399000 to 479000)	488.6 (443.5 to 533.2)	-13.2 (-20.1 to -5.6)
		Male	6230 (4220 to 8590)	15.9 (10.8 to 21.9)	-6 (-26.4 to 18.9)	324000 (290000 to 358000)	796.2 (712.5 to 879.2)	-18.2 (-25.9 to -9)
		Female	2540 (1740 to 3470)	5.1 (3.5 to 7)	5.9 (-17.3 to 35)	116000 (102000 to 128000)	240.1 (212.4 to 264.6)	-7.1 (-15.3 to 1.4)
	Larynx cancer	Both	1490 (977 to 2100)	1.7 (1.1 to 2.3)	-4.5 (-27.3 to 21.3)	10200 (8890 to 11900)	11.3 (9.9 to 13.2)	-17.8 (-27.8 to -6)
		Male	1310 (845 to 1850)	3.2 (2.1 to 4.4)	-8.8 (-31.7 to 18)	9230 (7980 to 11000)	22.7 (19.7 to 26.8)	-21.2 (-31.3 to -9.2)
		Female	180 (122 to 258)	0.4 (0.3 to 0.6)	-5.6 (-19.3 to 11.4)	942 (797 to 1120)	2 (1.7 to 2.4)	-22.5 (-33.3 to -9.1)
Moldova	TBL cancer	Both	250 (170 to 348)	4.3 (2.9 to 5.9)	-15.9 (-33.5 to 3.9)	27200 (23300 to 31400)	473.1 (406 to 544.7)	-20.7 (-33.6 to -7.3)
		Male	195 (132 to 272)	7.9 (5.3 to 10.9)	-18.1 (-38.3 to 4.2)	21900 (18500 to 25400)	875.2 (739.8 to 1012)	-22.5 (-36 to -9.3)
		Female	54 (35 to 79)	1.6 (1.1 to 2.4)	-3.8 (-34.4 to 37)	5310 (4300 to 6510)	166.4 (134.1 to 204)	-9.7 (-29.3 to 13.6)
	Larynx cancer	Both	103 (68 to 143)	1.8 (1.2 to 2.5)	-13.9 (-34.7 to 11.8)	4070 (3440 to 4760)	71.5 (60.4 to 83.5)	-25.8 (-38.1 to -12.4)
		Male	98 (65 to 138)	3.9 (2.6 to 5.5)	-13.3 (-35.4 to 14.1)	3940 (3320 to 4610)	156.6 (132.1 to 182.8)	-25.3 (-37.8 to -12.1)
		Female	4 (3 to 6)	0.1 (0.1 to 0.2)	-23.7 (-38.6 to -0.5)	130 (106 to 164)	4.2 (3.5 to 5.3)	-35.6 (-48 to -15)
Romania	TBL cancer	Both	2670 (1820 to 3670)	7.8 (5.3 to 10.8)	0.4 (-21.5 to 26.5)	271000 (222000 to 330000)	819.1 (668.5 to 1000.6)	-4.2 (-21.7 to 16.6)

	Larynx cancer	Male	2040 (1380 to 2820)	13.2 (8.8 to 18.2)	-4 (-26.7 to 23)	211000 (172000 to 259000)	1382.5 (1127.1 to 1695.8)	-8.5 (-25.2 to 11.9)
		Female	626 (408 to 914)	3.3 (2.2 to 4.9)	16.9 (-15.6 to 60.7)	59100 (48000 to 71400)	333.8 (269 to 406.2)	12.8 (-8.7 to 37.6)
		Both	1020 (672 to 1440)	3.2 (2.1 to 4.6)	3.7 (-25 to 36.7)	27600 (21900 to 33500)	86.6 (68 to 105.8)	-9.9 (-29.1 to 11.3)
		Male	958 (624 to 1350)	6.4 (4.2 to 9.1)	2.4 (-26.4 to 35.9)	26100 (20700 to 31900)	173.9 (136.2 to 213)	-10.9 (-30 to 10.1)
		Female	62 (41 to 87)	0.4 (0.2 to 0.5)	5.3 (-15 to 31.6)	1450 (1130 to 1810)	8.6 (6.6 to 10.9)	-6.8 (-27.7 to 18.1)
Russia	TBL cancer	Both	13700 (9520 to 18100)	5.8 (4 to 7.7)	-12 (-25.1 to 2.3)	1330000 (1130000 to 1570000)	572.9 (484 to 673.5)	-17.2 (-30.1 to -2.7)
		Male	10700 (7200 to 14500)	11.5 (7.8 to 15.5)	-18.2 (-32.9 to -2)	1080000 (881000 to 1300000)	1138.9 (933.8 to 1367.7)	-22.2 (-36.1 to -6.4)
		Female	2980 (2010 to 4040)	2.1 (1.4 to 2.9)	8 (-14 to 34.9)	255000 (207000 to 308000)	187.9 (151.5 to 230)	0.8 (-19.8 to 24.5)
	Larynx cancer	Both	3740 (2540 to 5190)	1.6 (1.1 to 2.3)	-9.4 (-24.2 to 9)	110000 (90400 to 132000)	48.5 (39.8 to 58.1)	-25 (-38.7 to -10.2)
		Male	3440 (2330 to 4790)	3.6 (2.5 to 5)	-12.6 (-27.9 to 6)	104000 (84500 to 126000)	108.8 (88.8 to 132.2)	-27.3 (-40.9 to -11.8)
		Female	297 (201 to 433)	0.2 (0.2 to 0.4)	4.5 (-11.7 to 24.4)	6500 (5240 to 8040)	5.1 (4.1 to 6.4)	-12.3 (-29.4 to 10.3)
Rwanda	TBL cancer	Both	111 (64 to 183)	1.9 (1.1 to 3.1)	10.9 (-17.7 to 42)	12600 (8630 to 18500)	197.6 (135.8 to 286.7)	9 (-11 to 32.7)
		Male	72 (36 to 132)	3 (1.5 to 5.4)	6.3 (-26.6 to 45.9)	8290 (5020 to 13100)	314.5 (192.2 to 487.9)	3.9 (-16.8 to 25.9)
		Female	39 (25 to 58)	1.1 (0.7 to 1.7)	18.5 (-14 to 61.5)	4330 (3180 to 5640)	115.9 (86.1 to 149.5)	17.7 (-5.9 to 45.6)
	Larynx cancer	Both	46 (30 to 68)	0.7 (0.5 to 1)	-0.4 (-19 to 22.5)	2340 (1750 to 3170)	33.9 (25.9 to 45.4)	-5.3 (-23.7 to 18.2)
		Male	35 (23 to 54)	1.3 (0.8 to 1.9)	-1 (-21.3 to 25.2)	1830 (1320 to 2610)	61.7 (45.7 to 87.6)	-5.8 (-25.1 to 18.7)
		Female	11 (7 to 16)	0.3 (0.2 to 0.4)	-2.6 (-23 to 22.8)	516 (380 to 689)	13.2 (9.9 to 17.3)	-7.9 (-29.2 to 20.1)
Saint Kitts and Nevis	TBL cancer	Both	2 (1 to 3)	2.7 (1.8 to 3.7)	16 (-9.4 to 44.8)	182 (146 to 221)	253.7 (208 to 305.7)	14.5 (-4.4 to 35.5)
		Male	1 (1 to 2)	3.6 (2.4 to 5.1)	18.1 (-10.9 to 55.3)	121 (97 to 146)	343.9 (280.2 to 408.6)	18.3 (-2.8 to 41.6)
		Female	1 (0 to 1)	1.8 (1.1 to 2.6)	5.9 (-25.3 to 54.1)	60 (47 to 75)	167.9 (131.3 to 207.9)	3.2 (-16.5 to 27.1)
	Larynx cancer	Both	1 (1 to 1)	1.2 (0.8 to 1.8)	30.4 (1.7 to 65.3)	30 (24 to 36)	40.7 (33.1 to 48.5)	24.8 (4.2 to 48.3)
		Male	1 (1 to 1)	2.5 (1.6 to 3.5)	27.3 (-0.9 to 61.5)	30 (24 to 36)	81.6 (66.9 to 97.1)	21.7 (2.2 to 44.1)
		Female	0 (0 to 0)	0.1 (0 to 0.1)	-2.7 (-18.4 to 15.5)	1 (0 to 1)	1.5 (1.2 to 2)	-8.8 (-27.2 to 12)

Saint Lucia	TBL cancer	Both	6 (4 to 9)	2.8 (1.9 to 4)	15.4 (-8.9 to 48.1)	630 (528 to 749)	286.9 (241.2 to 340.1)	13.4 (-5.7 to 34.7)
		Male	4 (3 to 6)	4.1 (2.7 to 5.8)	11 (-17.2 to 49.7)	429 (355 to 514)	406.9 (339.7 to 486.6)	9.9 (-9.2 to 33.2)
		Female	2 (1 to 3)	1.8 (1.1 to 2.5)	23.2 (-7.9 to 62.1)	201 (166 to 242)	177 (146.4 to 213.7)	19.8 (-2.6 to 46.2)
	Larynx cancer	Both	3 (2 to 4)	1.4 (1 to 1.9)	12.2 (-10.8 to 41.5)	113 (94 to 136)	51 (42.3 to 60.9)	9 (-10.3 to 30.7)
		Male	3 (2 to 4)	2.6 (1.8 to 3.6)	10.6 (-13.7 to 40.4)	105 (86 to 126)	98 (81.2 to 117.3)	7.6 (-12.2 to 29.2)
		Female	0 (0 to 0)	0.3 (0.2 to 0.4)	17.2 (-0.9 to 37.7)	8 (7 to 10)	7.3 (5.9 to 8.8)	14.9 (-7.1 to 40.3)
Saint Vincent and the Grenadines	TBL cancer	Both	3 (2 to 4)	2.4 (1.7 to 3.3)	10.8 (-14.2 to 39.8)	338 (292 to 392)	244.9 (212.1 to 284.4)	10.5 (-5.9 to 29.9)
		Male	2 (1 to 3)	3 (2 to 4.2)	7.5 (-22.2 to 46.9)	220 (188 to 258)	307.5 (264 to 360.6)	7.7 (-8.8 to 28.8)
		Female	1 (1 to 2)	1.7 (1.2 to 2.4)	12.7 (-18 to 54.7)	118 (99 to 139)	178.1 (148.8 to 209.6)	12.7 (-8.1 to 36.5)
	Larynx cancer	Both	2 (2 to 3)	1.6 (1.1 to 2.2)	16.3 (-6.6 to 44.5)	97 (83 to 114)	69.6 (59.9 to 81.3)	15.2 (-3.8 to 37.5)
		Male	2 (1 to 3)	3 (2.1 to 4.1)	13.1 (-10.4 to 41.4)	93 (80 to 109)	128.5 (110.8 to 150.4)	12.5 (-6.1 to 34.7)
		Female	0 (0 to 0)	0.2 (0.1 to 0.3)	8 (-6.8 to 25.4)	4 (4 to 5)	6.6 (5.5 to 7.9)	7.7 (-9.3 to 28.5)
Samoa	TBL cancer	Both	4 (2 to 5)	2.6 (1.7 to 3.7)	1.7 (-21 to 31.3)	423 (325 to 552)	277.4 (214.3 to 354.9)	0.9 (-14.6 to 20.2)
		Male	3 (2 to 4)	3.5 (2.2 to 5.3)	-2.2 (-28 to 29.5)	286 (204 to 385)	376.1 (271.5 to 494.9)	-2.9 (-18.2 to 15)
		Female	1 (1 to 2)	1.7 (1 to 2.5)	8.1 (-23.3 to 53.4)	137 (100 to 182)	180.7 (135.1 to 238.3)	7.3 (-14.8 to 36.3)
	Larynx cancer	Both	1 (0 to 1)	0.5 (0.3 to 0.7)	-1.6 (-18.3 to 18.4)	25 (21 to 31)	17.1 (14 to 20.8)	-5.6 (-20.9 to 13.9)
		Male	1 (0 to 1)	0.7 (0.5 to 1.1)	-2.6 (-21.9 to 21.3)	18 (14 to 22)	25.1 (20.3 to 30.9)	-6.6 (-22.1 to 13.8)
		Female	0 (0 to 0)	0.3 (0.2 to 0.4)	-1.5 (-23.9 to 27.1)	8 (6 to 10)	9.8 (7.2 to 13.2)	-5.4 (-28.9 to 26.2)
San Marino	TBL cancer	Both	5 (4 to 8)	9.2 (5.9 to 13.5)	0.1 (-21.2 to 28.6)	412 (264 to 607)	721.9 (456.3 to 1079.2)	-2.8 (-30.2 to 34.5)
		Male	4 (3 to 6)	13.4 (8.6 to 19.5)	-2.3 (-24.7 to 26.9)	290 (184 to 418)	1043.4 (655.9 to 1521)	-4.4 (-31.6 to 30.7)
		Female	2 (1 to 2)	5.2 (3.2 to 8.2)	4 (-26.7 to 47.6)	121 (77 to 191)	421.4 (263.1 to 665.3)	0.1 (-31.5 to 45.2)
	Larynx cancer	Both	1 (1 to 2)	2.4 (1.5 to 3.7)	5.1 (-23.5 to 40.3)	18 (11 to 26)	31.8 (19.5 to 47.6)	0.4 (-31.3 to 40.3)
		Male	1 (1 to 2)	4.9 (3.1 to 7.3)	6.1 (-23 to 42.1)	17 (11 to 25)	63.5 (39 to 95.2)	1.7 (-29.9 to 42.2)
		Female	0 (0 to 0)	0.1 (0.1 to 0.2)	0.5 (-22.1 to 29.1)	1 (0 to 1)	2.1 (1.3 to 3.4)	-4.5 (-37.9 to 42)
Sao Tome and Principe	TBL cancer	Both	4 (3 to 6)	4.2 (2.7 to 6)	11.6 (-13.4 to 41.6)	499 (381 to 637)	448 (347.2 to 563.6)	10.5 (-8.2 to 29.7)
		Male	3 (2 to 4)	6.1 (4 to 8.6)	8.3 (-18.7 to 46.8)	337 (266 to 427)	646.7 (520.9 to 798.2)	7.1 (-11.7 to 29)
		Female	1 (1 to 2)	2.5 (1.2 to 4)	18.8 (-16.7 to 63.7)	162 (93 to 236)	266 (151.3 to 382.6)	17.4 (-7.7 to 43)
	Larynx cancer	Both	1 (0 to 1)	0.6 (0.4 to 0.8)	9.6 (-13.6 to 36.2)	28 (22 to 35)	25.5 (20.1 to 32)	5.5 (-15.4 to 30.1)
		Male	1 (0 to 1)	1.1 (0.8 to 1.6)	9.7 (-14.6 to 38)	25 (20 to 32)	49.1 (38.7 to 61.2)	5.6 (-15.9 to 30.6)
		Female	0 (0 to 0)	0.1 (0.1 to 0.1)	1.4 (-19.4 to 27.2)	2 (2 to 3)	3.9 (2.9 to 5.2)	-4.6 (-25 to 21.1)

Saudi Arabia	TBL cancer	Both	382 (252 to 554)	2 (1.3 to 2.9)	-3.5 (-27 to 27.2)	45100 (34200 to 56600)	206 (161.1 to 250.4)	-5.3 (-23.9 to 15.3)
		Male	269 (170 to 397)	2.5 (1.5 to 3.7)	-9.7 (-36.2 to 25.6)	32600 (24300 to 41500)	258 (197.9 to 321.4)	-9.9 (-28.9 to 12)
		Female	113 (73 to 162)	1.3 (0.9 to 1.9)	8.7 (-19.8 to 43.9)	12500 (9260 to 16300)	125.9 (94.8 to 161.2)	3.2 (-20.3 to 31.1)
	Larynx cancer	Both	138 (91 to 202)	0.6 (0.4 to 0.9)	2.2 (-18.6 to 27.1)	3630 (2690 to 4910)	15.4 (12 to 20.3)	-19.2 (-35.8 to -0.1)
		Male	117 (77 to 175)	0.9 (0.6 to 1.3)	1.1 (-20.8 to 28.5)	3130 (2290 to 4280)	22.4 (17.6 to 29.7)	-19.5 (-36.4 to 0.3)
		Female	21 (13 to 31)	0.2 (0.1 to 0.3)	-6.6 (-28 to 19.8)	498 (351 to 686)	4.5 (3.3 to 6.1)	-28 (-45.5 to -4.4)
Senegal	TBL cancer	Both	200 (125 to 294)	2.7 (1.7 to 4)	9.2 (-19.6 to 46)	22300 (16400 to 29500)	288.3 (215.8 to 378.8)	9.4 (-13.4 to 35)
		Male	148 (91 to 230)	4.2 (2.6 to 6.5)	6.5 (-24.3 to 46.7)	16600 (12000 to 22800)	448.2 (328.8 to 610.6)	6.5 (-16.1 to 34.9)
		Female	52 (32 to 79)	1.3 (0.8 to 2)	20.4 (-17.7 to 73.2)	5730 (4240 to 7740)	139.3 (104.9 to 185.7)	22 (-3.3 to 53.8)
	Larynx cancer	Both	45 (29 to 65)	0.6 (0.4 to 0.9)	5.1 (-18.4 to 37.7)	2190 (1620 to 2880)	27.9 (21.1 to 36.5)	2.2 (-22.5 to 32.2)
		Male	40 (26 to 59)	1.1 (0.7 to 1.6)	6.2 (-18.8 to 41)	1960 (1420 to 2600)	52 (38.5 to 68.4)	3.3 (-21.8 to 34.8)
		Female	5 (3 to 7)	0.1 (0.1 to 0.2)	5.2 (-21.1 to 37.3)	228 (165 to 301)	5.5 (4 to 7.2)	1.9 (-25 to 33.6)
Serbia	TBL cancer	Both	1770 (1170 to 2490)	11.5 (7.6 to 16.3)	-3.8 (-26.8 to 25.5)	175000 (136000 to 221000)	1172 (910.3 to 1482.4)	-6.3 (-27.1 to 18.9)
		Male	1250 (809 to 1780)	17.3 (11.2 to 24.7)	-8.1 (-31.2 to 22.3)	127000 (98600 to 161000)	1782 (1380.8 to 2275.3)	-10.1 (-30.1 to 14.4)
		Female	518 (332 to 745)	6.5 (4.2 to 9.5)	6.7 (-22.8 to 46.6)	48200 (37300 to 61700)	627.9 (483.4 to 810.5)	3.7 (-19.9 to 34)
	Larynx cancer	Both	544 (351 to 785)	3.7 (2.4 to 5.4)	-3.4 (-28.8 to 30.2)	11100 (8490 to 14300)	75.4 (57.3 to 96.9)	-15.4 (-34.7 to 9.2)
		Male	471 (303 to 681)	6.7 (4.3 to 9.7)	-4.5 (-30.8 to 29.5)	10100 (7660 to 13000)	144.8 (109.3 to 186.8)	-16.1 (-35.7 to 8.7)
		Female	73 (47 to 105)	0.9 (0.6 to 1.3)	-0.4 (-25.1 to 32.4)	997 (745 to 1290)	12.8 (9.6 to 16.5)	-14.7 (-37 to 12.6)
Seychelles	TBL cancer	Both	4 (3 to 6)	3.9 (2.7 to 5.2)	4.1 (-15.3 to 29.1)	472 (410 to 549)	413 (360.2 to 479.5)	3.2 (-9.9 to 20.2)
		Male	3 (2 to 4)	6.2 (4.1 to 8.4)	-1.7 (-23.9 to 26.9)	364 (308 to 439)	655.2 (556.3 to 783.6)	-1.9 (-17.1 to 18.1)
		Female	1 (1 to 2)	1.9 (1.2 to 2.7)	9.7 (-20.5 to 47.5)	108 (88 to 130)	187.5 (154.1 to 226.3)	8.4 (-12.4 to 31.1)
	Larynx cancer	Both	4 (3 to 6)	3.8 (2.6 to 5.2)	10.2 (-10.8 to 36.9)	146 (122 to 175)	123.7 (103 to 147.8)	0.6 (-17.7 to 21.6)
		Male	4 (3 to 6)	7.3 (5 to 10)	5.8 (-15.7 to 33)	137 (112 to 167)	237.8 (196.8 to 287)	-3.2 (-20.7 to 17.6)
		Female	0 (0 to 0)	0.5 (0.4 to 0.7)	8.7 (-12.6 to 32.9)	9 (7 to 11)	15.3 (12.2 to 18.7)	1.7 (-18.4 to 27.3)
Sierra Leone	TBL cancer	Both	83 (50 to 125)	2.4 (1.4 to 3.5)	6.9 (-20.7 to 45.9)	9420 (6630 to 12800)	251.5 (178.4 to 339.1)	6.6 (-14.7 to 35.4)

		Male	61 (35 to 96)	3.5 (2 to 5.5)	1.6 (-28.8 to 44.4)	6980 (4680 to 9680)	376.8 (256.1 to 517.8)	1.3 (-19.8 to 30.3)
		Female	22 (14 to 32)	1.2 (0.8 to 1.8)	19.6 (-15.5 to 65.2)	2450 (1790 to 3220)	125 (92.7 to 165.1)	18.6 (-5.6 to 49.1)
	Larynx cancer	Both	23 (15 to 35)	0.6 (0.4 to 1)	0.3 (-21.1 to 29.4)	1170 (856 to 1600)	31.1 (23 to 42.1)	-4.2 (-24.8 to 22)
		Male	21 (13 to 31)	1.1 (0.7 to 1.7)	-1.2 (-23 to 29.8)	1040 (747 to 1430)	54.8 (39.8 to 74.9)	-5.7 (-26.7 to 21.3)
		Female	3 (2 to 4)	0.1 (0.1 to 0.2)	-4.4 (-25.4 to 22.5)	130 (92 to 181)	6.7 (4.8 to 9.3)	-8.9 (-30.1 to 19.7)
Singapore	TBL cancer	Both	610 (414 to 850)	7.9 (5.4 to 11)	-11.4 (-31 to 13.6)	31400 (29000 to 33500)	398.4 (366.2 to 425.6)	-20.2 (-25.2 to -15.1)
		Male	383 (249 to 539)	10.5 (7.1 to 14.8)	-17.4 (-37.7 to 7.3)	20500 (18700 to 22000)	538.8 (490.2 to 578.2)	-25.2 (-31.7 to -19.6)
		Female	227 (153 to 322)	5.7 (3.9 to 8.1)	-3.6 (-28.4 to 29.9)	10900 (9560 to 12100)	271.4 (238.4 to 300.8)	-13.3 (-21.7 to -4)
	Larynx cancer	Both	102 (67 to 148)	1.3 (0.9 to 1.9)	-19.6 (-39 to 5.1)	696 (616 to 790)	8.8 (7.8 to 10)	-30.4 (-38.2 to -20.4)
		Male	88 (58 to 129)	2.3 (1.5 to 3.3)	-24.1 (-43.7 to 1.5)	618 (539 to 709)	16.3 (14.3 to 18.6)	-34.1 (-42 to -23.5)
		Female	13 (9 to 19)	0.3 (0.2 to 0.5)	-2.6 (-21.2 to 18.8)	78 (66 to 95)	2 (1.7 to 2.4)	-15.5 (-26.7 to -2.3)
Slovakia	TBL cancer	Both	788 (509 to 1130)	8.5 (5.5 to 12.2)	-2.8 (-27.3 to 30.8)	58600 (45100 to 76300)	641.4 (491.6 to 833.8)	-9.9 (-30.7 to 17.1)
		Male	591 (376 to 865)	14.7 (9.3 to 21.1)	-6.3 (-31.7 to 26)	45000 (34200 to 59100)	1096.1 (834.6 to 1426.6)	-13.5 (-34.1 to 13.5)
		Female	197 (126 to 290)	3.9 (2.5 to 5.6)	0.3 (-30.8 to 40.5)	13600 (10500 to 17200)	278 (213.3 to 353)	-5.4 (-27.8 to 21.6)
	Larynx cancer	Both	171 (111 to 249)	1.9 (1.3 to 2.8)	-6.4 (-33 to 30.2)	4670 (3490 to 6160)	53.2 (39.5 to 70.2)	-17.3 (-38.3 to 10.5)
		Male	158 (102 to 232)	3.9 (2.5 to 5.7)	-9.4 (-35.7 to 26.7)	4430 (3290 to 5870)	108.5 (80.4 to 142.7)	-19.6 (-40 to 7.4)
		Female	13 (8 to 19)	0.3 (0.2 to 0.4)	-0.3 (-24 to 30.7)	237 (173 to 314)	5.2 (3.7 to 6.9)	-12.7 (-36.2 to 16.6)
Slovenia	TBL cancer	Both	332 (217 to 468)	8.3 (5.4 to 11.7)	-9.8 (-33.7 to 19)	27300 (20900 to 35600)	697.1 (531.5 to 906.7)	-12.3 (-32.7 to 14.7)
		Male	229 (147 to 324)	12.2 (7.9 to 17.3)	-15.2 (-39.1 to 11.7)	19400 (14800 to 25600)	1041.9 (793.7 to 1377.1)	-17.5 (-36.7 to 6.5)
		Female	104 (66 to 150)	4.9 (3.1 to 7.2)	-2 (-30.4 to 37.3)	7900 (5940 to 10300)	392 (292.2 to 514.7)	-3.8 (-28.1 to 28.4)
	Larynx cancer	Both	74 (47 to 108)	2 (1.2 to 2.9)	-4.4 (-30.3 to 31.7)	1230 (929 to 1640)	32.6 (24.8 to 43.5)	-18.7 (-38.7 to 9.2)
		Male	65 (42 to 97)	3.6 (2.3 to 5.3)	-7.7 (-34.2 to 28.1)	1110 (832 to 1480)	61.4 (46.5 to 81.8)	-21.3 (-40.1 to 6.5)
		Female	8 (5 to 13)	0.4 (0.3 to 0.7)	1.3 (-26.6 to 38)	123 (86 to 167)	6.2 (4.4 to 8.6)	-12 (-35.9 to 19.8)
Solomon Islands	TBL cancer	Both	24 (11 to 41)	7.2 (3.6 to 12.3)	6.6 (-16.2 to 38.6)	3060 (1540 to 5110)	852 (456.7 to 1377.7)	6.3 (-11.9 to 31.2)

		Male	19 (8 to 37)	11.5 (5.3 to 21.4)	5.6 (-18.1 to 40)	2490 (1170 to 4390)	1363.3 (678.6 to 2360.3)	5 (-13 to 30.2)
		Female	4 (2 to 7)	2.8 (1.6 to 4.4)	21.9 (-12.5 to 69.4)	569 (355 to 864)	321.7 (209.8 to 477.5)	22.3 (-1.9 to 51.4)
	Larynx cancer	Both	3 (2 to 4)	0.8 (0.5 to 1.1)	3.7 (-16 to 27.1)	124 (76 to 167)	34.8 (22.7 to 45.6)	-2.5 (-20.6 to 19.6)
		Male	2 (1 to 3)	1.2 (0.7 to 1.7)	4.8 (-17.4 to 32)	92 (48 to 127)	51.5 (29.7 to 68.1)	-2 (-21.4 to 22.7)
		Female	1 (0 to 1)	0.4 (0.2 to 0.6)	7 (-17.7 to 40)	32 (20 to 45)	17.5 (11.4 to 23.3)	1.7 (-22.4 to 36.1)
Somalia	TBL cancer	Both	88 (42 to 160)	1.3 (0.6 to 2.4)	-3.7 (-29.2 to 30.3)	10800 (6060 to 18100)	144.9 (82.1 to 238)	-5.3 (-24.6 to 17.3)
		Male	69 (32 to 132)	2.5 (1.2 to 4.7)	-4.7 (-32.4 to 37.4)	8550 (4690 to 15000)	274.2 (151.4 to 470.8)	-6.5 (-24.7 to 15.6)
		Female	19 (9 to 34)	0.5 (0.2 to 0.9)	2 (-22 to 29.2)	2210 (1210 to 3890)	51.8 (28.3 to 89.6)	1.3 (-22.7 to 28.1)
	Larynx cancer	Both	51 (30 to 78)	0.7 (0.4 to 1)	-7 (-25 to 16.4)	3090 (2120 to 4380)	37.8 (26.4 to 53)	-8.4 (-26.5 to 16.2)
		Male	37 (22 to 58)	1.1 (0.7 to 1.7)	-7.8 (-27.5 to 19.4)	2320 (1490 to 3290)	64.9 (42.5 to 90.8)	-9.3 (-28.1 to 16.1)
		Female	13 (8 to 21)	0.3 (0.2 to 0.5)	-3.9 (-25.5 to 24.6)	769 (496 to 1150)	16.8 (11 to 25.2)	-5.9 (-28.2 to 25.1)
South Africa	TBL cancer	Both	1920 (1340 to 2570)	4.2 (3 to 5.6)	-12.4 (-23.4 to 2.2)	220000 (196000 to 254000)	467.3 (417.7 to 537.5)	-16 (-25.6 to -3.1)
		Male	1320 (917 to 1790)	6.8 (4.8 to 9.2)	-15.5 (-28.1 to 0.7)	155000 (136000 to 183000)	760.9 (665.9 to 893.5)	-18.8 (-29.2 to -3.3)
		Female	604 (422 to 807)	2.4 (1.6 to 3.1)	-7.1 (-21 to 10.6)	64200 (55800 to 74000)	245 (213.4 to 281.7)	-10.6 (-22.4 to 3.9)
	Larynx cancer	Both	390 (280 to 520)	0.8 (0.6 to 1.1)	-19.5 (-27.3 to -9.9)	16700 (15000 to 18500)	34.6 (31.2 to 38.4)	-29.5 (-35.8 to -20.9)
		Male	326 (233 to 434)	1.6 (1.2 to 2.2)	-20.1 (-28.5 to -8.9)	14300 (12800 to 16100)	68.8 (61.8 to 77.6)	-29.9 (-36.8 to -20.4)
		Female	65 (45 to 87)	0.2 (0.2 to 0.3)	-18.4 (-27.3 to -8.3)	2380 (2110 to 2730)	8.8 (7.8 to 10.1)	-29.4 (-38.8 to -19)
South Sudan	TBL cancer	Both	73 (41 to 115)	2 (1.1 to 3)	-4.7 (-29.3 to 26.4)	8570 (5450 to 12600)	213 (137.3 to 305.7)	-4.8 (-23.3 to 18)
		Male	56 (29 to 89)	2.9 (1.5 to 4.6)	-4.3 (-33.3 to 32.4)	6510 (3820 to 9570)	312.7 (188.1 to 453.9)	-4.9 (-23.8 to 17.5)
		Female	17 (10 to 33)	0.9 (0.5 to 1.7)	4.9 (-20.6 to 42.2)	2060 (1260 to 3760)	99 (62.9 to 181.1)	8 (-15.2 to 41.5)
	Larynx cancer	Both	19 (12 to 30)	0.5 (0.3 to 0.7)	-8.2 (-26.2 to 14.7)	1080 (711 to 1600)	24.2 (16.4 to 35.4)	-10.2 (-29 to 14.9)
		Male	15 (9 to 25)	0.7 (0.4 to 1.1)	-6.4 (-25.9 to 20.5)	847 (544 to 1330)	36.7 (24 to 57.3)	-8.2 (-28.2 to 18.8)
		Female	4 (3 to 6)	0.2 (0.1 to 0.3)	-4.9 (-24.9 to 22)	229 (147 to 336)	10.2 (6.9 to 14.7)	-6.3 (-28.6 to 25.6)
Spain	TBL cancer	Both	7500 (4930 to 10700)	8.7 (5.6 to 12.5)	-6.8 (-31.4 to 24)	517000 (485000 to 545000)	624.8 (587.7 to 659.2)	-10.1 (-15.2 to -5.1)

		Male	5980 (3920 to 8600)	14.8 (9.7 to 21.3)	-11.1 (-34.7 to 18.7)	405000 (377000 to 430000)	1030.4 (959.8 to 1095.2)	-14.7 (-20.3 to -9.3)
		Female	1510 (965 to 2250)	3.4 (2.2 to 5.1)	11.2 (-23.5 to 60.4)	112000 (100000 to 124000)	269.8 (242.7 to 297.5)	8.9 (-1.6 to 20.7)
	Larynx cancer	Both	2260 (1470 to 3330)	2.8 (1.8 to 4.1)	-16.6 (-38.5 to 12.4)	32100 (29200 to 35600)	39 (35.4 to 43.1)	-22 (-30 to -12.9)
		Male	2130 (1380 to 3150)	5.5 (3.6 to 8.2)	-17.7 (-39.6 to 11.6)	30200 (27300 to 33500)	77.4 (69.8 to 86.1)	-23 (-31.3 to -13.6)
		Female	132 (83 to 202)	0.3 (0.2 to 0.5)	-0.2 (-22.3 to 29)	1930 (1440 to 2250)	4.6 (3.5 to 5.4)	-7.1 (-19.9 to 7.7)
Sri Lanka	TBL cancer	Both	594 (358 to 902)	2.3 (1.4 to 3.5)	-3 (-31.2 to 37.4)	60500 (43600 to 82900)	229.1 (165.9 to 312.2)	-6.1 (-31.9 to 26.9)
		Male	407 (239 to 632)	3.5 (2 to 5.3)	-7.9 (-39.7 to 38.4)	42500 (29400 to 60000)	352.5 (246 to 494.2)	-10.4 (-37.1 to 25.3)
		Female	188 (115 to 280)	1.3 (0.8 to 2)	11.7 (-22.9 to 57.4)	18100 (13100 to 24000)	127.3 (92.9 to 168.5)	7.9 (-21.2 to 42.6)
	Larynx cancer	Both	180 (111 to 267)	0.7 (0.4 to 1)	-4.3 (-31.2 to 28.6)	4350 (3070 to 5930)	16.4 (11.7 to 22.3)	-21.6 (-43.3 to 5.2)
		Male	147 (87 to 222)	1.2 (0.7 to 1.8)	-5.3 (-32.7 to 29.5)	3510 (2430 to 4890)	29.2 (20.4 to 40.4)	-23 (-45.2 to 3.7)
		Female	33 (21 to 49)	0.2 (0.1 to 0.3)	5.3 (-25.5 to 40.4)	838 (598 to 1140)	5.9 (4.2 to 8)	-11.1 (-37 to 19.3)
Sudan	TBL cancer	Both	364 (211 to 611)	1.9 (1.1 to 3.3)	2.7 (-25.1 to 42)	41100 (26600 to 63700)	201.9 (130.7 to 314.8)	1.9 (-18.5 to 29.4)
		Male	263 (139 to 488)	2.6 (1.4 to 4.9)	-2.4 (-33.2 to 46.1)	29600 (17700 to 50800)	276.8 (167.3 to 474.9)	-3.3 (-23.7 to 26.8)
		Female	101 (60 to 153)	1.1 (0.7 to 1.6)	21.2 (-11.6 to 60.9)	11500 (7710 to 16600)	111.6 (78.2 to 154)	22.3 (-7.5 to 58.8)
	Larynx cancer	Both	212 (129 to 316)	1.1 (0.7 to 1.6)	3 (-16.2 to 30.9)	9360 (6230 to 13000)	45.4 (31.1 to 61.7)	-6.6 (-24.1 to 16.1)
		Male	166 (94 to 252)	1.6 (0.9 to 2.4)	2.5 (-18.6 to 33.3)	7370 (4760 to 10300)	67.9 (45.3 to 93.7)	-6.9 (-24.9 to 18.2)
		Female	46 (27 to 73)	0.5 (0.3 to 0.7)	5.9 (-17.3 to 37.6)	1990 (1290 to 2950)	18.8 (12.5 to 26.8)	-4 (-25.7 to 25.4)
Suriname	TBL cancer	Both	21 (14 to 29)	3.4 (2.3 to 4.8)	7.6 (-18.9 to 38.3)	2290 (1860 to 2780)	367.1 (299.5 to 446)	7.4 (-12.2 to 31.3)
		Male	14 (9 to 19)	4.8 (3.1 to 6.9)	4.8 (-24.8 to 42)	1500 (1200 to 1850)	510.8 (412.8 to 632.2)	4.3 (-16.1 to 30.3)
		Female	7 (5 to 11)	2.3 (1.5 to 3.3)	14.8 (-18.7 to 62.9)	793 (635 to 974)	241.7 (193 to 294.7)	15.1 (-9.4 to 43.9)
	Larynx cancer	Both	3 (2 to 4)	0.5 (0.3 to 0.7)	6.4 (-16.9 to 36.5)	135 (107 to 167)	21.3 (17 to 26.3)	2.3 (-19.1 to 28.3)
		Male	3 (2 to 4)	0.9 (0.6 to 1.3)	6.4 (-18.9 to 39.5)	116 (91 to 147)	38.9 (30.7 to 48.6)	2.2 (-20.2 to 29.3)
		Female	1 (0 to 1)	0.2 (0.1 to 0.2)	10.8 (-9.3 to 38.4)	19 (15 to 24)	5.7 (4.6 to 7.1)	7.4 (-13.9 to 38.5)
Sweden	TBL cancer	Both	978 (686 to 1320)	4.8 (3.4 to 6.5)	-6.6 (-24.2 to 11.7)	80200 (75300 to 84400)	411.3 (388 to 431.9)	-11 (-15.2 to -6.7)

		Male	468 (312 to 656)	4.8 (3.2 to 6.6)	-14.2 (-36.4 to 12.9)	39500 (37000 to 41900)	412.7 (388.3 to 437.2)	-16.8 (-21.6 to -11.7)
		Female	510 (335 to 726)	4.9 (3.3 to 7.1)	0.7 (-23.8 to 33.6)	40700 (37500 to 43600)	413.5 (384.7 to 442.2)	-5.4 (-10.9 to 0.3)
	Larynx cancer	Both	158 (110 to 220)	0.9 (0.6 to 1.2)	-2.9 (-22.6 to 20.8)	1340 (1240 to 1470)	7.2 (6.7 to 7.9)	-10.4 (-17.8 to -1.3)
		Male	136 (94 to 191)	1.5 (1 to 2.1)	-4.8 (-27.6 to 21.9)	1070 (971 to 1190)	11.7 (10.6 to 13)	-12.2 (-20.9 to -1.9)
		Female	22 (15 to 32)	0.3 (0.2 to 0.4)	-0.2 (-16.6 to 19.1)	274 (241 to 310)	3 (2.7 to 3.4)	-8 (-17.6 to 4.7)
Switzerland	TBL cancer	Both	1070 (704 to 1530)	6.6 (4.3 to 9.4)	-14.2 (-35 to 14.7)	76800 (71300 to 81500)	488.2 (454.6 to 517.6)	-17.7 (-22.5 to -13.1)
		Male	640 (410 to 923)	8.3 (5.3 to 12)	-19.4 (-42 to 9.2)	47200 (43600 to 50900)	623.3 (575.5 to 670.1)	-22.3 (-28.1 to -16.5)
		Female	430 (285 to 623)	5.1 (3.3 to 7.5)	-7.3 (-35.2 to 26.6)	29500 (26900 to 32000)	367.9 (338 to 398.1)	-11.3 (-18.5 to -3.9)
	Larynx cancer	Both	194 (127 to 285)	1.3 (0.8 to 1.9)	-14.8 (-36.1 to 13.3)	2100 (1890 to 2350)	13.6 (12.3 to 15.2)	-19.7 (-28.5 to -9.6)
		Male	168 (108 to 246)	2.2 (1.5 to 3.3)	-17 (-39.2 to 11.1)	1760 (1560 to 1970)	23.6 (20.9 to 26.5)	-22 (-32.2 to -11.8)
		Female	27 (18 to 39)	0.4 (0.2 to 0.5)	-8.5 (-27.3 to 15.6)	346 (297 to 410)	4.4 (3.8 to 5.2)	-13.3 (-25.7 to 1.1)
Syria	TBL cancer	Both	327 (201 to 485)	2.6 (1.6 to 3.7)	-5.3 (-33.1 to 31.3)	36600 (26700 to 49100)	271.8 (201.1 to 360.7)	-5.4 (-29.3 to 24.1)
		Male	233 (140 to 355)	3.5 (2.1 to 5.3)	-7 (-38.4 to 36.3)	26600 (19200 to 35700)	385.8 (280.8 to 512)	-6.9 (-32.3 to 25.1)
		Female	94 (57 to 138)	1.6 (0.9 to 2.2)	3.4 (-30.5 to 44)	10100 (6890 to 13900)	153.7 (105.3 to 208.6)	4.1 (-25.2 to 39.7)
	Larynx cancer	Both	87 (56 to 130)	0.7 (0.4 to 1)	-0.5 (-23.8 to 29.9)	2610 (1920 to 3480)	19.7 (14.6 to 26)	-5.6 (-29.9 to 24.5)
		Male	68 (43 to 103)	1 (0.7 to 1.5)	1 (-25.4 to 39)	2210 (1620 to 2970)	32.5 (24.1 to 43.6)	-3.4 (-29.2 to 29.7)
		Female	19 (13 to 27)	0.3 (0.2 to 0.4)	1.3 (-17.4 to 24.6)	400 (293 to 543)	6.2 (4.6 to 8.2)	-3.9 (-28.5 to 29.6)
Taiwan (Province of China)	TBL cancer	Both	2970 (1980 to 4190)	7.5 (5.1 to 10.6)	-8.8 (-30.3 to 20.9)	247000 (193000 to 319000)	631.2 (495.1 to 815.9)	-3.6 (-24.9 to 24.2)
		Male	1830 (1190 to 2620)	10 (6.5 to 14.2)	-9 (-32.7 to 22.7)	154000 (121000 to 202000)	837.3 (657.6 to 1090.6)	-4.7 (-26.1 to 23.8)
		Female	1140 (747 to 1630)	5.4 (3.6 to 7.8)	-4.9 (-31.4 to 28.4)	92800 (72400 to 120000)	451.8 (351.3 to 585.9)	1.3 (-21 to 31.1)
	Larynx cancer	Both	479 (305 to 704)	1.2 (0.8 to 1.8)	2.5 (-23.6 to 37.5)	6320 (4830 to 8400)	16.2 (12.4 to 21.5)	-6.1 (-28.7 to 25.1)
		Male	453 (288 to 669)	2.4 (1.5 to 3.6)	5.3 (-21.8 to 42.3)	5940 (4510 to 7920)	32.1 (24.5 to 42.8)	-3.5 (-26.8 to 28.4)
		Female	26 (16 to 38)	0.1 (0.1 to 0.2)	0 (-24 to 31.2)	384 (290 to 519)	1.9 (1.5 to 2.6)	-7.1 (-30 to 20.8)

Tajikistan	TBL cancer	Both	143 (93 to 207)	2.8 (1.8 to 3.9)	-7.4 (-32.3 to 24.2)	17200 (13600 to 21600)	297.5 (241.9 to 370.1)	-10.5 (-29.6 to 13.9)
		Male	98 (61 to 149)	4 (2.6 to 5.9)	-10.1 (-38.5 to 27.3)	11700 (9240 to 15000)	432.7 (343.8 to 548.1)	-13.7 (-33.5 to 11)
		Female	45 (29 to 66)	1.6 (1 to 2.3)	5.4 (-26.5 to 54.2)	5430 (4290 to 6830)	173.7 (139.3 to 217.8)	3.4 (-19.5 to 32.9)
	Larynx cancer	Both	33 (22 to 50)	0.6 (0.4 to 0.9)	-22.9 (-39.6 to -2)	1460 (1110 to 2040)	24.2 (18.7 to 32.6)	-29 (-46.1 to -7.2)
		Male	21 (13 to 33)	0.8 (0.5 to 1.2)	-24.5 (-42.1 to -1.1)	918 (679 to 1340)	33.6 (25.4 to 49.9)	-31.1 (-48.5 to -7.5)
		Female	13 (8 to 18)	0.4 (0.3 to 0.6)	-15.3 (-34.4 to 7.7)	538 (401 to 710)	15.8 (12.1 to 20.5)	-20.3 (-41.8 to 5.3)
Thailand	TBL cancer	Both	5130 (3160 to 7510)	5 (3.1 to 7.3)	-8.8 (-36.7 to 25.7)	519000 (387000 to 692000)	507.9 (379.4 to 674)	-10.9 (-34.7 to 20.2)
		Male	3330 (2000 to 4970)	7.2 (4.3 to 10.6)	-9.7 (-38.1 to 32)	345000 (252000 to 462000)	728.6 (534.2 to 969.5)	-11.4 (-36.2 to 22)
		Female	1790 (1090 to 2670)	3.2 (2 to 4.8)	-7.1 (-38.3 to 32.4)	174000 (130000 to 233000)	318.4 (236.3 to 423.5)	-10.1 (-35.7 to 20.8)
	Larynx cancer	Both	1150 (724 to 1730)	1.1 (0.7 to 1.7)	4.5 (-24.9 to 46.2)	29000 (20800 to 40000)	28 (20.2 to 38.3)	-8.8 (-36.4 to 26.4)
		Male	1050 (651 to 1590)	2.2 (1.4 to 3.3)	6.4 (-25.1 to 51.8)	26600 (18900 to 36900)	55.1 (39.5 to 75.3)	-7.2 (-35.7 to 28.4)
		Female	104 (69 to 152)	0.2 (0.1 to 0.3)	-11.1 (-32.8 to 17.3)	2380 (1730 to 3180)	4.4 (3.1 to 5.9)	-24.1 (-47.4 to 5.4)
Timor-Leste	TBL cancer	Both	35 (22 to 52)	4.3 (2.7 to 6.2)	11.7 (-16.6 to 52.4)	4070 (2860 to 5350)	475.2 (338 to 623.9)	13.9 (-11.1 to 44.9)
		Male	25 (14 to 38)	6.1 (3.5 to 9.2)	10.8 (-22.3 to 55.8)	2870 (1880 to 3870)	676.7 (452.2 to 896.9)	12.7 (-12.9 to 45.4)
		Female	11 (6 to 17)	2.5 (1.5 to 4)	16.7 (-19.6 to 71.4)	1200 (832 to 1720)	277.7 (194.7 to 396.3)	19.1 (-9.8 to 53.9)
	Larynx cancer	Both	5 (3 to 7)	0.6 (0.3 to 0.8)	23.6 (-1.3 to 53.8)	200 (134 to 274)	23.7 (16 to 32.1)	20.4 (-4.8 to 50.9)
		Male	4 (2 to 6)	0.9 (0.5 to 1.4)	29.3 (0 to 67.2)	157 (97 to 225)	37.4 (23.5 to 53.5)	25.1 (-2.8 to 61.9)
		Female	1 (1 to 2)	0.3 (0.2 to 0.4)	9.9 (-11.2 to 35)	43 (30 to 55)	10.1 (7.1 to 12.9)	7.7 (-14.8 to 38)
Togo	TBL cancer	Both	92 (59 to 138)	2.5 (1.6 to 3.8)	0.9 (-24.5 to 34.8)	10700 (7660 to 14400)	271 (199 to 360.5)	-0.3 (-19.3 to 22.5)
		Male	64 (39 to 100)	4.3 (2.6 to 6.5)	-3 (-29.9 to 34.3)	7510 (5170 to 10500)	450 (319 to 620.1)	-5 (-22.9 to 17.1)
		Female	28 (17 to 41)	1.3 (0.8 to 2)	14.2 (-18.7 to 55.7)	3190 (2260 to 4180)	141.3 (100.8 to 183.2)	15.1 (-10.2 to 46.1)
	Larynx cancer	Both	26 (16 to 39)	0.7 (0.4 to 1)	-3.8 (-24.5 to 25)	1350 (973 to 1810)	33 (24.1 to 44.3)	-7.8 (-26.2 to 18.9)
		Male	24 (15 to 36)	1.4 (0.9 to 2.1)	-3.6 (-26.4 to 26.1)	1220 (864 to 1660)	69.1 (50.3 to 93)	-8 (-26.3 to 19.1)
		Female	3 (2 to 4)	0.1 (0.1 to 0.2)	-2.1 (-23.9 to 26.4)	127 (92 to 174)	5.6 (4.1 to 7.5)	-6.9 (-28.4 to 24.1)

Tokelau	TBL cancer	Both	0 (0 to 0)	5.4 (3.5 to 7.9)	3.1 (-19.6 to 30.4)	8 (6 to 10)	577.5 (444.3 to 754.9)	1.9 (-15 to 23.6)
		Male	0 (0 to 0)	6.8 (4.2 to 10.5)	-3 (-25.7 to 27)	5 (4 to 7)	718.9 (526.6 to 1014.7)	-3.9 (-21.1 to 17)
		Female	0 (0 to 0)	4 (2.3 to 6.2)	9.4 (-23.1 to 51.6)	3 (2 to 4)	435.9 (285.9 to 615)	8.6 (-13.4 to 37.1)
	Larynx cancer	Both	0 (0 to 0)	0.4 (0.3 to 0.6)	-1.2 (-17.4 to 18.9)	0 (0 to 0)	13.9 (10.8 to 17.7)	-10.7 (-26.4 to 6.7)
		Male	0 (0 to 0)	0.6 (0.4 to 0.8)	-3.8 (-22.9 to 21.7)	0 (0 to 0)	18.6 (14.3 to 23.8)	-12.4 (-30.3 to 8.7)
		Female	0 (0 to 0)	0.3 (0.2 to 0.4)	-3.7 (-25.1 to 23.8)	0 (0 to 0)	9.4 (6.7 to 12.8)	-12 (-34.1 to 16.3)
Tonga	TBL cancer	Both	5 (3 to 7)	6.1 (4 to 8.5)	0 (-18.7 to 25.2)	523 (421 to 641)	658 (532.6 to 804.4)	-0.4 (-16.5 to 18.6)
		Male	4 (2 to 5)	10.1 (6.8 to 14.1)	-2.4 (-22.8 to 25.9)	402 (321 to 498)	1080 (870.1 to 1335)	-2.8 (-18.6 to 16.9)
		Female	1 (1 to 2)	2.7 (1.7 to 4.1)	10.5 (-21.1 to 58)	121 (90 to 158)	288.9 (216 to 376.9)	10.8 (-13.3 to 41.9)
	Larynx cancer	Both	0 (0 to 0)	0.4 (0.3 to 0.6)	-1.8 (-19 to 19.1)	11 (8 to 14)	14.1 (10.8 to 17.8)	-6.9 (-23 to 12.3)
		Male	0 (0 to 0)	0.7 (0.5 to 1)	-1.7 (-19.7 to 22.4)	9 (7 to 11)	24.3 (18.5 to 31.3)	-6.9 (-23.2 to 12.8)
		Female	0 (0 to 0)	0.1 (0.1 to 0.2)	1.4 (-21.3 to 29.7)	2 (2 to 3)	5.4 (4 to 7.3)	-3 (-27.3 to 27.5)
Trinidad and Tobago	TBL cancer	Both	46 (28 to 68)	2.4 (1.5 to 3.6)	-6.8 (-34.6 to 31.3)	4690 (3490 to 6180)	250.9 (187.3 to 331.2)	-8.6 (-33 to 20.9)
		Male	34 (21 to 51)	3.8 (2.3 to 5.6)	-8.8 (-39 to 33.6)	3550 (2610 to 4740)	386.1 (286.3 to 513.5)	-10.6 (-34.7 to 19.8)
		Female	12 (7 to 18)	1.2 (0.7 to 1.8)	-0.9 (-30.9 to 39.3)	1140 (826 to 1520)	121.3 (87.5 to 162.8)	-2.5 (-28.2 to 31.3)
	Larynx cancer	Both	11 (7 to 17)	0.6 (0.4 to 0.9)	-5.4 (-31.8 to 26.1)	425 (311 to 560)	22.9 (16.8 to 30.1)	-9.1 (-34.2 to 21.6)
		Male	10 (7 to 15)	1.2 (0.7 to 1.7)	-6.3 (-32.4 to 25.8)	398 (291 to 526)	44 (32.5 to 58.2)	-9.9 (-35 to 20.7)
		Female	1 (1 to 1)	0.1 (0.1 to 0.1)	2.3 (-19.2 to 30.5)	27 (20 to 37)	2.8 (2.1 to 3.8)	-1.3 (-27.7 to 32.6)
Tunisia	TBL cancer	Both	557 (330 to 850)	4.3 (2.6 to 6.6)	-0.9 (-28.9 to 37.3)	61300 (42500 to 85500)	467.4 (325.7 to 648.4)	-2.5 (-27.7 to 30.7)
		Male	490 (284 to 754)	7.8 (4.6 to 12)	-1.7 (-30.1 to 38.5)	55100 (38100 to 77700)	857.4 (592.6 to 1198.4)	-3 (-28.2 to 31.5)
		Female	67 (41 to 102)	1 (0.6 to 1.6)	11 (-19.9 to 53.7)	6170 (4440 to 8310)	93 (66.8 to 125.1)	6.5 (-19.6 to 43.3)
	Larynx cancer	Both	269 (162 to 421)	2.1 (1.2 to 3.2)	8.9 (-22.8 to 55.7)	7540 (5310 to 10500)	57.3 (40.5 to 79.4)	-7.2 (-30.4 to 28.3)
		Male	247 (146 to 389)	3.9 (2.3 to 6)	9.6 (-23.2 to 59.7)	7040 (4900 to 9880)	109.3 (76.8 to 152.4)	-6.5 (-30.8 to 29.9)
		Female	22 (13 to 32)	0.3 (0.2 to 0.5)	7.5 (-19.9 to 43.2)	499 (355 to 677)	7.5 (5.3 to 10.1)	-9.5 (-33.8 to 23.9)
Turkey	TBL cancer	Both	6700 (4550 to 9480)	7.5 (5.1 to 10.5)	-3.1 (-27.5 to 29.4)	737000 (582000 to 921000)	807.1 (638.5 to 1008.2)	-6.2 (-27.5 to 22.6)
		Male	5470 (3700 to 7850)	12.8 (8.7 to 18.3)	-6.2 (-31.2 to 27.1)	620000 (489000 to 780000)	1419 (1120.9 to 1780.4)	-8.5 (-29.8 to 20.1)
		Female	1240 (795 to 1800)	2.6 (1.7 to 3.9)	13 (-20.2 to 58.5)	117000 (92500 to 146000)	247.6 (196.4 to 308.7)	7.2 (-17 to 39.9)
	Larynx cancer	Both	1300 (864 to 1830)	1.4 (1 to 2)	-7.8 (-30.1 to 24.7)	34200 (26400 to 43200)	37.7 (29.1 to 47.6)	-23.3 (-41 to 0.5)

		Male	1130 (748 to 1620)	2.6 (1.8 to 3.8)	-8.7 (-32.9 to 26.9)	30400 (23200 to 38600)	70.5 (54.1 to 89.3)	-23.8 (-42 to 0.5)
		Female	174 (114 to 245)	0.4 (0.2 to 0.5)	-1.7 (-24.4 to 29.1)	3870 (3020 to 4980)	8.2 (6.4 to 10.6)	-18.4 (-38.2 to 9.8)
Turkmenistan	TBL cancer	Both	102 (65 to 147)	2.4 (1.5 to 3.4)	15.5 (-16.9 to 54.9)	11800 (9290 to 14900)	263.5 (208 to 332.8)	14.6 (-9.6 to 45.3)
		Male	76 (48 to 112)	3.9 (2.5 to 5.8)	14 (-23 to 62)	8960 (6950 to 11500)	434.6 (339.9 to 548.3)	13.1 (-11.8 to 44.4)
		Female	26 (17 to 38)	1.2 (0.8 to 1.7)	17.9 (-7.8 to 48.8)	2850 (2190 to 3690)	121.5 (93.9 to 156.6)	17.1 (-8.9 to 47.9)
	Larynx cancer	Both	29 (20 to 41)	0.7 (0.5 to 1)	-1.1 (-20 to 23.6)	1140 (892 to 1460)	25.6 (20.2 to 32.7)	-9 (-29.1 to 15.2)
		Male	20 (14 to 29)	1 (0.7 to 1.5)	7.6 (-14.4 to 37)	833 (651 to 1060)	40.9 (32.3 to 51.9)	-0.2 (-22.7 to 28.1)
		Female	9 (6 to 13)	0.4 (0.3 to 0.6)	-16.5 (-34.1 to 9.1)	308 (234 to 421)	13 (9.9 to 17.6)	-26.8 (-44.4 to -2.7)
Tuvalu	TBL cancer	Both	1 (0 to 1)	5.7 (3.6 to 8.5)	1 (-22.4 to 29)	68 (49 to 94)	639.6 (466.2 to 880.4)	1.3 (-17.3 to 22.3)
		Male	0 (0 to 1)	8.4 (5.2 to 13.2)	-2.4 (-26.5 to 29.7)	47 (34 to 69)	939.6 (681.4 to 1349)	-2.3 (-20.3 to 19)
		Female	0 (0 to 0)	3.4 (2 to 5.1)	5 (-25.1 to 43.2)	21 (14 to 29)	371.8 (261.2 to 515.6)	5.3 (-16.5 to 30.7)
	Larynx cancer	Both	0 (0 to 0)	0.6 (0.3 to 0.8)	-1 (-19.7 to 22.4)	2 (2 to 3)	22.1 (15.9 to 30)	-5.7 (-23.4 to 17.3)
		Male	0 (0 to 0)	0.9 (0.6 to 1.4)	-0.8 (-22.5 to 26.4)	2 (1 to 2)	35.5 (24.3 to 48.5)	-5.7 (-25.4 to 18.9)
		Female	0 (0 to 0)	0.3 (0.2 to 0.4)	-6 (-27.6 to 21.2)	1 (0 to 1)	10.3 (7.3 to 14.4)	-11 (-33 to 18.5)
Uganda	TBL cancer	Both	239 (152 to 336)	1.7 (1.1 to 2.4)	5.7 (-19.3 to 37.8)	27500 (21600 to 33800)	180.8 (143 to 217.7)	6.3 (-13.4 to 27.9)
		Male	148 (93 to 216)	2.4 (1.6 to 3.6)	-0.9 (-29.7 to 38.8)	17100 (13100 to 21400)	258.2 (202.9 to 314.7)	-1 (-20.1 to 21.1)
		Female	91 (54 to 136)	1.1 (0.7 to 1.7)	20.9 (-13.9 to 70.4)	10500 (7800 to 13500)	121.4 (90.7 to 155.4)	23.7 (-7.1 to 58.9)
	Larynx cancer	Both	118 (79 to 167)	0.8 (0.5 to 1)	-3.7 (-22.4 to 19.6)	6210 (4710 to 7940)	37.7 (29.3 to 47.7)	-6.9 (-25.6 to 16.5)
		Male	88 (59 to 126)	1.3 (0.9 to 1.8)	-5 (-24.7 to 21)	4740 (3620 to 6160)	64.3 (49.8 to 83.4)	-8 (-27.7 to 16.7)
		Female	30 (19 to 42)	0.3 (0.2 to 0.5)	2.6 (-19.6 to 31.9)	1470 (1070 to 1920)	16 (11.9 to 20.7)	-0.5 (-23.8 to 30.7)
Ukraine	TBL cancer	Both	5010 (3350 to 6840)	6.8 (4.6 to 9.3)	15.7 (-11.9 to 48.7)	447000 (366000 to 534000)	624.3 (511.1 to 747.8)	15.3 (-6.1 to 39.6)
		Male	4160 (2690 to 5780)	14 (9.2 to 19.5)	14.6 (-14.9 to 49.9)	379000 (300000 to 464000)	1275.5 (1012 to 1566.4)	14.3 (-10.4 to 43)
		Female	852 (538 to 1210)	1.9 (1.2 to 2.8)	11.3 (-19.7 to 47.4)	68200 (53700 to 85700)	166.3 (129.8 to 210.7)	11.7 (-13.2 to 43.5)
	Larynx cancer	Both	1540 (996 to 2150)	2.2 (1.4 to 3.1)	16.2 (-13 to 53.1)	49700 (38600 to 62400)	71 (54.9 to 89.2)	14.6 (-11.5 to 45.1)

		Male	1450 (926 to 2050)	4.9 (3.2 to 6.9)	14.3 (-16.5 to 53.9)	48100 (36900 to 61000)	162.3 (124.6 to 204.7)	12.5 (-14 to 42.8)
		Female	92 (60 to 136)	0.2 (0.2 to 0.4)	10.6 (-10.5 to 36.2)	1530 (1160 to 2080)	4.1 (3.1 to 5.5)	10.4 (-17.6 to 45.5)
United Arab Emirates	TBL cancer	Both	131 (80 to 199)	4 (2.4 to 6.2)	-17.7 (-41 to 12.9)	16600 (12000 to 22000)	402.5 (292.4 to 553)	-17.3 (-36.4 to 7.1)
		Male	104 (63 to 164)	4.5 (2.7 to 7)	-15 (-40.4 to 17.6)	13300 (9470 to 18100)	464.2 (340.2 to 640.3)	-14.7 (-33.1 to 9.1)
		Female	27 (15 to 41)	2.8 (1.4 to 4.7)	-28 (-59.2 to 21.3)	3270 (2240 to 4330)	272.1 (163.1 to 391.9)	-28.4 (-54.3 to 7.8)
	Larynx cancer	Both	133 (78 to 214)	2.1 (1.3 to 3.2)	-8 (-29.7 to 22.9)	5340 (3360 to 8270)	74.2 (51.3 to 105.1)	-14.6 (-33.1 to 10.3)
		Male	121 (69 to 197)	2.6 (1.6 to 4)	-5.3 (-29.3 to 29.5)	4990 (3090 to 7890)	93.9 (63.8 to 135)	-13.1 (-33 to 13.6)
		Female	12 (8 to 18)	0.7 (0.5 to 1.1)	-36.7 (-50.2 to -16.2)	349 (234 to 506)	20.3 (13.7 to 28.2)	-43.3 (-57 to -24.8)
United Kingdom	TBL cancer	Both	12500 (8850 to 16500)	10 (7 to 13.2)	-3.2 (-18.5 to 14.5)	784000 (743000 to 817000)	656.4 (626.5 to 682.5)	-7.1 (-9.8 to -4.7)
		Male	6780 (4610 to 9290)	11.6 (7.8 to 15.9)	-7.6 (-27.2 to 16.1)	431000 (412000 to 447000)	762.1 (730.6 to 789.4)	-10.9 (-13.1 to -8.7)
		Female	5750 (3950 to 7950)	8.6 (5.9 to 12)	1.2 (-19.7 to 28.5)	353000 (328000 to 376000)	564 (529.2 to 598.2)	-3 (-7.7 to 1.6)
	Larynx cancer	Both	2090 (1460 to 2880)	1.8 (1.3 to 2.5)	0.1 (-16.3 to 19.9)	19100 (18200 to 19700)	16.8 (16.1 to 17.3)	-7.5 (-10 to -5)
		Male	1440 (971 to 2030)	2.6 (1.8 to 3.7)	-1 (-22 to 24.6)	15400 (14800 to 16000)	28.4 (27.2 to 29.4)	-8.9 (-11.9 to -6)
		Female	644 (427 to 916)	1.1 (0.8 to 1.6)	0.6 (-16.6 to 22.3)	3660 (2970 to 3940)	6.2 (5.2 to 6.7)	-4.3 (-8.5 to -0.4)
Tanzania	TBL cancer	Both	497 (298 to 818)	2 (1.2 to 3.3)	5.7 (-21.8 to 38.9)	56400 (39800 to 82800)	218.9 (156.7 to 317.8)	5.7 (-14.8 to 27.7)
		Male	352 (192 to 625)	3.1 (1.7 to 5.4)	0.8 (-29.7 to 38.5)	39900 (26700 to 62800)	328 (222.8 to 510.6)	0.5 (-20.2 to 23)
		Female	146 (96 to 212)	1.1 (0.7 to 1.6)	15.7 (-13.6 to 57.8)	16500 (12700 to 20700)	118.6 (93.7 to 145.3)	17.3 (-6.2 to 46.3)
	Larynx cancer	Both	179 (119 to 263)	0.7 (0.5 to 1)	-2.5 (-22 to 20.5)	9210 (6860 to 13000)	33.3 (25.1 to 46.8)	-6 (-25.8 to 17.6)
		Male	139 (91 to 214)	1.1 (0.7 to 1.7)	-4 (-24.4 to 21)	7270 (5240 to 10800)	54.7 (40 to 81)	-7.1 (-27 to 18.2)
		Female	40 (27 to 58)	0.3 (0.2 to 0.4)	-0.3 (-22.2 to 26.5)	1940 (1450 to 2540)	13.2 (10.1 to 17)	-3.5 (-26.8 to 25.5)
Virgin Islands	TBL cancer	Both	10 (7 to 14)	5.5 (3.7 to 7.6)	-4.8 (-22 to 15.3)	1000 (827 to 1170)	547.1 (448.7 to 640.9)	-4.7 (-19 to 9.5)
		Male	7 (4 to 9)	7.9 (5.3 to 11.2)	-5.1 (-26.4 to 19.1)	663 (531 to 792)	800.2 (643.8 to 963.9)	-4.2 (-19.4 to 13)

		Female	4 (2 to 5)	3.5 (2.3 to 5)	-1.4 (-27.7 to 30.1)	338 (275 to 409)	336.4 (270.1 to 409.1)	-2.7 (-21.1 to 17.2)
	Larynx cancer	Both	3 (2 to 4)	1.4 (1 to 2)	-3.8 (-24.2 to 22.3)	91 (74 to 109)	50.1 (40.5 to 60.2)	-3.4 (-21 to 15.7)
		Male	2 (2 to 3)	2.9 (2 to 4)	-0.9 (-22.9 to 27.9)	86 (69 to 103)	103.2 (82.8 to 124.4)	-0.5 (-18.8 to 19.7)
		Female	0 (0 to 0)	0.2 (0.2 to 0.3)	-7.5 (-22.3 to 10.4)	6 (5 to 7)	5.9 (4.7 to 7.3)	-8.6 (-26.1 to 12.4)
United States of America	TBL cancer	Both	61800 (43900 to 80400)	11.1 (7.9 to 14.4)	-11.4 (-23.4 to 2.1)	4120000 (3950000 to 4260000)	756.3 (725.4 to 781)	-11 (-13.6 to -8.6)
		Male	33100 (22500 to 44400)	12.9 (8.8 to 17.3)	-12.6 (-29.4 to 5.9)	2310000 (2240000 to 2390000)	909.3 (879.2 to 939.3)	-12.2 (-14.2 to -9.3)
		Female	28700 (20400 to 38300)	9.6 (6.8 to 12.8)	-10.5 (-26.9 to 8.7)	1810000 (1690000 to 1920000)	624.2 (586.5 to 659.3)	-9.8 (-14.5 to -5.4)
	Larynx cancer	Both	9760 (6700 to 13200)	1.8 (1.3 to 2.5)	-5.5 (-19.8 to 12.3)	108000 (104000 to 112000)	20.3 (19.6 to 21.1)	-7 (-10 to -3.8)
		Male	7910 (5350 to 10900)	3.1 (2.1 to 4.3)	-5.8 (-23.1 to 15.3)	86800 (83000 to 90400)	34.6 (33.1 to 36)	-6.8 (-10.6 to -2.9)
		Female	1850 (1270 to 2530)	0.7 (0.5 to 0.9)	-7 (-21.4 to 10.3)	21200 (19800 to 23000)	7.6 (7.1 to 8.3)	-9.6 (-13.7 to -4.7)
Uruguay	TBL cancer	Both	398 (255 to 568)	7.9 (5.1 to 11.4)	-4.1 (-27.7 to 24.4)	39100 (36400 to 41600)	805.2 (749.3 to 857.3)	-6.4 (-13.1 to 0.5)
		Male	290 (186 to 409)	13.1 (8.4 to 18.4)	-10 (-33.1 to 19.3)	29100 (27100 to 31000)	1331.6 (1239.9 to 1419.8)	-11.7 (-18.2 to -5.2)
		Female	107 (64 to 159)	3.8 (2.3 to 5.8)	15.5 (-22.9 to 68)	10100 (9190 to 11000)	379.6 (347.9 to 413.8)	11.9 (1.4 to 22.4)
	Larynx cancer	Both	121 (80 to 173)	2.5 (1.6 to 3.5)	-1.1 (-28.2 to 31.1)	3140 (2820 to 3470)	65 (58.1 to 72.2)	-7.8 (-18.2 to 4.5)
		Male	108 (70 to 156)	4.9 (3.2 to 7.1)	-1.9 (-29.6 to 32.8)	2860 (2550 to 3200)	131.6 (117.3 to 146.9)	-8.3 (-19.1 to 4.6)
		Female	13 (8 to 19)	0.5 (0.3 to 0.7)	2.5 (-23.7 to 40.3)	280 (230 to 326)	10.2 (8.4 to 11.9)	-5.9 (-19.4 to 10.5)
Uzbekistan	TBL cancer	Both	673 (444 to 943)	2.9 (2 to 4)	6.8 (-17.7 to 36.2)	79900 (65900 to 96000)	317.9 (264.7 to 376.9)	6.8 (-11.2 to 27.1)
		Male	479 (309 to 688)	4.7 (3.1 to 6.6)	4 (-24.7 to 41.2)	57300 (46600 to 69600)	505.6 (418.1 to 605.2)	4 (-14.8 to 25.5)
		Female	194 (127 to 277)	1.6 (1 to 2.3)	13.5 (-16.9 to 52.2)	22600 (18400 to 27400)	167.9 (139.1 to 201.2)	14.9 (-5.7 to 38.9)
	Larynx cancer	Both	300 (209 to 418)	1.2 (0.8 to 1.7)	-22.5 (-35.3 to -8)	11300 (9350 to 13500)	43.9 (36.6 to 52.1)	-31.5 (-43.9 to -18.2)
		Male	175 (119 to 243)	1.6 (1.1 to 2.2)	-20.6 (-36.5 to -1.9)	6960 (5660 to 8390)	61.3 (50.8 to 73.3)	-29.9 (-42.4 to -14.7)
		Female	125 (85 to 179)	0.9 (0.6 to 1.3)	-24.6 (-39.1 to -3.4)	4340 (3480 to 5310)	30.4 (24.8 to 36.8)	-33.6 (-47 to -10.8)

Vanuatu	TBL cancer	Both	9 (6 to 15)	5.4 (3.2 to 8.7)	4.4 (-17.2 to 36.3)	1120 (735 to 1690)	610.1 (403.5 to 917.5)	4.1 (-12.3 to 26.2)
		Male	8 (4 to 13)	8.3 (4.5 to 13.9)	2.4 (-21.3 to 36)	904 (544 to 1450)	940.4 (567.3 to 1502.6)	2 (-13.4 to 23.2)
		Female	2 (1 to 3)	2.3 (1.3 to 3.6)	12 (-20.4 to 60.6)	218 (137 to 318)	248.5 (159.7 to 359.9)	11.6 (-10.7 to 39)
	Larynx cancer	Both	1 (1 to 1)	0.5 (0.3 to 0.8)	1.8 (-17.6 to 25.4)	43 (29 to 59)	23.6 (16.1 to 32.5)	-0.6 (-19.3 to 20.8)
		Male	1 (0 to 1)	0.8 (0.5 to 1.2)	2 (-20.4 to 29.2)	34 (22 to 48)	36.4 (23.7 to 50.3)	-0.6 (-20.7 to 22.3)
		Female	0 (0 to 0)	0.2 (0.1 to 0.3)	1.3 (-19.8 to 28.3)	9 (5 to 13)	9.5 (6 to 13.8)	-0.9 (-24.1 to 29.6)
Venezuela	TBL cancer	Both	1340 (870 to 1990)	4.6 (3 to 6.7)	-2.1 (-30.1 to 33.2)	137000 (101000 to 178000)	455.6 (335 to 589.1)	-2.9 (-27.6 to 26.2)
		Male	796 (494 to 1180)	5.8 (3.6 to 8.5)	-5.9 (-34.8 to 29.7)	85900 (62800 to 112000)	603.5 (445.3 to 785.3)	-6.1 (-30.9 to 22.9)
		Female	545 (331 to 827)	3.5 (2.1 to 5.3)	3.1 (-31.3 to 53.3)	51000 (36300 to 70400)	323.7 (231.2 to 447.9)	2 (-27 to 41.1)
	Larynx cancer	Both	449 (284 to 662)	1.5 (1 to 2.2)	-1 (-31.6 to 33.4)	15500 (11400 to 20700)	51.4 (38 to 68)	-4.2 (-30 to 27.9)
		Male	377 (233 to 563)	2.7 (1.7 to 3.9)	-2.3 (-34.6 to 37.3)	13600 (9910 to 18200)	94.8 (69.6 to 126.3)	-5.4 (-31.3 to 27.7)
		Female	72 (47 to 105)	0.5 (0.3 to 0.7)	2.5 (-23.1 to 34.3)	1940 (1450 to 2620)	12.4 (9.3 to 16.6)	-0.2 (-26.2 to 31)
Vietnam	TBL cancer	Both	5930 (3920 to 8370)	6.1 (4 to 8.5)	6.9 (-17.2 to 35.1)	671000 (511000 to 868000)	662.7 (507.3 to 845.3)	4.6 (-16.2 to 28)
		Male	4280 (2720 to 6200)	10 (6.5 to 14.2)	3.9 (-21.5 to 34.4)	495000 (373000 to 638000)	1092.4 (831.7 to 1389.7)	2.1 (-18.9 to 26.1)
		Female	1650 (1020 to 2460)	3.1 (1.9 to 4.6)	12.5 (-22.1 to 57.6)	176000 (132000 to 233000)	322.2 (242.6 to 422)	8.9 (-15.5 to 36.9)
	Larynx cancer	Both	1630 (1060 to 2370)	1.6 (1.1 to 2.3)	20.3 (-9.7 to 56.6)	51200 (38700 to 65900)	49.4 (37.8 to 62.9)	1.6 (-19.5 to 26.6)
		Male	1510 (985 to 2210)	3.3 (2.2 to 4.8)	19.7 (-11 to 59.8)	47700 (35800 to 62100)	102.9 (78.4 to 131.7)	1.1 (-20 to 25.3)
		Female	118 (75 to 172)	0.2 (0.1 to 0.3)	8.2 (-15.9 to 38.9)	3460 (2510 to 4630)	6.3 (4.6 to 8.3)	-7.1 (-29.4 to 21.9)
Yemen	TBL cancer	Both	308 (181 to 505)	2.3 (1.3 to 3.8)	-1.7 (-30.1 to 31)	35900 (24200 to 53400)	246.5 (168 to 365.2)	-1.9 (-21.3 to 21.9)
		Male	236 (130 to 412)	3.6 (2 to 6.1)	-2.8 (-32.9 to 35.1)	27500 (17900 to 43900)	390.3 (252.3 to 616.5)	-2.9 (-23.7 to 22.1)
		Female	72 (46 to 109)	1 (0.7 to 1.5)	7.3 (-23.6 to 40.3)	8390 (5950 to 11500)	106.4 (78 to 144.1)	8.7 (-19.9 to 38.2)
	Larynx cancer	Both	189 (114 to 294)	1.3 (0.8 to 2)	5 (-18.4 to 32.8)	9220 (6160 to 12900)	61.4 (42.2 to 84.5)	2.5 (-18.6 to 27.9)
		Male	147 (83 to 231)	2.1 (1.2 to 3.3)	5.6 (-19.8 to 37.5)	7250 (4670 to 10400)	99.4 (65.4 to 141.1)	3.2 (-19.9 to 31.4)

		Female	42 (25 to 67)	0.6 (0.3 to 0.9)	7.7 (-17.6 to 39.5)	1970 (1290 to 2780)	24.4 (16.6 to 34.5)	5.2 (-19.2 to 37.9)
Zambia	TBL cancer	Both	165 (105 to 247)	2.5 (1.6 to 3.6)	2 (-25.4 to 35.8)	19600 (13900 to 25900)	267.5 (190.9 to 349.5)	0.1 (-22 to 27.2)
		Male	113 (64 to 176)	3.6 (2.1 to 5.5)	-2.3 (-32.4 to 36.1)	13400 (8750 to 18600)	391.3 (257.3 to 531.8)	-4.8 (-26.9 to 24.5)
		Female	52 (30 to 82)	1.4 (0.8 to 2.3)	17.6 (-21.9 to 68.4)	6160 (4230 to 8560)	156.3 (110.2 to 213.8)	15.8 (-12.3 to 52)
	Larynx cancer	Both	64 (41 to 97)	0.8 (0.6 to 1.3)	-6.3 (-29.1 to 19.6)	3440 (2510 to 4730)	42.9 (31.6 to 57.6)	-13.1 (-33.7 to 13.4)
		Male	51 (32 to 78)	1.4 (0.9 to 2.1)	-5.8 (-30 to 22.6)	2780 (1990 to 3900)	71.6 (51.9 to 98.9)	-12.9 (-34.8 to 16.4)
		Female	13 (9 to 20)	0.3 (0.2 to 0.5)	-8.2 (-29.6 to 21.3)	659 (457 to 919)	15.6 (11 to 21.6)	-16.1 (-37.7 to 13.9)
Zimbabwe	TBL cancer	Both	237 (157 to 339)	3.3 (2.2 to 4.7)	1.1 (-22.4 to 31.7)	28600 (21600 to 35600)	368.9 (282.1 to 451.1)	0.6 (-18.4 to 21.4)
		Male	133 (84 to 193)	4.4 (2.8 to 6.2)	0.7 (-28.2 to 40.7)	16600 (12800 to 20700)	499.9 (392.7 to 608.5)	-3 (-22.9 to 20.1)
		Female	104 (63 to 158)	2.5 (1.5 to 3.8)	3.2 (-29.3 to 52.7)	12000 (8390 to 15800)	272.9 (191.5 to 363)	7.8 (-19.6 to 45.5)
	Larynx cancer	Both	90 (61 to 126)	1.2 (0.8 to 1.6)	-2.9 (-21.8 to 19.3)	5040 (3910 to 6410)	61.9 (49.1 to 77.5)	-7.2 (-26.2 to 14.7)
		Male	70 (47 to 100)	2.1 (1.4 to 3)	0.8 (-20.8 to 28.9)	4060 (3200 to 5190)	116 (92.7 to 144.5)	-5.4 (-25.3 to 18.3)
		Female	20 (12 to 30)	0.4 (0.3 to 0.7)	-10.2 (-32.8 to 21.5)	976 (672 to 1360)	21.2 (14.8 to 29)	-10.6 (-34.3 to 23.7)

Table S4. Tracheal, bronchus, and lung (TBL) cancer deaths (counts and age-standardised rates) attributable to specific risk factors in 2019, at global, regional, national levels, and Socio-demographic Index (SDI) quintiles, by sex and for both sexes combined.

95% UIs given in parentheses.

Location	Sex	Smoking		Ambient particulate matter pollution		Household air pollution from solid fuels		Secondhand smoke		Residential radon		Diet low in fruits		High fasting plasma glucose		Occupational exposure to asbestos		Occupational exposure excluding asbestos	
		Number	ASDR per 100,000	Number	ASDR per 100,000	Number	ASDR per 100,000	Number	ASDR per 100,000	Number	ASDR per 100,000	Number	ASDR per 100,000	Number	ASDR per 100,000	Number	ASDR per 100,000	Number	ASDR per 100,000
Global	Both	1310000 (1200000 to 1430000)	16.1 (14.7 to 17.5)	308000 (227000 to 396000)	3.8 (2.8 to 4.9)	79800 (45100 to 125000)	1 (0.5 to 1.5)	113000 (67500 to 170000)	1.4 (0.8 to 2.1)	83700 (16500 to 162000)	1 (0.2 to 2)	77200 (22600 to 115000)	1 (0.3 to 1.4)	179000 (42700 to 389000)	2.2 (0.5 to 4.8)	199000 (140000 to 257000)	2.5 (1.8 to 3.3)	99600 (44100 to 164000)	1.1 (0.5 to 1.9)
	Male	1060000 (952000 to 1170000)	28.5 (25.7 to 31.3)	216000 (157000 to 281000)	5.8 (4.2 to 7.5)	51200 (27700 to 82200)	1.3 (0.7 to 2.1)	66300 (38300 to 101000)	1.8 (1 to 2.7)	56800 (11300 to 110000)	1.5 (0.3 to 2.9)	52300 (15200 to 78600)	1.4 (0.4 to 2.1)	124000 (21100 to 286000)	3.4 (0.6 to 7.9)	172000 (116000 to 231000)	5.1 (3.5 to 6.8)	73900 (31400 to 123000)	1.8 (0.7 to 3)
	Female	255000 (230000 to 277000)	5.8 (5.2 to 6.3)	91300 (65500 to 119000)	2.1 (1.5 to 2.7)	28500 (16000 to 44500)	0.7 (0.4 to 1)	47200 (27700 to 71200)	1.1 (0.6 to 1.6)	26900 (5220 to 52200)	0.6 (0.1 to 1.2)	24900 (7200 to 37400)	0.6 (0.2 to 0.9)	54900 (10800 to 129000)	1.3 (0.2 to 2.9)	26500 (15600 to 37200)	0.6 (0.4 to 0.8)	25700 (10200 to 43800)	0.6 (0.2 to 1)
High SDI	Both	396000 (369000 to 416000)	20.5 (19.2 to 21.4)	42900 (28100 to 61500)	2.2 (1.5 to 3.2)	277 (73 to 720)	0 (0 to 0)	20200 (12000 to 30400)	1.1 (0.7 to 1.6)	23900 (4840 to 46100)	1.2 (0.3 to 2.4)	22400 (5960 to 33300)	1.2 (0.3 to 1.7)	61900 (15100 to 132000)	3.1 (0.8 to 6.6)	120000 (87200 to 152000)	5.7 (4.1 to 7.3)	20000 (4080 to 37600)	1.1 (0.2 to 2.1)
	Male	265000 (250000 to 277000)	30.3 (28.6 to 31.7)	27600 (18400 to 39400)	3.2 (2.1 to 4.5)	152 (38 to 399)	0 (0 to 0)	12300 (7270 to 18500)	1.4 (0.9 to 2.2)	14500 (2890 to 27900)	1.7 (0.3 to 3.2)	13600 (3670 to 20200)	1.6 (0.4 to 2.3)	40000 (6940 to 89400)	4.5 (0.8 to 10.1)	104000 (72800 to	11.5 (8 to 14.9)	14000 (2740 to 26500)	1.6 (0.3 to 3.1)

																135000)			
	Female	131000 (119000 to 140000)	12.5 (11.5 to 13.2)	15300 (9750 to 22400)	1.4 (0.9 to 2.1)	125 (33 to 325)	0 (0 to 0)	7870 (4720 to 11800)	0.8 (0.5 to 1.2)	9340 (1880 to 18200)	0.9 (0.2 to 1.7)	8720 (2350 to 13100)	0.8 (0.2 to 1.2)	21900 (4350 to 50200)	2 (0.4 to 4.6)	15200 (8810 to 21400)	1.2 (0.7 to 1.7)	5940 (1060 to 11500)	0.6 (0.1 to 1.2)
High-middle SDI	Both	417000 (375000 to 460000)	20.2 (18.1 to 22.3)	109000 (80200 to 140000)	5.3 (3.9 to 6.8)	10900 (4300 to 22400)	0.5 (0.2 to 1.1)	39400 (23900 to 58600)	1.9 (1.2 to 2.9)	28400 (5190 to 57200)	1.4 (0.3 to 2.8)	20000 (5700 to 30100)	1 (0.3 to 1.5)	49600 (11200 to 110000)	2.4 (0.5 to 5.3)	47600 (32300 to 64500)	2.3 (1.6 to 3.2)	30500 (13400 to 50400)	1.4 (0.6 to 2.4)
	Male	351000 (309000 to 392000)	38.5 (33.9 to 42.9)	76800 (55300 to 100000)	8.5 (6.1 to 11)	6530 (2380 to 14000)	0.7 (0.3 to 1.5)	23700 (13700 to 35800)	2.6 (1.5 to 3.9)	20400 (3690 to 41000)	2.3 (0.4 to 4.5)	14200 (3930 to 21800)	1.6 (0.4 to 2.4)	36300 (5940 to 83500)	4.1 (0.7 to 9.3)	41700 (26700 to 58300)	4.9 (3.2 to 6.9)	22700 (9400 to 38100)	2.2 (0.9 to 3.8)
	Female	66600 (58900 to 75300)	5.8 (5.1 to 6.5)	31800 (23000 to 41700)	2.8 (2 to 3.7)	4400 (1800 to 8560)	0.4 (0.2 to 0.8)	15700 (9380 to 23800)	1.4 (0.8 to 2.1)	7950 (1450 to 16200)	0.7 (0.1 to 1.4)	5800 (1680 to 8840)	0.5 (0.1 to 0.8)	13300 (2520 to 31700)	1.1 (0.2 to 2.7)	5910 (3340 to 8430)	0.5 (0.3 to 0.7)	7850 (3200 to 13400)	0.7 (0.3 to 1.2)
Middle SDI	Both	384000 (326000 to 444000)	16 (13.7 to 18.5)	125000 (90300 to 163000)	5.2 (3.8 to 6.8)	34800 (17100 to 58900)	1.5 (0.7 to 2.5)	43000 (25300 to 65300)	1.8 (1.1 to 2.7)	21700 (3790 to 45100)	0.9 (0.2 to 1.9)	23800 (6630 to 36600)	1 (0.3 to 1.5)	48900 (11200 to 110000)	2.1 (0.5 to 4.7)	21900 (14200 to 31700)	1 (0.7 to 1.5)	35700 (17600 to 56700)	1.3 (0.6 to 2.1)
	Male	339000 (283000 to 398000)	30.2 (25.3 to 35.3)	88900 (61900 to 118000)	7.9 (5.5 to 10.4)	21700 (9840 to 38600)	1.9 (0.9 to 3.4)	23900 (13500 to 36900)	2.1 (1.2 to 3.3)	15000 (2680 to 30700)	1.3 (0.2 to 2.7)	16600 (4670 to 26300)	1.5 (0.4 to 2.3)	34300 (5570 to 80400)	3.2 (0.5 to 7.4)	18100 (10600 to 28000)	1.9 (1.1 to 2.9)	26400 (12500 to 43100)	2 (0.9 to 3.2)
	Female	45000 (37800 to 53300)	3.7 (3.1 to 4.4)	36600 (25800 to 48500)	2.9 (2.1 to 3.9)	13100 (6570 to 21900)	1 (0.5 to 1.7)	19100 (11100 to 29000)	1.5 (0.9 to 2.3)	6780 (1150 to 14100)	0.5 (0.1 to 1.1)	7240 (2080 to 11100)	0.6 (0.2 to 0.9)	14700 (2840 to 35300)	1.2 (0.2 to 2.9)	3840 (2160 to 5720)	0.3 (0.2 to 0.5)	9250 (4100 to 15300)	0.7 (0.3 to 1.1)
Low-middle SDI	Both	95000 (86000 to 105000)	7.3 (6.6 to 8)	25800 (16900 to 35000)	2 (1.3 to 2.6)	22900 (14100 to 32600)	1.7 (1.1 to 2.5)	9320 (5330 to 14200)	0.7 (0.4 to 1.1)	7530 (1500 to 15300)	0.6 (0.1 to 1.2)	8540 (3130 to 12900)	0.6 (0.2 to 1)	14900 (3360 to 33500)	1.2 (0.3 to 2.6)	7860 (5010 to 11300)	0.7 (0.4 to 1)	10700 (5270 to 16800)	0.7 (0.4 to 1.1)

	Male	84000 (75500 to 93000)	13.6 (12.2 to 15)	19100 (12500 to 25700)	3 (2 to 4.1)	15000 (8630 to 22100)	2.4 (1.4 to 3.5)	5260 (2970 to 8130)	0.8 (0.5 to 1.3)	5280 (1080 to 10700)	0.8 (0.2 to 1.7)	5970 (2190 to 9120)	0.9 (0.3 to 1.4)	10700 (1860 to 24800)	1.8 (0.3 to 4.1)	6590 (3830 to 9950)	1.2 (0.7 to 1.8)	8490 (4020 to 13500)	1.2 (0.6 to 1.9)
	Female	11000 (9350 to 12800)	1.7 (1.4 to 2)	6730 (4280 to 9420)	1 (0.6 to 1.4)	7930 (5020 to 11200)	1.2 (0.7 to 1.6)	4060 (2330 to 6080)	0.6 (0.3 to 0.9)	2250 (434 to 4520)	0.3 (0.1 to 0.7)	2570 (903 to 3940)	0.4 (0.1 to 0.6)	4250 (873 to 10200)	0.6 (0.1 to 1.5)	1270 (720 to 1920)	0.2 (0.1 to 0.3)	2210 (993 to 3620)	0.3 (0.1 to 0.5)
Low SDI	Both	18900 (15900 to 22400)	4 (3.4 to 4.7)	4860 (2860 to 7310)	1 (0.6 to 1.5)	10800 (7120 to 15300)	2.2 (1.5 to 3.1)	1540 (840 to 2420)	0.3 (0.2 to 0.5)	2180 (429 to 4470)	0.4 (0.1 to 0.9)	2440 (864 to 3820)	0.5 (0.2 to 0.8)	3620 (753 to 8310)	0.8 (0.2 to 1.8)	1620 (826 to 4090)	0.4 (0.2 to 1)	2690 (1260 to 4360)	0.5 (0.2 to 0.7)
	Male	17100 (14200 to 20500)	7.4 (6.2 to 8.8)	3890 (2220 to 5930)	1.6 (0.9 to 2.5)	7870 (4990 to 11500)	3.3 (2.1 to 4.8)	1090 (580 to 1750)	0.5 (0.2 to 0.7)	1640 (327 to 3390)	0.7 (0.1 to 1.4)	1840 (646 to 2980)	0.8 (0.3 to 1.2)	2820 (480 to 6920)	1.3 (0.2 to 3.1)	1350 (605 to 3800)	0.7 (0.3 to 1.9)	2260 (1020 to 3730)	0.8 (0.4 to 1.3)
	Female	1850 (1550 to 2140)	0.8 (0.7 to 0.9)	970 (539 to 1490)	0.4 (0.2 to 0.6)	2900 (1990 to 4110)	1.2 (0.8 to 1.6)	454 (244 to 716)	0.2 (0.1 to 0.3)	538 (109 to 1100)	0.2 (0 to 0.4)	596 (205 to 913)	0.2 (0.1 to 0.4)	800 (159 to 1900)	0.3 (0.1 to 0.8)	271 (115 to 432)	0.1 (0.1 to 0.2)	426 (193 to 706)	0.1 (0.1 to 0.2)
Central Asia	Both	8870 (7910 to 9910)	12 (10.8 to 13.3)	2230 (1530 to 3050)	3 (2.1 to 4.1)	381 (171 to 682)	0.5 (0.2 to 0.9)	822 (495 to 1230)	1.1 (0.7 to 1.7)	1030 (171 to 2410)	1.4 (0.2 to 3.3)	560 (155 to 857)	0.8 (0.2 to 1.2)	1130 (240 to 2520)	1.6 (0.4 to 3.6)	718 (445 to 1040)	1.1 (0.7 to 1.6)	910 (451 to 1440)	1 (0.5 to 1.6)
	Male	8470 (7530 to 9470)	27.4 (24.5 to 30.3)	1750 (1200 to 2410)	5.6 (3.8 to 7.6)	272 (116 to 515)	0.9 (0.4 to 1.7)	560 (333 to 850)	1.8 (1.1 to 2.8)	810 (135 to 1880)	2.6 (0.4 to 6)	435 (118 to 669)	1.4 (0.4 to 2.1)	882 (144 to 2050)	3 (0.5 to 6.9)	635 (365 to 942)	2.5 (1.4 to 3.7)	775 (368 to 1250)	1.9 (0.9 to 3.1)
	Female	400 (335 to 483)	1 (0.8 to 1.2)	478 (324 to 665)	1.2 (0.8 to 1.6)	109 (54 to 188)	0.3 (0.1 to 0.5)	262 (160 to 380)	0.6 (0.4 to 0.9)	219 (35 to 519)	0.6 (0.1 to 1.3)	125 (35 to 192)	0.3 (0.1 to 0.5)	248 (48 to 578)	0.6 (0.1 to 1.5)	82 (36 to 142)	0.2 (0.1 to 0.4)	134 (63 to 219)	0.3 (0.1 to 0.5)
Central Europe	Both	60500 (52800 to 69000)	28.3 (24.7 to 32.3)	11300 (8120 to 14900)	5.3 (3.8 to 7)	1430 (466 to 3200)	0.7 (0.2 to 1.5)	4650 (2730 to 7100)	2.2 (1.3 to 3.4)	4420 (788 to 8800)	2.1 (0.4 to 4.1)	3230 (850 to 4940)	1.5 (0.4 to 2.3)	8450 (1860 to 18500)	3.8 (0.8 to 8.4)	6780 (4150 to 9980)	3 (1.8 to 4.4)	3350 (555 to 6500)	1.6 (0.3 to 3.2)
	Male	46000 (39800 to 52800)	49.1 (42.5 to 56.2)	8100 (5810 to 10800)	8.7 (6.2 to 11.6)	911 (279 to 2080)	1 (0.3 to 2.2)	3180 (1850 to 4950)	3.4 (2 to 5.4)	3130 (565 to 6210)	3.4 (0.6 to 6.7)	2270 (597 to 3500)	2.4 (0.6 to 3.8)	6290 (1080 to 14300)	6.7 (1.1 to 15.2)	6130 (3570 to 9320)	6.4 (3.7 to 9.7)	2610 (415 to 5060)	2.7 (0.4 to 5.3)
	Female	14400 (12400	12.2 (10.5	3180 (2260	2.7 (1.9	519 (178	0.4 (0.2 to 1)	1470 (869	1.3 (0.7	1280 (231	1.1 (0.2	964 (268	0.8 (0.2	2170 (420	1.7 (0.3 to 4)	652 (370	0.5 (0.3	746 (102	0.7 (0.1

		to 16600)	to 14.1)	to 4230)	to 3.6)	to 1130)		to 2240)	to 1.9)	to 2600)	to 2.2)	to 1500)	to 1.3)	to 5010)		to 988)	to 0.8)	to 1520)	to 1.4)
Eastern Europe	Both	56600 (49300 to 64300)	16.2 (14.1 to 18.4)	6560 (3780 to 9700)	1.9 (1.1 to 2.8)	250 (73 to 614)	0.1 (0 to 0.2)	3460 (2040 to 5230)	1 (0.6 to 1.5)	5120 (933 to 10600)	1.5 (0.3 to 3.1)	3640 (1080 to 5560)	1.1 (0.3 to 1.6)	4050 (823 to 9490)	1.1 (0.2 to 2.7)	4900 (3000 to 7200)	1.4 (0.8 to 2)	2410 (434 to 4590)	0.7 (0.1 to 1.3)
	Male	52000 (44900 to 59600)	38.9 (33.5 to 44.5)	5280 (3040 to 7960)	4 (2.3 to 6)	185 (53 to 449)	0.1 (0 to 0.3)	2300 (1310 to 3530)	1.7 (1 to 2.6)	4080 (732 to 8480)	3.1 (0.5 to 6.4)	2910 (868 to 4470)	2.2 (0.7 to 3.4)	3240 (533 to 7890)	2.5 (0.4 to 6)	4300 (2400 to 6560)	3.4 (1.9 to 5.1)	1980 (344 to 3790)	1.3 (0.2 to 2.6)
	Female	4630 (3860 to 5540)	2.2 (1.8 to 2.7)	1270 (748 to 1910)	0.6 (0.3 to 0.9)	65 (19 to 161)	0 (0 to 0.1)	1160 (689 to 1780)	0.6 (0.3 to 0.9)	1040 (202 to 2100)	0.5 (0.1 to 1)	734 (217 to 1150)	0.3 (0.1 to 0.5)	808 (144 to 1950)	0.4 (0.1 to 0.9)	592 (325 to 904)	0.3 (0.1 to 0.4)	432 (66 to 865)	0.2 (0 to 0.4)
Australasia	Both	6850 (6220 to 7370)	13.6 (12.5 to 14.6)	356 (86 to 693)	0.7 (0.2 to 1.4)	3 (0 to 9)	0 (0 to 0)	347 (199 to 529)	0.7 (0.4 to 1.1)	169 (26 to 463)	0.3 (0.1 to 0.9)	491 (127 to 743)	1 (0.3 to 1.5)	980 (229 to 2160)	1.9 (0.4 to 4.1)	3820 (2890 to 4690)	7.1 (5.4 to 8.8)	458 (82 to 880)	0.9 (0.2 to 1.8)
	Male	4170 (3810 to 4520)	17.7 (16.1 to 19.1)	210 (51 to 412)	0.9 (0.2 to 1.7)	1 (0 to 4)	0 (0 to 0)	217 (123 to 334)	0.9 (0.5 to 1.5)	97 (15 to 265)	0.4 (0.1 to 1.1)	286 (72 to 432)	1.2 (0.3 to 1.8)	627 (109 to 1470)	2.6 (0.4 to 6.1)	3190 (2330 to 3990)	13 (9.5 to 16.3)	306 (52 to 593)	1.3 (0.2 to 2.5)
	Female	2670 (2370 to 2930)	10.1 (9 to 11)	146 (36 to 289)	0.5 (0.1 to 1.1)	1 (0 to 5)	0 (0 to 0)	129 (74 to 200)	0.5 (0.3 to 0.8)	72 (11 to 194)	0.3 (0 to 0.7)	205 (55 to 316)	0.8 (0.2 to 1.2)	353 (68 to 847)	1.3 (0.2 to 3)	632 (367 to 918)	2.1 (1.3 to 3.1)	152 (24 to 300)	0.6 (0.1 to 1.2)
High-income Asia Pacific	Both	72500 (64100 to 78000)	14.7 (13.3 to 15.7)	12600 (8280 to 17900)	2.6 (1.7 to 3.6)	21 (5 to 63)	0 (0 to 0)	3870 (2220 to 6020)	0.8 (0.5 to 1.3)	2620 (441 to 5590)	0.5 (0.1 to 1.2)	4710 (1340 to 7100)	0.9 (0.3 to 1.4)	8880 (1880 to 20000)	1.7 (0.4 to 3.9)	20100 (13200 to 27100)	3.6 (2.3 to 4.8)	3650 (659 to 6960)	0.9 (0.2 to 1.7)
	Male	60100 (54100 to 64100)	28.2 (25.5 to 30)	8740 (5880 to 12400)	4.1 (2.8 to 5.9)	13 (3 to 37)	0 (0 to 0)	2330 (1330 to 3670)	1.1 (0.7 to 1.8)	1830 (306 to 3930)	0.9 (0.1 to 1.9)	3250 (938 to 4890)	1.5 (0.4 to 2.3)	6680 (1100 to 15600)	3.1 (0.5 to 7.2)	18400 (11700 to 25300)	8.1 (5.2 to 11.2)	2900 (505 to 5560)	1.5 (0.3 to 2.8)
	Female	12400 (10000 to 14400)	4.3 (3.6 to 4.9)	3830 (2460 to 5570)	1.3 (0.9 to 1.9)	9 (2 to 26)	0 (0 to 0)	1530 (879 to 2320)	0.6 (0.3 to 0.9)	789 (135 to 1710)	0.3 (0 to 0.6)	1460 (424 to 2250)	0.5 (0.1 to 0.7)	2200 (400 to 5300)	0.7 (0.1 to 1.7)	1680 (721 to 2700)	0.4 (0.2 to 0.7)	743 (115 to 1480)	0.3 (0.1 to 0.7)
High-income North America	Both	164000 (153000 to 172000)	25.5 (23.9 to 26.8)	9060 (4330 to 15200)	1.4 (0.7 to 2.4)	28 (5 to 88)	0 (0 to 0)	7510 (4480 to 11100)	1.2 (0.7 to 1.8)	9760 (1940 to 18800)	1.5 (0.3 to 2.9)	8460 (2140 to 12600)	1.3 (0.3 to 2)	29600 (7340 to 61700)	4.5 (1.1 to 9.5)	41200 (29700 to 53200)	6.2 (4.4 to 7.9)	7030 (1190 to 13700)	1.1 (0.2 to 2.2)

	Male	94500 (89800 to 98500)	32.4 (30.8 to 33.9)	4990 (2390 to 8320)	1.7 (0.8 to 2.9)	12 (2 to 38)	0 (0 to 0)	4500 (2690 to 6680)	1.6 (0.9 to 2.3)	5360 (1070 to 10300)	1.9 (0.4 to 3.6)	4590 (1210 to 6910)	1.6 (0.4 to 2.4)	18000 (3240 to 38800)	6.1 (1.1 to 13.2)	35300 (24300 to 46700)	12.1 (8.4 to 16.1)	4310 (665 to 8450)	1.4 (0.2 to 2.8)
	Female	69300 (63100 to 74600)	19.8 (18.2 to 21.2)	4070 (1940 to 6840)	1.2 (0.6 to 1.9)	15 (3 to 48)	0 (0 to 0)	3010 (1790 to 4530)	0.9 (0.5 to 1.4)	4400 (868 to 8470)	1.3 (0.2 to 2.4)	3870 (1010 to 5910)	1.1 (0.3 to 1.7)	11700 (2390 to 26500)	3.2 (0.7 to 7.4)	5880 (3460 to 8620)	1.6 (0.9 to 2.3)	2720 (398 to 5400)	0.8 (0.1 to 1.6)
Southern Latin America	Both	12700 (11900 to 13500)	15.2 (14.2 to 16.1)	2010 (1310 to 2880)	2.4 (1.6 to 3.4)	109 (37 to 251)	0.1 (0 to 0.3)	1040 (602 to 1620)	1.2 (0.7 to 1.9)	559 (71 to 1560)	0.7 (0.1 to 1.9)	499 (125 to 749)	0.6 (0.1 to 0.9)	1890 (441 to 4080)	2.2 (0.5 to 4.8)	2050 (1290 to 2940)	2.4 (1.5 to 3.4)	1280 (644 to 2010)	1.5 (0.8 to 2.4)
	Male	9010 (8440 to 9600)	24.4 (22.8 to 25.9)	1320 (846 to 1890)	3.6 (2.3 to 5.2)	61 (20 to 142)	0.2 (0.1 to 0.4)	694 (402 to 1090)	1.9 (1.1 to 3)	371 (48 to 1040)	1 (0.1 to 2.8)	315 (78 to 484)	0.9 (0.2 to 1.3)	1290 (221 to 2950)	3.5 (0.6 to 8)	1770 (1050 to 2610)	4.9 (2.9 to 7.2)	1030 (493 to 1660)	2.7 (1.3 to 4.3)
	Female	3690 (3360 to 4030)	7.9 (7.2 to 8.6)	693 (455 to 984)	1.5 (1 to 2.1)	49 (18 to 106)	0.1 (0 to 0.2)	349 (201 to 537)	0.7 (0.4 to 1.2)	188 (25 to 515)	0.4 (0.1 to 1.1)	184 (47 to 279)	0.4 (0.1 to 0.6)	601 (116 to 1370)	1.2 (0.2 to 2.8)	282 (151 to 432)	0.6 (0.3 to 0.9)	249 (118 to 405)	0.6 (0.3 to 0.9)
Western Europe	Both	184000 (173000 to 193000)	20.5 (19.4 to 21.4)	20200 (13200 to 29400)	2.2 (1.5 to 3.3)	74 (19 to 187)	0 (0 to 0)	9360 (5520 to 14100)	1.1 (0.7 to 1.7)	14800 (2950 to 29300)	1.6 (0.3 to 3.3)	8750 (2310 to 13000)	1 (0.3 to 1.5)	27500 (6520 to 58900)	2.9 (0.7 to 6.2)	71000 (52400 to 89500)	7.1 (5.2 to 9)	9270 (1650 to 17700)	1.1 (0.2 to 2.2)
	Male	130000 (123000 to 136000)	31.7 (30.1 to 33.1)	13700 (8900 to 19800)	3.3 (2.2 to 4.8)	48 (13 to 124)	0 (0 to 0)	6450 (3810 to 9680)	1.7 (1 to 2.5)	9970 (1950 to 19600)	2.4 (0.5 to 4.8)	5610 (1460 to 8360)	1.4 (0.4 to 2)	18900 (3280 to 42600)	4.5 (0.8 to 10.1)	62700 (44200 to 80400)	14.4 (10.1 to 18.6)	7060 (1200 to 13500)	1.8 (0.3 to 3.5)
	Female	53900 (49000 to 57100)	11.2 (10.4 to 11.8)	6580 (4290 to 9510)	1.4 (0.9 to 2)	26 (7 to 66)	0 (0 to 0)	2910 (1710 to 4400)	0.7 (0.4 to 1)	4870 (959 to 9560)	1 (0.2 to 2)	3140 (858 to 4670)	0.6 (0.2 to 1)	8530 (1680 to 19600)	1.6 (0.3 to 3.7)	8340 (4840 to 11800)	1.4 (0.8 to 2)	2210 (340 to 4340)	0.5 (0.1 to 1)
Andean Latin America	Both	1630 (1270 to 2020)	3 (2.4 to 3.7)	976 (620 to 1430)	1.8 (1.1 to 2.6)	276 (130 to 471)	0.5 (0.2 to 0.9)	97 (43 to 171)	0.2 (0.1 to 0.3)	253 (28 to 683)	0.5 (0.1 to 1.2)	198 (52 to 320)	0.4 (0.1 to 0.6)	516 (120 to 1170)	1 (0.2 to 2.2)	370 (206 to 585)	0.7 (0.4 to 1.1)	442 (217 to 724)	0.8 (0.4 to 1.2)
	Male	1340 (1040 to 1660)	5.2 (4.1 to 6.4)	552 (348 to 810)	2.1 (1.3 to 3.1)	131 (57 to 236)	0.5 (0.2 to 0.9)	54 (23 to 96)	0.2 (0.1 to 0.4)	141 (15 to 381)	0.5 (0.1 to 1.5)	107 (28 to 174)	0.4 (0.1 to 0.7)	293 (49 to 701)	1.2 (0.2 to 2.7)	277 (143 to 468)	1.1 (0.6 to 1.9)	293 (133 to 495)	1 (0.5 to 1.8)

	Female	295 (225 to 388)	1 (0.8 to 1.4)	423 (268 to 626)	1.5 (0.9 to 2.2)	145 (72 to 253)	0.5 (0.2 to 0.9)	43 (19 to 78)	0.1 (0.1 to 0.3)	113 (13 to 305)	0.4 (0 to 1.1)	91 (24 to 146)	0.3 (0.1 to 0.5)	223 (45 to 537)	0.8 (0.2 to 1.9)	93 (47 to 162)	0.3 (0.2 to 0.6)	149 (69 to 255)	0.5 (0.2 to 0.8)
Caribbean	Both	6920 (5850 to 8060)	13.3 (11.3 to 15.6)	1210 (617 to 2000)	2.3 (1.2 to 3.9)	313 (180 to 506)	0.6 (0.3 to 1)	457 (256 to 705)	0.9 (0.5 to 1.4)	208 (34 to 528)	0.4 (0.1 to 1)	295 (78 to 461)	0.6 (0.2 to 0.9)	1320 (305 to 2860)	2.6 (0.6 to 5.5)	414 (237 to 637)	0.8 (0.5 to 1.2)	620 (302 to 993)	1.2 (0.6 to 1.9)
	Male	5030 (4250 to 5900)	21 (17.7 to 24.6)	792 (405 to 1310)	3.3 (1.7 to 5.4)	205 (108 to 368)	0.8 (0.4 to 1.5)	293 (165 to 461)	1.2 (0.7 to 1.9)	136 (22 to 341)	0.6 (0.1 to 1.4)	188 (48 to 299)	0.8 (0.2 to 1.2)	909 (165 to 2040)	3.8 (0.7 to 8.5)	365 (196 to 577)	1.6 (0.8 to 2.5)	449 (204 to 740)	1.8 (0.8 to 2.9)
	Female	1880 (1560 to 2240)	6.8 (5.6 to 8.1)	418 (209 to 703)	1.5 (0.8 to 2.5)	108 (66 to 163)	0.4 (0.2 to 0.6)	165 (94 to 256)	0.6 (0.3 to 0.9)	71 (11 to 184)	0.3 (0 to 0.7)	107 (27 to 166)	0.4 (0.1 to 0.6)	411 (82 to 964)	1.5 (0.3 to 3.5)	49 (27 to 79)	0.2 (0.1 to 0.3)	171 (78 to 285)	0.6 (0.3 to 1)
Central Latin America	Both	11400 (9520 to 13500)	5 (4.2 to 5.9)	3580 (2450 to 4870)	1.6 (1.1 to 2.1)	982 (559 to 1510)	0.4 (0.2 to 0.7)	746 (361 to 1250)	0.3 (0.2 to 0.5)	1410 (221 to 3130)	0.6 (0.1 to 1.4)	857 (228 to 1330)	0.4 (0.1 to 0.6)	3470 (845 to 7490)	1.5 (0.4 to 3.3)	1400 (881 to 2050)	0.6 (0.4 to 0.9)	1840 (911 to 2920)	0.7 (0.4 to 1.2)
	Male	8600 (7210 to 10100)	8.3 (6.9 to 9.7)	2200 (1500 to 2990)	2.1 (1.4 to 2.8)	491 (261 to 805)	0.5 (0.2 to 0.8)	445 (212 to 749)	0.4 (0.2 to 0.7)	845 (137 to 1890)	0.8 (0.1 to 1.8)	501 (131 to 780)	0.5 (0.1 to 0.7)	2190 (387 to 4930)	2.1 (0.4 to 4.8)	1160 (649 to 1770)	1.1 (0.6 to 1.7)	1350 (629 to 2200)	1.2 (0.5 to 1.9)
	Female	2760 (2210 to 3400)	2.2 (1.8 to 2.7)	1390 (931 to 1950)	1.1 (0.7 to 1.5)	491 (280 to 754)	0.4 (0.2 to 0.6)	301 (148 to 511)	0.2 (0.1 to 0.4)	563 (83 to 1250)	0.4 (0.1 to 1)	356 (95 to 560)	0.3 (0.1 to 0.4)	1280 (263 to 2950)	1 (0.2 to 2.4)	242 (135 to 372)	0.2 (0.1 to 0.3)	487 (227 to 807)	0.4 (0.2 to 0.6)
Tropical Latin America	Both	22600 (20900 to 24200)	9.4 (8.7 to 10.1)	2910 (1870 to 4100)	1.2 (0.8 to 1.7)	843 (363 to 1550)	0.4 (0.2 to 0.6)	1810 (1060 to 2730)	0.7 (0.4 to 1.1)	1560 (273 to 3410)	0.7 (0.1 to 1.4)	915 (249 to 1400)	0.4 (0.1 to 0.6)	3410 (799 to 7360)	1.4 (0.3 to 3.1)	3030 (2040 to 4060)	1.3 (0.9 to 1.8)	2170 (1120 to 3340)	0.8 (0.4 to 1.3)
	Male	14700 (13600 to 15700)	14 (12.9 to 15)	1730 (1110 to 2460)	1.6 (1 to 2.3)	443 (181 to 831)	0.4 (0.2 to 0.8)	1040 (594 to 1600)	1 (0.6 to 1.5)	918 (160 to 1980)	0.9 (0.2 to 1.9)	520 (140 to 814)	0.5 (0.1 to 0.8)	2100 (346 to 4800)	2 (0.3 to 4.6)	2160 (1310 to 3130)	2.2 (1.4 to 3.2)	1540 (740 to 2440)	1.3 (0.6 to 2.1)
	Female	7890 (7130 to 8600)	5.9 (5.3 to 6.5)	1180 (745 to 1670)	0.9 (0.6 to 1.3)	399 (178 to 718)	0.3 (0.1 to 0.5)	768 (452 to 1170)	0.6 (0.3 to 0.9)	646 (112 to 1400)	0.5 (0.1 to 1.1)	395 (102 to 615)	0.3 (0.1 to 0.5)	1310 (250 to 3050)	1 (0.2 to 2.3)	868 (526 to 1270)	0.7 (0.4 to 1)	634 (301 to 1020)	0.5 (0.2 to 0.7)
North Africa and	Both	47800 (41900	11.6 (10.2	14800 (10900	3.6 (2.6	791 (438	0.2 (0.1	4840 (2920	1.2 (0.7	2690 (439	0.7 (0.1	1240 (375	0.3 (0.1	7460 (1600	1.9 (0.4	6040 (3420	1.6 (0.9	4160 (1990	0.9 (0.4

Middle East		to 54500)	to 13.2)	to 19200)	to 4.6)	to 1340)	to 0.3)	to 7370)	to 1.8)	to 5450)	to 1.3)	to 1860)	to 0.4)	to 16400)	to 4.2)	to 9510)	to 2.5)	to 6710)	to 1.4)
	Male	44000 (38600 to 50300)	21.1 (18.6 to 24)	11600 (8440 to 15100)	5.5 (4 to 7.1)	581 (303 to 1080)	0.3 (0.1 to 0.5)	3470 (2080 to 5350)	1.7 (1 to 2.5)	2130 (346 to 4340)	1 (0.2 to 2.1)	929 (277 to 1400)	0.4 (0.1 to 0.7)	5900 (975 to 13600)	3 (0.5 to 6.9)	5590 (2980 to 9030)	3 (1.6 to 4.6)	3920 (1850 to 6360)	1.6 (0.7 to 2.5)
	Female	3730 (3110 to 4410)	1.9 (1.6 to 2.2)	3220 (2350 to 4160)	1.6 (1.2 to 2.1)	210 (128 to 316)	0.1 (0.1 to 0.1)	1370 (811 to 2040)	0.7 (0.4 to 1)	554 (91 to 1140)	0.3 (0 to 0.6)	306 (93 to 472)	0.1 (0 to 0.2)	1560 (323 to 3710)	0.8 (0.2 to 1.9)	451 (242 to 747)	0.3 (0.1 to 0.4)	238 (107 to 399)	0.1 (0 to 0.2)
South Asia	Both	58400 (48500 to 68500)	4.4 (3.6 to 5.1)	24100 (16600 to 32200)	1.8 (1.2 to 2.4)	13900 (8320 to 20400)	1 (0.6 to 1.5)	5640 (3070 to 8850)	0.4 (0.2 to 0.7)	5300 (1070 to 11200)	0.4 (0.1 to 0.8)	7580 (2740 to 11600)	0.6 (0.2 to 0.8)	11400 (2480 to 25600)	0.9 (0.2 to 1.9)	5810 (3470 to 9040)	0.5 (0.3 to 0.7)	8090 (3840 to 12900)	0.5 (0.2 to 0.8)
	Male	53400 (43900 to 63300)	8.2 (6.7 to 9.7)	18100 (12100 to 24500)	2.7 (1.8 to 3.7)	9570 (5390 to 14500)	1.4 (0.8 to 2.2)	3560 (1860 to 5750)	0.5 (0.3 to 0.9)	3860 (740 to 8120)	0.6 (0.1 to 1.2)	5480 (1940 to 8620)	0.8 (0.3 to 1.3)	8540 (1490 to 20100)	1.3 (0.2 to 3.1)	5150 (2870 to 8220)	0.9 (0.5 to 1.4)	7220 (3360 to 11700)	0.9 (0.4 to 1.5)
	Female	4970 (3890 to 6210)	0.8 (0.6 to 0.9)	6080 (4070 to 8300)	0.9 (0.6 to 1.2)	4320 (2600 to 6370)	0.6 (0.4 to 0.9)	2080 (1120 to 3270)	0.3 (0.2 to 0.5)	1450 (297 to 3020)	0.2 (0 to 0.4)	2100 (804 to 3310)	0.3 (0.1 to 0.5)	2850 (572 to 6910)	0.4 (0.1 to 1)	658 (338 to 1160)	0.1 (0.1 to 0.2)	876 (375 to 1480)	0.1 (0 to 0.2)
East Asia	Both	504000 (411000 to 605000)	24.6 (20.2 to 29.4)	175000 (125000 to 232000)	8.6 (6.1 to 11.3)	39100 (18700 to 68400)	1.9 (0.9 to 3.3)	60600 (36400 to 90200)	3 (1.8 to 4.5)	29100 (5300 to 61700)	1.4 (0.3 to 3.1)	27900 (7310 to 44000)	1.4 (0.4 to 2.2)	54700 (12200 to 124000)	2.7 (0.6 to 6.2)	24000 (15000 to 36000)	1.3 (0.8 to 1.9)	42800 (20400 to 70000)	1.9 (0.9 to 3)
	Male	441000 (350000 to 544000)	47.1 (37.8 to 57.5)	122000 (83600 to 168000)	13.1 (9.1 to 17.8)	24000 (10700 to 44900)	2.5 (1.1 to 4.7)	32800 (18800 to 51300)	3.5 (2 to 5.5)	19900 (3670 to 42500)	2.1 (0.4 to 4.5)	19400 (5080 to 32200)	2.1 (0.5 to 3.5)	38300 (6370 to 93700)	4.2 (0.7 to 10.2)	18700 (10200 to 30700)	2.5 (1.4 to 4)	29800 (13500 to 50100)	2.6 (1.2 to 4.4)
	Female	62500 (50100 to 77800)	5.9 (4.8 to 7.4)	52200 (36700 to 70400)	4.9 (3.4 to 6.6)	15000 (7290 to 26000)	1.4 (0.7 to 2.4)	27800 (16500 to 42200)	2.6 (1.5 to 3.9)	9220 (1570 to 19100)	0.9 (0.1 to 1.8)	8470 (2270 to 13300)	0.8 (0.2 to 1.3)	16400 (3040 to 40300)	1.5 (0.3 to 3.8)	5250 (2730 to 8090)	0.5 (0.3 to 0.8)	13000 (5620 to 21900)	1.1 (0.5 to 1.9)
Oceania	Both	766 (567 to 1100)	12.1 (9.2 to 17)	56 (18 to 134)	0.9 (0.3 to 2.1)	357 (222 to 572)	5.4 (3.5 to 8.5)	80 (40 to 141)	1.2 (0.6 to 2.2)	48 (5 to 149)	0.7 (0.1 to 2.3)	71 (22 to 124)	1.1 (0.3 to 1.9)	190 (39 to 442)	3.1 (0.7 to 7.1)	84 (43 to 150)	1.5 (0.8 to 2.7)	61 (28 to 108)	0.7 (0.3 to 1.3)

	Male	657 (474 to 969)	20.5 (15.4 to 29.3)	46 (14 to 111)	1.4 (0.4 to 3.3)	283 (170 to 466)	8.5 (5.1 to 13.8)	58 (28 to 106)	1.8 (0.9 to 3.3)	38 (4 to 122)	1.2 (0.1 to 3.6)	56 (17 to 101)	1.7 (0.5 to 3)	153 (27 to 375)	4.9 (0.9 to 11.6)	80 (40 to 146)	3 (1.7 to 5.3)	46 (19 to 85)	1 (0.4 to 1.9)
	Female	109 (78 to 153)	3.6 (2.6 to 5)	10 (4 to 24)	0.3 (0.1 to 0.8)	75 (46 to 119)	2.3 (1.4 to 3.7)	22 (11 to 39)	0.7 (0.3 to 1.2)	10 (1 to 30)	0.3 (0 to 0.9)	15 (5 to 27)	0.5 (0.1 to 0.8)	38 (8 to 93)	1.3 (0.3 to 3.1)	3 (1 to 6)	0.1 (0 to 0.2)	15 (6 to 27)	0.4 (0.2 to 0.7)
Southeast Asia	Both	76400 (64900 to 88400)	13.2 (11.3 to 15.3)	16200 (11100 to 22300)	2.8 (1.9 to 3.8)	12000 (6930 to 18300)	2 (1.1 to 3.1)	6980 (3820 to 11000)	1.2 (0.7 to 1.9)	2630 (477 to 6010)	0.4 (0.1 to 1)	5570 (1690 to 8680)	0.9 (0.3 to 1.5)	10800 (2430 to 24400)	2 (0.5 to 4.5)	4270 (2500 to 6590)	0.8 (0.5 to 1.3)	9060 (4500 to 14400)	1.3 (0.6 to 2.1)
	Male	68600 (58000 to 79300)	26.5 (22.6 to 30.5)	11400 (7840 to 15500)	4.4 (3 to 5.9)	7620 (4120 to 12100)	2.8 (1.6 to 4.5)	3560 (1950 to 5710)	1.4 (0.8 to 2.2)	1810 (326 to 4030)	0.7 (0.1 to 1.5)	3800 (1140 to 5900)	1.4 (0.4 to 2.2)	7420 (1250 to 17900)	3.1 (0.5 to 7.4)	3960 (2160 to 6220)	1.9 (1 to 2.9)	6840 (3210 to 11200)	2.1 (1 to 3.4)
	Female	7860 (6040 to 9990)	2.7 (2.1 to 3.3)	4810 (3100 to 7090)	1.5 (1 to 2.3)	4360 (2450 to 6830)	1.4 (0.8 to 2.1)	3420 (1770 to 5640)	1.1 (0.5 to 1.7)	824 (149 to 2000)	0.3 (0 to 0.6)	1770 (545 to 2970)	0.6 (0.2 to 0.9)	3400 (678 to 8400)	1.1 (0.2 to 2.8)	311 (173 to 534)	0.1 (0.1 to 0.2)	2220 (976 to 3790)	0.6 (0.3 to 1)
Central Sub-Saharan Africa	Both	2270 (1450 to 4070)	4.5 (2.9 to 7.9)	583 (269 to 1180)	1.1 (0.5 to 2.3)	1600 (813 to 3130)	3.1 (1.6 to 5.9)	112 (50 to 212)	0.2 (0.1 to 0.4)	262 (31 to 839)	0.5 (0.1 to 1.6)	343 (93 to 741)	0.7 (0.2 to 1.4)	565 (102 to 1630)	1.2 (0.2 to 3.5)	246 (72 to 929)	0.6 (0.2 to 2.3)	374 (145 to 791)	0.6 (0.2 to 1.2)
	Male	2160 (1350 to 3950)	10 (6.4 to 17.8)	463 (200 to 990)	2.1 (0.9 to 4.4)	1200 (548 to 2680)	5.3 (2.4 to 11.6)	79 (33 to 163)	0.3 (0.1 to 0.6)	200 (22 to 697)	0.9 (0.1 to 3.1)	261 (68 to 635)	1.2 (0.3 to 2.8)	460 (67 to 1450)	2.3 (0.4 to 7.3)	190 (44 to 844)	1.2 (0.3 to 5.4)	294 (103 to 675)	0.9 (0.3 to 2.2)
	Female	118 (83 to 168)	0.4 (0.3 to 0.6)	120 (59 to 213)	0.4 (0.2 to 0.8)	405 (252 to 644)	1.5 (0.9 to 2.4)	34 (16 to 58)	0.1 (0.1 to 0.2)	62 (8 to 191)	0.2 (0 to 0.7)	82 (24 to 139)	0.3 (0.1 to 0.5)	105 (19 to 257)	0.4 (0.1 to 1)	56 (14 to 117)	0.2 (0.1 to 0.5)	80 (34 to 147)	0.2 (0.1 to 0.4)
Eastern sub-Saharan Africa	Both	3810 (3080 to 4830)	2.7 (2.2 to 3.3)	638 (316 to 1070)	0.4 (0.2 to 0.7)	3280 (2240 to 4750)	2.2 (1.5 to 3.1)	218 (103 to 387)	0.1 (0.1 to 0.2)	450 (81 to 1030)	0.3 (0.1 to 0.7)	536 (192 to 878)	0.4 (0.1 to 0.6)	664 (137 to 1520)	0.5 (0.1 to 1.1)	517 (167 to 2130)	0.4 (0.1 to 1.7)	708 (341 to 1180)	0.4 (0.2 to 0.6)
	Male	3450 (2780 to 4410)	5.2 (4.2 to 6.5)	494 (234 to 839)	0.7 (0.3 to 1.2)	2330 (1550 to 3380)	3.4 (2.3 to 4.9)	140 (64 to 250)	0.2 (0.1 to 0.3)	326 (59 to 778)	0.5 (0.1 to 1.1)	393 (141 to 659)	0.6 (0.2 to 0.9)	514 (82 to 1240)	0.8 (0.1 to 2)	403 (103 to 2000)	0.7 (0.2 to 3.4)	529 (241 to 904)	0.6 (0.3 to 1)
	Female	358 (279 to 447)	0.5 (0.4	144 (72 to 246)	0.2 (0.1	954 (665	1.2 (0.8	78 (38 to 129)	0.1 (0 to 0.1)	124 (23 to 275)	0.2 (0 to 0.3)	144 (50 to 231)	0.2 (0.1	149 (27 to 361)	0.2 (0 to 0.5)	114 (31 to 204)	0.2 (0 to 0.3)	179 (81 to 298)	0.2 (0.1

			to 0.6)		to 0.3)	to 1350)	to 1.7)						to 0.3)						to 0.3)
Southern sub-Saharan Africa	Both	5630 (5030 to 6390)	10.4 (9.3 to 11.7)	1590 (1120 to 2190)	2.9 (2.1 to 4)	497 (299 to 742)	0.9 (0.5 to 1.3)	387 (204 to 621)	0.7 (0.4 to 1.1)	477 (81 to 1110)	0.9 (0.1 to 2.1)	608 (211 to 936)	1.1 (0.4 to 1.7)	979 (237 to 2110)	1.9 (0.5 to 4)	1680 (1170 to 2260)	3.4 (2.3 to 4.5)	228 (111 to 370)	0.4 (0.2 to 0.6)
	Male	4400 (3880 to 5050)	19.7 (17.6 to 22.4)	1080 (748 to 1510)	4.8 (3.3 to 6.7)	295 (169 to 463)	1.3 (0.7 to 2)	223 (117 to 366)	1 (0.5 to 1.6)	318 (53 to 759)	1.4 (0.2 to 3.3)	404 (140 to 639)	1.8 (0.6 to 2.8)	613 (105 to 1400)	2.9 (0.5 to 6.7)	1460 (961 to 2000)	7.6 (5.1 to 10.3)	175 (80 to 289)	0.6 (0.3 to 1)
	Female	1230 (1050 to 1410)	4 (3.4 to 4.6)	506 (347 to 689)	1.6 (1.1 to 2.2)	202 (124 to 291)	0.6 (0.4 to 0.9)	164 (87 to 261)	0.5 (0.3 to 0.8)	159 (29 to 376)	0.5 (0.1 to 1.2)	204 (72 to 310)	0.7 (0.2 to 1)	366 (75 to 832)	1.2 (0.2 to 2.7)	221 (103 to 324)	0.8 (0.4 to 1.1)	54 (24 to 89)	0.2 (0.1 to 0.3)
Western sub-Saharan Africa	Both	4830 (3920 to 5860)	2.9 (2.4 to 3.5)	2130 (1210 to 3160)	1.3 (0.7 to 1.9)	3590 (2330 to 5130)	2.1 (1.4 to 3)	466 (247 to 741)	0.3 (0.1 to 0.4)	766 (146 to 1570)	0.5 (0.1 to 0.9)	775 (254 to 1220)	0.5 (0.2 to 0.7)	1100 (233 to 2540)	0.7 (0.2 to 1.6)	351 (183 to 713)	0.2 (0.1 to 0.5)	645 (310 to 1060)	0.3 (0.1 to 0.5)
	Male	4520 (3650 to 5530)	5.6 (4.6 to 6.8)	1570 (855 to 2340)	2 (1.1 to 2.9)	2560 (1620 to 3830)	3.2 (2 to 4.7)	326 (173 to 520)	0.4 (0.2 to 0.6)	551 (103 to 1130)	0.7 (0.1 to 1.4)	558 (184 to 874)	0.7 (0.2 to 1.1)	815 (129 to 2000)	1.1 (0.2 to 2.7)	301 (145 to 657)	0.4 (0.2 to 0.9)	484 (219 to 812)	0.5 (0.2 to 0.8)
	Female	306 (239 to 391)	0.4 (0.3 to 0.5)	561 (318 to 850)	0.6 (0.4 to 1)	1020 (683 to 1470)	1.1 (0.8 to 1.6)	139 (73 to 221)	0.1 (0.1 to 0.2)	215 (41 to 442)	0.2 (0 to 0.5)	216 (73 to 344)	0.2 (0.1 to 0.4)	289 (53 to 692)	0.4 (0.1 to 0.9)	50 (22 to 84)	0.1 (0 to 0.1)	160 (73 to 271)	0.1 (0.1 to 0.2)
Afghanistan	Both	651 (372 to 1120)	5.8 (3.5 to 9.7)	130 (45 to 290)	1.1 (0.4 to 2.4)	358 (178 to 628)	3 (1.6 to 5.3)	93 (43 to 173)	0.8 (0.4 to 1.5)	76 (6 to 272)	0.6 (0.1 to 2.3)	79 (24 to 154)	0.7 (0.2 to 1.3)	158 (31 to 419)	1.5 (0.3 to 3.9)	22 (5 to 98)	0.2 (0.1 to 1)	99 (32 to 220)	0.6 (0.2 to 1.4)
	Male	616 (347 to 1080)	11.7 (6.9 to 20)	105 (34 to 244)	1.9 (0.7 to 4.4)	263 (123 to 507)	4.8 (2.3 to 9.1)	65 (28 to 136)	1.2 (0.6 to 2.6)	58 (4 to 209)	1 (0.1 to 3.9)	60 (17 to 125)	1.1 (0.3 to 2.2)	119 (18 to 337)	2.5 (0.4 to 6.8)	19 (3 to 92)	0.4 (0.1 to 2.1)	92 (29 to 209)	1.3 (0.4 to 2.8)
	Female	36 (22 to 55)	0.6 (0.4 to 0.9)	25 (9 to 52)	0.4 (0.1 to 0.7)	94 (53 to 151)	1.4 (0.9 to 2.2)	27 (13 to 47)	0.4 (0.2 to 0.6)	18 (1 to 65)	0.3 (0 to 1)	19 (6 to 34)	0.3 (0.1 to 0.5)	39 (8 to 97)	0.7 (0.1 to 1.6)	4 (1 to 11)	0.1 (0 to 0.2)	7 (2 to 15)	0.1 (0 to 0.2)
Albania	Both	872 (642 to 1170)	19.8 (14.5 to 26.6)	140 (88 to 207)	3.2 (2 to 4.8)	60 (23 to 124)	1.4 (0.5 to 2.8)	88 (48 to 144)	2 (1.1 to 3.3)	129 (20 to 289)	3 (0.5 to 6.7)	14 (4 to 26)	0.3 (0.1 to 0.6)	74 (14 to 186)	1.7 (0.3 to 4.2)	34 (12 to 94)	0.7 (0.3 to 2.1)	37 (6 to 77)	0.8 (0.1 to 1.7)

	Male	782 (565 to 1060)	37.7 (27.4 to 50.5)	113 (71 to 170)	5.5 (3.5 to 8.2)	44 (15 to 94)	2.1 (0.8 to 4.5)	64 (35 to 106)	3.1 (1.7 to 5.1)	103 (16 to 230)	5 (0.8 to 11.2)	11 (3 to 21)	0.6 (0.1 to 1)	61 (10 to 160)	2.9 (0.5 to 7.6)	27 (9 to 88)	1.3 (0.4 to 4.1)	32 (6 to 67)	1.5 (0.3 to 3.1)
	Female	91 (67 to 122)	3.9 (2.9 to 5.2)	27 (16 to 39)	1.2 (0.7 to 1.7)	16 (6 to 29)	0.7 (0.3 to 1.3)	24 (14 to 38)	1.1 (0.6 to 1.7)	26 (4 to 57)	1.2 (0.2 to 2.5)	4 (1 to 6)	0.2 (0 to 0.3)	13 (2 to 34)	0.6 (0.1 to 1.4)	6 (2 to 13)	0.3 (0.1 to 0.6)	5 (1 to 11)	0.2 (0 to 0.5)
Algeria	Both	2080 (1570 to 2750)	6.7 (5.2 to 8.9)	679 (421 to 1010)	2.2 (1.3 to 3.1)	2 (1 to 4)	0 (0 to 0)	281 (160 to 446)	0.9 (0.5 to 1.4)	109 (10 to 369)	0.3 (0 to 1.2)	87 (19 to 148)	0.3 (0.1 to 0.5)	398 (89 to 890)	1.3 (0.3 to 3)	24 (5 to 102)	0.1 (0 to 0.4)	155 (66 to 280)	0.4 (0.2 to 0.7)
	Male	1990 (1490 to 2650)	12.4 (9.4 to 16.4)	534 (321 to 806)	3.3 (2 to 4.9)	1 (0 to 3)	0 (0 to 0)	198 (108 to 320)	1.2 (0.7 to 2)	85 (8 to 298)	0.5 (0 to 1.8)	67 (14 to 119)	0.4 (0.1 to 0.7)	313 (59 to 735)	2 (0.4 to 4.7)	20 (2 to 96)	0.2 (0 to 0.7)	148 (62 to 269)	0.8 (0.3 to 1.4)
	Female	86 (61 to 119)	0.6 (0.4 to 0.9)	145 (93 to 213)	0.9 (0.6 to 1.4)	1 (0 to 1)	0 (0 to 0)	82 (48 to 124)	0.5 (0.3 to 0.8)	23 (2 to 77)	0.2 (0 to 0.5)	20 (5 to 33)	0.1 (0 to 0.2)	85 (17 to 194)	0.6 (0.1 to 1.3)	4 (2 to 9)	0 (0 to 0.1)	7 (3 to 13)	0 (0 to 0.1)
American Samoa	Both	8 (6 to 9)	16.7 (14.2 to 19.2)	0 (0 to 1)	1 (0.4 to 2.1)	0 (0 to 1)	0.7 (0.2 to 1.8)	1 (0 to 1)	1.7 (1 to 2.7)	0 (0 to 1)	0.8 (0.1 to 3)	0 (0 to 1)	1.1 (0.3 to 1.7)	3 (1 to 5)	5.9 (1.6 to 11.7)	1 (1 to 1)	2.3 (1.4 to 3.4)	1 (0 to 1)	1 (0.5 to 1.7)
	Male	6 (5 to 7)	27.8 (23.9 to 32)	0 (0 to 1)	1.5 (0.5 to 3.2)	0 (0 to 1)	0.9 (0.2 to 2.4)	1 (0 to 1)	2.4 (1.3 to 3.8)	0 (0 to 1)	1.1 (0.1 to 4.5)	0 (0 to 1)	1.6 (0.4 to 2.5)	2 (0 to 4)	8.7 (1.8 to 18.1)	1 (1 to 1)	4.6 (2.8 to 7)	0 (0 to 1)	1.5 (0.6 to 2.5)
	Female	2 (1 to 2)	6.9 (5.3 to 9)	0 (0 to 0)	0.6 (0.2 to 1.3)	0 (0 to 0)	0.6 (0.1 to 1.3)	0 (0 to 0)	1.1 (0.6 to 1.8)	0 (0 to 0)	0.5 (0 to 1.8)	0 (0 to 0)	0.7 (0.2 to 1.1)	1 (0 to 2)	3.5 (0.8 to 7.6)	0 (0 to 0)	0.3 (0.2 to 0.6)	0 (0 to 0)	0.6 (0.3 to 1.2)
Andorra	Both	34 (26 to 43)	24.2 (18.4 to 30.7)	3 (1 to 4)	1.9 (0.9 to 3.1)	0 (0 to 0)	0 (0 to 0)	2 (1 to 3)	1.5 (0.8 to 2.3)	3 (0 to 10)	1.9 (0.2 to 6.8)	1 (0 to 2)	0.9 (0.2 to 1.6)	4 (1 to 9)	2.9 (0.6 to 6.6)	16 (10 to 23)	11.4 (7 to 16.4)	2 (0 to 4)	1.4 (0.2 to 2.9)
	Male	29 (22 to 36)	40.8 (31.3 to 50.8)	2 (1 to 4)	3 (1.5 to 5)	0 (0 to 0)	0 (0 to 0)	2 (1 to 3)	2.3 (1.3 to 3.6)	2 (0 to 8)	3.1 (0.3 to 11)	1 (0 to 2)	1.5 (0.4 to 2.5)	3 (1 to 8)	4.8 (0.8 to 11.3)	15 (9 to 22)	22.3 (13.8 to 32.1)	2 (0 to 4)	2.4 (0.4 to 4.9)
	Female	5 (4 to 8)	7.7 (5.3	1 (0 to 1)	0.7 (0.3	0 (0 to 0)	0 (0 to 0)	0 (0 to 1)	0.6 (0.3 to 1)	1 (0 to 2)	0.8 (0.1	0 (0 to 0)	0.4 (0.1	1 (0 to 2)	0.9 (0.2	1 (0 to 1)	0.9 (0.3	0 (0 to 1)	0.4 (0 to 0.8)

			to 10.9)		to 1.3)						to 2.7)		to 0.7)		to 2.4)		to 1.9)		
Angola	Both	708 (547 to 919)	6.8 (5.5 to 8.6)	169 (79 to 295)	1.6 (0.8 to 2.8)	226 (131 to 344)	2.1 (1.3 to 3.2)	46 (23 to 78)	0.4 (0.2 to 0.7)	67 (4 to 256)	0.6 (0 to 2.4)	69 (18 to 116)	0.7 (0.2 to 1.1)	128 (26 to 298)	1.3 (0.3 to 3.1)	57 (20 to 229)	0.7 (0.3 to 2.7)	96 (42 to 172)	0.7 (0.3 to 1.2)
	Male	658 (509 to 856)	14.5 (11.7 to 18.2)	135 (62 to 235)	2.9 (1.3 to 4.9)	163 (92 to 256)	3.5 (2 to 5.4)	30 (15 to 52)	0.6 (0.3 to 1)	51 (3 to 194)	1.1 (0.1 to 4.1)	52 (14 to 87)	1.1 (0.3 to 1.8)	105 (16 to 252)	2.5 (0.4 to 5.8)	45 (12 to 220)	1.3 (0.4 to 6)	74 (31 to 135)	1.1 (0.5 to 2.1)
	Female	49 (32 to 72)	0.9 (0.6 to 1.3)	34 (15 to 62)	0.6 (0.3 to 1.1)	63 (39 to 96)	1.1 (0.7 to 1.7)	16 (8 to 26)	0.2 (0.1 to 0.4)	16 (1 to 62)	0.3 (0 to 1.1)	17 (5 to 30)	0.3 (0.1 to 0.5)	23 (4 to 58)	0.4 (0.1 to 1.1)	12 (3 to 24)	0.3 (0.1 to 0.6)	22 (9 to 42)	0.3 (0.1 to 0.5)
Antigua and Barbuda	Both	4 (3 to 5)	4.2 (3.5 to 5.1)	1 (0 to 2)	1.2 (0.4 to 2.2)	0 (0 to 0)	0 (0 to 0)	0 (0 to 0)	0.3 (0.2 to 0.5)	0 (0 to 1)	0.3 (0 to 0.9)	0 (0 to 0)	0.3 (0.1 to 0.4)	1 (0 to 3)	1.3 (0.3 to 2.8)	0 (0 to 0)	0.2 (0.1 to 0.4)	1 (0 to 1)	0.6 (0.3 to 1)
	Male	3 (3 to 4)	7 (5.8 to 8.4)	1 (0 to 1)	1.6 (0.6 to 3)	0 (0 to 0)	0 (0 to 0.1)	0 (0 to 0)	0.4 (0.2 to 0.7)	0 (0 to 1)	0.3 (0 to 1.3)	0 (0 to 0)	0.4 (0.1 to 0.6)	1 (0 to 2)	1.8 (0.3 to 4.2)	0 (0 to 0)	0.5 (0.3 to 0.8)	0 (0 to 1)	0.9 (0.4 to 1.5)
	Female	1 (1 to 1)	1.8 (1.4 to 2.4)	0 (0 to 1)	0.8 (0.3 to 1.5)	0 (0 to 0)	0 (0 to 0)	0 (0 to 0)	0.2 (0.1 to 0.3)	0 (0 to 0)	0.2 (0 to 0.7)	0 (0 to 0)	0.2 (0 to 0.3)	0 (0 to 1)	0.9 (0.2 to 2)	0 (0 to 0)	0.1 (0 to 0.1)	0 (0 to 0)	0.3 (0.1 to 0.5)
Argentina	Both	9370 (8700 to 10000)	17.3 (16.1 to 18.6)	1280 (755 to 1940)	2.4 (1.4 to 3.6)	68 (22 to 167)	0.1 (0 to 0.3)	724 (421 to 1130)	1.3 (0.8 to 2.1)	377 (30 to 1350)	0.7 (0.1 to 2.5)	278 (64 to 431)	0.5 (0.1 to 0.8)	1300 (298 to 2820)	2.4 (0.5 to 5.1)	1550 (963 to 2240)	2.8 (1.7 to 4)	941 (467 to 1500)	1.8 (0.9 to 2.8)
	Male	6710 (6200 to 7230)	28.2 (26.1 to 30.4)	878 (514 to 1320)	3.7 (2.2 to 5.6)	40 (12 to 99)	0.2 (0.1 to 0.4)	489 (282 to 775)	2.1 (1.2 to 3.3)	257 (20 to 909)	1.1 (0.1 to 3.9)	179 (39 to 292)	0.8 (0.2 to 1.3)	917 (155 to 2120)	3.9 (0.7 to 9)	1330 (780 to 1970)	5.8 (3.4 to 8.5)	776 (368 to 1270)	3.1 (1.5 to 5.1)
	Female	2650 (2380 to 2950)	8.8 (7.9 to 9.7)	407 (239 to 616)	1.3 (0.8 to 2)	28 (9 to 68)	0.1 (0 to 0.2)	235 (138 to 363)	0.8 (0.5 to 1.2)	120 (10 to 411)	0.4 (0 to 1.4)	100 (21 to 158)	0.3 (0.1 to 0.5)	383 (73 to 887)	1.2 (0.2 to 2.8)	219 (109 to 338)	0.7 (0.3 to 1)	165 (75 to 277)	0.6 (0.3 to 1)
Armenia	Both	960 (803 to 1140)	22.5 (18.9 to 26.6)	272 (176 to 383)	6.4 (4.2 to 9.1)	6 (2 to 15)	0.1 (0 to 0.4)	90 (53 to 136)	2.1 (1.3 to 3.3)	142 (17 to 404)	3.4 (0.4 to 9.6)	29 (7 to 49)	0.7 (0.2 to 1.2)	116 (23 to 270)	2.7 (0.5 to 6.3)	148 (86 to 223)	3.5 (2.1 to 5.3)	66 (31 to 109)	1.5 (0.7 to 2.4)

	Male	932 (779 to 1110)	51.7 (43.5 to 61)	222 (144 to 313)	12.4 (8 to 17.4)	5 (1 to 11)	0.3 (0.1 to 0.6)	63 (37 to 98)	3.5 (2 to 5.4)	116 (14 to 331)	6.5 (0.8 to 18.3)	23 (5 to 40)	1.3 (0.3 to 2.2)	94 (15 to 224)	5.2 (0.8 to 12.5)	124 (69 to 194)	7.3 (4.1 to 11.2)	59 (27 to 100)	3 (1.3 to 5)
	Female	28 (21 to 38)	1.1 (0.9 to 1.5)	49 (31 to 68)	2.1 (1.3 to 2.9)	2 (1 to 4)	0.1 (0 to 0.2)	26 (16 to 39)	1.1 (0.7 to 1.7)	26 (3 to 73)	1.1 (0.1 to 3.1)	6 (1 to 9)	0.2 (0.1 to 0.4)	22 (4 to 52)	0.9 (0.2 to 2.1)	24 (9 to 44)	0.9 (0.4 to 1.8)	7 (3 to 11)	0.3 (0.1 to 0.5)
Australia	Both	5650 (5120 to 6120)	13.3 (12.2 to 14.4)	309 (78 to 602)	0.7 (0.2 to 1.4)	2 (0 to 8)	0 (0 to 0)	291 (166 to 442)	0.7 (0.4 to 1.1)	110 (13 to 340)	0.3 (0 to 0.8)	422 (109 to 643)	1 (0.3 to 1.5)	832 (191 to 1830)	1.9 (0.4 to 4.1)	3310 (2500 to 4070)	7.3 (5.5 to 9)	375 (67 to 725)	0.9 (0.2 to 1.8)
	Male	3510 (3180 to 3830)	17.6 (16 to 19.2)	185 (46 to 359)	0.9 (0.2 to 1.8)	1 (0 to 4)	0 (0 to 0)	185 (104 to 286)	1 (0.5 to 1.5)	66 (8 to 202)	0.3 (0 to 1)	251 (62 to 381)	1.3 (0.3 to 1.9)	544 (95 to 1280)	2.7 (0.5 to 6.2)	2750 (2020 to 3430)	13.3 (9.7 to 16.6)	254 (42 to 496)	1.3 (0.2 to 2.5)
	Female	2140 (1880 to 2390)	9.6 (8.5 to 10.6)	124 (32 to 246)	0.5 (0.1 to 1.1)	1 (0 to 4)	0 (0 to 0)	106 (61 to 164)	0.5 (0.3 to 0.8)	44 (5 to 136)	0.2 (0 to 0.6)	172 (46 to 266)	0.8 (0.2 to 1.2)	288 (54 to 687)	1.2 (0.2 to 2.9)	563 (326 to 828)	2.3 (1.3 to 3.3)	121 (19 to 241)	0.6 (0.1 to 1.2)
Austria	Both	3010 (2800 to 3190)	17.5 (16.3 to 18.5)	352 (231 to 501)	2 (1.3 to 2.9)	1 (0 to 4)	0 (0 to 0)	232 (139 to 340)	1.4 (0.8 to 2)	398 (68 to 830)	2.3 (0.4 to 4.8)	99 (22 to 152)	0.6 (0.1 to 0.9)	361 (82 to 796)	2 (0.5 to 4.4)	751 (488 to 1020)	4 (2.6 to 5.4)	175 (31 to 340)	1.1 (0.2 to 2.1)
	Male	2010 (1860 to 2150)	25.6 (23.7 to 27.3)	219 (145 to 314)	2.8 (1.8 to 4)	1 (0 to 2)	0 (0 to 0)	156 (91 to 229)	2 (1.2 to 3)	248 (42 to 514)	3.2 (0.5 to 6.6)	60 (12 to 95)	0.8 (0.2 to 1.2)	238 (39 to 556)	3 (0.5 to 6.9)	672 (428 to 942)	8.2 (5.2 to 11.4)	126 (21 to 246)	1.6 (0.3 to 3.2)
	Female	997 (894 to 1100)	10.9 (9.9 to 11.9)	133 (86 to 188)	1.4 (0.9 to 2)	1 (0 to 2)	0 (0 to 0)	77 (46 to 115)	0.9 (0.5 to 1.3)	150 (25 to 316)	1.6 (0.3 to 3.4)	39 (9 to 62)	0.4 (0.1 to 0.7)	123 (23 to 289)	1.2 (0.2 to 2.9)	79 (44 to 121)	0.7 (0.4 to 1.1)	50 (8 to 100)	0.6 (0.1 to 1.2)
Azerbaijan	Both	1520 (1050 to 2010)	15.3 (10.7 to 19.9)	376 (199 to 570)	3.9 (2.1 to 5.9)	28 (10 to 63)	0.3 (0.1 to 0.7)	166 (96 to 257)	1.7 (1 to 2.6)	138 (13 to 514)	1.4 (0.1 to 5.2)	68 (15 to 115)	0.7 (0.2 to 1.2)	173 (36 to 403)	1.9 (0.4 to 4.3)	39 (15 to 73)	0.5 (0.2 to 0.9)	146 (65 to 260)	1.2 (0.5 to 2.1)
	Male	1500 (1030 to 1990)	34.1 (23.6 to 44.3)	304 (157 to 475)	6.8 (3.6 to 10.6)	21 (7 to 48)	0.5 (0.2 to 1.1)	112 (59 to 181)	2.6 (1.4 to 4.1)	111 (10 to 405)	2.5 (0.2 to 9.2)	54 (12 to 96)	1.2 (0.3 to 2.1)	138 (23 to 335)	3.3 (0.5 to 8)	32 (10 to 64)	0.9 (0.3 to 1.7)	125 (52 to 230)	2.2 (0.9 to 4)
	Female	26 (17 to 38)	0.5 (0.3	71 (39 to 112)	1.5 (0.8	7 (3 to 16)	0.2 (0.1	54 (32 to 82)	1.1 (0.6	27 (3 to 97)	0.5 (0.1 to 2)	14 (3 to 23)	0.3 (0.1	35 (7 to 86)	0.8 (0.1	7 (3 to 13)	0.2 (0.1	21 (9 to 40)	0.3 (0.1

			to 0.7)		to 2.3)		to 0.3)		to 1.6)				to 0.5)		to 1.9)		to 0.3)		to 0.6)
Bahamas	Both	23 (18 to 29)	6 (4.8 to 7.5)	6 (2 to 12)	1.6 (0.4 to 3.1)	0 (0 to 0)	0 (0 to 0.1)	2 (1 to 3)	0.5 (0.3 to 0.8)	2 (0 to 6)	0.4 (0 to 1.5)	2 (1 to 3)	0.5 (0.1 to 0.8)	6 (1 to 14)	1.7 (0.4 to 3.7)	3 (2 to 5)	0.8 (0.5 to 1.4)	4 (2 to 6)	0.9 (0.4 to 1.5)
	Male	20 (15 to 25)	11.4 (9.1 to 14.3)	4 (1 to 8)	2.4 (0.7 to 4.8)	0 (0 to 0)	0 (0 to 0.1)	1 (1 to 2)	0.8 (0.5 to 1.3)	1 (0 to 4)	0.6 (0 to 2.4)	1 (0 to 2)	0.8 (0.2 to 1.3)	4 (1 to 10)	2.8 (0.5 to 6.4)	3 (1 to 5)	1.9 (1 to 3.1)	3 (1 to 5)	1.5 (0.7 to 2.6)
	Female	3 (3 to 5)	1.7 (1.2 to 2.2)	2 (1 to 4)	0.9 (0.2 to 1.7)	0 (0 to 0)	0 (0 to 0.1)	1 (0 to 1)	0.3 (0.2 to 0.5)	0 (0 to 2)	0.2 (0 to 0.8)	1 (0 to 1)	0.3 (0.1 to 0.5)	2 (0 to 4)	0.9 (0.2 to 2.1)	0 (0 to 0)	0.1 (0 to 0.2)	1 (0 to 1)	0.3 (0.2 to 0.6)
Bahrain	Both	85 (63 to 114)	13.8 (10.4 to 17.8)	40 (27 to 57)	6.3 (4.2 to 8.6)	0 (0 to 0)	0 (0 to 0)	9 (5 to 15)	1.4 (0.8 to 2.3)	4 (0 to 15)	0.7 (0.1 to 2.3)	2 (1 to 4)	0.3 (0.1 to 0.6)	30 (7 to 62)	5.2 (1.3 to 10.5)	16 (7 to 27)	4.1 (1.9 to 6.5)	11 (5 to 19)	0.9 (0.4 to 1.6)
	Male	75 (54 to 103)	22.9 (17.1 to 29.4)	30 (20 to 45)	8.6 (5.8 to 12.2)	0 (0 to 0)	0 (0 to 0)	7 (4 to 11)	1.9 (1.1 to 3.2)	3 (0 to 11)	0.9 (0.1 to 3.2)	2 (0 to 3)	0.4 (0.1 to 0.9)	23 (4 to 49)	7.3 (1.5 to 15.3)	16 (7 to 27)	8.2 (3.6 to 12.8)	10 (4 to 18)	1.3 (0.6 to 2.4)
	Female	9 (7 to 13)	3.8 (2.8 to 5.1)	10 (7 to 14)	3.7 (2.6 to 5)	0 (0 to 0)	0 (0 to 0)	3 (1 to 4)	0.9 (0.5 to 1.4)	1 (0 to 4)	0.4 (0 to 1.5)	1 (0 to 1)	0.2 (0.1 to 0.4)	7 (2 to 15)	2.9 (0.7 to 6)	0 (0 to 0)	0.1 (0.1 to 0.2)	1 (0 to 2)	0.2 (0.1 to 0.4)
Bangladesh	Both	5640 (3670 to 8930)	4.5 (3 to 7)	1550 (784 to 2750)	1.2 (0.6 to 2.1)	1790 (919 to 3200)	1.4 (0.7 to 2.5)	432 (195 to 842)	0.3 (0.2 to 0.7)	347 (37 to 1190)	0.3 (0 to 0.9)	600 (185 to 1220)	0.5 (0.1 to 1)	731 (139 to 1980)	0.6 (0.1 to 1.6)	269 (97 to 769)	0.2 (0.1 to 0.7)	576 (222 to 1220)	0.4 (0.2 to 0.9)
	Male	5440 (3520 to 8650)	8.2 (5.4 to 12.9)	1260 (644 to 2310)	1.9 (1 to 3.4)	1330 (649 to 2470)	2 (1 to 3.7)	285 (126 to 569)	0.4 (0.2 to 0.9)	270 (27 to 924)	0.4 (0 to 1.4)	467 (144 to 977)	0.7 (0.2 to 1.5)	574 (90 to 1650)	0.9 (0.1 to 2.5)	231 (71 to 725)	0.4 (0.1 to 1.2)	507 (191 to 1090)	0.7 (0.3 to 1.5)
	Female	208 (124 to 332)	0.4 (0.2 to 0.6)	285 (142 to 488)	0.5 (0.2 to 0.8)	460 (254 to 776)	0.8 (0.4 to 1.3)	147 (68 to 269)	0.2 (0.1 to 0.4)	77 (8 to 254)	0.1 (0 to 0.4)	133 (46 to 249)	0.2 (0.1 to 0.4)	157 (27 to 415)	0.3 (0 to 0.7)	38 (15 to 79)	0.1 (0 to 0.2)	70 (25 to 142)	0.1 (0 to 0.2)
Barbados	Both	19 (15 to 22)	3.7 (3 to 4.5)	7 (3 to 12)	1.4 (0.6 to 2.5)	0 (0 to 0)	0 (0 to 0)	1 (1 to 2)	0.2 (0.1 to 0.4)	1 (0 to 5)	0.3 (0 to 1)	2 (1 to 4)	0.5 (0.2 to 0.8)	6 (1 to 13)	1.2 (0.3 to 2.6)	3 (1 to 4)	0.5 (0.3 to 0.8)	3 (1 to 4)	0.5 (0.3 to 0.9)

	Male	16 (13 to 20)	7.3 (5.9 to 8.8)	5 (2 to 8)	2 (0.8 to 3.5)	0 (0 to 0)	0 (0 to 0)	1 (0 to 1)	0.3 (0.2 to 0.5)	1 (0 to 3)	0.4 (0 to 1.5)	2 (1 to 2)	0.7 (0.2 to 1.1)	4 (1 to 9)	1.7 (0.3 to 4)	2 (1 to 4)	1.1 (0.6 to 1.7)	2 (1 to 3)	0.8 (0.3 to 1.3)
	Female	2 (2 to 3)	0.9 (0.6 to 1.1)	3 (1 to 4)	0.9 (0.4 to 1.6)	0 (0 to 0)	0 (0 to 0)	0 (0 to 1)	0.1 (0.1 to 0.2)	0 (0 to 2)	0.2 (0 to 0.7)	1 (0 to 1)	0.3 (0.1 to 0.5)	2 (0 to 5)	0.8 (0.2 to 1.8)	0 (0 to 1)	0.1 (0.1 to 0.2)	1 (0 to 1)	0.3 (0.1 to 0.5)
Belarus	Both	2640 (2040 to 3410)	16.4 (12.7 to 21.2)	414 (258 to 616)	2.6 (1.6 to 3.9)	4 (1 to 11)	0 (0 to 0.1)	127 (69 to 203)	0.8 (0.4 to 1.3)	60 (7 to 173)	0.4 (0 to 1.1)	154 (40 to 254)	1 (0.2 to 1.6)	169 (32 to 415)	1 (0.2 to 2.6)	140 (64 to 248)	0.9 (0.4 to 1.5)	121 (18 to 253)	0.8 (0.1 to 1.6)
	Male	2490 (1920 to 3200)	40.5 (31.5 to 51.7)	353 (218 to 527)	5.7 (3.5 to 8.5)	3 (1 to 8)	0.1 (0 to 0.1)	89 (48 to 145)	1.4 (0.8 to 2.3)	51 (6 to 146)	0.8 (0.1 to 2.4)	131 (33 to 217)	2.1 (0.6 to 3.5)	145 (24 to 364)	2.4 (0.4 to 6)	115 (51 to 212)	2 (0.9 to 3.7)	104 (15 to 216)	1.5 (0.2 to 3.1)
	Female	150 (106 to 205)	1.6 (1.1 to 2.2)	62 (38 to 93)	0.6 (0.4 to 0.9)	1 (0 to 2)	0 (0 to 0)	37 (21 to 59)	0.4 (0.2 to 0.6)	9 (1 to 26)	0.1 (0 to 0.3)	24 (6 to 39)	0.2 (0.1 to 0.4)	24 (4 to 61)	0.2 (0 to 0.6)	25 (6 to 65)	0.2 (0.1 to 0.6)	18 (2 to 39)	0.2 (0 to 0.4)
Belgium	Both	5740 (5350 to 6130)	25.5 (23.9 to 27.2)	696 (466 to 980)	3.1 (2.1 to 4.3)	1 (0 to 3)	0 (0 to 0)	296 (177 to 448)	1.4 (0.9 to 2.1)	439 (61 to 1090)	2 (0.3 to 4.8)	284 (68 to 427)	1.3 (0.3 to 1.9)	700 (159 to 1540)	3 (0.7 to 6.6)	2520 (1820 to 3210)	10.3 (7.3 to 13.2)	257 (43 to 498)	1.2 (0.2 to 2.4)
	Male	4280 (3980 to 4570)	41.5 (38.7 to 44.3)	494 (330 to 693)	4.8 (3.2 to 6.7)	1 (0 to 2)	0 (0 to 0)	203 (119 to 314)	2.1 (1.2 to 3.2)	311 (43 to 770)	3 (0.4 to 7.5)	199 (46 to 302)	1.9 (0.5 to 2.9)	516 (84 to 1200)	4.9 (0.8 to 11.4)	2350 (1660 to 3000)	21.9 (15.4 to 28.1)	204 (33 to 396)	2 (0.3 to 4)
	Female	1460 (1320 to 1600)	12.6 (11.5 to 13.8)	202 (134 to 285)	1.7 (1.1 to 2.4)	0 (0 to 1)	0 (0 to 0)	92 (55 to 139)	0.9 (0.5 to 1.3)	128 (17 to 324)	1.1 (0.1 to 2.8)	85 (21 to 129)	0.7 (0.2 to 1.1)	184 (36 to 428)	1.4 (0.3 to 3.4)	178 (100 to 269)	1.3 (0.7 to 2)	53 (8 to 107)	0.5 (0.1 to 1)
Belize	Both	17 (15 to 20)	6.7 (5.6 to 7.8)	5 (2 to 9)	1.7 (0.6 to 3.3)	1 (1 to 3)	0.5 (0.2 to 1)	1 (1 to 2)	0.4 (0.2 to 0.7)	1 (0 to 4)	0.4 (0 to 1.4)	0 (0 to 0)	0.1 (0 to 0.1)	3 (1 to 7)	1.3 (0.3 to 2.9)	4 (2 to 6)	1.5 (0.9 to 2.3)	3 (1 to 5)	1 (0.5 to 1.6)
	Male	16 (13 to 18)	11.8 (9.9 to 13.9)	4 (1 to 7)	2.6 (0.9 to 4.8)	1 (0 to 2)	0.7 (0.3 to 1.3)	1 (0 to 1)	0.6 (0.3 to 0.9)	1 (0 to 3)	0.5 (0 to 2.1)	0 (0 to 0)	0.1 (0 to 0.2)	2 (0 to 5)	1.8 (0.3 to 4.2)	4 (2 to 5)	2.9 (1.7 to 4.3)	2 (1 to 4)	1.6 (0.7 to 2.7)
	Female	2 (1 to 2)	1.4 (1.1	1 (0 to 2)	0.9 (0.3	0 (0 to 1)	0.3 (0.2	0 (0 to 1)	0.3 (0.2	0 (0 to 1)	0.2 (0 to 0.8)	0 (0 to 0)	0 (0 to 0.1)	1 (0 to 2)	0.8 (0.2	0 (0 to 0)	0.1 (0.1	0 (0 to 1)	0.3 (0.1

			to 1.7)		to 1.6)		to 0.6)		to 0.4)						to 1.9)		to 0.2)		to 0.5)
Benin	Both	164 (121 to 219)	3.8 (2.8 to 5.1)	38 (16 to 72)	0.9 (0.4 to 1.6)	139 (88 to 209)	3.1 (2 to 4.7)	12 (6 to 20)	0.3 (0.1 to 0.4)	22 (2 to 81)	0.5 (0 to 1.8)	28 (10 to 47)	0.6 (0.2 to 1.1)	39 (8 to 89)	1 (0.2 to 2.2)	12 (5 to 24)	0.3 (0.1 to 0.6)	26 (11 to 47)	0.5 (0.2 to 0.9)
	Male	153 (112 to 204)	7.9 (5.8 to 10.4)	30 (12 to 56)	1.5 (0.6 to 2.8)	98 (60 to 148)	4.9 (3 to 7.3)	8 (4 to 14)	0.4 (0.2 to 0.6)	16 (1 to 60)	0.8 (0.1 to 3)	20 (7 to 34)	1 (0.3 to 1.7)	29 (5 to 69)	1.6 (0.3 to 3.7)	10 (4 to 22)	0.6 (0.2 to 1.2)	19 (8 to 35)	0.7 (0.3 to 1.4)
	Female	11 (7 to 17)	0.5 (0.3 to 0.7)	8 (3 to 16)	0.3 (0.1 to 0.6)	41 (27 to 63)	1.7 (1.1 to 2.6)	4 (2 to 7)	0.1 (0.1 to 0.2)	6 (1 to 22)	0.2 (0 to 0.9)	8 (3 to 13)	0.3 (0.1 to 0.5)	10 (2 to 25)	0.5 (0.1 to 1.1)	1 (1 to 3)	0.1 (0 to 0.1)	7 (3 to 13)	0.2 (0.1 to 0.4)
Bermuda	Both	16 (13 to 19)	12.1 (10 to 14.6)	1 (0 to 2)	0.9 (0.2 to 1.8)	0 (0 to 0)	0.1 (0 to 0.2)	1 (1 to 2)	0.9 (0.5 to 1.4)	1 (0 to 4)	0.7 (0.1 to 2.7)	1 (0 to 2)	1 (0.3 to 1.6)	3 (1 to 7)	2.3 (0.5 to 5.2)	5 (3 to 7)	3.5 (2.2 to 5.1)	2 (1 to 3)	1.5 (0.7 to 2.4)
	Male	13 (11 to 15)	21.9 (18.2 to 26.1)	1 (0 to 2)	1.4 (0.2 to 2.9)	0 (0 to 0)	0.1 (0 to 0.3)	1 (0 to 1)	1.4 (0.8 to 2.3)	1 (0 to 2)	1.2 (0.1 to 4.3)	1 (0 to 1)	1.6 (0.5 to 2.5)	2 (0 to 5)	3.9 (0.7 to 9)	5 (3 to 7)	8 (4.9 to 11.7)	1 (1 to 2)	2.4 (1.1 to 4.1)
	Female	3 (3 to 5)	4.5 (3.4 to 5.9)	0 (0 to 1)	0.5 (0.1 to 1)	0 (0 to 0)	0.1 (0 to 0.2)	0 (0 to 1)	0.5 (0.3 to 0.8)	0 (0 to 1)	0.4 (0 to 1.5)	0 (0 to 1)	0.6 (0.2 to 0.9)	1 (0 to 2)	1.1 (0.2 to 2.6)	0 (0 to 1)	0.4 (0.2 to 0.7)	0 (0 to 1)	0.6 (0.3 to 1)
Bhutan	Both	19 (12 to 26)	3.5 (2.3 to 5)	6 (3 to 9)	1.1 (0.6 to 1.8)	7 (4 to 11)	1.2 (0.7 to 2)	1 (1 to 2)	0.3 (0.1 to 0.4)	3 (0 to 10)	0.6 (0.1 to 1.8)	2 (1 to 4)	0.4 (0.1 to 0.8)	4 (1 to 9)	0.7 (0.2 to 1.7)	3 (1 to 5)	0.6 (0.3 to 1)	2 (1 to 4)	0.3 (0.1 to 0.6)
	Male	16 (10 to 24)	6.2 (3.9 to 9)	4 (2 to 8)	1.6 (0.8 to 2.9)	5 (2 to 8)	1.7 (0.8 to 2.9)	1 (0 to 2)	0.4 (0.2 to 0.7)	2 (0 to 7)	0.8 (0.1 to 2.7)	2 (1 to 3)	0.6 (0.2 to 1.1)	3 (0 to 7)	1 (0.2 to 2.7)	2 (1 to 5)	1 (0.4 to 2)	1 (1 to 3)	0.5 (0.2 to 0.9)
	Female	2 (1 to 3)	0.8 (0.5 to 1.3)	1 (1 to 2)	0.5 (0.2 to 0.9)	2 (1 to 4)	0.8 (0.4 to 1.3)	0 (0 to 1)	0.1 (0.1 to 0.3)	1 (0 to 3)	0.3 (0 to 1)	1 (0 to 1)	0.2 (0.1 to 0.5)	1 (0 to 2)	0.4 (0.1 to 0.9)	0 (0 to 1)	0.1 (0 to 0.2)	1 (0 to 1)	0.2 (0.1 to 0.4)
Bolivia	Both	408 (243 to 579)	4.9 (2.9 to 6.9)	168 (86 to 280)	2 (1 to 3.3)	91 (40 to 162)	1.1 (0.5 to 1.9)	17 (7 to 32)	0.2 (0.1 to 0.4)	54 (3 to 196)	0.6 (0 to 2.4)	47 (11 to 81)	0.6 (0.1 to 1)	111 (23 to 282)	1.4 (0.3 to 3.5)	131 (57 to 235)	1.7 (0.8 to 3.1)	103 (42 to 188)	1.1 (0.4 to 2)

	Male	359 (207 to 518)	9.2 (5.3 to 13.2)	107 (53 to 181)	2.7 (1.3 to 4.6)	49 (20 to 92)	1.3 (0.5 to 2.3)	10 (4 to 20)	0.3 (0.1 to 0.5)	33 (2 to 123)	0.8 (0.1 to 3.1)	28 (7 to 51)	0.7 (0.2 to 1.3)	71 (11 to 185)	1.9 (0.3 to 4.9)	109 (43 to 204)	3.1 (1.3 to 5.8)	74 (27 to 142)	1.6 (0.6 to 3.1)
	Female	49 (28 to 78)	1.1 (0.6 to 1.7)	61 (31 to 104)	1.4 (0.7 to 2.3)	42 (20 to 72)	1 (0.4 to 1.6)	7 (3 to 13)	0.1 (0.1 to 0.3)	21 (1 to 77)	0.5 (0 to 1.7)	19 (4 to 33)	0.4 (0.1 to 0.7)	40 (8 to 106)	0.9 (0.2 to 2.4)	22 (9 to 44)	0.5 (0.2 to 1.1)	29 (11 to 55)	0.6 (0.2 to 1.1)
Bosnia and Herzegovina	Both	1920 (1490 to 2410)	31.4 (24.4 to 39.4)	413 (270 to 574)	6.8 (4.5 to 9.5)	117 (43 to 241)	1.9 (0.7 to 4)	184 (107 to 287)	3 (1.8 to 4.7)	117 (10 to 387)	1.9 (0.2 to 6.4)	82 (20 to 132)	1.4 (0.3 to 2.2)	309 (64 to 710)	5 (1 to 11.4)	118 (55 to 209)	1.9 (0.9 to 3.3)	77 (12 to 159)	1.3 (0.2 to 2.6)
	Male	1540 (1180 to 1940)	56.6 (44 to 71.3)	319 (210 to 437)	11.8 (7.8 to 16.1)	81 (28 to 169)	3 (1 to 6.2)	133 (76 to 211)	4.9 (2.8 to 7.8)	89 (7 to 295)	3.3 (0.3 to 10.9)	62 (15 to 101)	2.3 (0.6 to 3.8)	243 (42 to 574)	8.9 (1.5 to 21)	115 (53 to 206)	4.2 (2 to 7.6)	67 (10 to 137)	2.3 (0.3 to 4.7)
	Female	377 (292 to 480)	11.3 (8.7 to 14.4)	93 (61 to 131)	2.8 (1.8 to 3.9)	36 (14 to 71)	1.1 (0.4 to 2.2)	51 (29 to 78)	1.6 (0.9 to 2.4)	28 (2 to 95)	0.8 (0.1 to 2.9)	20 (5 to 33)	0.6 (0.1 to 1)	66 (14 to 157)	1.9 (0.4 to 4.6)	4 (1 to 8)	0.1 (0 to 0.2)	11 (1 to 23)	0.3 (0 to 0.7)
Botswana	Both	147 (104 to 192)	11.6 (8.4 to 15)	41 (23 to 65)	3.1 (1.8 to 4.8)	25 (11 to 45)	1.9 (0.9 to 3.5)	14 (7 to 25)	1.1 (0.5 to 1.9)	12 (1 to 43)	0.9 (0.1 to 3.2)	19 (6 to 32)	1.4 (0.5 to 2.4)	27 (6 to 63)	2.3 (0.5 to 5.3)	51 (28 to 80)	4.6 (2.5 to 6.9)	10 (4 to 19)	0.6 (0.2 to 1.1)
	Male	125 (89 to 160)	23.9 (17.1 to 30.1)	30 (17 to 46)	5.3 (3 to 8)	16 (7 to 30)	2.8 (1.2 to 5.3)	9 (4 to 15)	1.6 (0.8 to 2.7)	8 (1 to 30)	1.5 (0.1 to 5.2)	13 (4 to 22)	2.3 (0.8 to 3.8)	18 (3 to 44)	3.8 (0.7 to 9.2)	47 (25 to 73)	10.8 (5.9 to 16.2)	8 (3 to 15)	1.1 (0.4 to 2)
	Female	22 (14 to 33)	3.3 (2.1 to 4.9)	11 (6 to 20)	1.6 (0.8 to 2.7)	9 (4 to 17)	1.3 (0.6 to 2.3)	5 (3 to 10)	0.7 (0.4 to 1.3)	4 (0 to 13)	0.5 (0 to 1.8)	6 (2 to 10)	0.8 (0.2 to 1.4)	9 (2 to 22)	1.3 (0.2 to 3.2)	4 (2 to 9)	0.7 (0.3 to 1.5)	2 (1 to 4)	0.2 (0.1 to 0.4)
Brazil	Both	22000 (20400 to 23500)	9.4 (8.7 to 10)	2840 (1830 to 4010)	1.2 (0.8 to 1.7)	773 (322 to 1460)	0.3 (0.1 to 0.6)	1770 (1040 to 2680)	0.7 (0.4 to 1.1)	1540 (268 to 3350)	0.7 (0.1 to 1.4)	890 (241 to 1360)	0.4 (0.1 to 0.6)	3330 (784 to 7210)	1.4 (0.3 to 3.1)	2990 (2020 to 4000)	1.3 (0.9 to 1.8)	2110 (1090 to 3250)	0.8 (0.4 to 1.3)
	Male	14200 (13200 to 15200)	13.8 (12.8 to 14.8)	1680 (1060 to 2390)	1.6 (1 to 2.3)	394 (151 to 764)	0.4 (0.1 to 0.7)	1010 (574 to 1560)	1 (0.5 to 1.5)	896 (155 to 1930)	0.9 (0.1 to 1.9)	501 (135 to 787)	0.5 (0.1 to 0.8)	2030 (336 to 4660)	2 (0.3 to 4.6)	2130 (1290 to 3060)	2.3 (1.4 to 3.3)	1490 (712 to 2360)	1.3 (0.6 to 2)
	Female	7800 (7050	6 (5.4 to 6.5)	1160 (737	0.9 (0.6	379 (166	0.3 (0.1	757 (445	0.6 (0.3	640 (111	0.5 (0.1	389 (101	0.3 (0.1	1300 (247	1 (0.2 to 2.3)	862 (521	0.7 (0.4 to 1)	625 (296	0.5 (0.2

		to 8520)		to 1660)	to 1.3)	to 694)	to 0.5)	to 1150)	to 0.9)	to 1390)	to 1.1)	to 606)	to 0.5)	to 3000)		to 1260)		to 1010)	to 0.7)
Brunei	Both	59 (50 to 68)	25.9 (22.4 to 29.9)	4 (1 to 8)	1.8 (0.5 to 3.4)	0 (0 to 0)	0 (0 to 0.1)	4 (2 to 6)	1.5 (0.8 to 2.3)	1 (0 to 4)	0.4 (0 to 1.7)	5 (2 to 8)	2.3 (0.8 to 3.5)	19 (5 to 38)	8.8 (2.4 to 17.4)	8 (5 to 12)	4.8 (3.2 to 7)	4 (1 to 8)	1.3 (0.2 to 2.5)
	Male	41 (35 to 49)	45.1 (37.9 to 52.7)	2 (1 to 4)	2.5 (0.7 to 4.8)	0 (0 to 0)	0 (0 to 0.1)	2 (1 to 3)	1.6 (0.9 to 2.6)	1 (0 to 2)	0.6 (0 to 2.2)	3 (1 to 5)	3.1 (1 to 4.9)	12 (2 to 25)	13.6 (2.8 to 27.9)	6 (3 to 10)	11.6 (6.8 to 17.9)	3 (0 to 5)	1.8 (0.3 to 3.6)
	Female	17 (14 to 21)	14.3 (11.2 to 17.6)	2 (1 to 4)	1.5 (0.4 to 2.8)	0 (0 to 0)	0 (0 to 0.1)	2 (1 to 3)	1.4 (0.8 to 2.2)	0 (0 to 2)	0.4 (0 to 1.3)	2 (1 to 4)	1.9 (0.6 to 2.9)	7 (2 to 15)	6 (1.3 to 12.7)	2 (1 to 3)	1.9 (1 to 3)	1 (0 to 3)	0.8 (0.1 to 1.7)
Bulgaria	Both	3580 (2840 to 4430)	26.4 (20.8 to 32.9)	613 (423 to 858)	4.5 (3.1 to 6.4)	109 (31 to 258)	0.8 (0.2 to 1.9)	272 (152 to 432)	2.1 (1.1 to 3.3)	183 (26 to 434)	1.4 (0.2 to 3.2)	211 (63 to 343)	1.6 (0.5 to 2.6)	410 (79 to 981)	2.8 (0.5 to 6.8)	90 (42 to 156)	0.6 (0.3 to 1.1)	207 (33 to 420)	1.6 (0.3 to 3.3)
	Male	3020 (2390 to 3750)	49.2 (38.9 to 61.2)	486 (333 to 681)	8 (5.5 to 11.2)	78 (22 to 186)	1.3 (0.4 to 3.1)	199 (109 to 318)	3.3 (1.8 to 5.4)	144 (20 to 343)	2.4 (0.3 to 5.6)	165 (49 to 269)	2.7 (0.8 to 4.5)	332 (54 to 819)	5.3 (0.8 to 13)	79 (34 to 143)	1.2 (0.5 to 2.2)	169 (27 to 343)	2.8 (0.4 to 5.7)
	Female	561 (436 to 713)	7.7 (6 to 9.9)	127 (85 to 178)	1.7 (1.1 to 2.4)	31 (10 to 72)	0.4 (0.1 to 0.9)	73 (41 to 116)	1 (0.6 to 1.6)	39 (6 to 93)	0.5 (0.1 to 1.2)	46 (13 to 75)	0.6 (0.2 to 1)	78 (15 to 188)	0.9 (0.2 to 2.3)	10 (5 to 18)	0.1 (0.1 to 0.2)	37 (5 to 80)	0.5 (0.1 to 1.2)
Burkina Faso	Both	222 (157 to 322)	2.6 (1.8 to 3.8)	48 (15 to 109)	0.6 (0.2 to 1.3)	290 (178 to 472)	3.5 (2.1 to 5.6)	22 (10 to 38)	0.3 (0.1 to 0.4)	39 (3 to 140)	0.5 (0 to 1.7)	60 (20 to 99)	0.7 (0.2 to 1.2)	63 (13 to 152)	0.8 (0.2 to 2)	16 (7 to 38)	0.2 (0.1 to 0.5)	33 (14 to 61)	0.3 (0.1 to 0.6)
	Male	216 (152 to 316)	5.6 (4 to 8.2)	39 (12 to 89)	1 (0.3 to 2.4)	214 (129 to 355)	5.7 (3.5 to 9.4)	15 (7 to 28)	0.4 (0.2 to 0.7)	29 (2 to 106)	0.8 (0.1 to 2.9)	45 (15 to 77)	1.2 (0.4 to 2.1)	48 (8 to 121)	1.4 (0.2 to 3.6)	14 (5 to 36)	0.4 (0.1 to 1.1)	26 (10 to 49)	0.5 (0.2 to 1)
	Female	6 (3 to 10)	0.1 (0.1 to 0.2)	9 (3 to 20)	0.2 (0.1 to 0.4)	75 (45 to 130)	1.6 (1 to 2.8)	7 (3 to 12)	0.1 (0.1 to 0.2)	9 (1 to 33)	0.2 (0 to 0.7)	15 (5 to 25)	0.3 (0.1 to 0.5)	15 (3 to 37)	0.4 (0.1 to 0.9)	2 (1 to 5)	0.1 (0 to 0.1)	7 (3 to 13)	0.1 (0 to 0.2)
Burundi	Both	109 (75 to 153)	2.7 (1.9 to 3.8)	12 (4 to 29)	0.3 (0.1 to 0.7)	107 (63 to 171)	2.5 (1.5 to 4)	4 (2 to 8)	0.1 (0 to 0.2)	13 (1 to 54)	0.3 (0 to 1.3)	7 (1 to 12)	0.2 (0 to 0.3)	18 (3 to 45)	0.5 (0.1 to 1.2)	12 (3 to 55)	0.4 (0.1 to 1.6)	22 (9 to 42)	0.4 (0.2 to 0.8)

	Male	103 (71 to 145)	5 (3.5 to 6.9)	10 (3 to 24)	0.5 (0.1 to 1.1)	82 (48 to 132)	3.8 (2.3 to 6.1)	3 (1 to 6)	0.1 (0.1 to 0.2)	10 (1 to 41)	0.5 (0 to 1.9)	5 (1 to 9)	0.2 (0.1 to 0.4)	15 (2 to 38)	0.8 (0.1 to 2)	10 (2 to 53)	0.6 (0.1 to 3)	17 (7 to 32)	0.6 (0.2 to 1.1)
	Female	6 (3 to 10)	0.3 (0.2 to 0.4)	2 (1 to 5)	0.1 (0 to 0.2)	25 (14 to 43)	1.2 (0.7 to 2.1)	1 (0 to 2)	0 (0 to 0.1)	3 (0 to 12)	0.1 (0 to 0.6)	2 (0 to 3)	0.1 (0 to 0.1)	3 (0 to 8)	0.2 (0 to 0.5)	2 (0 to 5)	0.1 (0 to 0.3)	5 (2 to 11)	0.2 (0.1 to 0.4)
Cabo Verde	Both	22 (15 to 28)	5.5 (3.9 to 7.1)	16 (10 to 24)	4 (2.5 to 5.8)	5 (2 to 8)	1.2 (0.6 to 2)	2 (1 to 3)	0.5 (0.2 to 0.8)	4 (0 to 14)	0.9 (0.1 to 3.5)	4 (1 to 6)	1 (0.3 to 1.5)	7 (2 to 17)	1.9 (0.5 to 4.4)	1 (1 to 2)	0.3 (0.1 to 0.5)	2 (1 to 3)	0.4 (0.2 to 0.7)
	Male	19 (13 to 25)	11.6 (7.8 to 15.2)	11 (6 to 16)	6.5 (3.7 to 9.5)	3 (1 to 5)	1.6 (0.7 to 3)	1 (1 to 2)	0.7 (0.3 to 1.2)	2 (0 to 9)	1.4 (0.1 to 5.1)	2 (1 to 4)	1.5 (0.5 to 2.4)	4 (1 to 12)	3 (0.5 to 7.7)	1 (0 to 2)	0.7 (0.3 to 1.3)	1 (1 to 2)	0.6 (0.3 to 1.2)
	Female	3 (2 to 4)	1.2 (0.9 to 1.7)	6 (4 to 8)	2.4 (1.5 to 3.5)	2 (1 to 4)	0.9 (0.4 to 1.5)	1 (0 to 1)	0.3 (0.2 to 0.5)	1 (0 to 5)	0.6 (0 to 2.1)	1 (0 to 2)	0.6 (0.2 to 1)	3 (1 to 7)	1.2 (0.2 to 2.9)	0 (0 to 0)	0.1 (0 to 0.1)	1 (0 to 1)	0.2 (0.1 to 0.4)
Cambodia	Both	1930 (1490 to 2450)	17.5 (13.7 to 22.4)	200 (89 to 356)	1.8 (0.8 to 3.2)	711 (453 to 990)	6.3 (4 to 8.7)	155 (89 to 246)	1.3 (0.8 to 2.1)	58 (5 to 227)	0.5 (0 to 2)	176 (63 to 291)	1.6 (0.6 to 2.5)	284 (61 to 647)	2.7 (0.6 to 6.1)	144 (77 to 233)	1.5 (0.8 to 2.4)	219 (96 to 389)	1.6 (0.7 to 2.9)
	Male	1740 (1340 to 2250)	39.7 (30.6 to 52.1)	157 (68 to 278)	3.5 (1.5 to 6.2)	499 (305 to 716)	11.1 (6.7 to 16)	74 (41 to 123)	1.6 (0.9 to 2.7)	42 (3 to 163)	0.9 (0.1 to 3.5)	128 (44 to 211)	2.8 (1 to 4.7)	213 (37 to 511)	5.2 (0.9 to 12.3)	136 (70 to 223)	3.6 (1.9 to 5.9)	158 (66 to 286)	2.8 (1.2 to 5.1)
	Female	185 (137 to 239)	3 (2.3 to 3.8)	43 (19 to 79)	0.6 (0.3 to 1.2)	212 (137 to 297)	3.2 (2.1 to 4.4)	80 (45 to 127)	1.2 (0.7 to 1.8)	16 (1 to 61)	0.2 (0 to 0.9)	49 (18 to 80)	0.7 (0.3 to 1.2)	72 (14 to 169)	1.1 (0.2 to 2.6)	8 (3 to 15)	0.1 (0.1 to 0.2)	61 (24 to 113)	0.8 (0.3 to 1.5)
Cameroon	Both	531 (370 to 739)	4.9 (3.4 to 6.7)	261 (138 to 411)	2.4 (1.3 to 3.8)	269 (148 to 426)	2.5 (1.4 to 3.9)	31 (15 to 52)	0.3 (0.1 to 0.5)	78 (6 to 278)	0.7 (0.1 to 2.5)	51 (12 to 90)	0.5 (0.1 to 0.8)	114 (23 to 277)	1.2 (0.2 to 2.8)	56 (26 to 105)	0.6 (0.3 to 1.1)	74 (31 to 138)	0.5 (0.2 to 1)
	Male	512 (357 to 714)	9.8 (6.9 to 13.3)	196 (103 to 311)	3.8 (2.1 to 5.9)	179 (92 to 293)	3.5 (1.8 to 5.7)	18 (9 to 31)	0.3 (0.2 to 0.6)	55 (4 to 202)	1.1 (0.1 to 3.9)	36 (8 to 62)	0.7 (0.2 to 1.2)	83 (13 to 210)	1.8 (0.3 to 4.7)	51 (22 to 97)	1.1 (0.5 to 2.1)	56 (22 to 105)	0.8 (0.3 to 1.5)
	Female	19 (11 to 31)	0.4 (0.2	65 (32 to 106)	1.1 (0.6	90 (50 to 146)	1.6 (0.9	13 (6 to 22)	0.2 (0.1	23 (2 to 78)	0.4 (0 to 1.4)	16 (4 to 27)	0.3 (0.1	31 (6 to 81)	0.6 (0.1	5 (2 to 12)	0.1 (0 to 0.3)	18 (7 to 36)	0.3 (0.1

			to 0.6)		to 1.8)		to 2.5)		to 0.4)				to 0.5)		to 1.6)				to 0.5)
Canada	Both	16300 (14900 to 17600)	23 (21.2 to 24.8)	830 (353 to 1450)	1.2 (0.5 to 2)	2 (0 to 6)	0 (0 to 0)	781 (466 to 1190)	1.1 (0.7 to 1.7)	678 (76 to 1930)	1 (0.1 to 2.7)	862 (211 to 1300)	1.2 (0.3 to 1.8)	1900 (436 to 4210)	2.6 (0.6 to 5.8)	5540 (3950 to 7110)	7.5 (5.3 to 9.7)	750 (126 to 1470)	1.1 (0.2 to 2.2)
	Male	9320 (8540 to 10100)	28.5 (26.1 to 30.9)	449 (194 to 771)	1.4 (0.6 to 2.4)	1 (0 to 3)	0 (0 to 0)	461 (267 to 700)	1.4 (0.8 to 2.2)	367 (41 to 1050)	1.1 (0.1 to 3.2)	457 (105 to 697)	1.4 (0.3 to 2.2)	1130 (189 to 2640)	3.4 (0.6 to 8)	4810 (3370 to 6300)	14.6 (10.2 to 19)	452 (70 to 898)	1.4 (0.2 to 2.7)
	Female	6970 (6170 to 7750)	18.5 (16.4 to 20.4)	381 (161 to 668)	1 (0.4 to 1.8)	1 (0 to 3)	0 (0 to 0)	321 (186 to 490)	0.9 (0.5 to 1.4)	311 (35 to 892)	0.8 (0.1 to 2.4)	405 (105 to 621)	1.1 (0.3 to 1.6)	765 (145 to 1830)	2 (0.4 to 4.7)	726 (409 to 1110)	1.8 (1 to 2.7)	298 (44 to 607)	0.8 (0.1 to 1.7)
Central Africa Republic	Both	97 (45 to 186)	4.5 (2.3 to 8.3)	15 (3 to 39)	0.7 (0.1 to 1.7)	92 (41 to 185)	4.2 (2 to 8.1)	6 (2 to 13)	0.2 (0.1 to 0.5)	10 (1 to 41)	0.5 (0 to 1.8)	15 (4 to 34)	0.7 (0.2 to 1.5)	24 (4 to 73)	1.2 (0.2 to 3.6)	12 (3 to 39)	0.7 (0.2 to 2.2)	17 (5 to 40)	0.6 (0.2 to 1.4)
	Male	94 (42 to 181)	10.3 (5.3 to 18.6)	13 (3 to 35)	1.3 (0.3 to 3.5)	75 (29 to 160)	7.8 (3.3 to 15.9)	5 (2 to 11)	0.4 (0.1 to 1)	8 (1 to 35)	0.9 (0.1 to 3.5)	13 (3 to 30)	1.3 (0.3 to 3)	20 (3 to 67)	2.4 (0.3 to 7.6)	10 (2 to 37)	1.5 (0.3 to 5.3)	15 (4 to 36)	1.1 (0.3 to 2.7)
	Female	3 (2 to 5)	0.3 (0.2 to 0.5)	2 (1 to 4)	0.2 (0 to 0.4)	17 (10 to 29)	1.5 (0.9 to 2.6)	1 (1 to 2)	0.1 (0 to 0.2)	2 (0 to 6)	0.2 (0 to 0.6)	3 (1 to 5)	0.2 (0.1 to 0.4)	3 (1 to 8)	0.3 (0.1 to 0.8)	2 (0 to 4)	0.2 (0 to 0.4)	2 (1 to 4)	0.1 (0.1 to 0.3)
Chad	Both	240 (161 to 359)	4.8 (3.3 to 7.2)	35 (10 to 82)	0.7 (0.2 to 1.6)	211 (122 to 350)	4.1 (2.4 to 6.9)	14 (7 to 25)	0.3 (0.1 to 0.5)	27 (2 to 97)	0.5 (0 to 1.9)	42 (14 to 75)	0.8 (0.3 to 1.5)	46 (9 to 114)	1 (0.2 to 2.4)	12 (4 to 31)	0.2 (0.1 to 0.7)	26 (10 to 49)	0.4 (0.2 to 0.8)
	Male	230 (152 to 351)	8.5 (5.7 to 13)	30 (9 to 72)	1.1 (0.3 to 2.6)	169 (96 to 299)	6.1 (3.4 to 10.8)	11 (5 to 20)	0.4 (0.2 to 0.7)	22 (1 to 77)	0.8 (0.1 to 2.8)	34 (11 to 64)	1.2 (0.4 to 2.3)	38 (6 to 102)	1.5 (0.2 to 4)	10 (4 to 30)	0.4 (0.1 to 1.1)	22 (8 to 43)	0.6 (0.2 to 1.2)
	Female	10 (6 to 16)	0.5 (0.3 to 0.8)	5 (1 to 11)	0.2 (0.1 to 0.5)	42 (24 to 72)	1.7 (1 to 3)	4 (2 to 7)	0.1 (0.1 to 0.3)	5 (0 to 19)	0.2 (0 to 0.8)	8 (3 to 14)	0.3 (0.1 to 0.6)	8 (2 to 22)	0.4 (0.1 to 1)	1 (0 to 3)	0.1 (0 to 0.1)	4 (1 to 8)	0.1 (0 to 0.3)
Chile	Both	2160 (1970 to 2360)	8.9 (8.1 to 9.7)	623 (446 to 828)	2.6 (1.8 to 3.4)	35 (11 to 82)	0.1 (0 to 0.3)	213 (118 to 332)	0.9 (0.5 to 1.4)	129 (9 to 466)	0.5 (0 to 1.9)	158 (39 to 236)	0.7 (0.2 to 1)	459 (111 to 971)	1.9 (0.5 to 4)	343 (208 to 498)	1.4 (0.9 to 2.1)	226 (115 to 356)	0.9 (0.5 to 1.4)

	Male	1360 (1230 to 1490)	12.5 (11.3 to 13.8)	364 (262 to 484)	3.4 (2.4 to 4.5)	17 (5 to 42)	0.2 (0 to 0.4)	127 (69 to 200)	1.2 (0.6 to 1.9)	75 (5 to 273)	0.7 (0 to 2.6)	91 (23 to 138)	0.8 (0.2 to 1.3)	268 (48 to 607)	2.5 (0.4 to 5.7)	293 (167 to 433)	2.8 (1.6 to 4.2)	162 (76 to 263)	1.4 (0.7 to 2.3)
	Female	797 (698 to 897)	6 (5.2 to 6.7)	259 (184 to 344)	1.9 (1.4 to 2.5)	18 (5 to 43)	0.1 (0 to 0.3)	86 (47 to 136)	0.6 (0.3 to 1)	54 (4 to 204)	0.4 (0 to 1.5)	67 (16 to 103)	0.5 (0.1 to 0.8)	190 (38 to 425)	1.4 (0.3 to 3.1)	50 (27 to 82)	0.4 (0.2 to 0.6)	64 (30 to 105)	0.5 (0.2 to 0.8)
China	Both	492000 (399000 to 593000)	24.9 (20.4 to 29.9)	171000 (122000 to 228000)	8.8 (6.3 to 11.6)	37300 (17400 to 66200)	1.9 (0.9 to 3.3)	59200 (35500 to 88200)	3 (1.8 to 4.5)	28600 (5190 to 61100)	1.5 (0.3 to 3.1)	27200 (7160 to 43100)	1.4 (0.4 to 2.2)	53000 (11800 to 121000)	2.7 (0.6 to 6.2)	23200 (14400 to 34700)	1.3 (0.8 to 2)	41600 (19800 to 68000)	1.9 (0.9 to 3.1)
	Male	430000 (339000 to 534000)	47.7 (37.9 to 58.5)	120000 (81600 to 165000)	13.4 (9.2 to 18.2)	22900 (10000 to 43600)	2.5 (1.1 to 4.7)	32100 (18300 to 50200)	3.6 (2.1 to 5.6)	19500 (3620 to 41900)	2.2 (0.4 to 4.6)	19000 (5000 to 31500)	2.1 (0.6 to 3.5)	37200 (6190 to 91100)	4.2 (0.7 to 10.2)	18100 (9930 to 30000)	2.5 (1.4 to 4.1)	28900 (13000 to 48800)	2.6 (1.2 to 4.4)
	Female	61300 (48700 to 76500)	6.1 (4.8 to 7.5)	51100 (35900 to 69000)	5 (3.5 to 6.7)	14300 (6800 to 25000)	1.4 (0.7 to 2.4)	27100 (16000 to 41200)	2.6 (1.6 to 4)	9020 (1540 to 18900)	0.9 (0.1 to 1.8)	8210 (2220 to 12900)	0.8 (0.2 to 1.3)	15800 (2940 to 38900)	1.5 (0.3 to 3.8)	5070 (2590 to 7880)	0.5 (0.3 to 0.8)	12600 (5420 to 21300)	1.1 (0.5 to 1.9)
Colombia	Both	2470 (1880 to 3210)	4.7 (3.6 to 6.1)	898 (567 to 1320)	1.7 (1.1 to 2.5)	166 (67 to 332)	0.3 (0.1 to 0.6)	198 (98 to 339)	0.4 (0.2 to 0.6)	344 (28 to 1270)	0.6 (0.1 to 2.4)	185 (40 to 301)	0.3 (0.1 to 0.6)	725 (172 to 1650)	1.4 (0.3 to 3.1)	262 (130 to 458)	0.5 (0.2 to 0.9)	389 (185 to 659)	0.7 (0.3 to 1.2)
	Male	1620 (1210 to 2100)	6.8 (5.1 to 8.9)	495 (313 to 735)	2.1 (1.3 to 3.1)	74 (28 to 156)	0.3 (0.1 to 0.7)	117 (58 to 200)	0.5 (0.2 to 0.8)	186 (15 to 694)	0.8 (0.1 to 2.9)	97 (20 to 161)	0.4 (0.1 to 0.7)	421 (72 to 1010)	1.8 (0.3 to 4.3)	209 (96 to 382)	0.9 (0.4 to 1.6)	256 (114 to 451)	1 (0.5 to 1.8)
	Female	850 (641 to 1100)	3 (2.2 to 3.8)	403 (255 to 599)	1.4 (0.9 to 2)	92 (39 to 176)	0.3 (0.1 to 0.6)	82 (41 to 140)	0.3 (0.1 to 0.5)	158 (13 to 590)	0.5 (0 to 2)	88 (19 to 145)	0.3 (0.1 to 0.5)	304 (62 to 722)	1 (0.2 to 2.5)	54 (25 to 97)	0.2 (0.1 to 0.3)	133 (59 to 234)	0.5 (0.2 to 0.8)
Comoros	Both	14 (10 to 20)	3.1 (2.3 to 4.3)	2 (1 to 3)	0.4 (0.2 to 0.7)	9 (6 to 14)	2 (1.3 to 2.9)	1 (1 to 2)	0.3 (0.1 to 0.5)	1 (0 to 6)	0.3 (0 to 1.2)	1 (0 to 2)	0.3 (0.1 to 0.5)	2 (0 to 5)	0.5 (0.1 to 1.1)	2 (1 to 7)	0.4 (0.1 to 1.5)	2 (1 to 4)	0.4 (0.2 to 0.7)
	Male	13 (9 to 19)	6.6 (4.8	1 (1 to 3)	0.6 (0.3	6 (4 to 9)	2.9 (1.7	1 (0 to 1)	0.4 (0.2	1 (0 to 4)	0.4 (0 to 1.7)	1 (0 to 2)	0.4 (0.1	1 (0 to 4)	0.8 (0.1	1 (0 to 6)	0.7 (0.2	1 (1 to 3)	0.6 (0.2

			to 9.2)		to 1.2)		to 4.5)		to 0.7)				to 0.8)		to 1.9)		to 3.2)		to 1.1)
	Female	1 (1 to 2)	0.4 (0.3 to 0.7)	1 (0 to 1)	0.2 (0.1 to 0.4)	4 (2 to 5)	1.4 (0.9 to 1.9)	1 (0 to 1)	0.2 (0.1 to 0.3)	0 (0 to 2)	0.2 (0 to 0.8)	0 (0 to 1)	0.2 (0 to 0.3)	1 (0 to 1)	0.2 (0 to 0.6)	1 (0 to 1)	0.2 (0.1 to 0.4)	1 (0 to 1)	0.2 (0.1 to 0.4)
Congo	Both	143 (104 to 203)	6.1 (4.6 to 8.4)	66 (32 to 114)	2.7 (1.4 to 4.5)	54 (27 to 96)	2.2 (1.1 to 3.8)	10 (5 to 18)	0.4 (0.2 to 0.7)	15 (1 to 59)	0.6 (0 to 2.4)	20 (6 to 37)	0.8 (0.3 to 1.5)	36 (7 to 84)	1.6 (0.3 to 3.6)	20 (7 to 61)	1 (0.4 to 3.1)	17 (7 to 33)	0.5 (0.2 to 1)
	Male	135 (99 to 193)	12.4 (9.5 to 17.2)	48 (24 to 81)	4.1 (2.1 to 6.9)	34 (16 to 62)	2.9 (1.4 to 5.3)	6 (3 to 11)	0.5 (0.2 to 0.9)	10 (1 to 40)	0.9 (0.1 to 3.3)	14 (4 to 25)	1.2 (0.4 to 2.1)	26 (4 to 65)	2.6 (0.4 to 6.1)	15 (5 to 56)	1.7 (0.5 to 6.3)	12 (5 to 24)	0.8 (0.3 to 1.5)
	Female	7 (4 to 12)	0.6 (0.3 to 0.9)	18 (8 to 36)	1.4 (0.6 to 2.7)	20 (9 to 35)	1.5 (0.7 to 2.6)	4 (2 to 7)	0.3 (0.1 to 0.5)	5 (0 to 19)	0.4 (0 to 1.5)	6 (2 to 12)	0.5 (0.1 to 0.9)	9 (2 to 24)	0.8 (0.1 to 2)	5 (1 to 10)	0.5 (0.1 to 0.9)	5 (2 to 10)	0.3 (0.1 to 0.6)
Cook Islands	Both	4 (4 to 5)	17.5 (14.6 to 20.7)	0 (0 to 1)	0.9 (0.2 to 2.3)	0 (0 to 0)	0.2 (0 to 0.6)	0 (0 to 1)	1.8 (1 to 2.9)	0 (0 to 1)	1 (0.1 to 3.8)	0 (0 to 0)	1 (0.3 to 1.6)	1 (0 to 3)	4.8 (1.1 to 10.4)	0 (0 to 1)	1.5 (0.7 to 3)	0 (0 to 1)	1.5 (0.7 to 2.5)
	Male	4 (3 to 5)	31.8 (26.5 to 37.6)	0 (0 to 0)	1.6 (0.3 to 3.9)	0 (0 to 0)	0.3 (0 to 0.9)	0 (0 to 1)	2.8 (1.5 to 4.6)	0 (0 to 1)	1.7 (0.1 to 6.5)	0 (0 to 0)	1.6 (0.4 to 2.7)	1 (0 to 2)	8 (1.5 to 17.7)	0 (0 to 1)	3.2 (1.5 to 6.4)	0 (0 to 1)	2.5 (1.1 to 4.3)
	Female	1 (0 to 1)	4.2 (3 to 5.4)	0 (0 to 0)	0.4 (0.1 to 0.9)	0 (0 to 0)	0.1 (0 to 0.3)	0 (0 to 0)	0.8 (0.4 to 1.4)	0 (0 to 0)	0.4 (0 to 1.3)	0 (0 to 0)	0.4 (0.1 to 0.6)	0 (0 to 1)	1.8 (0.4 to 4.2)	0 (0 to 0)	0.1 (0 to 0.2)	0 (0 to 0)	0.4 (0.2 to 0.8)
Costa Rica	Both	240 (188 to 307)	4.8 (3.7 to 6.1)	60 (38 to 88)	1.2 (0.7 to 1.7)	6 (2 to 14)	0.1 (0 to 0.3)	19 (10 to 32)	0.4 (0.2 to 0.6)	23 (2 to 82)	0.4 (0 to 1.6)	13 (3 to 21)	0.3 (0.1 to 0.4)	61 (13 to 139)	1.2 (0.3 to 2.8)	16 (8 to 27)	0.3 (0.2 to 0.5)	27 (12 to 45)	0.5 (0.2 to 0.8)
	Male	190 (146 to 244)	8.3 (6.4 to 10.6)	39 (24 to 58)	1.7 (1.1 to 2.5)	4 (1 to 8)	0.2 (0 to 0.4)	12 (7 to 21)	0.5 (0.3 to 0.9)	15 (1 to 53)	0.6 (0 to 2.3)	8 (2 to 14)	0.3 (0.1 to 0.6)	43 (7 to 102)	1.9 (0.3 to 4.5)	14 (7 to 24)	0.6 (0.3 to 1.1)	21 (9 to 37)	0.8 (0.4 to 1.5)
	Female	50 (37 to 65)	1.8 (1.4 to 2.4)	21 (13 to 30)	0.8 (0.5 to 1.1)	3 (1 to 6)	0.1 (0 to 0.2)	7 (4 to 11)	0.2 (0.1 to 0.4)	8 (1 to 29)	0.3 (0 to 1)	5 (1 to 8)	0.2 (0 to 0.3)	18 (4 to 45)	0.7 (0.1 to 1.6)	2 (1 to 4)	0.1 (0 to 0.2)	5 (2 to 10)	0.2 (0.1 to 0.3)

Croatia	Both	2200 (1740 to 2770)	25.5 (20 to 32.3)	374 (246 to 532)	4.3 (2.9 to 6.2)	19 (5 to 50)	0.2 (0.1 to 0.6)	229 (131 to 364)	2.7 (1.6 to 4.3)	154 (21 to 388)	1.8 (0.2 to 4.5)	93 (22 to 149)	1.1 (0.3 to 1.7)	319 (64 to 733)	3.5 (0.7 to 8.2)	695 (405 to 1040)	7.5 (4.3 to 11.4)	95 (15 to 193)	1.2 (0.2 to 2.4)
	Male	1730 (1360 to 2180)	45.8 (36.2 to 57.7)	280 (184 to 401)	7.4 (4.9 to 10.6)	12 (3 to 34)	0.3 (0.1 to 0.9)	168 (95 to 267)	4.5 (2.6 to 7.1)	115 (16 to 283)	3.1 (0.4 to 7.5)	68 (16 to 111)	1.8 (0.4 to 3)	249 (41 to 593)	6.5 (1.1 to 15.4)	663 (385 to 1010)	16.8 (9.8 to 25.5)	77 (12 to 156)	2 (0.3 to 4.1)
	Female	469 (366 to 603)	9.8 (7.6 to 12.7)	94 (63 to 136)	2 (1.3 to 2.8)	6 (2 to 17)	0.1 (0 to 0.3)	61 (34 to 97)	1.3 (0.7 to 2.1)	39 (6 to 98)	0.8 (0.1 to 2)	25 (6 to 40)	0.5 (0.1 to 0.8)	70 (13 to 170)	1.3 (0.3 to 3.3)	32 (15 to 56)	0.6 (0.3 to 1)	18 (2 to 39)	0.4 (0.1 to 0.9)
Cuba	Both	4560 (3720 to 5540)	23.8 (19.4 to 28.8)	801 (383 to 1390)	4.2 (2 to 7.3)	26 (8 to 62)	0.1 (0 to 0.3)	307 (174 to 487)	1.6 (0.9 to 2.5)	83 (6 to 317)	0.4 (0 to 1.6)	170 (41 to 276)	0.9 (0.2 to 1.4)	788 (182 to 1710)	4.1 (0.9 to 8.8)	182 (99 to 300)	0.9 (0.5 to 1.5)	352 (168 to 585)	1.9 (0.9 to 3.1)
	Male	3240 (2630 to 3950)	36.1 (29.3 to 44)	510 (243 to 881)	5.7 (2.7 to 9.8)	14 (4 to 36)	0.2 (0 to 0.4)	191 (106 to 307)	2.1 (1.2 to 3.4)	53 (4 to 199)	0.6 (0 to 2.2)	105 (24 to 177)	1.2 (0.3 to 2)	549 (101 to 1230)	6.1 (1.1 to 13.7)	154 (77 to 263)	1.7 (0.8 to 2.9)	239 (106 to 411)	2.6 (1.2 to 4.5)
	Female	1330 (1050 to 1620)	13.1 (10.4 to 16)	291 (139 to 508)	2.9 (1.4 to 5)	12 (4 to 28)	0.1 (0 to 0.3)	116 (64 to 181)	1.2 (0.6 to 1.8)	30 (2 to 116)	0.3 (0 to 1.1)	65 (16 to 106)	0.6 (0.2 to 1)	238 (46 to 564)	2.3 (0.4 to 5.4)	28 (15 to 50)	0.3 (0.1 to 0.5)	113 (50 to 193)	1.1 (0.5 to 2)
Cyprus	Both	353 (308 to 402)	17.6 (15.4 to 20.1)	52 (34 to 73)	2.6 (1.7 to 3.7)	0 (0 to 0)	0 (0 to 0)	22 (13 to 33)	1.1 (0.7 to 1.7)	3 (0 to 8)	0.2 (0 to 0.4)	15 (3 to 23)	0.7 (0.2 to 1.2)	61 (14 to 130)	3 (0.7 to 6.5)	108 (72 to 147)	5.3 (3.5 to 7.2)	16 (3 to 31)	0.8 (0.2 to 1.6)
	Male	294 (253 to 338)	31.3 (27 to 36.1)	40 (26 to 57)	4.3 (2.8 to 6.1)	0 (0 to 0)	0 (0 to 0)	15 (8 to 23)	1.6 (0.9 to 2.5)	2 (0 to 6)	0.2 (0 to 0.6)	11 (3 to 18)	1.2 (0.3 to 1.9)	49 (9 to 108)	5.1 (0.9 to 11.3)	101 (66 to 139)	10.9 (7.1 to 15)	14 (3 to 27)	1.4 (0.3 to 2.8)
	Female	59 (49 to 70)	5.7 (4.7 to 6.7)	12 (8 to 17)	1.1 (0.8 to 1.6)	0 (0 to 0)	0 (0 to 0)	7 (4 to 10)	0.7 (0.4 to 1)	1 (0 to 2)	0.1 (0 to 0.2)	3 (1 to 5)	0.3 (0.1 to 0.5)	12 (2 to 27)	1.2 (0.2 to 2.6)	6 (3 to 10)	0.6 (0.3 to 0.9)	2 (0 to 5)	0.2 (0 to 0.5)
Czechia	Both	4560 (3760 to 5550)	21.1 (17.3 to 25.7)	747 (506 to 1050)	3.5 (2.4 to 4.9)	9 (2 to 24)	0 (0 to 0.1)	335 (191 to 534)	1.6 (0.9 to 2.6)	618 (95 to 1440)	2.9 (0.4 to 6.7)	281 (76 to 447)	1.3 (0.4 to 2.1)	999 (227 to 2190)	4.5 (1 to 9.9)	495 (280 to 802)	2.2 (1.2 to 3.5)	279 (42 to 559)	1.4 (0.2 to 2.7)
	Male	3330 (2720	34.9 (28.6	508 (342	5.4 (3.6	5 (1 to 15)	0.1 (0 to 0.2)	239 (135	2.6 (1.5 to 4)	420 (64 to 969)	4.5 (0.7	190 (52 to 308)	2 (0.5 to 3.2)	724 (132	7.5 (1.4	435 (230	4.5 (2.4	206 (31 to 411)	2.1 (0.3

		to 4070)	to 42.7)	to 716)	to 7.6)			to 378)			to 10.3)			to 1620)	to 16.9)	to 715)	to 7.4)		to 4.3)
	Female	1230 (994 to 1510)	10.3 (8.3 to 12.6)	239 (161 to 342)	2 (1.3 to 2.9)	4 (1 to 10)	0 (0 to 0.1)	97 (55 to 154)	0.9 (0.5 to 1.4)	198 (29 to 461)	1.7 (0.2 to 3.9)	91 (26 to 146)	0.8 (0.2 to 1.2)	275 (60 to 637)	2.2 (0.5 to 5.1)	60 (31 to 101)	0.5 (0.2 to 0.8)	73 (9 to 154)	0.7 (0.1 to 1.4)
Côte d’Ivoire	Both	574 (413 to 755)	6.1 (4.4 to 8)	146 (65 to 257)	1.5 (0.7 to 2.7)	297 (178 to 449)	3.1 (1.9 to 4.7)	57 (30 to 92)	0.6 (0.3 to 0.9)	59 (3 to 236)	0.6 (0 to 2.5)	54 (13 to 89)	0.6 (0.1 to 0.9)	92 (19 to 216)	1.1 (0.2 to 2.6)	34 (15 to 66)	0.4 (0.2 to 0.8)	58 (24 to 106)	0.5 (0.2 to 0.8)
	Male	526 (375 to 695)	10.7 (7.8 to 14)	120 (53 to 211)	2.5 (1.1 to 4.4)	222 (127 to 338)	4.6 (2.6 to 7)	41 (21 to 67)	0.8 (0.4 to 1.4)	46 (3 to 184)	0.9 (0.1 to 3.8)	41 (10 to 69)	0.9 (0.2 to 1.4)	70 (11 to 176)	1.7 (0.3 to 4.2)	31 (13 to 62)	0.8 (0.3 to 1.5)	47 (19 to 88)	0.7 (0.3 to 1.3)
	Female	48 (34 to 68)	1.2 (0.8 to 1.7)	27 (12 to 49)	0.6 (0.3 to 1.1)	76 (47 to 112)	1.7 (1.1 to 2.4)	16 (8 to 26)	0.3 (0.2 to 0.5)	14 (1 to 53)	0.3 (0 to 1.2)	13 (3 to 21)	0.3 (0.1 to 0.4)	22 (4 to 53)	0.5 (0.1 to 1.3)	3 (1 to 6)	0.1 (0 to 0.2)	10 (4 to 20)	0.2 (0.1 to 0.3)
North Korea	Both	5480 (4150 to 7180)	17 (13 to 22.1)	1270 (695 to 1920)	3.9 (2.2 to 5.9)	1710 (1010 to 2600)	5.3 (3.2 to 8.1)	637 (357 to 1010)	2 (1.1 to 3.2)	443 (40 to 1510)	1.4 (0.1 to 4.6)	401 (111 to 656)	1.2 (0.3 to 2)	591 (127 to 1370)	1.9 (0.4 to 4.3)	294 (159 to 466)	0.9 (0.5 to 1.5)	610 (262 to 1090)	1.8 (0.8 to 3.1)
	Male	4850 (3580 to 6400)	38.2 (28.4 to 49.6)	900 (467 to 1420)	7 (3.7 to 10.9)	1050 (570 to 1730)	8.2 (4.5 to 13.1)	302 (166 to 509)	2.4 (1.3 to 4)	293 (26 to 984)	2.3 (0.2 to 7.6)	263 (67 to 445)	2.1 (0.5 to 3.4)	386 (61 to 973)	3.2 (0.5 to 8)	231 (111 to 392)	2 (1 to 3.4)	421 (164 to 802)	2.7 (1.1 to 5.1)
	Female	638 (427 to 873)	3.3 (2.3 to 4.6)	368 (199 to 598)	1.9 (1.1 to 3.1)	656 (390 to 977)	3.5 (2 to 5.2)	335 (185 to 536)	1.8 (1 to 2.9)	150 (14 to 514)	0.8 (0.1 to 2.7)	138 (40 to 233)	0.7 (0.2 to 1.2)	205 (39 to 512)	1.1 (0.2 to 2.7)	63 (27 to 122)	0.3 (0.1 to 0.6)	189 (71 to 360)	1 (0.4 to 1.9)
Democratic Republic of the Congo	Both	1240 (588 to 2770)	3.6 (1.7 to 8)	273 (83 to 703)	0.8 (0.2 to 2)	1220 (573 to 2570)	3.6 (1.7 to 7.3)	44 (14 to 108)	0.1 (0 to 0.3)	161 (11 to 712)	0.5 (0 to 2)	232 (60 to 564)	0.7 (0.2 to 1.6)	351 (56 to 1150)	1.1 (0.2 to 3.7)	143 (29 to 573)	0.5 (0.1 to 2.1)	236 (76 to 577)	0.5 (0.2 to 1.3)
	Male	1180 (546 to 2710)	8.1 (3.8 to 18.3)	225 (60 to 632)	1.5 (0.4 to 4.1)	918 (376 to 2260)	6.1 (2.5 to 14.7)	33 (9 to 92)	0.2 (0.1 to 0.5)	124 (8 to 583)	0.8 (0.1 to 3.8)	178 (41 to 497)	1.2 (0.3 to 3.2)	287 (36 to 1060)	2.2 (0.3 to 8)	109 (16 to 526)	1 (0.2 to 5)	186 (52 to 510)	0.9 (0.3 to 2.4)
	Female	53 (31 to 88)	0.3 (0.2 to 0.5)	48 (18 to 103)	0.3 (0.1 to 0.6)	303 (175 to 512)	1.6 (0.9 to 2.8)	11 (4 to 22)	0.1 (0 to 0.1)	37 (2 to 146)	0.2 (0 to 0.8)	54 (16 to 97)	0.3 (0.1 to 0.5)	64 (11 to 164)	0.4 (0.1 to 1)	35 (8 to 80)	0.2 (0 to 0.5)	49 (18 to 98)	0.2 (0.1 to 0.4)

Denmark	Both	3250 (2980 to 3530)	27.2 (24.9 to 29.5)	269 (152 to 418)	2.3 (1.3 to 3.5)	0 (0 to 1)	0 (0 to 0)	154 (92 to 231)	1.4 (0.8 to 2.1)	257 (37 to 592)	2.2 (0.3 to 5)	118 (27 to 180)	1 (0.2 to 1.5)	343 (80 to 754)	2.8 (0.7 to 6.1)	1120 (810 to 1440)	8.8 (6.3 to 11.5)	149 (26 to 290)	1.3 (0.2 to 2.6)
	Male	1760 (1610 to 1910)	31.8 (29.1 to 34.4)	140 (79 to 216)	2.6 (1.4 to 4)	0 (0 to 1)	0 (0 to 0)	90 (53 to 136)	1.7 (1 to 2.5)	134 (19 to 305)	2.4 (0.4 to 5.6)	58 (13 to 92)	1.1 (0.2 to 1.7)	187 (30 to 445)	3.3 (0.5 to 7.9)	956 (670 to 1250)	16.6 (11.5 to 21.9)	91 (15 to 178)	1.7 (0.3 to 3.3)
	Female	1490 (1310 to 1670)	23.5 (20.8 to 26.2)	128 (73 to 200)	2.1 (1.2 to 3.2)	0 (0 to 1)	0 (0 to 0)	64 (37 to 96)	1.1 (0.7 to 1.7)	123 (18 to 286)	2 (0.3 to 4.6)	59 (14 to 94)	0.9 (0.2 to 1.5)	155 (30 to 369)	2.4 (0.5 to 5.6)	162 (93 to 258)	2.3 (1.3 to 3.7)	58 (9 to 117)	1 (0.2 to 2.1)
Djibouti	Both	33 (19 to 58)	6.4 (4 to 10.8)	12 (5 to 23)	2.2 (1 to 4.1)	5 (2 to 9)	0.8 (0.4 to 1.6)	2 (1 to 4)	0.4 (0.2 to 0.8)	2 (0 to 8)	0.4 (0 to 1.4)	4 (1 to 8)	0.7 (0.2 to 1.5)	4 (1 to 10)	0.8 (0.1 to 2.1)	3 (1 to 12)	0.7 (0.2 to 2.8)	3 (1 to 6)	0.4 (0.1 to 0.8)
	Male	31 (18 to 56)	11.4 (7 to 19.6)	9 (4 to 20)	3.3 (1.4 to 6.6)	3 (1 to 7)	1.1 (0.4 to 2.5)	2 (1 to 3)	0.5 (0.2 to 1.2)	2 (0 to 6)	0.5 (0 to 2.2)	3 (1 to 7)	1.1 (0.3 to 2.4)	3 (0 to 9)	1.2 (0.2 to 3.7)	2 (0 to 12)	1.1 (0.2 to 5.3)	2 (1 to 5)	0.5 (0.2 to 1.2)
	Female	2 (1 to 3)	0.8 (0.5 to 1.2)	2 (1 to 4)	0.9 (0.4 to 1.6)	1 (1 to 2)	0.5 (0.2 to 0.9)	1 (0 to 1)	0.3 (0.2 to 0.6)	0 (0 to 2)	0.2 (0 to 0.7)	1 (0 to 2)	0.4 (0.1 to 0.6)	1 (0 to 2)	0.3 (0.1 to 0.8)	0 (0 to 1)	0.2 (0.1 to 0.5)	0 (0 to 1)	0.1 (0.1 to 0.3)
Dominica	Both	6 (5 to 8)	7 (5.5 to 8.7)	2 (1 to 4)	2.2 (0.9 to 4)	0 (0 to 1)	0.3 (0.1 to 0.6)	0 (0 to 1)	0.5 (0.3 to 0.8)	1 (0 to 2)	0.6 (0 to 2.2)	0 (0 to 0)	0 (0 to 0.1)	2 (1 to 5)	2.7 (0.6 to 5.9)	2 (1 to 3)	2 (1.1 to 3.1)	1 (0 to 1)	0.9 (0.4 to 1.6)
	Male	5 (4 to 7)	12.4 (9.7 to 15.5)	1 (1 to 3)	3.2 (1.3 to 5.8)	0 (0 to 0)	0.3 (0.1 to 0.8)	0 (0 to 1)	0.7 (0.4 to 1.2)	0 (0 to 1)	0.8 (0.1 to 3.3)	0 (0 to 0)	0.1 (0 to 0.1)	2 (0 to 4)	4.2 (0.8 to 9.4)	2 (1 to 3)	4.4 (2.5 to 6.9)	1 (0 to 1)	1.4 (0.6 to 2.6)
	Female	1 (1 to 1)	2 (1.5 to 2.7)	1 (0 to 1)	1.3 (0.5 to 2.4)	0 (0 to 0)	0.2 (0.1 to 0.4)	0 (0 to 0)	0.3 (0.1 to 0.5)	0 (0 to 1)	0.3 (0 to 1.3)	0 (0 to 0)	0 (0 to 0.1)	1 (0 to 2)	1.4 (0.3 to 3.2)	0 (0 to 0)	0 (0 to 0.1)	0 (0 to 0)	0.4 (0.2 to 0.8)
Dominican Republic	Both	857 (627 to 1160)	9.6 (7.1 to 12.9)	162 (61 to 310)	1.8 (0.7 to 3.4)	38 (14 to 76)	0.4 (0.2 to 0.8)	49 (26 to 83)	0.5 (0.3 to 0.9)	39 (3 to 151)	0.4 (0 to 1.7)	12 (4 to 21)	0.1 (0 to 0.2)	100 (22 to 242)	1.1 (0.2 to 2.7)	36 (17 to 65)	0.4 (0.2 to 0.8)	101 (44 to 182)	1 (0.5 to 1.9)
	Male	598 (437 to 804)	14.1 (10.4	107 (41 to 202)	2.5 (0.9	22 (7 to 44)	0.5 (0.2 to 1)	33 (17 to 56)	0.8 (0.4	25 (2 to 96)	0.6 (0 to 2.2)	7 (2 to 14)	0.2 (0.1	65 (10 to 166)	1.6 (0.3	32 (14 to 58)	0.8 (0.4	80 (32 to 149)	1.7 (0.7

			to 18.9)		to 4.6)				to 1.3)				to 0.3)		to 3.9)		to 1.5)		to 3.1)
	Female	259 (177 to 360)	5.6 (3.8 to 7.7)	55 (20 to 110)	1.2 (0.4 to 2.3)	17 (6 to 32)	0.4 (0.1 to 0.7)	16 (8 to 28)	0.3 (0.2 to 0.6)	14 (1 to 52)	0.3 (0 to 1.1)	5 (2 to 10)	0.1 (0 to 0.2)	35 (7 to 91)	0.8 (0.1 to 2)	4 (2 to 8)	0.1 (0 to 0.2)	21 (8 to 41)	0.4 (0.2 to 0.8)
Ecuador	Both	584 (456 to 749)	4.1 (3.2 to 5.2)	210 (124 to 323)	1.5 (0.9 to 2.2)	23 (8 to 50)	0.2 (0.1 to 0.3)	23 (10 to 39)	0.2 (0.1 to 0.3)	72 (6 to 250)	0.5 (0 to 1.7)	22 (6 to 38)	0.1 (0 to 0.3)	162 (38 to 363)	1.2 (0.3 to 2.6)	77 (42 to 126)	0.6 (0.3 to 1)	100 (49 to 169)	0.6 (0.3 to 1.1)
	Male	457 (355 to 585)	6.8 (5.3 to 8.7)	119 (69 to 184)	1.8 (1 to 2.7)	11 (4 to 24)	0.2 (0.1 to 0.4)	13 (5 to 22)	0.2 (0.1 to 0.3)	40 (3 to 143)	0.6 (0 to 2.1)	11 (3 to 21)	0.2 (0 to 0.3)	91 (16 to 216)	1.4 (0.2 to 3.3)	57 (29 to 98)	1 (0.5 to 1.7)	69 (31 to 121)	0.9 (0.4 to 1.6)
	Female	128 (94 to 169)	1.7 (1.2 to 2.2)	92 (54 to 141)	1.2 (0.7 to 1.8)	13 (5 to 27)	0.2 (0.1 to 0.4)	10 (4 to 17)	0.1 (0.1 to 0.2)	32 (2 to 107)	0.4 (0 to 1.4)	10 (3 to 18)	0.1 (0 to 0.2)	72 (14 to 162)	1 (0.2 to 2.2)	20 (10 to 31)	0.3 (0.1 to 0.5)	31 (14 to 55)	0.4 (0.2 to 0.7)
Egypt	Both	3390 (2360 to 4760)	5.4 (3.8 to 7.5)	1770 (1150 to 2550)	2.8 (1.8 to 4)	1 (0 to 3)	0 (0 to 0)	405 (212 to 660)	0.6 (0.3 to 1)	64 (7 to 178)	0.1 (0 to 0.3)	97 (22 to 179)	0.2 (0 to 0.3)	515 (111 to 1230)	0.9 (0.2 to 2.1)	52 (8 to 206)	0.1 (0 to 0.4)	385 (158 to 717)	0.5 (0.2 to 0.9)
	Male	3290 (2280 to 4630)	9.4 (6.6 to 13.2)	1230 (764 to 1800)	3.5 (2.2 to 5.1)	1 (0 to 2)	0 (0 to 0)	218 (107 to 369)	0.6 (0.3 to 1)	44 (5 to 121)	0.1 (0 to 0.3)	64 (15 to 122)	0.2 (0 to 0.3)	348 (59 to 890)	1 (0.2 to 2.7)	38 (3 to 184)	0.1 (0 to 0.6)	361 (145 to 681)	0.9 (0.4 to 1.7)
	Female	108 (59 to 172)	0.5 (0.3 to 0.7)	544 (318 to 849)	2 (1.2 to 3.1)	1 (0 to 1)	0 (0 to 0)	188 (94 to 320)	0.6 (0.3 to 1.1)	20 (2 to 58)	0.1 (0 to 0.2)	33 (8 to 63)	0.1 (0 to 0.2)	167 (33 to 445)	0.7 (0.1 to 1.8)	14 (3 to 39)	0.1 (0 to 0.2)	24 (9 to 48)	0.1 (0 to 0.1)
El Salvador	Both	194 (143 to 256)	3.3 (2.4 to 4.3)	85 (48 to 141)	1.4 (0.8 to 2.4)	27 (13 to 47)	0.5 (0.2 to 0.8)	14 (7 to 24)	0.2 (0.1 to 0.4)	30 (2 to 109)	0.5 (0 to 1.8)	27 (7 to 45)	0.4 (0.1 to 0.7)	74 (18 to 168)	1.2 (0.3 to 2.8)	5 (2 to 10)	0.1 (0 to 0.2)	36 (17 to 63)	0.6 (0.3 to 1.1)
	Male	139 (101 to 181)	5.6 (4.1 to 7.3)	45 (25 to 73)	1.8 (1 to 2.9)	11 (5 to 21)	0.5 (0.2 to 0.8)	8 (4 to 13)	0.3 (0.2 to 0.5)	15 (1 to 53)	0.6 (0 to 2.1)	13 (4 to 22)	0.5 (0.1 to 0.9)	41 (7 to 95)	1.6 (0.3 to 3.8)	4 (1 to 7)	0.1 (0.1 to 0.3)	23 (10 to 42)	0.9 (0.4 to 1.7)
	Female	56 (38 to 80)	1.6 (1.1 to 2.3)	40 (23 to 68)	1.2 (0.7 to 2)	16 (8 to 27)	0.5 (0.2 to 0.8)	6 (3 to 11)	0.2 (0.1 to 0.3)	15 (1 to 53)	0.4 (0 to 1.5)	13 (4 to 22)	0.4 (0.1 to 0.6)	33 (7 to 82)	1 (0.2 to 2.4)	2 (1 to 3)	0 (0 to 0.1)	13 (6 to 24)	0.4 (0.2 to 0.7)

Equatorial Guinea	Both	24 (15 to 37)	5.5 (3.5 to 8.3)	17 (8 to 29)	3.8 (2 to 6.4)	3 (1 to 7)	0.7 (0.3 to 1.5)	2 (1 to 3)	0.4 (0.2 to 0.7)	3 (0 to 11)	0.7 (0 to 2.4)	2 (1 to 4)	0.5 (0.1 to 0.9)	7 (1 to 18)	1.7 (0.4 to 4.3)	3 (1 to 11)	0.9 (0.3 to 2.9)	3 (1 to 6)	0.5 (0.2 to 1)
	Male	22 (14 to 34)	12.6 (7.8 to 19.1)	11 (5 to 19)	5.9 (2.9 to 10.1)	2 (1 to 4)	0.9 (0.3 to 2)	1 (0 to 2)	0.5 (0.2 to 1)	2 (0 to 7)	1 (0.1 to 3.6)	1 (0 to 3)	0.7 (0.2 to 1.4)	5 (1 to 13)	2.9 (0.5 to 7.6)	2 (0 to 10)	1.6 (0.4 to 6.3)	2 (1 to 4)	0.9 (0.3 to 1.8)
	Female	2 (1 to 3)	0.6 (0.3 to 1.1)	6 (3 to 11)	2.4 (1.1 to 4.1)	1 (1 to 3)	0.6 (0.2 to 1.2)	1 (0 to 1)	0.3 (0.1 to 0.5)	1 (0 to 4)	0.4 (0 to 1.6)	1 (0 to 2)	0.3 (0.1 to 0.6)	2 (0 to 6)	0.9 (0.2 to 2.4)	1 (0 to 2)	0.5 (0.2 to 1.1)	1 (0 to 2)	0.2 (0.1 to 0.5)
Eritrea	Both	62 (45 to 85)	2.4 (1.8 to 3.2)	21 (8 to 38)	0.8 (0.3 to 1.5)	53 (31 to 80)	2.1 (1.2 to 3.1)	6 (3 to 10)	0.2 (0.1 to 0.4)	8 (1 to 36)	0.3 (0 to 1.4)	12 (4 to 20)	0.5 (0.1 to 0.7)	12 (3 to 29)	0.6 (0.1 to 1.3)	11 (4 to 29)	0.5 (0.2 to 1.4)	16 (7 to 29)	0.5 (0.2 to 0.9)
	Male	61 (44 to 83)	6 (4.4 to 7.9)	15 (6 to 28)	1.5 (0.6 to 2.8)	33 (20 to 52)	3.3 (2 to 4.9)	4 (2 to 7)	0.4 (0.2 to 0.6)	6 (0 to 23)	0.5 (0 to 2.3)	8 (2 to 13)	0.7 (0.2 to 1.2)	9 (1 to 22)	1 (0.2 to 2.6)	8 (2 to 26)	1 (0.3 to 3.6)	11 (5 to 21)	0.8 (0.3 to 1.4)
	Female	1 (1 to 2)	0.1 (0 to 0.1)	6 (2 to 11)	0.4 (0.1 to 0.8)	19 (11 to 30)	1.3 (0.8 to 2)	2 (1 to 4)	0.1 (0.1 to 0.2)	3 (0 to 12)	0.2 (0 to 0.8)	4 (1 to 7)	0.3 (0.1 to 0.5)	4 (1 to 9)	0.3 (0.1 to 0.7)	3 (1 to 6)	0.2 (0.1 to 0.5)	5 (2 to 9)	0.3 (0.1 to 0.5)
Estonia	Both	511 (400 to 644)	19.7 (15.3 to 25)	20 (7 to 37)	0.7 (0.3 to 1.4)	4 (1 to 15)	0.2 (0 to 0.6)	31 (17 to 50)	1.2 (0.7 to 2)	51 (6 to 118)	1.9 (0.2 to 4.5)	32 (9 to 54)	1.2 (0.3 to 2.1)	50 (11 to 116)	1.8 (0.4 to 4.3)	46 (25 to 75)	1.6 (0.8 to 2.6)	19 (3 to 39)	0.8 (0.1 to 1.7)
	Male	405 (317 to 515)	40.7 (31.8 to 51.6)	14 (4 to 26)	1.4 (0.4 to 2.7)	3 (0 to 9)	0.3 (0 to 0.9)	22 (12 to 35)	2.2 (1.2 to 3.6)	36 (4 to 85)	3.6 (0.4 to 8.6)	23 (6 to 38)	2.3 (0.6 to 3.9)	37 (6 to 88)	3.7 (0.6 to 8.8)	41 (20 to 69)	4.1 (2.1 to 7)	14 (2 to 28)	1.4 (0.2 to 2.9)
	Female	106 (80 to 135)	6.5 (4.9 to 8.5)	6 (2 to 11)	0.4 (0.1 to 0.7)	2 (0 to 6)	0.1 (0 to 0.3)	9 (5 to 15)	0.6 (0.3 to 1)	15 (2 to 34)	0.9 (0.1 to 2.1)	10 (3 to 16)	0.6 (0.2 to 1)	14 (3 to 34)	0.8 (0.1 to 1.9)	5 (2 to 9)	0.3 (0.1 to 0.5)	5 (1 to 11)	0.4 (0.1 to 0.8)
eSwatini	Both	28 (17 to 40)	5.4 (3.3 to 7.5)	12 (6 to 20)	2.1 (1 to 3.5)	15 (7 to 25)	2.7 (1.3 to 4.4)	2 (1 to 4)	0.3 (0.1 to 0.6)	5 (0 to 18)	0.8 (0 to 3.2)	5 (2 to 10)	1 (0.3 to 1.7)	10 (2 to 25)	1.9 (0.4 to 4.7)	23 (10 to 39)	4.4 (2.1 to 7.2)	3 (1 to 5)	0.4 (0.1 to 0.7)
	Male	23 (13 to 33)	11 (6.4	9 (4 to 15)	3.8 (1.8	10 (4 to 17)	4.3 (1.9	1 (0 to 2)	0.5 (0.2 to 1)	3 (0 to 13)	1.4 (0.1	4 (1 to 7)	1.7 (0.5 to 3)	7 (1 to 18)	3.4 (0.6	21 (10 to 36)	11.4 (5.5	2 (1 to 4)	0.7 (0.2

			to 15.4)		to 6.5)		to 7.6)				to 5.8)				to 8.9)		to 18.2)		to 1.4)
	Female	5 (3 to 8)	1.9 (1.1 to 3)	3 (1 to 5)	0.9 (0.4 to 1.7)	5 (3 to 9)	1.6 (0.8 to 2.8)	1 (0 to 1)	0.2 (0.1 to 0.4)	1 (0 to 5)	0.4 (0 to 1.6)	2 (0 to 3)	0.5 (0.2 to 1)	3 (1 to 8)	1 (0.2 to 2.7)	1 (1 to 3)	0.5 (0.2 to 1.1)	1 (0 to 1)	0.1 (0 to 0.3)
Ethiopia	Both	465 (296 to 674)	1.3 (0.8 to 1.9)	123 (55 to 236)	0.3 (0.1 to 0.6)	692 (403 to 1110)	1.8 (1.1 to 3)	21 (9 to 39)	0.1 (0 to 0.1)	95 (12 to 258)	0.3 (0 to 0.7)	150 (52 to 263)	0.4 (0.1 to 0.7)	119 (23 to 296)	0.3 (0.1 to 0.8)	115 (24 to 554)	0.3 (0.1 to 1.6)	146 (64 to 264)	0.3 (0.1 to 0.6)
	Male	456 (291 to 660)	2.5 (1.6 to 3.6)	100 (43 to 193)	0.5 (0.2 to 1)	541 (320 to 851)	2.8 (1.7 to 4.5)	16 (7 to 31)	0.1 (0 to 0.2)	75 (10 to 203)	0.4 (0.1 to 1.1)	119 (42 to 207)	0.6 (0.2 to 1.1)	99 (16 to 255)	0.5 (0.1 to 1.4)	97 (16 to 525)	0.6 (0.1 to 3)	120 (51 to 219)	0.5 (0.2 to 1)
	Female	9 (5 to 17)	0.1 (0 to 0.1)	23 (11 to 46)	0.1 (0.1 to 0.2)	151 (75 to 285)	0.8 (0.4 to 1.5)	5 (2 to 11)	0 (0 to 0)	20 (2 to 58)	0.1 (0 to 0.3)	32 (10 to 63)	0.2 (0.1 to 0.3)	20 (3 to 59)	0.1 (0 to 0.3)	18 (4 to 38)	0.1 (0 to 0.2)	26 (10 to 55)	0.1 (0 to 0.2)
Fiji	Both	36 (28 to 45)	5.1 (4.1 to 6.4)	5 (1 to 12)	0.7 (0.2 to 1.7)	4 (2 to 9)	0.7 (0.3 to 1.2)	3 (2 to 6)	0.5 (0.2 to 0.8)	2 (0 to 8)	0.3 (0 to 1.2)	4 (2 to 7)	0.6 (0.2 to 1)	15 (4 to 31)	2.2 (0.6 to 4.5)	5 (3 to 9)	0.9 (0.5 to 1.4)	4 (2 to 6)	0.4 (0.2 to 0.7)
	Male	29 (23 to 37)	9.7 (7.6 to 12)	3 (1 to 8)	1.1 (0.3 to 2.6)	3 (1 to 5)	0.8 (0.3 to 1.6)	2 (1 to 3)	0.6 (0.3 to 1)	1 (0 to 5)	0.5 (0 to 1.7)	3 (1 to 5)	0.9 (0.3 to 1.5)	10 (2 to 21)	3.4 (0.7 to 7.2)	5 (3 to 9)	2.2 (1.2 to 3.5)	3 (1 to 5)	0.6 (0.3 to 1.1)
	Female	6 (5 to 9)	1.7 (1.2 to 2.3)	2 (1 to 4)	0.5 (0.1 to 1.1)	2 (1 to 4)	0.5 (0.2 to 1)	1 (1 to 3)	0.4 (0.2 to 0.7)	1 (0 to 3)	0.2 (0 to 0.8)	2 (1 to 3)	0.4 (0.1 to 0.7)	5 (1 to 11)	1.4 (0.3 to 3.1)	0 (0 to 0)	0.1 (0 to 0.1)	1 (0 to 2)	0.2 (0.1 to 0.4)
Finland	Both	1650 (1500 to 1790)	13 (11.9 to 14.1)	48 (9 to 105)	0.4 (0.1 to 0.8)	0 (0 to 1)	0 (0 to 0)	62 (36 to 96)	0.5 (0.3 to 0.8)	200 (30 to 447)	1.6 (0.2 to 3.5)	115 (32 to 171)	0.9 (0.2 to 1.4)	297 (72 to 638)	2.2 (0.5 to 4.9)	719 (490 to 964)	5.2 (3.5 to 7)	90 (16 to 173)	0.8 (0.1 to 1.5)
	Male	1170 (1070 to 1280)	20.6 (18.7 to 22.4)	32 (6 to 69)	0.6 (0.1 to 1.2)	0 (0 to 1)	0 (0 to 0)	42 (24 to 66)	0.8 (0.4 to 1.2)	131 (20 to 293)	2.3 (0.3 to 5.2)	75 (20 to 112)	1.3 (0.4 to 2)	196 (34 to 453)	3.4 (0.6 to 7.8)	646 (436 to 866)	10.9 (7.3 to 14.6)	68 (12 to 132)	1.2 (0.2 to 2.4)
	Female	475 (414 to 536)	7 (6.2 to 7.8)	17 (3 to 36)	0.2 (0 to 0.5)	0 (0 to 0)	0 (0 to 0)	20 (11 to 31)	0.3 (0.2 to 0.5)	69 (10 to 154)	1 (0.1 to 2.2)	40 (11 to 61)	0.6 (0.2 to 0.9)	101 (21 to 232)	1.4 (0.3 to 3.2)	74 (39 to 114)	0.9 (0.5 to 1.4)	22 (3 to 44)	0.4 (0.1 to 0.7)

France	Both	26800 (24600 to 29000)	21 (19.4 to 22.6)	3090 (1980 to 4470)	2.4 (1.6 to 3.5)	8 (2 to 24)	0 (0 to 0)	1090 (611 to 1700)	1 (0.5 to 1.5)	2030 (335 to 4550)	1.6 (0.3 to 3.6)	1760 (480 to 2630)	1.4 (0.4 to 2.1)	2300 (482 to 5340)	1.7 (0.3 to 3.9)	12400 (8730 to 15900)	8.8 (6.1 to 11.5)	1480 (256 to 2860)	1.3 (0.2 to 2.5)
	Male	20600 (18800 to 22300)	35.4 (32.6 to 38.4)	2220 (1400 to 3210)	3.9 (2.4 to 5.6)	5 (1 to 14)	0 (0 to 0)	791 (442 to 1240)	1.5 (0.8 to 2.3)	1460 (239 to 3290)	2.5 (0.4 to 5.7)	1260 (328 to 1880)	2.2 (0.6 to 3.3)	1730 (273 to 4160)	2.9 (0.4 to 6.9)	11400 (7980 to 14900)	18.6 (12.8 to 24.4)	1210 (202 to 2350)	2.2 (0.4 to 4.3)
	Female	6260 (5470 to 6980)	9.1 (8.1 to 10.1)	866 (547 to 1250)	1.2 (0.8 to 1.8)	3 (1 to 8)	0 (0 to 0)	296 (165 to 462)	0.5 (0.3 to 0.8)	570 (97 to 1280)	0.8 (0.1 to 1.8)	502 (141 to 763)	0.7 (0.2 to 1.1)	574 (106 to 1420)	0.7 (0.1 to 1.8)	909 (421 to 1410)	1 (0.5 to 1.6)	268 (39 to 545)	0.5 (0.1 to 0.9)
Gabon	Both	68 (45 to 99)	6.7 (4.6 to 9.6)	43 (23 to 73)	4.2 (2.2 to 7.1)	3 (1 to 7)	0.3 (0.1 to 0.7)	5 (2 to 9)	0.5 (0.2 to 0.8)	7 (0 to 27)	0.7 (0 to 2.7)	3 (1 to 6)	0.3 (0.1 to 0.6)	21 (4 to 53)	2.2 (0.5 to 5.4)	11 (4 to 30)	1.3 (0.6 to 3.4)	6 (2 to 11)	0.4 (0.2 to 0.8)
	Male	64 (43 to 94)	13.9 (9.6 to 20.1)	31 (16 to 54)	6.7 (3.5 to 11.3)	2 (1 to 5)	0.5 (0.1 to 1)	3 (1 to 6)	0.7 (0.3 to 1.2)	5 (0 to 20)	1.1 (0.1 to 4.5)	2 (1 to 5)	0.5 (0.1 to 1)	16 (3 to 42)	3.8 (0.7 to 9.7)	9 (3 to 28)	2.6 (0.9 to 7.5)	4 (2 to 9)	0.7 (0.3 to 1.4)
	Female	4 (2 to 6)	0.7 (0.4 to 1.2)	11 (6 to 20)	2.2 (1.1 to 3.7)	1 (0 to 3)	0.2 (0.1 to 0.5)	2 (1 to 3)	0.3 (0.1 to 0.5)	2 (0 to 7)	0.4 (0 to 1.4)	1 (0 to 2)	0.2 (0 to 0.4)	4 (1 to 11)	0.9 (0.2 to 2.2)	2 (1 to 4)	0.4 (0.1 to 0.9)	1 (0 to 2)	0.2 (0.1 to 0.4)
The Gambia	Both	28 (21 to 35)	3.1 (2.4 to 4)	7 (3 to 12)	0.8 (0.3 to 1.4)	19 (11 to 28)	2.1 (1.3 to 3.1)	3 (2 to 5)	0.4 (0.2 to 0.6)	3 (0 to 12)	0.4 (0 to 1.3)	5 (2 to 8)	0.5 (0.2 to 0.9)	5 (1 to 12)	0.6 (0.1 to 1.4)	2 (1 to 4)	0.2 (0.1 to 0.5)	3 (1 to 5)	0.3 (0.1 to 0.5)
	Male	27 (20 to 34)	6.3 (4.9 to 8)	5 (2 to 9)	1.2 (0.5 to 2)	12 (7 to 17)	2.8 (1.6 to 4.1)	2 (1 to 3)	0.4 (0.2 to 0.7)	2 (0 to 8)	0.5 (0 to 1.8)	3 (1 to 5)	0.7 (0.3 to 1.2)	3 (1 to 8)	0.8 (0.1 to 2)	2 (1 to 3)	0.4 (0.2 to 0.9)	2 (1 to 4)	0.4 (0.2 to 0.8)
	Female	1 (1 to 2)	0.2 (0.1 to 0.4)	2 (1 to 4)	0.4 (0.2 to 0.8)	7 (4 to 11)	1.5 (0.8 to 2.3)	1 (1 to 2)	0.3 (0.1 to 0.5)	1 (0 to 4)	0.2 (0 to 0.8)	2 (1 to 3)	0.4 (0.1 to 0.6)	2 (0 to 5)	0.4 (0.1 to 1)	0 (0 to 1)	0.1 (0 to 0.2)	1 (0 to 1)	0.1 (0 to 0.2)
Georgia	Both	1270 (1050 to 1500)	21.8 (18 to 25.7)	206 (130 to 304)	3.5 (2.2 to 5.2)	92 (34 to 185)	1.6 (0.6 to 3.2)	130 (78 to 197)	2.2 (1.3 to 3.4)	120 (8 to 434)	2.1 (0.1 to 7.5)	83 (24 to 133)	1.4 (0.4 to 2.3)	184 (38 to 425)	3.1 (0.6 to 7.1)	190 (102 to 304)	3.2 (1.7 to 5.1)	83 (39 to 137)	1.5 (0.7 to 2.4)
	Male	1210 (1000	49.5 (41 to 58.5)	175 (111	7.2 (4.6	73 (26 to 150)	3 (1.1 to 6.2)	100 (60 to 154)	4.1 (2.5	101 (7 to 368)	4.2 (0.3	70 (20 to 113)	2.9 (0.8	158 (27 to 366)	6.4 (1.1	186 (99 to 300)	7.6 (4.1	75 (34 to 125)	2.9 (1.3

		to 1430)		to 260)	to 10.7)				to 6.3)		to 15.1)		to 4.7)		to 14.9)		to 12.2)		to 4.9)
	Female	59 (44 to 76)	1.7 (1.3 to 2.2)	30 (19 to 45)	0.9 (0.5 to 1.3)	19 (7 to 36)	0.5 (0.2 to 1)	30 (18 to 44)	0.9 (0.5 to 1.3)	19 (1 to 69)	0.5 (0 to 2)	13 (4 to 21)	0.4 (0.1 to 0.6)	26 (5 to 61)	0.7 (0.1 to 1.7)	4 (1 to 7)	0.1 (0 to 0.2)	8 (4 to 14)	0.3 (0.1 to 0.4)
Germany	Both	38200 (35400 to 40800)	20.6 (19.2 to 21.9)	4350 (2800 to 6300)	2.3 (1.5 to 3.4)	5 (1 to 14)	0 (0 to 0)	1660 (964 to 2540)	1 (0.6 to 1.5)	2850 (528 to 6380)	1.5 (0.3 to 3.4)	2120 (488 to 3230)	1.1 (0.3 to 1.7)	6790 (1640 to 14400)	3.5 (0.8 to 7.4)	13300 (9200 to 17600)	6.3 (4.3 to 8.4)	2280 (372 to 4410)	1.4 (0.2 to 2.6)
	Male	26700 (24600 to 28700)	31.1 (28.8 to 33.4)	2840 (1840 to 4110)	3.3 (2.1 to 4.8)	2 (0 to 8)	0 (0 to 0)	1110 (640 to 1700)	1.4 (0.8 to 2.1)	1860 (348 to 4140)	2.2 (0.4 to 4.8)	1380 (328 to 2100)	1.6 (0.4 to 2.4)	4570 (813 to 10400)	5.2 (0.9 to 11.7)	12000 (8080 to 16100)	13 (8.7 to 17.4)	1680 (260 to 3270)	2.1 (0.3 to 4)
	Female	11600 (10300 to 12700)	11.9 (10.6 to 13)	1510 (955 to 2170)	1.5 (1 to 2.2)	2 (0 to 6)	0 (0 to 0)	553 (315 to 838)	0.7 (0.4 to 1)	987 (180 to 2220)	1 (0.2 to 2.2)	747 (171 to 1150)	0.8 (0.2 to 1.2)	2230 (456 to 5010)	2.1 (0.4 to 4.7)	1300 (735 to 1900)	1.1 (0.6 to 1.6)	593 (86 to 1190)	0.7 (0.1 to 1.4)
Ghana	Both	317 (242 to 406)	2.2 (1.7 to 2.8)	247 (144 to 373)	1.6 (1 to 2.5)	195 (109 to 310)	1.3 (0.7 to 2)	22 (11 to 36)	0.1 (0.1 to 0.2)	76 (10 to 220)	0.5 (0.1 to 1.4)	24 (6 to 40)	0.2 (0 to 0.3)	112 (25 to 258)	0.8 (0.2 to 1.9)	38 (20 to 66)	0.3 (0.2 to 0.5)	75 (34 to 129)	0.4 (0.2 to 0.7)
	Male	287 (220 to 370)	4.7 (3.6 to 5.9)	179 (102 to 274)	2.8 (1.6 to 4.2)	124 (66 to 205)	1.9 (1 to 3.2)	15 (7 to 24)	0.2 (0.1 to 0.3)	52 (7 to 151)	0.8 (0.1 to 2.3)	16 (4 to 27)	0.2 (0.1 to 0.4)	81 (14 to 193)	1.4 (0.2 to 3.3)	33 (17 to 59)	0.6 (0.3 to 1.1)	53 (22 to 96)	0.6 (0.3 to 1.1)
	Female	30 (20 to 43)	0.4 (0.3 to 0.6)	67 (38 to 107)	0.8 (0.4 to 1.3)	71 (41 to 111)	0.8 (0.5 to 1.3)	8 (4 to 13)	0.1 (0 to 0.1)	24 (3 to 69)	0.3 (0 to 0.8)	9 (2 to 15)	0.1 (0 to 0.2)	31 (6 to 77)	0.4 (0.1 to 1)	4 (2 to 9)	0.1 (0 to 0.1)	22 (9 to 41)	0.2 (0.1 to 0.4)
Greece	Both	6960 (6450 to 7420)	30.4 (28.4 to 32.3)	873 (585 to 1230)	3.8 (2.5 to 5.3)	6 (1 to 15)	0 (0 to 0.1)	467 (277 to 694)	2.2 (1.3 to 3.2)	669 (112 to 1480)	2.9 (0.5 to 6.4)	96 (26 to 167)	0.4 (0.1 to 0.7)	823 (169 to 1850)	3.4 (0.7 to 7.7)	1020 (604 to 1530)	3.9 (2.3 to 5.9)	217 (38 to 422)	1.1 (0.2 to 2.1)
	Male	5710 (5300 to 6120)	54 (50.3 to 57.6)	684 (460 to 961)	6.5 (4.3 to 9.1)	4 (1 to 10)	0 (0 to 0.1)	355 (207 to 533)	3.6 (2.1 to 5.3)	524 (89 to 1160)	5 (0.8 to 10.9)	70 (19 to 128)	0.6 (0.2 to 1.2)	674 (117 to 1570)	6.1 (1 to 14.4)	967 (556 to 1460)	8.2 (4.7 to 12.5)	188 (32 to 366)	2 (0.3 to 3.9)
	Female	1240 (1100 to 1370)	10.3 (9.2 to 11.2)	189 (125 to 263)	1.5 (1 to 2.1)	2 (0 to 4)	0 (0 to 0)	112 (68 to 169)	1 (0.6 to 1.5)	145 (24 to 321)	1.2 (0.2 to 2.6)	26 (6 to 46)	0.2 (0 to 0.3)	149 (28 to 347)	1.1 (0.2 to 2.5)	55 (20 to 122)	0.4 (0.1 to 0.9)	29 (4 to 59)	0.3 (0 to 0.6)

Greenland	Both	41 (33 to 48)	59.8 (48.8 to 70.9)	2 (0 to 5)	2.3 (0.2 to 6.9)	0 (0 to 0)	0.1 (0 to 0.4)	3 (2 to 5)	4.4 (2.4 to 7.1)	4 (0 to 11)	5.8 (0.5 to 16.6)	2 (0 to 3)	2.5 (0.6 to 4)	4 (1 to 9)	6.2 (1.3 to 14)	10 (6 to 14)	16.3 (10.6 to 22.4)	2 (0 to 5)	3 (0.6 to 5.8)
	Male	25 (20 to 31)	70.9 (56.5 to 84.2)	1 (0 to 3)	2.7 (0.2 to 7.9)	0 (0 to 0)	0.1 (0 to 0.4)	2 (1 to 3)	5.7 (3 to 9.1)	2 (0 to 7)	6.7 (0.6 to 19.1)	1 (0 to 2)	2.8 (0.6 to 4.8)	3 (0 to 7)	8.5 (1.4 to 20.3)	8 (5 to 12)	28.1 (18.4 to 38.6)	2 (0 to 3)	3.8 (0.7 to 7.5)
	Female	15 (12 to 19)	48 (38.5 to 59.1)	1 (0 to 2)	1.9 (0.2 to 5.5)	0 (0 to 0)	0.1 (0 to 0.4)	1 (1 to 2)	3 (1.6 to 4.8)	2 (0 to 4)	4.8 (0.4 to 13.8)	1 (0 to 1)	2.1 (0.5 to 3.5)	1 (0 to 3)	3.7 (0.7 to 9.6)	1 (1 to 3)	4.7 (2.4 to 8.2)	1 (0 to 1)	2 (0.4 to 4)
Grenada	Both	7 (6 to 8)	6.1 (5.3 to 7)	2 (1 to 4)	2.1 (0.8 to 3.7)	0 (0 to 0)	0.1 (0 to 0.2)	0 (0 to 1)	0.4 (0.2 to 0.6)	0 (0 to 2)	0.4 (0 to 1.5)	0 (0 to 1)	0.4 (0.1 to 0.6)	2 (1 to 5)	2.1 (0.5 to 4.4)	1 (0 to 1)	0.7 (0.4 to 1)	1 (1 to 2)	1 (0.5 to 1.6)
	Male	6 (5 to 7)	11.2 (9.8 to 12.7)	2 (1 to 3)	3.1 (1.2 to 5.4)	0 (0 to 0)	0.1 (0 to 0.2)	0 (0 to 0)	0.5 (0.3 to 0.9)	0 (0 to 1)	0.6 (0 to 2.3)	0 (0 to 0)	0.5 (0.1 to 0.8)	2 (0 to 4)	3.3 (0.6 to 7.1)	1 (0 to 1)	1.3 (0.7 to 2)	1 (0 to 2)	1.5 (0.7 to 2.5)
	Female	1 (1 to 1)	1.8 (1.4 to 2.3)	1 (0 to 1)	1.3 (0.5 to 2.3)	0 (0 to 0)	0.1 (0 to 0.2)	0 (0 to 0)	0.2 (0.1 to 0.4)	0 (0 to 1)	0.2 (0 to 0.9)	0 (0 to 0)	0.2 (0.1 to 0.4)	1 (0 to 2)	1.2 (0.2 to 2.7)	0 (0 to 0)	0.3 (0.2 to 0.5)	0 (0 to 1)	0.5 (0.2 to 0.8)
Guam	Both	32 (25 to 39)	16.8 (13.5 to 20.4)	3 (1 to 5)	1.6 (0.6 to 2.8)	0 (0 to 1)	0.2 (0 to 0.6)	4 (2 to 6)	2 (1.1 to 3.3)	2 (0 to 7)	0.9 (0.1 to 3.5)	2 (0 to 3)	0.9 (0.2 to 1.5)	6 (1 to 14)	3.4 (0.8 to 7.5)	3 (2 to 5)	1.6 (0.9 to 2.7)	2 (1 to 4)	1.1 (0.5 to 1.9)
	Male	24 (19 to 29)	26.7 (21.6 to 32.5)	2 (1 to 4)	2.2 (0.8 to 4)	0 (0 to 1)	0.3 (0 to 0.8)	2 (1 to 4)	2.8 (1.5 to 4.6)	1 (0 to 4)	1.3 (0.1 to 5)	1 (0 to 2)	1.2 (0.3 to 2.1)	4 (1 to 10)	4.9 (0.8 to 11.4)	3 (2 to 5)	3.7 (2.1 to 6)	2 (1 to 3)	1.7 (0.7 to 2.9)
	Female	8 (6 to 10)	8 (6.1 to 10.3)	1 (0 to 2)	1 (0.4 to 1.9)	0 (0 to 1)	0.2 (0 to 0.5)	1 (1 to 2)	1.4 (0.7 to 2.3)	1 (0 to 2)	0.6 (0 to 2.2)	1 (0 to 1)	0.6 (0.1 to 1)	2 (0 to 5)	2.1 (0.4 to 5.2)	0 (0 to 0)	0 (0 to 0.1)	1 (0 to 1)	0.6 (0.3 to 1)
Guatemala	Both	294 (219 to 381)	2.8 (2.1 to 3.6)	100 (52 to 158)	0.9 (0.5 to 1.5)	132 (74 to 206)	1.2 (0.7 to 1.9)	18 (8 to 31)	0.2 (0.1 to 0.3)	46 (3 to 167)	0.4 (0 to 1.6)	38 (10 to 62)	0.4 (0.1 to 0.6)	114 (27 to 254)	1.1 (0.3 to 2.4)	15 (8 to 26)	0.2 (0.1 to 0.3)	56 (26 to 94)	0.5 (0.2 to 0.8)
	Male	247 (183 to 322)	5.3 (4 to 6.8)	64 (34 to 103)	1.4 (0.7	72 (38 to 115)	1.5 (0.8	10 (5 to 18)	0.2 (0.1	28 (2 to 99)	0.6 (0 to 2.1)	23 (6 to 36)	0.5 (0.1	71 (13 to 165)	1.5 (0.3	13 (6 to 23)	0.3 (0.2	41 (18 to 71)	0.8 (0.3

					to 2.2)		to 2.4)		to 0.4)				to 0.8)		to 3.5)		to 0.6)		to 1.4)
	Female	47 (32 to 65)	0.8 (0.6 to 1.2)	35 (18 to 57)	0.6 (0.3 to 1)	60 (36 to 90)	1 (0.6 to 1.5)	7 (3 to 13)	0.1 (0.1 to 0.2)	19 (1 to 68)	0.3 (0 to 1.1)	16 (4 to 25)	0.3 (0.1 to 0.4)	44 (9 to 100)	0.8 (0.2 to 1.7)	2 (1 to 5)	0 (0 to 0.1)	15 (6 to 26)	0.2 (0.1 to 0.4)
Guinea	Both	263 (198 to 341)	5 (3.8 to 6.4)	36 (13 to 72)	0.7 (0.3 to 1.3)	171 (105 to 258)	3.2 (2 to 4.8)	17 (8 to 28)	0.3 (0.2 to 0.5)	26 (2 to 97)	0.5 (0 to 1.8)	20 (5 to 34)	0.4 (0.1 to 0.6)	36 (7 to 87)	0.7 (0.2 to 1.8)	14 (6 to 28)	0.3 (0.1 to 0.6)	21 (9 to 39)	0.3 (0.1 to 0.6)
	Male	253 (190 to 328)	9.5 (7.2 to 12.2)	30 (11 to 60)	1.1 (0.4 to 2.2)	131 (80 to 199)	4.9 (3 to 7.4)	12 (6 to 21)	0.5 (0.2 to 0.7)	20 (2 to 76)	0.8 (0.1 to 2.8)	16 (4 to 27)	0.6 (0.1 to 1)	28 (5 to 69)	1.1 (0.2 to 2.8)	12 (5 to 26)	0.5 (0.2 to 1)	18 (7 to 34)	0.6 (0.2 to 1.1)
	Female	11 (6 to 15)	0.4 (0.3 to 0.6)	6 (2 to 13)	0.2 (0.1 to 0.5)	40 (24 to 62)	1.5 (0.9 to 2.4)	5 (2 to 8)	0.2 (0.1 to 0.3)	6 (0 to 21)	0.2 (0 to 0.8)	5 (1 to 8)	0.2 (0 to 0.3)	8 (1 to 20)	0.3 (0.1 to 0.8)	1 (0 to 3)	0.1 (0 to 0.1)	3 (1 to 7)	0.1 (0 to 0.2)
Guinea-Bissau	Both	22 (13 to 34)	3.3 (2 to 5)	7 (2 to 14)	1 (0.4 to 2.1)	27 (14 to 43)	4 (2.2 to 6.4)	3 (1 to 5)	0.4 (0.2 to 0.7)	4 (0 to 16)	0.6 (0 to 2.3)	5 (1 to 8)	0.7 (0.2 to 1.3)	6 (1 to 16)	1.1 (0.2 to 2.6)	3 (1 to 6)	0.5 (0.2 to 1)	4 (2 to 8)	0.5 (0.2 to 1)
	Male	21 (12 to 33)	7 (4.3 to 10.8)	5 (2 to 12)	1.8 (0.6 to 3.9)	19 (10 to 33)	6.6 (3.4 to 11.1)	2 (1 to 4)	0.6 (0.3 to 1.2)	3 (0 to 12)	1 (0.1 to 4.1)	3 (1 to 7)	1.2 (0.3 to 2.2)	5 (1 to 13)	1.8 (0.3 to 4.9)	3 (1 to 6)	1.1 (0.5 to 2.2)	3 (1 to 7)	0.9 (0.3 to 1.8)
	Female	1 (1 to 1)	0.3 (0.2 to 0.4)	1 (1 to 3)	0.4 (0.1 to 0.7)	7 (5 to 11)	2 (1.3 to 3)	1 (0 to 2)	0.2 (0.1 to 0.4)	1 (0 to 4)	0.3 (0 to 1)	1 (0 to 2)	0.3 (0.1 to 0.6)	2 (0 to 4)	0.5 (0.1 to 1.2)	0 (0 to 1)	0.1 (0 to 0.2)	1 (0 to 2)	0.2 (0.1 to 0.4)
Guyana	Both	22 (17 to 28)	3.6 (2.7 to 4.5)	7 (3 to 14)	1.2 (0.4 to 2.2)	1 (0 to 2)	0.1 (0.1 to 0.3)	2 (1 to 3)	0.3 (0.2 to 0.5)	2 (0 to 6)	0.2 (0 to 1)	3 (1 to 4)	0.4 (0.1 to 0.7)	8 (2 to 18)	1.3 (0.3 to 3)	3 (1 to 5)	0.5 (0.3 to 0.8)	4 (2 to 7)	0.5 (0.3 to 0.9)
	Male	18 (14 to 24)	6.4 (4.9 to 8.1)	5 (2 to 9)	1.6 (0.6 to 3)	0 (0 to 1)	0.2 (0.1 to 0.3)	1 (1 to 2)	0.4 (0.2 to 0.7)	1 (0 to 4)	0.3 (0 to 1.3)	2 (1 to 3)	0.6 (0.2 to 1)	5 (1 to 12)	1.9 (0.3 to 4.3)	2 (1 to 4)	0.8 (0.4 to 1.5)	3 (1 to 5)	0.9 (0.4 to 1.5)
	Female	4 (3 to 5)	1.2 (0.9 to 1.6)	3 (1 to 5)	0.8 (0.3 to 1.5)	0 (0 to 1)	0.1 (0.1 to 0.3)	1 (0 to 1)	0.2 (0.1 to 0.4)	1 (0 to 2)	0.2 (0 to 0.7)	1 (0 to 2)	0.3 (0.1 to 0.5)	3 (1 to 7)	0.9 (0.2 to 2.1)	1 (0 to 1)	0.2 (0.1 to 0.4)	1 (0 to 2)	0.3 (0.1 to 0.5)

Haiti	Both	217 (128 to 389)	3.3 (1.9 to 5.8)	38 (12 to 90)	0.6 (0.2 to 1.4)	221 (121 to 379)	3.3 (1.9 to 5.7)	15 (6 to 31)	0.2 (0.1 to 0.4)	22 (2 to 80)	0.3 (0 to 1.2)	34 (8 to 67)	0.5 (0.1 to 1)	93 (20 to 235)	1.5 (0.3 to 3.8)	57 (20 to 123)	1 (0.4 to 2.2)	45 (18 to 91)	0.6 (0.2 to 1.2)
	Male	192 (108 to 363)	6.1 (3.5 to 11.4)	30 (8 to 75)	1 (0.3 to 2.4)	151 (76 to 295)	4.9 (2.5 to 9.6)	11 (4 to 23)	0.3 (0.1 to 0.7)	15 (1 to 57)	0.5 (0 to 1.9)	24 (5 to 52)	0.8 (0.2 to 1.7)	63 (10 to 182)	2.2 (0.3 to 6.3)	51 (17 to 115)	2 (0.7 to 4.3)	33 (11 to 75)	0.9 (0.3 to 2)
	Female	25 (16 to 38)	0.7 (0.5 to 1.1)	9 (3 to 20)	0.2 (0.1 to 0.5)	70 (42 to 106)	1.9 (1.2 to 2.9)	4 (2 to 8)	0.1 (0.1 to 0.2)	6 (0 to 24)	0.2 (0 to 0.7)	10 (3 to 18)	0.3 (0.1 to 0.5)	30 (6 to 72)	0.9 (0.2 to 2.1)	6 (2 to 16)	0.2 (0 to 0.5)	11 (4 to 23)	0.3 (0.1 to 0.5)
Honduras	Both	529 (330 to 762)	9.2 (5.7 to 13.1)	112 (51 to 210)	1.9 (0.9 to 3.6)	250 (131 to 400)	4.3 (2.3 to 6.8)	57 (25 to 101)	1 (0.4 to 1.7)	68 (5 to 242)	1.2 (0.1 to 4.2)	56 (14 to 100)	1 (0.2 to 1.7)	166 (37 to 407)	3 (0.7 to 7.2)	80 (42 to 142)	1.5 (0.8 to 2.6)	100 (39 to 188)	1.5 (0.6 to 2.9)
	Male	354 (213 to 507)	13.1 (8 to 18.8)	53 (24 to 94)	2 (0.9 to 3.5)	93 (44 to 152)	3.4 (1.6 to 5.5)	22 (10 to 39)	0.8 (0.3 to 1.4)	28 (2 to 103)	1 (0.1 to 3.8)	23 (5 to 41)	0.8 (0.2 to 1.5)	76 (13 to 199)	2.9 (0.5 to 7.5)	52 (25 to 92)	2 (1 to 3.6)	51 (18 to 98)	1.7 (0.6 to 3.2)
	Female	176 (99 to 278)	5.7 (3.3 to 9)	58 (26 to 115)	1.9 (0.8 to 3.7)	157 (78 to 258)	5.1 (2.6 to 8.3)	35 (14 to 65)	1.1 (0.5 to 2.1)	40 (3 to 145)	1.3 (0.1 to 4.7)	33 (9 to 63)	1.1 (0.3 to 2)	90 (17 to 242)	3 (0.6 to 8.1)	28 (9 to 58)	1 (0.3 to 2)	49 (17 to 103)	1.4 (0.5 to 3)
Hungary	Both	6690 (5470 to 8130)	35.8 (29.2 to 43.4)	1040 (704 to 1450)	5.6 (3.8 to 7.8)	192 (49 to 460)	1 (0.3 to 2.5)	629 (357 to 989)	3.4 (1.9 to 5.4)	654 (103 to 1580)	3.5 (0.6 to 8.5)	396 (113 to 628)	2.1 (0.6 to 3.4)	953 (218 to 2140)	4.9 (1.1 to 11)	488 (270 to 781)	2.4 (1.3 to 3.9)	365 (55 to 740)	2.1 (0.3 to 4.2)
	Male	4420 (3590 to 5390)	55.9 (45.5 to 68.3)	643 (432 to 901)	8.2 (5.5 to 11.5)	99 (24 to 240)	1.3 (0.3 to 3.1)	374 (213 to 602)	4.8 (2.7 to 7.7)	401 (64 to 978)	5.1 (0.8 to 12.5)	241 (65 to 383)	3.1 (0.8 to 4.9)	640 (109 to 1510)	8 (1.4 to 19)	404 (207 to 681)	4.9 (2.5 to 8.3)	245 (35 to 498)	3.1 (0.4 to 6.2)
	Female	2270 (1840 to 2780)	21.6 (17.4 to 26.4)	395 (265 to 560)	3.7 (2.5 to 5.2)	93 (26 to 224)	0.9 (0.2 to 2.1)	254 (144 to 399)	2.5 (1.4 to 3.9)	252 (39 to 613)	2.4 (0.4 to 5.7)	155 (44 to 245)	1.5 (0.4 to 2.3)	313 (60 to 756)	2.7 (0.5 to 6.5)	84 (45 to 142)	0.7 (0.4 to 1.2)	120 (15 to 252)	1.2 (0.2 to 2.6)
Iceland	Both	100 (88 to 112)	18 (15.9 to 20.2)	3 (1 to 7)	0.5 (0.1 to 1.2)	0 (0 to 0)	0 (0 to 0)	7 (4 to 10)	1.3 (0.8 to 1.9)	2 (0 to 4)	0.3 (0 to 0.8)	6 (2 to 9)	1.1 (0.3 to 1.7)	13 (3 to 28)	2.2 (0.5 to 4.9)	30 (21 to 39)	5.1 (3.6 to 6.8)	6 (1 to 12)	1.2 (0.2 to 2.3)
	Male	56 (50 to 63)	21.3 (18.9	2 (0 to 3)	0.6 (0.1	0 (0 to 0)	0 (0 to 0)	4 (2 to 6)	1.5 (0.9	1 (0 to 2)	0.3 (0 to 0.8)	3 (1 to 5)	1.2 (0.3	7 (1 to 17)	2.7 (0.4	26 (18 to 35)	9.7 (6.6 to 13)	4 (1 to 8)	1.5 (0.3 to 3)

			to 23.9)		to 1.3)				to 2.2)				to 1.8)		to 6.3)				
	Female	43 (37 to 49)	14.9 (12.8 to 17)	1 (0 to 3)	0.5 (0.1 to 1.1)	0 (0 to 0)	0 (0 to 0)	3 (2 to 4)	1.1 (0.6 to 1.6)	1 (0 to 2)	0.3 (0 to 0.7)	3 (1 to 5)	1 (0.3 to 1.5)	5 (1 to 13)	1.7 (0.3 to 4.1)	3 (2 to 5)	1.1 (0.6 to 1.7)	2 (0 to 4)	0.8 (0.1 to 1.6)
India	Both	41000 (33100 to 50100)	3.8 (3.1 to 4.6)	19000 (13100 to 25500)	1.7 (1.2 to 2.3)	9150 (5370 to 13800)	0.8 (0.5 to 1.2)	4320 (2310 to 6850)	0.4 (0.2 to 0.6)	3970 (832 to 8230)	0.4 (0.1 to 0.7)	5880 (2220 to 9120)	0.5 (0.2 to 0.8)	8860 (1980 to 20200)	0.8 (0.2 to 1.9)	4920 (2920 to 7810)	0.5 (0.3 to 0.8)	5900 (2780 to 9520)	0.5 (0.2 to 0.8)
	Male	37000 (29100 to 45500)	7.1 (5.6 to 8.7)	13700 (9160 to 18900)	2.6 (1.7 to 3.5)	5910 (3230 to 9230)	1.1 (0.6 to 1.7)	2590 (1330 to 4220)	0.5 (0.3 to 0.8)	2780 (553 to 5770)	0.5 (0.1 to 1.1)	4110 (1500 to 6570)	0.8 (0.3 to 1.2)	6470 (1160 to 15500)	1.2 (0.2 to 3)	4380 (2460 to 7150)	1 (0.5 to 1.5)	5190 (2380 to 8500)	0.8 (0.4 to 1.4)
	Female	3990 (2950 to 5210)	0.7 (0.6 to 1)	5250 (3460 to 7250)	0.9 (0.6 to 1.3)	3240 (1880 to 4940)	0.6 (0.3 to 0.9)	1730 (911 to 2760)	0.3 (0.2 to 0.5)	1190 (234 to 2490)	0.2 (0 to 0.4)	1770 (667 to 2860)	0.3 (0.1 to 0.5)	2400 (480 to 5810)	0.4 (0.1 to 1)	544 (270 to 976)	0.1 (0.1 to 0.2)	705 (293 to 1220)	0.1 (0 to 0.2)
Indonesia	Both	27900 (21100 to 35700)	14 (10.7 to 17.6)	5670 (3490 to 8390)	2.8 (1.7 to 4.2)	3710 (1760 to 6470)	1.8 (0.9 to 3.2)	2590 (1250 to 4370)	1.3 (0.6 to 2.1)	970 (174 to 2270)	0.5 (0.1 to 1.1)	2260 (682 to 3710)	1.1 (0.3 to 1.8)	3440 (702 to 8310)	1.8 (0.4 to 4.4)	1370 (758 to 2250)	0.8 (0.5 to 1.4)	3290 (1570 to 5430)	1.3 (0.6 to 2.2)
	Male	25600 (18900 to 33600)	27.7 (21 to 35.4)	3990 (2400 to 5990)	4.2 (2.6 to 6.3)	2300 (998 to 4180)	2.4 (1.1 to 4.4)	1180 (598 to 1950)	1.3 (0.6 to 2.1)	662 (123 to 1610)	0.7 (0.1 to 1.7)	1540 (463 to 2650)	1.6 (0.5 to 2.8)	2430 (401 to 6120)	2.8 (0.5 to 7.1)	1240 (660 to 2080)	1.8 (0.9 to 3)	2510 (1130 to 4450)	2.1 (0.9 to 3.6)
	Female	2290 (1200 to 3550)	2.4 (1.3 to 3.6)	1690 (803 to 2960)	1.6 (0.8 to 2.8)	1410 (550 to 2700)	1.3 (0.5 to 2.6)	1410 (569 to 2650)	1.3 (0.5 to 2.4)	308 (49 to 791)	0.3 (0 to 0.7)	723 (191 to 1380)	0.7 (0.2 to 1.3)	1010 (166 to 2980)	1 (0.2 to 3)	131 (51 to 269)	0.2 (0.1 to 0.3)	783 (277 to 1540)	0.6 (0.2 to 1.2)
Iran	Both	4730 (4310 to 5200)	6.9 (6.3 to 7.6)	1970 (1490 to 2490)	2.8 (2.1 to 3.6)	4 (1 to 9)	0 (0 to 0)	577 (342 to 862)	0.8 (0.5 to 1.2)	439 (84 to 883)	0.6 (0.1 to 1.3)	108 (32 to 169)	0.2 (0 to 0.2)	906 (211 to 1940)	1.4 (0.3 to 3)	97 (53 to 176)	0.2 (0.1 to 0.3)	397 (193 to 633)	0.5 (0.2 to 0.8)
	Male	4230 (3840 to 4680)	12.4 (11.2 to 13.6)	1360 (1020 to 1740)	3.9 (3 to 5)	2 (1 to 5)	0 (0 to 0)	359 (212 to 542)	1 (0.6 to 1.6)	303 (59 to 614)	0.9 (0.2 to 1.8)	71 (21 to 115)	0.2 (0.1 to 0.3)	610 (105 to 1400)	1.8 (0.3 to 4.2)	87 (45 to 164)	0.3 (0.1 to 0.5)	369 (176 to 591)	0.9 (0.4 to 1.5)
	Female	498 (406 to 597)	1.4 (1.2 to 1.7)	612 (450 to 776)	1.8 (1.3 to 2.3)	2 (1 to 3)	0 (0 to 0)	218 (130 to 320)	0.6 (0.4 to 0.9)	136 (26 to 276)	0.4 (0.1 to 0.8)	37 (11 to 61)	0.1 (0 to 0.2)	296 (63 to 679)	0.9 (0.2 to 2.1)	9 (5 to 16)	0 (0 to 0.1)	28 (12 to 48)	0.1 (0 to 0.1)

Iraq	Both	2760 (2120 to 3360)	13.3 (10.5 to 15.9)	1070 (709 to 1450)	5 (3.4 to 6.8)	2 (1 to 5)	0 (0 to 0)	350 (204 to 547)	1.6 (1 to 2.6)	151 (13 to 629)	0.7 (0.1 to 3)	182 (50 to 293)	0.8 (0.2 to 1.4)	526 (115 to 1180)	2.6 (0.6 to 5.8)	253 (128 to 447)	1.5 (0.8 to 2.5)	220 (98 to 388)	0.8 (0.4 to 1.5)
	Male	2470 (1880 to 3000)	25 (19.6 to 29.8)	785 (526 to 1070)	7.8 (5.3 to 10.5)	2 (1 to 4)	0 (0 to 0)	237 (136 to 375)	2.4 (1.4 to 3.7)	111 (10 to 459)	1.1 (0.1 to 4.6)	132 (34 to 216)	1.3 (0.3 to 2.1)	388 (68 to 908)	4.1 (0.7 to 9.5)	241 (119 to 434)	3.1 (1.6 to 5.3)	211 (93 to 375)	1.6 (0.7 to 2.9)
	Female	292 (214 to 385)	2.8 (2 to 3.6)	283 (187 to 400)	2.5 (1.7 to 3.5)	1 (0 to 2)	0 (0 to 0)	113 (65 to 180)	1 (0.6 to 1.5)	40 (3 to 165)	0.4 (0 to 1.4)	49 (13 to 82)	0.4 (0.1 to 0.7)	138 (30 to 329)	1.3 (0.3 to 3.1)	12 (6 to 24)	0.1 (0.1 to 0.2)	9 (3 to 16)	0.1 (0 to 0.1)
Ireland	Both	1540 (1390 to 1670)	20.1 (18.2 to 21.9)	93 (44 to 154)	1.2 (0.6 to 2)	0 (0 to 1)	0 (0 to 0)	74 (43 to 115)	1 (0.6 to 1.5)	161 (27 to 354)	2.1 (0.4 to 4.6)	82 (20 to 123)	1.1 (0.3 to 1.6)	202 (48 to 437)	2.6 (0.6 to 5.6)	386 (245 to 541)	4.9 (3.1 to 6.9)	67 (12 to 130)	0.9 (0.2 to 1.7)
	Male	886 (793 to 974)	24.9 (22.3 to 27.3)	51 (25 to 87)	1.4 (0.7 to 2.4)	0 (0 to 1)	0 (0 to 0)	43 (24 to 67)	1.2 (0.7 to 1.9)	89 (15 to 201)	2.5 (0.4 to 5.7)	45 (11 to 68)	1.3 (0.3 to 1.9)	124 (20 to 287)	3.4 (0.6 to 8)	352 (221 to 492)	9.8 (6.1 to 13.7)	46 (8 to 89)	1.2 (0.2 to 2.4)
	Female	651 (574 to 731)	16.1 (14.2 to 18.1)	41 (19 to 68)	1 (0.5 to 1.7)	0 (0 to 1)	0 (0 to 0)	31 (17 to 48)	0.8 (0.4 to 1.2)	71 (12 to 156)	1.8 (0.3 to 3.9)	37 (9 to 57)	0.9 (0.2 to 1.4)	79 (15 to 185)	1.9 (0.4 to 4.5)	34 (11 to 87)	0.8 (0.3 to 2.1)	21 (3 to 43)	0.6 (0.1 to 1.1)
Israel	Both	1670 (1530 to 1790)	14.4 (13.2 to 15.4)	353 (252 to 465)	3 (2.2 to 4)	1 (0 to 2)	0 (0 to 0)	90 (50 to 138)	0.8 (0.5 to 1.2)	107 (17 to 249)	0.9 (0.2 to 2.1)	33 (8 to 54)	0.3 (0.1 to 0.5)	242 (58 to 526)	2 (0.5 to 4.4)	281 (170 to 406)	2.3 (1.4 to 3.4)	97 (17 to 188)	0.9 (0.1 to 1.7)
	Male	1210 (1110 to 1310)	22.9 (21.1 to 24.7)	231 (165 to 303)	4.4 (3.1 to 5.8)	0 (0 to 1)	0 (0 to 0)	62 (34 to 97)	1.2 (0.7 to 1.9)	70 (11 to 164)	1.3 (0.2 to 3.1)	20 (5 to 35)	0.4 (0.1 to 0.7)	157 (27 to 364)	2.9 (0.5 to 6.8)	250 (141 to 373)	4.7 (2.6 to 7)	77 (13 to 149)	1.4 (0.2 to 2.8)
	Female	459 (398 to 512)	7.1 (6.2 to 8)	121 (87 to 162)	1.9 (1.3 to 2.5)	0 (0 to 1)	0 (0 to 0)	28 (16 to 43)	0.5 (0.3 to 0.7)	37 (6 to 85)	0.6 (0.1 to 1.3)	13 (3 to 23)	0.2 (0.1 to 0.4)	85 (16 to 199)	1.3 (0.2 to 2.9)	31 (17 to 53)	0.5 (0.2 to 0.8)	21 (3 to 42)	0.3 (0.1 to 0.7)
Italy	Both	25700 (23600 to 27300)	17.8 (16.5 to 18.9)	4190 (2970 to 5680)	2.9 (2 to 3.9)	17 (4 to 47)	0 (0 to 0)	1720 (1020 to 2570)	1.3 (0.8 to 1.9)	2460 (428 to 5150)	1.7 (0.3 to 3.5)	671 (173 to 1040)	0.5 (0.1 to 0.7)	4300 (959 to 9250)	2.8 (0.6 to 6.1)	11800 (8500 to 15000)	7.3 (5.2 to 9.3)	1130 (197 to 2180)	0.9 (0.2 to 1.7)
	Male	19700 (18100	30.4 (28.1	2990 (2110	4.6 (3.3	11 (3 to 30)	0 (0 to 0)	1310 (766	2.1 (1.3	1750 (309	2.7 (0.5	470 (117	0.7 (0.2	3250 (573	4.9 (0.9	10400 (7370	15.1 (10.7	934 (157	1.6 (0.3 to 3)

		to 20900)	to 32.2)	to 4070)	to 6.3)			to 1970)	to 3.2)	to 3630)	to 5.6)	to 758)	to 1.1)	to 7260)	to 10.9)	to 13500)	to 19.6)	to 1800)	
	Female	5990 (5240 to 6550)	7.8 (7.1 to 8.5)	1200 (817 to 1620)	1.5 (1 to 2)	6 (2 to 17)	0 (0 to 0)	419 (243 to 627)	0.6 (0.4 to 0.9)	704 (119 to 1480)	0.9 (0.2 to 1.9)	201 (51 to 326)	0.3 (0.1 to 0.4)	1050 (205 to 2400)	1.2 (0.2 to 2.8)	1310 (680 to 1930)	1.4 (0.7 to 2)	200 (30 to 396)	0.3 (0 to 0.6)
Jamaica	Both	321 (253 to 405)	10.9 (8.6 to 13.8)	60 (34 to 93)	2 (1.2 to 3.1)	11 (4 to 25)	0.4 (0.1 to 0.8)	22 (12 to 35)	0.7 (0.4 to 1.2)	16 (1 to 64)	0.5 (0 to 2.2)	19 (5 to 31)	0.6 (0.2 to 1)	75 (16 to 169)	2.5 (0.6 to 5.8)	29 (15 to 50)	1 (0.5 to 1.7)	40 (19 to 68)	1.3 (0.6 to 2.2)
	Male	282 (220 to 356)	19.8 (15.5 to 25)	46 (26 to 73)	3.3 (1.8 to 5.1)	8 (3 to 18)	0.6 (0.2 to 1.2)	16 (9 to 26)	1.1 (0.6 to 1.8)	12 (1 to 50)	0.9 (0.1 to 3.5)	15 (4 to 24)	1 (0.3 to 1.7)	57 (10 to 135)	4.1 (0.7 to 9.6)	28 (14 to 47)	2 (1 to 3.4)	34 (15 to 60)	2.3 (1 to 4)
	Female	40 (30 to 52)	2.6 (1.9 to 3.4)	13 (8 to 20)	0.8 (0.5 to 1.3)	3 (1 to 7)	0.2 (0.1 to 0.5)	5 (3 to 9)	0.3 (0.2 to 0.6)	4 (0 to 15)	0.2 (0 to 1)	5 (1 to 7)	0.3 (0.1 to 0.5)	17 (4 to 41)	1.1 (0.2 to 2.6)	2 (1 to 3)	0.1 (0.1 to 0.2)	6 (3 to 11)	0.4 (0.2 to 0.7)
Japan	Both	55800 (49000 to 60600)	14.1 (12.7 to 15.1)	8030 (4860 to 12000)	2 (1.2 to 2.9)	18 (4 to 55)	0 (0 to 0)	3040 (1730 to 4760)	0.8 (0.5 to 1.3)	1340 (253 to 2730)	0.3 (0.1 to 0.7)	3770 (1100 to 5730)	0.9 (0.3 to 1.4)	6480 (1360 to 14800)	1.5 (0.3 to 3.5)	18300 (12100 to 24800)	4 (2.6 to 5.4)	2660 (480 to 5080)	0.8 (0.2 to 1.6)
	Male	45800 (40800 to 49100)	26.6 (24.1 to 28.4)	5510 (3320 to 8210)	3.2 (2 to 4.8)	11 (2 to 33)	0 (0 to 0)	1840 (1040 to 2940)	1.1 (0.6 to 1.8)	921 (174 to 1880)	0.5 (0.1 to 1.1)	2590 (755 to 3910)	1.5 (0.5 to 2.3)	4890 (808 to 11500)	2.7 (0.5 to 6.4)	16800 (10800 to 23200)	8.9 (5.7 to 12.2)	2090 (365 to 3990)	1.4 (0.2 to 2.7)
	Female	10000 (7940 to 11700)	4.5 (3.7 to 5.1)	2520 (1480 to 3850)	1 (0.6 to 1.5)	7 (2 to 23)	0 (0 to 0)	1200 (685 to 1840)	0.6 (0.3 to 0.8)	421 (79 to 868)	0.2 (0 to 0.4)	1180 (351 to 1850)	0.5 (0.1 to 0.7)	1590 (286 to 3910)	0.6 (0.1 to 1.4)	1530 (653 to 2490)	0.5 (0.2 to 0.7)	568 (87 to 1130)	0.3 (0.1 to 0.7)
Jordan	Both	653 (516 to 807)	10.9 (8.7 to 13.5)	183 (127 to 253)	3 (2.1 to 4.2)	0 (0 to 0)	0 (0 to 0)	66 (37 to 101)	1 (0.6 to 1.6)	41 (6 to 104)	0.7 (0.1 to 1.7)	36 (8 to 58)	0.6 (0.1 to 0.9)	112 (24 to 247)	2 (0.4 to 4.4)	59 (29 to 101)	1.3 (0.7 to 2.2)	41 (18 to 71)	0.5 (0.2 to 0.9)
	Male	584 (451 to 735)	18.6 (14.6 to 23.3)	142 (95 to 203)	4.5 (3 to 6.3)	0 (0 to 0)	0 (0 to 0)	46 (25 to 73)	1.4 (0.8 to 2.2)	32 (5 to 81)	1 (0.1 to 2.6)	28 (6 to 46)	0.9 (0.2 to 1.4)	91 (17 to 208)	3.1 (0.6 to 7.1)	58 (28 to 100)	2.4 (1.2 to 4.1)	39 (17 to 70)	1 (0.4 to 1.7)
	Female	69 (52 to 89)	2.6 (2 to 3.3)	41 (28 to 57)	1.4 (1 to 2)	0 (0 to 0)	0 (0 to 0)	20 (11 to 31)	0.6 (0.4 to 1)	9 (1 to 24)	0.3 (0 to 0.8)	8 (2 to 14)	0.3 (0.1 to 0.5)	22 (5 to 50)	0.8 (0.2 to 1.9)	1 (0 to 2)	0.1 (0 to 0.1)	1 (1 to 2)	0 (0 to 0.1)

Kazakhstan	Both	2470 (2090 to 2880)	13.8 (11.8 to 16.1)	499 (301 to 730)	2.8 (1.7 to 4.1)	62 (21 to 138)	0.4 (0.1 to 0.8)	179 (104 to 275)	1 (0.6 to 1.5)	306 (28 to 1040)	1.7 (0.2 to 5.9)	163 (43 to 253)	0.9 (0.2 to 1.4)	324 (71 to 733)	1.9 (0.4 to 4.3)	229 (134 to 352)	1.4 (0.8 to 2.1)	248 (120 to 404)	1.2 (0.6 to 2)
	Male	2330 (1970 to 2730)	33.1 (28.2 to 38.3)	398 (240 to 584)	5.6 (3.4 to 8.2)	45 (15 to 100)	0.6 (0.2 to 1.4)	121 (69 to 190)	1.7 (1 to 2.7)	243 (22 to 826)	3.4 (0.3 to 11.7)	129 (35 to 204)	1.8 (0.5 to 2.9)	244 (40 to 586)	3.6 (0.6 to 8.7)	197 (107 to 306)	3.1 (1.7 to 4.8)	210 (97 to 351)	2.4 (1.1 to 4)
	Female	138 (106 to 179)	1.3 (1 to 1.7)	101 (62 to 150)	1 (0.6 to 1.5)	17 (6 to 39)	0.2 (0.1 to 0.4)	58 (33 to 91)	0.6 (0.3 to 0.9)	63 (6 to 215)	0.6 (0.1 to 2.1)	35 (9 to 55)	0.3 (0.1 to 0.5)	80 (16 to 181)	0.8 (0.2 to 1.8)	33 (10 to 79)	0.3 (0.1 to 0.8)	38 (18 to 65)	0.3 (0.2 to 0.6)
Kenya	Both	478 (389 to 588)	2.5 (2 to 3)	92 (49 to 148)	0.5 (0.2 to 0.7)	284 (191 to 404)	1.4 (1 to 2)	25 (12 to 42)	0.1 (0.1 to 0.2)	52 (10 to 111)	0.3 (0.1 to 0.5)	62 (20 to 99)	0.3 (0.1 to 0.5)	73 (15 to 171)	0.4 (0.1 to 0.9)	67 (26 to 223)	0.4 (0.2 to 1.3)	91 (45 to 148)	0.4 (0.2 to 0.6)
	Male	447 (362 to 551)	5.2 (4.2 to 6.2)	72 (38 to 115)	0.8 (0.4 to 1.3)	207 (132 to 293)	2.3 (1.5 to 3.2)	17 (8 to 28)	0.2 (0.1 to 0.3)	39 (7 to 84)	0.4 (0.1 to 0.9)	46 (15 to 74)	0.5 (0.2 to 0.8)	61 (10 to 149)	0.7 (0.1 to 1.8)	55 (18 to 207)	0.8 (0.2 to 2.8)	71 (33 to 118)	0.6 (0.3 to 1)
	Female	31 (20 to 48)	0.3 (0.2 to 0.5)	20 (9 to 37)	0.2 (0.1 to 0.3)	78 (44 to 131)	0.7 (0.4 to 1.2)	8 (4 to 16)	0.1 (0 to 0.1)	13 (2 to 30)	0.1 (0 to 0.3)	16 (4 to 30)	0.1 (0 to 0.3)	13 (2 to 34)	0.1 (0 to 0.3)	12 (3 to 26)	0.1 (0 to 0.3)	19 (8 to 38)	0.1 (0.1 to 0.3)
Kiribati	Both	9 (7 to 12)	14.7 (11.1 to 18.8)	0 (0 to 1)	0.6 (0.2 to 1.6)	3 (2 to 4)	4.2 (2.5 to 6.1)	1 (0 to 2)	1.4 (0.7 to 2.3)	0 (0 to 2)	0.6 (0 to 2.5)	1 (0 to 1)	0.9 (0.2 to 1.5)	2 (0 to 5)	3.2 (0.7 to 7)	0 (0 to 0)	0.5 (0.3 to 0.9)	0 (0 to 1)	0.5 (0.2 to 1)
	Male	8 (6 to 11)	29.9 (22.2 to 38.5)	0 (0 to 1)	1.3 (0.4 to 3.2)	2 (1 to 3)	7.9 (4.7 to 11.8)	1 (0 to 1)	2.5 (1.3 to 4.2)	0 (0 to 1)	1.2 (0.1 to 5)	0 (0 to 1)	1.7 (0.5 to 3)	2 (0 to 4)	6.6 (1.3 to 14.9)	0 (0 to 0)	1.2 (0.7 to 2.1)	0 (0 to 1)	1 (0.4 to 1.9)
	Female	2 (1 to 2)	4.4 (3.2 to 5.9)	0 (0 to 0)	0.2 (0 to 0.4)	1 (0 to 1)	1.6 (1 to 2.4)	0 (0 to 0)	0.6 (0.3 to 1)	0 (0 to 0)	0.2 (0 to 0.9)	0 (0 to 0)	0.3 (0.1 to 0.6)	0 (0 to 1)	0.9 (0.2 to 2.2)	0 (0 to 0)	0.1 (0.1 to 0.2)	0 (0 to 0)	0.2 (0.1 to 0.3)
Kuwait	Both	143 (112 to 176)	7 (5.5 to 8.7)	65 (46 to 87)	3.1 (2.2 to 4.1)	0 (0 to 0)	0 (0 to 0)	18 (11 to 27)	0.8 (0.5 to 1.2)	5 (1 to 13)	0.2 (0 to 0.6)	8 (2 to 13)	0.4 (0.1 to 0.6)	36 (8 to 77)	1.9 (0.4 to 4)	22 (12 to 35)	1.3 (0.7 to 2.1)	14 (6 to 23)	0.5 (0.2 to 0.8)
	Male	134 (105 to 169)	11 (8.5	52 (36 to 71)	4.2 (2.9	0 (0 to 0)	0 (0 to 0)	13 (7 to 20)	1 (0.6 to 1.6)	4 (1 to 10)	0.3 (0 to 0.8)	6 (1 to 10)	0.5 (0.1	30 (5 to 66)	2.6 (0.5	21 (11 to 35)	2.1 (1.1	12 (5 to 22)	0.7 (0.3

			to 13.7)		to 5.7)								to 0.8)		to 5.7)		to 3.4)		to 1.3)
	Female	9 (6 to 11)	1 (0.7 to 1.4)	13 (10 to 18)	1.4 (1 to 1.9)	0 (0 to 0)	0 (0 to 0)	5 (3 to 8)	0.5 (0.3 to 0.8)	1 (0 to 3)	0.1 (0 to 0.3)	2 (0 to 3)	0.2 (0 to 0.3)	6 (1 to 13)	0.8 (0.2 to 1.7)	0 (0 to 0)	0 (0 to 0.1)	1 (1 to 2)	0.1 (0 to 0.2)
Kyrgyzstan	Both	366 (316 to 417)	8.1 (7 to 9.1)	74 (44 to 114)	1.6 (1 to 2.5)	37 (19 to 63)	0.8 (0.4 to 1.4)	40 (25 to 60)	0.9 (0.5 to 1.3)	44 (3 to 144)	1 (0.1 to 3.2)	28 (9 to 44)	0.6 (0.2 to 1)	28 (6 to 66)	0.7 (0.1 to 1.5)	16 (9 to 25)	0.4 (0.2 to 0.6)	39 (18 to 63)	0.7 (0.3 to 1.2)
	Male	338 (292 to 386)	17.6 (15.3 to 20)	57 (33 to 88)	2.9 (1.7 to 4.5)	26 (12 to 45)	1.3 (0.6 to 2.3)	24 (15 to 37)	1.3 (0.8 to 2)	33 (2 to 109)	1.7 (0.1 to 5.7)	21 (7 to 33)	1.1 (0.3 to 1.7)	21 (3 to 52)	1.2 (0.2 to 2.8)	15 (8 to 24)	0.9 (0.5 to 1.4)	33 (15 to 55)	1.4 (0.6 to 2.3)
	Female	28 (21 to 35)	1.1 (0.9 to 1.4)	17 (9 to 26)	0.7 (0.4 to 1)	12 (6 to 19)	0.5 (0.2 to 0.7)	16 (10 to 23)	0.6 (0.4 to 0.9)	11 (1 to 36)	0.4 (0 to 1.4)	7 (2 to 11)	0.3 (0.1 to 0.4)	7 (1 to 18)	0.3 (0.1 to 0.7)	1 (0 to 2)	0 (0 to 0.1)	5 (2 to 9)	0.2 (0.1 to 0.3)
Laos	Both	612 (448 to 782)	15.5 (11.6 to 19.4)	63 (30 to 109)	1.5 (0.7 to 2.6)	260 (157 to 378)	6.3 (3.9 to 9.1)	52 (27 to 85)	1.3 (0.7 to 2.1)	22 (2 to 79)	0.5 (0 to 1.9)	39 (10 to 65)	1 (0.2 to 1.6)	97 (21 to 224)	2.6 (0.6 to 6)	23 (11 to 41)	0.7 (0.3 to 1.2)	70 (30 to 126)	1.4 (0.6 to 2.5)
	Male	558 (411 to 717)	29.8 (22.3 to 37.7)	50 (24 to 87)	2.6 (1.2 to 4.5)	185 (109 to 274)	9.6 (5.7 to 14)	28 (14 to 47)	1.5 (0.8 to 2.5)	16 (1 to 59)	0.8 (0.1 to 3)	29 (7 to 48)	1.5 (0.4 to 2.5)	72 (12 to 178)	4.1 (0.7 to 10)	21 (9 to 38)	1.4 (0.6 to 2.5)	49 (20 to 88)	2 (0.8 to 3.6)
	Female	54 (38 to 80)	2.7 (1.9 to 4)	13 (6 to 24)	0.6 (0.3 to 1.1)	75 (47 to 113)	3.4 (2.2 to 5.2)	23 (12 to 40)	1 (0.5 to 1.8)	6 (0 to 21)	0.3 (0 to 0.9)	11 (3 to 18)	0.5 (0.1 to 0.8)	26 (5 to 63)	1.3 (0.3 to 3.2)	2 (1 to 4)	0.1 (0 to 0.2)	21 (8 to 41)	0.8 (0.3 to 1.6)
Latvia	Both	668 (543 to 826)	17.4 (14 to 21.6)	78 (48 to 119)	2 (1.2 to 3.1)	6 (1 to 17)	0.2 (0 to 0.4)	38 (21 to 60)	1 (0.5 to 1.6)	78 (9 to 189)	2 (0.2 to 4.9)	49 (15 to 81)	1.3 (0.4 to 2.1)	64 (13 to 148)	1.6 (0.3 to 3.7)	63 (34 to 106)	1.5 (0.8 to 2.5)	23 (4 to 46)	0.7 (0.1 to 1.4)
	Male	577 (454 to 734)	39.5 (31.1 to 50.2)	59 (36 to 93)	4.1 (2.4 to 6.4)	4 (1 to 12)	0.3 (0.1 to 0.8)	24 (13 to 40)	1.7 (0.9 to 2.8)	59 (7 to 146)	4.1 (0.5 to 10.1)	37 (11 to 63)	2.6 (0.8 to 4.4)	49 (8 to 121)	3.3 (0.5 to 8.2)	58 (29 to 102)	3.9 (1.9 to 6.8)	18 (3 to 36)	1.2 (0.2 to 2.5)
	Female	91 (66 to 122)	3.9 (2.8 to 5.2)	19 (11 to 30)	0.8 (0.5 to 1.2)	2 (0 to 5)	0.1 (0 to 0.2)	14 (7 to 22)	0.6 (0.3 to 1)	19 (2 to 47)	0.8 (0.1 to 1.9)	12 (4 to 21)	0.5 (0.1 to 0.8)	15 (3 to 37)	0.6 (0.1 to 1.4)	5 (2 to 9)	0.2 (0.1 to 0.3)	5 (1 to 11)	0.3 (0 to 0.6)

Lebanon	Both	1110 (909 to 1470)	21.3 (17.5 to 28.2)	276 (184 to 397)	5.3 (3.5 to 7.6)	1 (0 to 2)	0 (0 to 0)	131 (78 to 204)	2.5 (1.5 to 3.9)	63 (5 to 249)	1.2 (0.1 to 4.8)	17 (4 to 30)	0.3 (0.1 to 0.6)	190 (45 to 418)	3.6 (0.9 to 8)	10 (2 to 42)	0.2 (0 to 0.8)	66 (30 to 118)	1.3 (0.6 to 2.3)
	Male	798 (640 to 1070)	34 (27.3 to 45.5)	185 (121 to 266)	7.9 (5.2 to 11.3)	0 (0 to 1)	0 (0 to 0)	86 (51 to 134)	3.7 (2.2 to 5.7)	43 (3 to 164)	1.8 (0.1 to 7)	11 (3 to 20)	0.4 (0.1 to 0.8)	135 (25 to 310)	5.8 (1.1 to 13.2)	7 (0 to 37)	0.3 (0 to 1.6)	60 (26 to 109)	2.5 (1.1 to 4.6)
	Female	309 (221 to 435)	10.8 (7.8 to 15.2)	91 (58 to 135)	3.2 (2 to 4.7)	0 (0 to 1)	0 (0 to 0)	45 (25 to 72)	1.6 (0.9 to 2.5)	21 (2 to 82)	0.7 (0.1 to 2.9)	6 (2 to 12)	0.2 (0.1 to 0.4)	55 (11 to 130)	1.9 (0.4 to 4.5)	3 (1 to 8)	0.1 (0 to 0.3)	6 (2 to 11)	0.2 (0.1 to 0.4)
Lesotho	Both	135 (91 to 191)	10.9 (7.6 to 15.2)	24 (12 to 42)	1.9 (0.9 to 3.3)	41 (22 to 66)	3.3 (1.8 to 5.3)	11 (5 to 20)	0.9 (0.4 to 1.5)	12 (1 to 48)	1 (0.1 to 3.8)	15 (5 to 26)	1.2 (0.4 to 2.1)	19 (4 to 46)	1.6 (0.3 to 3.8)	67 (31 to 111)	5.5 (2.6 to 9)	10 (4 to 20)	0.7 (0.3 to 1.3)
	Male	123 (84 to 176)	24.8 (17.4 to 34.8)	19 (9 to 33)	3.8 (1.8 to 6.4)	29 (16 to 48)	5.8 (3.2 to 9.7)	7 (3 to 12)	1.4 (0.7 to 2.4)	9 (1 to 36)	1.8 (0.1 to 7)	11 (4 to 19)	2.2 (0.8 to 3.8)	14 (2 to 35)	3 (0.5 to 7.5)	64 (28 to 107)	14 (6.6 to 22.8)	9 (3 to 17)	1.4 (0.5 to 2.7)
	Female	12 (7 to 19)	2 (1.2 to 3)	5 (2 to 10)	0.7 (0.3 to 1.4)	12 (6 to 20)	1.7 (0.9 to 2.9)	4 (2 to 8)	0.6 (0.3 to 1.1)	3 (0 to 12)	0.5 (0 to 1.7)	4 (1 to 7)	0.5 (0.2 to 1)	5 (1 to 14)	0.8 (0.1 to 2)	3 (1 to 8)	0.5 (0.2 to 1.2)	1 (0 to 3)	0.2 (0.1 to 0.4)
Liberia	Both	54 (35 to 76)	3 (2 to 4.3)	14 (6 to 27)	0.8 (0.3 to 1.5)	50 (30 to 77)	2.8 (1.6 to 4.2)	3 (1 to 6)	0.2 (0.1 to 0.3)	8 (1 to 30)	0.5 (0 to 1.6)	10 (3 to 16)	0.5 (0.2 to 0.9)	16 (3 to 37)	1 (0.2 to 2.3)	3 (1 to 9)	0.2 (0.1 to 0.5)	9 (4 to 17)	0.4 (0.2 to 0.7)
	Male	50 (33 to 72)	5.6 (3.7 to 8)	11 (4 to 21)	1.2 (0.5 to 2.3)	35 (20 to 56)	3.9 (2.2 to 6.1)	2 (1 to 4)	0.2 (0.1 to 0.4)	6 (0 to 22)	0.7 (0.1 to 2.4)	7 (2 to 12)	0.8 (0.2 to 1.3)	12 (2 to 29)	1.5 (0.2 to 3.5)	3 (1 to 8)	0.4 (0.1 to 1)	7 (2 to 13)	0.5 (0.2 to 1.1)
	Female	4 (2 to 5)	0.4 (0.3 to 0.6)	3 (1 to 6)	0.3 (0.1 to 0.7)	15 (9 to 23)	1.6 (1 to 2.5)	1 (1 to 2)	0.1 (0.1 to 0.2)	2 (0 to 8)	0.3 (0 to 0.9)	3 (1 to 5)	0.3 (0.1 to 0.5)	4 (1 to 10)	0.5 (0.1 to 1.2)	1 (0 to 1)	0.1 (0 to 0.2)	2 (1 to 4)	0.2 (0.1 to 0.4)
Libya	Both	644 (467 to 823)	13.5 (9.8 to 17.2)	215 (130 to 311)	4.4 (2.7 to 6.4)	0 (0 to 1)	0 (0 to 0)	84 (49 to 131)	1.8 (1 to 2.7)	35 (3 to 124)	0.7 (0.1 to 2.6)	28 (7 to 47)	0.6 (0.1 to 0.9)	130 (26 to 294)	2.9 (0.6 to 6.5)	14 (3 to 62)	0.3 (0.1 to 1.5)	50 (22 to 92)	0.9 (0.4 to 1.6)
	Male	638 (462 to 814)	26.2 (19 to 33.5)	187 (112	7.6 (4.5	0 (0 to 1)	0 (0 to 0)	70 (40 to 109)	2.9 (1.7	31 (2 to 108)	1.2 (0.1	24 (6 to 40)	1 (0.2 to 1.6)	114 (20 to 266)	5 (0.9 to 11.5)	13 (3 to 62)	0.6 (0.1	48 (21 to 88)	1.6 (0.7 to 3)

				to 269)	to 10.9)				to 4.6)		to 4.4)						to 2.9)		
	Female	6 (3 to 9)	0.3 (0.2 to 0.4)	28 (16 to 43)	1.2 (0.6 to 1.8)	0 (0 to 0)	0 (0 to 0)	15 (8 to 23)	0.6 (0.3 to 0.9)	5 (0 to 16)	0.2 (0 to 0.7)	4 (1 to 7)	0.2 (0 to 0.3)	16 (3 to 38)	0.7 (0.2 to 1.7)	1 (0 to 2)	0 (0 to 0.1)	2 (1 to 4)	0.1 (0 to 0.1)
Lithuania	Both	876 (705 to 1060)	15.8 (12.7 to 19.3)	89 (52 to 138)	1.6 (0.9 to 2.5)	4 (1 to 12)	0.1 (0 to 0.2)	47 (26 to 76)	0.9 (0.5 to 1.4)	59 (7 to 154)	1.1 (0.1 to 2.8)	60 (17 to 98)	1.1 (0.3 to 1.8)	74 (15 to 176)	1.3 (0.2 to 3.1)	67 (37 to 107)	1.1 (0.6 to 1.8)	34 (6 to 67)	0.7 (0.1 to 1.3)
	Male	783 (627 to 949)	36.7 (29.4 to 44.5)	69 (39 to 108)	3.3 (1.9 to 5.1)	3 (1 to 8)	0.1 (0 to 0.4)	32 (18 to 53)	1.5 (0.8 to 2.5)	46 (6 to 119)	2.1 (0.3 to 5.6)	47 (13 to 77)	2.2 (0.6 to 3.6)	60 (9 to 148)	2.8 (0.4 to 6.9)	59 (30 to 99)	2.7 (1.4 to 4.6)	26 (4 to 53)	1.2 (0.2 to 2.5)
	Female	93 (73 to 117)	2.9 (2.2 to 3.7)	20 (11 to 31)	0.6 (0.3 to 0.9)	1 (0 to 4)	0 (0 to 0.1)	15 (8 to 24)	0.4 (0.2 to 0.7)	13 (2 to 34)	0.4 (0 to 1)	14 (4 to 22)	0.4 (0.1 to 0.6)	14 (3 to 36)	0.4 (0.1 to 0.9)	8 (4 to 14)	0.2 (0.1 to 0.3)	7 (1 to 15)	0.2 (0 to 0.5)
Luxembourg	Both	203 (176 to 233)	20.4 (17.7 to 23.4)	19 (11 to 29)	1.9 (1.1 to 2.9)	0 (0 to 0)	0 (0 to 0)	11 (7 to 18)	1.2 (0.7 to 1.8)	25 (4 to 52)	2.5 (0.4 to 5.2)	11 (3 to 17)	1.1 (0.3 to 1.7)	37 (9 to 81)	3.6 (0.8 to 8)	71 (48 to 97)	6.9 (4.6 to 9.5)	8 (1 to 16)	0.8 (0.1 to 1.6)
	Male	146 (126 to 167)	31.6 (27.4 to 36.2)	13 (7 to 20)	2.8 (1.6 to 4.2)	0 (0 to 0)	0 (0 to 0)	7 (4 to 11)	1.6 (0.9 to 2.5)	17 (3 to 35)	3.8 (0.6 to 7.7)	7 (2 to 11)	1.6 (0.4 to 2.5)	27 (5 to 60)	5.8 (1 to 13.1)	68 (45 to 93)	14.8 (9.8 to 20.4)	6 (1 to 12)	1.3 (0.2 to 2.6)
	Female	57 (46 to 69)	10.8 (8.8 to 13)	6 (3 to 9)	1.1 (0.6 to 1.7)	0 (0 to 0)	0 (0 to 0)	4 (2 to 7)	0.8 (0.5 to 1.3)	8 (1 to 17)	1.5 (0.2 to 3.2)	4 (1 to 6)	0.7 (0.2 to 1.1)	10 (2 to 24)	1.8 (0.4 to 4.2)	3 (2 to 6)	0.6 (0.3 to 1)	2 (0 to 3)	0.3 (0.1 to 0.7)
Madagascar	Both	182 (128 to 250)	1.9 (1.4 to 2.5)	25 (10 to 49)	0.3 (0.1 to 0.5)	213 (136 to 319)	2.1 (1.4 to 3.1)	14 (6 to 26)	0.1 (0.1 to 0.2)	29 (2 to 115)	0.3 (0 to 1.1)	36 (13 to 62)	0.4 (0.1 to 0.6)	35 (7 to 86)	0.4 (0.1 to 1)	17 (4 to 93)	0.2 (0.1 to 1.2)	44 (18 to 81)	0.3 (0.1 to 0.6)
	Male	171 (119 to 234)	3.7 (2.7 to 5)	19 (7 to 37)	0.4 (0.2 to 0.8)	143 (88 to 216)	3 (1.9 to 4.5)	8 (3 to 16)	0.2 (0.1 to 0.3)	20 (1 to 77)	0.4 (0 to 1.6)	25 (8 to 43)	0.5 (0.2 to 0.9)	25 (4 to 67)	0.6 (0.1 to 1.7)	11 (1 to 85)	0.3 (0 to 2.3)	28 (11 to 54)	0.4 (0.2 to 0.8)
	Female	11 (7 to 17)	0.2 (0.1 to 0.3)	6 (2 to 12)	0.1 (0 to 0.2)	71 (43 to 104)	1.3 (0.8 to 1.9)	5 (2 to 10)	0.1 (0 to 0.2)	9 (1 to 35)	0.2 (0 to 0.6)	12 (4 to 20)	0.2 (0.1 to 0.4)	9 (2 to 24)	0.2 (0 to 0.5)	6 (1 to 13)	0.1 (0 to 0.3)	16 (6 to 30)	0.2 (0.1 to 0.4)

Malawi	Both	169 (129 to 218)	2.6 (2 to 3.3)	16 (7 to 33)	0.2 (0.1 to 0.5)	135 (91 to 192)	2 (1.4 to 2.8)	8 (4 to 14)	0.1 (0.1 to 0.2)	16 (1 to 58)	0.2 (0 to 0.8)	20 (6 to 34)	0.3 (0.1 to 0.5)	32 (7 to 72)	0.5 (0.1 to 1.1)	18 (6 to 75)	0.3 (0.1 to 1.2)	24 (11 to 42)	0.3 (0.1 to 0.5)
	Male	154 (116 to 200)	5.8 (4.5 to 7.5)	13 (5 to 26)	0.4 (0.2 to 0.9)	93 (61 to 136)	3.2 (2.2 to 4.7)	5 (2 to 9)	0.2 (0.1 to 0.3)	11 (1 to 43)	0.4 (0 to 1.5)	14 (4 to 25)	0.5 (0.1 to 0.8)	24 (4 to 58)	0.9 (0.1 to 2.2)	14 (3 to 68)	0.6 (0.2 to 2.8)	17 (7 to 32)	0.5 (0.2 to 0.9)
	Female	15 (9 to 21)	0.4 (0.2 to 0.6)	4 (1 to 8)	0.1 (0 to 0.2)	42 (28 to 61)	1.1 (0.7 to 1.6)	3 (1 to 5)	0.1 (0 to 0.1)	5 (0 to 17)	0.1 (0 to 0.5)	6 (2 to 10)	0.2 (0.1 to 0.3)	7 (1 to 18)	0.2 (0 to 0.5)	5 (1 to 9)	0.1 (0 to 0.3)	7 (3 to 13)	0.2 (0.1 to 0.3)
Malaysia	Both	2900 (2210 to 3680)	11.6 (8.9 to 14.6)	614 (371 to 905)	2.4 (1.5 to 3.6)	6 (2 to 15)	0 (0 to 0.1)	300 (164 to 483)	1.2 (0.6 to 1.9)	77 (9 to 221)	0.3 (0 to 0.9)	190 (45 to 314)	0.8 (0.2 to 1.2)	568 (124 to 1320)	2.4 (0.5 to 5.5)	140 (68 to 240)	0.7 (0.3 to 1.1)	344 (155 to 596)	1.1 (0.5 to 2)
	Male	2710 (2070 to 3450)	21.8 (16.7 to 27.7)	434 (260 to 645)	3.4 (2.1 to 5.1)	4 (1 to 9)	0 (0 to 0.1)	170 (92 to 282)	1.4 (0.7 to 2.2)	54 (7 to 158)	0.4 (0.1 to 1.2)	132 (30 to 222)	1.1 (0.2 to 1.8)	399 (68 to 984)	3.4 (0.6 to 8.2)	127 (58 to 222)	1.3 (0.6 to 2.2)	273 (117 to 485)	1.8 (0.8 to 3.2)
	Female	188 (134 to 259)	1.6 (1.2 to 2.2)	180 (108 to 269)	1.4 (0.9 to 2.1)	2 (1 to 6)	0 (0 to 0)	129 (70 to 210)	1 (0.5 to 1.6)	23 (3 to 63)	0.2 (0 to 0.5)	58 (15 to 94)	0.5 (0.1 to 0.8)	169 (34 to 406)	1.4 (0.3 to 3.4)	12 (4 to 24)	0.1 (0 to 0.2)	71 (30 to 131)	0.5 (0.2 to 0.9)
Maldives	Both	17 (14 to 21)	6.8 (5.5 to 8.1)	2 (1 to 3)	0.7 (0.4 to 1.1)	1 (0 to 2)	0.3 (0.1 to 0.6)	2 (1 to 3)	0.6 (0.3 to 1)	1 (0 to 4)	0.4 (0 to 1.6)	1 (0 to 2)	0.5 (0.1 to 0.8)	3 (1 to 6)	1.1 (0.2 to 2.4)	3 (2 to 4)	1.2 (0.7 to 1.8)	1 (1 to 2)	0.4 (0.2 to 0.7)
	Male	16 (13 to 19)	11.7 (9.5 to 14.2)	1 (1 to 2)	1 (0.6 to 1.6)	1 (0 to 1)	0.4 (0.2 to 0.8)	1 (1 to 2)	0.8 (0.4 to 1.2)	1 (0 to 3)	0.6 (0.1 to 2.3)	1 (0 to 2)	0.7 (0.2 to 1.1)	2 (0 to 5)	1.6 (0.3 to 3.7)	3 (1 to 4)	2.1 (1.2 to 3.4)	1 (1 to 2)	0.6 (0.3 to 1.1)
	Female	1 (1 to 2)	1.1 (0.8 to 1.5)	0 (0 to 1)	0.3 (0.2 to 0.5)	0 (0 to 0)	0.2 (0.1 to 0.4)	1 (0 to 1)	0.4 (0.3 to 0.7)	0 (0 to 1)	0.2 (0 to 0.8)	0 (0 to 0)	0.2 (0.1 to 0.4)	1 (0 to 1)	0.5 (0.1 to 1.1)	0 (0 to 0)	0 (0 to 0.1)	0 (0 to 0)	0.1 (0.1 to 0.3)
Mali	Both	195 (139 to 259)	2.5 (1.8 to 3.3)	34 (10 to 74)	0.4 (0.1 to 0.9)	199 (119 to 320)	2.5 (1.5 to 4)	15 (7 to 25)	0.2 (0.1 to 0.3)	26 (2 to 100)	0.3 (0 to 1.2)	33 (11 to 56)	0.4 (0.1 to 0.7)	40 (8 to 94)	0.5 (0.1 to 1.3)	9 (3 to 26)	0.1 (0 to 0.4)	24 (10 to 42)	0.2 (0.1 to 0.4)
	Male	184 (129 to 246)	4.6 (3.3	26 (7 to 60)	0.6 (0.2	137 (77 to 222)	3.3 (1.9	9 (4 to 16)	0.2 (0.1	18 (1 to 70)	0.4 (0 to 1.7)	23 (7 to 41)	0.6 (0.2 to 1)	27 (4 to 67)	0.7 (0.1	7 (2 to 24)	0.2 (0.1	17 (7 to 33)	0.3 (0.1

			to 6.1)		to 1.4)		to 5.3)		to 0.4)						to 1.8)		to 0.6)		to 0.7)
	Female	11 (7 to 16)	0.3 (0.2 to 0.4)	8 (2 to 17)	0.2 (0.1 to 0.4)	62 (37 to 103)	1.6 (0.9 to 2.6)	5 (2 to 9)	0.1 (0.1 to 0.2)	8 (1 to 29)	0.2 (0 to 0.7)	10 (3 to 16)	0.2 (0.1 to 0.4)	13 (2 to 31)	0.4 (0.1 to 0.9)	2 (1 to 5)	0.1 (0 to 0.2)	6 (2 to 12)	0.1 (0.1 to 0.2)
Malta	Both	138 (122 to 155)	14.7 (13 to 16.5)	18 (11 to 25)	1.9 (1.2 to 2.7)	0 (0 to 0)	0 (0 to 0)	8 (5 to 12)	0.9 (0.5 to 1.4)	7 (0 to 24)	0.8 (0 to 2.5)	7 (2 to 11)	0.8 (0.2 to 1.2)	23 (5 to 50)	2.3 (0.5 to 5.1)	61 (42 to 81)	6 (4.1 to 8)	7 (1 to 14)	0.8 (0.1 to 1.6)
	Male	117 (104 to 131)	26.7 (23.6 to 29.7)	14 (9 to 21)	3.3 (2.1 to 4.7)	0 (0 to 0)	0 (0 to 0)	6 (4 to 10)	1.5 (0.9 to 2.4)	6 (0 to 19)	1.3 (0.1 to 4.4)	6 (1 to 9)	1.4 (0.3 to 2.1)	19 (3 to 42)	4.1 (0.7 to 9.3)	60 (41 to 79)	13.2 (8.9 to 17.5)	6 (1 to 13)	1.5 (0.2 to 2.9)
	Female	21 (17 to 25)	4.4 (3.7 to 5.2)	3 (2 to 5)	0.7 (0.4 to 1)	0 (0 to 0)	0 (0 to 0)	2 (1 to 2)	0.4 (0.2 to 0.6)	1 (0 to 5)	0.3 (0 to 0.9)	1 (0 to 2)	0.3 (0.1 to 0.5)	4 (1 to 10)	0.8 (0.2 to 1.9)	2 (1 to 3)	0.3 (0.2 to 0.5)	1 (0 to 2)	0.2 (0 to 0.4)
Marshall Islands	Both	5 (3 to 8)	16.9 (10.3 to 26.1)	0 (0 to 1)	1.5 (0.4 to 3.5)	1 (0 to 2)	2.8 (1.3 to 5.2)	1 (0 to 1)	1.7 (0.8 to 3.1)	0 (0 to 1)	1.2 (0.1 to 4.7)	0 (0 to 1)	1.6 (0.5 to 3)	2 (0 to 5)	6.6 (1.5 to 15.9)	1 (0 to 2)	3.2 (1.4 to 6.2)	0 (0 to 1)	0.9 (0.3 to 1.8)
	Male	4 (2 to 7)	28.6 (16.8 to 45)	0 (0 to 1)	2.3 (0.6 to 5.4)	1 (0 to 1)	3.8 (1.5 to 7.8)	0 (0 to 1)	2.2 (1 to 4.2)	0 (0 to 1)	1.7 (0.1 to 6.8)	0 (0 to 1)	2.3 (0.7 to 4.6)	1 (0 to 4)	9.4 (1.7 to 24.4)	1 (0 to 2)	5.9 (2.5 to 11.5)	0 (0 to 1)	1.5 (0.5 to 3.1)
	Female	1 (0 to 1)	4.3 (2.6 to 6.3)	0 (0 to 0)	0.7 (0.2 to 1.7)	0 (0 to 0)	1.7 (0.9 to 3)	0 (0 to 0)	1.1 (0.6 to 2)	0 (0 to 0)	0.6 (0 to 2.5)	0 (0 to 0)	0.8 (0.3 to 1.5)	1 (0 to 1)	3.4 (0.8 to 7.8)	0 (0 to 0)	0.3 (0.1 to 0.7)	0 (0 to 0)	0.3 (0.1 to 0.6)
Mauritania	Both	83 (54 to 123)	4.2 (2.8 to 6.2)	41 (20 to 68)	2.1 (1.1 to 3.5)	31 (16 to 51)	1.6 (0.8 to 2.6)	6 (3 to 10)	0.3 (0.1 to 0.5)	9 (1 to 34)	0.5 (0 to 1.7)	14 (5 to 25)	0.7 (0.3 to 1.3)	12 (3 to 28)	0.7 (0.1 to 1.5)	4 (2 to 10)	0.2 (0.1 to 0.6)	6 (2 to 12)	0.3 (0.1 to 0.5)
	Male	75 (49 to 115)	7.6 (5.1 to 11.5)	29 (14 to 52)	2.9 (1.4 to 5.2)	19 (9 to 35)	1.9 (0.9 to 3.5)	3 (1 to 7)	0.3 (0.1 to 0.6)	6 (0 to 24)	0.6 (0 to 2.4)	10 (3 to 18)	1 (0.3 to 1.8)	7 (1 to 19)	0.8 (0.1 to 2)	4 (1 to 9)	0.4 (0.1 to 1)	5 (2 to 10)	0.4 (0.2 to 0.9)
	Female	7 (4 to 11)	0.7 (0.4 to 1.1)	12 (6 to 21)	1.3 (0.6 to 2.2)	12 (6 to 20)	1.2 (0.6 to 2)	2 (1 to 4)	0.2 (0.1 to 0.4)	3 (0 to 12)	0.3 (0 to 1.2)	5 (2 to 8)	0.5 (0.2 to 0.9)	5 (1 to 11)	0.5 (0.1 to 1.2)	1 (0 to 2)	0.1 (0 to 0.2)	1 (0 to 2)	0.1 (0 to 0.2)

Mauritius	Both	108 (87 to 131)	6.2 (5.1 to 7.5)	22 (10 to 36)	1.3 (0.6 to 2.1)	0 (0 to 1)	0 (0 to 0.1)	11 (6 to 18)	0.7 (0.4 to 1.1)	5 (0 to 19)	0.3 (0 to 1.1)	12 (4 to 19)	0.7 (0.2 to 1.1)	35 (8 to 74)	2.1 (0.5 to 4.4)	3 (2 to 5)	0.2 (0.1 to 0.3)	13 (6 to 21)	0.6 (0.3 to 1.1)
	Male	100 (80 to 121)	13.3 (10.8 to 16.1)	15 (7 to 26)	2 (0.9 to 3.4)	0 (0 to 1)	0 (0 to 0.1)	7 (3 to 11)	0.9 (0.5 to 1.4)	4 (0 to 13)	0.5 (0 to 1.7)	8 (3 to 13)	1.1 (0.4 to 1.7)	25 (5 to 55)	3.4 (0.6 to 7.5)	3 (1 to 5)	0.5 (0.2 to 0.8)	10 (5 to 17)	1.1 (0.5 to 1.9)
	Female	8 (6 to 11)	0.9 (0.6 to 1.1)	6 (3 to 11)	0.7 (0.3 to 1.1)	0 (0 to 0)	0 (0 to 0)	5 (3 to 8)	0.5 (0.3 to 0.8)	2 (0 to 6)	0.2 (0 to 0.6)	3 (1 to 6)	0.4 (0.1 to 0.6)	10 (2 to 22)	1 (0.2 to 2.3)	0 (0 to 0)	0 (0 to 0)	2 (1 to 4)	0.2 (0.1 to 0.4)
Mexico	Both	4700 (3900 to 5570)	4.2 (3.5 to 5)	1400 (965 to 1930)	1.2 (0.9 to 1.7)	331 (173 to 561)	0.3 (0.2 to 0.5)	207 (83 to 373)	0.2 (0.1 to 0.3)	591 (110 to 1240)	0.5 (0.1 to 1.1)	305 (83 to 476)	0.3 (0.1 to 0.4)	1580 (389 to 3380)	1.4 (0.4 to 3)	864 (550 to 1240)	0.8 (0.5 to 1.1)	794 (385 to 1260)	0.6 (0.3 to 1)
	Male	3860 (3140 to 4690)	7.6 (6.2 to 9.2)	920 (615 to 1300)	1.8 (1.2 to 2.5)	198 (98 to 359)	0.4 (0.2 to 0.7)	131 (50 to 245)	0.3 (0.1 to 0.5)	382 (70 to 806)	0.7 (0.1 to 1.5)	195 (52 to 318)	0.4 (0.1 to 0.6)	1060 (193 to 2370)	2.1 (0.4 to 4.6)	740 (424 to 1120)	1.5 (0.9 to 2.2)	630 (285 to 1030)	1.1 (0.5 to 1.8)
	Female	845 (660 to 1040)	1.4 (1.1 to 1.8)	481 (321 to 679)	0.8 (0.5 to 1.1)	133 (68 to 221)	0.2 (0.1 to 0.4)	77 (30 to 138)	0.1 (0 to 0.2)	208 (40 to 437)	0.3 (0.1 to 0.7)	110 (31 to 177)	0.2 (0 to 0.3)	525 (107 to 1190)	0.9 (0.2 to 2)	124 (62 to 191)	0.2 (0.1 to 0.3)	163 (75 to 272)	0.2 (0.1 to 0.4)
Federatead States of Micronesia	Both	13 (8 to 21)	20.5 (12.5 to 30.2)	1 (0 to 3)	1.9 (0.5 to 4.8)	2 (1 to 4)	2.7 (1.1 to 5.2)	1 (0 to 2)	1.5 (0.7 to 2.8)	1 (0 to 3)	1 (0.1 to 4.1)	1 (0 to 2)	1.6 (0.5 to 2.9)	3 (1 to 8)	4.9 (1.1 to 11.6)	2 (1 to 4)	3.4 (1.4 to 6.5)	1 (0 to 3)	1.5 (0.5 to 2.9)
	Male	11 (6 to 17)	35.8 (21.8 to 55.7)	1 (0 to 2)	3.1 (0.8 to 7.9)	1 (0 to 3)	3.9 (1.4 to 8.4)	1 (0 to 1)	2.2 (0.9 to 4.2)	0 (0 to 2)	1.6 (0.1 to 6.5)	1 (0 to 2)	2.5 (0.7 to 4.9)	2 (0 to 6)	7.7 (1.3 to 19.7)	2 (1 to 4)	7.4 (3.1 to 14.1)	1 (0 to 2)	2.4 (0.7 to 4.9)
	Female	3 (2 to 4)	8.3 (5.1 to 12)	0 (0 to 1)	0.9 (0.2 to 2.5)	1 (0 to 1)	1.8 (0.8 to 3.2)	0 (0 to 1)	1 (0.4 to 1.8)	0 (0 to 1)	0.6 (0 to 2.4)	0 (0 to 1)	0.9 (0.3 to 1.6)	1 (0 to 2)	2.7 (0.6 to 6.7)	0 (0 to 0)	0.3 (0.1 to 0.8)	0 (0 to 1)	0.6 (0.2 to 1.2)
Monaco	Both	41 (34 to 49)	44.3 (35.8 to 53.7)	5 (2 to 8)	5.1 (2.7 to 8.1)	0 (0 to 0)	0 (0 to 0)	2 (1 to 4)	2.9 (1.6 to 4.4)	3 (0 to 9)	3 (0.3 to 10)	1 (0 to 1)	0.7 (0.2 to 1.3)	5 (1 to 12)	5.2 (1.2 to 11.7)	15 (10 to 21)	14.7 (9.5 to 20.7)	2 (0 to 4)	2.4 (0.4 to 4.9)
	Male	29 (24 to 34)	65.6 (54.1	3 (2 to 5)	7 (3.7 to 11.1)	0 (0 to 0)	0 (0 to 0)	2 (1 to 3)	3.9 (2.2	2 (0 to 6)	4.1 (0.5	0 (0 to 1)	0.9 (0.2	3 (1 to 8)	7.6 (1.3 to 18)	15 (10 to 20)	31 (20.1	2 (0 to 3)	4 (0.6 to 8.1)

			to 78.2)						to 6.1)		to 13.6)		to 1.9)				to 43.4)		
	Female	13 (9 to 16)	25.8 (19.2 to 33)	2 (1 to 3)	3.4 (1.8 to 5.5)	0 (0 to 0)	0 (0 to 0)	1 (0 to 1)	1.9 (1 to 3)	1 (0 to 3)	2 (0.2 to 6.7)	0 (0 to 0)	0.5 (0.1 to 1)	2 (0 to 4)	3.1 (0.6 to 7.5)	1 (0 to 1)	1.2 (0.5 to 2.3)	0 (0 to 1)	1 (0.1 to 2.1)
Mongolia	Both	411 (314 to 535)	20.4 (15.8 to 26)	120 (75 to 177)	5.9 (3.8 to 8.6)	50 (22 to 89)	2.5 (1.1 to 4.5)	30 (16 to 49)	1.5 (0.8 to 2.4)	65 (5 to 201)	3.2 (0.2 to 10)	43 (17 to 72)	2.1 (0.8 to 3.5)	25 (5 to 61)	1.4 (0.3 to 3.2)	25 (12 to 47)	1.3 (0.6 to 2.4)	38 (17 to 67)	1.4 (0.6 to 2.4)
	Male	380 (286 to 496)	46.5 (36.3 to 58.8)	96 (59 to 141)	11.4 (7.2 to 16.4)	36 (15 to 67)	4.3 (1.8 to 7.9)	19 (10 to 33)	2.3 (1.2 to 3.8)	51 (4 to 157)	6 (0.4 to 18.9)	34 (13 to 57)	4 (1.5 to 6.6)	20 (3 to 51)	2.6 (0.4 to 6.7)	24 (11 to 45)	3.1 (1.4 to 5.6)	32 (14 to 58)	2.6 (1.1 to 4.6)
	Female	31 (23 to 41)	3 (2.2 to 4.1)	24 (15 to 37)	2.3 (1.4 to 3.4)	14 (7 to 24)	1.3 (0.6 to 2.3)	10 (6 to 17)	0.9 (0.5 to 1.4)	14 (1 to 43)	1.3 (0.1 to 4)	9 (4 to 16)	0.9 (0.4 to 1.4)	5 (1 to 13)	0.5 (0.1 to 1.3)	1 (1 to 3)	0.1 (0.1 to 0.2)	6 (3 to 12)	0.4 (0.2 to 0.8)
Montenegro	Both	432 (355 to 527)	42.8 (35.3 to 52.3)	75 (50 to 102)	7.5 (5.1 to 10.3)	20 (6 to 46)	2 (0.6 to 4.6)	46 (26 to 69)	4.6 (2.6 to 7)	22 (2 to 64)	2.2 (0.2 to 6.4)	7 (2 to 12)	0.7 (0.2 to 1.2)	61 (13 to 138)	5.9 (1.3 to 13.5)	11 (6 to 18)	1.1 (0.6 to 1.8)	16 (3 to 33)	1.6 (0.3 to 3.2)
	Male	337 (272 to 415)	74.3 (60.1 to 91.4)	57 (38 to 79)	12.6 (8.5 to 17.4)	14 (4 to 31)	3.1 (0.9 to 6.9)	34 (19 to 53)	7.5 (4.2 to 11.6)	16 (2 to 49)	3.6 (0.4 to 10.8)	5 (1 to 9)	1.1 (0.3 to 2)	47 (8 to 109)	10.3 (1.8 to 24.1)	8 (4 to 15)	1.8 (0.9 to 3.3)	13 (2 to 27)	2.7 (0.5 to 5.6)
	Female	94 (75 to 118)	17.3 (13.8 to 21.7)	18 (12 to 25)	3.4 (2.2 to 4.7)	7 (2 to 14)	1.2 (0.4 to 2.6)	12 (7 to 18)	2.2 (1.3 to 3.4)	5 (1 to 16)	1 (0.1 to 2.9)	2 (0 to 3)	0.4 (0.1 to 0.6)	14 (3 to 33)	2.5 (0.5 to 5.9)	3 (2 to 5)	0.5 (0.3 to 0.9)	3 (0 to 6)	0.6 (0.1 to 1.2)
Morocco	Both	3210 (2250 to 4200)	10.1 (7.1 to 13.1)	1120 (717 to 1590)	3.6 (2.3 to 5)	60 (23 to 128)	0.2 (0.1 to 0.4)	293 (154 to 478)	0.9 (0.5 to 1.5)	315 (33 to 905)	1 (0.1 to 2.9)	77 (18 to 141)	0.3 (0.1 to 0.4)	576 (116 to 1370)	1.9 (0.4 to 4.6)	43 (6 to 216)	0.2 (0 to 0.8)	369 (156 to 661)	1 (0.4 to 1.8)
	Male	3170 (2220 to 4150)	20.1 (14.1 to 26.1)	984 (620 to 1410)	6.3 (4 to 8.9)	49 (19 to 109)	0.3 (0.1 to 0.7)	247 (129 to 405)	1.6 (0.8 to 2.6)	276 (28 to 790)	1.8 (0.2 to 5.1)	66 (14 to 123)	0.4 (0.1 to 0.8)	505 (89 to 1230)	3.4 (0.6 to 8.2)	39 (3 to 212)	0.3 (0 to 1.7)	355 (148 to 640)	1.9 (0.8 to 3.5)
	Female	41 (28 to 58)	0.3 (0.2 to 0.4)	136 (88 to 197)	0.9 (0.6 to 1.3)	10 (4 to 20)	0.1 (0 to 0.1)	46 (25 to 74)	0.3 (0.2 to 0.5)	39 (4 to 115)	0.3 (0 to 0.8)	12 (3 to 21)	0.1 (0 to 0.1)	70 (14 to 167)	0.5 (0.1 to 1.2)	5 (2 to 10)	0 (0 to 0.1)	14 (5 to 27)	0.1 (0 to 0.1)

Mozambique	Both	306 (227 to 409)	3.2 (2.4 to 4.2)	25 (9 to 53)	0.2 (0.1 to 0.5)	296 (186 to 439)	3 (1.9 to 4.3)	15 (6 to 28)	0.1 (0.1 to 0.3)	31 (2 to 120)	0.3 (0 to 1.2)	53 (18 to 91)	0.5 (0.2 to 0.9)	61 (12 to 145)	0.7 (0.1 to 1.6)	49 (19 to 151)	0.6 (0.2 to 1.8)	46 (20 to 82)	0.4 (0.2 to 0.7)
	Male	275 (202 to 365)	7 (5.3 to 9)	19 (7 to 41)	0.5 (0.2 to 1)	201 (130 to 295)	4.8 (3.1 to 6.9)	9 (4 to 17)	0.2 (0.1 to 0.4)	22 (1 to 85)	0.5 (0 to 2.1)	37 (13 to 63)	0.9 (0.3 to 1.5)	47 (7 to 113)	1.2 (0.2 to 3)	38 (12 to 141)	1.2 (0.4 to 4.1)	31 (13 to 58)	0.6 (0.2 to 1)
	Female	31 (18 to 49)	0.6 (0.3 to 0.9)	6 (2 to 13)	0.1 (0 to 0.2)	95 (52 to 156)	1.7 (0.9 to 2.7)	6 (2 to 11)	0.1 (0 to 0.2)	10 (1 to 36)	0.2 (0 to 0.6)	16 (5 to 31)	0.3 (0.1 to 0.5)	14 (3 to 38)	0.3 (0 to 0.7)	11 (3 to 23)	0.2 (0.1 to 0.5)	14 (5 to 29)	0.2 (0.1 to 0.4)
Myanmar	Both	6170 (4470 to 8600)	14.3 (10.4 to 19.7)	1120 (609 to 1790)	2.5 (1.4 to 4)	2110 (1200 to 3310)	4.7 (2.7 to 7.4)	415 (195 to 706)	0.9 (0.4 to 1.6)	231 (18 to 848)	0.5 (0 to 1.9)	583 (192 to 999)	1.3 (0.4 to 2.2)	1000 (225 to 2370)	2.4 (0.5 to 5.8)	372 (188 to 646)	0.9 (0.5 to 1.6)	579 (240 to 1060)	1.1 (0.5 to 2)
	Male	4930 (3480 to 7280)	26.8 (18.9 to 39.4)	811 (425 to 1380)	4.3 (2.2 to 7.4)	1340 (704 to 2300)	7.1 (3.7 to 12.1)	240 (107 to 439)	1.3 (0.6 to 2.4)	157 (13 to 559)	0.8 (0.1 to 2.9)	393 (127 to 731)	2.1 (0.7 to 3.8)	650 (107 to 1700)	3.8 (0.6 to 9.7)	342 (162 to 610)	2.1 (1 to 3.8)	430 (169 to 818)	1.8 (0.7 to 3.5)
	Female	1240 (922 to 1680)	5.2 (3.9 to 7.2)	304 (165 to 500)	1.2 (0.6 to 2)	767 (486 to 1100)	3 (1.9 to 4.4)	175 (84 to 288)	0.7 (0.3 to 1.1)	74 (6 to 264)	0.3 (0 to 1)	190 (65 to 313)	0.8 (0.3 to 1.3)	351 (72 to 840)	1.5 (0.3 to 3.5)	30 (14 to 57)	0.1 (0.1 to 0.2)	149 (59 to 278)	0.5 (0.2 to 1)
Namibia	Both	47 (37 to 59)	3.7 (2.9 to 4.6)	14 (8 to 21)	1 (0.6 to 1.6)	12 (6 to 21)	0.9 (0.5 to 1.5)	3 (1 to 5)	0.2 (0.1 to 0.4)	4 (0 to 17)	0.3 (0 to 1.2)	7 (2 to 11)	0.5 (0.2 to 0.8)	9 (2 to 20)	0.7 (0.2 to 1.6)	28 (15 to 42)	2.2 (1.2 to 3.3)	2 (1 to 4)	0.2 (0.1 to 0.3)
	Male	34 (27 to 43)	6.7 (5.3 to 8.3)	10 (5 to 15)	1.8 (1 to 2.7)	8 (4 to 13)	1.4 (0.7 to 2.4)	2 (1 to 3)	0.3 (0.2 to 0.6)	3 (0 to 11)	0.5 (0 to 2.1)	4 (2 to 7)	0.8 (0.3 to 1.3)	6 (1 to 15)	1.2 (0.2 to 2.9)	26 (13 to 40)	5.2 (2.7 to 7.8)	2 (1 to 3)	0.3 (0.1 to 0.5)
	Female	13 (9 to 17)	1.7 (1.2 to 2.3)	4 (2 to 7)	0.5 (0.3 to 0.9)	5 (3 to 8)	0.6 (0.3 to 1)	1 (0 to 2)	0.1 (0.1 to 0.2)	1 (0 to 6)	0.2 (0 to 0.7)	2 (1 to 4)	0.3 (0.1 to 0.5)	3 (1 to 7)	0.4 (0.1 to 1)	2 (1 to 4)	0.3 (0.1 to 0.5)	1 (0 to 1)	0.1 (0 to 0.1)
Nauru	Both	1 (0 to 1)	24.3 (16.2 to 32.4)	0 (0 to 0)	1.1 (0.2 to 2.8)	0 (0 to 0)	0.3 (0.1 to 0.9)	0 (0 to 0)	2 (0.9 to 3.5)	0 (0 to 0)	1.1 (0.1 to 4.6)	0 (0 to 0)	1.7 (0.5 to 3)	0 (0 to 0)	5.6 (1.2 to 12.8)	0 (0 to 0)	2.8 (1.4 to 5.3)	0 (0 to 0)	1.8 (0.7 to 3.4)
	Male	1 (0 to 1)	39.7 (25.9	0 (0 to 0)	1.6 (0.3	0 (0 to 0)	0.4 (0.1	0 (0 to 0)	2.8 (1.2	0 (0 to 0)	1.7 (0.1	0 (0 to 0)	2.6 (0.7	0 (0 to 0)	8.7 (1.5	0 (0 to 0)	5.7 (2.6	0 (0 to 0)	3.1 (1.1 to 6)

			to 54.7)		to 4.3)		to 1.2)		to 4.9)		to 7.1)		to 4.6)		to 21.3)		to 11.2)		
	Female	0 (0 to 0)	11.2 (6.7 to 16.9)	0 (0 to 0)	0.6 (0.2 to 1.6)	0 (0 to 0)	0.2 (0 to 0.7)	0 (0 to 0)	1.4 (0.6 to 2.5)	0 (0 to 0)	0.6 (0 to 2.7)	0 (0 to 0)	1 (0.3 to 1.8)	0 (0 to 0)	2.9 (0.5 to 7.4)	0 (0 to 0)	0.4 (0.1 to 0.9)	0 (0 to 0)	0.8 (0.3 to 1.7)
Nepal	Both	973 (710 to 1270)	4.8 (3.5 to 6.2)	298 (156 to 466)	1.4 (0.7 to 2.2)	329 (183 to 522)	1.5 (0.9 to 2.5)	65 (31 to 112)	0.3 (0.1 to 0.5)	108 (11 to 379)	0.5 (0.1 to 1.8)	94 (27 to 163)	0.4 (0.1 to 0.8)	158 (33 to 370)	0.8 (0.2 to 1.8)	91 (43 to 156)	0.5 (0.2 to 0.8)	127 (53 to 232)	0.5 (0.2 to 0.9)
	Male	750 (545 to 964)	7.6 (5.6 to 9.7)	218 (115 to 338)	2.1 (1.1 to 3.3)	211 (113 to 341)	2.1 (1.1 to 3.4)	45 (21 to 76)	0.4 (0.2 to 0.8)	74 (7 to 256)	0.7 (0.1 to 2.5)	64 (19 to 110)	0.6 (0.2 to 1.1)	115 (19 to 278)	1.2 (0.2 to 2.8)	76 (33 to 134)	0.9 (0.4 to 1.5)	91 (37 to 170)	0.8 (0.3 to 1.4)
	Female	223 (143 to 335)	2.2 (1.4 to 3.2)	80 (38 to 138)	0.7 (0.3 to 1.2)	118 (65 to 196)	1.1 (0.6 to 1.8)	21 (9 to 38)	0.2 (0.1 to 0.3)	34 (3 to 123)	0.3 (0 to 1.1)	30 (9 to 53)	0.3 (0.1 to 0.5)	44 (8 to 113)	0.4 (0.1 to 1.1)	15 (6 to 32)	0.2 (0.1 to 0.3)	36 (13 to 70)	0.3 (0.1 to 0.5)
Netherlands	Both	9550 (8850 to 10300)	27.3 (25.4 to 29.3)	1090 (720 to 1560)	3.2 (2.1 to 4.5)	1 (0 to 4)	0 (0 to 0)	445 (255 to 681)	1.4 (0.8 to 2.1)	271 (38 to 705)	0.8 (0.1 to 2)	453 (107 to 682)	1.3 (0.3 to 2)	1000 (229 to 2220)	2.8 (0.6 to 6.2)	4290 (3180 to 5320)	11.6 (8.6 to 14.5)	451 (79 to 870)	1.4 (0.2 to 2.6)
	Male	6040 (5570 to 6510)	37.3 (34.3 to 40.3)	663 (436 to 957)	4.1 (2.7 to 6)	1 (0 to 2)	0 (0 to 0)	277 (156 to 426)	1.8 (1 to 2.7)	165 (24 to 431)	1 (0.1 to 2.7)	271 (64 to 415)	1.7 (0.4 to 2.6)	666 (110 to 1570)	4 (0.7 to 9.5)	3800 (2800 to 4760)	22.9 (16.8 to 28.8)	310 (51 to 610)	1.9 (0.3 to 3.7)
	Female	3520 (3190 to 3860)	19.6 (17.8 to 21.4)	427 (279 to 611)	2.4 (1.6 to 3.4)	1 (0 to 2)	0 (0 to 0)	168 (97 to 257)	1 (0.6 to 1.6)	106 (15 to 276)	0.6 (0.1 to 1.6)	182 (43 to 280)	1 (0.2 to 1.6)	334 (63 to 790)	1.8 (0.3 to 4.2)	494 (280 to 738)	2.4 (1.4 to 3.6)	141 (21 to 283)	0.9 (0.1 to 1.7)
New Zealand	Both	1200 (1090 to 1290)	15 (13.8 to 16.2)	47 (8 to 101)	0.6 (0.1 to 1.3)	0 (0 to 1)	0 (0 to 0)	56 (32 to 87)	0.7 (0.4 to 1.2)	59 (6 to 193)	0.7 (0.1 to 2.4)	68 (17 to 103)	0.9 (0.2 to 1.3)	149 (35 to 327)	1.8 (0.4 to 4)	509 (378 to 634)	6.1 (4.5 to 7.6)	83 (15 to 159)	1.1 (0.2 to 2.1)
	Male	666 (608 to 728)	17.8 (16.3 to 19.5)	25 (4 to 53)	0.7 (0.1 to 1.4)	0 (0 to 1)	0 (0 to 0)	33 (18 to 50)	0.9 (0.5 to 1.4)	31 (3 to 103)	0.8 (0.1 to 2.8)	35 (9 to 54)	1 (0.2 to 1.4)	84 (14 to 198)	2.2 (0.4 to 5.2)	440 (319 to 559)	11.4 (8.3 to 14.6)	52 (9 to 101)	1.4 (0.2 to 2.7)
	Female	529 (470 to 582)	12.6 (11.3 to 13.9)	22 (4 to 47)	0.5 (0.1 to 1.1)	0 (0 to 1)	0 (0 to 0)	23 (13 to 36)	0.6 (0.3 to 0.9)	28 (3 to 89)	0.7 (0.1 to 2.1)	33 (8 to 51)	0.8 (0.2 to 1.2)	65 (12 to 154)	1.5 (0.3 to 3.5)	69 (39 to 101)	1.5 (0.9 to 2.3)	31 (5 to 61)	0.8 (0.1 to 1.5)

Nicaragua	Both	141 (112 to 174)	3.5 (2.8 to 4.3)	33 (16 to 54)	0.8 (0.4 to 1.3)	54 (31 to 82)	1.3 (0.8 to 2)	13 (6 to 20)	0.3 (0.2 to 0.5)	17 (1 to 66)	0.4 (0 to 1.6)	22 (8 to 35)	0.5 (0.2 to 0.8)	45 (11 to 99)	1.2 (0.3 to 2.5)	9 (5 to 14)	0.2 (0.1 to 0.4)	22 (11 to 37)	0.5 (0.2 to 0.8)
	Male	124 (97 to 153)	7.2 (5.7 to 8.8)	22 (11 to 36)	1.2 (0.6 to 2.1)	30 (16 to 49)	1.7 (0.9 to 2.8)	8 (4 to 12)	0.4 (0.2 to 0.7)	10 (1 to 41)	0.6 (0 to 2.3)	13 (5 to 21)	0.8 (0.3 to 1.2)	29 (5 to 67)	1.7 (0.3 to 3.9)	7 (4 to 12)	0.5 (0.2 to 0.8)	15 (7 to 26)	0.7 (0.3 to 1.2)
	Female	17 (13 to 23)	0.7 (0.5 to 1)	11 (5 to 18)	0.5 (0.2 to 0.8)	24 (14 to 34)	1 (0.6 to 1.5)	5 (3 to 8)	0.2 (0.1 to 0.3)	6 (1 to 25)	0.3 (0 to 1.1)	9 (3 to 13)	0.4 (0.1 to 0.6)	16 (3 to 37)	0.7 (0.1 to 1.7)	2 (1 to 3)	0.1 (0 to 0.1)	7 (3 to 13)	0.3 (0.1 to 0.5)
Niger	Both	149 (90 to 231)	2.3 (1.4 to 3.5)	33 (7 to 90)	0.5 (0.1 to 1.3)	241 (122 to 467)	3.5 (1.7 to 6.7)	17 (8 to 30)	0.2 (0.1 to 0.4)	29 (2 to 108)	0.4 (0 to 1.6)	41 (13 to 75)	0.6 (0.2 to 1.1)	33 (6 to 87)	0.5 (0.1 to 1.4)	10 (3 to 29)	0.2 (0.1 to 0.5)	32 (13 to 64)	0.4 (0.1 to 0.7)
	Male	145 (87 to 225)	4.6 (2.8 to 6.9)	27 (6 to 75)	0.8 (0.2 to 2.3)	187 (93 to 367)	5.7 (2.8 to 11.1)	12 (5 to 23)	0.3 (0.2 to 0.7)	23 (2 to 87)	0.7 (0 to 2.6)	33 (10 to 62)	1 (0.3 to 1.9)	26 (4 to 72)	0.9 (0.1 to 2.4)	8 (2 to 27)	0.3 (0.1 to 0.9)	26 (10 to 54)	0.6 (0.2 to 1.2)
	Female	4 (2 to 8)	0.1 (0.1 to 0.2)	5 (1 to 14)	0.1 (0 to 0.4)	53 (26 to 111)	1.5 (0.7 to 3.1)	4 (2 to 8)	0.1 (0 to 0.2)	6 (0 to 24)	0.2 (0 to 0.7)	9 (3 to 17)	0.2 (0.1 to 0.5)	7 (1 to 19)	0.2 (0 to 0.6)	1 (0 to 3)	0 (0 to 0.1)	6 (2 to 13)	0.1 (0 to 0.3)
Nigeria	Both	1280 (934 to 1790)	1.7 (1.3 to 2.4)	1010 (563 to 1540)	1.3 (0.8 to 2)	1010 (585 to 1560)	1.3 (0.8 to 2)	172 (88 to 286)	0.2 (0.1 to 0.4)	281 (60 to 587)	0.4 (0.1 to 0.8)	283 (77 to 472)	0.4 (0.1 to 0.6)	354 (74 to 861)	0.5 (0.1 to 1.3)	88 (39 to 250)	0.1 (0.1 to 0.4)	186 (85 to 322)	0.2 (0.1 to 0.3)
	Male	1180 (840 to 1680)	3.3 (2.4 to 4.6)	702 (373 to 1110)	2 (1.1 to 3.1)	704 (384 to 1160)	1.9 (1.1 to 3.2)	126 (62 to 217)	0.3 (0.2 to 0.6)	194 (39 to 420)	0.5 (0.1 to 1.1)	195 (55 to 339)	0.5 (0.2 to 0.9)	258 (41 to 658)	0.8 (0.1 to 2)	67 (23 to 228)	0.2 (0.1 to 0.7)	127 (52 to 235)	0.3 (0.1 to 0.5)
	Female	100 (63 to 151)	0.3 (0.2 to 0.4)	304 (164 to 478)	0.8 (0.4 to 1.2)	306 (178 to 477)	0.7 (0.4 to 1.1)	46 (23 to 78)	0.1 (0.1 to 0.2)	87 (19 to 185)	0.2 (0 to 0.4)	89 (28 to 151)	0.2 (0.1 to 0.4)	96 (17 to 250)	0.3 (0 to 0.7)	20 (9 to 37)	0.1 (0 to 0.1)	59 (25 to 107)	0.1 (0 to 0.2)
Niue	Both	0 (0 to 0)	16.9 (13.4 to 20.9)	0 (0 to 0)	1.1 (0.2 to 2.7)	0 (0 to 0)	0.2 (0 to 0.6)	0 (0 to 0)	2.2 (1.2 to 3.5)	0 (0 to 0)	1 (0.1 to 3.7)	0 (0 to 0)	1.3 (0.3 to 2.1)	0 (0 to 0)	5.9 (1.5 to 12.4)	0 (0 to 0)	1.7 (0.9 to 3.2)	0 (0 to 0)	1.2 (0.5 to 2.1)
	Male	0 (0 to 0)	30 (24.6	0 (0 to 0)	1.6 (0.3	0 (0 to 0)	0.3 (0 to 0.8)	0 (0 to 0)	3 (1.7 to 4.7)	0 (0 to 0)	1.4 (0.1	0 (0 to 0)	1.9 (0.5	0 (0 to 0)	9 (1.7 to 19.8)	0 (0 to 0)	3.8 (1.9	0 (0 to 0)	1.8 (0.8

			to 35.9)		to 3.9)						to 5.6)		to 3.1)				to 7.4)		to 3.2)
	Female	0 (0 to 0)	7 (4.7 to 9.8)	0 (0 to 0)	0.7 (0.2 to 1.7)	0 (0 to 0)	0.2 (0 to 0.5)	0 (0 to 0)	1.6 (0.9 to 2.8)	0 (0 to 0)	0.6 (0 to 2.3)	0 (0 to 0)	0.8 (0.2 to 1.4)	0 (0 to 0)	3.6 (0.8 to 8.4)	0 (0 to 0)	0.2 (0.1 to 0.5)	0 (0 to 0)	0.6 (0.2 to 1.2)
North Macedonia	Both	1020 (784 to 1300)	30.6 (23.6 to 39)	236 (158 to 332)	7.2 (4.8 to 10)	41 (13 to 90)	1.2 (0.4 to 2.7)	103 (57 to 162)	3.1 (1.7 to 4.9)	112 (18 to 280)	3.4 (0.5 to 8.5)	35 (7 to 59)	1.1 (0.2 to 1.8)	160 (33 to 370)	4.8 (1 to 11)	11 (4 to 23)	0.3 (0.1 to 0.7)	47 (7 to 98)	1.4 (0.2 to 2.8)
	Male	866 (656 to 1110)	54.1 (41.2 to 69.1)	193 (130 to 272)	12.2 (8.3 to 17)	30 (9 to 68)	1.9 (0.6 to 4.3)	81 (44 to 129)	5.1 (2.8 to 8)	91 (14 to 230)	5.7 (0.9 to 14.5)	28 (6 to 48)	1.8 (0.4 to 3)	131 (23 to 315)	8.3 (1.5 to 19.6)	9 (3 to 20)	0.6 (0.2 to 1.3)	40 (6 to 84)	2.3 (0.3 to 4.9)
	Female	154 (119 to 198)	9.1 (7 to 11.7)	43 (29 to 60)	2.6 (1.7 to 3.6)	10 (4 to 22)	0.6 (0.2 to 1.3)	22 (13 to 35)	1.3 (0.8 to 2.1)	21 (3 to 53)	1.3 (0.2 to 3.2)	7 (2 to 12)	0.4 (0.1 to 0.7)	28 (6 to 66)	1.6 (0.3 to 3.9)	2 (1 to 4)	0.1 (0.1 to 0.3)	7 (1 to 15)	0.4 (0 to 0.9)
Northern Mariana Islands	Both	13 (11 to 15)	26.8 (23.2 to 30.4)	1 (1 to 2)	2.4 (1.2 to 3.9)	1 (0 to 1)	1.1 (0.3 to 2.7)	1 (1 to 2)	2.5 (1.4 to 3.9)	1 (0 to 3)	1.3 (0.1 to 5.4)	1 (0 to 1)	1.5 (0.4 to 2.4)	3 (1 to 6)	6.4 (1.5 to 13.7)	1 (0 to 1)	1.9 (1.2 to 3)	1 (1 to 2)	2 (0.9 to 3.3)
	Male	11 (9 to 12)	47 (40.7 to 53.3)	1 (0 to 1)	3.7 (1.8 to 6.1)	0 (0 to 1)	1.6 (0.4 to 3.9)	1 (0 to 1)	3.6 (2 to 5.7)	0 (0 to 2)	2.1 (0.1 to 8.4)	1 (0 to 1)	2.4 (0.6 to 3.7)	2 (0 to 5)	10.1 (1.9 to 22)	1 (0 to 1)	4 (2.4 to 6)	1 (0 to 2)	2.7 (1.2 to 4.7)
	Female	2 (1 to 2)	7.3 (5.4 to 9.8)	0 (0 to 0)	1.1 (0.6 to 1.9)	0 (0 to 0)	0.7 (0.2 to 1.6)	0 (0 to 1)	1.4 (0.8 to 2.4)	0 (0 to 1)	0.6 (0 to 2.6)	0 (0 to 0)	0.8 (0.2 to 1.3)	1 (0 to 2)	2.9 (0.6 to 6.6)	0 (0 to 0)	0.1 (0.1 to 0.3)	0 (0 to 1)	1.1 (0.5 to 2)
Norway	Both	1360 (1250 to 1470)	14.1 (13 to 15.2)	67 (23 to 128)	0.7 (0.2 to 1.3)	0 (0 to 1)	0 (0 to 0)	76 (45 to 118)	0.8 (0.5 to 1.3)	152 (29 to 313)	1.6 (0.3 to 3.2)	73 (18 to 111)	0.8 (0.2 to 1.1)	239 (57 to 518)	2.4 (0.6 to 5.2)	586 (424 to 764)	5.7 (4.1 to 7.4)	83 (16 to 156)	0.9 (0.2 to 1.7)
	Male	815 (749 to 884)	17.8 (16.3 to 19.3)	36 (12 to 69)	0.8 (0.3 to 1.5)	0 (0 to 0)	0 (0 to 0)	45 (26 to 69)	1 (0.6 to 1.5)	83 (16 to 171)	1.8 (0.3 to 3.8)	39 (10 to 60)	0.9 (0.2 to 1.3)	136 (22 to 312)	2.9 (0.5 to 6.7)	465 (323 to 613)	9.9 (6.9 to 13.1)	55 (10 to 104)	1.2 (0.2 to 2.3)
	Female	547 (487 to 610)	11 (9.9 to 12.2)	32 (11 to 59)	0.6 (0.2 to 1.2)	0 (0 to 0)	0 (0 to 0)	32 (18 to 48)	0.7 (0.4 to 1)	69 (13 to 143)	1.4 (0.3 to 2.8)	34 (9 to 53)	0.7 (0.2 to 1)	103 (20 to 242)	1.9 (0.4 to 4.5)	121 (68 to 239)	2.2 (1.2 to 4.3)	27 (5 to 54)	0.6 (0.1 to 1.2)

Oman	Both	60 (46 to 82)	4.6 (3.7 to 5.8)	35 (23 to 52)	2.6 (1.8 to 3.5)	0 (0 to 0)	0 (0 to 0)	8 (5 to 13)	0.6 (0.3 to 0.9)	4 (0 to 16)	0.3 (0 to 1.1)	2 (1 to 4)	0.1 (0 to 0.2)	17 (4 to 39)	1.5 (0.3 to 3.2)	17 (9 to 27)	1.6 (0.9 to 2.4)	9 (4 to 17)	0.4 (0.2 to 0.8)
	Male	56 (42 to 79)	8.3 (6.5 to 10.5)	25 (16 to 39)	3.5 (2.4 to 4.9)	0 (0 to 0)	0 (0 to 0)	5 (3 to 10)	0.8 (0.4 to 1.3)	3 (0 to 12)	0.4 (0 to 1.5)	1 (0 to 3)	0.2 (0 to 0.3)	13 (2 to 30)	2.1 (0.4 to 4.7)	17 (9 to 26)	3.2 (1.9 to 4.8)	8 (3 to 16)	0.7 (0.3 to 1.3)
	Female	4 (3 to 6)	0.7 (0.5 to 1)	10 (7 to 14)	1.6 (1.1 to 2.2)	0 (0 to 0)	0 (0 to 0)	3 (1 to 4)	0.4 (0.2 to 0.6)	1 (0 to 4)	0.2 (0 to 0.7)	1 (0 to 1)	0.1 (0 to 0.2)	4 (1 to 10)	0.8 (0.2 to 1.9)	0 (0 to 1)	0.1 (0 to 0.1)	1 (0 to 1)	0.1 (0 to 0.1)
Pakistan	Both	10700 (7920 to 14300)	10.4 (7.8 to 13.8)	3280 (1950 to 4940)	3 (1.8 to 4.5)	2620 (1440 to 4090)	2.4 (1.3 to 3.8)	824 (433 to 1340)	0.8 (0.4 to 1.2)	872 (132 to 2320)	0.8 (0.1 to 2.2)	1000 (340 to 1690)	0.9 (0.3 to 1.5)	1640 (328 to 4060)	1.6 (0.3 to 4)	522 (232 to 1160)	0.6 (0.3 to 1.3)	1490 (649 to 2600)	1.2 (0.5 to 2)
	Male	10200 (7440 to 13800)	19.2 (14.1 to 25.6)	2820 (1640 to 4330)	5.1 (2.9 to 7.7)	2120 (1110 to 3420)	3.8 (2 to 6.1)	635 (325 to 1070)	1.1 (0.6 to 1.9)	733 (109 to 1960)	1.3 (0.2 to 3.5)	840 (278 to 1430)	1.5 (0.5 to 2.5)	1380 (233 to 3570)	2.6 (0.5 to 6.7)	463 (193 to 1110)	1.1 (0.5 to 2.4)	1420 (609 to 2510)	2.1 (0.9 to 3.7)
	Female	551 (380 to 773)	1.1 (0.8 to 1.6)	462 (266 to 742)	0.9 (0.5 to 1.4)	497 (286 to 750)	1 (0.5 to 1.4)	189 (96 to 317)	0.4 (0.2 to 0.6)	140 (21 to 383)	0.3 (0 to 0.7)	163 (55 to 276)	0.3 (0.1 to 0.5)	258 (50 to 637)	0.5 (0.1 to 1.4)	60 (23 to 134)	0.1 (0.1 to 0.3)	65 (26 to 122)	0.1 (0 to 0.2)
Palau	Both	4 (3 to 5)	20.2 (16 to 25.6)	0 (0 to 1)	1.4 (0 to 3.9)	0 (0 to 0)	0 (0 to 0)	1 (0 to 1)	3.2 (1.8 to 5.2)	0 (0 to 1)	1.4 (0.1 to 5.4)	0 (0 to 1)	1.8 (0.4 to 3)	1 (0 to 3)	7.3 (1.8 to 15.5)	0 (0 to 1)	1.7 (0.9 to 3.4)	1 (0 to 1)	1.9 (0.9 to 3.2)
	Male	3 (2 to 4)	29.9 (24.1 to 37.4)	0 (0 to 0)	1.6 (0 to 4.1)	0 (0 to 0)	0 (0 to 0)	0 (0 to 0)	3.1 (1.6 to 4.9)	0 (0 to 1)	1.5 (0.1 to 5.7)	0 (0 to 0)	1.9 (0.5 to 3.2)	1 (0 to 2)	7.8 (1.4 to 17.2)	0 (0 to 1)	3.2 (1.4 to 6.4)	0 (0 to 1)	2.3 (1 to 4.1)
	Female	1 (1 to 2)	11.3 (8 to 15.6)	0 (0 to 0)	1.3 (0 to 3.6)	0 (0 to 0)	0 (0 to 0)	0 (0 to 1)	3.4 (1.8 to 5.5)	0 (0 to 0)	1.3 (0.1 to 5)	0 (0 to 0)	1.7 (0.4 to 2.9)	1 (0 to 2)	6.8 (1.5 to 15.3)	0 (0 to 0)	0.5 (0.2 to 1)	0 (0 to 0)	1.5 (0.6 to 2.7)
Palestine	Both	346 (290 to 405)	16 (13.5 to 18.7)	105 (72 to 143)	4.8 (3.3 to 6.4)	1 (1 to 3)	0.1 (0 to 0.1)	39 (24 to 59)	1.7 (1 to 2.6)	26 (3 to 73)	1.2 (0.1 to 3.3)	20 (5 to 32)	0.9 (0.2 to 1.4)	72 (16 to 156)	3.6 (0.8 to 7.6)	4 (2 to 7)	0.2 (0.1 to 0.4)	29 (13 to 49)	1.1 (0.5 to 1.8)
	Male	329 (276 to 385)	33.1 (28.1	84 (57 to 114)	8.3 (5.6	1 (0 to 2)	0.1 (0 to 0.2)	27 (16 to 41)	2.6 (1.5	20 (2 to 58)	2 (0.2 to 5.7)	16 (4 to 26)	1.6 (0.4	59 (10 to 130)	6.4 (1.2 to 14)	3 (2 to 6)	0.5 (0.2	28 (13 to 48)	2.1 (0.9

			to 38.8)		to 11.2)				to 3.9)				to 2.5)				to 0.8)		to 3.5)
	Female	17 (12 to 23)	1.6 (1.1 to 2.2)	22 (15 to 30)	1.8 (1.2 to 2.5)	0 (0 to 1)	0 (0 to 0.1)	12 (7 to 18)	1 (0.6 to 1.5)	5 (1 to 15)	0.5 (0.1 to 1.3)	4 (1 to 7)	0.4 (0.1 to 0.6)	13 (3 to 30)	1.2 (0.3 to 2.8)	0 (0 to 1)	0 (0 to 0.1)	1 (0 to 2)	0.1 (0 to 0.1)
Panama	Both	182 (140 to 235)	4.4 (3.4 to 5.7)	40 (22 to 62)	1 (0.5 to 1.5)	9 (3 to 18)	0.2 (0.1 to 0.4)	12 (6 to 20)	0.3 (0.2 to 0.5)	20 (2 to 74)	0.5 (0 to 1.8)	19 (5 to 30)	0.4 (0.1 to 0.7)	57 (13 to 126)	1.4 (0.3 to 3.1)	13 (6 to 22)	0.3 (0.1 to 0.5)	26 (13 to 45)	0.6 (0.3 to 1.1)
	Male	148 (112 to 191)	7.5 (5.7 to 9.7)	26 (14 to 41)	1.3 (0.7 to 2.1)	5 (2 to 10)	0.3 (0.1 to 0.5)	8 (4 to 14)	0.4 (0.2 to 0.7)	13 (1 to 48)	0.6 (0.1 to 2.4)	12 (3 to 19)	0.6 (0.2 to 1)	38 (7 to 89)	1.9 (0.3 to 4.5)	11 (5 to 20)	0.6 (0.3 to 1.1)	21 (9 to 37)	1 (0.4 to 1.8)
	Female	35 (25 to 46)	1.6 (1.2 to 2.1)	14 (8 to 22)	0.6 (0.4 to 1)	4 (2 to 8)	0.2 (0.1 to 0.4)	4 (2 to 6)	0.2 (0.1 to 0.3)	7 (1 to 28)	0.3 (0 to 1.3)	7 (2 to 11)	0.3 (0.1 to 0.5)	19 (4 to 45)	0.9 (0.2 to 2.1)	2 (1 to 3)	0.1 (0 to 0.1)	6 (2 to 10)	0.3 (0.1 to 0.5)
Papua New Guinea	Both	495 (339 to 765)	11.9 (8.2 to 17.9)	34 (7 to 93)	0.8 (0.2 to 2.2)	284 (174 to 457)	6.6 (4.2 to 10.6)	54 (25 to 101)	1.2 (0.6 to 2.3)	33 (2 to 126)	0.8 (0 to 2.9)	49 (15 to 91)	1.1 (0.3 to 2.1)	121 (24 to 294)	3 (0.6 to 7)	53 (24 to 105)	1.5 (0.7 to 2.8)	36 (15 to 70)	0.6 (0.3 to 1.2)
	Male	429 (286 to 677)	19.5 (13.4 to 30)	29 (6 to 79)	1.3 (0.3 to 3.6)	226 (136 to 371)	10.2 (6.2 to 16.6)	40 (19 to 76)	1.8 (0.8 to 3.5)	27 (2 to 105)	1.2 (0.1 to 4.6)	39 (12 to 75)	1.8 (0.5 to 3.3)	101 (17 to 255)	4.7 (0.8 to 11.6)	51 (23 to 101)	2.8 (1.4 to 5.3)	27 (10 to 54)	0.9 (0.3 to 1.7)
	Female	67 (39 to 107)	3.5 (2 to 5.5)	5 (1 to 13)	0.2 (0 to 0.6)	57 (32 to 97)	2.8 (1.5 to 4.8)	13 (6 to 26)	0.6 (0.3 to 1.2)	6 (0 to 24)	0.3 (0 to 1.2)	9 (3 to 19)	0.5 (0.1 to 0.9)	20 (3 to 55)	1 (0.2 to 2.8)	2 (1 to 4)	0.1 (0 to 0.2)	9 (3 to 19)	0.3 (0.1 to 0.7)
Paraguay	Both	568 (435 to 735)	10.7 (8.3 to 13.8)	67 (37 to 105)	1.2 (0.7 to 1.9)	70 (32 to 120)	1.3 (0.6 to 2.2)	40 (22 to 68)	0.7 (0.4 to 1.3)	28 (3 to 90)	0.5 (0.1 to 1.7)	25 (6 to 41)	0.5 (0.1 to 0.8)	81 (16 to 189)	1.6 (0.3 to 3.6)	41 (20 to 72)	0.8 (0.4 to 1.4)	63 (29 to 110)	1.1 (0.5 to 1.9)
	Male	481 (366 to 626)	19.3 (14.9 to 24.9)	52 (28 to 83)	2.1 (1.1 to 3.3)	49 (21 to 88)	1.9 (0.8 to 3.5)	29 (16 to 50)	1.1 (0.6 to 1.9)	21 (3 to 68)	0.8 (0.1 to 2.7)	19 (4 to 31)	0.7 (0.2 to 1.2)	63 (10 to 152)	2.6 (0.4 to 6.2)	35 (16 to 63)	1.5 (0.7 to 2.7)	53 (23 to 95)	1.9 (0.8 to 3.3)
	Female	87 (64 to 114)	3.1 (2.3 to 4.1)	15 (8 to 23)	0.5 (0.3 to 0.8)	21 (10 to 34)	0.7 (0.4 to 1.2)	11 (6 to 19)	0.4 (0.2 to 0.6)	7 (1 to 21)	0.2 (0 to 0.7)	6 (1 to 10)	0.2 (0 to 0.4)	18 (4 to 46)	0.7 (0.1 to 1.6)	6 (3 to 11)	0.2 (0.1 to 0.4)	10 (4 to 17)	0.3 (0.1 to 0.6)

Peru	Both	639 (451 to 874)	2 (1.4 to 2.8)	597 (363 to 881)	1.9 (1.1 to 2.8)	161 (72 to 286)	0.5 (0.2 to 0.9)	57 (25 to 106)	0.2 (0.1 to 0.3)	127 (9 to 436)	0.4 (0 to 1.4)	130 (32 to 217)	0.4 (0.1 to 0.7)	243 (55 to 570)	0.8 (0.2 to 1.8)	162 (75 to 305)	0.5 (0.2 to 1)	239 (113 to 411)	0.7 (0.3 to 1.3)
	Male	521 (362 to 728)	3.5 (2.4 to 4.8)	326 (198 to 484)	2.1 (1.3 to 3.2)	71 (28 to 132)	0.5 (0.2 to 0.9)	31 (13 to 58)	0.2 (0.1 to 0.4)	67 (5 to 240)	0.4 (0 to 1.6)	68 (17 to 113)	0.4 (0.1 to 0.7)	131 (21 to 333)	0.9 (0.1 to 2.2)	111 (45 to 230)	0.7 (0.3 to 1.5)	150 (64 to 272)	0.9 (0.4 to 1.7)
	Female	119 (78 to 171)	0.7 (0.5 to 1)	271 (162 to 409)	1.6 (1 to 2.5)	90 (43 to 160)	0.5 (0.3 to 1)	26 (12 to 48)	0.2 (0.1 to 0.3)	60 (4 to 209)	0.4 (0 to 1.3)	62 (15 to 103)	0.4 (0.1 to 0.6)	112 (21 to 277)	0.7 (0.1 to 1.7)	51 (22 to 105)	0.3 (0.1 to 0.6)	89 (38 to 161)	0.5 (0.2 to 1)
Philippines	Both	8650 (6810 to 10800)	11.7 (9.3 to 14.5)	1490 (986 to 2170)	2 (1.3 to 2.9)	1610 (903 to 2580)	2.1 (1.2 to 3.4)	877 (474 to 1380)	1.2 (0.6 to 1.8)	295 (56 to 633)	0.4 (0.1 to 0.8)	460 (122 to 741)	0.6 (0.2 to 1)	984 (213 to 2260)	1.4 (0.3 to 3.2)	259 (143 to 450)	0.4 (0.2 to 0.7)	983 (476 to 1600)	1.1 (0.5 to 1.8)
	Male	7240 (5470 to 9310)	21.5 (16.4 to 27.6)	1050 (650 to 1580)	3.1 (1.9 to 4.5)	1030 (555 to 1750)	3 (1.6 to 5.1)	531 (283 to 857)	1.6 (0.9 to 2.6)	202 (37 to 442)	0.6 (0.1 to 1.3)	308 (74 to 518)	0.9 (0.2 to 1.5)	638 (99 to 1640)	2.1 (0.3 to 5.2)	235 (118 to 422)	0.8 (0.4 to 1.5)	777 (348 to 1320)	1.8 (0.8 to 3.1)
	Female	1410 (1080 to 1820)	3.8 (3 to 4.9)	437 (276 to 635)	1.1 (0.7 to 1.6)	576 (325 to 886)	1.4 (0.8 to 2.2)	346 (181 to 566)	0.8 (0.4 to 1.4)	93 (16 to 200)	0.2 (0 to 0.5)	153 (40 to 256)	0.4 (0.1 to 0.6)	347 (68 to 846)	0.9 (0.2 to 2.2)	24 (13 to 40)	0.1 (0 to 0.1)	206 (93 to 353)	0.4 (0.2 to 0.8)
Poland	Both	23200 (19400 to 27700)	32.7 (27.2 to 39.1)	4750 (3360 to 6450)	6.7 (4.8 to 9.2)	466 (130 to 1130)	0.7 (0.2 to 1.6)	1450 (817 to 2250)	2.1 (1.2 to 3.3)	1180 (196 to 2480)	1.7 (0.3 to 3.5)	1390 (388 to 2210)	2 (0.6 to 3.1)	3180 (684 to 6990)	4.4 (0.9 to 9.7)	3600 (2170 to 5460)	4.9 (3 to 7.4)	1290 (229 to 2530)	1.9 (0.3 to 3.6)
	Male	17100 (13400 to 21200)	56.7 (44.7 to 70.3)	3330 (2310 to 4680)	11.2 (7.7 to 15.7)	284 (76 to 699)	1 (0.3 to 2.3)	936 (514 to 1510)	3.1 (1.7 to 5.1)	825 (133 to 1730)	2.8 (0.4 to 5.8)	962 (272 to 1560)	3.2 (0.9 to 5.2)	2330 (408 to 5380)	7.7 (1.4 to 17.9)	3290 (1890 to 5170)	10.9 (6.3 to 17.1)	1000 (174 to 1970)	3.1 (0.5 to 6.2)
	Female	6140 (4810 to 7730)	15.4 (12 to 19.5)	1420 (982 to 1950)	3.5 (2.4 to 4.9)	182 (51 to 438)	0.5 (0.1 to 1.1)	509 (286 to 798)	1.3 (0.7 to 2.1)	357 (57 to 777)	0.9 (0.1 to 1.9)	430 (125 to 701)	1.1 (0.3 to 1.8)	854 (157 to 2030)	2 (0.4 to 4.8)	310 (143 to 498)	0.7 (0.3 to 1.2)	285 (37 to 590)	0.8 (0.1 to 1.6)
Portugal	Both	2920 (2690 to 3130)	13.1 (12.1 to 14.1)	219 (115 to 350)	1 (0.5 to 1.5)	3 (1 to 8)	0 (0 to 0)	182 (105 to 278)	0.9 (0.5 to 1.3)	296 (51 to 721)	1.3 (0.2 to 3.2)	140 (33 to 214)	0.6 (0.1 to 0.9)	572 (128 to 1230)	2.4 (0.5 to 5.1)	521 (309 to 771)	2 (1.2 to 3)	207 (36 to 401)	1 (0.2 to 2)
	Male	2540 (2340	25.3 (23.3	166 (87 to 265)	1.7 (0.9	2 (0 to 6)	0 (0 to 0.1)	128 (74 to 199)	1.3 (0.8	224 (39 to 546)	2.2 (0.4	104 (21 to 161)	1 (0.2 to 1.6)	437 (76 to 987)	4.2 (0.7	497 (286	4.5 (2.6	174 (30 to 337)	1.8 (0.3

		to 2740)	to 27.2)		to 2.6)				to 2.1)		to 5.4)				to 9.4)	to 740)	to 6.7)		to 3.6)
	Female	377 (323 to 430)	3.3 (2.8 to 3.7)	53 (28 to 86)	0.4 (0.2 to 0.7)	1 (0 to 3)	0 (0 to 0)	54 (31 to 82)	0.5 (0.3 to 0.7)	72 (12 to 175)	0.6 (0.1 to 1.3)	36 (9 to 56)	0.3 (0.1 to 0.4)	136 (28 to 307)	0.9 (0.2 to 2.1)	24 (13 to 39)	0.2 (0.1 to 0.2)	33 (4 to 68)	0.3 (0 to 0.6)
Puerto Rico	Both	413 (311 to 528)	5.6 (4.2 to 7.2)	27 (5 to 53)	0.4 (0.1 to 0.7)	0 (0 to 0)	0 (0 to 0)	25 (13 to 42)	0.4 (0.2 to 0.6)	23 (2 to 89)	0.3 (0 to 1.2)	25 (6 to 41)	0.3 (0.1 to 0.6)	130 (32 to 284)	1.7 (0.4 to 3.8)	56 (28 to 94)	0.7 (0.3 to 1.2)	15 (2 to 32)	0.2 (0 to 0.5)
	Male	296 (223 to 381)	9.1 (6.9 to 11.8)	16 (3 to 32)	0.5 (0.1 to 1)	0 (0 to 0)	0 (0 to 0)	16 (8 to 27)	0.5 (0.3 to 0.9)	14 (1 to 54)	0.4 (0 to 1.7)	14 (3 to 24)	0.4 (0.1 to 0.8)	81 (15 to 182)	2.4 (0.5 to 5.5)	53 (27 to 90)	1.5 (0.8 to 2.6)	11 (2 to 23)	0.4 (0.1 to 0.8)
	Female	118 (85 to 154)	2.8 (2 to 3.6)	11 (2 to 21)	0.3 (0.1 to 0.5)	0 (0 to 0)	0 (0 to 0)	9 (5 to 15)	0.2 (0.1 to 0.4)	9 (1 to 36)	0.2 (0 to 0.9)	11 (2 to 18)	0.3 (0.1 to 0.4)	49 (11 to 114)	1.1 (0.2 to 2.6)	3 (1 to 6)	0.1 (0 to 0.1)	5 (1 to 10)	0.1 (0 to 0.3)
Qatar	Both	67 (46 to 97)	12.5 (9.4 to 16.2)	36 (23 to 53)	6.4 (4.4 to 8.8)	0 (0 to 0)	0 (0 to 0)	9 (5 to 14)	1.4 (0.8 to 2.2)	3 (0 to 11)	0.6 (0.1 to 2)	1 (0 to 2)	0.2 (0.1 to 0.3)	24 (6 to 52)	5.1 (1.2 to 10.2)	2 (1 to 4)	0.6 (0.3 to 1.3)	16 (7 to 30)	1.3 (0.5 to 2.4)
	Male	66 (45 to 95)	16.4 (12.3 to 21.3)	31 (19 to 46)	7.3 (5 to 10.3)	0 (0 to 0)	0 (0 to 0)	7 (4 to 12)	1.6 (0.9 to 2.5)	3 (0 to 9)	0.7 (0.1 to 2.3)	1 (0 to 2)	0.2 (0.1 to 0.4)	21 (4 to 46)	5.8 (1.3 to 12.1)	2 (1 to 4)	0.8 (0.3 to 1.6)	15 (6 to 29)	1.6 (0.7 to 3.1)
	Female	1 (1 to 2)	0.9 (0.6 to 1.4)	6 (4 to 8)	3.6 (2.5 to 4.9)	0 (0 to 0)	0 (0 to 0)	2 (1 to 3)	0.9 (0.5 to 1.4)	1 (0 to 2)	0.3 (0 to 1.1)	0 (0 to 0)	0.1 (0 to 0.2)	3 (1 to 7)	2.8 (0.7 to 5.9)	0 (0 to 0)	0.2 (0.1 to 0.4)	1 (0 to 1)	0.2 (0.1 to 0.3)
South Korea	Both	15700 (13900 to 17500)	17.7 (15.6 to 19.7)	4330 (3090 to 5710)	4.9 (3.5 to 6.5)	3 (1 to 8)	0 (0 to 0)	778 (456 to 1180)	0.9 (0.5 to 1.3)	1260 (161 to 3360)	1.4 (0.2 to 3.8)	892 (219 to 1370)	1 (0.2 to 1.6)	2230 (488 to 4890)	2.5 (0.5 to 5.5)	1530 (892 to 2300)	1.8 (1 to 2.6)	929 (158 to 1810)	1 (0.2 to 1.9)
	Male	13500 (12000 to 15000)	36.4 (31.8 to 40.6)	3090 (2200 to 4060)	8.3 (5.9 to 11)	2 (0 to 5)	0 (0 to 0)	464 (261 to 725)	1.2 (0.7 to 1.9)	900 (117 to 2360)	2.4 (0.3 to 6.4)	633 (156 to 986)	1.7 (0.4 to 2.7)	1680 (268 to 3860)	4.5 (0.7 to 10.5)	1400 (776 to 2140)	4.4 (2.4 to 6.7)	771 (125 to 1520)	1.7 (0.3 to 3.4)
	Female	2210 (1740 to 2690)	4.3 (3.4 to 5.2)	1240 (877 to 1680)	2.4 (1.7 to 3.3)	1 (0 to 3)	0 (0 to 0)	315 (185 to 479)	0.6 (0.4 to 1)	362 (45 to 961)	0.7 (0.1 to 1.9)	259 (66 to 405)	0.5 (0.1 to 0.8)	554 (105 to 1320)	1.1 (0.2 to 2.5)	133 (64 to 214)	0.3 (0.1 to 0.4)	158 (23 to 324)	0.3 (0 to 0.6)

Moldova	Both	716 (614 to 830)	12.1 (10.4 to 14)	98 (51 to 153)	1.7 (0.9 to 2.6)	12 (4 to 25)	0.2 (0.1 to 0.4)	45 (27 to 68)	0.8 (0.5 to 1.2)	60 (4 to 187)	1 (0.1 to 3.2)	48 (14 to 75)	0.8 (0.2 to 1.3)	71 (14 to 164)	1.2 (0.2 to 2.8)	18 (10 to 29)	0.3 (0.2 to 0.5)	22 (4 to 42)	0.4 (0.1 to 0.7)
	Male	667 (569 to 773)	27 (23 to 31.1)	77 (41 to 122)	3.1 (1.6 to 4.9)	8 (3 to 19)	0.3 (0.1 to 0.8)	29 (18 to 45)	1.2 (0.7 to 1.8)	47 (4 to 147)	1.9 (0.1 to 6)	38 (11 to 59)	1.5 (0.4 to 2.4)	56 (9 to 134)	2.3 (0.4 to 5.4)	16 (8 to 26)	0.6 (0.3 to 1)	17 (3 to 34)	0.7 (0.1 to 1.3)
	Female	48 (35 to 64)	1.4 (1 to 1.9)	21 (11 to 33)	0.6 (0.3 to 1)	3 (1 to 7)	0.1 (0 to 0.2)	16 (9 to 24)	0.5 (0.3 to 0.7)	13 (1 to 41)	0.4 (0 to 1.2)	11 (3 to 17)	0.3 (0.1 to 0.5)	15 (3 to 36)	0.4 (0.1 to 1)	2 (1 to 4)	0.1 (0 to 0.1)	4 (1 to 9)	0.1 (0 to 0.3)
Romania	Both	7770 (6400 to 9530)	21.9 (18 to 27)	1210 (814 to 1680)	3.4 (2.3 to 4.8)	182 (53 to 452)	0.5 (0.1 to 1.3)	611 (344 to 965)	1.7 (1 to 2.8)	491 (74 to 1360)	1.4 (0.2 to 3.9)	405 (93 to 663)	1.1 (0.3 to 1.9)	805 (166 to 1870)	2.2 (0.4 to 5)	465 (255 to 755)	1.2 (0.7 to 2)	502 (78 to 1010)	1.5 (0.2 to 3)
	Male	6560 (5380 to 8050)	41.5 (34.1 to 50.9)	933 (625 to 1300)	5.9 (4 to 8.3)	125 (35 to 329)	0.8 (0.2 to 2.1)	446 (247 to 714)	2.8 (1.6 to 4.6)	376 (56 to 1040)	2.4 (0.4 to 6.6)	308 (69 to 509)	2 (0.4 to 3.3)	639 (101 to 1520)	4 (0.6 to 9.5)	399 (199 to 678)	2.5 (1.2 to 4.2)	410 (64 to 822)	2.6 (0.4 to 5.2)
	Female	1210 (974 to 1500)	6.1 (4.9 to 7.6)	279 (186 to 386)	1.4 (0.9 to 1.9)	57 (18 to 134)	0.3 (0.1 to 0.7)	165 (92 to 261)	0.8 (0.5 to 1.3)	115 (18 to 320)	0.6 (0.1 to 1.6)	97 (23 to 156)	0.5 (0.1 to 0.8)	166 (31 to 408)	0.8 (0.1 to 1.9)	66 (36 to 117)	0.3 (0.2 to 0.5)	92 (12 to 193)	0.5 (0.1 to 1.1)
Russia	Both	38900 (32500 to 46200)	16.3 (13.6 to 19.3)	4140 (2160 to 6530)	1.7 (0.9 to 2.7)	126 (31 to 327)	0.1 (0 to 0.1)	2440 (1400 to 3750)	1 (0.6 to 1.6)	3810 (740 to 7570)	1.6 (0.3 to 3.2)	2410 (680 to 3780)	1 (0.3 to 1.6)	2730 (546 to 6400)	1.1 (0.2 to 2.6)	3440 (2010 to 5110)	1.4 (0.8 to 2.1)	1670 (297 to 3230)	0.7 (0.1 to 1.3)
	Male	35500 (29200 to 42600)	39.1 (32.5 to 46.8)	3280 (1660 to 5250)	3.6 (1.9 to 5.8)	91 (21 to 243)	0.1 (0 to 0.3)	1590 (878 to 2500)	1.7 (1 to 2.7)	3010 (572 to 6060)	3.3 (0.6 to 6.7)	1900 (540 to 3050)	2.1 (0.6 to 3.4)	2150 (349 to 5240)	2.4 (0.4 to 5.9)	3030 (1610 to 4690)	3.5 (1.9 to 5.5)	1360 (232 to 2640)	1.3 (0.2 to 2.6)
	Female	3420 (2730 to 4230)	2.4 (1.9 to 3)	864 (458 to 1340)	0.6 (0.3 to 0.9)	34 (9 to 93)	0 (0 to 0.1)	855 (507 to 1320)	0.6 (0.4 to 0.9)	801 (159 to 1580)	0.5 (0.1 to 1.1)	515 (146 to 815)	0.4 (0.1 to 0.6)	587 (105 to 1430)	0.4 (0.1 to 0.9)	417 (230 to 656)	0.3 (0.1 to 0.4)	319 (50 to 647)	0.2 (0 to 0.5)
Rwanda	Both	248 (162 to 370)	4.8 (3.2 to 7.1)	36 (14 to 73)	0.6 (0.2 to 1.3)	134 (79 to 216)	2.4 (1.4 to 3.9)	11 (5 to 21)	0.2 (0.1 to 0.3)	21 (1 to 79)	0.4 (0 to 1.4)	1 (1 to 2)	0 (0 to 0)	27 (5 to 70)	0.5 (0.1 to 1.4)	24 (8 to 91)	0.5 (0.2 to 1.9)	28 (11 to 55)	0.4 (0.2 to 0.8)
	Male	192 (117 to 302)	9.3 (5.8	26 (9 to 57)	1.2 (0.4	85 (45 to 151)	3.9 (2 to 6.8)	7 (3 to 14)	0.3 (0.1	14 (1 to 56)	0.6 (0 to 2.5)	1 (0 to 1)	0 (0 to 0.1)	20 (3 to 56)	1 (0.2 to 2.8)	17 (4 to 83)	1 (0.3 to 4.5)	19 (7 to 41)	0.6 (0.2

			to 14.3)		to 2.5)				to 0.6)										to 1.4)
	Female	56 (40 to 75)	1.9 (1.4 to 2.6)	10 (4 to 19)	0.3 (0.1 to 0.6)	49 (32 to 70)	1.5 (1 to 2.1)	4 (2 to 7)	0.1 (0.1 to 0.2)	7 (0 to 27)	0.2 (0 to 0.8)	0 (0 to 1)	0 (0 to 0)	8 (1 to 19)	0.3 (0 to 0.6)	7 (2 to 14)	0.2 (0.1 to 0.5)	9 (3 to 16)	0.2 (0.1 to 0.4)
Saint Kitts and Nevis	Both	3 (2 to 3)	4.3 (3.6 to 5.1)	0 (0 to 1)	0.6 (0.3 to 1)	0 (0 to 0)	0 (0 to 0.1)	0 (0 to 0)	0.4 (0.2 to 0.6)	0 (0 to 1)	0.3 (0 to 1.2)	0 (0 to 1)	0.6 (0.2 to 1)	1 (0 to 2)	1.5 (0.4 to 3.2)	1 (1 to 2)	1.8 (1.2 to 2.5)	1 (0 to 1)	0.7 (0.3 to 1.1)
	Male	2 (2 to 3)	7.4 (6.3 to 8.7)	0 (0 to 0)	0.8 (0.3 to 1.4)	0 (0 to 0)	0.1 (0 to 0.1)	0 (0 to 0)	0.5 (0.3 to 0.9)	0 (0 to 1)	0.4 (0 to 1.6)	0 (0 to 0)	0.8 (0.3 to 1.3)	1 (0 to 1)	2.2 (0.4 to 4.8)	1 (1 to 1)	3.9 (2.5 to 5.5)	0 (0 to 1)	1.1 (0.5 to 1.8)
	Female	0 (0 to 1)	1.5 (1.1 to 1.9)	0 (0 to 0)	0.4 (0.2 to 0.7)	0 (0 to 0)	0 (0 to 0.1)	0 (0 to 0)	0.3 (0.1 to 0.4)	0 (0 to 0)	0.2 (0 to 0.8)	0 (0 to 0)	0.4 (0.2 to 0.7)	0 (0 to 1)	1 (0.2 to 2.3)	0 (0 to 0)	0.1 (0.1 to 0.2)	0 (0 to 0)	0.3 (0.1 to 0.6)
Saint Lucia	Both	13 (10 to 15)	5.8 (4.9 to 7)	4 (2 to 7)	1.8 (0.7 to 3.1)	0 (0 to 0)	0.1 (0 to 0.2)	1 (0 to 1)	0.3 (0.2 to 0.5)	1 (0 to 3)	0.3 (0 to 1.3)	1 (0 to 1)	0.4 (0.1 to 0.7)	4 (1 to 9)	1.9 (0.5 to 4.1)	1 (1 to 1)	0.4 (0.3 to 0.7)	2 (1 to 3)	0.8 (0.4 to 1.2)
	Male	11 (9 to 13)	10.6 (8.8 to 12.7)	3 (1 to 5)	2.6 (1 to 4.5)	0 (0 to 0)	0.1 (0 to 0.3)	0 (0 to 1)	0.5 (0.2 to 0.8)	0 (0 to 2)	0.5 (0 to 1.9)	1 (0 to 1)	0.6 (0.1 to 1)	3 (0 to 6)	2.8 (0.5 to 6.1)	1 (0 to 1)	0.8 (0.4 to 1.3)	1 (1 to 2)	1.2 (0.5 to 2)
	Female	2 (2 to 3)	1.7 (1.3 to 2.3)	1 (0 to 2)	1.1 (0.4 to 1.9)	0 (0 to 0)	0.1 (0 to 0.2)	0 (0 to 0)	0.2 (0.1 to 0.3)	0 (0 to 1)	0.2 (0 to 0.8)	0 (0 to 1)	0.3 (0.1 to 0.4)	1 (0 to 3)	1.3 (0.3 to 2.8)	0 (0 to 0)	0.2 (0.1 to 0.3)	0 (0 to 1)	0.4 (0.2 to 0.6)
Saint Vincent and the Grenadines	Both	5 (5 to 6)	4 (3.5 to 4.8)	2 (1 to 4)	1.5 (0.5 to 2.6)	0 (0 to 0)	0.1 (0 to 0.2)	0 (0 to 1)	0.3 (0.2 to 0.5)	0 (0 to 1)	0.3 (0 to 1.1)	0 (0 to 1)	0.3 (0.1 to 0.4)	2 (1 to 4)	1.5 (0.4 to 3.3)	1 (0 to 1)	0.5 (0.3 to 0.7)	1 (0 to 1)	0.6 (0.3 to 1)
	Male	5 (4 to 6)	6.7 (5.8 to 8)	1 (0 to 2)	1.9 (0.7 to 3.4)	0 (0 to 0)	0.1 (0 to 0.2)	0 (0 to 0)	0.4 (0.2 to 0.6)	0 (0 to 1)	0.4 (0 to 1.4)	0 (0 to 0)	0.3 (0.1 to 0.5)	1 (0 to 3)	1.9 (0.4 to 4.3)	1 (0 to 1)	0.9 (0.5 to 1.4)	1 (0 to 1)	0.9 (0.4 to 1.5)
	Female	1 (1 to 1)	1.2 (1 to 1.6)	1 (0 to 1)	1.1 (0.4 to 1.9)	0 (0 to 0)	0.1 (0 to 0.2)	0 (0 to 0)	0.2 (0.1 to 0.4)	0 (0 to 1)	0.2 (0 to 0.8)	0 (0 to 0)	0.2 (0 to 0.3)	1 (0 to 2)	1.1 (0.2 to 2.5)	0 (0 to 0)	0.1 (0 to 0.2)	0 (0 to 0)	0.3 (0.1 to 0.5)

Samoa	Both	11 (8 to 14)	7.9 (6 to 9.9)	1 (0 to 2)	0.6 (0.2 to 1.4)	3 (2 to 4)	2 (1.2 to 3)	1 (1 to 2)	0.9 (0.5 to 1.5)	0 (0 to 2)	0.3 (0 to 1.3)	1 (0 to 1)	0.5 (0.2 to 0.9)	2 (1 to 5)	1.6 (0.4 to 3.7)	1 (0 to 1)	0.6 (0.3 to 1)	1 (0 to 1)	0.4 (0.2 to 0.6)
	Male	9 (6 to 11)	12.7 (9.2 to 16.2)	1 (0 to 1)	0.9 (0.3 to 2.1)	2 (1 to 3)	2.5 (1.3 to 4)	1 (0 to 1)	1.2 (0.6 to 1.9)	0 (0 to 1)	0.5 (0 to 1.9)	1 (0 to 1)	0.7 (0.2 to 1.3)	1 (0 to 3)	2.3 (0.4 to 5.2)	1 (0 to 1)	1.2 (0.6 to 2.1)	0 (0 to 1)	0.6 (0.2 to 1.1)
	Female	3 (2 to 3)	3.5 (2.6 to 4.7)	0 (0 to 1)	0.3 (0.1 to 0.8)	1 (1 to 2)	1.5 (0.9 to 2.2)	1 (0 to 1)	0.7 (0.4 to 1.2)	0 (0 to 1)	0.2 (0 to 0.8)	0 (0 to 0)	0.4 (0.1 to 0.6)	1 (0 to 2)	1.1 (0.2 to 2.6)	0 (0 to 0)	0.1 (0 to 0.2)	0 (0 to 0)	0.2 (0.1 to 0.3)
San Marino	Both	14 (9 to 20)	21.7 (13.9 to 31.8)	1 (0 to 3)	2 (0.7 to 4)	0 (0 to 0)	0 (0 to 0)	1 (0 to 2)	1.6 (0.7 to 2.8)	1 (0 to 4)	1.6 (0.1 to 5.6)	1 (0 to 1)	1 (0.2 to 1.7)	2 (0 to 5)	2.9 (0.6 to 7.5)	6 (3 to 9)	8 (4.1 to 13.5)	1 (0 to 2)	1.3 (0.2 to 2.9)
	Male	10 (7 to 15)	34.8 (22.4 to 49.3)	1 (0 to 2)	3.1 (1.1 to 5.9)	0 (0 to 0)	0 (0 to 0)	1 (0 to 1)	2.3 (1.1 to 4.1)	1 (0 to 3)	2.4 (0.2 to 8.4)	0 (0 to 1)	1.4 (0.3 to 2.6)	1 (0 to 4)	4.6 (0.8 to 11.9)	5 (3 to 8)	15.8 (8.2 to 26.5)	1 (0 to 1)	2.1 (0.3 to 4.7)
	Female	3 (2 to 5)	10 (6.1 to 15.9)	0 (0 to 1)	1.1 (0.4 to 2.3)	0 (0 to 0)	0 (0 to 0)	0 (0 to 0)	0.9 (0.4 to 1.7)	0 (0 to 1)	0.9 (0.1 to 3.1)	0 (0 to 0)	0.5 (0.1 to 1.1)	1 (0 to 1)	1.4 (0.3 to 4.1)	1 (0 to 1)	1.3 (0.4 to 2.9)	0 (0 to 0)	0.5 (0.1 to 1.2)
São Tome and Príncipe	Both	5 (4 to 7)	5.8 (4.6 to 7.3)	2 (1 to 4)	2.3 (1.1 to 3.9)	3 (2 to 5)	3.4 (2 to 5)	0 (0 to 0)	0.2 (0.1 to 0.4)	1 (0 to 4)	1 (0.1 to 3.7)	0 (0 to 1)	0.4 (0.1 to 0.7)	2 (0 to 3)	1.8 (0.4 to 4)	0 (0 to 1)	0.4 (0.2 to 1)	1 (0 to 1)	0.5 (0.2 to 0.9)
	Male	5 (4 to 6)	11.3 (8.9 to 14.1)	2 (1 to 3)	3.6 (1.7 to 6.1)	2 (1 to 3)	4.9 (2.8 to 7.1)	0 (0 to 0)	0.3 (0.2 to 0.5)	1 (0 to 2)	1.5 (0.1 to 5.4)	0 (0 to 0)	0.6 (0.1 to 1)	1 (0 to 3)	2.7 (0.5 to 6.5)	0 (0 to 1)	0.8 (0.3 to 2)	0 (0 to 1)	0.8 (0.3 to 1.5)
	Female	0 (0 to 1)	1 (0.5 to 1.5)	1 (0 to 1)	1.1 (0.4 to 2.1)	1 (1 to 2)	2.2 (1 to 3.6)	0 (0 to 0)	0.1 (0.1 to 0.3)	0 (0 to 1)	0.6 (0 to 2.2)	0 (0 to 0)	0.2 (0.1 to 0.5)	0 (0 to 1)	0.9 (0.2 to 2.7)	0 (0 to 0)	0.1 (0 to 0.3)	0 (0 to 0)	0.1 (0.1 to 0.3)
Saudi Arabia	Both	792 (596 to 991)	5.1 (3.9 to 6.4)	421 (283 to 568)	2.6 (1.8 to 3.5)	1 (0 to 2)	0 (0 to 0)	117 (68 to 179)	0.7 (0.4 to 1.1)	24 (4 to 65)	0.1 (0 to 0.4)	55 (13 to 92)	0.3 (0.1 to 0.5)	184 (41 to 403)	1.4 (0.3 to 2.9)	10 (4 to 18)	0.1 (0 to 0.2)	89 (38 to 162)	0.4 (0.2 to 0.7)
	Male	750 (559 to 950)	8.1 (6.2	314 (209	3.3 (2.3	1 (0 to 1)	0 (0 to 0)	75 (42 to 117)	0.8 (0.5	18 (3 to 47)	0.2 (0 to 0.5)	40 (9 to 68)	0.4 (0.1	144 (26 to 326)	1.8 (0.3 to 4)	8 (3 to 16)	0.1 (0 to 0.2)	86 (36 to 158)	0.6 (0.3

			to 10.1)	to 429)	to 4.5)				to 1.3)				to 0.7)						to 1.1)
	Female	42 (27 to 61)	0.8 (0.5 to 1.1)	108 (70 to 151)	1.5 (1 to 2.1)	0 (0 to 1)	0 (0 to 0)	42 (24 to 66)	0.5 (0.3 to 0.8)	6 (1 to 16)	0.1 (0 to 0.2)	15 (4 to 24)	0.2 (0 to 0.3)	41 (8 to 96)	0.7 (0.2 to 1.7)	2 (1 to 4)	0 (0 to 0.1)	3 (1 to 6)	0 (0 to 0.1)
Senegal	Both	344 (256 to 472)	4.9 (3.6 to 6.7)	95 (43 to 178)	1.4 (0.6 to 2.5)	219 (132 to 332)	3.2 (1.9 to 4.8)	47 (24 to 77)	0.7 (0.4 to 1.1)	40 (3 to 150)	0.6 (0 to 2.2)	54 (19 to 88)	0.8 (0.3 to 1.3)	89 (19 to 205)	1.4 (0.3 to 3.2)	32 (14 to 61)	0.5 (0.2 to 1)	32 (13 to 60)	0.4 (0.2 to 0.7)
	Male	333 (247 to 459)	9.8 (7.3 to 13.5)	76 (35 to 147)	2.3 (1.1 to 4.4)	159 (92 to 252)	4.8 (2.8 to 7.5)	32 (17 to 55)	1 (0.5 to 1.7)	30 (2 to 111)	0.9 (0.1 to 3.3)	40 (14 to 69)	1.2 (0.4 to 2.1)	68 (12 to 168)	2.2 (0.4 to 5.4)	29 (12 to 59)	0.9 (0.4 to 1.9)	27 (11 to 52)	0.7 (0.3 to 1.3)
	Female	11 (7 to 16)	0.3 (0.2 to 0.4)	19 (8 to 36)	0.5 (0.2 to 1)	61 (39 to 92)	1.7 (1.1 to 2.5)	14 (8 to 23)	0.4 (0.2 to 0.6)	10 (1 to 36)	0.3 (0 to 1)	13 (5 to 22)	0.4 (0.1 to 0.6)	22 (4 to 52)	0.6 (0.1 to 1.5)	3 (1 to 5)	0.1 (0 to 0.2)	5 (2 to 9)	0.1 (0 to 0.2)
Serbia	Both	5580 (4370 to 6960)	34.9 (27.2 to 43.5)	1200 (800 to 1670)	7.6 (5.1 to 10.6)	202 (61 to 471)	1.3 (0.4 to 3)	489 (276 to 773)	3.1 (1.8 to 4.9)	449 (47 to 1260)	2.8 (0.3 to 7.9)	164 (33 to 270)	1.1 (0.2 to 1.8)	852 (184 to 1950)	5.1 (1.1 to 11.7)	398 (201 to 682)	2.3 (1.2 to 4)	275 (39 to 566)	1.8 (0.3 to 3.7)
	Male	4250 (3320 to 5330)	57.4 (44.6 to 71.8)	871 (593 to 1220)	11.9 (8 to 16.4)	129 (36 to 305)	1.8 (0.5 to 4.1)	349 (194 to 550)	4.8 (2.7 to 7.5)	324 (34 to 906)	4.4 (0.5 to 12.3)	114 (23 to 196)	1.6 (0.3 to 2.7)	642 (113 to 1530)	8.5 (1.5 to 20.3)	358 (171 to 629)	4.6 (2.2 to 8)	212 (30 to 440)	2.9 (0.4 to 6)
	Female	1330 (1040 to 1680)	15.7 (12.3 to 20)	323 (216 to 451)	3.9 (2.6 to 5.4)	73 (24 to 167)	0.9 (0.3 to 2)	140 (79 to 228)	1.7 (1 to 2.8)	124 (13 to 349)	1.5 (0.2 to 4.1)	49 (11 to 83)	0.6 (0.1 to 1)	210 (41 to 502)	2.3 (0.5 to 5.6)	40 (21 to 70)	0.4 (0.2 to 0.8)	62 (8 to 133)	0.8 (0.1 to 1.7)
Seychelles	Both	12 (10 to 14)	11 (9.3 to 13)	2 (1 to 4)	2 (1 to 3.3)	0 (0 to 0)	0 (0 to 0.1)	1 (1 to 2)	0.9 (0.5 to 1.5)	0 (0 to 1)	0.4 (0 to 1.3)	1 (0 to 1)	0.7 (0.2 to 1.2)	3 (1 to 6)	2.9 (0.7 to 6.2)	1 (0 to 1)	0.6 (0.3 to 1.1)	1 (1 to 2)	1 (0.5 to 1.6)
	Male	11 (9 to 13)	22.7 (19 to 26.9)	2 (1 to 3)	3.3 (1.7 to 5.5)	0 (0 to 0)	0 (0 to 0.1)	1 (0 to 1)	1.3 (0.7 to 2.1)	0 (0 to 1)	0.6 (0.1 to 2.2)	1 (0 to 1)	1.2 (0.3 to 1.9)	2 (0 to 5)	4.9 (0.9 to 10.9)	1 (0 to 1)	1.4 (0.7 to 2.8)	1 (0 to 2)	1.6 (0.7 to 2.7)
	Female	1 (1 to 1)	1.8 (1.3 to 2.4)	1 (0 to 1)	1 (0.5 to 1.6)	0 (0 to 0)	0 (0 to 0)	0 (0 to 1)	0.6 (0.3 to 0.9)	0 (0 to 0)	0.2 (0 to 0.6)	0 (0 to 0)	0.4 (0.1 to 0.6)	1 (0 to 2)	1.4 (0.3 to 3.1)	0 (0 to 0)	0.1 (0 to 0.2)	0 (0 to 0)	0.4 (0.2 to 0.7)

Sierra Leone	Both	155 (106 to 214)	4.7 (3.3 to 6.5)	26 (11 to 51)	0.8 (0.3 to 1.5)	110 (68 to 168)	3.3 (2.1 to 5.1)	11 (5 to 19)	0.3 (0.2 to 0.6)	17 (1 to 64)	0.5 (0 to 1.8)	21 (7 to 35)	0.6 (0.2 to 1.1)	17 (4 to 42)	0.6 (0.1 to 1.4)	7 (3 to 19)	0.2 (0.1 to 0.6)	15 (6 to 29)	0.4 (0.2 to 0.7)
	Male	146 (99 to 202)	9 (6.2 to 12.4)	21 (8 to 42)	1.3 (0.5 to 2.6)	81 (48 to 127)	5 (3 to 7.8)	7 (3 to 13)	0.5 (0.2 to 0.8)	13 (1 to 50)	0.8 (0 to 2.9)	16 (5 to 27)	1 (0.3 to 1.6)	13 (2 to 32)	0.8 (0.1 to 2.1)	6 (2 to 17)	0.4 (0.2 to 1.2)	12 (5 to 23)	0.6 (0.2 to 1.1)
	Female	9 (6 to 14)	0.5 (0.3 to 0.8)	5 (2 to 10)	0.3 (0.1 to 0.6)	29 (19 to 45)	1.7 (1.1 to 2.6)	4 (2 to 7)	0.2 (0.1 to 0.4)	4 (0 to 16)	0.2 (0 to 0.9)	5 (2 to 9)	0.3 (0.1 to 0.5)	5 (1 to 12)	0.3 (0.1 to 0.8)	1 (0 to 2)	0.1 (0 to 0.2)	3 (1 to 6)	0.2 (0.1 to 0.3)
Singapore	Both	831 (744 to 907)	11 (9.8 to 12.1)	209 (129 to 304)	2.8 (1.7 to 4)	0 (0 to 1)	0 (0 to 0)	39 (22 to 62)	0.5 (0.3 to 0.8)	15 (1 to 52)	0.2 (0 to 0.7)	38 (8 to 60)	0.5 (0.1 to 0.8)	154 (36 to 337)	2.1 (0.5 to 4.6)	200 (124 to 293)	2.9 (1.8 to 4.2)	58 (10 to 111)	0.7 (0.1 to 1.3)
	Male	671 (600 to 731)	19.4 (17.3 to 21.3)	135 (83 to 197)	3.9 (2.4 to 5.7)	0 (0 to 1)	0 (0 to 0)	24 (13 to 38)	0.7 (0.4 to 1)	10 (1 to 34)	0.3 (0 to 1)	24 (5 to 38)	0.7 (0.1 to 1.1)	109 (18 to 248)	3.2 (0.6 to 7.4)	182 (111 to 265)	6.1 (3.7 to 8.9)	42 (7 to 82)	1 (0.2 to 2)
	Female	160 (130 to 194)	3.9 (3.2 to 4.8)	74 (45 to 110)	1.8 (1.1 to 2.7)	0 (0 to 0)	0 (0 to 0)	16 (9 to 24)	0.4 (0.2 to 0.6)	5 (0 to 18)	0.1 (0 to 0.4)	15 (3 to 23)	0.4 (0.1 to 0.6)	45 (9 to 107)	1.1 (0.2 to 2.7)	18 (6 to 40)	0.4 (0.2 to 1)	16 (2 to 32)	0.4 (0.1 to 0.8)
Slovakia	Both	1720 (1330 to 2210)	18.2 (14.1 to 23.4)	334 (222 to 481)	3.6 (2.4 to 5.1)	3 (1 to 8)	0 (0 to 0.1)	136 (73 to 221)	1.5 (0.8 to 2.4)	211 (29 to 500)	2.3 (0.3 to 5.3)	118 (36 to 195)	1.3 (0.4 to 2.1)	219 (43 to 520)	2.3 (0.5 to 5.4)	115 (59 to 206)	1.2 (0.6 to 2.1)	115 (17 to 236)	1.2 (0.2 to 2.5)
	Male	1440 (1100 to 1880)	36 (27.5 to 46.9)	254 (168 to 367)	6.4 (4.2 to 9.2)	2 (0 to 6)	0.1 (0 to 0.1)	100 (53 to 168)	2.5 (1.3 to 4.2)	160 (22 to 382)	4 (0.6 to 9.6)	89 (27 to 149)	2.2 (0.7 to 3.7)	172 (29 to 435)	4.3 (0.7 to 10.9)	104 (49 to 190)	2.6 (1.3 to 4.8)	96 (14 to 199)	2.2 (0.3 to 4.6)
	Female	278 (212 to 352)	5.3 (4.1 to 6.8)	80 (53 to 115)	1.5 (1 to 2.2)	1 (0 to 3)	0 (0 to 0)	35 (19 to 58)	0.7 (0.4 to 1.1)	51 (8 to 117)	1 (0.1 to 2.2)	29 (9 to 47)	0.5 (0.2 to 0.9)	47 (9 to 118)	0.8 (0.2 to 2.1)	11 (6 to 20)	0.2 (0.1 to 0.3)	19 (2 to 41)	0.4 (0 to 0.8)
Slovenia	Both	909 (705 to 1180)	21.6 (16.7 to 28.2)	154 (100 to 223)	3.6 (2.3 to 5.3)	11 (2 to 29)	0.3 (0.1 to 0.7)	79 (45 to 126)	1.9 (1.1 to 3.1)	94 (12 to 232)	2.2 (0.3 to 5.5)	35 (8 to 59)	0.8 (0.2 to 1.4)	115 (25 to 267)	2.6 (0.5 to 6.1)	258 (144 to 412)	5.7 (3.2 to 9.3)	48 (7 to 99)	1.2 (0.2 to 2.5)
	Male	679 (523 to 883)	35.5 (27.3	108 (70 to 156)	5.7 (3.7	7 (1 to 18)	0.3 (0.1 to 1)	57 (32 to 91)	3 (1.7 to 4.9)	66 (9 to 163)	3.5 (0.5	24 (5 to 41)	1.3 (0.3	85 (15 to 204)	4.4 (0.8	234 (128	12 (6.6	36 (5 to 75)	1.9 (0.3

			to 46.3)		to 8.2)						to 8.6)		to 2.2)		to 10.6)	to 376)	to 19.3)		to 3.9)
	Female	230 (173 to 301)	10.3 (7.7 to 13.4)	46 (29 to 68)	2 (1.3 to 3)	4 (1 to 11)	0.2 (0 to 0.5)	22 (13 to 36)	1 (0.6 to 1.6)	28 (4 to 69)	1.2 (0.2 to 3)	11 (3 to 19)	0.5 (0.1 to 0.8)	30 (6 to 72)	1.2 (0.2 to 2.9)	24 (10 to 43)	0.9 (0.4 to 1.6)	12 (1 to 25)	0.6 (0.1 to 1.3)
Solomon Islands	Both	61 (32 to 101)	22 (12.6 to 35.2)	3 (1 to 9)	1 (0.2 to 2.9)	30 (14 to 52)	10.1 (5.2 to 17.1)	5 (2 to 11)	1.9 (0.8 to 3.7)	3 (0 to 13)	1.1 (0.1 to 4.5)	5 (1 to 10)	1.7 (0.5 to 3.4)	13 (2 to 34)	4.7 (0.9 to 12.1)	6 (2 to 12)	2.6 (1.2 to 4.9)	7 (2 to 15)	1.9 (0.6 to 3.9)
	Male	54 (27 to 94)	38.4 (21.2 to 64)	3 (1 to 8)	1.7 (0.4 to 5.1)	24 (11 to 46)	16 (7.8 to 29.4)	4 (2 to 9)	2.8 (1.1 to 5.9)	3 (0 to 11)	1.7 (0.1 to 7.4)	4 (1 to 9)	2.7 (0.7 to 5.6)	11 (2 to 30)	7.8 (1.4 to 21.6)	6 (2 to 12)	4.8 (2.1 to 9.2)	6 (2 to 13)	2.9 (0.9 to 6.5)
	Female	7 (4 to 10)	4.9 (3.2 to 7.2)	0 (0 to 1)	0.3 (0.1 to 0.8)	6 (3 to 9)	4 (2.4 to 6.3)	1 (1 to 3)	0.9 (0.4 to 1.6)	1 (0 to 2)	0.4 (0 to 1.7)	1 (0 to 2)	0.7 (0.2 to 1.2)	2 (0 to 5)	1.4 (0.3 to 3.3)	0 (0 to 1)	0.3 (0.1 to 0.7)	2 (1 to 3)	0.8 (0.3 to 1.6)
Somalia	Both	137 (73 to 240)	2.3 (1.2 to 4)	5 (1 to 17)	0.1 (0 to 0.3)	154 (69 to 329)	2.4 (1.1 to 5.1)	8 (3 to 16)	0.1 (0 to 0.2)	12 (1 to 53)	0.2 (0 to 0.8)	23 (7 to 45)	0.4 (0.1 to 0.7)	22 (4 to 61)	0.4 (0.1 to 1.1)	12 (2 to 58)	0.2 (0 to 1.1)	33 (11 to 69)	0.4 (0.1 to 0.9)
	Male	131 (69 to 230)	5.6 (3 to 9.6)	4 (1 to 15)	0.2 (0 to 0.6)	121 (53 to 269)	4.8 (2.1 to 10.5)	6 (2 to 13)	0.2 (0.1 to 0.5)	10 (1 to 42)	0.4 (0 to 1.7)	18 (5 to 38)	0.7 (0.2 to 1.5)	18 (2 to 51)	0.8 (0.1 to 2.3)	10 (1 to 55)	0.5 (0.1 to 2.9)	27 (9 to 60)	0.8 (0.3 to 1.7)
	Female	6 (2 to 11)	0.2 (0.1 to 0.3)	1 (0 to 2)	0 (0 to 0.1)	33 (14 to 79)	0.9 (0.4 to 2.2)	2 (1 to 4)	0.1 (0 to 0.1)	2 (0 to 10)	0.1 (0 to 0.3)	5 (1 to 10)	0.1 (0 to 0.3)	4 (1 to 11)	0.1 (0 to 0.3)	2 (0 to 6)	0.1 (0 to 0.2)	5 (2 to 12)	0.1 (0 to 0.3)
South Africa	Both	4800 (4210 to 5540)	11 (9.7 to 12.6)	1430 (1000 to 1980)	3.3 (2.3 to 4.5)	172 (80 to 317)	0.4 (0.2 to 0.7)	320 (164 to 519)	0.7 (0.4 to 1.2)	401 (68 to 984)	0.9 (0.2 to 2.3)	497 (171 to 781)	1.1 (0.4 to 1.8)	825 (194 to 1800)	2 (0.5 to 4.3)	1460 (998 to 2010)	3.6 (2.5 to 4.9)	168 (81 to 276)	0.3 (0.2 to 0.5)
	Male	3720 (3210 to 4350)	20.8 (18.1 to 24)	971 (672 to 1370)	5.4 (3.7 to 7.6)	109 (47 to 207)	0.6 (0.3 to 1.1)	190 (97 to 314)	1.1 (0.5 to 1.7)	270 (45 to 673)	1.5 (0.3 to 3.7)	335 (114 to 541)	1.8 (0.6 to 3)	522 (89 to 1210)	3.1 (0.5 to 7.1)	1280 (827 to 1780)	8.2 (5.5 to 11.4)	132 (60 to 222)	0.6 (0.3 to 1)
	Female	1080 (914 to 1260)	4.4 (3.7 to 5)	458 (314 to 627)	1.8 (1.3 to 2.5)	63 (31 to 112)	0.3 (0.1 to 0.5)	129 (68 to 207)	0.5 (0.3 to 0.8)	131 (22 to 311)	0.5 (0.1 to 1.3)	162 (58 to 247)	0.6 (0.2 to 1)	302 (60 to 693)	1.2 (0.2 to 2.8)	189 (80 to 284)	0.8 (0.3 to 1.2)	37 (16 to 62)	0.1 (0.1 to 0.2)

South Sudan	Both	123 (74 to 181)	3.8 (2.3 to 5.6)	21 (7 to 42)	0.6 (0.2 to 1.2)	97 (55 to 153)	2.8 (1.6 to 4.4)	8 (3 to 14)	0.2 (0.1 to 0.4)	11 (1 to 47)	0.3 (0 to 1.4)	13 (3 to 25)	0.4 (0.1 to 0.7)	20 (4 to 51)	0.6 (0.1 to 1.6)	9 (2 to 52)	0.3 (0.1 to 1.8)	17 (7 to 33)	0.4 (0.2 to 0.8)
	Male	118 (69 to 176)	6.8 (4 to 10.1)	18 (6 to 36)	1 (0.3 to 2)	74 (40 to 121)	4.1 (2.3 to 6.5)	5 (2 to 10)	0.3 (0.1 to 0.5)	9 (1 to 36)	0.5 (0 to 2)	10 (2 to 19)	0.6 (0.1 to 1)	17 (2 to 43)	1 (0.2 to 2.6)	7 (1 to 50)	0.5 (0.1 to 3.2)	13 (5 to 26)	0.6 (0.2 to 1.1)
	Female	5 (3 to 10)	0.4 (0.2 to 0.7)	4 (1 to 8)	0.2 (0.1 to 0.5)	23 (13 to 42)	1.3 (0.8 to 2.5)	2 (1 to 4)	0.1 (0 to 0.2)	2 (0 to 10)	0.1 (0 to 0.6)	3 (1 to 6)	0.2 (0 to 0.3)	3 (1 to 9)	0.2 (0 to 0.6)	2 (1 to 5)	0.2 (0 to 0.4)	4 (1 to 9)	0.2 (0.1 to 0.4)
Spain	Both	18400 (17000 to 19600)	20.3 (18.9 to 21.6)	1500 (894 to 2240)	1.6 (1 to 2.5)	27 (7 to 75)	0 (0 to 0.1)	1080 (630 to 1640)	1.3 (0.7 to 1.9)	1690 (258 to 3910)	1.8 (0.3 to 4.2)	446 (108 to 720)	0.5 (0.1 to 0.8)	2770 (573 to 6110)	2.9 (0.6 to 6.4)	4700 (2960 to 6590)	4.6 (2.9 to 6.5)	818 (149 to 1580)	1 (0.2 to 1.9)
	Male	15600 (14400 to 16700)	37.5 (34.6 to 39.9)	1190 (714 to 1780)	2.9 (1.7 to 4.3)	19 (5 to 53)	0 (0 to 0.1)	837 (486 to 1280)	2.1 (1.2 to 3.2)	1340 (203 to 3110)	3.2 (0.5 to 7.5)	342 (79 to 579)	0.8 (0.2 to 1.4)	2290 (402 to 5290)	5.4 (0.9 to 12.4)	4480 (2770 to 6390)	10.1 (6.2 to 14.4)	703 (124 to 1360)	1.8 (0.3 to 3.4)
	Female	2750 (2390 to 3080)	6.3 (5.5 to 7)	310 (183 to 467)	0.6 (0.4 to 1)	8 (2 to 21)	0 (0 to 0)	238 (136 to 358)	0.5 (0.3 to 0.8)	349 (54 to 806)	0.7 (0.1 to 1.7)	104 (26 to 169)	0.2 (0.1 to 0.3)	480 (90 to 1110)	0.9 (0.2 to 2)	223 (116 to 352)	0.4 (0.2 to 0.6)	115 (18 to 234)	0.3 (0 to 0.6)
Sri Lanka	Both	1070 (748 to 1490)	4.2 (3 to 5.8)	289 (159 to 462)	1.1 (0.6 to 1.8)	249 (110 to 451)	1 (0.4 to 1.8)	76 (35 to 138)	0.3 (0.1 to 0.5)	52 (4 to 197)	0.2 (0 to 0.8)	135 (44 to 232)	0.5 (0.2 to 0.9)	375 (87 to 862)	1.5 (0.4 to 3.5)	103 (49 to 193)	0.5 (0.2 to 0.8)	153 (65 to 277)	0.5 (0.2 to 1)
	Male	996 (699 to 1390)	9 (6.4 to 12.4)	205 (113 to 330)	1.8 (1 to 2.9)	154 (63 to 300)	1.4 (0.6 to 2.7)	43 (20 to 79)	0.4 (0.2 to 0.7)	35 (3 to 135)	0.3 (0 to 1.2)	92 (30 to 161)	0.8 (0.3 to 1.4)	255 (44 to 630)	2.4 (0.4 to 5.7)	91 (40 to 170)	1 (0.5 to 1.8)	127 (52 to 235)	1 (0.4 to 1.8)
	Female	77 (50 to 111)	0.6 (0.4 to 0.8)	84 (46 to 135)	0.6 (0.3 to 1)	95 (44 to 164)	0.7 (0.3 to 1.2)	33 (15 to 59)	0.2 (0.1 to 0.4)	16 (1 to 64)	0.1 (0 to 0.5)	42 (14 to 72)	0.3 (0.1 to 0.5)	120 (27 to 269)	0.9 (0.2 to 1.9)	13 (5 to 26)	0.1 (0 to 0.2)	26 (10 to 49)	0.2 (0.1 to 0.3)
Sudan	Both	878 (536 to 1500)	5.2 (3.2 to 8.8)	285 (137 to 510)	1.6 (0.8 to 2.9)	175 (83 to 327)	1 (0.5 to 1.9)	118 (61 to 213)	0.7 (0.3 to 1.2)	43 (3 to 171)	0.2 (0 to 1)	64 (15 to 117)	0.4 (0.1 to 0.7)	172 (34 to 446)	1.1 (0.2 to 2.7)	11 (2 to 51)	0.1 (0 to 0.3)	97 (36 to 205)	0.5 (0.2 to 1)
	Male	826 (496 to 1440)	8.9 (5.4	221 (104	2.3 (1.1	122 (51 to 257)	1.3 (0.5	84 (40 to 168)	0.9 (0.4	32 (2 to 134)	0.3 (0 to 1.4)	47 (10 to 95)	0.5 (0.1 to 1)	133 (21 to 369)	1.5 (0.2	9 (1 to 49)	0.1 (0 to 0.6)	88 (32 to 190)	0.8 (0.3

			to 15.3)	to 430)	to 4.5)		to 2.7)		to 1.8)						to 4.2)				to 1.7)
	Female	52 (33 to 77)	0.7 (0.4 to 1)	64 (33 to 105)	0.8 (0.4 to 1.2)	54 (29 to 85)	0.6 (0.4 to 1)	34 (18 to 55)	0.4 (0.2 to 0.6)	11 (1 to 38)	0.1 (0 to 0.5)	17 (5 to 29)	0.2 (0.1 to 0.3)	39 (8 to 94)	0.5 (0.1 to 1.3)	2 (1 to 5)	0 (0 to 0.1)	9 (3 to 18)	0.1 (0 to 0.2)
Suriname	Both	53 (43 to 65)	8.9 (7.2 to 10.9)	13 (5 to 22)	2.1 (0.9 to 3.7)	2 (1 to 4)	0.4 (0.1 to 0.7)	5 (3 to 7)	0.8 (0.4 to 1.2)	2 (0 to 8)	0.4 (0 to 1.4)	4 (1 to 6)	0.6 (0.2 to 1)	13 (3 to 28)	2.3 (0.6 to 4.8)	3 (2 to 5)	0.5 (0.3 to 0.9)	6 (3 to 11)	1 (0.5 to 1.6)
	Male	42 (34 to 52)	15.6 (12.7 to 19.3)	8 (3 to 15)	3 (1.3 to 5.4)	1 (0 to 3)	0.4 (0.2 to 0.9)	3 (1 to 4)	1 (0.5 to 1.6)	2 (0 to 5)	0.6 (0 to 2)	2 (1 to 4)	0.8 (0.2 to 1.4)	9 (2 to 20)	3.4 (0.6 to 7.7)	2 (1 to 4)	1 (0.5 to 1.7)	5 (2 to 9)	1.7 (0.7 to 2.9)
	Female	11 (8 to 14)	3.4 (2.6 to 4.4)	4 (2 to 8)	1.3 (0.5 to 2.4)	1 (0 to 2)	0.3 (0.1 to 0.6)	2 (1 to 3)	0.6 (0.3 to 0.9)	1 (0 to 3)	0.3 (0 to 1)	1 (0 to 2)	0.4 (0.1 to 0.7)	5 (1 to 11)	1.4 (0.3 to 3.4)	1 (0 to 1)	0.2 (0.1 to 0.4)	1 (1 to 2)	0.4 (0.2 to 0.7)
Sweden	Both	2930 (2700 to 3150)	13.4 (12.4 to 14.3)	85 (20 to 184)	0.4 (0.1 to 0.9)	0 (0 to 1)	0 (0 to 0)	122 (72 to 184)	0.6 (0.4 to 0.9)	276 (36 to 726)	1.3 (0.2 to 3.4)	157 (39 to 238)	0.7 (0.2 to 1.1)	400 (97 to 862)	1.8 (0.4 to 3.8)	937 (664 to 1200)	3.9 (2.7 to 5)	138 (24 to 268)	0.7 (0.1 to 1.4)
	Male	1470 (1360 to 1580)	14.1 (13 to 15.2)	42 (10 to 91)	0.4 (0.1 to 0.9)	0 (0 to 1)	0 (0 to 0)	76 (45 to 118)	0.8 (0.5 to 1.2)	137 (18 to 366)	1.3 (0.2 to 3.6)	77 (19 to 117)	0.7 (0.2 to 1.1)	216 (36 to 492)	2 (0.3 to 4.6)	793 (538 to 1040)	7.1 (4.8 to 9.4)	83 (14 to 160)	0.9 (0.1 to 1.7)
	Female	1460 (1310 to 1600)	12.9 (11.7 to 14)	43 (10 to 93)	0.4 (0.1 to 0.8)	0 (0 to 1)	0 (0 to 0)	45 (26 to 68)	0.5 (0.3 to 0.7)	139 (18 to 358)	1.2 (0.2 to 3.2)	80 (19 to 123)	0.7 (0.2 to 1.1)	185 (38 to 431)	1.5 (0.3 to 3.6)	144 (81 to 217)	1.1 (0.6 to 1.7)	56 (8 to 112)	0.6 (0.1 to 1.1)
Switzerland	Both	2720 (2460 to 2930)	15.7 (14.3 to 16.9)	240 (146 to 361)	1.4 (0.8 to 2.1)	0 (0 to 1)	0 (0 to 0)	136 (79 to 206)	0.8 (0.5 to 1.3)	262 (40 to 613)	1.5 (0.2 to 3.6)	137 (33 to 206)	0.8 (0.2 to 1.2)	354 (84 to 778)	2 (0.5 to 4.3)	1090 (781 to 1390)	5.9 (4.1 to 7.5)	151 (25 to 293)	1 (0.2 to 1.9)
	Male	1780 (1610 to 1940)	22.2 (20.1 to 24.2)	149 (89 to 221)	1.9 (1.1 to 2.8)	0 (0 to 0)	0 (0 to 0)	86 (49 to 133)	1.1 (0.6 to 1.7)	162 (25 to 383)	2 (0.3 to 4.8)	84 (20 to 127)	1.1 (0.3 to 1.6)	232 (39 to 545)	2.8 (0.5 to 6.7)	987 (688 to 1280)	11.9 (8.3 to 15.4)	107 (16 to 209)	1.4 (0.2 to 2.7)
	Female	937 (832 to 1040)	10.3 (9.2 to 11.4)	92 (55 to 136)	1 (0.6 to 1.5)	0 (0 to 0)	0 (0 to 0)	50 (29 to 77)	0.6 (0.3 to 0.9)	100 (15 to 232)	1.1 (0.2 to 2.5)	53 (13 to 82)	0.6 (0.1 to 0.9)	122 (23 to 285)	1.3 (0.2 to 2.9)	102 (55 to 154)	1 (0.5 to 1.5)	45 (7 to 90)	0.6 (0.1 to 1.1)

Syria	Both	865 (628 to 1140)	7.3 (5.4 to 9.5)	274 (180 to 410)	2.3 (1.6 to 3.4)	0 (0 to 1)	0 (0 to 0)	93 (51 to 151)	0.8 (0.4 to 1.2)	46 (7 to 123)	0.4 (0.1 to 1)	42 (10 to 71)	0.4 (0.1 to 0.6)	146 (32 to 334)	1.3 (0.3 to 3)	14 (6 to 29)	0.2 (0.1 to 0.3)	87 (37 to 161)	0.6 (0.3 to 1.1)
	Male	786 (568 to 1040)	12.7 (9.3 to 16.5)	202 (131 to 299)	3.2 (2.1 to 4.7)	0 (0 to 1)	0 (0 to 0)	62 (33 to 100)	1 (0.5 to 1.6)	34 (5 to 90)	0.5 (0.1 to 1.4)	30 (7 to 52)	0.5 (0.1 to 0.8)	108 (18 to 262)	1.8 (0.3 to 4.4)	13 (6 to 27)	0.3 (0.1 to 0.5)	84 (36 to 156)	1.1 (0.5 to 2.1)
	Female	79 (51 to 112)	1.5 (1 to 2)	73 (46 to 109)	1.4 (0.9 to 2)	0 (0 to 0)	0 (0 to 0)	32 (17 to 51)	0.5 (0.3 to 0.8)	12 (2 to 33)	0.2 (0 to 0.6)	12 (3 to 20)	0.2 (0.1 to 0.4)	38 (8 to 89)	0.8 (0.2 to 1.8)	1 (0 to 2)	0 (0 to 0.1)	3 (1 to 6)	0 (0 to 0.1)
Taiwan (Province of China)	Both	6430 (5050 to 8210)	16.1 (12.6 to 20.6)	1940 (1340 to 2760)	4.9 (3.4 to 6.9)	82 (18 to 227)	0.2 (0 to 0.6)	795 (448 to 1250)	2 (1.1 to 3.2)	125 (23 to 344)	0.3 (0.1 to 0.9)	284 (67 to 466)	0.7 (0.2 to 1.2)	1100 (253 to 2490)	2.8 (0.6 to 6.2)	487 (243 to 869)	1.2 (0.6 to 2.2)	667 (317 to 1130)	1.6 (0.8 to 2.8)
	Male	5840 (4580 to 7510)	31.8 (25 to 40.8)	1220 (836 to 1720)	6.7 (4.6 to 9.4)	43 (9 to 119)	0.2 (0 to 0.7)	421 (232 to 690)	2.3 (1.3 to 3.8)	78 (14 to 217)	0.4 (0.1 to 1.2)	168 (37 to 279)	0.9 (0.2 to 1.5)	690 (117 to 1660)	3.8 (0.6 to 9.1)	370 (173 to 673)	2.1 (1 to 3.8)	443 (195 to 775)	2.3 (1 to 4)
	Female	596 (425 to 821)	2.8 (2 to 3.9)	727 (495 to 1030)	3.4 (2.3 to 4.8)	39 (9 to 110)	0.2 (0 to 0.5)	374 (213 to 594)	1.8 (1 to 2.8)	47 (8 to 128)	0.2 (0 to 0.6)	116 (25 to 198)	0.5 (0.1 to 0.9)	410 (80 to 967)	1.9 (0.4 to 4.4)	117 (37 to 276)	0.5 (0.2 to 1.3)	224 (100 to 393)	1.1 (0.5 to 1.8)
Tajikistan	Both	306 (242 to 392)	6.7 (5.4 to 8.5)	98 (49 to 159)	2.1 (1.1 to 3.4)	49 (24 to 84)	1.1 (0.5 to 1.8)	33 (19 to 51)	0.7 (0.4 to 1.1)	34 (3 to 116)	0.7 (0.1 to 2.5)	30 (10 to 50)	0.7 (0.2 to 1.1)	45 (10 to 106)	1.1 (0.2 to 2.5)	13 (6 to 23)	0.3 (0.2 to 0.6)	29 (13 to 50)	0.4 (0.2 to 0.8)
	Male	288 (226 to 370)	13.6 (10.9 to 17.2)	71 (36 to 114)	3.3 (1.7 to 5.2)	31 (15 to 56)	1.5 (0.7 to 2.6)	21 (12 to 34)	1 (0.6 to 1.6)	24 (2 to 83)	1.1 (0.1 to 3.8)	21 (7 to 35)	1 (0.3 to 1.6)	32 (5 to 78)	1.6 (0.3 to 3.9)	10 (5 to 20)	0.6 (0.3 to 1.1)	24 (11 to 43)	0.8 (0.3 to 1.4)
	Female	18 (13 to 26)	0.7 (0.5 to 1)	27 (12 to 46)	1.1 (0.5 to 1.8)	18 (9 to 30)	0.7 (0.4 to 1.2)	11 (7 to 18)	0.4 (0.2 to 0.6)	10 (1 to 36)	0.4 (0 to 1.4)	9 (3 to 15)	0.4 (0.1 to 0.6)	13 (3 to 32)	0.6 (0.1 to 1.4)	2 (1 to 4)	0.1 (0 to 0.2)	5 (2 to 9)	0.1 (0.1 to 0.3)
Thailand	Both	12500 (9320 to 16400)	12.4 (9.2 to 16.2)	3870 (2570 to 5630)	3.8 (2.6 to 5.6)	868 (324 to 1830)	0.9 (0.3 to 1.8)	978 (513 to 1660)	1 (0.5 to 1.6)	397 (46 to 1110)	0.4 (0 to 1.1)	579 (138 to 948)	0.6 (0.1 to 0.9)	2040 (450 to 4830)	2.1 (0.5 to 4.8)	835 (379 to 1540)	0.9 (0.4 to 1.6)	1260 (575 to 2180)	1.2 (0.5 to 2)
	Male	11000 (8100	24.3 (18.1	2550 (1680	5.6 (3.7	487 (170	1.1 (0.4	488 (251	1.1 (0.6	257 (30 to 718)	0.6 (0.1	361 (82 to 618)	0.8 (0.2	1330 (220	3 (0.5 to 7.5)	801 (354	2 (0.9 to 3.6)	856 (363	1.7 (0.7 to 3)

		to 14400)	to 31.8)	to 3720)	to 8.2)	to 1060)	to 2.4)	to 851)	to 1.9)		to 1.6)		to 1.4)	to 3280)		to 1490)		to 1540)	
	Female	1580 (1140 to 2080)	2.8 (2.1 to 3.7)	1320 (862 to 1910)	2.4 (1.6 to 3.4)	382 (150 to 779)	0.7 (0.3 to 1.4)	490 (257 to 819)	0.9 (0.5 to 1.5)	140 (16 to 397)	0.3 (0 to 0.7)	219 (52 to 364)	0.4 (0.1 to 0.7)	716 (141 to 1720)	1.3 (0.3 to 3.1)	33 (10 to 79)	0.1 (0 to 0.1)	404 (168 to 731)	0.7 (0.3 to 1.3)
Timor-Leste	Both	93 (62 to 122)	11.9 (8.1 to 15.5)	9 (4 to 17)	1.1 (0.5 to 2.1)	40 (24 to 58)	5 (3.1 to 7.4)	7 (3 to 12)	0.9 (0.4 to 1.5)	4 (0 to 13)	0.4 (0 to 1.7)	10 (3 to 18)	1.3 (0.4 to 2.2)	15 (3 to 34)	2 (0.4 to 4.6)	4 (2 to 8)	0.7 (0.3 to 1.2)	10 (4 to 18)	1.1 (0.4 to 2)
	Male	87 (58 to 114)	22.6 (14.8 to 29.4)	7 (3 to 13)	1.8 (0.7 to 3.4)	27 (16 to 41)	7 (4.1 to 10.5)	4 (2 to 7)	1.1 (0.5 to 1.9)	3 (0 to 10)	0.7 (0.1 to 2.4)	7 (2 to 12)	1.9 (0.6 to 3.2)	11 (2 to 26)	3 (0.5 to 7)	4 (2 to 7)	1.2 (0.5 to 2.3)	7 (3 to 14)	1.6 (0.6 to 3)
	Female	6 (3 to 9)	1.5 (0.9 to 2.4)	2 (1 to 4)	0.5 (0.2 to 1)	12 (8 to 19)	3.1 (1.9 to 4.7)	3 (1 to 5)	0.7 (0.3 to 1.3)	1 (0 to 4)	0.3 (0 to 0.9)	3 (1 to 5)	0.7 (0.3 to 1.3)	4 (1 to 9)	1 (0.2 to 2.5)	0 (0 to 1)	0.1 (0 to 0.3)	3 (1 to 5)	0.6 (0.2 to 1.1)
Togo	Both	176 (125 to 241)	5.4 (3.9 to 7.3)	37 (17 to 67)	1.1 (0.5 to 2)	100 (62 to 150)	3 (1.9 to 4.5)	13 (7 to 22)	0.4 (0.2 to 0.6)	18 (1 to 67)	0.5 (0 to 2)	26 (9 to 44)	0.8 (0.3 to 1.3)	20 (4 to 48)	0.7 (0.1 to 1.6)	10 (5 to 21)	0.4 (0.2 to 0.7)	18 (8 to 34)	0.4 (0.2 to 0.8)
	Male	156 (109 to 215)	12 (8.6 to 16.2)	28 (12 to 51)	2 (0.9 to 3.7)	67 (39 to 108)	4.9 (2.9 to 7.8)	9 (4 to 15)	0.6 (0.3 to 1)	13 (1 to 46)	0.9 (0.1 to 3.4)	18 (6 to 31)	1.3 (0.5 to 2.3)	14 (2 to 37)	1.3 (0.2 to 3.1)	9 (4 to 19)	0.8 (0.4 to 1.6)	14 (5 to 26)	0.7 (0.3 to 1.3)
	Female	20 (13 to 28)	1.1 (0.8 to 1.6)	9 (4 to 16)	0.5 (0.2 to 0.8)	33 (21 to 47)	1.7 (1.1 to 2.4)	4 (2 to 7)	0.2 (0.1 to 0.3)	6 (0 to 20)	0.3 (0 to 1)	8 (3 to 13)	0.4 (0.1 to 0.7)	6 (1 to 15)	0.3 (0.1 to 0.8)	1 (0 to 3)	0.1 (0 to 0.2)	5 (2 to 9)	0.2 (0.1 to 0.4)
Tokelau	Both	0 (0 to 0)	14.2 (10.9 to 18.7)	0 (0 to 0)	0.8 (0 to 2.3)	0 (0 to 0)	0 (0 to 0)	0 (0 to 0)	1.7 (0.9 to 2.7)	0 (0 to 0)	1.7 (0.1 to 5.8)	0 (0 to 0)	1.1 (0.3 to 1.9)	0 (0 to 0)	3.6 (0.9 to 8)	0 (0 to 0)	1.3 (0.7 to 2.3)	0 (0 to 0)	1 (0.4 to 1.8)
	Male	0 (0 to 0)	22 (16.5 to 30.1)	0 (0 to 0)	1 (0 to 3)	0 (0 to 0)	0 (0 to 0)	0 (0 to 0)	1.9 (1 to 3.3)	0 (0 to 0)	2.2 (0.1 to 7)	0 (0 to 0)	1.5 (0.4 to 2.6)	0 (0 to 0)	4.6 (0.8 to 11)	0 (0 to 0)	2.3 (1.2 to 4.3)	0 (0 to 0)	1.3 (0.5 to 2.5)
	Female	0 (0 to 0)	6.6 (4.1 to 9.8)	0 (0 to 0)	0.6 (0 to 1.7)	0 (0 to 0)	0 (0 to 0)	0 (0 to 0)	1.4 (0.7 to 2.5)	0 (0 to 0)	1.2 (0.1 to 4.2)	0 (0 to 0)	0.8 (0.2 to 1.5)	0 (0 to 0)	2.7 (0.6 to 6.8)	0 (0 to 0)	0.3 (0.1 to 0.6)	0 (0 to 0)	0.6 (0.2 to 1.2)

Tonga	Both	15 (12 to 18)	19.4 (16 to 23.2)	1 (0 to 3)	1.8 (0.6 to 4.2)	2 (1 to 3)	2.5 (1.2 to 4.3)	1 (1 to 2)	1.8 (1 to 2.9)	1 (0 to 3)	1 (0 to 3.9)	1 (0 to 2)	1.4 (0.4 to 2.2)	3 (1 to 7)	4.2 (0.9 to 9.2)	1 (1 to 2)	1.7 (0.9 to 2.8)	1 (0 to 1)	0.8 (0.4 to 1.5)
	Male	13 (11 to 16)	38.3 (31.3 to 46)	1 (0 to 3)	3.2 (1 to 7.4)	1 (1 to 2)	4 (1.7 to 7.1)	1 (1 to 2)	2.8 (1.5 to 4.6)	1 (0 to 2)	1.6 (0.1 to 6.6)	1 (0 to 1)	2.3 (0.7 to 3.8)	2 (0 to 6)	7.2 (1.4 to 16.3)	1 (1 to 2)	4 (2.2 to 6.5)	0 (0 to 1)	1.3 (0.5 to 2.3)
	Female	2 (1 to 2)	4.2 (3.1 to 5.7)	0 (0 to 1)	0.7 (0.2 to 1.7)	1 (0 to 1)	1.4 (0.7 to 2.3)	0 (0 to 1)	1 (0.5 to 1.6)	0 (0 to 1)	0.4 (0 to 1.7)	0 (0 to 0)	0.6 (0.2 to 1)	1 (0 to 2)	1.8 (0.4 to 4.2)	0 (0 to 0)	0.1 (0.1 to 0.3)	0 (0 to 0)	0.5 (0.2 to 0.9)
Trinidad and Tobago	Both	100 (74 to 131)	5.3 (4 to 6.9)	28 (10 to 53)	1.5 (0.5 to 2.9)	0 (0 to 0)	0 (0 to 0)	7 (4 to 12)	0.4 (0.2 to 0.7)	5 (0 to 19)	0.3 (0 to 1)	9 (3 to 16)	0.5 (0.2 to 0.8)	31 (7 to 69)	1.6 (0.4 to 3.7)	6 (3 to 12)	0.4 (0.2 to 0.6)	15 (7 to 26)	0.7 (0.3 to 1.3)
	Male	87 (64 to 114)	9.7 (7.1 to 12.6)	21 (7 to 40)	2.3 (0.8 to 4.5)	0 (0 to 0)	0 (0 to 0)	5 (2 to 9)	0.6 (0.3 to 0.9)	4 (0 to 15)	0.4 (0 to 1.6)	7 (2 to 12)	0.8 (0.2 to 1.3)	23 (4 to 54)	2.6 (0.5 to 6)	6 (3 to 11)	0.7 (0.3 to 1.3)	12 (5 to 22)	1.3 (0.5 to 2.3)
	Female	13 (9 to 18)	1.3 (0.9 to 1.9)	7 (3 to 14)	0.8 (0.3 to 1.4)	0 (0 to 0)	0 (0 to 0)	2 (1 to 4)	0.2 (0.1 to 0.4)	1 (0 to 5)	0.1 (0 to 0.5)	2 (1 to 4)	0.3 (0.1 to 0.4)	7 (2 to 18)	0.8 (0.2 to 1.8)	1 (0 to 1)	0.1 (0 to 0.1)	2 (1 to 4)	0.2 (0.1 to 0.4)
Tunisia	Both	1900 (1330 to 2640)	15.2 (10.7 to 21.1)	500 (308 to 761)	4 (2.5 to 6.1)	1 (0 to 3)	0 (0 to 0)	183 (94 to 301)	1.5 (0.8 to 2.4)	124 (16 to 327)	1 (0.1 to 2.6)	60 (13 to 107)	0.5 (0.1 to 0.9)	359 (67 to 864)	2.9 (0.6 to 7)	22 (3 to 106)	0.2 (0 to 0.9)	156 (64 to 289)	1.1 (0.5 to 2.1)
	Male	1860 (1300 to 2590)	30.8 (21.8 to 42.8)	449 (273 to 692)	7.4 (4.5 to 11.4)	1 (0 to 3)	0 (0 to 0)	157 (80 to 261)	2.6 (1.3 to 4.3)	111 (15 to 298)	1.8 (0.2 to 4.9)	53 (11 to 97)	0.9 (0.2 to 1.6)	329 (58 to 803)	5.6 (1 to 13.4)	20 (2 to 104)	0.4 (0 to 2.1)	152 (62 to 284)	2.2 (0.9 to 4.1)
	Female	41 (27 to 58)	0.6 (0.4 to 0.9)	51 (32 to 76)	0.8 (0.5 to 1.2)	0 (0 to 0)	0 (0 to 0)	25 (13 to 41)	0.4 (0.2 to 0.6)	13 (2 to 32)	0.2 (0 to 0.5)	7 (2 to 11)	0.1 (0 to 0.2)	30 (6 to 73)	0.5 (0.1 to 1.2)	1 (0 to 3)	0 (0 to 0.1)	4 (1 to 7)	0.1 (0 to 0.1)
Turkey	Both	22200 (17500 to 27700)	25 (19.8 to 31.2)	5300 (3660 to 7370)	6 (4.2 to 8.4)	27 (8 to 67)	0 (0 to 0.1)	1820 (1060 to 2910)	2.1 (1.2 to 3.3)	1050 (154 to 2650)	1.2 (0.2 to 3)	184 (72 to 338)	0.2 (0.1 to 0.4)	2720 (572 to 6270)	3.1 (0.7 to 7.2)	5330 (2950 to 8460)	6.2 (3.5 to 9.8)	1730 (772 to 2970)	1.8 (0.8 to 3.1)
	Male	20300 (16000	48.8 (38.6	4410 (3010	10.6 (7.3	20 (6 to 52)	0 (0 to 0.1)	1400 (806	3.4 (1.9	871 (129	2.1 (0.3	143 (58 to 279)	0.3 (0.1	2270 (384	5.6 (1 to 13.3)	4940 (2630	12.5 (6.8	1620 (710	3.5 (1.5

		to 25400)	to 60.9)	to 6150)	to 14.8)			to 2250)	to 5.4)	to 2200)	to 5.3)		to 0.7)	to 5410)		to 7880)	to 19.9)	to 2800)	to 6.1)
	Female	1910 (1480 to 2400)	4.1 (3.2 to 5.1)	894 (606 to 1250)	1.9 (1.3 to 2.7)	6 (2 to 16)	0 (0 to 0)	420 (250 to 663)	0.9 (0.5 to 1.4)	178 (25 to 452)	0.4 (0.1 to 1)	41 (13 to 74)	0.1 (0 to 0.2)	455 (89 to 1090)	1 (0.2 to 2.4)	387 (194 to 664)	0.9 (0.4 to 1.5)	112 (47 to 198)	0.2 (0.1 to 0.4)
Turkmenistan	Both	240 (187 to 304)	5.9 (4.6 to 7.3)	68 (38 to 106)	1.7 (0.9 to 2.6)	0 (0 to 0)	0 (0 to 0)	36 (21 to 55)	0.9 (0.5 to 1.4)	21 (2 to 77)	0.5 (0 to 1.9)	14 (3 to 22)	0.3 (0.1 to 0.5)	26 (5 to 61)	0.7 (0.1 to 1.6)	9 (4 to 18)	0.2 (0.1 to 0.5)	24 (11 to 41)	0.5 (0.2 to 0.9)
	Male	223 (173 to 282)	12.5 (9.8 to 15.7)	51 (29 to 81)	2.8 (1.6 to 4.4)	0 (0 to 0)	0 (0 to 0)	24 (14 to 37)	1.4 (0.8 to 2.1)	16 (1 to 57)	0.9 (0.1 to 3.2)	10 (2 to 17)	0.6 (0.1 to 0.9)	19 (3 to 47)	1.2 (0.2 to 2.8)	6 (3 to 13)	0.4 (0.2 to 0.8)	20 (9 to 36)	0.9 (0.4 to 1.6)
	Female	17 (12 to 24)	0.7 (0.5 to 1)	17 (9 to 26)	0.8 (0.4 to 1.2)	0 (0 to 0)	0 (0 to 0)	12 (7 to 18)	0.5 (0.3 to 0.8)	5 (0 to 19)	0.2 (0 to 0.9)	3 (1 to 6)	0.2 (0 to 0.3)	6 (1 to 16)	0.3 (0.1 to 0.8)	2 (1 to 7)	0.1 (0 to 0.3)	3 (1 to 6)	0.1 (0.1 to 0.2)
Tuvalu	Both	2 (1 to 2)	16.2 (12.1 to 22.1)	0 (0 to 0)	0.8 (0.3 to 1.9)	0 (0 to 0)	0.6 (0.2 to 1.4)	0 (0 to 0)	1.6 (0.8 to 2.7)	0 (0 to 0)	0.9 (0.1 to 3.6)	0 (0 to 0)	1.4 (0.4 to 2.3)	0 (0 to 1)	3.9 (0.9 to 8.8)	0 (0 to 0)	1.8 (0.9 to 3.4)	0 (0 to 0)	1.2 (0.5 to 2.1)
	Male	1 (1 to 2)	28 (20.7 to 39.7)	0 (0 to 0)	1.3 (0.4 to 3)	0 (0 to 0)	0.8 (0.2 to 2)	0 (0 to 0)	2.2 (1.1 to 3.9)	0 (0 to 0)	1.4 (0.1 to 5.5)	0 (0 to 0)	2 (0.6 to 3.5)	0 (0 to 1)	5.8 (1 to 13.7)	0 (0 to 0)	3.8 (1.9 to 7.1)	0 (0 to 0)	2 (0.8 to 3.7)
	Female	0 (0 to 0)	6.3 (4.2 to 8.9)	0 (0 to 0)	0.5 (0.2 to 1.1)	0 (0 to 0)	0.5 (0.1 to 1)	0 (0 to 0)	1.1 (0.6 to 1.9)	0 (0 to 0)	0.5 (0 to 2.1)	0 (0 to 0)	0.8 (0.3 to 1.4)	0 (0 to 0)	2.4 (0.5 to 5.7)	0 (0 to 0)	0.3 (0.1 to 0.6)	0 (0 to 0)	0.4 (0.2 to 0.8)
Uganda	Both	275 (211 to 345)	2.2 (1.7 to 2.8)	66 (30 to 113)	0.5 (0.2 to 0.9)	295 (198 to 406)	2.2 (1.5 to 3.1)	19 (9 to 31)	0.1 (0.1 to 0.2)	42 (3 to 160)	0.3 (0 to 1.2)	15 (4 to 26)	0.1 (0 to 0.2)	73 (16 to 166)	0.6 (0.1 to 1.4)	38 (13 to 148)	0.3 (0.1 to 1.3)	56 (26 to 97)	0.3 (0.2 to 0.6)
	Male	232 (180 to 290)	4.4 (3.4 to 5.4)	46 (20 to 80)	0.8 (0.4 to 1.5)	177 (116 to 247)	3.2 (2.1 to 4.4)	11 (5 to 19)	0.2 (0.1 to 0.3)	26 (2 to 101)	0.5 (0 to 1.8)	9 (2 to 16)	0.2 (0 to 0.3)	50 (8 to 119)	1 (0.2 to 2.4)	25 (6 to 130)	0.6 (0.1 to 2.9)	37 (15 to 67)	0.5 (0.2 to 0.9)
	Female	43 (28 to 60)	0.6 (0.4 to 0.9)	20 (9 to 38)	0.3 (0.1 to 0.5)	118 (76 to 169)	1.6 (1 to 2.2)	8 (4 to 13)	0.1 (0 to 0.2)	16 (1 to 60)	0.2 (0 to 0.8)	6 (1 to 11)	0.1 (0 to 0.1)	23 (4 to 57)	0.3 (0.1 to 0.8)	13 (4 to 25)	0.2 (0.1 to 0.4)	20 (8 to 37)	0.2 (0.1 to 0.4)

Ukraine	Both	12300 (9900 to 14800)	16.3 (13.1 to 19.7)	1720 (957 to 2660)	2.3 (1.3 to 3.6)	94 (26 to 237)	0.1 (0 to 0.3)	730 (422 to 1150)	1 (0.6 to 1.6)	1000 (88 to 3070)	1.3 (0.1 to 4.1)	880 (297 to 1390)	1.2 (0.4 to 1.9)	885 (175 to 2130)	1.2 (0.2 to 2.8)	1120 (638 to 1740)	1.4 (0.8 to 2.2)	515 (89 to 1030)	0.7 (0.1 to 1.4)
	Male	11600 (9200 to 14000)	39.2 (31.2 to 47.4)	1430 (782 to 2230)	4.9 (2.7 to 7.6)	73 (19 to 189)	0.2 (0.1 to 0.6)	520 (292 to 839)	1.8 (1 to 2.8)	835 (74 to 2550)	2.8 (0.3 to 8.6)	731 (250 to 1170)	2.5 (0.9 to 3.9)	746 (128 to 1860)	2.5 (0.4 to 6.2)	991 (534 to 1600)	3.4 (1.8 to 5.4)	442 (73 to 887)	1.4 (0.2 to 2.9)
	Female	719 (546 to 940)	1.6 (1.2 to 2.1)	283 (158 to 442)	0.6 (0.3 to 1)	22 (6 to 52)	0 (0 to 0.1)	210 (119 to 325)	0.5 (0.3 to 0.7)	167 (14 to 500)	0.4 (0 to 1.1)	150 (47 to 243)	0.3 (0.1 to 0.5)	139 (24 to 349)	0.3 (0.1 to 0.7)	130 (48 to 281)	0.3 (0.1 to 0.6)	73 (11 to 152)	0.2 (0 to 0.4)
United Arab Emirates	Both	275 (197 to 372)	11.5 (8.3 to 15.9)	129 (81 to 190)	5 (3.1 to 7.6)	0 (0 to 0)	0 (0 to 0)	45 (25 to 72)	1.8 (1 to 2.9)	15 (1 to 53)	0.6 (0 to 2)	13 (3 to 22)	0.5 (0.1 to 0.8)	73 (17 to 167)	3.9 (0.9 to 8.9)	3 (0 to 16)	0.3 (0 to 1)	52 (22 to 97)	1 (0.4 to 1.9)
	Male	256 (182 to 348)	15.4 (11.2 to 21.4)	104 (65 to 156)	5.9 (3.7 to 8.8)	0 (0 to 0)	0 (0 to 0)	34 (19 to 56)	2 (1.1 to 3.4)	12 (1 to 43)	0.7 (0.1 to 2.4)	10 (2 to 18)	0.5 (0.1 to 1)	60 (11 to 139)	4.5 (0.9 to 10.3)	3 (0 to 15)	0.3 (0 to 1.4)	49 (20 to 92)	1.3 (0.5 to 2.5)
	Female	19 (11 to 29)	2.8 (1.5 to 4.7)	25 (15 to 37)	3.3 (1.7 to 5.6)	0 (0 to 0)	0 (0 to 0)	11 (6 to 18)	1.3 (0.6 to 2.3)	3 (0 to 11)	0.4 (0 to 1.5)	3 (1 to 5)	0.3 (0.1 to 0.7)	13 (3 to 31)	2.5 (0.5 to 6.5)	0 (0 to 1)	0.1 (0 to 0.2)	3 (1 to 6)	0.2 (0.1 to 0.4)
United Kingdom	Both	30600 (28400 to 32300)	23.5 (21.9 to 24.8)	2620 (1560 to 3970)	2 (1.2 to 3)	2 (0 to 7)	0 (0 to 0)	1410 (803 to 2140)	1.1 (0.7 to 1.7)	2270 (444 to 4600)	1.7 (0.3 to 3.5)	1910 (596 to 2850)	1.5 (0.5 to 2.2)	5590 (1410 to 11700)	4.2 (1 to 8.7)	14300 (11100 to 17100)	10.3 (8.1 to 12.4)	1430 (257 to 2730)	1.2 (0.2 to 2.3)
	Male	17100 (16100 to 17900)	28.7 (27 to 30.1)	1430 (853 to 2170)	2.4 (1.4 to 3.7)	1 (0 to 3)	0 (0 to 0)	798 (449 to 1220)	1.4 (0.8 to 2.1)	1230 (236 to 2520)	2.1 (0.4 to 4.2)	1020 (320 to 1530)	1.7 (0.5 to 2.6)	3200 (569 to 7040)	5.3 (0.9 to 11.7)	11200 (8340 to 13700)	18.1 (13.5 to 22.3)	961 (165 to 1840)	1.7 (0.3 to 3.2)
	Female	13500 (12300 to 14600)	19.2 (17.6 to 20.6)	1190 (711 to 1790)	1.7 (1 to 2.5)	1 (0 to 4)	0 (0 to 0)	610 (339 to 947)	0.9 (0.5 to 1.5)	1040 (209 to 2080)	1.5 (0.3 to 3)	885 (280 to 1340)	1.3 (0.4 to 1.9)	2400 (497 to 5450)	3.2 (0.7 to 7.3)	3070 (1660 to 4320)	3.9 (2.1 to 5.5)	469 (71 to 916)	0.8 (0.1 to 1.5)
Tanzania	Both	951 (659 to 1430)	4.3 (3 to 6.4)	120 (52 to 230)	0.5 (0.2 to 1)	643 (400 to 1020)	2.8 (1.8 to 4.3)	61 (28 to 111)	0.3 (0.1 to 0.5)	88 (5 to 373)	0.4 (0 to 1.6)	90 (22 to 174)	0.4 (0.1 to 0.7)	121 (24 to 311)	0.6 (0.1 to 1.5)	103 (28 to 463)	0.5 (0.1 to 2.3)	137 (56 to 270)	0.5 (0.2 to 1)
	Male	843 (573 to 1310)	8.1 (5.6	94 (38 to 190)	0.9 (0.4	452 (262	4.2 (2.5	37 (16 to 76)	0.3 (0.2	64 (4 to 274)	0.6 (0 to 2.5)	64 (15 to 133)	0.6 (0.1	92 (14 to 254)	0.9 (0.1	78 (15 to 424)	0.9 (0.2	100 (38 to 211)	0.8 (0.3

			to 12.4)		to 1.7)	to 778)	to 7.1)		to 0.7)				to 1.2)		to 2.5)		to 4.7)		to 1.6)
	Female	108 (77 to 144)	0.9 (0.7 to 1.3)	26 (12 to 47)	0.2 (0.1 to 0.4)	192 (132 to 261)	1.6 (1.1 to 2.1)	23 (12 to 39)	0.2 (0.1 to 0.3)	24 (2 to 92)	0.2 (0 to 0.8)	26 (7 to 42)	0.2 (0.1 to 0.3)	29 (5 to 72)	0.3 (0 to 0.6)	25 (7 to 53)	0.2 (0.1 to 0.5)	37 (15 to 68)	0.3 (0.1 to 0.5)
Virgin Islands	Both	21 (17 to 26)	10.9 (8.8 to 13.2)	2 (1 to 4)	1.3 (0.6 to 2.2)	0 (0 to 0)	0 (0 to 0.1)	2 (1 to 3)	1 (0.6 to 1.5)	1 (0 to 5)	0.7 (0.1 to 2.4)	1 (0 to 1)	0.5 (0.1 to 0.8)	6 (1 to 13)	3.1 (0.8 to 6.6)	7 (4 to 10)	3.7 (2.3 to 5.3)	3 (1 to 4)	1.4 (0.7 to 2.3)
	Male	15 (12 to 19)	17.9 (14.4 to 21.7)	2 (1 to 3)	1.9 (0.8 to 3.2)	0 (0 to 0)	0 (0 to 0.1)	1 (1 to 2)	1.5 (0.8 to 2.4)	1 (0 to 3)	1 (0.1 to 3.6)	1 (0 to 1)	0.6 (0.2 to 1.1)	4 (1 to 9)	4.6 (0.8 to 10.6)	6 (4 to 10)	7.9 (4.7 to 11.9)	2 (1 to 4)	2.3 (1 to 4)
	Female	6 (4 to 8)	5.4 (4 to 7)	1 (0 to 2)	0.8 (0.4 to 1.5)	0 (0 to 0)	0 (0 to 0.1)	1 (0 to 1)	0.6 (0.3 to 0.9)	0 (0 to 2)	0.4 (0 to 1.5)	0 (0 to 1)	0.3 (0.1 to 0.6)	2 (0 to 5)	1.9 (0.4 to 4.3)	1 (0 to 1)	0.6 (0.3 to 0.9)	1 (0 to 1)	0.6 (0.3 to 1.1)
United States of America	Both	147000 (138000 to 155000)	25.8 (24.2 to 27.1)	8230 (3930 to 13800)	1.4 (0.7 to 2.4)	26 (5 to 82)	0 (0 to 0)	6720 (4050 to 10000)	1.2 (0.7 to 1.8)	9080 (1800 to 17400)	1.6 (0.3 to 3.1)	7600 (1920 to 11300)	1.3 (0.3 to 2)	27700 (6900 to 57300)	4.8 (1.2 to 9.9)	35600 (25700 to 46300)	6 (4.3 to 7.8)	6280 (1040 to 12200)	1.1 (0.2 to 2.2)
	Male	85100 (80800 to 88800)	32.9 (31.2 to 34.4)	4540 (2180 to 7550)	1.8 (0.8 to 2.9)	12 (2 to 37)	0 (0 to 0)	4030 (2410 to 5980)	1.6 (0.9 to 2.3)	4990 (994 to 9570)	1.9 (0.4 to 3.7)	4130 (1110 to 6250)	1.6 (0.4 to 2.4)	16800 (3050 to 36300)	6.5 (1.2 to 13.9)	30500 (20700 to 40700)	11.8 (8.1 to 15.8)	3860 (586 to 7570)	1.5 (0.2 to 2.9)
	Female	62300 (56700 to 67300)	19.9 (18.3 to 21.5)	3690 (1780 to 6170)	1.2 (0.6 to 2)	14 (3 to 45)	0 (0 to 0)	2690 (1610 to 4070)	0.9 (0.5 to 1.4)	4090 (809 to 7870)	1.3 (0.3 to 2.5)	3460 (900 to 5320)	1.1 (0.3 to 1.7)	10900 (2250 to 24700)	3.4 (0.7 to 7.7)	5150 (3020 to 7600)	1.5 (0.9 to 2.3)	2420 (352 to 4810)	0.8 (0.1 to 1.6)
Uruguay	Both	1180 (1090 to 1260)	22.7 (21 to 24.4)	104 (47 to 174)	2 (0.9 to 3.3)	7 (2 to 16)	0.1 (0 to 0.3)	106 (62 to 161)	2 (1.2 to 3.1)	54 (4 to 201)	1 (0.1 to 3.8)	62 (14 to 94)	1.2 (0.3 to 1.8)	133 (28 to 301)	2.4 (0.5 to 5.5)	155 (93 to 228)	2.7 (1.6 to 3.9)	111 (55 to 176)	2.3 (1.1 to 3.6)
	Male	940 (870 to 1010)	41.7 (38.6 to 44.7)	77 (34 to 128)	3.4 (1.5 to 5.7)	4 (1 to 11)	0.2 (0.1 to 0.5)	77 (45 to 117)	3.4 (2 to 5.2)	40 (3 to 148)	1.8 (0.1 to 6.6)	45 (11 to 69)	2 (0.5 to 3.1)	105 (17 to 244)	4.6 (0.8 to 10.6)	142 (81 to 211)	6.1 (3.5 to 9.1)	91 (42 to 147)	4.1 (1.9 to 6.6)
	Female	239 (210 to 271)	8.3 (7.4 to 9.3)	27 (13 to 45)	0.9 (0.4 to 1.5)	2 (1 to 6)	0.1 (0 to 0.2)	29 (17 to 43)	1 (0.6 to 1.5)	14 (1 to 52)	0.5 (0 to 1.7)	17 (4 to 26)	0.6 (0.1 to 0.9)	28 (5 to 68)	0.9 (0.2 to 2.1)	13 (7 to 20)	0.4 (0.2 to 0.6)	20 (9 to 34)	0.8 (0.3 to 1.3)

Uzbekistan	Both	1330 (1080 to 1620)	6.4 (5.2 to 7.6)	518 (314 to 765)	2.5 (1.6 to 3.7)	56 (21 to 117)	0.3 (0.1 to 0.6)	119 (64 to 192)	0.6 (0.3 to 0.9)	160 (14 to 597)	0.8 (0.1 to 2.9)	101 (23 to 161)	0.5 (0.1 to 0.8)	208 (44 to 468)	1.1 (0.2 to 2.4)	50 (28 to 83)	0.3 (0.2 to 0.5)	237 (113 to 392)	0.8 (0.4 to 1.4)
	Male	1280 (1040 to 1550)	14.3 (11.8 to 17)	377 (226 to 560)	4.1 (2.5 to 6.2)	36 (13 to 80)	0.4 (0.1 to 0.9)	75 (39 to 120)	0.9 (0.4 to 1.4)	115 (10 to 436)	1.3 (0.1 to 4.8)	72 (16 to 117)	0.8 (0.2 to 1.3)	156 (25 to 361)	1.8 (0.3 to 4.2)	41 (21 to 72)	0.6 (0.3 to 1)	197 (90 to 335)	1.5 (0.7 to 2.6)
	Female	55 (38 to 78)	0.5 (0.3 to 0.6)	141 (84 to 210)	1.3 (0.8 to 1.9)	20 (8 to 41)	0.2 (0.1 to 0.4)	44 (25 to 70)	0.4 (0.2 to 0.6)	45 (4 to 166)	0.4 (0 to 1.6)	29 (6 to 46)	0.3 (0.1 to 0.4)	53 (10 to 126)	0.5 (0.1 to 1.3)	8 (4 to 14)	0.1 (0.1 to 0.2)	40 (18 to 70)	0.3 (0.1 to 0.5)
Vanuatu	Both	21 (13 to 31)	13 (8.2 to 19.9)	2 (0 to 5)	1.1 (0.3 to 2.8)	11 (6 to 18)	6.9 (4 to 11.1)	2 (1 to 3)	0.9 (0.4 to 1.6)	1 (0 to 6)	0.9 (0.1 to 3.5)	2 (0 to 3)	1.1 (0.2 to 1.9)	6 (1 to 13)	3.6 (0.7 to 8.3)	4 (1 to 8)	2.7 (1 to 5)	2 (1 to 5)	1.2 (0.5 to 2.4)
	Male	19 (12 to 30)	23.5 (14.5 to 36.4)	2 (0 to 4)	1.9 (0.5 to 4.8)	9 (5 to 15)	10.4 (5.5 to 17.7)	1 (0 to 2)	1.3 (0.6 to 2.5)	1 (0 to 5)	1.3 (0.1 to 5.5)	1 (0 to 3)	1.6 (0.3 to 3.1)	5 (1 to 11)	5.5 (0.9 to 13.6)	4 (1 to 7)	5.1 (1.8 to 9.5)	2 (1 to 4)	2 (0.8 to 4)
	Female	1 (1 to 2)	1.4 (0.9 to 2.1)	0 (0 to 1)	0.4 (0.1 to 0.8)	2 (1 to 4)	3.1 (1.8 to 4.7)	0 (0 to 1)	0.5 (0.2 to 0.8)	0 (0 to 1)	0.4 (0 to 1.3)	0 (0 to 1)	0.4 (0.1 to 0.8)	1 (0 to 3)	1.4 (0.3 to 3.4)	0 (0 to 0)	0.2 (0 to 0.3)	0 (0 to 1)	0.4 (0.2 to 0.8)
Venezuela	Both	2610 (1920 to 3420)	9.1 (6.7 to 12)	853 (490 to 1290)	3 (1.7 to 4.5)	6 (2 to 14)	0 (0 to 0)	208 (102 to 359)	0.7 (0.4 to 1.2)	272 (25 to 932)	0.9 (0.1 to 3.2)	193 (45 to 317)	0.7 (0.2 to 1.1)	646 (147 to 1510)	2.3 (0.5 to 5.3)	133 (62 to 238)	0.5 (0.2 to 0.9)	389 (178 to 665)	1.2 (0.6 to 2.1)
	Male	1930 (1410 to 2550)	14.6 (10.8 to 19.2)	531 (306 to 818)	4 (2.3 to 6.1)	3 (1 to 7)	0 (0 to 0.1)	130 (63 to 224)	1 (0.5 to 1.7)	169 (16 to 575)	1.3 (0.1 to 4.3)	118 (26 to 193)	0.9 (0.2 to 1.4)	413 (72 to 1000)	3.2 (0.6 to 7.6)	107 (45 to 202)	0.9 (0.4 to 1.6)	294 (126 to 520)	2 (0.8 to 3.5)
	Female	685 (477 to 966)	4.5 (3.2 to 6.3)	322 (182 to 504)	2.1 (1.2 to 3.3)	3 (1 to 7)	0 (0 to 0)	79 (38 to 140)	0.5 (0.2 to 0.9)	103 (10 to 366)	0.7 (0.1 to 2.4)	75 (17 to 128)	0.5 (0.1 to 0.8)	233 (46 to 589)	1.5 (0.3 to 3.9)	26 (12 to 50)	0.2 (0.1 to 0.3)	95 (40 to 175)	0.6 (0.2 to 1.1)
Vietnam	Both	14400 (11100 to 18200)	15.5 (12.1 to 19.5)	2860 (1750 to 4180)	3.1 (1.9 to 4.4)	2410 (1180 to 4260)	2.6 (1.3 to 4.6)	1510 (840 to 2450)	1.6 (0.9 to 2.6)	519 (48 to 1850)	0.6 (0.1 to 2)	1110 (290 to 1840)	1.2 (0.3 to 2)	1950 (414 to 4420)	2.3 (0.5 to 5.2)	1010 (530 to 1790)	1.2 (0.6 to 2.1)	2130 (938 to 3700)	2 (0.9 to 3.4)
	Male	13600 (10400	34.9 (27.3	2140 (1290	5.4 (3.3	1590 (737	4 (1.9 to 7.2)	789 (424	2.1 (1.1	374 (34 to 1340)	0.9 (0.1	800 (201	2 (0.5 to 3.3)	1390 (225	4.1 (0.7	956 (483	2.9 (1.6	1630 (680	3.3 (1.4

		to 17200)	to 43.7)	to 3120)	to 7.7)	to 2890)		to 1300)	to 3.4)		to 3.3)	to 1320)		to 3350)	to 9.8)	to 1710)	to 5.1)	to 2930)	to 5.9)
	Female	827 (570 to 1150)	1.7 (1.2 to 2.3)	725 (427 to 1100)	1.4 (0.8 to 2.1)	818 (425 to 1350)	1.6 (0.8 to 2.6)	723 (401 to 1170)	1.4 (0.8 to 2.2)	145 (13 to 512)	0.3 (0 to 1)	314 (87 to 532)	0.6 (0.2 to 1)	566 (106 to 1380)	1.1 (0.2 to 2.8)	56 (24 to 108)	0.1 (0.1 to 0.2)	494 (198 to 924)	0.9 (0.3 to 1.6)
Yemen	Both	906 (610 to 1380)	7.4 (5 to 11.3)	208 (93 to 382)	1.7 (0.8 to 3)	156 (79 to 289)	1.2 (0.6 to 2.3)	103 (55 to 176)	0.8 (0.4 to 1.4)	49 (4 to 195)	0.4 (0 to 1.6)	72 (22 to 135)	0.6 (0.2 to 1.1)	102 (20 to 258)	0.9 (0.2 to 2.2)	10 (2 to 46)	0.1 (0 to 0.4)	80 (30 to 160)	0.5 (0.2 to 1.1)
	Male	807 (528 to 1270)	13.4 (8.8 to 21.1)	169 (75 to 323)	2.8 (1.2 to 5.3)	116 (53 to 227)	1.9 (0.9 to 3.7)	76 (39 to 138)	1.3 (0.6 to 2.3)	39 (4 to 156)	0.6 (0.1 to 2.6)	56 (17 to 111)	0.9 (0.3 to 1.8)	81 (13 to 219)	1.4 (0.2 to 3.8)	9 (1 to 43)	0.2 (0 to 0.9)	78 (30 to 157)	1.1 (0.4 to 2.1)
	Female	99 (72 to 133)	1.6 (1.2 to 2.1)	39 (17 to 67)	0.6 (0.3 to 1)	40 (22 to 64)	0.6 (0.3 to 1)	27 (15 to 44)	0.4 (0.2 to 0.6)	11 (1 to 40)	0.2 (0 to 0.6)	16 (5 to 27)	0.2 (0.1 to 0.4)	21 (4 to 50)	0.4 (0.1 to 0.8)	2 (1 to 4)	0 (0 to 0.1)	2 (1 to 3)	0 (0 to 0)
Zambia	Both	250 (174 to 331)	4.4 (3.2 to 5.7)	60 (29 to 105)	1 (0.5 to 1.7)	160 (93 to 241)	2.6 (1.5 to 3.8)	15 (6 to 27)	0.2 (0.1 to 0.4)	27 (2 to 104)	0.4 (0 to 1.7)	47 (16 to 79)	0.7 (0.3 to 1.3)	45 (9 to 107)	0.8 (0.2 to 1.9)	38 (14 to 122)	0.7 (0.3 to 2.3)	43 (18 to 79)	0.6 (0.2 to 1)
	Male	216 (145 to 291)	8.1 (5.5 to 10.7)	45 (22 to 79)	1.6 (0.7 to 2.7)	106 (60 to 167)	3.7 (2.1 to 5.8)	9 (3 to 17)	0.3 (0.1 to 0.6)	19 (1 to 70)	0.6 (0 to 2.5)	32 (11 to 56)	1.1 (0.4 to 1.9)	34 (6 to 84)	1.3 (0.2 to 3.3)	30 (9 to 114)	1.3 (0.4 to 4.7)	32 (12 to 61)	0.8 (0.3 to 1.6)
	Female	34 (23 to 47)	1.3 (0.9 to 1.7)	15 (7 to 27)	0.4 (0.2 to 0.8)	54 (32 to 81)	1.6 (1 to 2.4)	6 (3 to 12)	0.2 (0.1 to 0.3)	8 (0 to 31)	0.2 (0 to 0.9)	14 (5 to 24)	0.4 (0.1 to 0.7)	11 (2 to 27)	0.4 (0.1 to 0.9)	8 (2 to 16)	0.3 (0.1 to 0.6)	11 (4 to 21)	0.3 (0.1 to 0.5)
Zimbabwe	Both	475 (377 to 575)	7.4 (5.8 to 8.8)	66 (31 to 114)	1 (0.4 to 1.6)	231 (147 to 325)	3.4 (2.2 to 4.8)	37 (20 to 60)	0.5 (0.3 to 0.9)	43 (3 to 161)	0.6 (0 to 2.4)	66 (22 to 105)	1 (0.3 to 1.5)	89 (21 to 196)	1.4 (0.3 to 3.1)	45 (25 to 75)	0.8 (0.4 to 1.3)	35 (15 to 62)	0.4 (0.2 to 0.7)
	Male	380 (303 to 461)	14.1 (11.5 to 16.9)	42 (19 to 72)	1.5 (0.7 to 2.5)	123 (78 to 176)	4.3 (2.8 to 6.1)	14 (7 to 23)	0.5 (0.3 to 0.8)	24 (2 to 88)	0.8 (0.1 to 3.1)	37 (13 to 59)	1.3 (0.4 to 2.1)	46 (8 to 110)	1.8 (0.3 to 4.3)	24 (12 to 44)	1.1 (0.6 to 2)	22 (9 to 42)	0.6 (0.3 to 1.1)
	Female	95 (63 to 133)	2.7 (1.8 to 3.8)	24 (10 to 42)	0.6 (0.3 to 1.1)	107 (65 to 156)	2.8 (1.7 to 4)	23 (12 to 39)	0.6 (0.3 to 0.9)	19 (1 to 71)	0.5 (0 to 1.8)	28 (9 to 48)	0.7 (0.2 to 1.2)	44 (8 to 106)	1.2 (0.2 to 2.9)	21 (8 to 40)	0.6 (0.2 to 1.2)	13 (5 to 24)	0.3 (0.1 to 0.5)

Table S5. Larynx cancer deaths (counts and age-standardised rates) attributable to specific risk factors in 2019, at global, regional, national levels, and Socio-demographic Index (SDI) quintiles, by sex and for both sexes combined.

95% UIs given in parentheses.

Location	Sex	Smoking		Alcohol use		Occupational asbestos exposure		Occupational exposure to sulfuric acid	
		Number	ASDR per 100,000	Number	ASDR per 100,000	Number	ASDR per 100,000	Number	ASDR per 100,000
Global	Both	78300 (68000 to 88300)	0.95 (0.82 to 1.07)	23900 (14100 to 32600)	0.29 (0.17 to 0.39)	3680 (2040 to 5530)	0.05 (0.03 to 0.07)	4030 (1730 to 7470)	0.05 (0.02 to 0.09)
	Male	73400 (63900 to 82700)	1.91 (1.66 to 2.14)	22900 (13700 to 31100)	0.59 (0.35 to 0.8)	3450 (1870 to 5280)	0.1 (0.05 to 0.15)	3590 (1520 to 6670)	0.09 (0.04 to 0.16)
	Female	4870 (3720 to 5950)	0.11 (0.08 to 0.14)	1010 (487 to 1550)	0.02 (0.01 to 0.04)	234 (106 to 377)	0.01 (0 to 0.01)	438 (188 to 799)	0.01 (0 to 0.02)
High SDI	Both	10400 (8750 to 11700)	0.56 (0.48 to 0.63)	4240 (2570 to 5630)	0.23 (0.14 to 0.31)	1500 (854 to 2180)	0.07 (0.04 to 0.11)	348 (92 to 746)	0.02 (0.01 to 0.04)
	Male	9040 (7670 to 10200)	1.06 (0.91 to 1.19)	3820 (2360 to 4990)	0.45 (0.28 to 0.59)	1430 (791 to 2110)	0.16 (0.09 to 0.23)	299 (81 to 635)	0.04 (0.01 to 0.08)
	Female	1310 (1030 to 1550)	0.13 (0.11 to 0.15)	424 (223 to 617)	0.04 (0.02 to 0.06)	68 (30 to 111)	0.01 (0 to 0.01)	49 (12 to 107)	0.01 (0 to 0.01)
High-middle SDI	Both	21900 (19200 to 24400)	1.06 (0.93 to 1.18)	8220 (5050 to 10900)	0.4 (0.24 to 0.53)	1050 (573 to 1590)	0.05 (0.03 to 0.08)	841 (340 to 1570)	0.04 (0.02 to 0.08)
	Male	20800 (18200 to 23100)	2.23 (1.95 to 2.48)	7980 (4900 to 10500)	0.85 (0.52 to 1.12)	989 (523 to 1530)	0.12 (0.06 to 0.18)	760 (305 to 1430)	0.08 (0.03 to 0.14)
	Female	1150 (888 to 1410)	0.1 (0.08 to 0.12)	242 (113 to 375)	0.02 (0.01 to 0.03)	56 (26 to 92)	0 (0 to 0.01)	80 (34 to 148)	0.01 (0 to 0.01)
Middle SDI	Both	21900 (18500 to 25200)	0.89 (0.75 to 1.02)	6310 (3630 to 8930)	0.25 (0.14 to 0.35)	512 (280 to 804)	0.02 (0.01 to 0.04)	1230 (512 to 2280)	0.05 (0.02 to 0.08)
	Male	20800 (17600 to 24000)	1.8 (1.53 to 2.06)	6120 (3560 to 8650)	0.51 (0.29 to 0.71)	464 (231 to 753)	0.05 (0.02 to 0.08)	1090 (454 to 2010)	0.08 (0.03 to 0.15)
	Female	1120 (791 to 1440)	0.09 (0.06 to 0.12)	185 (81 to 299)	0.01 (0.01 to 0.02)	48 (22 to 80)	0 (0 to 0.01)	143 (59 to 264)	0.01 (0 to 0.02)
Low-middle SDI	Both	18800 (15600 to 22300)	1.38 (1.15 to 1.64)	3920 (2160 to 5760)	0.28 (0.15 to 0.41)	508 (269 to 819)	0.04 (0.02 to 0.07)	1200 (507 to 2250)	0.08 (0.03 to 0.15)
	Male	17900 (14900 to 21200)	2.76 (2.31 to 3.26)	3820 (2110 to 5620)	0.56 (0.31 to 0.83)	463 (232 to 762)	0.08 (0.04 to 0.14)	1080 (459 to 2030)	0.15 (0.06 to 0.28)
	Female	920 (635 to 1240)	0.13 (0.09 to 0.18)	103 (41 to 174)	0.01 (0.01 to 0.02)	46 (19 to 82)	0.01 (0 to 0.01)	120 (49 to 225)	0.02 (0.01 to 0.03)

Low SDI	Both	5220 (4060 to 6600)	1.02 (0.8 to 1.28)	1190 (609 to 1770)	0.22 (0.11 to 0.33)	116 (55 to 258)	0.03 (0.01 to 0.06)	410 (170 to 771)	0.07 (0.03 to 0.13)
	Male	4870 (3780 to 6160)	1.95 (1.53 to 2.44)	1130 (586 to 1670)	0.42 (0.22 to 0.63)	99 (42 to 236)	0.05 (0.02 to 0.11)	364 (149 to 688)	0.12 (0.05 to 0.23)
	Female	358 (247 to 487)	0.14 (0.1 to 0.19)	58 (25 to 94)	0.02 (0.01 to 0.03)	17 (6 to 30)	0.01 (0 to 0.01)	47 (19 to 85)	0.02 (0.01 to 0.03)
Central Asia	Both	869 (747 to 997)	1.14 (0.98 to 1.31)	260 (150 to 365)	0.32 (0.18 to 0.45)	22 (12 to 35)	0.03 (0.02 to 0.05)	48 (20 to 90)	0.05 (0.02 to 0.1)
	Male	848 (729 to 977)	2.66 (2.27 to 3.04)	247 (144 to 346)	0.7 (0.4 to 0.99)	20 (10 to 32)	0.08 (0.04 to 0.12)	40 (16 to 75)	0.1 (0.04 to 0.19)
	Female	21 (13 to 31)	0.05 (0.03 to 0.07)	12 (4 to 21)	0.03 (0.01 to 0.05)	2 (1 to 4)	0.01 (0 to 0.01)	8 (3 to 14)	0.02 (0.01 to 0.03)
Central Europe	Both	3990 (3330 to 4700)	1.96 (1.64 to 2.3)	1850 (1160 to 2460)	0.92 (0.57 to 1.22)	155 (79 to 245)	0.07 (0.04 to 0.11)	121 (27 to 273)	0.06 (0.01 to 0.14)
	Male	3730 (3110 to 4400)	4.06 (3.38 to 4.78)	1800 (1120 to 2390)	1.97 (1.23 to 2.61)	149 (75 to 240)	0.16 (0.08 to 0.25)	112 (25 to 251)	0.12 (0.03 to 0.28)
	Female	259 (198 to 323)	0.23 (0.18 to 0.29)	48 (19 to 78)	0.04 (0.02 to 0.07)	6 (2 to 10)	0 (0 to 0.01)	10 (2 to 22)	0.01 (0 to 0.02)
Eastern Europe	Both	5160 (4340 to 6000)	1.51 (1.27 to 1.75)	2200 (1320 to 2940)	0.65 (0.39 to 0.87)	127 (65 to 207)	0.04 (0.02 to 0.06)	118 (26 to 261)	0.04 (0.01 to 0.08)
	Male	5090 (4290 to 5920)	3.72 (3.14 to 4.32)	2150 (1290 to 2860)	1.56 (0.94 to 2.09)	121 (60 to 201)	0.09 (0.05 to 0.15)	111 (25 to 247)	0.08 (0.02 to 0.17)
	Female	76 (52 to 103)	0.04 (0.03 to 0.06)	50 (23 to 78)	0.03 (0.01 to 0.04)	5 (2 to 9)	0 (0 to 0)	7 (1 to 15)	0 (0 to 0.01)
Australasia	Both	143 (109 to 174)	0.29 (0.23 to 0.35)	98 (60 to 130)	0.2 (0.12 to 0.26)	53 (31 to 76)	0.1 (0.06 to 0.14)	6 (1 to 14)	0.01 (0 to 0.03)
	Male	124 (94 to 152)	0.54 (0.42 to 0.66)	90 (56 to 119)	0.39 (0.24 to 0.51)	51 (29 to 73)	0.21 (0.12 to 0.3)	6 (1 to 13)	0.03 (0.01 to 0.06)
	Female	19 (14 to 24)	0.07 (0.05 to 0.09)	8 (4 to 12)	0.03 (0.01 to 0.05)	2 (1 to 4)	0.01 (0 to 0.01)	1 (0 to 2)	0 (0 to 0.01)
High-income Asia Pacific	Both	1280 (1060 to 1450)	0.27 (0.23 to 0.3)	419 (236 to 600)	0.09 (0.05 to 0.13)	137 (69 to 210)	0.02 (0.01 to 0.04)	31 (7 to 68)	0.01 (0 to 0.02)
	Male	1200 (1010 to 1360)	0.58 (0.48 to 0.65)	391 (223 to 559)	0.19 (0.11 to 0.27)	133 (66 to 206)	0.06 (0.03 to 0.09)	28 (7 to 61)	0.01 (0 to 0.03)
	Female	72 (49 to 99)	0.03 (0.02 to 0.03)	27 (14 to 44)	0.01 (0.01 to 0.02)	4 (1 to 7)	0 (0 to 0)	3 (1 to 6)	0 (0 to 0)
High-income North America	Both	3910 (3180 to 4500)	0.62 (0.51 to 0.71)	1340 (775 to 1820)	0.22 (0.13 to 0.29)	476 (266 to 699)	0.07 (0.04 to 0.1)	151 (37 to 331)	0.03 (0.01 to 0.05)
	Male	3210 (2610 to 3680)	1.11 (0.9 to 1.27)	1160 (680 to 1560)	0.4 (0.24 to 0.55)	451 (241 to 675)	0.16 (0.08 to 0.23)	122 (29 to 264)	0.04 (0.01 to 0.09)
	Female	703 (565 to 828)	0.21 (0.17 to 0.24)	179 (92 to 267)	0.05 (0.03 to 0.08)	24 (11 to 40)	0.01 (0 to 0.01)	29 (7 to 64)	0.01 (0 to 0.02)

Southern Latin America	Both	858 (705 to 1000)	1.03 (0.85 to 1.21)	430 (267 to 573)	0.52 (0.32 to 0.69)	56 (28 to 91)	0.07 (0.03 to 0.11)	46 (19 to 84)	0.06 (0.02 to 0.1)
	Male	772 (635 to 902)	2.08 (1.7 to 2.43)	399 (250 to 527)	1.08 (0.68 to 1.43)	53 (25 to 87)	0.15 (0.07 to 0.24)	42 (17 to 77)	0.11 (0.05 to 0.2)
	Female	85 (65 to 105)	0.19 (0.14 to 0.23)	31 (16 to 48)	0.07 (0.04 to 0.1)	4 (2 to 7)	0.01 (0 to 0.01)	4 (2 to 7)	0.01 (0 to 0.02)
Western Europe	Both	6870 (5890 to 7700)	0.8 (0.69 to 0.88)	3260 (2030 to 4190)	0.38 (0.24 to 0.49)	1240 (705 to 1810)	0.12 (0.07 to 0.18)	182 (42 to 409)	0.02 (0.01 to 0.05)
	Male	6230 (5340 to 6980)	1.57 (1.35 to 1.74)	3020 (1890 to 3850)	0.77 (0.48 to 0.97)	1190 (667 to 1760)	0.27 (0.15 to 0.41)	164 (38 to 369)	0.04 (0.01 to 0.1)
	Female	641 (481 to 770)	0.14 (0.11 to 0.16)	245 (133 to 355)	0.05 (0.03 to 0.08)	50 (21 to 82)	0.01 (0 to 0.01)	17 (4 to 39)	0 (0 to 0.01)
Andean Latin America	Both	129 (89 to 177)	0.24 (0.16 to 0.33)	57 (27 to 89)	0.1 (0.05 to 0.16)	11 (5 to 18)	0.02 (0.01 to 0.03)	14 (6 to 27)	0.03 (0.01 to 0.05)
	Male	121 (84 to 167)	0.47 (0.32 to 0.64)	53 (26 to 82)	0.2 (0.09 to 0.31)	9 (4 to 17)	0.04 (0.02 to 0.07)	12 (5 to 22)	0.04 (0.02 to 0.08)
	Female	8 (4 to 12)	0.03 (0.02 to 0.04)	4 (1 to 7)	0.01 (0 to 0.02)	1 (1 to 2)	0 (0 to 0.01)	3 (1 to 5)	0.01 (0 to 0.02)
Caribbean	Both	1010 (807 to 1230)	1.93 (1.55 to 2.36)	305 (165 to 441)	0.58 (0.32 to 0.85)	21 (10 to 35)	0.04 (0.02 to 0.07)	55 (23 to 101)	0.11 (0.04 to 0.19)
	Male	920 (738 to 1120)	3.78 (3.04 to 4.63)	293 (158 to 423)	1.19 (0.64 to 1.72)	20 (9 to 33)	0.08 (0.04 to 0.14)	50 (21 to 92)	0.2 (0.08 to 0.37)
	Female	85 (63 to 109)	0.31 (0.23 to 0.39)	12 (5 to 19)	0.04 (0.02 to 0.07)	1 (1 to 2)	0 (0 to 0.01)	5 (2 to 10)	0.02 (0.01 to 0.04)
Central Latin America	Both	1190 (894 to 1520)	0.52 (0.39 to 0.66)	467 (255 to 669)	0.2 (0.11 to 0.28)	54 (28 to 86)	0.02 (0.01 to 0.04)	97 (40 to 179)	0.04 (0.02 to 0.07)
	Male	1110 (833 to 1400)	1.05 (0.79 to 1.33)	446 (245 to 637)	0.41 (0.22 to 0.58)	49 (24 to 82)	0.05 (0.02 to 0.08)	86 (36 to 158)	0.08 (0.03 to 0.14)
	Female	87 (56 to 127)	0.07 (0.04 to 0.1)	21 (8 to 37)	0.02 (0.01 to 0.03)	4 (2 to 8)	0 (0 to 0.01)	11 (4 to 22)	0.01 (0 to 0.02)
Tropical Latin America	Both	3190 (2610 to 3730)	1.3 (1.06 to 1.53)	1180 (674 to 1630)	0.47 (0.27 to 0.65)	152 (85 to 233)	0.06 (0.04 to 0.1)	200 (83 to 370)	0.08 (0.03 to 0.15)
	Male	2880 (2390 to 3360)	2.6 (2.13 to 3.05)	1130 (652 to 1560)	0.99 (0.57 to 1.36)	133 (69 to 211)	0.13 (0.07 to 0.21)	182 (75 to 334)	0.15 (0.06 to 0.28)
	Female	306 (225 to 387)	0.23 (0.17 to 0.29)	48 (20 to 79)	0.04 (0.02 to 0.06)	19 (9 to 32)	0.01 (0.01 to 0.02)	18 (7 to 35)	0.01 (0.01 to 0.03)
North Africa and Middle East	Both	5100 (4310 to 5920)	1.22 (1.02 to 1.43)	263 (128 to 414)	0.06 (0.03 to 0.09)	133 (63 to 237)	0.04 (0.02 to 0.07)	212 (86 to 388)	0.04 (0.02 to 0.08)
	Male	4890 (4130 to 5660)	2.31 (1.94 to 2.7)	253 (123 to 397)	0.11 (0.05 to 0.17)	124 (56 to 224)	0.07 (0.03 to 0.12)	199 (81 to 366)	0.08 (0.03 to 0.15)
	Female	217 (157 to 285)	0.1 (0.07 to 0.14)	9 (4 to 17)	0 (0 to 0.01)	9 (4 to 16)	0 (0 to 0.01)	12 (5 to 23)	0.01 (0 to 0.01)

South Asia	Both	21700 (17400 to 27000)	1.55 (1.26 to 1.93)	4530 (2390 to 6800)	0.31 (0.16 to 0.46)	617 (322 to 996)	0.05 (0.03 to 0.08)	1530 (644 to 2890)	0.1 (0.04 to 0.19)
	Male	20700 (16600 to 25700)	3.02 (2.43 to 3.73)	4450 (2360 to 6680)	0.61 (0.32 to 0.93)	570 (279 to 941)	0.1 (0.05 to 0.16)	1410 (585 to 2680)	0.18 (0.08 to 0.35)
	Female	1010 (674 to 1410)	0.14 (0.1 to 0.2)	82 (30 to 148)	0.01 (0 to 0.02)	48 (18 to 93)	0.01 (0 to 0.01)	118 (47 to 229)	0.02 (0.01 to 0.03)
East Asia	Both	15800 (12500 to 19300)	0.75 (0.6 to 0.92)	4800 (2770 to 7010)	0.23 (0.13 to 0.33)	236 (126 to 381)	0.01 (0.01 to 0.02)	779 (323 to 1500)	0.03 (0.01 to 0.07)
	Male	14800 (11600 to 18200)	1.54 (1.23 to 1.88)	4660 (2690 to 6820)	0.47 (0.27 to 0.68)	196 (93 to 333)	0.03 (0.01 to 0.04)	648 (266 to 1280)	0.06 (0.02 to 0.11)
	Female	972 (645 to 1330)	0.09 (0.06 to 0.12)	139 (60 to 233)	0.01 (0.01 to 0.02)	40 (16 to 68)	0 (0 to 0.01)	131 (55 to 249)	0.01 (0 to 0.02)
Oceania	Both	27 (19 to 37)	0.42 (0.3 to 0.55)	4 (1 to 7)	0.05 (0.01 to 0.1)	1 (0 to 2)	0.02 (0.01 to 0.03)	1 (0 to 2)	0.01 (0.01 to 0.03)
	Male	23 (16 to 31)	0.73 (0.53 to 0.97)	4 (1 to 7)	0.1 (0.02 to 0.2)	1 (0 to 2)	0.04 (0.02 to 0.06)	1 (0 to 2)	0.02 (0.01 to 0.04)
	Female	4 (2 to 6)	0.11 (0.07 to 0.16)	0 (0 to 0)	0 (0 to 0.01)	0 (0 to 0)	0 (0 to 0)	0 (0 to 1)	0.01 (0 to 0.02)
Southeast Asia	Both	4810 (3970 to 5860)	0.81 (0.67 to 0.98)	1310 (756 to 1830)	0.21 (0.12 to 0.29)	90 (44 to 152)	0.02 (0.01 to 0.03)	265 (110 to 498)	0.04 (0.02 to 0.07)
	Male	4600 (3820 to 5630)	1.73 (1.45 to 2.11)	1280 (744 to 1780)	0.45 (0.26 to 0.63)	86 (41 to 149)	0.04 (0.02 to 0.07)	231 (96 to 433)	0.07 (0.03 to 0.13)
	Female	202 (137 to 273)	0.07 (0.05 to 0.09)	28 (12 to 47)	0.01 (0 to 0.01)	4 (2 to 6)	0 (0 to 0)	34 (14 to 65)	0.01 (0 to 0.02)
Central Sub-Saharan Africa	Both	271 (188 to 371)	0.5 (0.35 to 0.67)	109 (48 to 178)	0.2 (0.09 to 0.32)	8 (3 to 40)	0.02 (0.01 to 0.09)	22 (9 to 43)	0.03 (0.01 to 0.07)
	Male	263 (182 to 359)	1.1 (0.77 to 1.47)	100 (45 to 162)	0.41 (0.18 to 0.65)	7 (2 to 38)	0.04 (0.01 to 0.22)	19 (7 to 37)	0.06 (0.02 to 0.12)
	Female	8 (5 to 13)	0.03 (0.02 to 0.04)	9 (3 to 17)	0.03 (0.01 to 0.06)	2 (0 to 3)	0.01 (0 to 0.01)	4 (1 to 7)	0.01 (0 to 0.02)
Eastern sub-Saharan Africa	Both	744 (524 to 1000)	0.45 (0.32 to 0.61)	364 (197 to 531)	0.21 (0.11 to 0.31)	25 (8 to 112)	0.02 (0.01 to 0.08)	83 (34 to 155)	0.04 (0.02 to 0.08)
	Male	696 (493 to 941)	0.89 (0.64 to 1.18)	332 (180 to 484)	0.4 (0.22 to 0.59)	20 (5 to 106)	0.03 (0.01 to 0.17)	67 (27 to 126)	0.07 (0.03 to 0.13)
	Female	48 (28 to 71)	0.06 (0.04 to 0.09)	32 (15 to 49)	0.04 (0.02 to 0.05)	5 (1 to 10)	0.01 (0 to 0.01)	17 (7 to 31)	0.02 (0.01 to 0.03)
Southern sub-Saharan Africa	Both	511 (415 to 600)	0.89 (0.73 to 1.04)	214 (127 to 292)	0.36 (0.21 to 0.49)	58 (32 to 87)	0.11 (0.06 to 0.17)	14 (6 to 27)	0.02 (0.01 to 0.04)
	Male	470 (383 to 550)	1.97 (1.62 to 2.3)	200 (120 to 274)	0.81 (0.48 to 1.1)	55 (29 to 83)	0.28 (0.15 to 0.42)	12 (5 to 24)	0.04 (0.02 to 0.08)
	Female	41 (30 to 52)	0.13 (0.09 to 0.16)	13 (7 to 20)	0.04 (0.02 to 0.06)	4 (1 to 6)	0.01 (0 to 0.02)	2 (1 to 4)	0.01 (0 to 0.01)

Western sub-Saharan Africa	Both	716 (502 to 959)	0.39 (0.27 to 0.51)	443 (247 to 651)	0.23 (0.13 to 0.34)	14 (6 to 31)	0.01 (0 to 0.02)	57 (23 to 107)	0.03 (0.01 to 0.05)
	Male	704 (492 to 944)	0.8 (0.56 to 1.05)	430 (241 to 633)	0.48 (0.27 to 0.7)	13 (6 to 30)	0.02 (0.01 to 0.04)	52 (21 to 99)	0.05 (0.02 to 0.09)
	Female	12 (7 to 17)	0.01 (0.01 to 0.02)	13 (6 to 22)	0.01 (0.01 to 0.02)	1 (0 to 1)	0 (0 to 0)	4 (2 to 8)	0 (0 to 0.01)
Afghanistan	Both	192 (120 to 275)	1.55 (1 to 2.15)	2 (0 to 5)	0.01 (0 to 0.03)	2 (0 to 9)	0.02 (0.01 to 0.1)	14 (5 to 27)	0.09 (0.03 to 0.16)
	Male	178 (110 to 257)	3.07 (1.96 to 4.33)	2 (0 to 5)	0.02 (0 to 0.06)	2 (0 to 8)	0.04 (0.01 to 0.19)	12 (4 to 24)	0.16 (0.06 to 0.3)
	Female	14 (7 to 23)	0.18 (0.1 to 0.29)	0 (0 to 0)	0 (0 to 0)	0 (0 to 2)	0.01 (0 to 0.02)	2 (1 to 4)	0.02 (0.01 to 0.04)
Albania	Both	73 (52 to 99)	1.68 (1.21 to 2.28)	19 (9 to 30)	0.44 (0.21 to 0.71)	1 (0 to 2)	0.02 (0.01 to 0.05)	1 (0 to 3)	0.03 (0.01 to 0.08)
	Male	68 (49 to 93)	3.35 (2.4 to 4.56)	18 (9 to 29)	0.91 (0.43 to 1.44)	1 (0 to 2)	0.03 (0.01 to 0.1)	1 (0 to 3)	0.06 (0.01 to 0.14)
	Female	4 (3 to 6)	0.2 (0.13 to 0.28)	0 (0 to 1)	0.02 (0.01 to 0.04)	0 (0 to 0)	0.01 (0 to 0.01)	0 (0 to 0)	0.01 (0 to 0.02)
Algeria	Both	323 (245 to 421)	1.04 (0.79 to 1.34)	18 (8 to 33)	0.05 (0.02 to 0.09)	1 (0 to 5)	0.01 (0 to 0.02)	10 (4 to 19)	0.03 (0.01 to 0.05)
	Male	317 (241 to 413)	1.95 (1.49 to 2.53)	18 (8 to 33)	0.1 (0.04 to 0.18)	1 (0 to 5)	0.01 (0 to 0.04)	9 (4 to 19)	0.05 (0.02 to 0.1)
	Female	6 (4 to 9)	0.04 (0.02 to 0.06)	0 (0 to 1)	0 (0 to 0)	0 (0 to 0)	0 (0 to 0)	0 (0 to 1)	0 (0 to 0)
American Samoa	Both	0 (0 to 0)	0.35 (0.27 to 0.44)	0 (0 to 0)	0.02 (0 to 0.05)	0 (0 to 0)	0.02 (0.01 to 0.03)	0 (0 to 0)	0.01 (0 to 0.02)
	Male	0 (0 to 0)	0.71 (0.55 to 0.88)	0 (0 to 0)	0.04 (-0.01 to 0.1)	0 (0 to 0)	0.04 (0.02 to 0.06)	0 (0 to 0)	0.02 (0.01 to 0.03)
	Female	0 (0 to 0)	0.04 (0.03 to 0.06)	0 (0 to 0)	0 (0 to 0)	0 (0 to 0)	0 (0 to 0)	0 (0 to 0)	0 (0 to 0.01)
Andorra	Both	1 (1 to 1)	0.74 (0.54 to 0.97)	1 (0 to 1)	0.36 (0.2 to 0.51)	0 (0 to 0)	0.15 (0.08 to 0.26)	0 (0 to 0)	0.02 (0.01 to 0.05)
	Male	1 (1 to 1)	1.42 (1.03 to 1.86)	0 (0 to 1)	0.69 (0.39 to 0.98)	0 (0 to 0)	0.31 (0.15 to 0.52)	0 (0 to 0)	0.04 (0.01 to 0.1)
	Female	0 (0 to 0)	0.05 (0.03 to 0.07)	0 (0 to 0)	0.02 (0.01 to 0.03)	0 (0 to 0)	0 (0 to 0.01)	0 (0 to 0)	0 (0 to 0)
Angola	Both	94 (67 to 128)	0.85 (0.62 to 1.13)	48 (26 to 71)	0.42 (0.23 to 0.61)	2 (1 to 10)	0.03 (0.01 to 0.11)	6 (2 to 11)	0.04 (0.02 to 0.08)
	Male	91 (65 to 124)	1.86 (1.36 to 2.52)	44 (24 to 65)	0.87 (0.48 to 1.28)	2 (0 to 10)	0.05 (0.01 to 0.26)	5 (2 to 10)	0.08 (0.03 to 0.16)
	Female	3 (2 to 5)	0.05 (0.03 to 0.08)	4 (2 to 6)	0.06 (0.03 to 0.1)	0 (0 to 1)	0.01 (0 to 0.01)	1 (0 to 1)	0.01 (0 to 0.02)

Antigua and Barbuda	Both	1 (1 to 1)	0.89 (0.66 to 1.14)	0 (0 to 1)	0.34 (0.18 to 0.5)	0 (0 to 0)	0.02 (0.01 to 0.03)	0 (0 to 0)	0.05 (0.02 to 0.1)
	Male	1 (1 to 1)	1.88 (1.4 to 2.42)	0 (0 to 1)	0.71 (0.38 to 1.05)	0 (0 to 0)	0.04 (0.02 to 0.06)	0 (0 to 0)	0.11 (0.04 to 0.19)
	Female	0 (0 to 0)	0.02 (0.01 to 0.03)	0 (0 to 0)	0.01 (0 to 0.01)	0 (0 to 0)	0 (0 to 0)	0 (0 to 0)	0 (0 to 0)
Argentina	Both	691 (568 to 811)	1.3 (1.07 to 1.52)	337 (210 to 455)	0.63 (0.4 to 0.85)	45 (23 to 72)	0.08 (0.04 to 0.13)	36 (15 to 66)	0.07 (0.03 to 0.13)
	Male	618 (506 to 729)	2.59 (2.11 to 3.06)	313 (197 to 417)	1.32 (0.83 to 1.76)	42 (20 to 68)	0.18 (0.09 to 0.3)	33 (13 to 60)	0.14 (0.05 to 0.25)
	Female	73 (55 to 91)	0.25 (0.19 to 0.3)	24 (12 to 38)	0.08 (0.04 to 0.13)	3 (1 to 6)	0.01 (0 to 0.02)	3 (1 to 6)	0.01 (0 to 0.02)
Armenia	Both	85 (69 to 104)	2 (1.62 to 2.43)	15 (7 to 25)	0.36 (0.16 to 0.57)	4 (2 to 7)	0.09 (0.05 to 0.16)	4 (2 to 7)	0.09 (0.04 to 0.16)
	Male	84 (68 to 103)	4.63 (3.76 to 5.61)	15 (6 to 24)	0.77 (0.34 to 1.24)	3 (2 to 6)	0.2 (0.1 to 0.35)	4 (1 to 7)	0.19 (0.07 to 0.35)
	Female	1 (1 to 1)	0.04 (0.02 to 0.06)	1 (0 to 1)	0.03 (0.02 to 0.06)	1 (0 to 1)	0.02 (0.01 to 0.04)	0 (0 to 0)	0.01 (0 to 0.02)
Australia	Both	122 (92 to 150)	0.3 (0.23 to 0.36)	87 (53 to 117)	0.21 (0.13 to 0.28)	48 (27 to 70)	0.11 (0.06 to 0.15)	6 (1 to 13)	0.01 (0 to 0.03)
	Male	107 (81 to 132)	0.55 (0.42 to 0.68)	81 (50 to 107)	0.42 (0.26 to 0.55)	46 (26 to 66)	0.22 (0.12 to 0.32)	5 (1 to 12)	0.03 (0.01 to 0.06)
	Female	15 (11 to 19)	0.07 (0.05 to 0.09)	6 (3 to 10)	0.03 (0.01 to 0.04)	2 (1 to 4)	0.01 (0 to 0.01)	1 (0 to 1)	0 (0 to 0.01)
Austria	Both	107 (92 to 122)	0.65 (0.56 to 0.74)	51 (31 to 67)	0.31 (0.19 to 0.41)	11 (6 to 18)	0.06 (0.03 to 0.09)	3 (1 to 8)	0.02 (0.01 to 0.05)
	Male	97 (83 to 110)	1.27 (1.09 to 1.45)	47 (29 to 62)	0.62 (0.38 to 0.82)	11 (6 to 17)	0.14 (0.07 to 0.21)	3 (1 to 7)	0.04 (0.01 to 0.1)
	Female	10 (8 to 13)	0.12 (0.09 to 0.15)	4 (2 to 6)	0.04 (0.02 to 0.06)	0 (0 to 1)	0 (0 to 0.01)	0 (0 to 1)	0 (0 to 0.01)
Azerbaijan	Both	170 (126 to 235)	1.7 (1.26 to 2.31)	57 (31 to 87)	0.55 (0.29 to 0.83)	1 (0 to 2)	0.02 (0.01 to 0.03)	8 (3 to 15)	0.06 (0.02 to 0.13)
	Male	169 (124 to 234)	3.8 (2.82 to 5.16)	54 (30 to 82)	1.14 (0.62 to 1.73)	1 (0 to 2)	0.03 (0.01 to 0.06)	7 (2 to 13)	0.12 (0.04 to 0.23)
	Female	2 (1 to 3)	0.03 (0.02 to 0.05)	3 (1 to 7)	0.06 (0.02 to 0.12)	0 (0 to 1)	0.01 (0 to 0.01)	1 (0 to 2)	0.02 (0.01 to 0.04)
Bahamas	Both	5 (4 to 7)	1.26 (0.91 to 1.7)	2 (1 to 3)	0.46 (0.14 to 0.77)	0 (0 to 0)	0.06 (0.03 to 0.1)	0 (0 to 1)	0.07 (0.03 to 0.13)
	Male	5 (3 to 6)	2.73 (1.98 to 3.64)	2 (1 to 3)	0.98 (0.3 to 1.64)	0 (0 to 0)	0.13 (0.06 to 0.24)	0 (0 to 1)	0.14 (0.05 to 0.27)
	Female	0 (0 to 0)	0.08 (0.05 to 0.12)	0 (0 to 0)	0.02 (0 to 0.04)	0 (0 to 0)	0 (0 to 0)	0 (0 to 0)	0.01 (0 to 0.02)

Bahrain	Both	6 (4 to 7)	0.9 (0.62 to 1.19)	0 (0 to 1)	0.04 (0.02 to 0.08)	0 (0 to 1)	0.11 (0.04 to 0.21)	0 (0 to 0)	0.02 (0.01 to 0.04)
	Male	5 (4 to 7)	1.69 (1.14 to 2.25)	0 (0 to 1)	0.07 (0.03 to 0.13)	0 (0 to 1)	0.23 (0.07 to 0.42)	0 (0 to 0)	0.03 (0.01 to 0.06)
	Female	0 (0 to 0)	0.04 (0.03 to 0.06)	0 (0 to 0)	0 (0 to 0)	0 (0 to 0)	0 (0 to 0)	0 (0 to 0)	0 (0 to 0)
Bangladesh	Both	1850 (1310 to 2770)	1.44 (1.03 to 2.13)	54 (4 to 117)	0.04 (0 to 0.09)	22 (8 to 61)	0.02 (0.01 to 0.05)	109 (41 to 220)	0.08 (0.03 to 0.16)
	Male	1820 (1280 to 2710)	2.71 (1.93 to 4.01)	53 (4 to 114)	0.08 (0.01 to 0.17)	20 (6 to 59)	0.03 (0.01 to 0.09)	99 (37 to 204)	0.14 (0.05 to 0.29)
	Female	39 (21 to 62)	0.06 (0.03 to 0.1)	2 (0 to 4)	0 (0 to 0.01)	2 (1 to 5)	0 (0 to 0.01)	9 (4 to 19)	0.01 (0.01 to 0.03)
Barbados	Both	4 (3 to 5)	0.72 (0.54 to 0.91)	2 (1 to 3)	0.36 (0.21 to 0.51)	0 (0 to 0)	0.03 (0.01 to 0.05)	0 (0 to 0)	0.04 (0.02 to 0.08)
	Male	4 (3 to 4)	1.57 (1.19 to 1.98)	2 (1 to 2)	0.78 (0.45 to 1.09)	0 (0 to 0)	0.07 (0.03 to 0.12)	0 (0 to 0)	0.09 (0.03 to 0.16)
	Female	0 (0 to 0)	0.03 (0.02 to 0.05)	0 (0 to 0)	0.02 (0.01 to 0.04)	0 (0 to 0)	0 (0 to 0)	0 (0 to 0)	0.01 (0 to 0.02)
Belarus	Both	287 (215 to 383)	1.82 (1.37 to 2.42)	136 (82 to 197)	0.87 (0.52 to 1.26)	4 (2 to 7)	0.02 (0.01 to 0.04)	7 (2 to 18)	0.05 (0.01 to 0.11)
	Male	284 (214 to 380)	4.47 (3.38 to 5.95)	135 (81 to 194)	2.1 (1.27 to 3.04)	3 (1 to 7)	0.06 (0.02 to 0.11)	7 (1 to 17)	0.1 (0.02 to 0.25)
	Female	2 (1 to 4)	0.03 (0.02 to 0.04)	2 (1 to 3)	0.02 (0.01 to 0.03)	0 (0 to 1)	0 (0 to 0.01)	0 (0 to 1)	0 (0 to 0.01)
Belgium	Both	176 (146 to 202)	0.83 (0.7 to 0.95)	85 (53 to 113)	0.4 (0.25 to 0.54)	35 (21 to 51)	0.14 (0.08 to 0.21)	5 (1 to 10)	0.02 (0.01 to 0.05)
	Male	154 (127 to 178)	1.56 (1.3 to 1.79)	76 (47 to 100)	0.77 (0.48 to 1.02)	34 (19 to 50)	0.32 (0.18 to 0.47)	4 (1 to 9)	0.04 (0.01 to 0.1)
	Female	22 (17 to 27)	0.2 (0.16 to 0.24)	9 (5 to 14)	0.08 (0.05 to 0.12)	1 (1 to 2)	0.01 (0 to 0.02)	0 (0 to 1)	0.01 (0 to 0.01)
Belize	Both	3 (2 to 3)	1 (0.79 to 1.24)	1 (1 to 1)	0.35 (0.19 to 0.51)	0 (0 to 0)	0.08 (0.04 to 0.13)	0 (0 to 0)	0.07 (0.03 to 0.13)
	Male	3 (2 to 3)	1.92 (1.52 to 2.36)	1 (1 to 1)	0.68 (0.38 to 0.99)	0 (0 to 0)	0.15 (0.07 to 0.25)	0 (0 to 0)	0.13 (0.05 to 0.24)
	Female	0 (0 to 0)	0.06 (0.04 to 0.09)	0 (0 to 0)	0.01 (0 to 0.02)	0 (0 to 0)	0 (0 to 0.01)	0 (0 to 0)	0.01 (0 to 0.01)
Benin	Both	20 (13 to 28)	0.43 (0.29 to 0.6)	8 (3 to 13)	0.16 (0.07 to 0.26)	0 (0 to 1)	0.01 (0 to 0.02)	2 (1 to 3)	0.03 (0.01 to 0.06)
	Male	20 (13 to 27)	0.92 (0.62 to 1.27)	7 (3 to 12)	0.32 (0.14 to 0.54)	0 (0 to 1)	0.02 (0.01 to 0.05)	2 (1 to 3)	0.06 (0.02 to 0.12)
	Female	0 (0 to 1)	0.01 (0.01 to 0.02)	0 (0 to 1)	0.01 (0 to 0.03)	0 (0 to 0)	0 (0 to 0)	0 (0 to 0)	0.01 (0 to 0.01)

Bermuda	Both	1 (1 to 1)	0.89 (0.64 to 1.15)	1 (0 to 1)	0.42 (0.23 to 0.61)	0 (0 to 0)	0.11 (0.05 to 0.17)	0 (0 to 0)	0.05 (0.02 to 0.09)
	Male	1 (1 to 1)	1.92 (1.38 to 2.46)	1 (0 to 1)	0.91 (0.5 to 1.32)	0 (0 to 0)	0.26 (0.13 to 0.41)	0 (0 to 0)	0.1 (0.04 to 0.19)
	Female	0 (0 to 0)	0.06 (0.04 to 0.09)	0 (0 to 0)	0.02 (0.01 to 0.03)	0 (0 to 0)	0 (0 to 0)	0 (0 to 0)	0 (0 to 0.01)
Bhutan	Both	6 (4 to 9)	1.08 (0.67 to 1.62)	1 (0 to 1)	0.12 (0.03 to 0.24)	0 (0 to 0)	0.05 (0.02 to 0.09)	0 (0 to 1)	0.06 (0.02 to 0.13)
	Male	5 (3 to 8)	1.99 (1.23 to 2.99)	1 (0 to 1)	0.23 (0.06 to 0.46)	0 (0 to 0)	0.09 (0.03 to 0.16)	0 (0 to 1)	0.1 (0.04 to 0.2)
	Female	0 (0 to 1)	0.14 (0.08 to 0.22)	0 (0 to 0)	0 (0 to 0.01)	0 (0 to 0)	0.01 (0 to 0.02)	0 (0 to 0)	0.03 (0.01 to 0.06)
Bolivia	Both	44 (29 to 63)	0.52 (0.35 to 0.75)	16 (7 to 28)	0.18 (0.07 to 0.31)	5 (2 to 9)	0.07 (0.03 to 0.12)	4 (2 to 9)	0.05 (0.02 to 0.09)
	Male	42 (28 to 60)	1.05 (0.7 to 1.51)	15 (7 to 25)	0.36 (0.15 to 0.61)	5 (2 to 9)	0.13 (0.05 to 0.26)	4 (1 to 7)	0.08 (0.03 to 0.16)
	Female	2 (1 to 4)	0.05 (0.02 to 0.08)	1 (0 to 2)	0.02 (0 to 0.05)	0 (0 to 1)	0.01 (0 to 0.02)	1 (0 to 2)	0.02 (0.01 to 0.03)
Bosnia and Herzegovina	Both	128 (99 to 165)	2.12 (1.63 to 2.73)	41 (23 to 60)	0.68 (0.38 to 1)	3 (1 to 5)	0.04 (0.02 to 0.08)	3 (1 to 6)	0.04 (0.01 to 0.1)
	Male	118 (90 to 153)	4.4 (3.39 to 5.67)	40 (23 to 59)	1.48 (0.83 to 2.18)	3 (1 to 5)	0.1 (0.04 to 0.19)	2 (1 to 6)	0.08 (0.02 to 0.2)
	Female	10 (7 to 13)	0.3 (0.22 to 0.4)	1 (0 to 2)	0.03 (0.01 to 0.05)	0 (0 to 0)	0 (0 to 0)	0 (0 to 0)	0.01 (0 to 0.01)
Botswana	Both	18 (12 to 25)	1.29 (0.91 to 1.8)	7 (4 to 11)	0.46 (0.24 to 0.72)	2 (1 to 4)	0.18 (0.08 to 0.33)	1 (0 to 1)	0.03 (0.01 to 0.06)
	Male	17 (11 to 24)	2.85 (2.04 to 3.97)	6 (3 to 10)	1.03 (0.54 to 1.6)	2 (1 to 4)	0.46 (0.2 to 0.81)	0 (0 to 1)	0.06 (0.02 to 0.12)
	Female	1 (1 to 2)	0.17 (0.1 to 0.27)	0 (0 to 1)	0.04 (0.01 to 0.07)	0 (0 to 0)	0.01 (0 to 0.03)	0 (0 to 0)	0.01 (0 to 0.02)
Brazil	Both	3130 (2570 to 3670)	1.3 (1.06 to 1.53)	1160 (659 to 1590)	0.47 (0.27 to 0.65)	151 (85 to 231)	0.07 (0.04 to 0.1)	196 (82 to 363)	0.08 (0.03 to 0.15)
	Male	2830 (2330 to 3300)	2.61 (2.13 to 3.06)	1110 (638 to 1530)	0.99 (0.57 to 1.37)	132 (68 to 209)	0.13 (0.07 to 0.21)	178 (74 to 328)	0.15 (0.06 to 0.28)
	Female	303 (222 to 383)	0.23 (0.17 to 0.29)	48 (20 to 78)	0.04 (0.02 to 0.06)	19 (9 to 32)	0.01 (0.01 to 0.02)	18 (7 to 34)	0.01 (0.01 to 0.03)
Brunei	Both	2 (1 to 2)	0.73 (0.57 to 0.92)	0 (0 to 0)	0.01 (-0.01 to 0.04)	0 (0 to 0)	0.05 (0.03 to 0.09)	0 (0 to 0)	0.01 (0 to 0.03)
	Male	2 (1 to 2)	1.61 (1.22 to 2.05)	0 (0 to 0)	0.02 (-0.02 to 0.09)	0 (0 to 0)	0.15 (0.07 to 0.26)	0 (0 to 0)	0.03 (0.01 to 0.06)
	Female	0 (0 to 0)	0.18 (0.12 to 0.25)	0 (0 to 0)	0 (0 to 0.01)	0 (0 to 0)	0.01 (0 to 0.02)	0 (0 to 0)	0 (0 to 0.01)

Bulgaria	Both	312 (234 to 401)	2.41 (1.78 to 3.1)	154 (92 to 215)	1.19 (0.71 to 1.66)	3 (1 to 5)	0.02 (0.01 to 0.03)	9 (2 to 21)	0.08 (0.02 to 0.18)
	Male	302 (226 to 387)	5.06 (3.77 to 6.5)	150 (90 to 210)	2.53 (1.5 to 3.53)	2 (1 to 5)	0.04 (0.02 to 0.07)	9 (2 to 20)	0.15 (0.03 to 0.35)
	Female	10 (7 to 14)	0.15 (0.11 to 0.21)	3 (2 to 6)	0.05 (0.02 to 0.08)	0 (0 to 0)	0 (0 to 0)	1 (0 to 1)	0.01 (0 to 0.02)
Burkina Faso	Both	32 (19 to 49)	0.35 (0.21 to 0.52)	32 (16 to 50)	0.36 (0.18 to 0.56)	1 (0 to 2)	0.01 (0 to 0.02)	3 (1 to 6)	0.03 (0.01 to 0.06)
	Male	32 (19 to 48)	0.77 (0.46 to 1.14)	30 (15 to 47)	0.75 (0.37 to 1.15)	1 (0 to 2)	0.02 (0.01 to 0.05)	3 (1 to 6)	0.06 (0.02 to 0.12)
	Female	0 (0 to 1)	0.01 (0 to 0.01)	2 (1 to 4)	0.05 (0.02 to 0.08)	0 (0 to 0)	0 (0 to 0)	0 (0 to 1)	0.01 (0 to 0.01)
Burundi	Both	27 (16 to 42)	0.57 (0.34 to 0.85)	18 (9 to 29)	0.37 (0.19 to 0.58)	1 (0 to 3)	0.02 (0 to 0.09)	3 (1 to 7)	0.06 (0.02 to 0.12)
	Male	26 (16 to 40)	1.05 (0.64 to 1.56)	17 (9 to 28)	0.67 (0.34 to 1.06)	1 (0 to 3)	0.03 (0 to 0.17)	3 (1 to 5)	0.08 (0.03 to 0.17)
	Female	1 (0 to 2)	0.05 (0.02 to 0.08)	1 (0 to 2)	0.04 (0.02 to 0.07)	0 (0 to 0)	0.01 (0 to 0.02)	1 (0 to 2)	0.03 (0.01 to 0.06)
Cabo Verde	Both	2 (1 to 3)	0.46 (0.32 to 0.62)	1 (1 to 2)	0.27 (0.15 to 0.4)	0 (0 to 0)	0.01 (0 to 0.01)	0 (0 to 0)	0.02 (0.01 to 0.04)
	Male	2 (1 to 3)	1.07 (0.75 to 1.46)	1 (1 to 2)	0.61 (0.34 to 0.9)	0 (0 to 0)	0.02 (0.01 to 0.04)	0 (0 to 0)	0.04 (0.01 to 0.08)
	Female	0 (0 to 0)	0.02 (0.01 to 0.03)	0 (0 to 0)	0.02 (0.01 to 0.03)	0 (0 to 0)	0 (0 to 0)	0 (0 to 0)	0 (0 to 0)
Cambodia	Both	137 (101 to 182)	1.22 (0.89 to 1.63)	45 (24 to 69)	0.38 (0.19 to 0.58)	3 (1 to 6)	0.03 (0.01 to 0.06)	8 (3 to 15)	0.06 (0.02 to 0.11)
	Male	129 (93 to 174)	2.86 (2.08 to 3.89)	43 (23 to 65)	0.89 (0.46 to 1.34)	3 (1 to 6)	0.08 (0.03 to 0.15)	6 (2 to 12)	0.1 (0.04 to 0.21)
	Female	8 (5 to 12)	0.13 (0.08 to 0.19)	2 (1 to 4)	0.03 (0.01 to 0.05)	0 (0 to 0)	0 (0 to 0.01)	2 (1 to 3)	0.02 (0.01 to 0.04)
Cameroon	Both	69 (44 to 102)	0.57 (0.36 to 0.84)	44 (24 to 67)	0.37 (0.2 to 0.56)	2 (1 to 4)	0.02 (0.01 to 0.04)	5 (2 to 10)	0.04 (0.01 to 0.07)
	Male	68 (43 to 101)	1.17 (0.75 to 1.71)	42 (22 to 64)	0.73 (0.4 to 1.12)	2 (1 to 4)	0.04 (0.02 to 0.09)	5 (2 to 9)	0.07 (0.03 to 0.14)
	Female	1 (0 to 1)	0.02 (0.01 to 0.03)	2 (1 to 3)	0.03 (0.01 to 0.06)	0 (0 to 0)	0 (0 to 0)	0 (0 to 1)	0.01 (0 to 0.01)
Canada	Both	351 (278 to 417)	0.5 (0.4 to 0.59)	134 (77 to 186)	0.2 (0.11 to 0.27)	69 (38 to 100)	0.09 (0.05 to 0.13)	12 (3 to 26)	0.02 (0 to 0.04)
	Male	299 (236 to 356)	0.93 (0.73 to 1.1)	118 (69 to 164)	0.37 (0.22 to 0.51)	66 (36 to 97)	0.2 (0.11 to 0.3)	10 (2 to 22)	0.03 (0.01 to 0.07)
	Female	53 (40 to 67)	0.14 (0.11 to 0.18)	15 (7 to 25)	0.04 (0.02 to 0.07)	3 (1 to 5)	0.01 (0 to 0.01)	2 (0 to 4)	0.01 (0 to 0.01)

Central Africa Republic	Both	15 (9 to 23)	0.65 (0.41 to 0.96)	5 (1 to 10)	0.21 (0.05 to 0.42)	1 (0 to 2)	0.03 (0.01 to 0.11)	1 (0 to 3)	0.05 (0.02 to 0.1)
	Male	15 (9 to 22)	1.42 (0.93 to 2.11)	5 (1 to 10)	0.44 (0.11 to 0.86)	0 (0 to 2)	0.07 (0.02 to 0.26)	1 (0 to 2)	0.09 (0.03 to 0.17)
	Female	0 (0 to 1)	0.04 (0.02 to 0.06)	0 (0 to 1)	0.03 (0 to 0.07)	0 (0 to 0)	0.01 (0 to 0.02)	0 (0 to 0)	0.01 (0 to 0.03)
Chad	Both	28 (17 to 39)	0.52 (0.34 to 0.73)	9 (3 to 16)	0.16 (0.05 to 0.28)	0 (0 to 1)	0.01 (0 to 0.02)	2 (1 to 4)	0.03 (0.01 to 0.06)
	Male	27 (17 to 38)	0.94 (0.61 to 1.32)	8 (3 to 14)	0.27 (0.08 to 0.48)	0 (0 to 1)	0.01 (0 to 0.04)	2 (1 to 3)	0.05 (0.02 to 0.1)
	Female	1 (0 to 1)	0.02 (0.01 to 0.04)	1 (0 to 1)	0.02 (0 to 0.04)	0 (0 to 0)	0 (0 to 0)	0 (0 to 0)	0.01 (0 to 0.01)
Chile	Both	77 (56 to 100)	0.32 (0.23 to 0.41)	53 (32 to 73)	0.22 (0.13 to 0.3)	7 (4 to 11)	0.03 (0.01 to 0.05)	5 (2 to 9)	0.02 (0.01 to 0.04)
	Male	70 (50 to 91)	0.64 (0.46 to 0.84)	49 (30 to 66)	0.45 (0.27 to 0.62)	7 (3 to 11)	0.07 (0.03 to 0.11)	5 (2 to 8)	0.04 (0.02 to 0.07)
	Female	8 (5 to 10)	0.06 (0.04 to 0.08)	4 (2 to 7)	0.03 (0.02 to 0.05)	0 (0 to 1)	0 (0 to 0)	1 (0 to 1)	0 (0 to 0.01)
China	Both	15300 (12100 to 18900)	0.76 (0.6 to 0.93)	4680 (2700 to 6860)	0.23 (0.13 to 0.33)	229 (121 to 370)	0.01 (0.01 to 0.02)	755 (313 to 1460)	0.03 (0.01 to 0.07)
	Male	14400 (11300 to 17800)	1.55 (1.23 to 1.9)	4540 (2630 to 6680)	0.47 (0.27 to 0.69)	190 (89 to 321)	0.03 (0.01 to 0.04)	627 (258 to 1240)	0.06 (0.02 to 0.11)
	Female	957 (632 to 1320)	0.09 (0.06 to 0.13)	137 (58 to 230)	0.01 (0.01 to 0.02)	39 (16 to 66)	0 (0 to 0.01)	128 (53 to 245)	0.01 (0 to 0.02)
Colombia	Both	189 (123 to 276)	0.35 (0.23 to 0.52)	74 (36 to 119)	0.14 (0.07 to 0.23)	9 (4 to 17)	0.02 (0.01 to 0.03)	18 (7 to 33)	0.03 (0.01 to 0.06)
	Male	164 (106 to 240)	0.68 (0.44 to 1)	71 (34 to 111)	0.29 (0.14 to 0.46)	8 (3 to 16)	0.03 (0.01 to 0.07)	15 (6 to 27)	0.06 (0.02 to 0.11)
	Female	25 (15 to 40)	0.09 (0.05 to 0.14)	4 (1 to 8)	0.01 (0 to 0.03)	1 (0 to 2)	0 (0 to 0.01)	3 (1 to 6)	0.01 (0 to 0.02)
Comoros	Both	2 (1 to 3)	0.44 (0.3 to 0.63)	0 (0 to 0)	0.03 (0 to 0.06)	0 (0 to 0)	0.02 (0 to 0.06)	0 (0 to 0)	0.03 (0.01 to 0.07)
	Male	2 (1 to 3)	0.93 (0.63 to 1.3)	0 (0 to 0)	0.05 (0 to 0.12)	0 (0 to 0)	0.03 (0.01 to 0.14)	0 (0 to 0)	0.05 (0.02 to 0.11)
	Female	0 (0 to 0)	0.05 (0.02 to 0.08)	0 (0 to 0)	0.01 (0 to 0.02)	0 (0 to 0)	0.01 (0 to 0.02)	0 (0 to 0)	0.02 (0.01 to 0.03)
Congo	Both	17 (12 to 23)	0.69 (0.48 to 0.94)	8 (3 to 13)	0.29 (0.12 to 0.49)	1 (0 to 2)	0.03 (0.01 to 0.12)	1 (0 to 2)	0.03 (0.01 to 0.06)
	Male	17 (11 to 23)	1.45 (1.02 to 2)	7 (3 to 12)	0.55 (0.23 to 0.93)	1 (0 to 2)	0.06 (0.02 to 0.26)	1 (0 to 2)	0.05 (0.02 to 0.1)
	Female	0 (0 to 1)	0.02 (0.01 to 0.04)	1 (0 to 2)	0.06 (0.02 to 0.11)	0 (0 to 0)	0.01 (0 to 0.02)	0 (0 to 0)	0.01 (0 to 0.02)

Cook Islands	Both	0 (0 to 0)	0.29 (0.22 to 0.36)	0 (0 to 0)	0.12 (0.06 to 0.17)	0 (0 to 0)	0.01 (0 to 0.02)	0 (0 to 0)	0.01 (0 to 0.02)
	Male	0 (0 to 0)	0.55 (0.41 to 0.71)	0 (0 to 0)	0.24 (0.12 to 0.35)	0 (0 to 0)	0.02 (0.01 to 0.04)	0 (0 to 0)	0.02 (0.01 to 0.03)
	Female	0 (0 to 0)	0.04 (0.03 to 0.07)	0 (0 to 0)	0 (0 to 0.01)	0 (0 to 0)	0 (0 to 0)	0 (0 to 0)	0 (0 to 0.01)
Costa Rica	Both	30 (22 to 41)	0.6 (0.42 to 0.8)	9 (4 to 14)	0.18 (0.08 to 0.28)	1 (0 to 1)	0.01 (0.01 to 0.03)	2 (1 to 4)	0.04 (0.01 to 0.07)
	Male	29 (21 to 38)	1.25 (0.88 to 1.66)	9 (4 to 14)	0.38 (0.18 to 0.59)	1 (0 to 1)	0.03 (0.01 to 0.06)	2 (1 to 3)	0.07 (0.03 to 0.13)
	Female	1 (1 to 2)	0.05 (0.03 to 0.08)	0 (0 to 0)	0.01 (0 to 0.02)	0 (0 to 0)	0 (0 to 0)	0 (0 to 0)	0 (0 to 0.01)
Croatia	Both	156 (120 to 200)	1.85 (1.42 to 2.39)	66 (37 to 96)	0.79 (0.45 to 1.15)	21 (10 to 37)	0.23 (0.11 to 0.4)	3 (1 to 8)	0.04 (0.01 to 0.1)
	Male	148 (114 to 191)	3.99 (3.07 to 5.12)	65 (36 to 94)	1.75 (0.99 to 2.54)	21 (10 to 37)	0.54 (0.25 to 0.93)	3 (1 to 7)	0.09 (0.02 to 0.2)
	Female	8 (6 to 11)	0.17 (0.12 to 0.23)	1 (0 to 3)	0.03 (0.01 to 0.06)	0 (0 to 1)	0 (0 to 0.01)	0 (0 to 0)	0 (0 to 0.01)
Cuba	Both	702 (551 to 883)	3.68 (2.87 to 4.63)	187 (100 to 282)	0.99 (0.53 to 1.5)	9 (4 to 17)	0.05 (0.02 to 0.08)	36 (15 to 67)	0.19 (0.08 to 0.36)
	Male	646 (505 to 812)	7.19 (5.62 to 9.07)	180 (96 to 271)	2.01 (1.07 to 3.03)	9 (4 to 16)	0.1 (0.04 to 0.18)	33 (14 to 62)	0.36 (0.15 to 0.7)
	Female	56 (40 to 75)	0.55 (0.39 to 0.74)	7 (3 to 12)	0.07 (0.03 to 0.12)	1 (0 to 1)	0.01 (0 to 0.01)	3 (1 to 6)	0.03 (0.01 to 0.07)
Cyprus	Both	12 (10 to 15)	0.63 (0.5 to 0.77)	5 (3 to 7)	0.27 (0.16 to 0.36)	2 (1 to 3)	0.08 (0.04 to 0.13)	0 (0 to 1)	0.01 (0 to 0.03)
	Male	12 (9 to 14)	1.29 (1.01 to 1.57)	5 (3 to 7)	0.56 (0.33 to 0.76)	2 (1 to 3)	0.18 (0.09 to 0.29)	0 (0 to 0)	0.02 (0.01 to 0.05)
	Female	1 (0 to 1)	0.06 (0.05 to 0.09)	0 (0 to 0)	0.02 (0.01 to 0.03)	0 (0 to 0)	0 (0 to 0.01)	0 (0 to 0)	0 (0 to 0)
Czechia	Both	202 (158 to 255)	1.02 (0.79 to 1.29)	106 (66 to 149)	0.54 (0.34 to 0.76)	7 (3 to 13)	0.03 (0.01 to 0.06)	7 (2 to 16)	0.04 (0.01 to 0.09)
	Male	188 (147 to 237)	2.07 (1.61 to 2.6)	100 (63 to 141)	1.11 (0.7 to 1.56)	7 (3 to 12)	0.07 (0.03 to 0.13)	7 (2 to 15)	0.07 (0.02 to 0.17)
	Female	14 (10 to 19)	0.13 (0.09 to 0.17)	6 (3 to 9)	0.05 (0.03 to 0.08)	0 (0 to 1)	0 (0 to 0.01)	1 (0 to 1)	0.01 (0 to 0.01)
Côte d'Ivoire	Both	72 (48 to 98)	0.69 (0.46 to 0.94)	35 (16 to 55)	0.32 (0.14 to 0.52)	1 (0 to 3)	0.01 (0.01 to 0.03)	4 (2 to 8)	0.03 (0.01 to 0.07)
	Male	70 (47 to 96)	1.3 (0.89 to 1.8)	33 (16 to 54)	0.6 (0.27 to 0.96)	1 (0 to 3)	0.03 (0.01 to 0.06)	4 (2 to 8)	0.06 (0.02 to 0.12)
	Female	2 (1 to 3)	0.04 (0.02 to 0.06)	1 (0 to 2)	0.02 (0.01 to 0.04)	0 (0 to 0)	0 (0 to 0)	0 (0 to 1)	0 (0 to 0.01)

North Korea	Both	220 (167 to 285)	0.67 (0.51 to 0.86)	61 (31 to 91)	0.18 (0.09 to 0.28)	4 (2 to 6)	0.01 (0.01 to 0.02)	14 (6 to 26)	0.04 (0.02 to 0.08)
	Male	207 (157 to 267)	1.54 (1.2 to 1.97)	59 (31 to 89)	0.42 (0.22 to 0.63)	3 (1 to 5)	0.02 (0.01 to 0.04)	11 (4 to 21)	0.07 (0.03 to 0.14)
	Female	14 (8 to 21)	0.07 (0.04 to 0.11)	2 (0 to 3)	0.01 (0 to 0.02)	1 (0 to 1)	0 (0 to 0.01)	3 (1 to 5)	0.01 (0.01 to 0.03)
Democratic Republic of the Congo	Both	136 (81 to 204)	0.37 (0.22 to 0.54)	42 (9 to 86)	0.11 (0.02 to 0.23)	5 (1 to 23)	0.02 (0 to 0.08)	14 (5 to 28)	0.03 (0.01 to 0.06)
	Male	131 (78 to 197)	0.8 (0.49 to 1.19)	38 (8 to 78)	0.23 (0.05 to 0.46)	4 (1 to 22)	0.03 (0.01 to 0.2)	11 (4 to 22)	0.05 (0.02 to 0.11)
	Female	4 (2 to 7)	0.02 (0.01 to 0.04)	4 (0 to 9)	0.02 (0 to 0.04)	1 (0 to 2)	0.01 (0 to 0.01)	3 (1 to 5)	0.01 (0 to 0.02)
Denmark	Both	78 (66 to 89)	0.69 (0.59 to 0.79)	34 (20 to 46)	0.3 (0.18 to 0.41)	14 (8 to 22)	0.12 (0.06 to 0.18)	2 (0 to 5)	0.02 (0 to 0.05)
	Male	65 (54 to 74)	1.23 (1.04 to 1.41)	29 (17 to 39)	0.55 (0.33 to 0.75)	14 (7 to 21)	0.24 (0.13 to 0.37)	2 (0 to 4)	0.03 (0.01 to 0.08)
	Female	14 (11 to 17)	0.22 (0.18 to 0.27)	5 (3 to 7)	0.08 (0.04 to 0.13)	1 (0 to 1)	0.01 (0 to 0.02)	0 (0 to 1)	0.01 (0 to 0.01)
Djibouti	Both	5 (3 to 7)	0.8 (0.54 to 1.19)	0 (0 to 1)	0.03 (-0.01 to 0.09)	0 (0 to 0)	0.02 (0.01 to 0.09)	0 (0 to 0)	0.03 (0.01 to 0.06)
	Male	5 (3 to 7)	1.43 (0.96 to 2.13)	0 (0 to 1)	0.05 (-0.02 to 0.15)	0 (0 to 0)	0.04 (0.01 to 0.17)	0 (0 to 0)	0.04 (0.02 to 0.09)
	Female	0 (0 to 0)	0.08 (0.04 to 0.13)	0 (0 to 0)	0 (0 to 0.01)	0 (0 to 0)	0.01 (0 to 0.02)	0 (0 to 0)	0.01 (0 to 0.02)
Dominica	Both	1 (1 to 1)	1.1 (0.76 to 1.51)	0 (0 to 1)	0.54 (0.28 to 0.8)	0 (0 to 0)	0.12 (0.05 to 0.2)	0 (0 to 0)	0.08 (0.03 to 0.15)
	Male	1 (1 to 1)	2.13 (1.48 to 2.93)	0 (0 to 1)	1.04 (0.55 to 1.54)	0 (0 to 0)	0.26 (0.12 to 0.45)	0 (0 to 0)	0.14 (0.05 to 0.26)
	Female	0 (0 to 0)	0.11 (0.07 to 0.17)	0 (0 to 0)	0.05 (0.02 to 0.08)	0 (0 to 0)	0 (0 to 0)	0 (0 to 0)	0.01 (0.01 to 0.03)
Dominican Republic	Both	125 (92 to 168)	1.39 (1.02 to 1.85)	35 (18 to 54)	0.37 (0.19 to 0.58)	2 (1 to 3)	0.02 (0.01 to 0.04)	7 (3 to 14)	0.08 (0.03 to 0.14)
	Male	106 (77 to 146)	2.45 (1.79 to 3.35)	33 (17 to 51)	0.72 (0.36 to 1.12)	2 (1 to 3)	0.04 (0.02 to 0.08)	7 (3 to 12)	0.14 (0.06 to 0.26)
	Female	19 (14 to 27)	0.41 (0.3 to 0.57)	2 (1 to 4)	0.04 (0.02 to 0.07)	0 (0 to 0)	0 (0 to 0.01)	1 (0 to 2)	0.02 (0.01 to 0.03)
Ecuador	Both	46 (31 to 63)	0.32 (0.22 to 0.43)	13 (6 to 21)	0.08 (0.04 to 0.13)	2 (1 to 4)	0.02 (0.01 to 0.03)	4 (1 to 8)	0.03 (0.01 to 0.05)
	Male	43 (29 to 59)	0.63 (0.43 to 0.86)	12 (6 to 20)	0.17 (0.08 to 0.27)	2 (1 to 3)	0.03 (0.01 to 0.06)	3 (1 to 7)	0.04 (0.02 to 0.09)
	Female	3 (2 to 5)	0.04 (0.02 to 0.06)	1 (0 to 1)	0.01 (0 to 0.01)	0 (0 to 0)	0 (0 to 0.01)	1 (0 to 1)	0.01 (0 to 0.01)

Egypt	Both	460 (325 to 655)	0.71 (0.51 to 1.05)	13 (3 to 28)	0.02 (0 to 0.04)	2 (0 to 8)	0 (0 to 0.02)	22 (8 to 43)	0.03 (0.01 to 0.06)
	Male	453 (319 to 649)	1.27 (0.89 to 1.87)	12 (3 to 27)	0.03 (0.01 to 0.07)	1 (0 to 8)	0.01 (0 to 0.03)	21 (8 to 42)	0.05 (0.02 to 0.1)
	Female	7 (4 to 12)	0.03 (0.02 to 0.05)	0 (0 to 1)	0 (0 to 0)	0 (0 to 1)	0 (0 to 0)	1 (0 to 2)	0 (0 to 0.01)
El Salvador	Both	17 (11 to 25)	0.29 (0.19 to 0.42)	5 (2 to 9)	0.09 (0.04 to 0.15)	0 (0 to 0)	0 (0 to 0)	2 (1 to 3)	0.03 (0.01 to 0.05)
	Male	16 (10 to 22)	0.63 (0.41 to 0.9)	5 (2 to 8)	0.21 (0.09 to 0.33)	0 (0 to 0)	0 (0 to 0.01)	1 (1 to 3)	0.06 (0.02 to 0.11)
	Female	1 (1 to 2)	0.04 (0.02 to 0.07)	0 (0 to 1)	0.01 (0 to 0.02)	0 (0 to 0)	0 (0 to 0)	0 (0 to 1)	0.01 (0 to 0.02)
Equatorial Guinea	Both	2 (1 to 3)	0.45 (0.27 to 0.72)	1 (1 to 2)	0.27 (0.14 to 0.45)	0 (0 to 0)	0.02 (0.01 to 0.1)	0 (0 to 0)	0.02 (0.01 to 0.04)
	Male	2 (1 to 3)	1.08 (0.66 to 1.73)	1 (1 to 2)	0.6 (0.29 to 1.01)	0 (0 to 0)	0.05 (0.01 to 0.24)	0 (0 to 0)	0.04 (0.01 to 0.08)
	Female	0 (0 to 0)	0.02 (0.01 to 0.04)	0 (0 to 0)	0.05 (0.02 to 0.09)	0 (0 to 0)	0.01 (0 to 0.02)	0 (0 to 0)	0 (0 to 0.01)
Eritrea	Both	13 (8 to 19)	0.41 (0.25 to 0.62)	3 (1 to 7)	0.1 (0.02 to 0.21)	1 (0 to 2)	0.02 (0.01 to 0.07)	2 (1 to 4)	0.05 (0.02 to 0.11)
	Male	12 (7 to 19)	0.95 (0.58 to 1.46)	3 (1 to 6)	0.21 (0.04 to 0.45)	0 (0 to 2)	0.05 (0.01 to 0.17)	1 (1 to 3)	0.09 (0.03 to 0.18)
	Female	0 (0 to 0)	0.01 (0.01 to 0.02)	0 (0 to 1)	0.02 (0 to 0.04)	0 (0 to 0)	0.01 (0 to 0.03)	0 (0 to 1)	0.03 (0.01 to 0.05)
Estonia	Both	24 (18 to 31)	0.99 (0.74 to 1.3)	12 (7 to 18)	0.52 (0.31 to 0.75)	1 (0 to 1)	0.02 (0.01 to 0.04)	0 (0 to 1)	0.02 (0.01 to 0.05)
	Male	23 (17 to 30)	2.34 (1.74 to 3.05)	12 (7 to 17)	1.24 (0.74 to 1.77)	1 (0 to 1)	0.07 (0.03 to 0.13)	0 (0 to 1)	0.05 (0.01 to 0.11)
	Female	1 (1 to 1)	0.07 (0.04 to 0.09)	0 (0 to 1)	0.03 (0.01 to 0.04)	0 (0 to 0)	0 (0 to 0)	0 (0 to 0)	0 (0 to 0.01)
eSwatini	Both	4 (3 to 6)	0.72 (0.47 to 1.04)	3 (2 to 5)	0.48 (0.27 to 0.72)	1 (1 to 2)	0.24 (0.1 to 0.41)	0 (0 to 0)	0.03 (0.01 to 0.07)
	Male	4 (2 to 6)	1.63 (1.07 to 2.43)	3 (2 to 4)	1.11 (0.63 to 1.66)	1 (1 to 2)	0.64 (0.28 to 1.1)	0 (0 to 0)	0.07 (0.02 to 0.14)
	Female	0 (0 to 1)	0.13 (0.07 to 0.21)	0 (0 to 0)	0.05 (0.02 to 0.09)	0 (0 to 0)	0.01 (0 to 0.03)	0 (0 to 0)	0.01 (0 to 0.01)
Ethiopia	Both	88 (53 to 135)	0.22 (0.13 to 0.33)	53 (22 to 94)	0.12 (0.05 to 0.22)	5 (1 to 24)	0.01 (0 to 0.07)	14 (6 to 27)	0.03 (0.01 to 0.06)
	Male	87 (52 to 134)	0.42 (0.25 to 0.64)	50 (21 to 88)	0.23 (0.1 to 0.41)	4 (1 to 24)	0.02 (0 to 0.13)	12 (5 to 24)	0.05 (0.02 to 0.1)
	Female	1 (1 to 2)	0.01 (0 to 0.01)	4 (1 to 7)	0.02 (0 to 0.03)	1 (0 to 2)	0 (0 to 0.01)	2 (1 to 4)	0.01 (0 to 0.02)

Fiji	Both	3 (2 to 4)	0.41 (0.28 to 0.54)	1 (0 to 1)	0.09 (0.04 to 0.14)	0 (0 to 0)	0.03 (0.01 to 0.05)	0 (0 to 0)	0.01 (0.01 to 0.03)
	Male	3 (2 to 3)	0.9 (0.63 to 1.18)	1 (0 to 1)	0.2 (0.09 to 0.32)	0 (0 to 0)	0.07 (0.03 to 0.12)	0 (0 to 0)	0.03 (0.01 to 0.05)
	Female	0 (0 to 0)	0.04 (0.02 to 0.07)	0 (0 to 0)	0 (0 to 0)	0 (0 to 0)	0 (0 to 0)	0 (0 to 0)	0 (0 to 0.01)
Finland	Both	28 (22 to 34)	0.24 (0.2 to 0.29)	13 (8 to 17)	0.11 (0.07 to 0.15)	6 (3 to 9)	0.04 (0.02 to 0.07)	1 (0 to 2)	0.01 (0 to 0.02)
	Male	26 (20 to 31)	0.48 (0.39 to 0.58)	12 (7 to 16)	0.23 (0.14 to 0.31)	6 (3 to 9)	0.1 (0.05 to 0.16)	1 (0 to 2)	0.02 (0 to 0.03)
	Female	2 (2 to 3)	0.04 (0.03 to 0.05)	1 (0 to 1)	0.01 (0.01 to 0.02)	0 (0 to 0)	0 (0 to 0)	0 (0 to 0)	0 (0 to 0)
France	Both	1230 (1030 to 1440)	1.01 (0.85 to 1.16)	682 (434 to 887)	0.56 (0.36 to 0.73)	272 (151 to 416)	0.19 (0.11 to 0.3)	35 (8 to 78)	0.03 (0.01 to 0.07)
	Male	1120 (933 to 1300)	1.98 (1.66 to 2.28)	625 (405 to 808)	1.12 (0.73 to 1.44)	263 (144 to 411)	0.43 (0.23 to 0.67)	32 (7 to 72)	0.06 (0.01 to 0.14)
	Female	118 (83 to 148)	0.18 (0.13 to 0.23)	56 (30 to 82)	0.08 (0.04 to 0.12)	8 (3 to 15)	0.01 (0 to 0.02)	3 (1 to 7)	0.01 (0 to 0.01)
Gabon	Both	7 (5 to 11)	0.68 (0.43 to 0.98)	5 (3 to 8)	0.48 (0.26 to 0.72)	0 (0 to 1)	0.04 (0.01 to 0.14)	0 (0 to 1)	0.03 (0.01 to 0.06)
	Male	7 (5 to 10)	1.45 (0.95 to 2.09)	5 (3 to 7)	0.98 (0.53 to 1.47)	0 (0 to 1)	0.09 (0.03 to 0.33)	0 (0 to 1)	0.05 (0.02 to 0.1)
	Female	0 (0 to 0)	0.02 (0.01 to 0.04)	0 (0 to 1)	0.06 (0.03 to 0.1)	0 (0 to 0)	0.01 (0 to 0.01)	0 (0 to 0)	0 (0 to 0.01)
The Gambia	Both	3 (2 to 4)	0.31 (0.22 to 0.41)	1 (0 to 2)	0.09 (0.04 to 0.16)	0 (0 to 0)	0.01 (0 to 0.01)	0 (0 to 0)	0.01 (0.01 to 0.03)
	Male	3 (2 to 4)	0.64 (0.45 to 0.85)	1 (0 to 1)	0.18 (0.08 to 0.3)	0 (0 to 0)	0.01 (0 to 0.03)	0 (0 to 0)	0.02 (0.01 to 0.05)
	Female	0 (0 to 0)	0.01 (0 to 0.01)	0 (0 to 0)	0.01 (0 to 0.02)	0 (0 to 0)	0 (0 to 0)	0 (0 to 0)	0 (0 to 0.01)
Georgia	Both	159 (126 to 194)	2.75 (2.19 to 3.36)	52 (31 to 74)	0.93 (0.55 to 1.32)	8 (4 to 14)	0.13 (0.06 to 0.23)	7 (3 to 13)	0.12 (0.05 to 0.24)
	Male	157 (124 to 192)	6.41 (5.14 to 7.81)	52 (31 to 74)	2.12 (1.25 to 3.03)	8 (3 to 14)	0.31 (0.14 to 0.54)	7 (3 to 13)	0.27 (0.1 to 0.51)
	Female	2 (1 to 3)	0.06 (0.04 to 0.1)	0 (0 to 1)	0.01 (0 to 0.02)	0 (0 to 0)	0 (0 to 0)	0 (0 to 1)	0.01 (0 to 0.02)
Germany	Both	1210 (1020 to 1400)	0.68 (0.58 to 0.77)	659 (423 to 840)	0.37 (0.24 to 0.47)	202 (108 to 308)	0.09 (0.05 to 0.15)	40 (9 to 91)	0.03 (0.01 to 0.06)
	Male	1080 (904 to 1250)	1.3 (1.1 to 1.5)	590 (379 to 754)	0.72 (0.47 to 0.91)	194 (103 to 299)	0.21 (0.11 to 0.33)	36 (8 to 81)	0.05 (0.01 to 0.11)
	Female	132 (95 to 163)	0.14 (0.1 to 0.18)	69 (39 to 97)	0.07 (0.04 to 0.1)	7 (3 to 12)	0.01 (0 to 0.01)	4 (1 to 10)	0.01 (0 to 0.01)

Ghana	Both	73 (46 to 106)	0.49 (0.32 to 0.7)	54 (27 to 85)	0.34 (0.17 to 0.53)	2 (1 to 4)	0.02 (0.01 to 0.03)	7 (3 to 13)	0.04 (0.01 to 0.07)
	Male	71 (45 to 104)	1.15 (0.74 to 1.62)	52 (26 to 82)	0.77 (0.38 to 1.2)	2 (1 to 4)	0.04 (0.02 to 0.07)	6 (2 to 12)	0.07 (0.03 to 0.15)
	Female	2 (1 to 3)	0.02 (0.01 to 0.04)	2 (0 to 3)	0.02 (0 to 0.03)	0 (0 to 0)	0 (0 to 0)	1 (0 to 1)	0.01 (0 to 0.01)
Greece	Both	331 (282 to 372)	1.42 (1.23 to 1.58)	128 (76 to 171)	0.56 (0.34 to 0.75)	19 (9 to 30)	0.07 (0.03 to 0.11)	5 (1 to 11)	0.03 (0.01 to 0.06)
	Male	306 (262 to 345)	2.87 (2.49 to 3.21)	125 (75 to 167)	1.19 (0.73 to 1.59)	19 (9 to 30)	0.15 (0.07 to 0.24)	5 (1 to 10)	0.05 (0.01 to 0.11)
	Female	24 (18 to 30)	0.18 (0.14 to 0.22)	3 (1 to 6)	0.02 (0.01 to 0.05)	1 (0 to 1)	0 (0 to 0.01)	0 (0 to 1)	0 (0 to 0.01)
Greenland	Both	1 (1 to 1)	1.1 (0.85 to 1.38)	0 (0 to 0)	0.34 (0.16 to 0.55)	0 (0 to 0)	0.16 (0.08 to 0.24)	0 (0 to 0)	0.03 (0.01 to 0.07)
	Male	1 (1 to 1)	1.96 (1.5 to 2.46)	0 (0 to 0)	0.61 (0.28 to 0.98)	0 (0 to 0)	0.31 (0.16 to 0.47)	0 (0 to 0)	0.05 (0.01 to 0.12)
	Female	0 (0 to 0)	0.14 (0.1 to 0.18)	0 (0 to 0)	0.03 (0.01 to 0.05)	0 (0 to 0)	0.01 (0 to 0.01)	0 (0 to 0)	0 (0 to 0.01)
Grenada	Both	1 (0 to 1)	0.57 (0.41 to 0.72)	0 (0 to 1)	0.31 (0.18 to 0.43)	0 (0 to 0)	0.02 (0.01 to 0.03)	0 (0 to 0)	0.04 (0.02 to 0.08)
	Male	1 (0 to 1)	1.16 (0.85 to 1.46)	0 (0 to 0)	0.63 (0.36 to 0.88)	0 (0 to 0)	0.05 (0.02 to 0.08)	0 (0 to 0)	0.08 (0.03 to 0.15)
	Female	0 (0 to 0)	0.06 (0.03 to 0.08)	0 (0 to 0)	0.03 (0.01 to 0.05)	0 (0 to 0)	0 (0 to 0.01)	0 (0 to 0)	0.01 (0 to 0.02)
Guam	Both	0 (0 to 0)	0.19 (0.14 to 0.26)	0 (0 to 0)	0.03 (0 to 0.08)	0 (0 to 0)	0.01 (0 to 0.01)	0 (0 to 0)	0.01 (0 to 0.01)
	Male	0 (0 to 0)	0.35 (0.25 to 0.48)	0 (0 to 0)	0.06 (0 to 0.15)	0 (0 to 0)	0.02 (0.01 to 0.03)	0 (0 to 0)	0.01 (0 to 0.02)
	Female	0 (0 to 0)	0.05 (0.03 to 0.08)	0 (0 to 0)	0 (0 to 0.01)	0 (0 to 0)	0 (0 to 0)	0 (0 to 0)	0 (0 to 0.01)
Guatemala	Both	29 (19 to 40)	0.27 (0.18 to 0.38)	6 (3 to 10)	0.05 (0.02 to 0.09)	1 (0 to 1)	0.01 (0 to 0.01)	3 (1 to 5)	0.02 (0.01 to 0.05)
	Male	26 (17 to 35)	0.55 (0.38 to 0.75)	5 (2 to 9)	0.1 (0.04 to 0.17)	0 (0 to 1)	0.01 (0.01 to 0.02)	2 (1 to 4)	0.04 (0.02 to 0.08)
	Female	3 (2 to 5)	0.05 (0.03 to 0.09)	1 (0 to 1)	0.01 (0 to 0.02)	0 (0 to 0)	0 (0 to 0)	1 (0 to 1)	0.01 (0 to 0.02)
Guinea	Both	36 (24 to 50)	0.67 (0.44 to 0.94)	5 (2 to 10)	0.1 (0.03 to 0.18)	1 (0 to 1)	0.01 (0 to 0.03)	2 (1 to 3)	0.03 (0.01 to 0.06)
	Male	35 (23 to 49)	1.29 (0.86 to 1.81)	5 (2 to 10)	0.18 (0.06 to 0.34)	1 (0 to 1)	0.02 (0.01 to 0.05)	2 (1 to 3)	0.05 (0.02 to 0.1)
	Female	1 (0 to 1)	0.03 (0.01 to 0.04)	0 (0 to 0)	0.01 (0 to 0.01)	0 (0 to 0)	0 (0 to 0)	0 (0 to 0)	0.01 (0 to 0.01)

Guinea-Bissau	Both	4 (2 to 6)	0.54 (0.33 to 0.78)	2 (1 to 4)	0.28 (0.12 to 0.49)	0 (0 to 0)	0.02 (0.01 to 0.05)	0 (0 to 1)	0.04 (0.02 to 0.09)
	Male	4 (2 to 6)	1.17 (0.72 to 1.7)	2 (1 to 4)	0.59 (0.25 to 1.03)	0 (0 to 0)	0.05 (0.02 to 0.1)	0 (0 to 1)	0.09 (0.03 to 0.17)
	Female	0 (0 to 0)	0.01 (0.01 to 0.02)	0 (0 to 0)	0.02 (0.01 to 0.04)	0 (0 to 0)	0 (0 to 0)	0 (0 to 0)	0.01 (0 to 0.01)
Guyana	Both	4 (3 to 5)	0.57 (0.39 to 0.8)	2 (1 to 3)	0.31 (0.16 to 0.47)	0 (0 to 0)	0.02 (0.01 to 0.04)	0 (0 to 1)	0.05 (0.02 to 0.09)
	Male	4 (2 to 5)	1.17 (0.8 to 1.63)	2 (1 to 3)	0.66 (0.35 to 0.98)	0 (0 to 0)	0.05 (0.02 to 0.09)	0 (0 to 1)	0.09 (0.03 to 0.17)
	Female	0 (0 to 0)	0.05 (0.03 to 0.07)	0 (0 to 0)	0.01 (0 to 0.02)	0 (0 to 0)	0 (0 to 0.01)	0 (0 to 0)	0.01 (0 to 0.01)
Haiti	Both	47 (25 to 74)	0.67 (0.36 to 1.05)	38 (18 to 61)	0.53 (0.24 to 0.86)	5 (1 to 10)	0.08 (0.03 to 0.17)	6 (2 to 11)	0.07 (0.03 to 0.14)
	Male	44 (24 to 70)	1.35 (0.73 to 2.13)	36 (17 to 59)	1.08 (0.5 to 1.75)	4 (1 to 9)	0.17 (0.05 to 0.35)	5 (2 to 10)	0.14 (0.05 to 0.28)
	Female	2 (1 to 4)	0.06 (0.03 to 0.11)	2 (1 to 3)	0.04 (0.01 to 0.07)	0 (0 to 1)	0.01 (0 to 0.02)	1 (0 to 1)	0.02 (0.01 to 0.03)
Honduras	Both	38 (28 to 51)	0.67 (0.48 to 0.88)	9 (4 to 14)	0.14 (0.07 to 0.23)	2 (1 to 4)	0.04 (0.02 to 0.07)	3 (1 to 6)	0.05 (0.02 to 0.09)
	Male	35 (25 to 45)	1.27 (0.92 to 1.64)	8 (4 to 13)	0.29 (0.15 to 0.46)	2 (1 to 3)	0.07 (0.03 to 0.12)	2 (1 to 5)	0.08 (0.03 to 0.15)
	Female	4 (2 to 6)	0.13 (0.08 to 0.2)	0 (0 to 1)	0.02 (0.01 to 0.03)	0 (0 to 1)	0.02 (0 to 0.04)	1 (0 to 1)	0.02 (0.01 to 0.04)
Hungary	Both	367 (290 to 458)	2.13 (1.67 to 2.66)	175 (106 to 239)	1.02 (0.62 to 1.4)	9 (4 to 16)	0.05 (0.02 to 0.08)	13 (3 to 28)	0.08 (0.02 to 0.17)
	Male	331 (260 to 414)	4.33 (3.4 to 5.43)	168 (102 to 230)	2.21 (1.34 to 3.01)	8 (4 to 15)	0.11 (0.05 to 0.19)	11 (3 to 25)	0.15 (0.03 to 0.33)
	Female	37 (25 to 48)	0.39 (0.26 to 0.51)	7 (3 to 13)	0.08 (0.03 to 0.14)	1 (0 to 1)	0.01 (0 to 0.01)	2 (0 to 3)	0.02 (0 to 0.04)
Iceland	Both	2 (1 to 2)	0.32 (0.26 to 0.39)	1 (0 to 1)	0.13 (0.08 to 0.18)	0 (0 to 0)	0.05 (0.03 to 0.07)	0 (0 to 0)	0.01 (0 to 0.03)
	Male	1 (1 to 2)	0.57 (0.45 to 0.69)	1 (0 to 1)	0.24 (0.14 to 0.34)	0 (0 to 0)	0.1 (0.05 to 0.15)	0 (0 to 0)	0.02 (0 to 0.05)
	Female	0 (0 to 0)	0.09 (0.07 to 0.12)	0 (0 to 0)	0.03 (0.01 to 0.04)	0 (0 to 0)	0 (0 to 0.01)	0 (0 to 0)	0 (0 to 0.01)
India	Both	15700 (12200 to 19800)	1.38 (1.08 to 1.73)	4120 (2150 to 6280)	0.34 (0.18 to 0.53)	534 (276 to 887)	0.05 (0.03 to 0.09)	1120 (465 to 2110)	0.09 (0.04 to 0.17)
	Male	14900 (11500 to 19000)	2.71 (2.1 to 3.42)	4050 (2130 to 6180)	0.69 (0.36 to 1.07)	497 (240 to 840)	0.11 (0.05 to 0.18)	1040 (423 to 1970)	0.17 (0.07 to 0.32)
	Female	795 (523 to 1130)	0.14 (0.09 to 0.2)	73 (26 to 136)	0.01 (0 to 0.02)	37 (14 to 73)	0.01 (0 to 0.01)	86 (34 to 168)	0.01 (0.01 to 0.03)

Indonesia	Both	1380 (986 to 1960)	0.68 (0.5 to 0.96)	33 (FALSE to 104)	0.01 (-0.01 to 0.05)	22 (11 to 40)	0.01 (0.01 to 0.03)	74 (28 to 151)	0.03 (0.01 to 0.06)
	Male	1310 (933 to 1880)	1.41 (1.02 to 2)	32 (FALSE to 103)	0.03 (-0.01 to 0.09)	21 (9 to 38)	0.03 (0.01 to 0.06)	62 (23 to 128)	0.05 (0.02 to 0.11)
	Female	61 (37 to 91)	0.06 (0.04 to 0.09)	0 (0 to 1)	0 (0 to 0)	2 (1 to 3)	0 (0 to 0)	13 (5 to 26)	0.01 (0 to 0.02)
Iran	Both	849 (691 to 988)	1.21 (0.97 to 1.41)	39 (20 to 58)	0.05 (0.03 to 0.08)	5 (3 to 10)	0.01 (0 to 0.02)	33 (13 to 62)	0.04 (0.02 to 0.08)
	Male	800 (658 to 930)	2.28 (1.85 to 2.66)	36 (19 to 55)	0.09 (0.05 to 0.14)	5 (2 to 9)	0.02 (0.01 to 0.03)	30 (12 to 57)	0.08 (0.03 to 0.14)
	Female	49 (32 to 67)	0.13 (0.09 to 0.19)	2 (1 to 3)	0.01 (0 to 0.01)	0 (0 to 1)	0 (0 to 0)	2 (1 to 5)	0.01 (0 to 0.01)
Iraq	Both	369 (279 to 459)	1.71 (1.31 to 2.1)	13 (4 to 27)	0.05 (0.01 to 0.1)	10 (5 to 19)	0.06 (0.03 to 0.11)	13 (5 to 25)	0.05 (0.02 to 0.1)
	Male	339 (255 to 422)	3.3 (2.53 to 4.06)	12 (4 to 25)	0.1 (0.03 to 0.2)	10 (4 to 18)	0.13 (0.05 to 0.22)	12 (5 to 24)	0.1 (0.04 to 0.19)
	Female	30 (18 to 44)	0.25 (0.15 to 0.36)	1 (0 to 2)	0 (0 to 0.01)	1 (0 to 1)	0.01 (0 to 0.01)	1 (0 to 1)	0 (0 to 0.01)
Ireland	Both	43 (36 to 51)	0.58 (0.48 to 0.67)	19 (11 to 26)	0.26 (0.15 to 0.35)	5 (3 to 9)	0.07 (0.04 to 0.11)	1 (0 to 3)	0.02 (0 to 0.04)
	Male	37 (30 to 44)	1.06 (0.87 to 1.25)	17 (10 to 23)	0.49 (0.3 to 0.66)	5 (3 to 9)	0.15 (0.08 to 0.24)	1 (0 to 2)	0.03 (0.01 to 0.06)
	Female	6 (5 to 8)	0.15 (0.11 to 0.19)	2 (1 to 3)	0.05 (0.02 to 0.07)	0 (0 to 0)	0 (0 to 0.01)	0 (0 to 0)	0 (0 to 0.01)
Israel	Both	66 (55 to 77)	0.57 (0.48 to 0.66)	11 (4 to 18)	0.1 (0.04 to 0.16)	5 (2 to 8)	0.04 (0.02 to 0.07)	2 (0 to 4)	0.02 (0 to 0.04)
	Male	60 (49 to 70)	1.13 (0.94 to 1.32)	10 (4 to 17)	0.2 (0.08 to 0.33)	5 (2 to 8)	0.09 (0.04 to 0.15)	2 (0 to 4)	0.03 (0.01 to 0.07)
	Female	6 (5 to 8)	0.1 (0.07 to 0.13)	1 (0 to 1)	0.01 (0 to 0.02)	0 (0 to 0)	0 (0 to 0.01)	0 (0 to 0)	0 (0 to 0.01)
Italy	Both	1190 (990 to 1360)	0.86 (0.73 to 0.96)	532 (327 to 713)	0.39 (0.24 to 0.51)	267 (149 to 396)	0.16 (0.09 to 0.25)	27 (6 to 58)	0.02 (0.01 to 0.05)
	Male	1100 (921 to 1260)	1.77 (1.5 to 1.99)	504 (312 to 671)	0.81 (0.51 to 1.08)	256 (141 to 383)	0.37 (0.2 to 0.56)	25 (6 to 54)	0.04 (0.01 to 0.1)
	Female	85 (62 to 109)	0.11 (0.09 to 0.14)	28 (14 to 43)	0.04 (0.02 to 0.06)	11 (4 to 19)	0.01 (0 to 0.02)	2 (0 to 4)	0 (0 to 0.01)
Jamaica	Both	23 (16 to 30)	0.78 (0.56 to 1.03)	6 (3 to 10)	0.22 (0.1 to 0.35)	1 (0 to 1)	0.03 (0.01 to 0.05)	1 (0 to 2)	0.04 (0.02 to 0.08)
	Male	22 (16 to 29)	1.57 (1.12 to 2.07)	6 (3 to 10)	0.44 (0.21 to 0.69)	1 (0 to 1)	0.06 (0.02 to 0.1)	1 (0 to 2)	0.08 (0.03 to 0.16)
	Female	1 (0 to 1)	0.05 (0.03 to 0.07)	0 (0 to 0)	0.01 (0 to 0.02)	0 (0 to 0)	0 (0 to 0)	0 (0 to 0)	0 (0 to 0.01)

Japan	Both	849 (696 to 971)	0.22 (0.19 to 0.25)	255 (131 to 390)	0.07 (0.04 to 0.11)	122 (62 to 186)	0.03 (0.01 to 0.04)	19 (5 to 43)	0.01 (0 to 0.01)
	Male	800 (658 to 909)	0.47 (0.4 to 0.53)	237 (121 to 362)	0.15 (0.08 to 0.22)	118 (59 to 183)	0.06 (0.03 to 0.1)	17 (4 to 39)	0.01 (0 to 0.03)
	Female	49 (31 to 69)	0.02 (0.02 to 0.03)	17 (8 to 30)	0.01 (0 to 0.02)	3 (1 to 6)	0 (0 to 0)	2 (0 to 4)	0 (0 to 0)
Jordan	Both	36 (27 to 48)	0.59 (0.45 to 0.8)	1 (0 to 3)	0.02 (0.01 to 0.04)	1 (0 to 2)	0.03 (0.01 to 0.05)	1 (0 to 2)	0.01 (0 to 0.02)
	Male	34 (25 to 46)	1.07 (0.79 to 1.46)	1 (0 to 3)	0.03 (0.01 to 0.07)	1 (0 to 2)	0.05 (0.02 to 0.09)	1 (0 to 2)	0.02 (0.01 to 0.04)
	Female	2 (1 to 3)	0.08 (0.05 to 0.11)	0 (0 to 0)	0 (0 to 0)	0 (0 to 0)	0 (0 to 0)	0 (0 to 0)	0 (0 to 0)
Kazakhstan	Both	192 (155 to 228)	1.05 (0.85 to 1.24)	61 (33 to 91)	0.32 (0.17 to 0.48)	5 (3 to 9)	0.03 (0.02 to 0.05)	10 (4 to 19)	0.05 (0.02 to 0.1)
	Male	188 (152 to 224)	2.55 (2.08 to 3.04)	59 (32 to 86)	0.74 (0.39 to 1.1)	5 (2 to 8)	0.08 (0.04 to 0.13)	9 (4 to 18)	0.11 (0.04 to 0.2)
	Female	3 (2 to 5)	0.03 (0.02 to 0.05)	2 (1 to 4)	0.02 (0.01 to 0.04)	0 (0 to 1)	0 (0 to 0.01)	1 (0 to 2)	0.01 (0 to 0.02)
Kenya	Both	131 (86 to 190)	0.57 (0.39 to 0.83)	66 (33 to 103)	0.27 (0.14 to 0.43)	5 (2 to 17)	0.03 (0.01 to 0.09)	15 (5 to 29)	0.05 (0.02 to 0.11)
	Male	127 (84 to 185)	1.19 (0.81 to 1.74)	63 (32 to 99)	0.55 (0.27 to 0.87)	4 (1 to 16)	0.05 (0.01 to 0.2)	13 (5 to 26)	0.1 (0.03 to 0.19)
	Female	4 (2 to 6)	0.04 (0.02 to 0.06)	3 (1 to 5)	0.02 (0.01 to 0.04)	1 (0 to 1)	0.01 (0 to 0.01)	2 (1 to 4)	0.01 (0.01 to 0.03)
Kiribati	Both	0 (0 to 0)	0.55 (0.43 to 0.7)	0 (0 to 0)	0.03 (0 to 0.07)	0 (0 to 0)	0.01 (0 to 0.01)	0 (0 to 0)	0.01 (0 to 0.02)
	Male	0 (0 to 0)	1.1 (0.83 to 1.41)	0 (0 to 0)	0.07 (0 to 0.17)	0 (0 to 0)	0.01 (0.01 to 0.02)	0 (0 to 0)	0.02 (0.01 to 0.03)
	Female	0 (0 to 0)	0.22 (0.15 to 0.29)	0 (0 to 0)	0 (0 to 0)	0 (0 to 0)	0 (0 to 0.01)	0 (0 to 0)	0.01 (0 to 0.01)
Kuwait	Both	11 (8 to 14)	0.52 (0.39 to 0.67)	0 (0 to 0)	0 (0 to 0.01)	1 (0 to 1)	0.04 (0.02 to 0.07)	0 (0 to 1)	0.01 (0 to 0.02)
	Male	10 (8 to 13)	0.84 (0.63 to 1.09)	0 (0 to 0)	0 (0 to 0.01)	1 (0 to 1)	0.07 (0.03 to 0.11)	0 (0 to 1)	0.02 (0.01 to 0.03)
	Female	0 (0 to 1)	0.04 (0.02 to 0.06)	0 (0 to 0)	0 (0 to 0)	0 (0 to 0)	0 (0 to 0)	0 (0 to 0)	0 (0 to 0)
Kyrgyzstan	Both	30 (24 to 36)	0.63 (0.51 to 0.75)	8 (5 to 12)	0.16 (0.09 to 0.25)	0 (0 to 1)	0.01 (0 to 0.01)	1 (1 to 3)	0.03 (0.01 to 0.05)
	Male	29 (23 to 35)	1.43 (1.17 to 1.72)	8 (5 to 12)	0.36 (0.2 to 0.54)	0 (0 to 1)	0.02 (0.01 to 0.03)	1 (1 to 3)	0.05 (0.02 to 0.1)
	Female	1 (1 to 1)	0.03 (0.02 to 0.05)	0 (0 to 0)	0.01 (0 to 0.02)	0 (0 to 0)	0 (0 to 0)	0 (0 to 0)	0 (0 to 0.01)

Laos	Both	35 (25 to 49)	0.87 (0.62 to 1.19)	10 (4 to 17)	0.22 (0.09 to 0.38)	0 (0 to 1)	0.01 (0 to 0.02)	2 (1 to 4)	0.04 (0.02 to 0.09)
	Male	33 (23 to 46)	1.71 (1.23 to 2.35)	9 (4 to 16)	0.42 (0.18 to 0.73)	0 (0 to 1)	0.02 (0.01 to 0.05)	2 (1 to 3)	0.06 (0.02 to 0.13)
	Female	2 (1 to 4)	0.12 (0.07 to 0.18)	1 (0 to 2)	0.03 (0.01 to 0.07)	0 (0 to 0)	0 (0 to 0)	1 (0 to 1)	0.02 (0.01 to 0.04)
Latvia	Both	52 (39 to 67)	1.46 (1.1 to 1.92)	25 (15 to 37)	0.73 (0.43 to 1.05)	2 (1 to 3)	0.04 (0.02 to 0.07)	1 (0 to 2)	0.03 (0.01 to 0.07)
	Male	51 (38 to 66)	3.55 (2.67 to 4.64)	25 (15 to 36)	1.75 (1.04 to 2.51)	1 (1 to 3)	0.1 (0.04 to 0.18)	1 (0 to 2)	0.06 (0.01 to 0.14)
	Female	1 (1 to 2)	0.05 (0.03 to 0.08)	0 (0 to 1)	0.02 (0.01 to 0.04)	0 (0 to 0)	0 (0 to 0)	0 (0 to 0)	0 (0 to 0.01)
Lebanon	Both	124 (98 to 160)	2.38 (1.9 to 3.09)	9 (4 to 14)	0.17 (0.09 to 0.28)	0 (0 to 2)	0.01 (0 to 0.03)	3 (1 to 7)	0.07 (0.03 to 0.13)
	Male	111 (87 to 144)	4.74 (3.72 to 6.13)	9 (4 to 14)	0.38 (0.19 to 0.6)	0 (0 to 2)	0.01 (0 to 0.07)	3 (1 to 6)	0.14 (0.06 to 0.27)
	Female	13 (9 to 18)	0.46 (0.32 to 0.65)	0 (0 to 0)	0.01 (0 to 0.01)	0 (0 to 0)	0 (0 to 0.01)	0 (0 to 0)	0.01 (0 to 0.01)
Lesotho	Both	24 (18 to 32)	1.83 (1.35 to 2.43)	7 (3 to 12)	0.53 (0.24 to 0.87)	5 (2 to 8)	0.37 (0.14 to 0.66)	1 (0 to 1)	0.04 (0.02 to 0.09)
	Male	22 (16 to 29)	4.18 (3.17 to 5.46)	7 (3 to 11)	1.2 (0.55 to 1.95)	5 (2 to 8)	0.95 (0.35 to 1.67)	1 (0 to 1)	0.09 (0.03 to 0.17)
	Female	2 (1 to 3)	0.25 (0.15 to 0.38)	0 (0 to 1)	0.05 (0.02 to 0.1)	0 (0 to 0)	0.02 (0.01 to 0.04)	0 (0 to 0)	0.01 (0 to 0.03)
Liberia	Both	8 (5 to 11)	0.38 (0.23 to 0.57)	4 (2 to 7)	0.2 (0.09 to 0.34)	0 (0 to 0)	0.01 (0 to 0.02)	1 (0 to 1)	0.03 (0.01 to 0.06)
	Male	7 (5 to 11)	0.73 (0.44 to 1.08)	4 (2 to 7)	0.38 (0.17 to 0.63)	0 (0 to 0)	0.01 (0 to 0.04)	1 (0 to 1)	0.05 (0.02 to 0.09)
	Female	0 (0 to 0)	0.02 (0.01 to 0.03)	0 (0 to 0)	0.02 (0.01 to 0.04)	0 (0 to 0)	0 (0 to 0)	0 (0 to 0)	0.01 (0 to 0.01)
Libya	Both	102 (76 to 136)	2.01 (1.51 to 2.65)	3 (1 to 5)	0.05 (0.01 to 0.08)	1 (0 to 3)	0.02 (0 to 0.08)	3 (1 to 6)	0.05 (0.02 to 0.11)
	Male	101 (75 to 134)	3.92 (2.93 to 5.16)	3 (1 to 5)	0.09 (0.02 to 0.15)	1 (0 to 3)	0.03 (0.01 to 0.15)	3 (1 to 6)	0.1 (0.04 to 0.2)
	Female	1 (0 to 1)	0.04 (0.02 to 0.06)	0 (0 to 0)	0 (0 to 0)	0 (0 to 0)	0 (0 to 0)	0 (0 to 0)	0.01 (0 to 0.01)
Lithuania	Both	85 (65 to 106)	1.64 (1.25 to 2.05)	44 (26 to 60)	0.86 (0.51 to 1.18)	2 (1 to 3)	0.03 (0.01 to 0.05)	2 (0 to 4)	0.04 (0.01 to 0.08)
	Male	83 (64 to 104)	3.97 (3.03 to 4.96)	43 (25 to 58)	2.05 (1.22 to 2.79)	2 (1 to 3)	0.08 (0.03 to 0.14)	2 (0 to 4)	0.08 (0.02 to 0.17)
	Female	2 (1 to 3)	0.07 (0.04 to 0.09)	1 (1 to 2)	0.04 (0.02 to 0.07)	0 (0 to 0)	0 (0 to 0)	0 (0 to 0)	0 (0 to 0.01)

Luxembourg	Both	6 (5 to 8)	0.67 (0.54 to 0.8)	3 (2 to 5)	0.35 (0.22 to 0.46)	1 (1 to 2)	0.1 (0.05 to 0.15)	0 (0 to 0)	0.01 (0 to 0.03)
	Male	6 (5 to 7)	1.22 (0.99 to 1.47)	3 (2 to 4)	0.67 (0.42 to 0.89)	1 (1 to 2)	0.22 (0.11 to 0.34)	0 (0 to 0)	0.03 (0.01 to 0.06)
	Female	1 (1 to 1)	0.16 (0.12 to 0.21)	0 (0 to 1)	0.06 (0.03 to 0.1)	0 (0 to 0)	0 (0 to 0.01)	0 (0 to 0)	0 (0 to 0.01)
Madagascar	Both	33 (19 to 50)	0.29 (0.17 to 0.44)	11 (3 to 22)	0.09 (0.02 to 0.18)	1 (0 to 4)	0.01 (0 to 0.05)	5 (2 to 10)	0.04 (0.01 to 0.07)
	Male	31 (18 to 48)	0.57 (0.34 to 0.86)	10 (3 to 20)	0.16 (0.04 to 0.33)	0 (0 to 4)	0.01 (0 to 0.1)	4 (1 to 7)	0.05 (0.02 to 0.1)
	Female	2 (1 to 3)	0.03 (0.02 to 0.06)	1 (0 to 2)	0.02 (0 to 0.04)	0 (0 to 1)	0.01 (0 to 0.01)	2 (1 to 3)	0.02 (0.01 to 0.04)
Malawi	Both	17 (12 to 23)	0.24 (0.17 to 0.32)	5 (2 to 8)	0.06 (0.03 to 0.11)	0 (0 to 2)	0.01 (0 to 0.03)	2 (1 to 3)	0.02 (0.01 to 0.04)
	Male	16 (11 to 22)	0.54 (0.39 to 0.72)	4 (2 to 7)	0.13 (0.06 to 0.21)	0 (0 to 2)	0.01 (0 to 0.08)	1 (0 to 2)	0.03 (0.01 to 0.06)
	Female	1 (0 to 2)	0.02 (0.01 to 0.04)	1 (0 to 1)	0.01 (0 to 0.03)	0 (0 to 0)	0 (0 to 0.01)	0 (0 to 1)	0.01 (0 to 0.02)
Malaysia	Both	214 (157 to 279)	0.85 (0.63 to 1.1)	24 (9 to 41)	0.08 (0.03 to 0.15)	3 (1 to 6)	0.02 (0.01 to 0.03)	11 (4 to 21)	0.04 (0.01 to 0.07)
	Male	208 (151 to 272)	1.66 (1.23 to 2.14)	23 (8 to 40)	0.16 (0.06 to 0.29)	3 (1 to 6)	0.03 (0.01 to 0.06)	10 (4 to 19)	0.07 (0.03 to 0.13)
	Female	6 (4 to 10)	0.05 (0.03 to 0.09)	1 (0 to 1)	0 (0 to 0.01)	0 (0 to 0)	0 (0 to 0)	1 (1 to 3)	0.01 (0 to 0.02)
Maldives	Both	2 (1 to 2)	0.67 (0.54 to 0.83)	0 (0 to 0)	0.04 (-0.01 to 0.1)	0 (0 to 0)	0.04 (0.02 to 0.07)	0 (0 to 0)	0.02 (0.01 to 0.03)
	Male	2 (1 to 2)	1.24 (0.99 to 1.53)	0 (0 to 0)	0.07 (-0.01 to 0.18)	0 (0 to 0)	0.08 (0.04 to 0.14)	0 (0 to 0)	0.03 (0.01 to 0.06)
	Female	0 (0 to 0)	0.02 (0.01 to 0.04)	0 (0 to 0)	0 (0 to 0)	0 (0 to 0)	0 (0 to 0)	0 (0 to 0)	0 (0 to 0)
Mali	Both	31 (21 to 46)	0.37 (0.25 to 0.53)	5 (2 to 8)	0.05 (0.02 to 0.09)	0 (0 to 1)	0 (0 to 0.01)	3 (1 to 5)	0.03 (0.01 to 0.05)
	Male	30 (20 to 44)	0.69 (0.46 to 0.98)	4 (2 to 6)	0.08 (0.04 to 0.14)	0 (0 to 1)	0.01 (0 to 0.02)	2 (1 to 4)	0.04 (0.01 to 0.08)
	Female	1 (1 to 2)	0.03 (0.01 to 0.05)	1 (0 to 2)	0.02 (0.01 to 0.04)	0 (0 to 0)	0 (0 to 0.01)	1 (0 to 1)	0.01 (0 to 0.02)
Malta	Both	6 (5 to 7)	0.7 (0.57 to 0.84)	2 (1 to 3)	0.29 (0.17 to 0.4)	1 (1 to 2)	0.11 (0.06 to 0.17)	0 (0 to 0)	0.02 (0.01 to 0.05)
	Male	6 (4 to 7)	1.35 (1.09 to 1.63)	2 (1 to 3)	0.58 (0.34 to 0.8)	1 (1 to 2)	0.25 (0.13 to 0.38)	0 (0 to 0)	0.04 (0.01 to 0.1)
	Female	1 (0 to 1)	0.12 (0.09 to 0.16)	0 (0 to 0)	0.03 (0.01 to 0.05)	0 (0 to 0)	0 (0 to 0.01)	0 (0 to 0)	0 (0 to 0.01)

Marshall Islands	Both	0 (0 to 0)	0.64 (0.43 to 0.91)	0 (0 to 0)	0.1 (0.01 to 0.21)	0 (0 to 0)	0.04 (0.02 to 0.08)	0 (0 to 0)	0.02 (0.01 to 0.04)
	Male	0 (0 to 0)	1.11 (0.73 to 1.57)	0 (0 to 0)	0.18 (0.03 to 0.38)	0 (0 to 0)	0.07 (0.03 to 0.14)	0 (0 to 0)	0.03 (0.01 to 0.06)
	Female	0 (0 to 0)	0.12 (0.07 to 0.2)	0 (0 to 0)	0 (0 to 0.01)	0 (0 to 0)	0 (0 to 0.01)	0 (0 to 0)	0.01 (0 to 0.02)
Mauritania	Both	7 (5 to 10)	0.36 (0.23 to 0.51)	0 (0 to 0)	0 (0 to 0)	0 (0 to 0)	0.01 (0 to 0.01)	0 (0 to 1)	0.01 (0 to 0.03)
	Male	7 (4 to 10)	0.69 (0.44 to 0.98)	0 (0 to 0)	0 (0 to 0)	0 (0 to 0)	0.01 (0 to 0.03)	0 (0 to 1)	0.03 (0.01 to 0.05)
	Female	0 (0 to 0)	0.01 (0.01 to 0.03)	0 (0 to 0)	0 (0 to 0)	0 (0 to 0)	0 (0 to 0)	0 (0 to 0)	0 (0 to 0)
Mauritius	Both	13 (10 to 16)	0.73 (0.56 to 0.92)	4 (2 to 6)	0.21 (0.1 to 0.34)	0 (0 to 0)	0.01 (0 to 0.01)	1 (0 to 1)	0.03 (0.01 to 0.06)
	Male	13 (10 to 16)	1.62 (1.24 to 2.03)	4 (2 to 6)	0.47 (0.21 to 0.73)	0 (0 to 0)	0.02 (0.01 to 0.03)	1 (0 to 1)	0.06 (0.02 to 0.11)
	Female	0 (0 to 0)	0.03 (0.01 to 0.05)	0 (0 to 0)	0.01 (0 to 0.01)	0 (0 to 0)	0 (0 to 0)	0 (0 to 0)	0 (0 to 0.01)
Mexico	Both	517 (381 to 680)	0.46 (0.34 to 0.6)	220 (122 to 321)	0.19 (0.1 to 0.28)	34 (18 to 56)	0.03 (0.02 to 0.05)	42 (17 to 78)	0.04 (0.01 to 0.07)
	Male	490 (362 to 647)	0.95 (0.71 to 1.24)	210 (118 to 307)	0.39 (0.22 to 0.57)	32 (16 to 54)	0.06 (0.03 to 0.11)	38 (15 to 71)	0.07 (0.03 to 0.13)
	Female	27 (16 to 42)	0.05 (0.03 to 0.07)	10 (4 to 19)	0.02 (0.01 to 0.03)	2 (1 to 4)	0 (0 to 0.01)	4 (2 to 8)	0.01 (0 to 0.01)
Federatead States of Micronesia	Both	0 (0 to 1)	0.66 (0.46 to 0.9)	0 (0 to 0)	0.08 (0.03 to 0.14)	0 (0 to 0)	0.04 (0.02 to 0.08)	0 (0 to 0)	0.02 (0.01 to 0.05)
	Male	0 (0 to 1)	1.26 (0.87 to 1.71)	0 (0 to 0)	0.17 (0.06 to 0.3)	0 (0 to 0)	0.09 (0.03 to 0.18)	0 (0 to 0)	0.04 (0.01 to 0.07)
	Female	0 (0 to 0)	0.2 (0.12 to 0.3)	0 (0 to 0)	0 (0 to 0)	0 (0 to 0)	0 (0 to 0.01)	0 (0 to 0)	0.01 (0 to 0.03)
Monaco	Both	2 (2 to 3)	2.36 (1.79 to 2.97)	1 (0 to 1)	0.73 (0 to 1.44)	0 (0 to 1)	0.37 (0.19 to 0.6)	0 (0 to 0)	0.07 (0.02 to 0.18)
	Male	2 (1 to 2)	4.61 (3.53 to 5.82)	1 (0 to 1)	1.46 (0 to 2.88)	0 (0 to 1)	0.82 (0.41 to 1.31)	0 (0 to 0)	0.14 (0.03 to 0.33)
	Female	0 (0 to 0)	0.34 (0.22 to 0.47)	0 (0 to 0)	0.07 (0 to 0.19)	0 (0 to 0)	0.01 (0 to 0.01)	0 (0 to 0)	0.01 (0 to 0.02)
Mongolia	Both	21 (16 to 28)	1.17 (0.78 to 1.53)	7 (4 to 10)	0.34 (0.17 to 0.52)	0 (0 to 1)	0.02 (0.01 to 0.05)	1 (0 to 1)	0.03 (0.01 to 0.06)
	Male	20 (15 to 27)	3 (1.94 to 3.88)	7 (4 to 10)	0.85 (0.42 to 1.33)	0 (0 to 1)	0.06 (0.02 to 0.11)	1 (0 to 1)	0.06 (0.02 to 0.12)
	Female	1 (1 to 1)	0.08 (0.05 to 0.14)	0 (0 to 0)	0.01 (0 to 0.03)	0 (0 to 0)	0 (0 to 0)	0 (0 to 0)	0.01 (0 to 0.01)

Montenegro	Both	37 (30 to 46)	3.75 (3 to 4.65)	14 (8 to 20)	1.45 (0.84 to 2.04)	0 (0 to 1)	0.03 (0.02 to 0.05)	1 (0 to 2)	0.08 (0.02 to 0.19)
	Male	31 (25 to 40)	6.87 (5.46 to 8.63)	13 (8 to 19)	2.9 (1.69 to 4.07)	0 (0 to 0)	0.05 (0.02 to 0.09)	1 (0 to 2)	0.14 (0.03 to 0.33)
	Female	6 (4 to 7)	1.12 (0.84 to 1.4)	1 (0 to 2)	0.22 (0.07 to 0.38)	0 (0 to 0)	0.02 (0.01 to 0.03)	0 (0 to 0)	0.03 (0.01 to 0.06)
Morocco	Both	590 (415 to 783)	1.87 (1.34 to 2.45)	29 (8 to 57)	0.08 (0.02 to 0.16)	2 (0 to 12)	0.01 (0 to 0.05)	29 (11 to 58)	0.08 (0.03 to 0.16)
	Male	586 (413 to 777)	3.76 (2.69 to 4.93)	29 (8 to 57)	0.16 (0.05 to 0.32)	2 (0 to 12)	0.02 (0 to 0.1)	28 (10 to 56)	0.16 (0.06 to 0.31)
	Female	4 (2 to 7)	0.03 (0.02 to 0.04)	0 (0 to 0)	0 (0 to 0)	0 (0 to 0)	0 (0 to 0)	1 (0 to 3)	0.01 (0 to 0.01)
Mozambique	Both	77 (49 to 109)	0.68 (0.44 to 0.96)	18 (2 to 38)	0.15 (0.02 to 0.31)	3 (1 to 11)	0.03 (0.01 to 0.12)	8 (3 to 15)	0.06 (0.02 to 0.11)
	Male	73 (47 to 103)	1.49 (0.99 to 2.07)	17 (2 to 35)	0.32 (0.03 to 0.65)	2 (1 to 10)	0.07 (0.02 to 0.27)	6 (2 to 12)	0.11 (0.04 to 0.2)
	Female	4 (2 to 7)	0.06 (0.03 to 0.11)	1 (0 to 3)	0.02 (0 to 0.04)	0 (0 to 1)	0.01 (0 to 0.02)	2 (1 to 3)	0.02 (0.01 to 0.04)
Myanmar	Both	297 (228 to 398)	0.67 (0.52 to 0.9)	65 (33 to 105)	0.14 (0.07 to 0.22)	6 (3 to 11)	0.02 (0.01 to 0.03)	15 (6 to 30)	0.03 (0.01 to 0.06)
	Male	256 (197 to 347)	1.37 (1.05 to 1.83)	63 (32 to 101)	0.3 (0.15 to 0.49)	6 (2 to 10)	0.03 (0.02 to 0.07)	12 (5 to 24)	0.05 (0.02 to 0.1)
	Female	41 (26 to 59)	0.17 (0.11 to 0.24)	2 (0 to 4)	0.01 (0 to 0.02)	0 (0 to 1)	0 (0 to 0)	3 (1 to 6)	0.01 (0 to 0.02)
Namibia	Both	18 (12 to 25)	1.28 (0.91 to 1.76)	10 (5 to 15)	0.66 (0.34 to 1.01)	4 (2 to 7)	0.32 (0.12 to 0.56)	0 (0 to 1)	0.03 (0.01 to 0.06)
	Male	15 (10 to 21)	2.65 (1.88 to 3.65)	9 (5 to 14)	1.42 (0.75 to 2.15)	4 (1 to 7)	0.76 (0.28 to 1.36)	0 (0 to 1)	0.06 (0.02 to 0.11)
	Female	3 (2 to 4)	0.33 (0.2 to 0.49)	1 (0 to 2)	0.1 (0.03 to 0.19)	0 (0 to 0)	0.02 (0.01 to 0.04)	0 (0 to 0)	0.01 (0 to 0.02)
Nauru	Both	0 (0 to 0)	0.75 (0.53 to 0.99)	0 (0 to 0)	0.2 (0.08 to 0.33)	0 (0 to 0)	0.03 (0.01 to 0.06)	0 (0 to 0)	0.03 (0.01 to 0.05)
	Male	0 (0 to 0)	1.37 (0.96 to 1.83)	0 (0 to 0)	0.42 (0.18 to 0.7)	0 (0 to 0)	0.06 (0.02 to 0.12)	0 (0 to 0)	0.05 (0.02 to 0.09)
	Female	0 (0 to 0)	0.21 (0.12 to 0.31)	0 (0 to 0)	0.01 (0 to 0.02)	0 (0 to 0)	0 (0 to 0.01)	0 (0 to 0)	0.01 (0 to 0.03)
Nepal	Both	310 (214 to 423)	1.43 (1 to 1.95)	66 (19 to 126)	0.29 (0.08 to 0.55)	8 (4 to 16)	0.04 (0.02 to 0.08)	24 (10 to 48)	0.1 (0.04 to 0.2)
	Male	266 (183 to 368)	2.59 (1.82 to 3.56)	63 (18 to 119)	0.57 (0.16 to 1.09)	7 (3 to 14)	0.08 (0.03 to 0.16)	18 (7 to 37)	0.16 (0.06 to 0.32)
	Female	43 (27 to 61)	0.39 (0.25 to 0.54)	3 (1 to 7)	0.03 (0 to 0.06)	1 (0 to 2)	0.01 (0 to 0.02)	6 (2 to 11)	0.05 (0.02 to 0.09)

Netherlands	Both	193 (162 to 222)	0.56 (0.48 to 0.65)	86 (51 to 115)	0.25 (0.15 to 0.34)	48 (28 to 67)	0.13 (0.08 to 0.18)	5 (1 to 11)	0.02 (0 to 0.04)
	Male	160 (134 to 185)	1.02 (0.85 to 1.17)	74 (45 to 99)	0.47 (0.29 to 0.63)	45 (26 to 65)	0.28 (0.16 to 0.4)	4 (1 to 10)	0.03 (0.01 to 0.06)
	Female	33 (21 to 41)	0.18 (0.12 to 0.22)	12 (6 to 18)	0.07 (0.03 to 0.1)	2 (1 to 4)	0.01 (0 to 0.02)	1 (0 to 2)	0.01 (0 to 0.01)
New Zealand	Both	21 (16 to 25)	0.27 (0.21 to 0.32)	11 (7 to 15)	0.14 (0.09 to 0.19)	5 (3 to 8)	0.06 (0.04 to 0.09)	1 (0 to 2)	0.01 (0 to 0.02)
	Male	17 (13 to 21)	0.46 (0.37 to 0.56)	9 (6 to 13)	0.26 (0.16 to 0.35)	5 (3 to 8)	0.13 (0.07 to 0.2)	1 (0 to 1)	0.02 (0 to 0.04)
	Female	4 (3 to 5)	0.1 (0.07 to 0.12)	2 (1 to 3)	0.04 (0.02 to 0.07)	0 (0 to 0)	0.01 (0 to 0.01)	0 (0 to 0)	0 (0 to 0.01)
Nicaragua	Both	27 (20 to 35)	0.67 (0.49 to 0.88)	7 (4 to 11)	0.17 (0.09 to 0.27)	0 (0 to 1)	0.01 (0.01 to 0.02)	2 (1 to 4)	0.04 (0.02 to 0.08)
	Male	26 (19 to 35)	1.51 (1.11 to 1.98)	7 (4 to 11)	0.37 (0.2 to 0.59)	0 (0 to 1)	0.03 (0.01 to 0.05)	2 (1 to 3)	0.08 (0.03 to 0.16)
	Female	1 (0 to 1)	0.03 (0.01 to 0.04)	0 (0 to 0)	0.01 (0 to 0.01)	0 (0 to 0)	0 (0 to 0)	0 (0 to 0)	0.01 (0 to 0.01)
Niger	Both	20 (12 to 29)	0.31 (0.2 to 0.44)	2 (0 to 5)	0.02 (0 to 0.06)	0 (0 to 1)	0.01 (0 to 0.01)	2 (1 to 5)	0.03 (0.01 to 0.05)
	Male	20 (12 to 29)	0.63 (0.4 to 0.9)	2 (0 to 4)	0.04 (-0.01 to 0.12)	0 (0 to 1)	0.01 (0 to 0.03)	2 (1 to 4)	0.05 (0.02 to 0.09)
	Female	0 (0 to 1)	0.01 (0 to 0.01)	0 (0 to 0)	0 (0 to 0.01)	0 (0 to 0)	0 (0 to 0)	0 (0 to 1)	0.01 (0 to 0.01)
Nigeria	Both	234 (137 to 363)	0.26 (0.16 to 0.4)	224 (121 to 358)	0.26 (0.14 to 0.4)	3 (1 to 12)	0.01 (0 to 0.02)	21 (8 to 43)	0.02 (0.01 to 0.04)
	Male	232 (135 to 361)	0.56 (0.33 to 0.86)	221 (120 to 354)	0.54 (0.29 to 0.85)	3 (1 to 12)	0.01 (0 to 0.03)	20 (7 to 42)	0.04 (0.02 to 0.09)
	Female	2 (1 to 4)	0.01 (0 to 0.01)	3 (1 to 6)	0.01 (0 to 0.01)	0 (0 to 0)	0 (0 to 0)	1 (0 to 2)	0 (0 to 0)
Niue	Both	0 (0 to 0)	0.43 (0.31 to 0.56)	0 (0 to 0)	0.11 (0.01 to 0.2)	0 (0 to 0)	0.01 (0.01 to 0.03)	0 (0 to 0)	0.01 (0.01 to 0.03)
	Male	0 (0 to 0)	0.9 (0.65 to 1.19)	0 (0 to 0)	0.23 (0.03 to 0.44)	0 (0 to 0)	0.03 (0.01 to 0.07)	0 (0 to 0)	0.02 (0.01 to 0.04)
	Female	0 (0 to 0)	0.09 (0.05 to 0.13)	0 (0 to 0)	0 (0 to 0.01)	0 (0 to 0)	0 (0 to 0)	0 (0 to 0)	0.01 (0 to 0.01)
North Macedonia	Both	97 (74 to 125)	2.97 (2.28 to 3.81)	37 (21 to 54)	1.14 (0.64 to 1.65)	0 (0 to 1)	0.01 (0 to 0.02)	2 (1 to 5)	0.07 (0.01 to 0.16)
	Male	91 (70 to 118)	5.84 (4.47 to 7.48)	37 (21 to 53)	2.34 (1.33 to 3.38)	0 (0 to 1)	0.02 (0.01 to 0.04)	2 (0 to 5)	0.13 (0.03 to 0.3)
	Female	6 (4 to 8)	0.36 (0.26 to 0.48)	0 (0 to 1)	0.03 (0 to 0.06)	0 (0 to 0)	0 (0 to 0.01)	0 (0 to 0)	0.01 (0 to 0.03)

Northern Mariana Islands	Both	0 (0 to 0)	0.35 (0.26 to 0.45)	0 (0 to 0)	0.04 (0 to 0.1)	0 (0 to 0)	0.01 (0 to 0.01)	0 (0 to 0)	0.02 (0.01 to 0.03)
	Male	0 (0 to 0)	0.62 (0.47 to 0.84)	0 (0 to 0)	0.07 (0 to 0.2)	0 (0 to 0)	0.02 (0.01 to 0.03)	0 (0 to 0)	0.02 (0.01 to 0.04)
	Female	0 (0 to 0)	0.09 (0.05 to 0.13)	0 (0 to 0)	0 (0 to 0.01)	0 (0 to 0)	0 (0 to 0)	0 (0 to 0)	0.01 (0 to 0.02)
Norway	Both	19 (15 to 23)	0.2 (0.16 to 0.24)	10 (5 to 14)	0.1 (0.06 to 0.15)	5 (3 to 8)	0.05 (0.03 to 0.07)	1 (0 to 1)	0.01 (0 to 0.02)
	Male	16 (13 to 20)	0.37 (0.28 to 0.44)	9 (5 to 12)	0.19 (0.11 to 0.27)	5 (3 to 7)	0.1 (0.06 to 0.15)	1 (0 to 1)	0.01 (0 to 0.03)
	Female	3 (2 to 4)	0.06 (0.04 to 0.08)	1 (0 to 2)	0.02 (0.01 to 0.04)	0 (0 to 1)	0.01 (0 to 0.02)	0 (0 to 0)	0 (0 to 0.01)
Oman	Both	6 (4 to 7)	0.4 (0.3 to 0.51)	0 (0 to 0)	0.01 (0 to 0.02)	0 (0 to 1)	0.05 (0.02 to 0.08)	0 (0 to 1)	0.01 (0 to 0.02)
	Male	5 (4 to 7)	0.75 (0.57 to 0.97)	0 (0 to 0)	0.02 (0 to 0.03)	0 (0 to 1)	0.1 (0.05 to 0.16)	0 (0 to 0)	0.02 (0.01 to 0.04)
	Female	0 (0 to 0)	0.02 (0.01 to 0.03)	0 (0 to 0)	0 (0 to 0)	0 (0 to 0)	0 (0 to 0)	0 (0 to 0)	0 (0 to 0)
Pakistan	Both	3850 (2780 to 5310)	3.47 (2.54 to 4.74)	285 (129 to 495)	0.23 (0.1 to 0.39)	52 (23 to 116)	0.06 (0.03 to 0.13)	276 (101 to 557)	0.21 (0.08 to 0.43)
	Male	3730 (2670 to 5180)	6.53 (4.77 to 8.97)	282 (128 to 490)	0.44 (0.2 to 0.74)	46 (18 to 110)	0.1 (0.04 to 0.24)	260 (94 to 530)	0.38 (0.14 to 0.77)
	Female	127 (72 to 198)	0.22 (0.13 to 0.34)	4 (1 to 7)	0.01 (0 to 0.01)	7 (2 to 16)	0.02 (0 to 0.03)	17 (6 to 34)	0.03 (0.01 to 0.05)
Palau	Both	0 (0 to 0)	0.23 (0.16 to 0.31)	0 (0 to 0)	0.03 (0 to 0.07)	0 (0 to 0)	0.01 (0 to 0.01)	0 (0 to 0)	0.01 (0.01 to 0.03)
	Male	0 (0 to 0)	0.32 (0.23 to 0.44)	0 (0 to 0)	0.06 (0 to 0.12)	0 (0 to 0)	0.01 (0 to 0.02)	0 (0 to 0)	0.01 (0 to 0.02)
	Female	0 (0 to 0)	0.15 (0.09 to 0.23)	0 (0 to 0)	0 (0 to 0.02)	0 (0 to 0)	0 (0 to 0.01)	0 (0 to 0)	0.02 (0.01 to 0.04)
Palestine	Both	16 (13 to 20)	0.76 (0.62 to 0.93)	1 (1 to 2)	0.05 (0.03 to 0.08)	0 (0 to 0)	0 (0 to 0.01)	0 (0 to 1)	0.02 (0.01 to 0.03)
	Male	16 (13 to 20)	1.65 (1.33 to 2.02)	1 (1 to 2)	0.11 (0.06 to 0.17)	0 (0 to 0)	0.01 (0 to 0.01)	0 (0 to 1)	0.03 (0.01 to 0.07)
	Female	0 (0 to 0)	0.03 (0.02 to 0.04)	0 (0 to 0)	0 (0 to 0)	0 (0 to 0)	0 (0 to 0)	0 (0 to 0)	0 (0 to 0)
Panama	Both	21 (14 to 29)	0.51 (0.35 to 0.71)	9 (5 to 13)	0.21 (0.12 to 0.32)	0 (0 to 1)	0.01 (0 to 0.02)	1 (1 to 3)	0.03 (0.01 to 0.06)
	Male	20 (14 to 28)	1.01 (0.69 to 1.39)	8 (5 to 13)	0.42 (0.23 to 0.64)	0 (0 to 1)	0.02 (0.01 to 0.04)	1 (0 to 2)	0.06 (0.02 to 0.12)
	Female	1 (1 to 2)	0.05 (0.03 to 0.08)	0 (0 to 1)	0.01 (0.01 to 0.02)	0 (0 to 0)	0 (0 to 0)	0 (0 to 0)	0 (0 to 0.01)

Papua New Guinea	Both	17 (11 to 24)	0.38 (0.25 to 0.55)	2 (0 to 5)	0.04 (0 to 0.1)	1 (0 to 1)	0.02 (0.01 to 0.03)	1 (0 to 1)	0.01 (0 to 0.03)
	Male	14 (9 to 21)	0.64 (0.42 to 0.92)	2 (0 to 5)	0.08 (0 to 0.19)	1 (0 to 1)	0.03 (0.01 to 0.06)	0 (0 to 1)	0.02 (0.01 to 0.03)
	Female	3 (1 to 4)	0.11 (0.06 to 0.17)	0 (0 to 0)	0 (0 to 0.01)	0 (0 to 0)	0 (0 to 0)	0 (0 to 0)	0.01 (0 to 0.02)
Paraguay	Both	60 (43 to 80)	1.09 (0.79 to 1.45)	25 (14 to 39)	0.45 (0.25 to 0.68)	1 (0 to 2)	0.02 (0.01 to 0.04)	4 (1 to 7)	0.06 (0.03 to 0.12)
	Male	57 (41 to 76)	2.16 (1.58 to 2.87)	25 (14 to 38)	0.91 (0.5 to 1.38)	1 (0 to 2)	0.05 (0.02 to 0.09)	3 (1 to 7)	0.12 (0.05 to 0.23)
	Female	3 (2 to 4)	0.11 (0.07 to 0.16)	1 (0 to 1)	0.02 (0.01 to 0.03)	0 (0 to 0)	0 (0 to 0.01)	0 (0 to 0)	0.01 (0 to 0.01)
Peru	Both	39 (22 to 65)	0.12 (0.07 to 0.21)	28 (12 to 48)	0.09 (0.04 to 0.15)	4 (1 to 7)	0.01 (0 to 0.02)	6 (2 to 12)	0.02 (0.01 to 0.04)
	Male	37 (21 to 61)	0.25 (0.14 to 0.41)	25 (11 to 44)	0.17 (0.07 to 0.29)	3 (1 to 6)	0.02 (0.01 to 0.04)	5 (2 to 10)	0.03 (0.01 to 0.06)
	Female	2 (1 to 4)	0.01 (0.01 to 0.02)	2 (0 to 4)	0.01 (0 to 0.03)	0 (0 to 1)	0 (0 to 0.01)	1 (0 to 2)	0.01 (0 to 0.01)
Philippines	Both	471 (352 to 612)	0.62 (0.47 to 0.8)	161 (91 to 245)	0.2 (0.11 to 0.3)	5 (2 to 8)	0.01 (0 to 0.01)	25 (10 to 49)	0.03 (0.01 to 0.06)
	Male	429 (318 to 563)	1.24 (0.93 to 1.61)	151 (86 to 229)	0.4 (0.22 to 0.61)	4 (2 to 8)	0.02 (0.01 to 0.03)	22 (8 to 43)	0.05 (0.02 to 0.1)
	Female	41 (27 to 57)	0.11 (0.08 to 0.15)	11 (5 to 18)	0.03 (0.01 to 0.04)	0 (0 to 1)	0 (0 to 0)	4 (1 to 7)	0.01 (0 to 0.02)
Poland	Both	1330 (1030 to 1700)	1.97 (1.51 to 2.52)	624 (365 to 884)	0.93 (0.54 to 1.33)	80 (39 to 136)	0.11 (0.05 to 0.19)	40 (9 to 90)	0.06 (0.01 to 0.14)
	Male	1220 (923 to 1590)	4.09 (3.09 to 5.3)	611 (358 to 866)	2.05 (1.2 to 2.91)	78 (36 to 132)	0.25 (0.12 to 0.43)	36 (8 to 82)	0.12 (0.03 to 0.27)
	Female	109 (77 to 144)	0.29 (0.21 to 0.39)	13 (3 to 26)	0.04 (0.01 to 0.07)	3 (1 to 5)	0.01 (0 to 0.01)	4 (1 to 8)	0.01 (0 to 0.02)
Portugal	Both	253 (209 to 293)	1.23 (1.02 to 1.42)	145 (92 to 189)	0.7 (0.44 to 0.91)	16 (8 to 25)	0.06 (0.03 to 0.1)	9 (2 to 19)	0.05 (0.01 to 0.1)
	Male	249 (205 to 288)	2.65 (2.2 to 3.06)	142 (91 to 185)	1.51 (0.96 to 1.96)	15 (7 to 25)	0.14 (0.07 to 0.23)	8 (2 to 19)	0.09 (0.02 to 0.21)
	Female	4 (3 to 6)	0.04 (0.03 to 0.06)	3 (2 to 5)	0.02 (0.01 to 0.04)	0 (0 to 0)	0 (0 to 0)	0 (0 to 1)	0 (0 to 0.01)
Puerto Rico	Both	35 (24 to 48)	0.5 (0.34 to 0.69)	12 (6 to 18)	0.18 (0.09 to 0.27)	2 (1 to 3)	0.02 (0.01 to 0.04)	1 (0 to 2)	0.01 (0 to 0.03)
	Male	33 (22 to 45)	1.05 (0.72 to 1.44)	11 (6 to 17)	0.38 (0.2 to 0.57)	2 (1 to 3)	0.05 (0.02 to 0.1)	1 (0 to 2)	0.03 (0.01 to 0.06)
	Female	3 (2 to 4)	0.06 (0.04 to 0.1)	0 (0 to 1)	0.01 (0 to 0.02)	0 (0 to 0)	0 (0 to 0)	0 (0 to 0)	0 (0 to 0)

Qatar	Both	9 (6 to 13)	1.91 (1.15 to 2.69)	0 (0 to 1)	0.05 (0.01 to 0.1)	0 (0 to 0)	0.03 (0.01 to 0.07)	0 (0 to 1)	0.04 (0.01 to 0.08)
	Male	9 (6 to 13)	2.5 (1.51 to 3.53)	0 (0 to 1)	0.07 (0.01 to 0.13)	0 (0 to 0)	0.04 (0.01 to 0.09)	0 (0 to 1)	0.05 (0.02 to 0.11)
	Female	0 (0 to 0)	0.02 (0.01 to 0.04)	0 (0 to 0)	0 (0 to 0)	0 (0 to 0)	0 (0 to 0)	0 (0 to 0)	0 (0 to 0.01)
South Korea	Both	406 (333 to 482)	0.45 (0.37 to 0.54)	161 (97 to 223)	0.18 (0.11 to 0.25)	13 (6 to 22)	0.02 (0.01 to 0.03)	11 (2 to 24)	0.01 (0 to 0.03)
	Male	384 (317 to 455)	1.04 (0.86 to 1.24)	151 (91 to 209)	0.4 (0.24 to 0.55)	13 (6 to 22)	0.04 (0.02 to 0.07)	10 (2 to 22)	0.02 (0.01 to 0.05)
	Female	22 (14 to 30)	0.04 (0.03 to 0.06)	10 (5 to 15)	0.02 (0.01 to 0.03)	0 (0 to 1)	0 (0 to 0)	1 (0 to 2)	0 (0 to 0)
Moldova	Both	114 (94 to 135)	1.96 (1.61 to 2.3)	52 (32 to 70)	0.89 (0.56 to 1.2)	1 (0 to 1)	0.01 (0.01 to 0.02)	2 (0 to 4)	0.03 (0.01 to 0.07)
	Male	113 (93 to 134)	4.51 (3.71 to 5.3)	51 (32 to 68)	2.02 (1.26 to 2.71)	1 (0 to 1)	0.03 (0.01 to 0.05)	2 (0 to 4)	0.07 (0.01 to 0.15)
	Female	1 (1 to 1)	0.03 (0.02 to 0.04)	1 (0 to 1)	0.03 (0.01 to 0.04)	0 (0 to 0)	0 (0 to 0)	0 (0 to 0)	0 (0 to 0.01)
Romania	Both	764 (596 to 944)	2.27 (1.77 to 2.81)	384 (229 to 526)	1.15 (0.69 to 1.58)	14 (6 to 24)	0.04 (0.02 to 0.07)	27 (6 to 61)	0.08 (0.02 to 0.19)
	Male	741 (579 to 918)	4.8 (3.73 to 5.96)	375 (225 to 513)	2.44 (1.48 to 3.35)	13 (6 to 24)	0.08 (0.04 to 0.15)	25 (6 to 57)	0.17 (0.04 to 0.38)
	Female	23 (16 to 31)	0.13 (0.09 to 0.18)	8 (4 to 14)	0.05 (0.02 to 0.07)	1 (0 to 1)	0 (0 to 0.01)	1 (0 to 3)	0.01 (0 to 0.02)
Russia	Both	3240 (2630 to 3950)	1.38 (1.12 to 1.68)	1390 (818 to 1910)	0.59 (0.35 to 0.82)	82 (41 to 137)	0.03 (0.02 to 0.06)	76 (17 to 171)	0.03 (0.01 to 0.07)
	Male	3180 (2580 to 3880)	3.41 (2.78 to 4.15)	1350 (796 to 1870)	1.43 (0.84 to 1.99)	78 (38 to 133)	0.09 (0.04 to 0.15)	71 (16 to 161)	0.07 (0.02 to 0.16)
	Female	59 (40 to 83)	0.05 (0.03 to 0.06)	37 (16 to 59)	0.03 (0.01 to 0.04)	4 (2 to 7)	0 (0 to 0)	5 (1 to 11)	0 (0 to 0.01)
Rwanda	Both	47 (33 to 66)	0.79 (0.57 to 1.11)	22 (12 to 34)	0.35 (0.18 to 0.53)	1 (0 to 4)	0.02 (0.01 to 0.09)	3 (1 to 6)	0.04 (0.02 to 0.08)
	Male	39 (28 to 57)	1.61 (1.13 to 2.31)	20 (11 to 31)	0.75 (0.4 to 1.13)	1 (0 to 4)	0.05 (0.01 to 0.2)	2 (1 to 5)	0.07 (0.03 to 0.15)
	Female	7 (5 to 10)	0.22 (0.15 to 0.31)	2 (1 to 4)	0.07 (0.03 to 0.11)	0 (0 to 1)	0.01 (0 to 0.02)	1 (0 to 2)	0.02 (0.01 to 0.04)
Saint Kitts and Nevis	Both	1 (0 to 1)	0.78 (0.56 to 1)	0 (0 to 0)	0.21 (0 to 0.49)	0 (0 to 0)	0.15 (0.08 to 0.24)	0 (0 to 0)	0.06 (0.03 to 0.12)
	Male	1 (0 to 1)	1.63 (1.18 to 2.1)	0 (0 to 0)	0.42 (0 to 1.01)	0 (0 to 0)	0.33 (0.17 to 0.53)	0 (0 to 0)	0.12 (0.05 to 0.23)
	Female	0 (0 to 0)	0.01 (0.01 to 0.02)	0 (0 to 0)	0 (0 to 0.01)	0 (0 to 0)	0 (0 to 0)	0 (0 to 0)	0 (0 to 0)

Saint Lucia	Both	3 (2 to 3)	1.16 (0.88 to 1.49)	1 (1 to 2)	0.55 (0.31 to 0.79)	0 (0 to 0)	0.03 (0.01 to 0.04)	0 (0 to 0)	0.07 (0.03 to 0.12)
	Male	2 (2 to 3)	2.39 (1.81 to 3.08)	1 (1 to 2)	1.13 (0.63 to 1.61)	0 (0 to 0)	0.06 (0.03 to 0.1)	0 (0 to 0)	0.13 (0.05 to 0.24)
	Female	0 (0 to 0)	0.07 (0.04 to 0.11)	0 (0 to 0)	0.03 (0.01 to 0.05)	0 (0 to 0)	0 (0 to 0.01)	0 (0 to 0)	0.01 (0 to 0.02)
Saint Vincent and the Grenadines	Both	2 (1 to 2)	1.39 (1.04 to 1.79)	1 (1 to 1)	0.72 (0.41 to 1)	0 (0 to 0)	0.06 (0.03 to 0.09)	0 (0 to 0)	0.1 (0.04 to 0.18)
	Male	2 (1 to 2)	2.68 (1.98 to 3.43)	1 (1 to 1)	1.38 (0.78 to 1.93)	0 (0 to 0)	0.11 (0.06 to 0.19)	0 (0 to 0)	0.18 (0.07 to 0.34)
	Female	0 (0 to 0)	0.05 (0.03 to 0.07)	0 (0 to 0)	0.03 (0.01 to 0.04)	0 (0 to 0)	0 (0 to 0)	0 (0 to 0)	0.01 (0 to 0.01)
Samoa	Both	1 (1 to 1)	0.53 (0.43 to 0.66)	0 (0 to 0)	0.07 (0.02 to 0.13)	0 (0 to 0)	0.02 (0.01 to 0.03)	0 (0 to 0)	0.01 (0 to 0.02)
	Male	1 (0 to 1)	0.96 (0.76 to 1.18)	0 (0 to 0)	0.15 (0.04 to 0.26)	0 (0 to 0)	0.03 (0.01 to 0.05)	0 (0 to 0)	0.02 (0.01 to 0.03)
	Female	0 (0 to 0)	0.17 (0.11 to 0.24)	0 (0 to 0)	0 (0 to 0.01)	0 (0 to 0)	0 (0 to 0.01)	0 (0 to 0)	0.01 (0 to 0.01)
San Marino	Both	1 (0 to 1)	0.96 (0.58 to 1.43)	0 (0 to 0)	0.43 (0.01 to 0.78)	0 (0 to 0)	0.15 (0.06 to 0.29)	0 (0 to 0)	0.03 (0.01 to 0.08)
	Male	1 (0 to 1)	1.96 (1.2 to 2.94)	0 (0 to 0)	0.9 (0.02 to 1.6)	0 (0 to 0)	0.33 (0.14 to 0.63)	0 (0 to 0)	0.06 (0.01 to 0.15)
	Female	0 (0 to 0)	0.05 (0.03 to 0.07)	0 (0 to 0)	0.02 (0 to 0.04)	0 (0 to 0)	0 (0 to 0.01)	0 (0 to 0)	0 (0 to 0)
São Tome and Príncipe	Both	0 (0 to 1)	0.36 (0.24 to 0.53)	0 (0 to 0)	0.26 (0.14 to 0.38)	0 (0 to 0)	0.01 (0 to 0.02)	0 (0 to 0)	0.01 (0.01 to 0.03)
	Male	0 (0 to 1)	0.76 (0.49 to 1.1)	0 (0 to 0)	0.52 (0.28 to 0.77)	0 (0 to 0)	0.02 (0.01 to 0.05)	0 (0 to 0)	0.03 (0.01 to 0.06)
	Female	0 (0 to 0)	0.01 (0.01 to 0.02)	0 (0 to 0)	0.02 (0.01 to 0.03)	0 (0 to 0)	0 (0 to 0)	0 (0 to 0)	0 (0 to 0)
Saudi Arabia	Both	73 (53 to 98)	0.4 (0.3 to 0.53)	2 (0 to 4)	0.01 (0 to 0.02)	0 (0 to 0)	0 (0 to 0)	3 (1 to 7)	0.01 (0.01 to 0.03)
	Male	71 (52 to 96)	0.65 (0.48 to 0.87)	2 (0 to 4)	0.01 (0 to 0.03)	0 (0 to 0)	0 (0 to 0.01)	3 (1 to 7)	0.02 (0.01 to 0.05)
	Female	2 (1 to 3)	0.02 (0.01 to 0.04)	0 (0 to 0)	0 (0 to 0)	0 (0 to 0)	0 (0 to 0)	0 (0 to 0)	0 (0 to 0)
Senegal	Both	33 (21 to 48)	0.43 (0.29 to 0.63)	2 (1 to 4)	0.03 (0.01 to 0.05)	1 (0 to 2)	0.01 (0.01 to 0.03)	2 (1 to 4)	0.02 (0.01 to 0.05)
	Male	32 (21 to 47)	0.89 (0.6 to 1.28)	2 (1 to 4)	0.05 (0.01 to 0.1)	1 (0 to 2)	0.03 (0.01 to 0.06)	2 (1 to 4)	0.05 (0.02 to 0.09)
	Female	0 (0 to 0)	0.01 (0 to 0.01)	0 (0 to 0)	0 (0 to 0)	0 (0 to 0)	0 (0 to 0)	0 (0 to 0)	0 (0 to 0.01)

Serbia	Both	355 (268 to 457)	2.27 (1.72 to 2.91)	144 (83 to 207)	0.93 (0.54 to 1.35)	9 (4 to 17)	0.05 (0.02 to 0.1)	10 (2 to 23)	0.07 (0.01 to 0.15)
	Male	330 (248 to 426)	4.54 (3.43 to 5.84)	140 (81 to 202)	1.95 (1.13 to 2.81)	9 (4 to 16)	0.11 (0.05 to 0.21)	9 (2 to 21)	0.13 (0.03 to 0.3)
	Female	25 (18 to 34)	0.31 (0.22 to 0.41)	3 (1 to 6)	0.04 (0.02 to 0.07)	0 (0 to 1)	0 (0 to 0.01)	1 (0 to 2)	0.01 (0 to 0.02)
Seychelles	Both	4 (3 to 5)	3.76 (3.06 to 4.58)	2 (1 to 2)	1.32 (0.72 to 1.94)	0 (0 to 0)	0.06 (0.03 to 0.12)	0 (0 to 0)	0.14 (0.06 to 0.28)
	Male	4 (3 to 5)	8.17 (6.63 to 9.9)	1 (1 to 2)	2.77 (1.51 to 4.06)	0 (0 to 0)	0.15 (0.06 to 0.3)	0 (0 to 0)	0.27 (0.1 to 0.52)
	Female	0 (0 to 0)	0.14 (0.09 to 0.21)	0 (0 to 0)	0.03 (0.01 to 0.06)	0 (0 to 0)	0 (0 to 0.01)	0 (0 to 0)	0.02 (0.01 to 0.03)
Sierra Leone	Both	20 (13 to 28)	0.56 (0.37 to 0.81)	8 (4 to 14)	0.24 (0.12 to 0.39)	0 (0 to 1)	0.01 (0 to 0.02)	1 (0 to 2)	0.02 (0.01 to 0.05)
	Male	19 (12 to 28)	1.11 (0.73 to 1.58)	8 (4 to 13)	0.45 (0.22 to 0.75)	0 (0 to 1)	0.02 (0.01 to 0.04)	1 (0 to 2)	0.04 (0.02 to 0.08)
	Female	0 (0 to 1)	0.02 (0.01 to 0.04)	0 (0 to 1)	0.03 (0.01 to 0.05)	0 (0 to 0)	0 (0 to 0)	0 (0 to 0)	0.01 (0 to 0.01)
Singapore	Both	20 (15 to 24)	0.26 (0.2 to 0.31)	3 (1 to 5)	0.03 (0.01 to 0.06)	2 (1 to 4)	0.03 (0.02 to 0.05)	1 (0 to 1)	0.01 (0 to 0.02)
	Male	18 (15 to 22)	0.52 (0.4 to 0.64)	3 (1 to 5)	0.07 (0.02 to 0.12)	2 (1 to 3)	0.07 (0.04 to 0.12)	1 (0 to 1)	0.01 (0 to 0.03)
	Female	1 (1 to 2)	0.03 (0.02 to 0.04)	0 (0 to 0)	0 (0 to 0.01)	0 (0 to 0)	0 (0 to 0)	0 (0 to 0)	0 (0 to 0)
Slovakia	Both	130 (95 to 170)	1.42 (1.03 to 1.87)	68 (41 to 98)	0.75 (0.45 to 1.08)	3 (1 to 5)	0.03 (0.01 to 0.05)	5 (1 to 11)	0.05 (0.01 to 0.12)
	Male	126 (92 to 165)	3.09 (2.27 to 4.04)	67 (40 to 96)	1.65 (0.99 to 2.37)	2 (1 to 5)	0.06 (0.03 to 0.12)	4 (1 to 10)	0.11 (0.02 to 0.25)
	Female	4 (2 to 5)	0.08 (0.05 to 0.11)	1 (0 to 2)	0.02 (0.01 to 0.04)	0 (0 to 0)	0 (0 to 0)	0 (0 to 0)	0 (0 to 0.01)
Slovenia	Both	38 (27 to 52)	0.94 (0.68 to 1.29)	14 (5 to 23)	0.34 (0.13 to 0.56)	5 (2 to 8)	0.1 (0.05 to 0.19)	1 (0 to 3)	0.03 (0.01 to 0.07)
	Male	35 (25 to 48)	1.87 (1.34 to 2.56)	14 (5 to 22)	0.73 (0.28 to 1.18)	4 (2 to 8)	0.23 (0.1 to 0.42)	1 (0 to 3)	0.06 (0.01 to 0.14)
	Female	3 (2 to 4)	0.15 (0.09 to 0.21)	0 (0 to 1)	0.02 (0 to 0.05)	0 (0 to 0)	0.01 (0 to 0.01)	0 (0 to 0)	0.01 (0 to 0.01)
Solomon Islands	Both	3 (2 to 3)	0.88 (0.6 to 1.17)	0 (0 to 1)	0.07 (0 to 0.15)	0 (0 to 0)	0.03 (0.01 to 0.06)	0 (0 to 0)	0.04 (0.02 to 0.08)
	Male	2 (1 to 3)	1.5 (0.97 to 2)	0 (0 to 1)	0.13 (0 to 0.29)	0 (0 to 0)	0.06 (0.02 to 0.12)	0 (0 to 0)	0.05 (0.02 to 0.11)
	Female	0 (0 to 1)	0.24 (0.14 to 0.34)	0 (0 to 0)	0 (0 to 0.01)	0 (0 to 0)	0.01 (0 to 0.01)	0 (0 to 0)	0.03 (0.01 to 0.06)

Somalia	Both	36 (21 to 54)	0.51 (0.31 to 0.77)	0 (0 to 0)	0 (0 to 0)	1 (0 to 4)	0.02 (0 to 0.07)	4 (2 to 9)	0.05 (0.02 to 0.1)
	Male	34 (20 to 51)	1.14 (0.7 to 1.7)	0 (0 to 0)	0 (0 to 0)	1 (0 to 3)	0.03 (0 to 0.16)	3 (1 to 7)	0.09 (0.03 to 0.18)
	Female	2 (1 to 4)	0.06 (0.03 to 0.11)	0 (0 to 0)	0 (0 to 0)	0 (0 to 1)	0.01 (0 to 0.02)	1 (0 to 2)	0.02 (0.01 to 0.05)
South Africa	Both	346 (278 to 413)	0.76 (0.61 to 0.9)	159 (96 to 218)	0.34 (0.21 to 0.47)	44 (24 to 65)	0.11 (0.06 to 0.16)	9 (3 to 17)	0.02 (0.01 to 0.03)
	Male	319 (255 to 378)	1.69 (1.35 to 2.01)	149 (91 to 203)	0.77 (0.46 to 1.05)	41 (22 to 62)	0.27 (0.15 to 0.4)	8 (3 to 15)	0.04 (0.01 to 0.07)
	Female	27 (20 to 36)	0.11 (0.08 to 0.14)	10 (5 to 15)	0.04 (0.02 to 0.06)	3 (1 to 4)	0.01 (0 to 0.02)	1 (0 to 2)	0 (0 to 0.01)
South Sudan	Both	14 (8 to 23)	0.38 (0.22 to 0.6)	1 (0 to 2)	0.02 (-0.01 to 0.05)	0 (0 to 2)	0.01 (0 to 0.05)	1 (0 to 3)	0.03 (0.01 to 0.06)
	Male	14 (8 to 22)	0.68 (0.4 to 1.08)	1 (0 to 2)	0.03 (-0.01 to 0.09)	0 (0 to 2)	0.01 (0 to 0.1)	1 (0 to 2)	0.04 (0.01 to 0.09)
	Female	1 (0 to 1)	0.04 (0.02 to 0.07)	0 (0 to 0)	0 (0 to 0.01)	0 (0 to 0)	0.01 (0 to 0.01)	0 (0 to 1)	0.01 (0 to 0.03)
Spain	Both	1110 (934 to 1270)	1.23 (1.05 to 1.41)	469 (287 to 630)	0.53 (0.33 to 0.71)	120 (61 to 194)	0.12 (0.06 to 0.19)	24 (5 to 53)	0.03 (0.01 to 0.07)
	Male	1070 (902 to 1230)	2.61 (2.22 to 2.98)	457 (281 to 612)	1.12 (0.69 to 1.5)	118 (59 to 193)	0.26 (0.13 to 0.43)	23 (5 to 51)	0.06 (0.01 to 0.13)
	Female	35 (25 to 45)	0.08 (0.06 to 0.1)	13 (6 to 20)	0.03 (0.01 to 0.04)	2 (1 to 3)	0 (0 to 0.01)	1 (0 to 2)	0 (0 to 0.01)
Sri Lanka	Both	89 (57 to 127)	0.35 (0.23 to 0.5)	37 (19 to 59)	0.14 (0.07 to 0.22)	3 (1 to 6)	0.01 (0.01 to 0.02)	6 (2 to 11)	0.02 (0.01 to 0.04)
	Male	85 (56 to 121)	0.77 (0.51 to 1.08)	35 (18 to 56)	0.3 (0.16 to 0.48)	3 (1 to 5)	0.03 (0.01 to 0.06)	5 (2 to 10)	0.04 (0.01 to 0.08)
	Female	4 (2 to 7)	0.03 (0.02 to 0.05)	1 (1 to 2)	0.01 (0 to 0.02)	0 (0 to 1)	0 (0 to 0.01)	1 (0 to 2)	0.01 (0 to 0.01)
Sudan	Both	218 (143 to 301)	1.23 (0.81 to 1.7)	0 (0 to 1)	0 (0 to 0.01)	1 (0 to 4)	0.01 (0 to 0.02)	11 (4 to 20)	0.05 (0.02 to 0.1)
	Male	209 (137 to 291)	2.18 (1.43 to 3.03)	0 (0 to 1)	0 (0 to 0.01)	1 (0 to 3)	0.01 (0 to 0.04)	10 (4 to 18)	0.09 (0.03 to 0.16)
	Female	9 (5 to 14)	0.1 (0.06 to 0.17)	0 (0 to 0)	0 (0 to 0)	0 (0 to 0)	0 (0 to 0)	1 (0 to 2)	0.01 (0 to 0.02)
Suriname	Both	3 (2 to 4)	0.54 (0.4 to 0.7)	1 (1 to 2)	0.17 (0.09 to 0.26)	0 (0 to 0)	0.01 (0 to 0.02)	0 (0 to 0)	0.03 (0.01 to 0.05)
	Male	3 (2 to 4)	1.1 (0.82 to 1.43)	1 (1 to 2)	0.36 (0.19 to 0.54)	0 (0 to 0)	0.02 (0.01 to 0.04)	0 (0 to 0)	0.06 (0.02 to 0.1)
	Female	0 (0 to 0)	0.07 (0.05 to 0.11)	0 (0 to 0)	0.02 (0.01 to 0.03)	0 (0 to 0)	0 (0 to 0)	0 (0 to 0)	0 (0 to 0.01)

Sweden	Both	49 (39 to 58)	0.23 (0.18 to 0.27)	22 (13 to 31)	0.11 (0.06 to 0.15)	10 (5 to 14)	0.04 (0.02 to 0.06)	1 (0 to 3)	0.01 (0 to 0.01)
	Male	40 (31 to 48)	0.4 (0.31 to 0.48)	19 (11 to 26)	0.19 (0.11 to 0.26)	9 (5 to 14)	0.08 (0.05 to 0.12)	1 (0 to 2)	0.01 (0 to 0.02)
	Female	9 (7 to 11)	0.08 (0.07 to 0.1)	4 (2 to 5)	0.03 (0.02 to 0.05)	0 (0 to 1)	0 (0 to 0.01)	0 (0 to 0)	0 (0 to 0.01)
Switzerland	Both	77 (65 to 89)	0.45 (0.38 to 0.51)	35 (22 to 48)	0.21 (0.13 to 0.28)	16 (9 to 24)	0.08 (0.05 to 0.13)	2 (1 to 5)	0.01 (0 to 0.03)
	Male	66 (56 to 76)	0.83 (0.7 to 0.96)	30 (19 to 41)	0.39 (0.24 to 0.52)	15 (9 to 23)	0.19 (0.1 to 0.28)	2 (0 to 4)	0.03 (0.01 to 0.06)
	Female	11 (9 to 14)	0.12 (0.09 to 0.15)	5 (3 to 7)	0.05 (0.03 to 0.08)	1 (0 to 1)	0.01 (0 to 0.01)	0 (0 to 1)	0 (0 to 0.01)
Syria	Both	71 (52 to 97)	0.63 (0.47 to 0.84)	3 (1 to 5)	0.02 (0.01 to 0.04)	0 (0 to 1)	0 (0 to 0.01)	3 (1 to 5)	0.02 (0.01 to 0.04)
	Male	68 (50 to 93)	1.13 (0.84 to 1.53)	3 (1 to 5)	0.04 (0.02 to 0.08)	0 (0 to 1)	0.01 (0 to 0.02)	3 (1 to 5)	0.04 (0.01 to 0.07)
	Female	3 (2 to 5)	0.06 (0.03 to 0.09)	0 (0 to 0)	0 (0 to 0)	0 (0 to 0)	0 (0 to 0)	0 (0 to 0)	0 (0 to 0)
Taiwan (Province of China)	Both	204 (155 to 273)	0.51 (0.39 to 0.69)	59 (32 to 91)	0.15 (0.08 to 0.23)	4 (2 to 7)	0.01 (0 to 0.02)	10 (4 to 20)	0.03 (0.01 to 0.05)
	Male	203 (154 to 271)	1.1 (0.84 to 1.47)	59 (32 to 90)	0.32 (0.17 to 0.48)	4 (1 to 7)	0.02 (0.01 to 0.04)	10 (4 to 19)	0.05 (0.02 to 0.1)
	Female	1 (1 to 2)	0.01 (0 to 0.01)	0 (0 to 1)	0 (0 to 0)	0 (0 to 1)	0 (0 to 0)	1 (0 to 1)	0 (0 to 0.01)
Tajikistan	Both	25 (18 to 39)	0.52 (0.38 to 0.8)	5 (2 to 9)	0.09 (0.04 to 0.16)	0 (0 to 1)	0.01 (0 to 0.02)	1 (0 to 2)	0.02 (0.01 to 0.04)
	Male	24 (17 to 37)	1.08 (0.77 to 1.64)	5 (2 to 8)	0.17 (0.08 to 0.3)	0 (0 to 1)	0.01 (0.01 to 0.03)	1 (0 to 2)	0.03 (0.01 to 0.06)
	Female	1 (1 to 2)	0.04 (0.03 to 0.07)	0 (0 to 1)	0.01 (0 to 0.02)	0 (0 to 0)	0 (0 to 0.01)	0 (0 to 1)	0.01 (0 to 0.02)
Thailand	Both	817 (585 to 1110)	0.8 (0.57 to 1.07)	331 (181 to 506)	0.32 (0.18 to 0.48)	18 (7 to 35)	0.02 (0.01 to 0.04)	41 (16 to 78)	0.04 (0.01 to 0.07)
	Male	797 (569 to 1080)	1.74 (1.25 to 2.33)	326 (178 to 499)	0.69 (0.38 to 1.04)	18 (7 to 35)	0.04 (0.02 to 0.08)	37 (14 to 72)	0.07 (0.03 to 0.14)
	Female	20 (12 to 32)	0.04 (0.02 to 0.06)	6 (2 to 10)	0.01 (0 to 0.02)	0 (0 to 0)	0 (0 to 0)	4 (1 to 7)	0.01 (0 to 0.01)
Timor-Leste	Both	5 (3 to 7)	0.65 (0.43 to 0.93)	1 (0 to 2)	0.14 (0.05 to 0.24)	0 (0 to 0)	0.01 (0 to 0.02)	0 (0 to 0)	0.03 (0.01 to 0.05)
	Male	5 (3 to 7)	1.24 (0.82 to 1.79)	1 (0 to 2)	0.27 (0.11 to 0.48)	0 (0 to 0)	0.02 (0.01 to 0.05)	0 (0 to 0)	0.04 (0.02 to 0.09)
	Female	0 (0 to 0)	0.06 (0.03 to 0.1)	0 (0 to 0)	0.01 (0 to 0.02)	0 (0 to 0)	0 (0 to 0)	0 (0 to 0)	0.01 (0 to 0.03)

Togo	Both	26 (18 to 35)	0.73 (0.5 to 0.98)	7 (3 to 12)	0.18 (0.08 to 0.31)	0 (0 to 1)	0.01 (0.01 to 0.03)	1 (1 to 3)	0.03 (0.01 to 0.06)
	Male	25 (17 to 34)	1.72 (1.2 to 2.33)	7 (3 to 12)	0.42 (0.17 to 0.7)	0 (0 to 1)	0.03 (0.01 to 0.07)	1 (0 to 3)	0.06 (0.02 to 0.13)
	Female	1 (0 to 1)	0.04 (0.02 to 0.06)	0 (0 to 1)	0.01 (0 to 0.03)	0 (0 to 0)	0 (0 to 0)	0 (0 to 0)	0.01 (0 to 0.01)
Tokelau	Both	0 (0 to 0)	0.34 (0.25 to 0.45)	0 (0 to 0)	0.06 (0.02 to 0.11)	0 (0 to 0)	0.01 (0 to 0.02)	0 (0 to 0)	0.01 (0 to 0.02)
	Male	0 (0 to 0)	0.58 (0.42 to 0.76)	0 (0 to 0)	0.12 (0.04 to 0.21)	0 (0 to 0)	0.02 (0.01 to 0.03)	0 (0 to 0)	0.01 (0.01 to 0.03)
	Female	0 (0 to 0)	0.12 (0.07 to 0.18)	0 (0 to 0)	0 (0 to 0.01)	0 (0 to 0)	0 (0 to 0.01)	0 (0 to 0)	0.01 (0 to 0.02)
Tonga	Both	0 (0 to 0)	0.46 (0.35 to 0.6)	0 (0 to 0)	0.04 (0 to 0.08)	0 (0 to 0)	0.01 (0.01 to 0.02)	0 (0 to 0)	0.01 (0 to 0.02)
	Male	0 (0 to 0)	0.96 (0.74 to 1.25)	0 (0 to 0)	0.08 (0 to 0.17)	0 (0 to 0)	0.03 (0.01 to 0.05)	0 (0 to 0)	0.02 (0.01 to 0.03)
	Female	0 (0 to 0)	0.07 (0.04 to 0.11)	0 (0 to 0)	0 (0 to 0)	0 (0 to 0)	0 (0 to 0)	0 (0 to 0)	0.01 (0 to 0.01)
Trinidad and Tobago	Both	10 (7 to 14)	0.54 (0.37 to 0.74)	4 (2 to 6)	0.21 (0.11 to 0.33)	0 (0 to 0)	0.01 (0.01 to 0.03)	1 (0 to 1)	0.03 (0.01 to 0.06)
	Male	10 (7 to 13)	1.1 (0.77 to 1.52)	4 (2 to 6)	0.44 (0.22 to 0.67)	0 (0 to 0)	0.03 (0.01 to 0.06)	1 (0 to 1)	0.06 (0.02 to 0.12)
	Female	0 (0 to 0)	0.03 (0.02 to 0.05)	0 (0 to 0)	0.01 (0 to 0.02)	0 (0 to 0)	0 (0 to 0)	0 (0 to 0)	0 (0 to 0.01)
Tunisia	Both	243 (169 to 341)	1.94 (1.36 to 2.69)	21 (9 to 36)	0.16 (0.07 to 0.27)	1 (0 to 4)	0.01 (0 to 0.04)	8 (3 to 15)	0.06 (0.02 to 0.11)
	Male	240 (167 to 337)	3.99 (2.81 to 5.53)	21 (9 to 36)	0.32 (0.14 to 0.55)	1 (0 to 4)	0.02 (0 to 0.09)	8 (3 to 15)	0.11 (0.04 to 0.23)
	Female	3 (2 to 5)	0.05 (0.03 to 0.07)	0 (0 to 1)	0.01 (0 to 0.01)	0 (0 to 0)	0 (0 to 0)	0 (0 to 0)	0 (0 to 0.01)
Turkey	Both	1080 (815 to 1390)	1.23 (0.94 to 1.58)	88 (42 to 144)	0.1 (0.05 to 0.16)	103 (45 to 176)	0.12 (0.05 to 0.21)	42 (16 to 84)	0.04 (0.02 to 0.09)
	Male	1040 (777 to 1330)	2.55 (1.93 to 3.25)	83 (40 to 136)	0.19 (0.09 to 0.31)	97 (42 to 168)	0.25 (0.11 to 0.44)	39 (15 to 79)	0.09 (0.03 to 0.18)
	Female	49 (32 to 67)	0.1 (0.07 to 0.14)	5 (2 to 9)	0.01 (0 to 0.02)	6 (2 to 12)	0.01 (0.01 to 0.03)	2 (1 to 5)	0.01 (0 to 0.01)
Turkmenistan	Both	21 (16 to 28)	0.52 (0.39 to 0.69)	8 (4 to 11)	0.17 (0.09 to 0.26)	0 (0 to 1)	0.01 (0 to 0.02)	1 (0 to 2)	0.03 (0.01 to 0.05)
	Male	20 (15 to 27)	1.14 (0.84 to 1.51)	7 (4 to 10)	0.37 (0.19 to 0.54)	0 (0 to 0)	0.01 (0.01 to 0.02)	1 (0 to 2)	0.05 (0.02 to 0.09)
	Female	1 (1 to 2)	0.05 (0.03 to 0.08)	0 (0 to 1)	0.02 (0 to 0.04)	0 (0 to 0)	0.01 (0 to 0.02)	0 (0 to 0)	0.01 (0 to 0.02)

Tuvalu	Both	0 (0 to 0)	0.58 (0.4 to 0.79)	0 (0 to 0)	0.06 (0.01 to 0.14)	0 (0 to 0)	0.02 (0.01 to 0.04)	0 (0 to 0)	0.02 (0.01 to 0.04)
	Male	0 (0 to 0)	1.09 (0.73 to 1.51)	0 (0 to 0)	0.14 (0.03 to 0.28)	0 (0 to 0)	0.04 (0.02 to 0.09)	0 (0 to 0)	0.03 (0.01 to 0.07)
	Female	0 (0 to 0)	0.14 (0.09 to 0.22)	0 (0 to 0)	0 (0 to 0.01)	0 (0 to 0)	0 (0 to 0.01)	0 (0 to 0)	0.01 (0 to 0.02)
Uganda	Both	63 (39 to 92)	0.44 (0.28 to 0.64)	60 (34 to 89)	0.4 (0.22 to 0.59)	2 (1 to 10)	0.02 (0.01 to 0.08)	8 (3 to 15)	0.05 (0.02 to 0.09)
	Male	56 (35 to 81)	0.9 (0.58 to 1.28)	52 (30 to 76)	0.78 (0.45 to 1.13)	1 (0 to 9)	0.03 (0.01 to 0.19)	6 (2 to 11)	0.08 (0.03 to 0.15)
	Female	7 (3 to 11)	0.09 (0.05 to 0.15)	8 (4 to 13)	0.1 (0.05 to 0.16)	1 (0 to 1)	0.01 (0 to 0.02)	2 (1 to 4)	0.02 (0.01 to 0.04)
Ukraine	Both	1360 (1030 to 1730)	1.86 (1.4 to 2.37)	541 (301 to 786)	0.75 (0.42 to 1.09)	36 (17 to 63)	0.05 (0.02 to 0.08)	30 (7 to 68)	0.04 (0.01 to 0.1)
	Male	1350 (1020 to 1720)	4.55 (3.45 to 5.77)	532 (296 to 774)	1.79 (0.99 to 2.61)	35 (16 to 61)	0.12 (0.05 to 0.21)	29 (6 to 66)	0.1 (0.02 to 0.22)
	Female	10 (6 to 16)	0.03 (0.02 to 0.04)	8 (4 to 14)	0.02 (0.01 to 0.04)	1 (0 to 3)	0 (0 to 0.01)	1 (0 to 2)	0 (0 to 0.01)
United Arab Emirates	Both	80 (49 to 126)	1.83 (1.22 to 2.61)	11 (3 to 21)	0.16 (0.04 to 0.31)	0 (0 to 1)	0.01 (0 to 0.06)	7 (2 to 14)	0.08 (0.03 to 0.17)
	Male	78 (48 to 123)	2.5 (1.68 to 3.57)	11 (3 to 21)	0.21 (0.06 to 0.41)	0 (0 to 1)	0.01 (0 to 0.08)	6 (2 to 13)	0.11 (0.04 to 0.22)
	Female	2 (1 to 3)	0.15 (0.08 to 0.24)	0 (0 to 0)	0.01 (0 to 0.02)	0 (0 to 0)	0 (0 to 0.01)	0 (0 to 0)	0.01 (0 to 0.02)
United Kingdom	Both	670 (551 to 768)	0.53 (0.44 to 0.61)	266 (152 to 374)	0.22 (0.13 to 0.3)	179 (108 to 252)	0.13 (0.08 to 0.18)	18 (4 to 41)	0.02 (0 to 0.04)
	Male	548 (449 to 629)	0.95 (0.78 to 1.08)	236 (138 to 326)	0.42 (0.24 to 0.57)	166 (96 to 237)	0.27 (0.16 to 0.39)	16 (4 to 35)	0.03 (0.01 to 0.06)
	Female	122 (91 to 143)	0.18 (0.14 to 0.21)	30 (12 to 49)	0.05 (0.02 to 0.08)	14 (5 to 22)	0.02 (0.01 to 0.03)	3 (1 to 6)	0.01 (0 to 0.01)
Tanzania	Both	147 (97 to 216)	0.6 (0.4 to 0.86)	83 (45 to 130)	0.33 (0.18 to 0.5)	4 (1 to 20)	0.02 (0 to 0.1)	13 (5 to 26)	0.05 (0.02 to 0.09)
	Male	133 (89 to 199)	1.14 (0.78 to 1.66)	75 (41 to 115)	0.61 (0.34 to 0.94)	3 (0 to 19)	0.03 (0.01 to 0.2)	10 (4 to 21)	0.07 (0.03 to 0.15)
	Female	14 (8 to 22)	0.11 (0.06 to 0.17)	8 (4 to 14)	0.06 (0.03 to 0.11)	1 (0 to 2)	0.01 (0 to 0.02)	3 (1 to 6)	0.02 (0.01 to 0.04)
Virgin Islands	Both	2 (1 to 3)	1.06 (0.76 to 1.39)	1 (0 to 1)	0.38 (0 to 0.75)	0 (0 to 0)	0.15 (0.07 to 0.26)	0 (0 to 0)	0.07 (0.03 to 0.13)
	Male	2 (1 to 3)	2.27 (1.63 to 2.98)	1 (0 to 1)	0.82 (0 to 1.62)	0 (0 to 0)	0.35 (0.17 to 0.6)	0 (0 to 0)	0.14 (0.05 to 0.27)
	Female	0 (0 to 0)	0.09 (0.06 to 0.13)	0 (0 to 0)	0.03 (0 to 0.06)	0 (0 to 0)	0 (0 to 0.01)	0 (0 to 0)	0.01 (0 to 0.01)

United States of America	Both	3560 (2890 to 4090)	0.64 (0.52 to 0.73)	1200 (694 to 1640)	0.22 (0.13 to 0.3)	407 (223 to 605)	0.07 (0.04 to 0.1)	139 (34 to 304)	0.03 (0.01 to 0.06)
	Male	2910 (2370 to 3330)	1.13 (0.92 to 1.3)	1040 (608 to 1410)	0.41 (0.24 to 0.55)	385 (204 to 583)	0.15 (0.08 to 0.23)	112 (27 to 245)	0.04 (0.01 to 0.1)
	Female	650 (523 to 767)	0.22 (0.17 to 0.25)	164 (84 to 244)	0.06 (0.03 to 0.08)	22 (10 to 36)	0.01 (0 to 0.01)	27 (6 to 60)	0.01 (0 to 0.02)
Uruguay	Both	89 (73 to 105)	1.78 (1.48 to 2.06)	40 (24 to 55)	0.78 (0.47 to 1.08)	5 (2 to 7)	0.08 (0.04 to 0.13)	5 (2 to 9)	0.1 (0.04 to 0.19)
	Male	84 (69 to 98)	3.8 (3.13 to 4.42)	38 (22 to 52)	1.69 (1.01 to 2.31)	4 (2 to 7)	0.19 (0.09 to 0.31)	5 (2 to 8)	0.21 (0.09 to 0.39)
	Female	5 (4 to 7)	0.18 (0.13 to 0.23)	2 (1 to 4)	0.08 (0.04 to 0.11)	0 (0 to 0)	0.01 (0 to 0.01)	0 (0 to 1)	0.01 (0.01 to 0.02)
Uzbekistan	Both	165 (129 to 204)	0.76 (0.61 to 0.93)	46 (22 to 71)	0.18 (0.08 to 0.27)	2 (1 to 4)	0.01 (0.01 to 0.02)	15 (6 to 27)	0.05 (0.02 to 0.1)
	Male	156 (123 to 193)	1.7 (1.35 to 2.08)	42 (22 to 65)	0.36 (0.18 to 0.55)	2 (1 to 3)	0.02 (0.01 to 0.04)	11 (4 to 19)	0.08 (0.03 to 0.15)
	Female	9 (5 to 14)	0.06 (0.03 to 0.1)	4 (0 to 8)	0.03 (0 to 0.06)	1 (0 to 1)	0.01 (0 to 0.01)	4 (2 to 8)	0.03 (0.01 to 0.05)
Vanuatu	Both	1 (1 to 1)	0.49 (0.32 to 0.68)	0 (0 to 0)	0.06 (0.02 to 0.13)	0 (0 to 0)	0.04 (0.01 to 0.07)	0 (0 to 0)	0.02 (0.01 to 0.04)
	Male	1 (0 to 1)	0.89 (0.58 to 1.23)	0 (0 to 0)	0.12 (0.04 to 0.24)	0 (0 to 0)	0.07 (0.02 to 0.13)	0 (0 to 0)	0.03 (0.01 to 0.06)
	Female	0 (0 to 0)	0.04 (0.02 to 0.07)	0 (0 to 0)	0 (0 to 0)	0 (0 to 0)	0 (0 to 0.01)	0 (0 to 0)	0.01 (0 to 0.02)
Venezuela	Both	323 (221 to 444)	1.11 (0.76 to 1.52)	127 (62 to 200)	0.42 (0.2 to 0.67)	6 (2 to 11)	0.02 (0.01 to 0.04)	24 (9 to 47)	0.08 (0.03 to 0.15)
	Male	301 (204 to 412)	2.24 (1.55 to 3.04)	122 (60 to 192)	0.87 (0.42 to 1.36)	5 (2 to 10)	0.04 (0.02 to 0.08)	22 (9 to 43)	0.15 (0.06 to 0.29)
	Female	22 (13 to 35)	0.14 (0.09 to 0.22)	5 (2 to 9)	0.03 (0.01 to 0.06)	1 (0 to 1)	0 (0 to 0.01)	2 (1 to 4)	0.01 (0 to 0.03)
Vietnam	Both	1340 (1020 to 1730)	1.4 (1.07 to 1.77)	595 (339 to 874)	0.61 (0.35 to 0.89)	29 (12 to 54)	0.03 (0.01 to 0.06)	81 (32 to 156)	0.08 (0.03 to 0.14)
	Male	1330 (1010 to 1710)	3.28 (2.53 to 4.13)	591 (337 to 867)	1.41 (0.81 to 2.05)	28 (12 to 54)	0.09 (0.04 to 0.16)	75 (30 to 144)	0.15 (0.06 to 0.29)
	Female	16 (9 to 26)	0.03 (0.02 to 0.05)	4 (1 to 8)	0.01 (0 to 0.02)	0 (0 to 1)	0 (0 to 0)	6 (2 to 12)	0.01 (0 to 0.02)
Yemen	Both	235 (155 to 330)	1.82 (1.23 to 2.56)	8 (3 to 15)	0.05 (0.02 to 0.1)	1 (0 to 4)	0.01 (0 to 0.04)	8 (3 to 17)	0.05 (0.02 to 0.11)
	Male	211 (136 to 304)	3.34 (2.2 to 4.76)	8 (3 to 14)	0.11 (0.04 to 0.2)	1 (0 to 4)	0.02 (0 to 0.08)	8 (3 to 17)	0.11 (0.04 to 0.22)
	Female	24 (15 to 35)	0.35 (0.22 to 0.52)	0 (0 to 1)	0 (0 to 0.01)	0 (0 to 0)	0 (0 to 0.01)	0 (0 to 1)	0 (0 to 0.01)

Zambia	Both	44 (27 to 65)	0.66 (0.42 to 0.95)	21 (10 to 36)	0.28 (0.13 to 0.48)	2 (1 to 6)	0.03 (0.01 to 0.11)	5 (2 to 9)	0.06 (0.02 to 0.12)
	Male	41 (25 to 60)	1.25 (0.8 to 1.81)	19 (10 to 33)	0.53 (0.25 to 0.89)	1 (0 to 5)	0.05 (0.01 to 0.22)	4 (1 to 8)	0.09 (0.04 to 0.19)
	Female	3 (2 to 5)	0.11 (0.07 to 0.18)	2 (1 to 3)	0.05 (0.02 to 0.09)	0 (0 to 1)	0.01 (0 to 0.02)	1 (0 to 2)	0.02 (0.01 to 0.04)
Zimbabwe	Both	101 (77 to 128)	1.41 (1.09 to 1.77)	28 (13 to 45)	0.35 (0.15 to 0.58)	2 (1 to 4)	0.03 (0.02 to 0.06)	4 (2 to 7)	0.04 (0.02 to 0.09)
	Male	94 (72 to 119)	3.12 (2.45 to 3.89)	26 (12 to 43)	0.77 (0.35 to 1.27)	1 (1 to 3)	0.06 (0.03 to 0.12)	3 (1 to 6)	0.08 (0.03 to 0.16)
	Female	7 (4 to 11)	0.21 (0.13 to 0.31)	1 (0 to 3)	0.03 (0.01 to 0.06)	1 (0 to 1)	0.02 (0.01 to 0.04)	1 (0 to 1)	0.02 (0.01 to 0.03)

Figure S1. Annualised rate of change in the age-standardised death rate of larynx cancer attributable to smoking, 2010–2019.

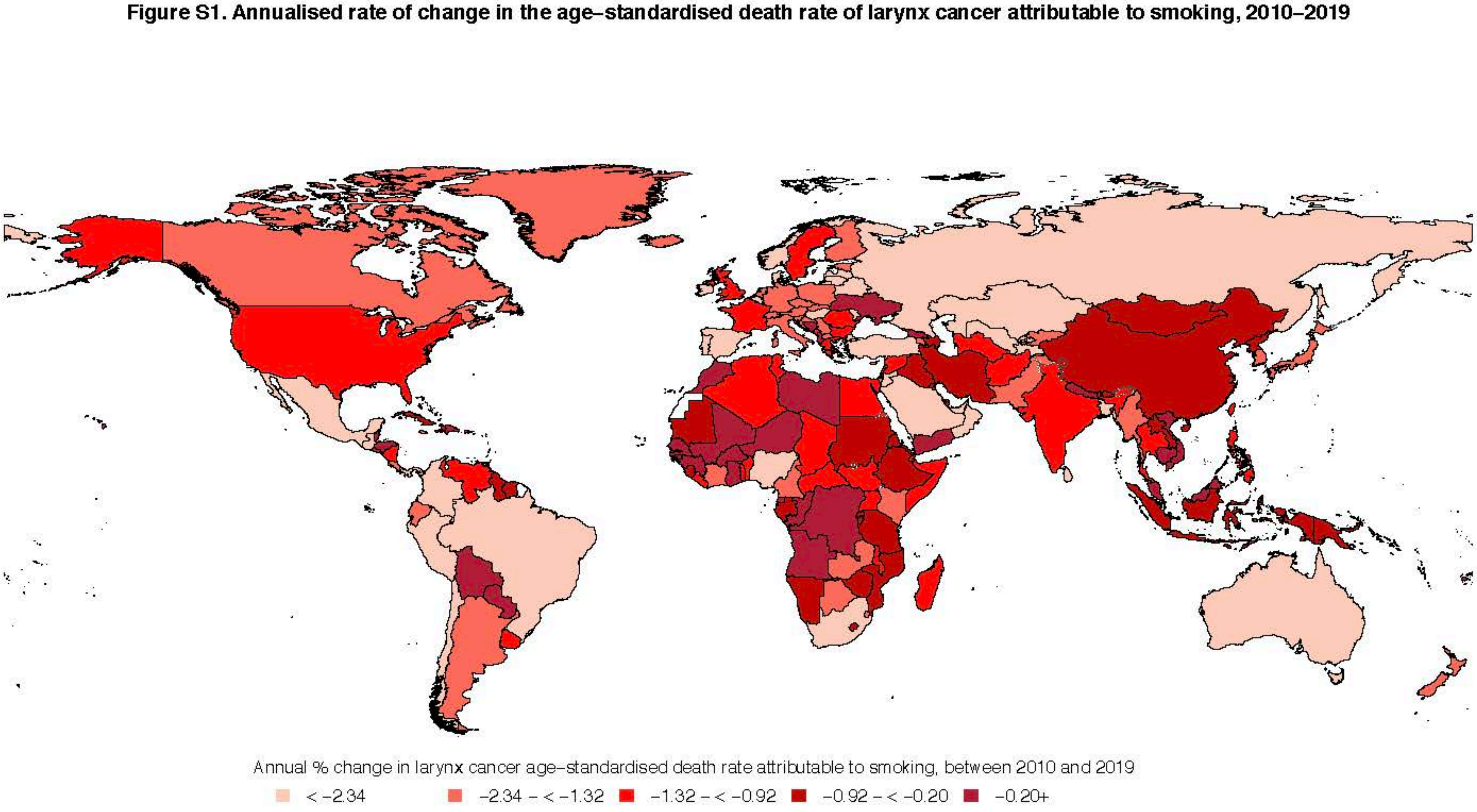

Supplement: Supplementary appendix 2 [file mmc2.pdf]
